# Checklist of the grasses of India

**DOI:** 10.3897/phytokeys.163.38393

**Published:** 2020-10-16

**Authors:** Elizabeth A. Kellogg, J. Richard Abbott, Kamaljit S. Bawa, Kanchi N. Gandhi, B. R. Kailash, K.N. Ganeshaiah, Uttam Babu Shrestha, Peter Raven

**Affiliations:** 1 Donald Danforth Plant Science Center St. Louis United States of America; 2 Missouri Botanical Garden St. Louis United States of America; 3 University of Massachusetts, Boston Boston United States of America; 4 Harvard University Herbaria Cambridge United States of America; 5 5Ashoka Trust for Research in Ecology and the Environment (ATREE) Bangalore India; 6 University of Agricultural Sciences Bangalore India; 7 University of Southern Queensland Toowoomba Australia; 8 Missouri Botanical Garden St. Louis, MO United States of America

**Keywords:** Poaceae, taxonomy, biodiversity, biogeography, south Asia

## Abstract

A checklist of the grasses of India is presented, as compiled from survey of all available literature. Of the twelve subfamilies of grasses, ten are represented in India. Most subfamilies have been examined by taxonomic experts for up-to-date nomenclature. The list includes 1506 species plus infraspecific taxa and presents information on types, synonyms, distribution within India, and habit. Twelve new combinations are made, viz. *Arctopoa tibetica *(Munro ex Stapf) Prob. var. *aristulata *(Stapf) E.A. Kellogg, **comb. nov.**; *Chimonocalamus nagalandianus *(H.B. Naithani) L.G. Clark, **comb. nov.**; *Chionachne digitata *(L.f.) E.A. Kellogg, **comb. nov.**; *Chionachne wallichiana *(Nees) E.A. Kellogg, **comb. nov.**; *Dinebra polystachyo*s (R. Br.) E.A. Kellogg, **comb. nov.**; *Moorochloa eruciformis *(Sm.) Veldkamp var. *divaricata *(Basappa & Muniv.) E.A. Kellogg, **comb. nov.**; *Phyllostachys nigra *(Lodd. ex Lindl.) Munro var. *puberula *(Miq.) Kailash, **comb. & stat. nov.**; *Tzveleviochloa schmidii *(Hook. f.) E.A. Kellogg, **comb. nov.**; *Urochloa lata *(Schumach.) C.E. Hubb. var. *pubescens *(C.E. Hubb.) E.A. Kellogg, **comb. nov.**; *Urochloa ramosa *(L.) T.Q. Nguyen var. *pubescens *(Basappa & Muniy.) E.A. Kellogg, **comb. nov.**; *Urochloa semiundulata *(Hochst. ex A. Rich.) Ashalatha & V.J. Nair var. *intermedia *(Basappa & Muniy.) E.A. Kellogg, **comb. nov.**

## Introduction

The following is a checklist of the grasses of India, produced as part of the Plants of India project. The grass family has been the subject of two major new classifications, Kellogg (2015b) and Soreng et al. (2015, 2017), based on phylogenetic data. Even before these appeared, molecular phylogenies had resolved circumscription of the subfamilies and there has been general agreement for at least the last 15 years on the monophyly of the major subfamilies. Nearly all known genera have been firmly assigned to subfamily and a large majority to tribe. Division into subtribes remains an item of discussion in some groups.

The two current classifications are quite similar, which is not surprising since they are based on the same underlying data and phylogenies. Many of the differences reflect simply different preferences for classification. Kellogg (2015b) generally avoids monogeneric tribes or subtribes on the grounds that they convey no phylogenetic information and are unnecessary. This means that, while all genera are assigned to subfamily, the smaller subfamilies (e.g. Pharoideae) are not divided into tribes. In contrast, Soreng et al. (2015, 2017) prefer consistency in the use of ranks throughout the classification, meaning that all subfamilies include tribes, even if or when the tribes are unnecessary from the point of view of identification or phylogeny. The same arguments apply to subtribes. The Soreng et al. (2015, 2017) classification is thus more split than Kellogg (2015b). In addition, there are a few differences in generic circumscription. Because Kellogg (2015b) appeared in book form, it really represents the state of data a couple of years earlier, whereas Soreng et al. (2017) includes more recent information.

All grass subfamilies are represented in India except for Anomochlooideae, which is found only in the New World, and Puelioideae, which is restricted to Africa. We follow Kellogg (2015b) in using italics in the taxonomic descriptions to indicate putative synapomorphies (shared derived characters) indicating monophyly of the described group. Numbers of tribes and genera are listed in Table 1. This list includes 266 genera, representing about a third of the 711 (Kellogg 2015b) to 768 (Soreng et al. 2017) genera currently recognized in the family.

**Table 1.** The ten subfamilies of grasses represented in the flora of India, showing numbers of tribes, genera, and species recorded in India for each. Tribes are not recognized here for Pharoideae and Aristidoideae because they are redundant, nor for Micrairoideae and Arundinoideae because the number of genera is too small to require tribal designations. Two hundred sixty-six genera have been recorded for the country, representing about 1/3 of the total in the family.

**Subfamily**	**Tribes**	**Genera**	**Species + subspecies + varieties**
Pharoideae	(1, redundant)	1	1
Bambusoideae	2 (of 3)	32	148
Oryzoideae	3 (of 3)	6	29
Pooideae	8 (of 11)	70	383
Aristidoideae	(1, redundant)	2	15
Panicoideae	6 (of 8)	98	668
Danthonioideae	(1, redundant)	3	5
Chloridoideae	5 (of 5)	43	201
Micrairoideae	(4, not recognized here)	7	50
Arundinoideae	(2, not recognized here)	4	6

Many of the tribes and subfamilies have been checked by taxonomic experts and those experts are acknowledged in the appropriate section. Because of in some cases considerable time elapsed between the review of the list and the current publication, recent taxonomic changes may have been missed. Most nomenclatural information is consistent with that in the Tropicos database (http://www.tropicos.org), but the synonymy is not fully consistent with that in GrassBase (Clayton et al. 2006 onwards)

The compilation presented here is meant to be a starting point for future work, which requires detailed mapping and verification with herbarium material. Distributions within India are compiled from a broad range of sources only some of which included specimen citations to support their claims of geographical distribution. All distribution records should thus be considered provisional. Of particular note is the work of Naithani (1990), which added many new distribution records but largely without documentation. When Naithani (1990) is the only source for a record, this is noted in the text. We have avoided use of the term “endemic” since endemism is hard to verify in the absence of extensive herbarium material. Synonyms are ones that have been used in the Indian literature in the past. Some of these are New World names and are included here as a way to be able to follow up older Indian literature.

India is the most poorly studied of all subcontinents for grasses. The country has been largely inaccessible to foreign botanists for the last several decades and the revolution in molecular systematics has largely missed Indian grasses because of the near-impossibility of obtaining material. We present this list in hopes of showing the richness of the grass flora and the discoveries to be made.

## Background and methods

The objective of the overall project is to produce an authenticated and authoritative digital list of vascular plants of India, both native and naturalized. This list is to include all taxa of seed plants with their accepted names, synonyms, author names, publication details, habit, and distribution ranges. It has been a major task to coalesce the available taxonomic information into a unified list of names representing India’s plant diversity. Doing so required a significant amount of effort and time to compile the information in source materials and produce a functional list. Despite its tentative nature, this list contains the best information available for many groups, and is likely to find diverse uses and applications.

The current checklist has resulted from collaboration between the Ashoka Trust for Research in Ecology and the Environment (ATREE), the Missouri Botanical Garden (MBG), and Harvard University Herbaria (HUH). In mid-1990s, prior to the present collaboration, Dr. K.N. Ganeshaiah of the University of Agricultural Sciences, Bangalore and Dr. Sebastian Padayatty of Shaman Scientific, Bangalore, along with Kanchi N. Gandhi of the HUH, were independently compiling data on plant names for a checklist of vascular plants of India. Dr. Peter H. Raven (MBG) and Dr. Kamal Bawa (ATREE) organized a meeting of relevant staff from the two teams at the Missouri Botanical Garden in 2002. At this meeting, it was agreed that a team would compile the available data at ATREE, the nomenclature would be verified at the Harvard University Herbaria, and taxonomic revision would be carried out by specialists under the guidance of Dr. Raven. The Sehgal Family Foundation, a previous donor to both ATREE and MBG, provided the initial funds for preparing a comprehensive list of India’s plants.

National and international botanical journals, floras, manuals, monographs, and revisionary works were reviewed, with the data compiled by a team of energetic staff members under the supervision of the late Dr. S. N. I. Yoganarasimhan (1944–2017). At the HUH, Gandhi received a copy of the data that had been compiled at ATREE and verified the nomenclature over a period almost 6 years (2007–2013). Initially, two assistants, Mr. Uttam B. Shrestha (a Ph.D. student of Dr. Bawa) and Mr. B. R. Kailash (ATREE staff) worked under the supervision of Gandhi; later Uttam’s wife Sujata Shrestha also joined the crew for a few months.

Regardless of the status of a name (accepted or synonym), an effort was made to check each protologue to verify its validity, authorship, bibliography, and legitimacy. The investigation was helpful in correcting errors that had found their way into earlier botanical works. A number of recent monographs and journals were consulted to add new taxa reported for India and to revise taxonomy and ranges of taxa. In the course of this work, type information was added when feasible.

## Poaceae Barnh., 1895, nom. cons.

**Type**. *Poa* L.

Ancestrally rhizomatous perennials but diversified into caespitose or scarcely branched forms; annual habit derived multiple times. Culms herbaceous but woody culms derived more than once. Height varying from a few mm to several m. Leaf blades broad, pseudopetioles synapomorphic for the family but repeatedly lost. Ligule membranous or a fringe of hairs. Inflorescences branched or unbranched. Stamens ancestrally 6, but reduced to 3 in many species. Style branches and stigmas ancestrally 3, but reduced to 2 in many species. Pollen monoporate, *lacking scrobiculi in the exine*. Ovule 1. *Embryo lateral, highly differentiated with clear root and shoot meristems, each enclosed by a sheath, and with several well-developed leaves and a lateral haustorial organ (scutellum)*. Fruit indehiscent with a single seed, *the seed coat tightly appressed or fused to the inner wall of the pericarp*. *Leaf mesophyll with fusoid cells and with cells with invaginated cell walls*, midrib complex; these characters lost during evolution of the family. Epidermis with multicellular microhairs, with alternating long and short cells, the short cells developing silica bodies. Photosynthetic pathway ancestrally C3.

Poaceae is a monophyletic family as documented by decades of phylogenetic studies (e.g., (Campbell and Kellogg 1987; Clark et al. 1995; Grass Phylogeny Working Group 2001; Grass Phylogeny Working Group II 2012; Linder 1987)). Morphological characters that are unique to the family (synapomorphic) include the highly differentiated embryo, the unique fruit structure, and the unique leaf morphology. In all grasses except Anomochlooideae (i.e., in all Indian species), the branches terminate in units known as spikelets, with each spikelet subtended by two bracts (glumes) and bearing one or more flowers. Also in this slightly more restricted group the inner perianth is modified to form tiny hyaline structures known as lodicules that function in flower opening and then wither after anthesis.

### Subfamily Pharoideae L. G. Clark & Judz., 1996

Plants herbaceous, rhizomatous. Leaf blades with a pseudopetiole, *resupinate, with lateral veins running obliquely from midvein to margins*. Inflorescence branched, *with hooked hairs on the branches and spikelets*. Spikelets unisexual, with staminate and pistillate spikelets borne in pairs. Lemma margins fused such that lemmas are tubular and often inflated. Lodicules lacking, or present only in staminate flowers. *Inner bundle sheath of veins with multiple layers of cells. Intercostal epidermis with files of long fibers alternating with normal files of intercostal long cells*.

Subfamily Pharoideae includes only about 12 species, which grow in shade in tropical forests. The subfamily is monophyletic, with obvious morphological and molecular synapomorphies. The spikelets are unisexual and paired, with a large pistillate spikelet associated with a much smaller staminate spikelet. The leaf anatomy is also distinctive in that the inner bundle sheath of the veins has multiple layers of cells (Judziewicz and Clark 2007). The intercostal epidermis has files of long fibers alternating with normal files of intercostal long cells (Soderstrom et al. 1987). It is unclear whether this subfamily actually occurs in India, although *Leptaspis urceolata* has been reported, as noted below.

***Leptaspis* R. Br., Prodr. Fl. Nov. Holland. 211. 1810**

**Type.***Leptaspis banksii *R. Br.

**1. **Leptaspis urceolata** (Roxb.) R. Br., Pl. Jav. Rar. (Bennett) 23. t. 6. 1838 **

*Pharus urceolatus* Roxb., Fl. Ind. (Roxburgh) 3: 611. 1832. Type: Malaysia: A native of Pulo Pinaug

*Leptaspis manillensis* Steud., Syn. Pl. Glumac. 1: 8. 1853. Type: Thailand: Manila; *Cuming Herb. no. 1739*

*Leptaspis zeylanica* Nees, Syn. Pl. Glumac. (Steudel) 1: 8. 1853. Type: Sri Lanka.

**Distribution.** Kerala [Sri Lanka].

**Habit.** Rhizomatous perennial.

**Remarks.** Record for India fide Naithani (1990).

### Subfamily Bambusoideae Luerss., 1893

Woody or herbaceous perennials. Leaves pseudopetiolate, the culm leaves generally morphologically distinct from those on the lateral branches. Ligule membranous, with or without a fringe of hairs. Inflorescences branched or unbranched, bearing conventional spikelets or pseudospikelets. Glumes 0 or 1 to several. Lodicules 3. Stamens 3 or 6. *First seedling leaf lacking a lamina*.

Bambusoideae includes both woody and herbaceous bamboos. The lack of a lamina on the first seedling leaf may be synapomorphic (Soderstrom and Ellis 1987). In addition, the leaf anatomy is distinctive. The leaf mesophyll is marked by fusoid cells, which appear large and seemingly empty in cross section; this cell type is actually symplesiomorphic in the family (Clark et al. 1995; Kellogg 2015b; Leandro et al. 2018). Adaxial and abaxial to the fusoid cells are mesophyll cells with invaginated cell walls, known as arm cells. In the bamboos, these are asymmetric, a characteristic that is synapomorphic for the subfamily. The last several years have seen considerable progress on phylogeny and classification of bamboos (Bamboo Phylogeny Group 2012; Kelchner and Bamboo Phylogeny Group 2013), but in general work on Old World species has proceeded more slowly than that in the New World. Of the three tribes, only two occur in India.

Much of the data presented here relies on the authoritative World Checklist of Bamboos and Rattans (Vorontsova et al. 2016).

Taxonomic Editor: Lynn G. Clark, Iowa State University

### Tribe Arundinarieae Asch. & Graebn., 1902

Plants woody, with rhizomes leptomorph (running) and the plants forming more or less diffuse stands, or pachymorph (clumping), these taxa sometimes with elongated necks. Branches developing from the top to the bottom of the plant (basipetal). Foliage leaves with an outer ligule. Spikelets laterally compressed. Midrib with complex vasculature.

All members of this tribe are woody and temperate and are tetraploid (2n = 48) (Bamboo Phylogeny Group 2012; Kelchner and Bamboo Phylogeny Group 2013). Generic limits are not well defined in most cases and hybridization has been documented repeatedly (Triplett et al. 2010, 2014; Wong and Low 2011).

***Ampelocalamus* S.L. Chen, T.H. Wen & G.Y. Sheng, Acta Phytotax. Sin. 19: 332. 1981**

**Type.***Ampelocalamus actinotrichus* (Merrill & Chun) S.L. Chen, T.H. Wen & G.Y. Sheng

**2. **Ampelocalamus patellaris** (Gamble) Stapleton, Edinburgh J. Bot. 51(3): 321. 1994 **

*Dendrocalamus patellaris* Gamble, Ann. Roy. Bot. Gard. (Calcutta) 7: 86–87. t. 75. 1896. Type: Sikkim, Jungat, alt. 4000 ft.; November, 1881; *Gamble 10045* (K). LT designated by Stapleton in Edinburgh J. Bot. 51: 321. 1994.

*Patellocalamus patellaris* (Gamble) W.T. Lin, J. S. China Agric. Univ. 10(2): 46. 1989

*Sinocalamus patellaris* (Gamble) T.Q. Nguyen, Bot. Žur. (Moscow & Leningrad) 74: 1662. 1989

*Chimonobambusa jainiana* C.R. Das & D.C. Pal, J. Econ. Taxon. Bot. 4(3): 1023. 1983. Type: India: West Bengal, Kalimpong; *CNH 12178 *(CAL).

*Drepanostachyum jainianum (*C.R. Das & D.C. Pal) R.B. Majumdar, Bull. Bot. Surv. India 25(1–4): 235. 1985 (“1983”) (as “jainiana”)

*Sinarundinaria jainiana* (C.R. Das & D.C. Pal) H.B. Naithani, Indian Forester 116(12): 990. 1990

**Distribution.** Arunachal Pradesh, Assam, Jammu and Kashmir, Nagaland, Sikkim, Uttar Pradesh, Uttarakhand, West Bengal [Bhutan, China, Laos, Myanmar, Nepal].

**Habit.** Woody perennial.

***Chimonobambusa* Makino, Bot. Mag. (Tokyo) 28: 153. 1914**

**Lectotype. ***Chimonobambusa marmorea* (Mitford) Makino (*Bambusa marmorea* Mitford). LT designated by McClure in Taxon 6(7): 201–202. 1957.

**3. **Chimonobambusa armata** (Gamble) J. R. Xue & T. P. Yi, J. Bamboo Res. 2(1): 38. 1983 **

*Arundinaria armata* Gamble Ann. Roy. Bot. Gard. (Calcutta) 7: 130, pl. 119. 1896. Type: Burma: “Hills of Upper Burma. Bernardmyo”; *Mr. J.W. Oliver 1894* (K).

*Oreocalamus armatus* (Gamble) T. H. Wen, J. Bamboo Res. 5(2): 22. 1986

*Chimonocalamus armatus* (Gamble) R.B. Majumdar in S. Karthikeyan et al., Fl. Ind. ser. 4, 1 (Monocotyledon): 275. 1989

**Distribution.** India, cultivated only [China, Myanmar].

**Habit.** Woody perennial.

**4. **Chimonobambusa arunachalensis** T. P. Sharma & Borthakur, Pleione 2(1): 1. 2008 **

**Type.** India: Arunachal Pradesh, Ziro, Lower Subansiri district, 8 May 2006, *TP0140A*.

**Distribution.** Arunachal Pradesh.

**Habit.** Woody perennial.

**5. **Chimonobambusa callosa** (Munro) Nakai, J. Arnold Arbor. 6(3): 151. 1925 **

*Arundinaria callosa* Munro, Trans. Linn. Soc. London 26(1): 30. 1868. Lectotype: India: Meghalaya, Myrung; July 6, 1850; *J.D. Hooker & Thompson 1504 *(K). LT designated by C.S. Chao & Renvoize in Kew Bull. 44(2): 366. 1989.

*Chimonocalamus callosus* (Munro) J. R. Xue & T. P. Yi, Acta Bot. Yunnan. 1(2): 84. 1979

*Sinobambusa callosa *(Munro) T.H. Wen, J. Bamboo Res. 1(1): 35. 1982

*Arundinaria phar *E.G. Camus, Bambusées: 37. 1913

**Distribution.** Arunachal Pradesh, Assam, Manipur, Meghalaya, Mizoram, Nagaland [Bhutan].

**Habit.** Woody perennial.

**6. **Chimonobambusa jainii** T. P. Sharma & Borthakur, J. Econ. Taxon. Bot. 32(4): 783–785, f. 1. 2008 **

**Type. **India: Arunachal Pradesh, Ziro, Lower Subansiri district, 6 May 2006, TP0140.

**Distribution.** Arunachal Pradesh.

**Habit.** Woody perennial.

**7. **Chimonobambusa quadrangularis** (Franceschi) Makino, Bot. Mag. (Tokyo) 28: 153. 1914 **

*Bambusa quadrangularis* Franceschi, Bull. Soc. Tosc. Ortic. 5: 401. 1880 Type unknown.

*Arundinaria quadrangularis* (Franceschi) Makino, Bot. Mag. (Tokyo) 9: 71. 1895

*Phyllostachys quadrangularis* (Fraceschi) Rendle, J. Linn. Soc., Bot. 36: 443. 1904

*Tetragonocalamus quadrangularis* (Franceschi) Nakai, J. Jap. Bot. 9: 90. 1933

**Distribution.** Meghalaya, cultivated? [China, Japan].

**Habit.** Woody perennial.

***Chimonocalamus* J. R. Xue & T. P. Yi, Acta Bot. Yunnan. 1(2): 76. 1979**

**Type. ***Chimonocalamus delicatus* J. R. Xue & T. P. Yi.

**8. **Chimonocalamus griffithianus** (Munro) J. R. Xue & T. P. Yi, Acta Bot. Yunnan. 1(2): 83–84. 1979 **

*Arundinaria griffithiana* Munro, Trans. Linn. Soc. London 26(1): 20. 1888. Syntypes: India: Assam, Khasia Mountain, Mofong; *Griffith Assam Dept. 39* (K). *J.D. Hooker s.n. *(K).

*Chimonobambusa griffithiana* (Munro) Nakai, J. Arnold Arbor. 6(3): 151. 1925

*Sinarundinaria griffithiana* (Munro) C.S. Chao & Renvoize, Kew Bull. 44(2): 353. 1989

**Distribution.** Arunachal Pradesh, Assam, Manipur, Meghalaya, Mizoram, Nagaland, Sikkim, West Bengal [China, Myanmar].

**Habit.** Woody perennial.

**9. **Chimonocalamus longiusculus** Hsueh & T. P. Yi, Acta Bot. Yunnan. 1(2): 80–81, pl. 6. 1979. **

**Type.** China: Yunnan: Xichou, evergreen broad-leaved forests, 1600–1700 m, 31 May 1977; *S.W. Xian 6979*?

**Distribution.** Arunachal Pradesh.

**Habit.** Woody perennial.

**10. **Chimonocalamus lushaiensis** Ohrnb., Bamboos World Introd. 3: 14. 1996. **

*Sinarundinaria longispiculata* C.S. Chao & Renvoize, Kew Bull. 43: 411. 1988. Type: India: Assam, Sangao, Lushai Hills, 1300 m; Mar 1953; *Thakur Rup Chand 6889* (K) [Replaced synonym]

*Chimonocalamus longispiculatus* R. B. Majumdar. Fl. Ind. Enum. Monocot 276. 1989. Type: ?

*Chimonocalamus longispiculatus* (C.S. Chao & Renvoize) D.Z. Li, Acta Bot. Yunnan. 16: 41. 1994. Nom. illegit.

*Sinarundinaria arunachalensis* H.B. Naithani, Indian Forester 117(1): 78. 1991

**Distribution.** Arunachal Pradesh, Assam.

**Habit.** Woody perennial.

**11. **Chimonocalamus nagalandianus** (H.B. Naithani) L.G. Clark, comb. nov. **

urn:lsid:ipni.org:names:77212051-1

*Sinarundinaria nagalandiana* H.B. Naithani, Indian Forester 120(12): 1120. 1994. Type: India: Nagaland, Niriyo Peak, alt. 3840 ft., Wokha; November, 1986; *H.B. Naithani 1477* (DD).

**Distribution.** Nagaland.

**Habit.** Woody perennial.

**Remarks.**Seethalakshmi and Kumar (1998) indicate that this species is allied to *Chimonocalamus griffithianus* (as *Sinarundinaria griffithianus*) so a new combination in *Chimonocalamus* is here proposed. This species has the root spines characteristic of this genus, but is unknown in flower.

***Drepanostachyum* Keng f., Journal of Bamboo Research 2(1): 15. 1983**

**Type. ***Drepanostachyum falcatum* (Nees) Keng f.

**12. **Drepanostachyum falcatum** (Nees) Keng f., J. Bamboo Res. 2(1): 16. 1983 **

*Arundinaria falcata* Nees, Linnaea 9: 478. 1835

*Sinarundinaria falcata* (Nees) C.S. Chao & Renvoize, Kew Bull. 44(2): 357. 1989

*Arundinaria gracilis *(hort. ex Rivière & C. Rivière) Blanch., Rev. Hist. Nat. [Paris]. 58: 490. 1886

*Bambusa gracilis* hort. ex Rivière & C. Rivière, Bull. Soc. Natl. Acclim. France Ser. 3, 5(25): 682. 1878

**Type.** “In Nepalia legit, Royle.” Lectotype: Northwest India; *Royle s.n. *(K). LT designated by C.S. Chao & Renvoize in Kew Bull. 44(2): 358. 1989.

**Distribution.** Himachal Pradesh, Uttar Pradesh, Uttarakhand [Nepal, Pakistan].

**Habit.** Woody perennial.

**Remarks.***Arundinaria gracilis* (hort. ex Rivière & C. Rivière) Blanch. is included in Seethalakshmi and Kumar (1998) but is the subject of taxonomic confusion. The name is based on *Bambusa gracilis* hort. ex Rivière & C. Rivière, but this is a synonym of *Drepanostachyum falcatum* (Nees) Keng f., according to Vorontsova et al. (2016). As noted by taxonomic editor Clark, *Arundinaria gracilis* Lafossse is listed as a nomen nudum in TROPICOS, apparently because the protologue fails to cite a type. This entity requires further investigation.

**13. **Drepanostachyum intermedium** (Munro) Keng f., J. Bamboo Res. 2(1): 18. 1983 **

*Arundinaria intermedia* Munro, Trans. Linn. Soc. London 26(1): 28. 1868. Type: India: Sikkim, alt. 7000–8000 ft.; 1848; *J.D. Hooker s.n. *(K)

*Chimonobambusa intermedia* (Munro) Nakai, J. Arnold Arbor. 6: 151. 1925

*Sinarundinaria intermedia* (Munro) C.S. Chao & Renvoize, Kew Bull. 44(2): 357. 1989

**Distribution.** Arunachal Pradesh, Mizoram, Sikkim, West Bengal [Bhutan, Nepal].

**Habit.** Woody perennial.

**14. **Drepanostachyum khasianum** (Munro) Keng f., Journal of Bamboo Research 2(1): 18. 1983 **

*Arundinaria khasiana* Munro, Trans. Linn. Soc. London 26(1): 28. 1868. Type: India: Khasia, Shillong, 5800 ft.; *Griffith 1058* (K)

*Chimonobambusa khasiana* (Munro) Nakai, J. Arnold Arbor. 6: 151. 1925

**Distribution.** Assam, Manipur, Meghalaya, Sikkim, Uttarakhand, West Bengal [Bhutan, Nepal].

**Habit.** Woody perennial.

**15. **Drepanostachyum kurzii** (Gamble) Pandey ex D.N. Tewari, Monogr. Bamboo 90. 1993 **

*Arundinaria kurzii* Gamble, Ann. Roy. Bot. Gard. (Calcutta) 7: 25. t. 25. 1896. Type: Burma: Southern coast; 1878; *Kurz s.n.* (K).

*Sinarundinaria kurzii* (Gamble) M. Kumar, Bamboos of India, compendium 279. 1998

**Distribution.** Manipur, Meghalaya, Nagaland, West Bengal.

**Habit.** Woody perennial.

**16. **Drepanostachyum polystachyum** (Kurz ex Gamble) R.B. Majumdar in S. Karthikeyan et al., Fl. Ind. ser. 4, 1 (Monocotyledon): 277. 1989 **

*Arundinaria polystachya* Kurz ex Gamble, Ann. Roy. Bot. Gard. (Calcutta) 7: 7. t. 5. 1896. Lectotype: India: Sikkim, alt. 4000–5000 ft.; 1868; *Kurz & Anderson s.n.* (K). LT designated by C.S. Chao & Renvoize in Kew Bull 44(2): 359. 1989

*Chimonobambusa polystachya* (Kurz ex Gamble) Nakai, J. Arnold Arbor. 6: 151. 1925

*Sinarundinaria polystachya* (Kurz ex Gamble) C.S. Chao & Renvoize, Kew Bull. 44(2): 359. 1989

**Distribution.** Manipur, Meghalaya, Sikkim, Tripura, West Bengal.

**Habit.** Woody perennial.

**17. **Drepanostachyum suberectum** (Munro) R.B. Majumdar, Bull. Bot. Surv. India 25(1–4): 236. 1985 (“1983”) **

*Arundinaria suberecta* Munro, Trans. Linn. Soc. London 26(1): 32. 1868. Lectotype: India: Meghalaya, Mamlo; October 27, 1835; *Griffith 558* (K). LT designated by Stapleton, Edinburgh J. Bot. 51: 308. 1994.

*Sinarundinaria suberecta* (Munro) M. Kumar, Bamboos of India, compendium 286. 1998

**Distribution.** Arunachal Pradesh, Manipur, Meghalaya, Sikkim, West Bengal.

**Habit.** Woody perennial.

***Himalayacalamus* Keng f., J. Bamboo Res. 2(1): 23. 1983**

**Type. ***Himalayacalamus falconeri* (Hook. f. ex Munro) Keng f. (*Thamnocalamus falconeri* Hook. f. ex Munro**) **

**18. **Himalayacalamus falconeri** (Hook. f. ex Munro) Keng f., J. Bamboo Res. 2(1): 24. 1983 **

*Thamnocalamus falconeri* Hook. f. ex Munro, Trans. Linn. Soc. London 26(1): 34. 1868. Type: Nepal: Bagmati Zone, Kathmandu, Sheopore [Shivapuri], alt. 8000 ft.; 1821; Numer. List [Wallich] no. 5040 (K).

**Distribution.** Sikkim, Uttar Pradesh, West Bengal [Bhutan, China, Nepal].

**Habit.** Woody perennial.

**19. **Himalayacalamus hookerianus **(Munro) Stapleton, Bamboo Soc. Newsl. 17: 21. 1993 **

*Arundinaria hookeriana* Munro, Trans. Linn. Soc. London 26(1): 29. 1868. Lectotype: India: Sikkim [Neongong, 6800 ft.,] “Praong, 4000–6000 ft.”; December, 1848; *J.D. Hooker s.n.* (K). LT designated by Stapleton in Edinburgh J. Bot. 51: 318. 1995.

*Sinarundinaria hookeriana* (Munro) C.S. Chao & Renvoize, Kew Bull.44(2): 358. 1989

*Drepanostachyum hookerianum* (Munro) Keng f., J. Bamboo Res. 2(1): 17. 1983

*Chimonobambusa hookeriana* (Munro) Nakai, J. Arnold Arbor. 6(3): 151. 1925

**Distribution.** Arunachal Pradesh, Manipur, Meghalaya, Sikkim, West Bengal [Bhutan]. Native to Bhutan and north-eastern India, but commonly cultivated in West Bengal and also in Nepal.

**Habit.** Woody perennial.

***Kuruna* Attigala, Kathriar. & L.G. Clark, Phytotaxa 174: 199–200. 2014**

**Type. ***Kuruna debilis* (Thwaites) Attigala, Kathriar. & L.G. Clark.

**20. **Kuruna densifolia** (Munro) Attigala, Kathriar. & L.G. Clark, Phytotaxa 174: 200. 2014 **

*Arundinaria densifolia* Munro, Trans. Linn. Soc. London 26(1): 32. 1868. Lectotype: Sri Lanka; *Watson 25* (K). LT designated by Soderstrom & Ellis in Smithsonian Contr. Bot. 72: 12. 1988

*Chimonobambusa densifolia* (Munro) Nakai, J. Arnold Arbor. 6: 151. 1925

*Sinarundinaria densifolia* (Munro) C.S. Chao & Renvoize, Kew Bull. 44(2): 354. 1989

*Yushania densifolia* (Munro) R.B. Majumdar in S. Karthikeyan et al., Fl. Ind. ser. 4, 1 (Monocotyledon): 282. 1989

**Distribution.** Kerala [Sri Lanka].

**Habit.** Woody perennial.

**21. **Kuruna floribunda** (Thwaites) Attigala, Kathriar. & L.G. Clark, Phytotaxa 174: 200. 2014 **

*Arundinaria floribunda* Thwaites, Enum. Pl. Zeyl. (Thwaites) 375. 1864. Type: Sri Lanka: Maturatte District, alt. 5000 ft.; 1863; *Thwaites Ceylon Plant 2624* (PDA).

*Indocalamus floribundus* (Thwaites) Nakai, J. Arnold Arbor. 6: 148. 1925

*Sinarundinaria floribunda* (Thwaites) C.S. Chao & Renvoize, Kew Bull. 44(2): 356. 1989

*Yushania floribunda* (Thwaites) Demoly, Bambou 48: 12. 2006

**Distribution.** Kerala [Sri Lanka].

**Habit.** Woody perennial.

**22. **Kuruna walkeriana** (Munro) Attigala, Kathriar. & L.G. Clark, Phytotaxa 174: 200. 2014 **

*Arundinaria walkeriana* Munro, Trans. Linn. Soc. London 26(1): 21. 1868. Lectotype: Sri Lanka; *Mrs. Walker 96* (K). LT designated by Soderstrom & Ellis in Smithsonian Contr. Bot. 72: 27. 1988

*Indocalamus walkerianus* (Munro) Nakai, J. Arnold Arbor. 6: 148. 1925

*Sinarundinaria walkeriana* (Munro) C.S. Chao & Renvoize, Kew Bull. 44(2): 354. 1989

*Yushania walkeriana* (Munro) R.B. Majumdar in S. Karthikeyan et al., Fl. Ind. ser. 4, 1 (Monocotyledon): 283. 1989

**Distribution.** Kerala, Tamil Nadu [Sri Lanka].

**Habit.** Woody perennial.

**23. **Kuruna wightiana** (Nees) Attigala, Kathriar. & L.G. Clark, Syst. Bot. 41(1): 191, f. 13. 2016. **

*Arundinaria wightiana* Nees, Linnaea 9: 482. 1835. Type: “Peninsula Indiae orientalis; Herb Wight.” India: Nilgiris District; *Wight 1797 *(K, Isotype).

*Indocalamus wightianus* (Nees) Nakai, J. Arnold Arbor. 6: 149. 1925

*Sinarundinaria wightiana* (Nees) C.S. Chao & Renvoize, Kew Bull. 44(2): 356. 1989

*Yushania wightiana* (Nees) R.B. Majumdar in S. Karthikeyan et al., Fl. Ind. ser. 4, 1 (Monocotyledon): 283. 1989

*Indocalamus wightianus* (Nees) Nakai var. *hispida* (Steud.) Nakai, J. Arnold Arbor. 6: 149. 1925

*Arundinaria wightiana* Nees var. *hispida *(Steud.) Gamble, Ann. Roy. Bot. Gard. (Calcutta) 7: 5. 1896

*Yushania wightiana* (Nees) R.B. Majumdar var. *hispida *(Steud.) R.B. Majumdar & S. Karthikeyan in S. Karthikeyan et al., Fl. Ind. ser. 4, 1(Monocotyledon): 283. 1989

*Arundinaria hispida* Steud., Syn. Pl. Glumac. 1: 335. 1854. Type: India: “In sylvis prope Sispara Nilagiri”; *Hohenacker 1282* (K).

**Distribution.** Kerala, Tamil Nadu.

**Habit.** Woody perennial.

***Phyllostachys* Siebold & Zucc., Abh. Math.-Phys. Cl. Königl. Bayer. Akad. Wiss. 3: 745. 1843, nom. cons.**

**Type. ***Phyllostachys bambusoides* Siebold & Zucc.

**24. **Phyllostachys aurea** Carrière ex Rivière & C. Rivière, Bull. Soc. Natl. Acclim. France ser. 3, 5: 716 f. 36, 37. 1878 **

**Type.** Tunis, Cultivated in “Jardim du Hamma”; *Anonymous s.n.* (P).

**Distribution.** Uttarakhand [China]. Cultivated; native to China and Japan, widely cultivated.

**Habit.** Woody perennial.

**25. **Phyllostachys bambusoides** Siebold & Zucc., Abh. Math.-Phys. Cl. Königl. Bayer. Akad. Wiss. 3(3): 746. pl. 5. f. 3. 1843 **

**Type.** Japan; *P.F. von Siebold s.n.* (L).

**Distribution. **Assam, Himachal Pradesh [China]. Cultivated; native to China and Japan, widely cultivated.

**Habit.** Woody perennial.

**26. **Phyllostachys mannii** Gamble, Ann. Roy. Bot. Gard. (Calcutta) 7: 28. t. 28. 1896 **

*Phyllostachys assamica* Gamble ex Brandis, Indian Trees 667. 1906. Type: India: Mishmi Hills, Namdang, Lakhimpur District, Sadiya; *Griffith s.n.*

*Phyllostachys bambusoides* sensu Gamble, Fl. Brit. India (J.D. Hooker). 7(22): 386. 1896, non Siebold & Zucc., 1843

**Type.** India: Shillong, Kashia Hills and said to have come from the Naga Hills; 1889; *G. Mann s.n.*

**Distribution.** Arunachal Pradesh, Assam, Meghalaya, Nagaland [China, Myanmar]. Native, but also cultivated.

**Habit.** Woody perennial.

**27. **Phyllostachys nigra** (Lodd. ex Lindl.) Munro, Trans. Linn. Soc. London 26(1): 38, 123. 1868 **

*Bambusa nigra* Lodd. ex Lindl., Penny Cyclop. 3: 357. 1835

*Phyllostachys puberula* var. *nigra* (Lodd. ex Lindl.) Makino, Bot. Mag. (Tokyo) 14: 64. 1900

*Phyllostachys puberula* var. *nigra* (Lodd. ex Lindl.) J. Houz., Act. Congr. Int. Bot. (Brux.) 2: 223. 1912, isonym.

*Sinoarundinaria nigra* (Lodd. ex Lindl.) Ohwi ex Mayeb., Fl. Austro-Higo. 86. 1931

**Distribution.** India. Cultivated only; native to S Hunan, China.

**Habit.** Woody perennial.

**28. **Phyllostachys nigra** (Lodd. ex Lindl.) Munro var. **puberula** (Miq.) Kailash, comb. & stat. nov. **

urn:lsid:ipni.org:names:77212052-1

*Phyllostachys puberula *var. *puberula* in Makino, Bot. Mag. (Tokyo) 14: 64. 1900

*Phyllostachys henonis* Mitford, Garden (London 1871–1927) 47: 3. 1895

*Bambusa puberula* Miq. Ann. Mus. Bot. Lugduno-Batavi 2: 285. 1866. Type: Japan: Osakka; *Siebold, Buerger & Textor s.n.*

*Phyllostachys puberula* (Miq.) Munro, Gard. Chron., n. s. 6: 773, 774. 1876

*Phyllostachys nigra* (Lodd. ex Lindl.) Munro var. *henonis* (Mitford) Stapf ex Rendle, J. Linn. Soc., Bot. 36(254): 442–443. 1904

**Distribution.** India. Cultivated; native to S Hunan, China.

**Habit.** Woody perennial.

***Pleioblastus* Nakai, J. Arnold Arbor. 6(3): 145. 1925**

**Lectotype. ***Pleioblastus communis* (Makino) Nakai (*Arundinaria communis* Makino). LT designated by McClure in Taxon 6: 207. 1957.

**29. **Pleioblastus simonii** (Carrière) Nakai, J. Arnold Arbor. 6(3): 147. 1925 **

*Bambusa simonii* Carrière, Rev. Hort. [Paris]. 37: 380. 1866. Type: China?; 1862; *E. Simon s.n.* (P).

**Distribution.** Arunachal Pradesh. Possibly cultivated in India; probably native to Japan.

**Habit.** Woody perennial.

**Remarks.** Taxonomic editor Clark notes, “Although the type is supposedly from China, I know of no valid records from that country.”

***Pseudosasa* Makino ex Nakai, J. Arnold Arbor. 6(1): 150. 1925**

**Lectotype. ***Pseudosasa japonica* (Siebold & Zucc. ex Steud.) Nakai (*Arundinaria japonica* Siebold & Zucc. ex Steud.). LT designated by LT designated by McClure in Taxon 6(7): 207. 1957.

**30. **Pseudosasa japonica** (Siebold & Zucc. ex Steud.) Makino. ex Nakai, J. Arnold Arbor. 6: 150. 1925 **

*Arundinaria japonica* Siebold & Zucc. ex Steud., Syn. Pl. Glumac. 1: 334. 1854. Type: “Java” Japan: Metake; *P. F. von Siebold s.n.* (L).

**Distribution.** Meghalaya, West Bengal [China]. Cultivated only; native to Japan.

**Habit.** Woody perennial.

***Sarocalamus* Stapleton, Novon 14(3): 346. 2004**

**Type. ***Sarocalamus racemosus* (Munro) Stapleton.

**31. **Sarocalamus racemosus** (Munro) Stapleton, Novon 14(3): 347. 2004 **

*Arundinaria racemosa* Munro, Trans. Linn. Soc. London 26: 17. 1868 Lectotype: India: North East Himalaya [Sikkim & Darjeeling District], Birch Hill, alt. 6000 ft.; August, 1857; *Thomson s.n.* (K). LT selected by Gamble, 1912 (fide Stapleton et al. 2004).

*Fargesia racemosa* (Munro) T. P. Yi, J. Bamboo Res. 2: 39. 1983

*Yushania racemosa* (Munro) R. B. Majumdar in S. Karthikeyan, Fl. Ind. ser. 4, 1 (Monocotyledon): 283. 1989

**Distribution.** Arunachal Pradesh, Manipur, Nagaland, Sikkim, West Bengal [Bhutan, China, Nepal].

**Habit.** Woody perennial.

***Semiarundinaria* Makino ex Nakai, J. Arnold Arbor. 6: 150. 1925**

**Lectotype. ***Semiarundinaria fastuosa* (Mitford) Nakai (*Bambusa fastuosa* Mitford). LT designated by A. Rehder in Bibliogr. Cult. Trees Shrubs 638. 1949

**32. **Semiarundinaria densiflora** (Rendle) T.H. Wen, J. Bamboo Res. 8(1): 24. 1989 **

*Arundinaria densiflora* Rendle, J. Linn. Soc., Bot. 36: 434. 1904. Syntypes: Kiangsu: Kiangsu Hills; *Faber 19* (K). Chekiang: Hills near Huchan, Taiboo Lake; *Charles 227*(K).

*Fargesia densiflora* (Rendle) Nakai, J. Arnold Arbor. 6: 152. 1925

*Brachystachyum densiflorum* (Rendle) Keng, Sunyatsenia 4 (3–4): 153. 1940

**Distribution.** Kerala [China]. Cultivated in India.

**Habit.** Woody perennial.

***Thamnocalamus* Munro, Trans. Linn. Soc. London 26: 33, 157. 1868**

**Lectotype. ***Thamnocalamus spathiflorus* (Trin.) Munro (*Arundinaria spathiflora* Trin.). LT designated by A. Rehder in Bibliogr. Cult. Trees 639. 1949.

**33. **Thamnocalamus aristatus** (Gamble) E.G. Camus, Bambusees (Camus) 54. 1913 **

*Arundinaria aristata* Gamble, Ann. Roy. Bot. Gard. (Calcutta) 7: 18, t. 17. 1896. Lectotype: India: Sikkim, Phalut, alt. 10000 ft.; *Gammie s.n.* (K). LT designated by C.S. Chao & Renvoize in Kew Bull. 44(2): 363. 1989.

*Thamnocalamus spathiflorus* Munro, Trans. Linn. Soc. London 26(1): 34. 1868, p.p., non Trin. 1935

**Distribution.** Arunachal Pradesh, Assam, Manipur, Sikkim, West Bengal [Bhutan, Nepal].

**Habit. **Woody perennial.

**34. **Thamnocalamus occidentalis** (Stapleton) Stapleton, J. Bot. Res. Inst. Texas 1(1): 140. 2007 **

*Thamnocalamus spathiflorus* Munro ssp. *occidentalis* Stapleton, Edinburgh J. Bot. 51(2): 283. 1994. Type: India: Uttar Pradesh, Kedarkanda, 9000 ft.; June, 1893; *Gamble 24341* (K).

**Distribution.** Himachal Pradesh, Uttar Pradesh.

**Habit. **Woody perennial.

**35. **Thamnocalamus spathiflorus** (Trin.) Munro, Trans. Linn. Soc. London 26(1): 34. 1868 **

*Arundinaria spathiflora* Trin., Mém. Acad. Imp. Sci. Saint-Pétersbourg, Sér. 6, Sci. Math., Seconde Pt. Sci. Nat. 3: 617. 1835. Lectotype: Nepal: North West of Himalaya, alt. 7000 ft.; Numer. List [Wallich] no. 5041 (K). LT designated by C.S. Chao & Renvoize in Kew Bull. 44(2): 363. 1989

**Distribution.** Arunachal Pradesh, Assam, Himachal Pradesh, Uttar Pradesh, Uttarakhand [Bhutan, China, Nepal].

**Habit.** Woody perennial.

***Yushania* Keng f., Acta Phytotaxonomica Sinica 6(4): 355–356. 1957**

**Type. ***Yushania niitakaymensis* (Hayata) P. C. Keng (*Arundinaria niitakaymensis* Hayata)

**36. **Yushania anceps** (Mitf.) W.C. Lin, Bull. Taiwan Forest Res. Inst. 248: 9. 1974 **

*Arundinaria anceps* Mitf., Bamb. Gard. 181. 1896. Type: Asia.

*Sinarundinaria anceps* (Mitf.) C.S. Chao & Renvoize, Kew Bull. 44(2): 359. 1989

*Arundinaria jaunsarensis* Gamble, Ann. Roy. Bot. Gard. (Calcutta) 7: 23. t. 22. 1896. Type: India: Uttar Pradesh, Jaunsar Hills, Mundali, alt. 7500 ft.; May, 1891; *Gamble 23134* (K).

*Chimonobambusa jaunsarensis* (Gamble) K.N. Bahadur & H.B. Naithani, Indian J. Forest. 1(1): 41. 1978

*Yushania jaunsarensis* (Gamble) T.P. Yi, J. Bamboo Res. 2(1): 39. 1983

*Fargesia elegans* (Kurz) J. Campbell ssp. *jaunsarensis* (Gamble) J. Campbell, Gen. Himal. Bamb. 38. 1985

**Distribution.** Uttar Pradesh. Cultivated in Europe and the U.S.A.

**Habit.** Woody perennial.

**37. **Yushania elegans** (Kurz) R.B. Majumdar in S. Karthikeyan et al., Fl. Ind. ser. 4, 1 (Monocotyledon): 282. 1989 **

*Arundinaria elegans* Kurz, J. Asiat. Soc. Bengal, Pt. 2, Nat. Hist. 42: 249. 1873. Type: Burma: Martaban, Nattaung Hills, alt. 5000–7000 ft.; *Kurz 144* (K).

*Sinobambusa elegans* (Kurz) Nakai, J. Arnold Arbor. 6: 152. 1925

*Fargesia elegans* (Kurz) J. Campbell, Gen. Himal. Bamb. 37. 1985

*Sinarundinaria elegans* (Kurz) C.S. Chao & Renvoize, Kew Bull. 44(2): 357. 1989

**Distribution.** Arunachal Pradesh, Nagaland [Myanmar].

**Habit.** Woody perennial.

**38. **Yushania hirsuta** (Munro) R.B. Majumdar in S. Karthikeyan et al., Fl. Ind. ser. 4, 1 (Monocotyledon): 282. 1989 **

*Arundinaria hirsuta* Munro, Trans. Linn. Soc. London 26(1): 30. 1868. Lectotype: India: Khasi Hills, among rocks, 5600 ft.; November, 1835; *Griffith 6726* (K). LT designated by C.S. Chao & Renvoize in Kew Bull. 44(2): 355. 1989.

*Sinarundinaria hirsuta* (Munro) C.S. Chao & Renvoize, Kew Bull. 44(2): 355. 1989

**Distribution.** Arunachal Pradesh, Assam, Meghalaya, Nagaland, Sikkim, West Bengal [Bhutan].

**Habit.** Woody perennial.

**39. **Yushania maling **(Gamble) R.B. Majumdar in S. Karthikeyan et al., Fl. Ind. ser. 4, 1 (Monocotyledon): 283. 1989 **

*Arundinaria maling* Gamble, Bull. Misc. Inform. Kew 1912: 139. 1912. Lectotype: India: Darjeeling District, Tonglo [Tanglu], alt. 9000 ft.; May 23, 1904; *Osmaston s.n.* (K). LT designated by C.S. Chao & Renvoize in Kew Bull. 44(2): 356. 1989.

*Fargesia maling* (Gamble) J. Campbell, Gen. Himal. Bamb. 40. 1985

*Sinarundinaria maling* (Gamble) C.S. Chao & Renvoize, Kew Bull. 44(2): 356. 1988

**Distribution.** Arunachal Pradesh, Sikkim, West Bengal (all Naithani). [Bhutan, Nepal].

**Habit.** Woody perennial.

**40. **Yushania microphylla** (Munro) R.B. Majumdar in S. Karthikeyan et al., Fl. Ind. ser. 4, 1 (Monocotyledon): 283. 1989 **

*Arundinaria microphylla* Munro, Trans. Linn. Soc. London 26(1): 32. 1868. Syntypes: Bhutan: Tashigang District, Sanah, alt. 7000 ft.; *Griffiths 623 *(K). Bhutan: Sanah, alt. 6000–10000 ft.; *Griffiths s.n.* (K).

*Bambusa microphylla* Griff., J. Trav. 1: 242, 259. 1847, nom. nud.

*Sinarundinaria microphylla* (Munro) C.S. Chao & Renvoize, Kew Bull. 44(2): 354. 1989

**Distribution.** Assam, Kerala, Sikkim, Meghalaya [Bhutan, Nepal].

**Habit.** Woody perennial.

**41. **Yushania pantlingii** (Gamble) R.B. Majumdar in S. Karthikeyan et al., Fl. Ind. ser. 4, 1 (Monocotyledon): 283. 1989 **

*Arundinaria pantlingii* Gamble, Ann. Roy. Bot. Gard. (Calcutta) 7: 129 (‘Pantlingi’). t. 118. 1896. Type: India: Darjeeling District, Sikkim border “Hills of British Bhutan,” Rechi La, 11000 ft.; September, 1895; *Pantling’s collectors s.n.* (K).

*Fargesia pantlingii* (Gamble) J. Campbell, Gen. Himal. Bamb. 41. 1985

*Semiarundinaria pantlingii* (Gamble) Nakai, J. Arnold Arbor. 6: 151. 1925

*Sinarundinaria pantlingii* (Gamble) C.S. Chao & Renvoize, Kew Bull. 44(2): 359. 1989

**Distribution.** Arunachal Pradesh, Sikkim, West Bengal [Bhutan, Nepal].

**Habit.** Woody perennial.

**42. **Yushania rolloana** (Gamble) T.P. Yi, J. Bamboo Res. 2(1): 39. 1983 **

*Arundinaria rolloana* Gamble, Ann. Roy. Bot. Gard. (Calcutta) 7: 24. t. 23. 1896. Type: India: Assam, Naga Hills, Zullah Valley, alt. 5000–7000 ft.;1889; “Gopal Banerjee”; *J. Rollo s.n.* (K).

*Sinarundinaria rolloana* (Gamble) C.S. Chao & Renvoize, Kew Bull. 44(2): 355. 1989

**Distribution.** Assam, Nagaland.

**Habit. **Woody perennial.

Taxonomic editor Clark suggests that it is doubtful that the following species of Arundinarieae occur in India:

*Himalayacalamus gyirongensis* (T.P. Yi) Stapleton, J. Bot. Res. Inst. Texas 1(1): 141. 2007 [doubtful species; also, this is considered a synonym of *Himalayacalamus falconeri* in the Flora of China.]

*Himalayacalamus planatus *Stapleton, J. Bot. Res. Inst. Texas 1(1): 137. 2007 [known from Nepal]

*Himalayacalamus porcatus *Stapleton, J. Bot. Res. Inst. Texas 1(1): 141. 2007 [doubtful species]

*Thamnocalamus chigar* (Stapleton) Stapleton, J. Bot. Res. Inst. Texas 1(1): 140. 2007 [doubtful species]

*Thamnocalamus nepalensis* (Stapleton) Stapleton, J. Bot. Res. Inst. Texas 1(1): 140. 2007 [doubtful species]

### Tribe Bambuseae Kunth ex Dumort., 1829

Woody bamboos, often tree-like. Rhizomes pachymorph and the plants clumping, but necks sometimes elongated; one New World genus sometimes with amphimorph rhizomes.Branches developing either from the bottom to the top of the plant (acropetal) or bidirectional. Outer ligule generally present. Spikelets laterally compressed, disarticulating above the glumes. Midrib mostly with complex vasculature.

Members of this tribe are tropical and either tetraploid or hexaploid, mostly with 2n = 48 or 72, although some aneuploid numbers have been reported (Bamboo Phylogeny Group 2012). Pseudospikelets, in which the spikelet themselves form branched structures, are common but not universal in this group (Bamboo Phylogeny Group 2012). The Old World species of Bambuseae form a clade separate from the New World species (Kelchner and Bamboo Phylogeny Group 2013; Triplett et al. 2014).

***Bambusa* Schreb., Gen. Pl., ed. 8[a] 236. 1789, nom. cons.**

**Type.***Bambusa arundinacea* (Retz.) Willd. (*Bambos arundinacea* Retz.).

**43. **Bambusa affinis** Munro, Trans. Linn. Soc. London 26(1): 93. 1868 **

**Type.** “Hab. in Ind. or. Burmah, Yoongalum (Teesheincolis); *Brandis 18*.”

**Distribution. **Tripura [Myanmar].

**Habit.** Woody perennial.

**Remarks.** Record for India fide Naithani (1990).

**44. **Bambusa alamii** Stapleton, J. Edinb. J. Bot. 51(1): 10, f. 3. 1994. **

**Type.** Nepal; 9 Jan 1991; *Stapleton 907* (E).

**Distribution.** Assam [Bangladesh, Bhutan, Nepal].

**Habit.** Woody perennial.

**45. **Bambusa alemtemshii** H.B. Naithani, Indian Forester 133(11): 1575. 2007 **

**Type.** India: Nagaland, Longsa village, Mokokchung District; December 5, 2004; *H.B. Naithani 4338* (DD).

**Distribution.** Assam, Nagaland.

**Habit.** Woody perennial.

**46. **Bambusa balcooa** Roxb., Fl. Ind. (Roxburgh) 2: 196. 1832 **

*Dendrocalamus balcooa* (Roxb.) Voigt, Hort. SubLurb. Calcutt. 718. 1845

**Type.** “A native of India: Bengal.” Lectotype: *Roxb. Icones 1402* (K). LT designated by Stapleton in Edinburgh J. Bot. 51(1): 12. 1994.

**Distribution.** Arunachal Pradesh, Assam, Bihar, Kerala, Manipur, Meghalaya, Mizoram, Nagaland, Odisha, Tripura, Uttarakhand, Uttar Pradesh, West Bengal [Bangladesh, China]. Widely cultivated in northern India and Bangladesh, thought to originate from this region but known only in cultivation.

**Habit.** Woody perennial.

**47. **Bambusa bambos** (L.) Voss, Vilm. Blumengärtn., ed. 3. 1: 1189. 1895 var. **bambos

*Arundo bambos* L., Sp. Pl. 1: 81. 1753. Type: “Habitat in India utraque.” Lectotype = “Ily” in Rheede, Hort. Malab. 1: 25. t. 16. 1678. LT designated by Judziewicz in Görts-van Rijn (ed.), Fl. Guianas, ser. A, 8: 50. 1990.

*Bambusa arundinacea* (Retz.) Willd., Sp. Pl., ed. 4 [Willdenow] 2: 245. 1799

*Bambos arundinacea* Retz., Observ. Bot. (Retzius) 5: 24. 1788 (“1789”). Lectotype: India orientalis: *Herbarium Retzii s.n. right-hand side only* (No. 2), H554/90 (LD). LT designated by Xia & Stapleton in Kew Bull. 52(3): 697. 1997.

**Distribution.** Andhra Pradesh, Assam, Chhattisgarh, Goa, Gujarat, Himachal Pradesh, Karnataka, Kerala, Madhya Pradesh, Maharashtra, Manipur, Rajasthan, Tamil Nadu, West Bengal [Bangladesh, China, Myanmar, Pakistan, Sri Lanka]. Cultivated and naturalized in India.

**Habit.** Woody perennial.

**Remarks. **Taxonomic editor Clark notes that there are more synonyms in the literature; see Ohrnberger (1999) and Vorontsova et al. (2016).

**48. **Bambusa burmanica** Gamble, Ann. Roy. Bot. Gard. (Calcutta) 7: 35–36. t. 33. 1896 **

**Type.** Burma: Katha District, dry slopes; *J.W. Oliver s.n.*

**Distribution.** Arunachal Pradesh, Assam, Manipur, Uttar Pradesh, West Bengal [China, Myanmar].

**Habit.** Woody perennial.

**49. **Bambusa cacharensis** R.B. Majumdar, Bull. Bot. Surv. India 25(1–4): 237. f. 1. 1985 (“1983”) **

**Type.** India: Cachar, Lakhimpur, Mar Bastee; *Majumdar 74265A* (CAL).

**Distribution.** Assam, Manipur, Meghalaya, Tripura [Bangladesh].

**Habit.** Woody perennial.

**Remarks.** Taxonomic editor Clark notes that Devi and Bhattacharyya (2013) reported the range listed here. Record for Assam fide Naithani (1990).

**50. **Bambusa copelandii** Gamble ex Brandis, Indian Trees 671. 1906 (as “copelandi”) **

*Sinocalamus copelandii* (Gamble ex Brandis) Raizada, Indian Forester 74(1): 10. 1948 (as “copelandi”)

*Dendrocalamus copelandii* (Gamble ex Brandis) N.H.Xia & Stapleton, Kew Bull. 52: 484. 1997

**Lectotype.** India: Dehra Dun, cultivated, alt. 2200 ft.; September, 1898; *Gamble 27166* (K). LT designated by Xia & Stapleton in Kew Bull. 52: 484. 1997.

**Distribution.** Uttar Pradesh, West Bengal [China].

**Habit.** Woody perennial.

**Remarks.** Taxonomic editor Clark wrote, “This species is indicated as occurring in these two states in Seethalakshmi & Kumar (1998).” She also notes that it is cultivated in the Botanical Garden at the Calcutta & Forest Research Institute, Dehra Dun, as well as in the Tropical Botanical Garden and Research Institute, Kerala.

**51. **Bambusa griffithiana** Munro, Trans. Linn. Soc. London 26(1): 99. 1868 **

**Type.** “Hab. in Ind. or. Burmah, Mogoung; *Griffith s.n.*”

**Distribution.** Manipur [Myanmar].

**Habit.** Woody perennial.

**Remarks.** Record for Manipur fide Naithani (1990).

**52. **Bambusa jaintiana** R.B. Majumdar in S. Karthikeyan et al., Fl. Ind. ser. 4, 1 (Monocotyledon): 274. 1989 **

**Type.** India: Shillong; *G.K. Deka 31765* (CAL).

**Distribution.** Assam, Meghalaya [Bangladesh, Bhutan, Myanmar].

**Habit.** Woody perennial.

**53. **Bambusa khasiana** Munro, Trans. Linn. Soc. London 26(1): 97. 1868 **

**Syntypes.** India: Khasia, alt. 2000–4000 ft., Churra; *Hooker no. 1097*. Jasper Hill, Mahadeb; *Hooker 496*

**Distribution.** Assam, Manipur, Meghalaya [Myanmar].

**Habit.** Woody perennial.

**54. **Bambusa kingiana** Gamble, Ann. Roy. Bot. Gard. (Calcutta) 7: 46. t. 42 1896 **

**Type.** Burma: Katha District, Petsut; *J.W. Oliver s.n.*

**Distribution.** Manipur [Myanmar].

**Habit.** Woody perennial.

**Remarks.** Record for Manipur fide Naithani (1990).

**55. **Bambusa longispiculata** Gamble ex Brandis, Indian Trees 668. 1906 **

**Type.** Bangladesh: Chittagong Hill tracts, Ruby mine District, alt. 3500 ft.;* Griffith s.n.*

**Distribution.** Meghalaya, Mizoram, Tamil Nadu, Uttar Pradesh, West Bengal [Bangladesh, China, Myanmar].

**Habit.** Woody perennial.

**Remarks.** Records for Meghalaya and Mizoram fide Naithani (1990).

**56. **Bambusa majumdari** P. Kumari & P. Singh, Kew Bull. 64: 565. 2009 **

**Type.** India: Meghalaya: Garo Hills, near Tura, 14 Sept. 2005, *Kumari & Singh 34696* (CAL).

**Distribution.** Meghalaya.

**Habit.** Woody perennial.

**57. **Bambusa mohanramii** P. Kumari & P. Singh, Kew Bull. 64: 567. 2009 **

**Type.** India: Meghalaya: Jaintia hills, Khleiriaht, 9 April 2004, *Kumari 34608* (CAL).

**Distribution.** Meghalaya.

**Habit.** Woody perennial.

**58. **Bambusa multiplex** (Lour.) Raeusch. ex Schult. & Schult.f., Syst. Veg., ed. 15 bis [Roemer & Schultes] 7(2): 1350. 1830 **

*Arundo multiplex* Lour., Fl. Cochinch. 1: 58. 1790. Type: “Habitat loca culta in provinciis Borialibus Cochinchinae, ex eaque Plantantur fepes ad divifionem hortorum.”

*Bambusa nana* Roxb., Fl. Ind. (Roxburgh) 2: 199. 1832. Type: “*Keu-fa*, of the Chinese; a native of their country, and now plentiful in the Botanic Garden at Calcutta…”

*Ludolfia glaucescens* Willd., Ges. Naturf. Freunde Berlin Mag. Neuesten Entdeck. Gesammten Naturk. 2: 320. 1808. Type: “Die von Michaux beschriebene Art soll ein hoher Baum werden und in einer Rispe bluhen. Ich nenne sie.”

*Bambusa glaucescens* Siebold ex Munro, Trans. Linn. Soc. London 26(1): 89. 1868, nom. invalid, pro. syn.

**Distribution.** Assam, Bihar, Meghalaya [Bangladesh, China, Myanmar, Sri Lanka]. Probably naturalized in addition to being cultivated; probably native to Indo-China and southern China, but now known only in cultivation

**Habit.** Woody perennial.

**Remarks.** Taxonomic editor Clark wrote, “Introduced into the Indian Botanical Garden in 1794.”

**59. **Bambusa nagalandiana** H.B. Naithani, Indian Forester 133(9): 1267–1269. 2007 (as “nagalandeana”) **

**Type.** India: Nagaland, Mon District, Wanching village; December 9, 2004; *H.R. Naithani 4344* (DD).

**Distribution.** Nagaland.

**Habit.** Woody perennial.

**60. **Bambusa nairiana** P. Kumari & P. Singh, Bull. Surv. India 50: 13. 2009 (“2008”) **

**Type.** India: Meghalaya: Garo hills, near Nokrek, 14 Sept. 2005, *Kumari & Singh 34698* (CAL).

**Distribution.** Meghalaya.

**Habit.** Woody perennial.

**61. **Bambusa nutans** Wall ex Munro, Trans. Linn. Soc. London 26(1): 92. 1868 **

*Bambusa falconeri* Munro, Trans. Linn. Soc. London 26: 95. 1868

**Lectotype.** Nepal: Kathmandu, Naga-Arjun [Nagarjun, 27°44'N, 85°71'E]; 1821; Numer. List [Wallich] no. 5031(K-W). LT designated by Stapleton in Edinburgh J. Bot. 51: 17 1994.

**Distribution.** Arunachal Pradesh, Assam, Bihar, Himachal Pradesh, Jammu and Kashmir, Madhya Pradesh, Odisha, Sikkim, Tripura, Uttar Pradesh, Uttarakhand, West Bengal [Bangladesh].

**Habit.** Woody perennial.

**62. **Bambusa oliveriana** Gamble, Ann. Roy. Bot. Gard. (Calcutta) 7: 130. t. 116. 1896 **

**Syntypes.** Burma: 30 miles north from Mandalay, alt. 1000–2000 ft.; 1893–94; *J.W. Oliver s.n.* Ruby Mines Hills; *C.S. Rogers s.n.*

**Distribution.** Mizoram, Uttarakhand, West Bengal [Myanmar]. Cultivated; native to Myanmar.

**Habit. **Woody perennial.

**Remarks.** Record for Mizoram fide Naithani (1990).

**63. **Bambusa pallida** Munro, Trans. Linn. Soc. London 26(1): 97. 1868 **

*Bambusa critica* Kurz, J. Asiat. Soc. Bengal, Pt. 2, Nat. Hist. 42: 255. 1873. Type: Burma.

*Dendrocalamus criticus* (Kurz) Kurz, Prelim. Rep. Forest Pegu, App. B: 94. 1875

*Bambusa pseudopallida* R.B. Majumdar in S. Karthikeyan et al., Fl. Ind. ser. 4, 1 (Monocotyledon): 275. 1989. Type: India: Umtaph on Dawki-Jarain Road; *N.P. Balakrishnan 42700* (CAL).

**Syntypes.** India: Bengalia, Pundua & Silhet (cult.), Cachar, Khasia, Joowye, alt. 3500 ft. *Hooker & Thomson 602, 607* (K).

**Distribution.** Arunachal Pradesh, Assam, Karnataka, Kerala, Meghalaya, Mizoram, Nagaland, North Bengal, Sikkim, Tamil Nadu, Tripura, Uttaranchal, Uttar Pradesh, West Bengal [Bangladesh, Bhutan, China, Myanmar].

**Habit.** Woody perennial.

**64. **Bambusa polymorpha** Munro, Trans. Linn. Soc. London 26(1): 98. 1868 **

**Syntypes.** Burma: Martaban, Pegu, (“Kijathounwa”); *Brandis no. 1*. Rangoon; *McClelland s.n.*

**Distribution.** Arunachal Pradesh, Assam, Karnataka, Kerala, Madhya Pradesh, Manipur, Meghalaya, Tamil Nadu, Tripura, West Bengal [Bangladesh, China, Myanmar]. Cultivated in northwestern parts of India, introduced in Karnataka, Kerala and Tamil Nadu; probably native to northeastern India, Bangladesh and Myanmar.

**Habit. **Woody perennial.

**Remarks.** Records for Assam, Madhya Pradesh, and Tripura fide Naithani (1990).

**65. **Bambusa teres** Buch.-Ham. ex Munro, Trans. Linn. Soc. London 26(1): 95. 1868 **

**Syntypes.** India: Bengal, Gongachora; *Buchanan-Hamilton 881, Griffith s.n.* Assam; *Jenkins s.n.*

**Distribution.** Arunachal Pradesh, Assam, Chhattisgarh, Madhya Pradesh, Manipur, Meghalaya, Nagaland, Tripura, West Bengal [Bangladesh, Bhutan, China, Myanmar, Nepal].

**Habit. **Woody perennial.

**66. **Bambusa tulda** Roxb., Fl. Ind. (Roxburgh) 2: 193. 1832 var. **tulda

**Type.** “The common bamboo of India: Bengal, where it grows in the greatest abundance every where.” Lectotype: *Roxb. Icones 1403 *(K). LT designated by Stapleton in Edinburg J. Bot. 51(1): 19. 1994.

**Distribution.** Andhra Pradesh, Arunachal Pradesh, Assam, Bihar, Jharkhand, Karnataka, Manipur, Meghalaya, Mizoram, Nagaland, North Bengal, Odisha, Sikkim, Tripura, Uttar Pradesh, Uttarakhand, West Bengal [Bangladesh, Bhutan, China, Myanmar, Nepal].

**Habit.** Woody perennial.

**Remarks.** Taxonomic editor Clark notes “Kumari & Singh (2014) indicate that this variety is cultivated in Arunachal Pradesh, Karnataka, North Bengal and Uttar Pradesh.”

**67. **Bambusa tulda** Roxb. var. **gamblei** P. Kumari & P. Singh, Bamboo of Meghalaya 58. 2014 **

**Type.** India: Meghalaya: Garo hills, near Nokrek, 14 Sept. 2005, *Kumari & Singh 346700* (CAL).

**Distribution.** Meghalaya.

**Habit.** Woody perennial.

**68. **Bambusa vulgaris** J.C. Wendl., Coll. Pl. 2: 26, t. 47. 1810, nom. cons. **

*Bambusa surinamensis* Rupr., Bambuseae 49, t. 11. f. 49. 1839. Type: Surinam; 1827–1828; *Weigelt s.n.*

*Bambusa thouarsii* Kunth, Révis. Gramin. 1: 323, t. 73, 74. 1830. Type: “Crescit in insulis Madagascariae et Borboniae.”

**Type.** “*Bambusa vulgaris* Wend.”, India; ex herb Wendland (GOET nos. 13986–13919) (typ. cons.).

**Distribution.** Assam, Bihar, Chhattisgarh, Goa, Kerala, Madhya Pradesh, Maharashtra, Manipur, Meghalaya, Odisha, Rajasthan, West Bengal [Bangladesh, China, Sri Lanka].

**Habit.** Woody perennial.

**Remarks.** Taxonomic editor Clark notes that there are many other synonyms in the literature; see Ohrnberger (1999) and Vorontsova et al. (2016).

***Cephalostachyum* Munro, Trans. Linn. Soc. London 26: 138. 1868.**

**Type. **LT.: *C. capitatum* Munro (designated by McClure, Taxon 6: 201. 1957).

**69. **Cephalostachyum capitatum** Munro, Trans. Linn. Soc. London 26(1): 139. 1868 var. **capitatum

*Schizostachyum capitatum* (Munro) R.B. Majumdar in S. Karthikeyan et al., Fl. Ind. ser. 4, 1 (Monocotyledon): 281. 1989, non (Trin.) Rupr., 1840

*Schizostachyum munroi* S.Kumar & Par. Singh, J. Indian Bot. Soc. 70(1–4): 423. 1991

**Syntypes.** “Hab. in India or montibus, Khasia, Churra, 4000 ped. s.m.; *Wallich et Griffith 1078*, 1392 (in Herb. Griff.) Nunklow (“Sillee et Sullea”), no. 1813. Sikkim, 4000–6000 ft., Myong Valle (“Pyong”), Hooker & Thomson.”

**Distribution.** Arunachal Pradesh, Assam, Meghalaya, Mizoram, Nagaland, Sikkim [Bhutan].

**Habit.** Woody perennial.

**Remarks.** Taxonomic editor Clark notes that, “According to Kumari & Singh (2014), this was lectotypified by Stapleton in 1994. This needs to be verified, as they cite *Griffith hb. No. 2682* as the LT.”

**70. **Cephalostachyum capitatum** Munro var. **decompositum** Gamble, Ann. Roy. Bot. Gard. (Calcutta) 7: 105. 1896 (as “decomposita”) **

*Schizostachyum capitatum* (Trin.) Rupr. var. *decompositum* (Gamble) R.B. Majumdar in S. Karthikeyan et al., Fl. Ind. ser. 4, 1 (Monocotyledon): 281. 1989

**Type.** India: Sikkim; *T. Anderson & Kurz s.n.*

**Distribution.** Meghalaya, Sikkim.

**Habit. **Woody perennial.

**Remarks.** Taxonomic editor Clark notes that this variety was “not recognized in Kumari & Singh (2014), but also not formally synonymized.”

**71. **Cephalostachyum flavescens** Kurz, J. Asiat. Soc. Bengal, Pt. 2, Nat. Hist. 42: 252. 1873 **

*Schizostachyum flavescens* (Kurz) R.B. Majumdar in S. Karthikeyan et al., Fl. Ind. ser. 4, 1 (Monocotyledon): 281. 1989

**Type.** Pegu: H.B.C. culta.

**Distribution.** Andaman and Nicobar Islands, Tamil Nadu [Myanmar].

**Habit.** Woody perennial.

**Remarks.** Taxonomic editor Clark notes that this is also cultivated in West Bengal (Indian Botanic Gardens, Kolkata). Record for Andaman & Nicobar Islands fide Naithani (1990).

**72. **Cephalostachyum latifolium** Munro, Trans. Linn. Soc. London 26(1): 140. 1868 **

*Schizostachyum latifolium* (Munro) R.B. Majumdar in S. Karthikeyan, Fl. Ind. ser. 4, 1 (Monocotyledon): 281. 1989, non Gamble, 1896

*Cephalostachyum fuchsianum* Gamble, Ann. Roy. Bot. Gard. (Calcutta) 7: 107, t. 94. 1896. Type: India: Sikkim, Songchonglu, alt. 6000 ft.; 1889–1892; *Gammie s.n.* (K).

*Schizostachyum fuchsianum* (Gamble) R.B. Majumdar in S. Karthikeyan, Fl. Ind. ser. 4, 1 (Monocotyledon): 281. 1989

*Cephalostachyum longwanum* H.B. Naithani, Indian Forester 136: 406. 2010

**Lectotype.** Bhutan; *W. Griffith 2682* (K). LT designated by Stapleton in Edinburgh J. Bot. 51: 29. 1994.

**Distribution.** Arunachal Pradesh, Manipur, Nagaland, Sikkim, West Bengal [Bhutan, China, Myanmar, Nepal].

**Habit.** Woody perennial.

**73. **Cephalostachyum mannii** (Gamble) Stapleton & D. Z. Li, Kew Bull. 52: 700. 1997 **

*Arundinaria mannii* Gamble, Ann. Roy. Bot. Gard. (Calcutta) 7: 26. 1896. Type: India: Meghalaya, Amkasur, Jainita Hills, 3000 ft.; April 24, 1889; *G. Mann 21845* (K).

*Neomicrocalamus mannii* (Gamble) R.B. Majumdar in S. Karthikeyan et al., Fl. Ind. ser. 4, 1 (Monocotyledon): 279. 1989

*Racemobambos mannii* (Gamble) M. Kumar, Bamboos of India, Compendium 230. 1998

**Distribution.** Meghalaya [China].

**Habit.** Woody perennial.

**74. **Cephalostachyum mishimianum** H. B. Naithani, Indian Forester 140(7): 733. 2014 (as “mishmieanum”) **

**Type.** Arunchal Pradesh: 5 km after Udayk Pass, way to Hayuling 1200 m, Lohit District, *H.B. Naithani 2141* (holotype: DD).

**Distribution.** Arunachal Pradesh.

**Habit.** Woody perennial.

**75. **Cephalostachyum pallidum** Munro, Trans. Linn. Soc. London 26(1): 139. 1868 **

*Schizostachyum pallidum* (Munro) R.B. Majumdar in S. Karthikeyan et al., Fl. Ind. ser. 4, 1 (Monocotyledon): 282. 1989

**Type.** India: Mishmee, Birmah, Patkaye, 5000 ft.; *Griffith s.n.*

**Distribution.** Arunachal Pradesh, Manipur, Meghalaya [Myanmar].

**Habit.** Woody perennial.

**Remarks.** Taxonomic editor Clark notes that “In their discussion on p. 63, Kumari and Singh (2014) evidently consider this species as a synonym of *C. capitatum*, but do not formally synonymize it.”

**76. **Cephalostachyum pergracile** Munro, Trans. Linn. Soc. London 26(1): 141. 1868 **

*Schizostachyum pergracile* (Munro) R.B. Majumdar in S. Karthikeyan et al., Fl. Ind. ser. 4, 1 (Monocotyledon): 282. 1989

**Syntypes.** Burma: Rangoon; *J.E. McClelland s.n.* Pegu; *Brandis s.n.*

**Distribution.** Andhra Pradesh, Arunachal Pradesh, Assam, Bihar, Madhya Pradesh, Manipur, Nagaland, Odisha [China, Myanmar].

**Habit.** Woody perennial.

***Dendrocalamus* Nees, Linnaea 9: 476. 1835**

**Type.***Dendrocalamus strictus* (Roxb.) Nees (*Bambusa stricta* Roxb.).

**77. **Dendrocalamus asper** (Schult. & Schult. f.) Backer ex K. Heyne, Nutt. Pl. Ned.-Ind., ed. 2, 1: 301. 1927 **

*Bambusa aspera* Schult. & Schult. f., Syst. Veg., ed. 15 bis [Roemer & Schultes] 7(2): 1352–1353. 1830. Type: “Arundarbor aspera Rumphius, Herb. Amb. 4: 11. In Amboina et Huamchela ad montium pedes.”

**Distribution.** Andaman and Nicobar Islands [China, Myanmar].

**Habit.** Woody perennial.

**Remarks.** Taxonomic editor Clark notes that this name has other synonyms in the literature; see Ohrnberger (1999) and Vorontsova et al. (2016).

**78. **Dendrocalamus brandisii** (Munro) Kurz, Prelim. Rep. Forest Pegu, App. B: 94. 1875  **

*Bambusa brandisii* Munro, Trans. Linn. Soc. London 26(1): 109–110. 1868. Type: “Hab. in Ind. or. Tenasserim, Martaban, Pegu, usque ad 4000 ped. S.M., praecipue in saxis calcareis, Brandis no. 2

**Distribution.** Andaman and Nicobar Islands, Karnataka, Kerala, Manipur, West Bengal [Myanmar, China, Myanmar]. Naturalized and cultivated; native to Laos, Myanmar, Thailand and Vietnam but its natural distribution may include Manipur and the Andaman Islands.

**Habit.** Woody perennial.

**Remarks.** Record for Karnataka fide Naithani (1990).

**79. **Dendrocalamus calostachyus** (Kurz) Kurz, Prelim. Rep. Forest Pegu, App. B: 94. 1875 **

*Bambusa calostachya* Kurz, J. Asiat. Soc. Bengal, Pt. 2, Nat. Hist 42: 250. 1873. Type: Burma: Ava, Bhamo, Kakhyen Hills, alt. 3500 ft.; *Kurz s.n.*

**Distribution.** Meghalaya, Nagaland [China, Myanmar]. Native to northeastern India, China and Myanmar and also cultivated more widely in India.

**Habit.** Woody perennial.

**80. **Dendrocalamus collettianus** Gamble, Ann. Roy. Bot. Gard. (Calcutta) 7: 93. t. 83. 1896 **

**Type.** Burma: Fort Stedman; 1892; *Abdul Huk s.n.*

**Distribution.** West Bengal [China, Myanmar]. Only cultivated in India.

**Habit.** Woody perennial.

**81. **Dendrocalamus giganteus** Munro, Trans. Linn. Soc. London 26(1): 150. 1868 **

**Type.** “Cult. Calcatta” [Botanical Garden, Calcutta], Lectotype: *Hb. Munro* (K). LT designated by Stapleton in Edinburg J. Bot. 51(1): 23. 1994.

**Distribution.** Arunachal Pradesh, Assam, Maharashtra, Manipur, Meghalaya, Nagaland, Sikkim, Uttar Pradesh, Uttarakhand, West Bengal [China, Myanmar, Nepal, Sri Lanka]. Probably only cultivated in India; probably native to Malaysia, Myanmar and Thailand.

**Habit.** Woody perennial.

**82. **Dendrocalamus hamiltonii** Nees & Arn. ex Munro, Trans. Linn. Soc. London 26(1): 151. 1868 **

*Bambusa monogynia* Griff., Not. Pl. Asiat. 3: 63. 1851 (non *B. monogyna* Blanco, 1837)

**Lectotype.** India: Assam, Goalpara; July 17, 1808; *Buchanan-Hamilton 882* (E). LT designated by Stapleton in Edinburg J. Bot. 51(1): 23. 1994

**Distribution.** Arunachal Pradesh, Assam, Bihar, Himachal Pradesh, Manipur, Meghalaya, Mizoram, Nagaland, Odisha, Sikkim, Tripura, Uttar Pradesh, Uttarakhand [Bangladesh, Bhutan, China, Myanmar, Nepal, Pakistan].

**Habit. **Woody perennial.

**83. **Dendrocalamus hookeri** Munro, Trans. Linn. Soc. London 26(1): 151. 1868 var. **hookeri

**Lectotype.** India: Bengal orient, Pundua; June 11, 1850; *J.D. Hooker & Thomson 411* (K). LT designated by Stapleton in Edinburg J. Bot. 51(1): 26. 1994

**Distribution.** Arunachal Pradesh, Assam, Manipur, Meghalaya, Mizoram, Nagaland, North Bengal, Sikkim, West Bengal [Bhutan, Myanmar, Nepal].

**Habit.** Woody perennial.

**84. **Dendrocalamus longispathus** Kurz, Prelim. Rep. Forest Pegu, App. B: 94. 1875 **

**Type.** Burma: mixed and tropical forests of Arracan, Pegu Yomah and Martaban; *Metam s.n.*

**Distribution.** Assam, Bihar, Meghalaya, Mizoram, Odisha, Tripura, West Bengal [Bangladesh, China, Myanmar, Thailand].

**Habit.** Woody perennial.

**85. **Dendrocalamus membranaceus** Munro, Trans. Linn. Soc. London 26(1): 149. 1868 **

*Bambusa membranacea* (Munro) Stapleton & N.H. Xia, Kew Bull. 52(1): 238. 1997

*Dendrocalamus longifimbriatus* Gamble, Ann. Roy. Bot. Gard. (Calcutta) 7: 92. 1896

**Type.** Burma: Martaban, Trogla; March 12, 1827; Numer. List [Wallich] no 5029 (K). LT designated by Stapleton & N.H. Xia in Kew Bull. 52: 238. 1997.

**Distribution.** Kerala, West Bengal [China, Myanmar]. Apparently only cultivated in India; native to Laos, Myanmar and Thailand.

**Habit.** Woody perennial.

**86. **Dendrocalamus parishii** Munro, Trans. Linn. Soc. London 26(1): 149. 1868 **

*Dendrocalamus hookeri* Munro var. *parishii* (Munro) Blatt., Indian Forester 55(11): 594. 1929

**Type.** India: Punjab Himalaya; *Parish s.n.*

**Distribution.** Arunachal Pradesh, Assam, Himachal Pradesh, Manipur, Meghalaya Mizoram, Nagaland, Punjab, Sikkim, West Bengal [China, Myanmar, Nepal, Pakistan].

**Habit.** Woody perennial.

**Remarks.** Taxonomic editor Clark notes that “This species is not treated in Kumari and Singh (2014) and is not even included in their index. Perhaps does not occur in Meghalaya? Or more likely, given the distribution, is considered synonymous with *D. hookeri*. Needs further study.”

**87. **Dendrocalamus sahnii** H.B. Naithani & Bahadur, Indian Forester 108(3): 212. f. 1. 1982 **

**Type.** India: Arunachal Pradesh, Subansiri District, Zoram, alt. 6000 ft.; April 28, 1977; *H.B. Naithani ser II no. 902* (DD).

**Distribution.** Arunachal Pradesh.

**Habit.** Woody perennial.

**88. **Dendrocalamus sericeus** Munro, Trans. Linn. Soc. London 26(1): 148. 1868 **

*Dendrocalamus strictus* (Roxb.) Nees var. *sericeus *(Munro) Gamble, Man. Ind. Timb., ed. 2, 751. 1902

**Syntypes.** India: Behar [Bihar], Parasnath, alt. 4000 ft.; September, 1858; *Thomson s.n., *(K). India: Behar [Bihar], Parasnath; February, 1848; *Hooker s.n.* (K).

**Distribution.** Bihar.

**Habit.** Woody perennial.

**Remarks.** Taxonomic editor Clark notes that this was treated in Ohrnberger (1999) as a variety of *D. strictus*. Further study is required.

**89. **Dendrocalamus sikkimensis** Gamble ex Oliver, Hooker’s Icon. Pl. 18: t. 1770. 1888 **

**Lectotype.** India: Sikkim; July 20, 1885; *Pantling s.n.* (K). LT designated by Stapleton in Edinburg J. Bot. 51(1): 26. 1994

**Distribution.** Arunachal Pradesh, Assam, Meghalaya, Mizoram, Nagaland, North Bengal, Sikkim, West Bengal [Bhutan, China, Nepal].

**Habit.** Woody perennial.

**90. **Dendrocalamus somdevae** H.B. Naithani, Indian Forester 119(6): 504. 1993 (as “somdevai”) **

**Type.** Uttarakhand: Dehra Dun, Hardwar road between Jogiwala and Majri (Mokhampur), 11.3.1991, Som Deva 10985 (Holotype DD).

**Distribution.** Uttar Pradesh.

**Habit. **Woody perennial.

**91. **Dendrocalamus strictus** (Roxb.) Nees, Linnaea 9: 476–477. 1835 **

*Bambos stricta* Roxb., Pl. Coromandel 1: 58. t. 80. 1798. Type: India: undershrub in deciduous forests; *Roxburgh s.n.* (K).

**Distribution.** Andhra Pradesh, Arunachal Pradesh, Assam, Bihar, Chhattisgarh, Dadra and Nagar Haveli, Gujarat, Himachal Pradesh, Jharkhand, Karnataka, Kerala, Madhya Pradesh, Maharashtra, Meghalaya, Odisha, Rajasthan, Tamil Nadu, Uttar Pradesh [Bangladesh, China, Nepal, Pakistan].

**Habit.** Woody perennial.

**Remarks.** Author K. Gandhi observes, “Since Roxburgh’s collections from the Coromandel Coast were destroyed in a flood, a good comprehensive collection with the same local name from the same area should be made and designated as a neotype.” See also Stapleton (1994).

***Dinochloa* Buse, Gram. (Buse) 47. 1854**

**Type.***Dinochloa tjankorreh* Buse, *nom. illeg.* (*Bambusa scandens* Blume, *Dinochloa scandens* (Blume) Kuntze).

**92. **Dinochloa andamanica** Kurz, J. Asiat. Soc. Bengal, Pt. 2, Nat. Hist. 42: 253. 1873 **

**Type.** India: “Andamans (etiam in insula Nicobaricis)”.

**Distribution.** Andaman and Nicobar Islands.

**Habit.** Woody perennial.

**93. **Dinochloa mcclellandii** (Munro) Kurz, J. Asiat. Soc. Bengal, Pt. 2, Nat. Hist. 42: 253. 1873 (as “maclellandii”) **

*Bambusa mcclellandii* Munro, Trans. Linn. Soc. London 26(1): 114. 1868 (as “McClellandi”). Type: “Hab. in Ind. or Pegu, Rangon, Phoungee Valley; M’Clelland v.s.”

**Distribution.** Arunachal Pradesh, Assam, Uttarakhand, West Bengal [Myanmar].

**Habit.** Woody perennial.

**Remarks.** Records for Arunachal Pradesh and Assam fide Naithani (1990).

**94. **Dinochloa nicobariana** R.B. Majumdar in S. Karthikeyan et al., Fl. Ind. ser. 4, 1 (Monocotyledon): 277. 1989 **

**Type.** India: Nicobar Island, North Nicobar, Katchal Island, Coastal Forest; *P. Chakravarty 1129* (CAL).

**Distribution.** Andaman and Nicobar Islands.

**Habit.** Woody perennial.

**95. **Dinochloa scandens** (Blume) Kuntze, Revis. Gen. Pl. 2: 773. 1891 **

*Bambusa scandens* Blume, Flora 7: 291. 1824. Type: Java: Megamendung; *Blume s.n.* (L).

**Distribution.** Andaman and Nicobar Islands. Cultivated; native to Indonesia.

**Habit.** Woody perennial.

***Gigantochloa* Kurz ex Munro, Trans. Linn. Soc. London 26: 123. 1868**

**Lectotype.***Gigantochloa atter* (Hassk.) Kurz ex Munro (*Bambusa vulgaris* var. *atter* Hassk.). LT designated by Holttum in Taxon 5: 28–30. 1956.

**96. **Gigantochloa albociliata** (Munro) Kurz, Prelim. Rep. Forest Pegu, App. A: cxxxvi; App. B: 93. 1875 (as “albo-ciliata”)  **

*Oxytenanthera albociliata* Munro, Trans. Linn. Soc. London 26(1): 129. 1868 (as “albo-ciliata”). Type: “Hab. in India or.” [Burma]: Pegu; *Brandis 19* (“Wapyoogle” incolis). India: Moulmein; *Falconer 27*

**Distribution.** Arunachal Pradesh, Assam, Meghalaya, Tripura, West Bengal [China, Myanmar]. Cultivated, perhaps also naturalized; native to Myanmar.

**Habit. **Woody perennial.

**97. **Gigantochloa andamanica** (Kurz) Kurz, Prelim. Rep. For. Veg. Pegu, App. A. cxxxvi; App. B. 93 in clavi. 1875 **

*Bambusa andamanica* Kurz, J. Asiat. Soc. Bengal n.s. 39 (2): 88. 1870. Type: Andaman Islands, south Andaman; 1875; Kurz s.n.

**Distribution.** Andaman Islands [Myanmar].

**Habit.** Woody perennial.

**Remarks.** Taxonomic editor Clark notes that this name was treated as a synonym of *Gigantochloa nigrociliata* in Vorontsova et al. (2016); see also remarks under that species. The status of this taxon requires further study.

**98. **Gigantochloa apus** (Schult. & Schult. f.) Kurz ex Munro, Trans. Linn. Soc. London 26: 126. 1868 **

*Bambusa apus* Schult. & Schult.f., Syst. Veg., ed. 15 bis [Roemer & Schultes] 7(2): 1353. 1830. Type: Indonesia: “Java ad montem Salaki”; *Blume s.n.* (check author).

*Gigantochloa kurzii* Gamble, Ann. Roy. Bot. Gard. (Calcutta) 7: 65. 1896

**Distribution.** Meghalaya [Myanmar].

**Habit.** Woody perennial.

**99. **Gigantochloa atroviolacea** Widjaja, Reinwardtia 10(3): 323. 1987 **

**Type.** Indonesia: Java, Wonosari, Nagasari; February, 1983; *Ramlanto s.n.* (BO).

**Distribution.** West Bengal. Known only from cultivation; also in Indonesia and Malaysia.

**Habit.** Woody perennial.

**100. **Gigantochloa atte**r (Hassk.) Kurz ex Munro, Trans. Linn. Soc. London 26: 125. 1868 **

*Bambusa vulgaris* Schrad. ex J.C. Wendl. var. *atter* Hassk., Cat. Hort. Bot. Bogor. (Hasskarl) 19. 1844

*Bambusa thouarsii* Kunth var. *atter *Hassk., Pl. Jav. Rar. 41. 1848

**Syntypes.** Indonesia: Java; *Horsfield s.n.* Java: Buitenzorg; *Kurz s.n.*

**Distribution. **West Bengal. Cultivated only. [Indonesia and Malaysia].

**Habit.** Woody perennial.

**101. **Gigantochloa auriculata** (Kurz) Kurz, Prelim. Rep. Forest Pegu, App. A: cxxxvii; App. B: 94. 1875  **

*Bambusa auriculata* Kurz, J. Asiat. Soc. Bengal, Pt. 2, Nat. Hist 39: 86. 1870. Type: Burma: Southern Pegu; *Kurz 20 *(CAL).

**Distribution.** Assam [Bangladesh, Myanmar].

**Habit.** Woody perennial.

**Remarks.** Taxonomic editor Clark notes that Seethalakshmi and Kumar (1998) treat this as a member of the genus *Bambusa*, but Widjaja, quoted in Ohrnberger (1999), discussed it as a species of *Gigantochloa*. The placement here follows Widjaja, but on the basis of the available information, she does not regard the generic placement as settled. Both Wu et al. (2006) and Vorontsova et al. (2016) treat this name as a synonym of *Bambusa vulgaris*.

**102. **Gigantochloa hasskarliana** (Kurz) Backer ex K. Heyne, Nutt. Pl. Ned.-Ind., ed. 2 1: 299. 1927 **

*Schizostachyum hasskarlianum* Kurz, Indian Forester 1: 352. 1876. Syntypes: Indonesia: Java, alt. 2000–3000 ft. Sumatra: Padang

**Distribution.** Chhattisgarh, Madhya Pradesh, cultivated [also in Indonesia, where it is native].

**Habit.** Woody perennial.

**Remarks.** Record for Madhya Pradesh fide Naithani (1990).

**103. **Gigantochloa macrostachya** Kurz, J. Asiat. Soc. Bengal, Pt. 2, Nat. Hist. 42: 251. 1873. **

**Type.** Martaban, Tenasserim

**Distribution.** Arunachal Pradesh, Assam, Meghalaya, Mizoram [Bangladesh, Myanmar].

**Habit.** Woody perennial.

**104. **Gigantochloa nigrociliata** (Buse) Kurz, Indian Forester 1(4): 345, 1876 var. **nigrociliata

*Bambusa nigrociliata* Buse, Pl. Jungh. 389. 1854 (as “Nigro-ciliata”). Type: “Habitat insulam Javae Prope Tjiberem”; *Junghuhn s.n.*

*Oxytenanthera nigrociliata* (Buse) Munro, Trans. Linn. Soc. London 26(1): 228. 1868 (as “nigro-ciliata”)

**Distribution. **Andaman and Nicobar Islands, Kerala, cultivated [Myanmar]. Native to Indonesia.

**Habit.** Woody perennial.

**Remarks.** Taxonomic editor Clark notes that Seethalakshmi and Kumar (1998) consider this to be a synonym of *Gigantochloa rostrata*, but other treatments maintain it as a species. Ohrnberger (1999) indicates that it occurs in the “eastern, central, and north-eastern parts” of India in addition to the Andamans. Dransfield and Widjaja (1995) list *G. nigrociliata* as doubtful for India, suggesting that the Indian records refer to other species. The status of the species and the following variety both need reevaluation in India and generally.

**105. **Gigantochloa nigrociliata** (Buse) Kurz var. **hohenackeri** (Fischer) H. B. Naithani, Fl. Pl. India, Nepal & Bhutan 514. 1990 **

*Oxytenanthera nigrociliata* (Buse) Munro var. *hohenackeri* Fischer, Kew Bull. 1935:96. 1935

**Distribution.** Karnataka. Overall occurrence not understood well.

**Habit.** Woody perennial.

**Remarks.** Record for Karnataka fide Naithani (1990).

**106. **Gigantochloa parvifolia** (Brandis ex Gamble) T.Q. Nguyen, Bot. Zhurn. 75(2): 224. 1990 **

*Pseudoxytenanthera parvifolia* (Brandis ex Gamble) T.Q. Nyugen, Bot. Zurn. (Moscow and Leningrad) 76(7): 993. 1991

*Oxytenanthera parvifolia* Brandis ex Gamble, Ann. Roy. Bot. Gard. (Calcutta) 7: 72. t. 63. 1896. Type: Burma: Yonzalin Valley; March, 1880; *D. Brandis s.n.*

**Distribution.** Assam, Mizoram [Myanmar]. Cultivated.

**Habit.** Woody perennial.

**Remarks.** Records for Assam and Mizoram fide Naithani (1990).

**107. **Gigantochloa pseudoarundinacea** (Steud.) Widjaja, Reinwardtia 10(3): 305. 1987 **

*Bambusa pseudoarundinacea* Steud., Syn. Pl. Glumac. 1: 330. 1854. Type: Indonesia: Java; *H. Zollinger 3479 *(L).

*Bambusa verticillata* Willd., Sp. Pl., ed. 4 [Willdenow] 2: 245. 1799

*Gigantochloa verticillata* (Willd.) Munro, Trans. Linn. Soc. London 26(1): 124. 1868

*Nastus verticillatus* (Willd.) Sm., Cycl. (Rees) 24: n. 3. 1819

**Distribution.** West Bengal [Myanmar]. Known only in cultivation.

**Habit. **Woody perennial.

**108. **Gigantochloa rostrata** K.M. Wong, Malaysian Forester 45(3): 349. 1982 **

**Type.** Malaya; October 15, 1980; *K.M. Wong KEP FRI 28981* (L).

**Distribution.** Andaman and Nicobar Islands, Assam, Bihar, Karnataka, Madhya Pradesh, Maharashtra, Meghalaya, Odisha, Tripura [Bangladesh]. Known only in cultivation.

**Habit.** Woody perennial.

**Remarks.** Taxonomic editor Clark notes that “Kumari and Singh (2014) synonymize *G. nigrociliata* under this species, and in the discussion mention the *G. nigrociliata* of Widjaja. This needs to be addressed, as noted under* G. nigrociliata*.”

**109. **Gigantochloa tekserah** E.G. Camus, Bamb. 141. 1913 **

**Type.** Garo Hills; 1889; *G. Mann s.n. *(?).

**Distribution.** Meghalaya.

**Habit.** Woody perennial.

**Remarks.** Taxonomic editor Clark notes that this species is not mentioned by Kumari and Singh (2014), and furthermore, that this name is treated as a synonym of *Gigantochloa macrostachya* by Vorontsova et al. (2016).

***Melocalamus* Benth. in Benth. & J.D. Hooker, Gen. Pl. 3, 2: 1095, 1212. 1883**

**Type.***Melocalamus compactiflorus* (Kurz) Benth. (*Pseudostachyum compactiflorum* Kurz).

**110. **Melocalamus compactiflorus** (Kurz) Benth., Gen. Pl. (Bentham & Hooker f.) 3: 1212. 1883 **

*Pseudostachyum compactiflorum* Kurz, J. Asiat. Soc. Bengal, Pt. 2, Nat. Hist. 42: 252. 1873. Type: ?

*Dinochloa compactiflora* (Kurz) McClure, Bull. Misc. Inform. Kew 1936: 253. 1936

**Distribution.** Arunachal Pradesh, Assam, Manipur, Meghalaya, Mizoram, Nagaland, Tripura [Bangladesh, China, Myanmar].

**Habit.** Woody perennial.

**111. **Melocalamus indicus** R.B. Majumdar, Bull. Bot. Surv. India 25(1–4): 236. 1985 (“1983”) **

*Dinochloa indica* (R.B. Majumdar) Bennet, Van Vigyan 27(2): 121. 1989

**Type.** India: Cachar, Bhuban Hills; *Majumder 73083* (CAL).

**Distribution. **Assam, Manipur.

**Habit.** Woody perennial.

**Remarks.** Records for India fide Naithani (1990).

**112. **Melocalamus mastersii** (Munro) R.B. Majumdar, in S. Karthikeyan et al., Fl. Ind. ser. 4, 1 (Monocotyledon): 278. 1989 **

*Bambusa mastersii* Munro, Trans. Linn. Soc. London 26(1): 113. 1868. Type: India: Assam, Dibrooghur; *Masters 1123*

*Melocalamus gracilis* R.B. Majumdar in S. Karthikeyan et al., Fl. Ind. ser. 4, 1 (Monocotyledon): 278. 1989. Type: India: Barail Range, near Kailana, 9 km from Gumri rest house on Shillong, Cachar Road near P.W.D. Shed; *R.B. Majumdar 1138* (CAL).

*Dinochloa gracilis* (R.B. Majumdar) Bennet & Jain ex D.N. Tewari, Monogr. Bamboo 82. 1992.

**Distribution.** Assam.

**Habit.** Woody perennial.

***Melocanna* Trin. in K.P.J. Sprengel, Neue Entdeck. Pflanzenk. 2: 43. 1820 (sero) (“1821”)**

**Type. ***Melocanna bambusoides* Trin., *nom. illeg.* (*Bambusa baccifera* Roxb.; *Melocanna baccifera* (Roxb.) Kurz).

**113. **Melocanna baccifera** (Roxb.) Kurz, Prelim. Rep. Forest Pegu App. B: 94. 1875, nom. cons. **

*Bambusa baccifera* Roxb., Pl. Coromandel 3: 37, t. 243. 1795. Lectotype: *Roxburgh, Icones 1401* (K). LT designated by Stapleton in Edinburgh J. Bot. 51: 27. 1994.

**Distribution.** Assam, Karnataka, Maharashtra, Manipur, Meghalaya, Mizoram, Sikkim, Tripura, West Bengal [Bangladesh, China, Pakistan].

**Habit.** Woody perennial.

**114. **Melocanna clarkei** (Gamble ex Brandis) P. Kumari & P. Singh, Nelumbo 51: 234. 2009 **

*Arundinaria clarkei* Gamble ex Brandis, Indian Trees 666. 1906 (Note: considered to be an invalid name *fide* Stapleton, 1994).

*Neomicrocalamus clarkei* (Gamble ex Brandis) R.B. Majumdar in S. Karthikeyan et al., Fl. Ind. ser. 4, 1 (Monocotyledon): 279. 1989

*Racemobambos clarkei* (Gamble ex Brandis) M. Kumar, Bamboos of India, Compendium 230. 1998

*Schizostachyum mannii* R.B. Majumdar in S. Karthikeyan et al., Fl. Ind. ser. 4, 1 (Monocotyledon) 281. 1989. Type: India: Jowai, Khasia & Jaintia Hills; *G. Mann 31982* (CAL).

*Bambusa khasiana *sensu Gamble, Ann. Roy. Bot. Gard. (Calcutta) 7: 39. t. 37. 1896, non Munro, 1868.

**Distribution.** Assam, Meghalaya Manipur, Sikkim.

**Habit.** Woody perennial.

**Remarks.** Record for Manipur fide Naithani (1990). Taxonomic editor Clark notes that *S. mannii* has been synonymized with *M. clarkei* by Kumari and Singh (2014).

**115. **Melocanna humilis** Kurz, J. Asiat. Soc. Bengal, Pt. 2, Nat. Hist. 42(4): 251. 1873, non Roep. ex Trin., 1822 **

*Melocanna arundina* C.E. Parkinson, Indian Forester 61: 326. 1935

**Distribution.** Assam, West Bengal [China, Myanmar, Thailand].

**Habit.** Woody perennial.

**Remarks.** Record for Assam fide Naithani (1990). Taxonomic editor Clark notes that “Kumari and Singh (2014) indicate that there are two species of *Melocanna*, *M. baccifera* and *M. clarkei*, and that both occur in Meghalaya, but they do not make any reference to *M. humilis*.”

***Neohouzeaua* A. Camus, Bull. Mus. Nation. Hist. Nat. Paris 28: 100. 1922**

**Type. **LT.: *Neohouzeaua mekongensis* A. Camus (vide McClure, Taxon 6: 206. 1957).

**116. **Neohouzeaua dullooa** (Gamble) A. Camus, Bull. Mus. Hist. Nat. (Paris) 28: 101. 1922 **

*Teinostachyum dullooa* Gamble, Ann. Roy. Bot. Gard. (Calcutta) 7: 101. 1896 (as “dulloa”). Type: Burma: Katha District, Hawyaw Monastery Garden; February, 1892; *Oliver s.n.* (K).

*Schizostachyum dullooa* (Gamble) R.B. Majumdar in S. Karthikeyan et al., Fl. Ind. ser. 4, 1 (Monocotyledon): 281. 1989

**Distribution.** Assam, Manipur, Meghalaya, Mizoram, Nagaland, Sikkim, Uttarakhand, West Bengal [Bangladesh, Bhutan, Myanmar].

**Habit.** Woody perennial.

**117. **Neohouzeaua helferi** (Munro) Gamble, Bull. Misc. Inform. Kew 1923: 91. 1923 **

*Bambusa helferi* Munro, Trans. Linn. Soc. London 26(1): 114. 1868. Type: Burma: Tenasserim; *Helfer 411*

*Pseudostachyum helferi* (Munro) Kurz, J. Asiat. Soc. Bengal, Pt. 2, Nat. Hist. 42: 253. 1872

*Teinostachyum helferi* (Munro) Gamble, Ann. Roy. Bot. Gard. (Calcutta) 7: 102. t. 90. 1896

*Schizostachyum helferi* (Munro) R.B. Majumdar in S. Karthikeyan, Fl. Ind. ser. 4, 1 (Monocotyledon): 281. 1989

**Distribution.** Arunachal Pradesh, Assam, Meghalaya [Myanmar].

**Habit.** Woody perennial.

***Neololeba* Widjaja, Reinwardtia 11(2): 112–113. 1997**

**Type. ***Neololeba atra* (Lindl.) Widjaja (*Bambusa atra* Lindl.).

**118. **Neololeba atra** (Lindl.) Widjaja, Reinwardtia 11(2): 114. 1997. **

*Bambusa atra* Lindl., Penny, Cyclop. 3: 357. 1835. Type: found in Amboyna

*Bambusa lineata* Munro, Trans. Linn. Soc. London 26(1): 118. 1868. Type: “Hab. in Ins. Ternate et Celebe. In Amboina exotica, Rumph.”

**Distribution.** Andaman and Nicobar Islands. Cultivated and perhaps also naturalized; native to New Guinea, the Moluccas and Sangihe Island and possibly also the Philippines.

**Habit.** Woody perennial.

**Remarks.** Taxonomic editor Clark notes that this name has many other synonyms in the literature; see Ohrnberger (1999) and Vorontsova et al. (2016).

***Neomicrocalamus* P.C. Keng, J. Bamboo Res. 2(2): 10. 1983**

**Type. ***Neomicrocalamus prainii* (Gamble) Keng f. (*Microcalamus prainii* Gamble). Substitute name for *Microcalamus* J.S. Gamble 1890, non A.R. Franchet 1889.

**119. **Neomicrocalamus andropogonifolius** (Griff.) Stapleton, Edinburgh J. Bot. 51(3): 325. 1994 **

*Bambusa andropogonifolia* Griff., Itin. Pl. Khasyah Mts. 124. 1848. Type: Bhutan, Tashigang District, Diri Chhu, [27°10'N, 91°26'E], alt. 3600 ft.; *Griffith Itin 417* (K).

**Distribution.** Nagaland [Bhutan].

**Habit.** Woody perennial.

**120. **Neomicrocalamus prainii** (Gamble) Keng f., J. Bamboo Res. 2(2): 10. 1983 **

*Microcalamus prainii* Gamble, J. Asiat. Soc. Bengal, Pt. 2, Nat. Hist. 59(2): 207. pl. 7. 1890. Type: India: Assam, Naga Hills, 8300 ft.; April 23, 1886; *Prain s.n.* (K).

*Arundinaria prainii *(Gamble) Gamble, Ann. Roy. Bot. Gard. (Calcutta) 7: 21. t. 19. 1896

*Racemobambos prainii* (Gamble) Keng f. & T.H. Wen, J. Bamboo Res. 5(2): 13. 1986

**Distribution.** Arunachal Pradesh, Assam, Meghalaya, Nagaland [China].

**Habit.** Woody perennial.

***Ochlandra* Thwaites, Enum. Pl. Zeyl. (Thwaites). 376. 1864**

**Type. ***Ochlandra stridula* Thwaites.

**121. **Ochlandra beddomei** Gamble, Ann. Roy. Bot. Gard. (Calcutta) 7: 124. t. 110. 1896 **

**Type.** India: Wynaad; *Beddome s.n.*

**Distribution.** Kerala, Tamil Nadu.

**Habit. **Woody perennial.

**122. **Ochlandra ebracteata** Raizada & Chatterjee, Indian Forester 89(5): 362. 1963 **

**Type.** India: Kerala, Parithipally range, Kottur reserve, Trivandrum division; June 28, 1961; *Managing Agents, Punalur Paper Mills s.n.* (DD).

**Distribution.** Kerala, Tamil Nadu.

**Habit.** Perennial.

**Remarks.** Record for Kerala fide Naithani (1990).

**123. **Ochlandra keralensis** M. Kumar, Remesh & Sequiera, J. Econ. Taxon. Bot. 25(1): 49–51. f. 1(A–N). 2001 **

**Type.** India: Kerala, Pathanamthitta District, Pachakkanam, 3200 ft.; December 9, 1998; *Remesh; Stephen 20730* (KFRI).

**Distribution.** Kerala.

**Habit.** Woody perennial.

**124. **Ochlandra scriptoria** (Dennst.) C.E.C. Fisch., Fl. Madras 3(10): 1863. 1934  **

*Bambusa scriptoria* Dennst., Schlussel Hort. Malab. 31. 1818. Type: Rheede, Hortus. Malab. 3. t. 60 as “Beesha”

*Beesha rheedii* Kunth, Enum. Pl. 1: 434. 1833. Type: Rheede, Hortus. Malab. 5. 119. t. 60 as “Beesha”

*Ochlandra rheedii* (Kunth) Benth. & Hook. f. ex Gamble, Ann. Roy. Bot. Gard. (Calcutta) 7: 121. t. 107. 1896

**Distribution.** Karnataka, Kerala, Tamil Nadu.

**Habit.** Woody perennial.

**125. **Ochlandra setigera** Gamble, Ann. Roy. Bot. Gard. (Calcutta) 7: 128. t. 115. 1896 **

**Type.** India: western slopes of Nilgiri Hills, Gudalur, alt. 3000 ft.; *H. Trimen s.n.*

**Distribution.** Kerala, Tamil Nadu.

**Habit.** Woody perennial.

**126. **Ochlandra sivagiriana** (Gamble) E.G. Camus, Bambusees (Camus) 181. t. 99. f. c. 1913 **

*Ochlandra rheedii* Benth. ex Gamble var. *sivagiriana* Gamble, Ann. Roy. Bot. Gard. (Calcutta) 7: 122. t. 108. 1896. Syntypes: India: Sivagiri Hills, 4000–4500 ft.; *Gamble s.n.* (K). Pulney Hills, alt. 1300–1400 ft.; 1873; *Beddome s.n.*

*Ochlandra scriptoria* (Dennst.) Fisch. var. *sivagiriana* (Gamble) C.E.C. Fisch., Fl. Madras 3: 1863. 1934

**Distribution.** Kerala, Tamil Nadu.

**Habit.** Woody perennial.

**127. **Ochlandra soderstromiana** M. Kumar & Sequiera, Rheedea 9(1): 33. f. 2. 1999 **

**Type.** India: Kerala, Idukki District, Kallar, Kallar Valley Estate, alt. 3200 ft.; June 15, 1998; *Stephen Sequiera 008883* (KFRI).

**Distribution.** Kerala.

**Habit.** Woody perennial.

**128. **Ochlandra spirostylis** M. Kumar, K.K. Seethal. & Sequiera, Rheedea 9(1): 31–33. 1999 **

**Type.** India: Kerala, Idukki District, Adimali, Chattuparakudy, 3000 ft.; May 30, 1998; *Stephen Sequiera 008884* (KFRI).

**Distribution.** Kerala.

**Habit. **Woody perennial.

**129. **Ochlandra talbotii **Brandis, Indian Trees 684. 1906 (as “talboti”) **

**Type.** India: North Kanara; *Brandis s.n.*

**Distribution.** Karnataka.

**Habit.** Woody perennial.

**130. **Ochlandra travancorica** (Bedd.) Benth. ex Gamble, Ann. Roy. Bot. Gard. (Calcutta) 7: 125. t. 111. 1896 var. **travancorica

*Beesha travancorica* Bedd., Forester’s Man. Bot. 234. 1873. Type: India: Mountains of Tinnevelly and Travancore, alt. 3000–5000 ft., Madras; *Beddome s.n.*

**Distribution.** Karnataka, Kerala, Tamil Nadu [Sri Lanka].

**Habit.** Woody perennial.

**131. **Ochlandra travancorica** (Bedd.) Benth. ex Gamble var. hirsuta Gamble, Ann. Roy. Bot. Gard. (Calcutta) 7: 126. t. 112. 1896 **

**Type. **India: Travancore Hills; *Beddome s.n.*

**Distribution.** Kerala.

**Habit. **Woody perennial.

**132. **Ochlandra wightii** (Munro) C.E.C. Fisch., Fl. Madras 3(10): 1864. 1934 **

*Bambusa wightii* Munro, Trans. Linn. Soc. London 26(1): 111. 1868. Syntypes: India: Courtallum; *Wight 1009*. Malabar; *Wight 117, Wight 1346.*

*Ochlandra brandisii* Gamble, Ann. Roy. Bot. Gard. (Calcutta) 7: 126. t. 113. 1896. Type: India: Tinnevelly Ghats, Courtallum, alt. 3000 ft.; 1835; *Wight 1009*

**Distribution.** Kerala, Tamil Nadu.

**Habit.** Woody perennial.

**Remarks. ***Ochlandra kadambaranii*. Taxonomic editor Clark suggests that the name *Ochlandra kadambaranii*, for which UmaShaanker et al. (2004) provide a distribution map, is probably a *nomen nudum*, but this needs to be checked.

***Oxytenanthera* Munro, Trans. Linn. Soc. London 26(1): 126. 1868**

**Lectotype.***Oxytenanthera abyssinica* (A. Rich.) Munro (*Bambusa abyssinica* A. Rich.). LT designated by McClure in Taxon 6: 206. 1957.

**133. **Oxytenanthera abyssinica** (A. Rich.) Munro, Trans. Linn. Soc. London 26(1): 127. 1868 **

*Bambusa abyssinica* A. Rich., Tent. Fl. Abyss. 2: 439. 1850–1851. Syntypes: Ethiopia: near Djeladjeranne [Tchelatchekanne]; December 15, 1839; *G.H.W. Schimper s.n. [501]* (P). Ethiopia: Banks of R. Tacazze; *Quartin Dillon & Petit s.n. *(P). Ethiopia: Aderbati; *Quartin Dillon & Petit s.n.* (P).

**Distribution.** Uttarakhand. Cultivated; native to tropical Africa.

**Habit.** Woody perennial.

***Pseudobambusa* Nguyen, Bot. Zurn. 76(7): 992. 1991**

**Type. ***Pseudobambusa kurzii* (Munro) Ohrnb.

**134. **Pseudobambusa kurzii** (Munro) Ohrnb., Bamb. World Introd. Ed. 4: 19. 1997 **

*Melocanna kurzii* Munro, Trans. Linn. Soc. London 26(1): 134. 1868. Type: India: Andaman Island; *Kurz s.n.*

*Bambusa kurzii* (Munro) N.P. Balakr., Bull. Bot. Surv. India 22(1–4): 176. 1982 (“1980”)

*Schizostachyum kurzii* (Munro) R.B. Majumdar in S. Karthikeyan, Fl. Ind. ser. 4, 1 (Monocotyledon): 281. 1989

*Bambusa schizostachyoides* Kurz in Gamble, Ann. Roy. Bot. Gard. (Calcutta) 7: 48, t. 44. 1896, nom. superfl. & illeg.

*Teinostachyum schizostachyoides* Kurz, J. Asiat. Soc. Bengal, Pt. 2, Nat. Hist. 39: 89. 1870, nom. superfl. & illeg.

*Cephalostachyum schizostachyoides* Kurz, Prelim. Rep. Forest Pegu Append. App. B. 94., in clavi, A. A., 137, 1875, nom. superfl. & illeg.

**Distribution.** Andaman and Nicobar Islands, Manipur [Myanmar, Vietnam].

**Habit. **Woody perennial.

**Remarks.** Taxonomic editor Clark observes that Manipur seems unlikely to be part of the native range, since this species is native to the southern part of Myanmar and also the southern part of Vietnam, as well the Andamans.

***Pseudostachyum* Munro, Trans. Linn. Soc. London 26: 141. 1868**

**Type. ***Pseudostachyum polymorphum* Munro.

**135. **Pseudostachyum polymorphum** Munro, Trans. Linn. Soc. London 26(1): 142. t. 4. 1868 **

*Schizostachyum polymorphum* (Munro) R.B. Majumdar in S. Karthikeyan et al., Fl. Ind. ser. 4, 1 (Monocotyledon): 282. 1989

**Lectotype.** India: Assam, Nigrigam; January 18, 1836; *Griffith 6735* (K). LT designated by Stapleton in Edinburgh J. Bot. 51: 30. 1994.

**Distribution.** Arunachal Pradesh, Assam, Manipur, Meghalaya, Mizoram, Nagaland, Sikkim, West Bengal [Bhutan, China, Myanmar, Nepal].

**Habit.** Woody perennial.

***Pseudoxytenanthera* Soderstr. & R.P. Ellis, Smithsonian Contr. Bot. 72: 52. 1988**

**Type.***Pseudoxytenanthera monadelpha* (Thwaites) Soderstr. & R.P. Ellis (*Dendrocalamus monadelphus* Thwaites).

**136. **Pseudoxytenanthera bourdillonii** (Gamble) H.B. Naithani, J. Bombay Nat. Hist. Soc. 87: 440. 1991 (“1990”) **

*Oxytenanthera bourdillonii* Gamble, Ann. Roy. Bot. Gard. (Calcutta) 7: 76. 1896 (as “ bourdilloni”). Type: “Western Ghats of Travancore, grows only on steep precipitous places and wet rocks at elevations of 3000–4000 ft.”; *J.F. Bourdillon s.n.*

**Distribution.** Kerala.

**Habit.** Woody perennial.

**137. **Pseudoxytenanthera monadelpha** (Thwaites) Soderstr. & R.P. Ellis, Smithsonian Contr. Bot. 72: 52. 1988 **

*Dendrocalamus monadelphus* Thwaites, Enum. Pl. Zeyl. 5: 376. 1864. Lectotype: Sri Lanka: Ambagamuwa; December, 1854; *Thwaites C.P. 3359* (PDA). LT designated by Soderstrom and Ellis in Smithsonian Contr. Bot. 72: 52. 1988.

**Distribution.** Andhra Pradesh, Karnataka, Kerala, Tamil Nadu [Sri Lanka].

**Habit.** Woody perennial.

**138. **Pseudoxytenanthera ritcheyi** (Munro) H.B. Naithani, J. Bombay Nat. Hist. Soc. 87(3): 440. 1991 (“1990”) **

*Bambusa ritcheyi* Munro, Trans. Linn. Soc. London 26(1): 113. 1868. Type: India: Bombay, Kala Nuddi; *Ritchie 820*

**Distribution.** Andhra Pradesh, Karnataka, Kerala, Maharashtra, Tamil Nadu.

**Habit.** Woody perennial.

**139. **Pseudoxytenanthera stocksii** (Munro) H.B. Naithani, J. Bombay Nat. Hist. Soc. 87(3): 440 1991 (“1990”) **

*Oxytenanthera stocksii* Munro, Trans. Linn. Soc. London 26(1): 130. 1868. Type: India: Concan; *Stocks s.n.*

*Dendrocalamus stocksii* (Munro) M. Kumar, Remesh & Unnikr. Sida 21(1): 95. 2004

**Distribution.** Goa, Karnataka, Kerala, Maharashtra [also in Vietnam]. Cultivated, or perhaps also native.

**Habit.** Woody perennial.

***Schizostachyum* Nees, Fl. Bras. Enum. Pl. 2(1): 535. 1829**

**Type. ***Schizostachyum blumei* Nees.

**140. **Schizostachyum brachycladum** (Kurz ex Munro) Kurz, J. Asiat. Soc. Bengal, Pt. 2, Nat. Hist. 39(2): 89. t. 6. f. 2. 29. 1870 **

*Melocanna zollingeri* (Steud.) Kurz ex Munro var. *brachyclada* Kurz ex Munro, Trans. Linn. Soc. London 26(1): 134. 1868

*Schizostachyum zollingeri* Steud., Syn. Pl. Glumac. 1: 332. 1854. Type: Java

**Distribution.** West Bengal [also in Indonesia, Malaysia, Philippines, Singapore]. Probably only cultivated in India.

**Habit.** Woody perennial.

**141. **Schizostachyum rogersii** Brandis, Indian Trees 679. 1906 **

**Type. **India: Andamans; *G. Rogers s.n.*

**Distribution.** Andaman and Nicobar Islands, Assam, Nagaland.

**Habit.** Woody perennial.

**Remarks.** Taxonomic editor Clark notes that Seethalakshmi and Kumar (1998) consider this species to be endemic to the Andamans, suggesting that the mainland records refer to cultivated plants or are erroneous.

**142. **Schizostachyum virgatum** (Munro) H.B. Naithani & Bennet, Indian Forester 117(1): 68. 1991 **

*Melocanna virgata* Munro, Trans. Linn. Soc. London 26(1): 133. 1868. Type: Hab. in India or Burmah, Keouksik ad fluv. Mogong; *Griffith s.n.* (K).

*Cephalostachyum virgatum* (Munro) Kurz, Prelim. Rep. Forest Pegu App. A 137: 1875

**Distribution.** India, cultivated [China, Myanmar].

**Habit. **Woody perennial.

***Stapletonia* P. Singh, S.S. Dash, & P. Kumari, Nelumbo 51: 241. 2009**

**Type. ***Stapletonia arunachalensis* (H.B. Naithani) P. Singh, S.S. Dash & P. Kumari (*Schizostachyumarunachalense* H.B. Naithani).

**143. **Stapletonia arunachalensis** (H.B. Naithani) P. Singh, S.S. Dash & P. Kumari, Nelumbo 51: 241. 2009 **

*Schizostachyum arunachalensis* H.B. Naithani, Indian Forester 118(3): 230. 1992. Type: India: Arunachal Pradesh: Subansiri District, Baja near Daporijo, 300 m; 17.3.1986; *H.B. Naithani 1406* (holotype, DD).

**Distribution.** Arunachal Pradesh.

**Habit.** Woody perennial.

**144. **Stapletonia rigoensis** L. B. Singha, P. Niri, & R. Devi, Phytotaxa 350(1): 82. 2018 **

**Type. **India: Arunachal Pradesh, Near Rigo village, 400 – 500 m, 001/LBS/2017 (holotype: ASSAM).

**Distribution.** Arunachal Pradesh.

**Habit.** Perennial

**145. **Stapletonia seshagiriana** (R.B. Majumdar) H.B. Naithani, Indian Forester 139: 1048. 2013 **

*Schizostachyum seshagirianum* R.B. Majumdar in S. Karthikeyan et al., Fl. Ind. ser. 4, 1 (Monocotyledon): 282. 1989. Type: India: Arunachal Pradesh, Garsing to Eyo, Spiang, alt. 1900 ft.; *R.S. Rao 1794* (CAL).

**Distribution.** Arunachal Pradesh.

**Habit.** Woody perennial.

***Teinostachyum* Munro, Trans. Linn. Soc. London 26: 142. 1868**

**Lectotype.***Teinostachyum griffithii *Munro (designated by McClure, Taxon 6: 209. 1957).

**146. **Teinostachyum beddomei** C.E.C. Fisch., Fl. Madras 3(9): 1860. 1934 **

*Schizostachyum beddomei* (C.E.C. Fisch.) R.B. Majumdar in S. Karthikeyan, Fl. Ind. ser. 4, 1 (Monocotyledon): 281. 1989

*Bambusa wightii* Munro, Trans. Linn. Soc. London 26(1): 111. 1868. Syntypes: India; *R.H. Beddome 117, 1009, 1346 *(US-79430). Hab. in Ind. or. Courtallum; *Wight 1009*. India: Malabar; *Wight 117*. India; *Wight 1346*

*Teinostachyum wightii* (Munro) Bedd., Fl. Sylv. S. India t. 323. 1873

**Type. **Nilgiri and Travancore Hills, alt. 3000–5000 ft.

**Distribution.** Karnataka, Kerala, Tamil Nadu.

**Habit.** Woody perennial.

**Remarks.** Taxonomic editor Clark notes that the nomenclature here needs to be investigated and the type material studied. The name *B. wightii* has several syntypes and was based on vegetative material only, so it is unclear what species name really applies to although it appears to be validly published. In addition, *T. wightii* may be misapplied *fide*Ohrnberger (1999).

**147. **Teinostachyum griffithii** Munro, Trans. Linn. Soc. London 26: 143, t. 3. 1868 **

*Schizostachyum griffithii* (Munro) R.B. Majumdar in S. Karthikeyan, Fl. Ind. ser. 4, 1 (Monocotyledon): 281. 1989

**Type. **Burma: Prope Wulloboom “in sylvis collinis”; *Griffith s.n.*

**Distribution.** Arunachal Pradesh, Assam, Meghalaya [Myanmar].

**Habit.** Woody perennial.

***Thyrsostachys* Gamble, Indian Forester 20: 1. 1894**

**Type. ***Thyrsostachys oliveri* Gamble.

**148. **Thyrsostachys oliveri** Gamble, Indian Forester 20: 1. 1894 **

**Type.** “The bamboo was discovered by Mr. J.W. Oliver, conservator of forests of the eastern circile of Upper Burma and the photograph from the negative of which our picture has been obtained, was taken by him.” Burma; *J.W. Oliver s.n.*

**Distribution.** Arunachal Pradesh, Manipur, Uttar Pradesh, Uttarakhand, West Bengal [China, Myanmar].

**Habit. **Woody perennial.

**Remarks.** Taxonomic editor Clark notes that this species is cultivated in the Indian Botanical Garden, Calcutta and Forest Research Institute, Dehra Dun.

**149. **Thyrsostachys siamensis** Gamble, Ann. Roy. Bot. Gard. (Calcutta) 7: 59–60. t. 51. 1896, nom. cons. **

*Bambusa regia* Thomson ex Munro, Trans. Linn. Soc. London 26(1): 116. 1868 Lectotype: *Kurz s.n.* (K). LT designated by Stapleton in Taxon 47: 739. 1998.

*Thyrsostachys regia* (Thomson ex Munro) Bennet, Indian Forester 114(10): 711. 1988

**Distribution.** Uttarakhand, West Bengal [China, Myanmar]. Probably only cultivated in India.

**Habit.** Woody perennial.

**Remarks.** Taxonomic editor Clark notes that this species is cultivated in the Indian Botanical Garden, Calcutta and Forest Research Institute, Dehra Dun.

### Subfamily Oryzoideae Kunth x Beilschm. (1833)

Plants perennial, generally herbaceous and with or without rhizomes or stolons. Spikelets with *2 glumes, or the glumes lacking. Flowers 3, the proximal two sterile and only the distal one fertile*. Lemma with or without an awn. Lodicules 2. Fusoid cells absent or present, mesophyll cells with or without invaginated cell walls, midrib simple or complex.

Within Oryzoideae, Ehrharteae and Oryzeae are clearly sisters (e.g., (Kellogg 2009; Saarela et al. 2018; Tang et al. 2010)). Members of these two tribes have characteristic “oryzoid” silica bodies in the leaves. These are narrow and elongated perpendicular to the proximo-distal axis of the leaf and form over the veins, which is an unusual position for silica bodies (Metcalfe 1960). Most species have a chromosome base number of *x *= 12. All Oryzeae and many Ehrharteae are plants of moist habitats, a character that may be ancestral (Kellogg 2015b).

In contrast to Ehrharteae and Oryzeae, the position of *Streptogyna* has long been controversial. However, recent phylogenetic work using complete plastomes places it sister to the Ehrharteae + Oryzeae clade (Saarela et al. 2018). It lacks the distinctive characters of the other two tribes having multi-flowered spikelets and lacking the distinctive silica bodies. Nuclear phylogenies place it sister to the BOP clade (Mathews et al. 2000) or to Bambusoideae (Hisamoto et al. 2008).

Taxonomic editor: G. Anthony Verboom, University of Cape Town

### Tribe Ehrharteae Nevski, 1937

Ehrharteae has no obvious diagnostic character, although the simple vasculature in the midrib and lack of an epiblast in the embryo might be synapomorphic (Kellogg 2015b). The plants vary in habit, presence or absence of auricles in the leaves, and ligule morphology. Stamen number also varies, with individual species having 2, 3, 4, or 6 stamens. This character has been used in the past to segregate genera from *Ehrharta* but phylogenetic studies (Verboom et al. 2003) show that all species are best placed in a single genus.

***Ehrharta* Thunb., Kongl. Vetensk. Acad. Handl. 40: 217. t. 8. 1779, nom. cons**. **[incl. **Microlaena** R. Br., **Tetrarrhena** R. Br., **Zotovia** Edgar & Connor] **

**Type. ***Ehrharta capensis* Thunb.

**150. **Ehrharta abyssinica** Hochst., Flora 38: 193. 1855 **

*Ehrharta erecta* Lam. var. *abyssinica* (Hochst.) Pilg., Notizbl. Bot. Gart. Berlin-Dahlem 9(87): 508. 1926. Type: Abyssina; *Buchinger 1460*

**Type.** Abyssina [Ethiopia]; 1853; *Schimper, Hb. abyss. Buchinger 1460 *(STR).

**Distribution.** Tamil Nadu, presumably naturalized. Native to Africa.

**Habit.** Perennial.

**Remarks.** Taxonomic editor Verboom notes that this species may or may not be distinct from *E. erecta*, and might be treated better as a variety of that species.

**151. **Ehrharta calycina** Sm., Pl. Icon. Ined. 2: t. 33. 1790**

*Trochera calycina* (Sm.) P. Beauv., Ess. Agrostogr. 62. t. 12 f. 4. 1812

*Ehrharta auriculata* Steud., Flora 12: 491. 1829

*Melica geniculata* Thunb., Prodr. Pl. Cap. 1: 21. 1794. Type: Promontorio Bonae Spei Africas. [South Africa: Cape of Good Hope]; *Thunberg s.n.*

*Ehrharta geniculata* (Thunb.) Thunb., Prodr. Pl. Cap. 2: 192. 1800

*Ehrharta ovata* Nees, Linnaea 7: 336. 1832. Type: “Sandige Flache unter dem Tigerberge bei Duiker vallei.”; *Ecklon s.n.*

*Ehrharta paniculata* Poir., Encycl. (Lamarck) Suppl. 2: 542. 1812

*Ehrharta adscendens* Schrad. ex Schult. & Schult. f., Syst. Veg., ed. 15 bis [Roemer & Schultes] 7(2): 1372. 1830 pro syn.

*Ehrharta undulata* Nees ex Trin., Mém. Acad. Imp. Sci. Saint-Pétersbourg, Sér. 6, Sci. Math., Seconde Pt. Sci. Nat. 5, 3(3): 70. 1839, nom. nud., pro syn.

*Melica festucoides* Licht. ex Trin., Mém. Acad. Imp. Sci. Saint-Pétersbourg, Sér. 6, Sci. Math., Seconde Pt. Sci. Nat. 5, 3(3): 70. 1839, pro syn. *Ehrharta calycina* Sm.

*Ehrharta mnemateia* Thunb., Prodr. Pl. Cap. 1: 66. 1794, non L.f., 1782. Type: Africa; *Thunberg s.n.*

*Ehrharta laxiflora* sensu Schult. & Schult. f., Syst. Veg., ed. 15 bis [Roemer & Schultes] 7(2): 1373. 1830, non Schrad., 1821

*Aira capensis* L.f., Suppl. Pl. 108. 1782 (“1781”), non *Ehrharta capensis* Thunb., 1779. Type: “Habitat ad Cap. Bonae spei, Sparrmann.”

**Distribution.** India. Naturalized, but state localities not reported. Native to Africa, and also naturalized in Australasia, South America.

**Habit.** Annual or perennial.

**152. **Ehrharta capensis** Thunb., Kongl. Vetensk. Acad. Handl. 40: 217. t. 8. 1779 **

*Ehrharta mnemateia* L.f., Suppl. Pl. 209. 1782 (“1781”). Type: “Habitat in Africa.” *D.D. Thunberg s.n.*

*Ehrharta nutans* Lam., Encycl. (Lamarck) 2(1): 346. 1786, nom. superfl. & illeg. for *E. capensis*. Type: “Cette plante croit au Cap de Bonne-Esperance, & nous a été communiquée par M. Sonnerat, ainsi que celle qui fuit.”

**Type. **“Herr Ehrhart vistas nu I Hannover.”

**Distribution.** India. Naturalized, but state localities not reported. Native to Africa.

**Habit.** Perennial.

**153. **Ehrharta erecta** Lam., Encycl. (Lamarck) 2(1): 347. 1786 **

*Ehrharta panicea* Sm., Pl. Icon. Ined. t. 9. 1789. Type: “Ex Capite Bonae Spei adtulit Sonnerat anno 1776.” *Sonnerat s.n.* (Herb. Thouin).

*Ehrharta paniciformis* Nees ex Trin., Mém. Acad. Imp. Sci. Saint-Pétersbourg, Sér. 6, Sci. Math., Seconde Pt. Sci. Nat. 5, 3(3): 64. 1839. Type: not given

*Panicum deflexum* Guss., Fl. Napol. 5: 320. 1835, nom. later hom., non Schumach, 1827

*Trochera panicea* Baill., Hist. Pl. (Baillon) 12: 171, f. 313, 314. 1894, non (Sm.) Kuntze, 1891

**Type.** “Cette plante se trouve fans aucune etiquette dans la collection de Graminees que M. Sonnerat nous a communiquee; mais nous ne savons pas positivement si ell est du Cap, comme la precedente ou si elle est de l’Inde.”

**Distribution.** India. Naturalized, but state localities not reported. Native to southern Africa, and also naturalized in China.

**Habit.** Perennial.

**154. **Ehrharta longiflora** Sm., Pl. Icon. Ined. 2: t. 32. 1790 **

*Ehrharta aristata* Thunb., Prodr. Pl. Cap. 1: 66. 1794. Type: Promontoria Bonae Spei Africes; 1772–1775; *Thurnberg s.n.*

*Ehrharta eckloniana* Schrad. ex Schult. & Schult. f., Syst. Veg., ed. 15 bis [Roemer & Schultes] 7(2): 1376. 1830. Type: “In Promont. b. spei, prope muros ad urbem Cap, *Ecklon s.n.*”

*Ehrharta longiseta* Schrad., Gött. Gel. Anz. 3: 2078. 1821. Type: South Africa: Cape, Cape Good Hope; *Hesse s.n.*

*Ehrharta urvilleana* Kunth, Révis. Gramin. 1: 9. t. 6. 1829. Type: “Legit cel Dumont d’Urville in insula S. Helenae.”

*Ehrharta banksii* J.F. Gmel., Syst. Nat., ed. 13[bis] 2: 549. 1791, nom. superfl & illeg? (cited Smith pl. ic. ined. p. 9)

**Type.** “Ad Prom. Bonae Spei legit Masson. Herb. Banks. Herb Lin. Ex eadem regione relata adest nescio quo lecta botanico.”

**Distribution. **Reported for India. Apparently naturalized, but state localities not reported. Native to Africa.

**Habit.** Annual.

**155. **Ehrharta stipoides** Labill., Nov. Holl. Pl. 1: 91, t. 118. 1805 **

*Microlaena stipoides* (Labill.) R. Br., Prodr. Fl. Nov. Holland. 210. 1810

**Type.** “Habitat in capite Van-Diemen.” Australia: Tasmania, Capite van-Dieman; *Labillardière s.n.* (FI).

**Distribution.** Tamil Nadu [Sri Lanka]. Apparently naturalized. Likely native to Australia.

**Habit.** Perennial.

### Tribe Oryzeae Dumort. (1824)

Plants annual or perennial. Leaves with a membranous ligule, with or without a pseudopetiole. Spikelets with the sterile flowers reduced to lemmas or lacking entirely. *Glumes reduced or absent. Midrib with two or more vascular bundles, with bundles placed ad- and abaxially, and often with air spaces*.

The distinctive midrib anatomy that is here interpreted as synapomorphic for the tribe is lost in *Luziola* and *Hygroryza* (Kellogg 2015b; Tateoka 1963). All members of the tribe grow in sites that are moist or regularly flooded, and a few are truly aquatic (Kellogg 2015b).

***Hygroryza* Nees, Edinburgh New Philos. J. 15: 380. 1833**

**Type. ***Hygroryza aristata* (Retz.) Nees (*Pharus aristatus *Retz.).

**156. **Hygroryza aristata** (Retz.) Nees, Edinburgh New Philos. J. 15: 380. 1833. **

*Pharus aristatus* Retz., Observ. Bot. (Retzius) 5: 23. 1788 (“1789”). Type: “Habitat in Indiae aquosis.”

*Leersia aristata* (Retz.) Roxb., Fl. Ind. 2: 207. 1832.

*Zizania aristata* (Retz.) Kunth, Révis. Gramin. 1: 8. 1829

*Potamochloa retzii* Griff., J. Asiat. Soc. Bengal 5: 571. t. 24. 1836, nom. illeg. superfl.?

*Zizania retzii* Spreng., Syst. Veg. (ed. 16) [Sprengel] 2: 136. 1824 (“1825”), nom. superfl. & illeg. for *Pharus aristatus* Retz.

**Distribution.** Andhra Pradesh, Arunachal Pradesh, Assam, Bihar, Goa, Gujarat, Jammu and Kashmir, Karnataka, Kerala, Madhya Pradesh, Maharashtra, Manipur, Meghalaya, Nagaland, Odisha, Rajasthan, Tamil Nadu, Tripura, Uttar Pradesh, West Bengal [Bangladesh, China, Myanmar, Nepal, Pakistan, Sri Lanka].

**Habit.** Aquatic perennial.

**Leersia Sw., Prodr. (Swartz) 1, 21. 1788, nom. cons.**

**Type. ***Leersia oryzoides* (L.) Sw. (*Phalaris oryzoides* L.) (*typ. cons.*)

**157. **Leersia hackelii** Keng, Sinensia 11: 412. 1940 **

*Leersia oryzoides* (L.) Sw. var. *japonica* Hack., Bull. Herb. Boissier 7: 645. 1899, non *Leersiajaponica* Makino, 1892. Type: Coll. Matsum: Tokyo

*Leersia oryzoides* sensu Hook. f., Fl. Brit. India (J.D. Hooker). 7(21): 94. 1896, non (L.) Sw., 1788

**Distribution.** Jammu and Kashmir [China].

**Habit.** Perennial.

**158. **Leersia hexandra** Sw., Prodr. (Swartz) 21. 1788 **

*Asprella hexandra* (Sw.) Roem. & Schult., Syst. Veg., ed. 15 bis [Roemer & Schultes] 2: 267. 1817

*Leersia australis* R. Br., Prodr. Fl. Nov. Holland. 1: 210. 1810. Type: “Designat vicinitatem Coloniae apud Portum Jackson, inclusis ripis aestuarii Hunter’s River vel Coal River nuncupati”; *Brown s.n. *(BM). Littora Novae Hollandiae intra tropicum; *R. Brown s.n. *

*Asprella australis* (R. Br.) Roem. & Schult., Syst. Veg., ed. 15 bis [Roemer & Schultes] 2: 267. 1817

*Leersia abyssinica* Hochst. ex A. Rich., Tent. Fl. Abyss. 2: 356. 1850–1851. Type: “Crescit in locis paludosis planitiei montanae Chiré, mense Octobre, Schimp. no. 1823.” Ethiopia: Locis Paludosis in Planitie Montana, Provincia Schire; October 10, 1840; *W. Schimper 1823*

*Leersia capensis* C. Muell, Bot. Zeitung (Berlin) 14(20): 345. 1856. Type: South Africa “Prom. bonae spei, Uitenhagen”; 1847; *Alexander s.n. *(K)

*Leersia griffithiana* C. Muell, Bot. Zeitung (Berlin) 14(20): 345. 1856. Type: Bengal; *Griffith s.n.* (B?).

*Leersia luzonensis* J. Presl in C. Presl, Reliq. Haenk. 1(4–5): 207. 1830. Type: “Hab. in insula Luzonia.” Philippines: Luzonia; *Haenke s.n.*

*Leersia mauritanica* Salzm. ex Trin., Mém. Acad. Imp. Sci. Saint-Pétersbourg, Sér. 6, Sci. Math. Seconde Pt. Sci. Nat. 5, 3(4): 174. 1840. Type: Egypt: Tingit; *Sieber s.n.* (M, Isotype).

*Leersia mexicana* Kunth, Nov. Gen. Sp. [H.B.K.] 1: 195 (ed. qto.). 1816. Type: “Crescit in convsalle Mexicana inter Chalco et Xochimilco, alt. 1168 hexap.” Mexico: Chalco, Xochimilco; *Humboldt & Bonpland s.n. *(P).

*Asprella mexicana* (Kunth) Roem. & Schult., Syst. Veg., ed. 15 bis [Roemer & Schultes] 2: 267. 1817

*Leersia parviflora* Desv., Opusc. Sci. Phys. Nat. 61. 1831. Type: “Crescit in Aegyptia.”

*Leersia triniana* Siebold ex Trin., Mém. Acad. Imp. Sci. Saint-Pétersbourg, Sér. 6, Sci. Math., Seconde Pt. Sci. Nat. 5: 174. 1840, nom. invalid? (vide Tropicos)

*Oryza hexandra* (Sw.) Döll, Fl. Bras. (Martius) 2(2): 10–11. 1871

*Pharus ciliatus* Retz., Observ. Bot. (Retzius) 5: 23. 1788 (“1789”). Type: “Ad margines stagnorum lectum dedit honor Koenig.”

**Type.** Jamaica; *Swartz s.n.* (S).

**Distribution.** Andaman and Nicobar Islands, Andhra Pradesh, Assam, Bihar, Chhattisgarh, Himachal Pradesh, Jharkhand, Karnataka, Kerala, Madhya Pradesh, Maharashtra, Manipur, Meghalaya, Nagaland, Odisha, Rajasthan, Tamil Nadu, Tripura, Uttar Pradesh, West Bengal [Bangladesh, Bhutan, China, Myanmar, Nepal, Sri Lanka].

**Habit.** Perennial.

**159. **Leersia oryzoides** (L.) Sw., Prodr. (Swartz) 21. 1788 **

*Phalaris oryzoides* L., Sp. Pl. 1: 55. 1753. Type: “Habitat in Virginiae paludibus nemorosis.” Lectotype: *Herb. Linn. No. 78.10 *(LINN). LT designated by Hitchcock in Contr. U. S. Natl. Herb. 12: 115. 1908.

*Leersia hackelii* sensu Bor, Grasses Burma, Ceylon, India and Pakistan 599. 1960, non Keng., 1940

**Distribution.** Jammu and Kashmir [China, Pakistan]. Possibly naturalized in India.

**Habit.** Perennial.

***Oryza* L., Sp. Pl. 1: 333. 1753**

**Type. ***Oryza sativa* L.

**160. **Oryza australiensis** Domin, Biblioth. Bot. 20 (Heft. 85): 333. 1915 **

**Type.** Nord Australien: Queensl.; *F.M. Bailey s.n.*

**Distribution.** India; states not reported [China]. Naturalized in both countries; native to Australia and New Zealand.

**Habit.** Perennial.

**161. **Oryza barthii** A. Chev., Bull. Mus. Natl. Hist. Nat. 16(7): 405–406. 1911 **

*Oryza breviligulata* A. Chev. & Roehr., Compt. Rend. Hebd. Séances Acad. Sci. 159: 560. 1914

**Type.***Chevalier 9615*; Chad.

**Distribution.** India. Naturalized, but state localities not reported. Native to Africa.

**Habit.** Annual.

**162. **Oryza brachyantha** A. Chev. & Roehr., Compt. Rend. Hebd. Séances Acad. Sci. 159: 561. 1914 **

**Syntypes.***Chevalier s.n*.; Sudan: western Sudan, Ségou; *Schweinfurth s.n*.; Egypt: pays des Djurs.

**Distribution.** India. Naturalized, but state localities not reported. Native to Africa.

**Habit.** Annual.

**163. **Oryza coarctata** Roxb., Fl. Ind. (Roxburgh) 2: 206. 1832 **

*Porteresia coarctata* (Roxb.) Tateoka, Bull. Natl. Sci. Mus., Tokyo 8(3): 406. 1965

*Sclerophyllum coarctatum* (Roxb.) Griff., Not. Pl. Asiat. 3: 8. 1851

*Oryza triticoides* Griff., Not. Pl. Asiat. 3: 8. t. 142. f. 1. 1851

**Type.** India: Delta of the Gangas; 1796; *Buchanan-Hamilton s.n.*

**Distribution.** Andhra Pradesh, Karnataka, Odisha.

**Habit.** Perennial.

**164. **Oryza glaberrima** Steud., Syn. Pl. Glumac. 1: 3. 1853 **

**Type.** Guinea; *Jardin s.n.* (P).

**Distribution.** Karnataka, Madhya Pradesh [China]. Naturalized in both countries; native to Africa.

**Habit.** Annual.

**Remarks. **Records for India fide Naithani (1990).

**165. **Oryza grandiglumis** (Döll) Prodhl., Bot. Arch. 1: 233. 1922 **

*Oryza sativa* L. var. *grandiglumis* Döll, Fl. Bras. (Martius) 2(2): 8. 1871. Type: Brasilia: in valle Broco lectae; *Luschnath s.n. *

**Type.** Brasilien; *Riedel 1261.*

**Distribution. **India. Naturalized, but state localities not reported. Native to South America; also naturalized in China.

**Habit.** Annual.

**166. **Oryza indandamanica** Ellis, Bull. Bot. Surv. India 27(1–4): 225. f. 1–7. 1987 (“1985”) **

**Type.** India: Andamans Island, Rutland Island; July 26, 1986; *Ellis PBL 12248A.*

**Distribution. **Andaman and Nicobar Islands.

**Habit.** Perennial.

**167. **Oryza jeyporensis** Govindasw. & K.H. Krishnam., Sci. & Cult. 24: 234. 1956, nom. invalid. **

**Distribution.** Odisha.

**Habit.** Not reported.

**Remarks.** Protologue lacks Latin description.

**168. Oryza latifolia Desv., J. Bot. Agric. 1: 77. 1813**

*Oryza sativa* L. var. *latifolia* (Desv.) Döll, Fl. Bras. (Martius) 2(2): 7. 1871

**Type.** “Habitat in Carolina (error), insulaque Portorici.” Puerto Rico; *Desvaux s.n.* (P-Juss).

**Distribution.** Assam, Karnataka, Maharashtra, Sikkim [China].

**Habit.** Perennial.

**169. **Oryza meyeriana** (Zollinger & Moritzi) Baill. ssp. **granulata** (Nees & Arn. ex G. Watt) Tateoka, Bot. Mag. (Tokyo) 75: 460. 1962 **

*Oryza granulata* Nees & Arn. ex G. Watt., Dict. Econ. Prod. India 5: 500. 1891. Syntypes: Sikkim, Assam, Burma, Bengal, Malabar, Courtallum; *Wight s.n., Griffith s.n., Simons s.n., Hooker s.n., Beddome s.n., Kurz s.n., Brandeis s.n.*

*Oryza meyeriana* (Zollinger & Moritzi) Baill. var. *granulata* (Nees & Arn. ex Watt) Duistermaat, Blumea 32: 185. 1987

*Oryza meyeriana* sensu C.E.C. Fisch., Fl. Madras 3(10): 1845. 1934, non Baill., 1894

*Oryza indandamanica* J.L. Ellis, Bull. Bot. Surv. Ind. 27: 225. 1985. Type: Andaman Islands, Rutland Is., 30 m alt., 26 Jul 1986; *PBL 12248 (Ellis)*

**Distribution.** Assam, Bihar, Karnataka, Kerala, Odisha, Sikkim, Tamil Nadu, Tripura, Uttar Pradesh, West Bengal [China, Myanmar, Sri Lanka].

**Habit.** Perennial.

**170. **Oryza minuta** J. Presl in C. Presl, Reliq. Haenk. 1(4–5): 208. 1830 **

*Oryza manilensis* Merr., Philipp. J. Sci., C. 3(4): 219. 1908. Type: Philippines: Luzon Island, Rizal Province; March, 1907; *M. Ramos BS 2194* (PNH).

*Oryza latifolia* sensu Hook. f., Fl. Brit. India (J.D. Hooker). 7(21): 92. 1896, non Desv., 1813

**Type.** Habitat in insula Luzonia.” Philippines: Luzon;* Haenke s.n.* (PR).

**Distribution.** Andhra Pradesh, Assam, Madhya Pradesh, Sikkim, Tamil Nadu [China, Myanmar, Nepal].

**Habit.** Perennial.

**171. **Oryza nivara** Sharma & Shashtry, Indian J. Genet. Pl. Breed. 25: 161. 1965 **

*Oryza sativa* L. var. *fatua* Prain, Bengal Pl. 2: 1184. 1903, p.p. Type: India: “Orissa, Sundribuns; W. Bengal, N. Bengal.”

**Distribution.** Gujarat, Maharashtra, Madhya Pradesh.

**Habit.** Annual.

**Remarks.** Records for Gujarat and Maharashtra fide Naithani (1990).

**172. **Oryza officinalis** Wall. ex G. Watt, Dict. Econ. Prod. India 5: 501. 1891 ssp. **officinalis

*Oryza latifolia* sensu Hook. f., Fl. Brit. India (J.D. Hooker). 7(21): 92. 1896, non Desv.,

*Oryza minuta* sensu Bor, Grasses Burma, Ceylon, India & Pakistan 605. 1960, p.p., non J. Presl, 1830

**Type.** Numer. List [Wallich] no. 8685 and cited many syntypes.

**Distribution.** Andhra Pradesh, Assam, Bihar, Kerala, Madhya Pradesh, Maharashtra, Manipur, Odisha, Sikkim, Tamil Nadu [Bhutan, China, Myanmar, Nepal, Sri Lanka].

**Habit.** Perennial.

**173. **Oryza malampuzhaensis** Krishnasw. & Chandras., Madras Agric. J. 45: 471. 1958 **

*Oryza officinalis* Wall. ex G. Watt ssp. *malampuzhaensis* (Krishnasw. & Chandras.) Tateoka, Bot. Mag. (Tokyo) 75: 422. 1962

**Type.** India: Madras.

**Distribution.** Andhra Pradesh, Kerala, Tamil Nadu.

**Habit.** Perennial.

**Remarks.** Record for Tamil Nadu fide Naithani (1990).

**174. **Oryza rufipogon** Griff., Not. Pl. Asiat. 3: 5. t. 144. f. 2. 1851 **

*Oryza sativa* L. var. *rufipogon* (Giff.) G. Watt, Dict. Econ. Prod. India 5: 504. 1891

*Oryza fatua* Trin. var. *longiaristata* Ridl., Fl. Malay. Penin. 5: 252. 1925 (as “longe-aristata”). Type: Malaysia: Malacca, Batu Berendam; *Burkill s.n.*

*Oryza sativa* L. fo. *spontanea* Roshev., Bull. App. Bot. & Pl. 27: 37. 1931

*Oryza sativa* L. var. *coarctata* G. Watt, Dict. Econ. Prod. India 5: 504. 1891. Type: unknown locality; *Buchanan-Hamilton s.n.*

*Oryza sativa* L. var. *bengalensis* G. Watt, Dict. Econ. Prod. India 5: 504. 1891. Type: India, Bengal

*Oryza sativa* L. var. *abuensis* G. Watt, Dict. Econ. Prod. India 5: 504–505. 1891. Type: India: Bengal and Madras

**Type.** “Eastern part of Bengal, In aquis (Jheel dictis) prope Hubbegunge et Nubbeyunge; October 1, 1835.” NT: Bangladesh; *Tim s.n.* (CAL). NT designated by Sharma and Shastry in Indian J. Genet. Pl. Breed. 25(20): 157–167. 1965.

**Distribution.** Andhra Pradesh, Arunachal Pradesh, Assam, Bihar, Chhattisgarh, Karnataka, Kerala, Madhya Pradesh, Maharashtra, Manipur, Nagaland, Odisha, Rajasthan, Tamil Nadu, Tripura, Uttar Pradesh [Bhutan, China, Myanmar, Nepal, Sri Lanka].

**Habit.** Perennial.

**175. **Oryza sativa** L., Sp. Pl. 1: 333. 1753 var. **sativa

**Type.** “Habitat forte in Aethiopia, colitur in Indiae paludosis.” Lectotype: *Herb. Linn. No. 460.1* (LINN). LT designated by Meikle in Fl. Cyprus 2: 1716. 1985.

**Distribution.** Andhra Pradesh, Assam, Bihar, Chhattisgarh, Delhi, Goa, Gujarat, Himachal Pradesh, Jharkhand, Kerala, Karnataka, Madhya Pradesh, Maharashtra, Odisha, Rajasthan, Tamil Nadu, Tripura, Uttar Pradesh, Uttarakhand, West Bengal [Bhutan, China, Myanmar, Nepal, Sri Lanka].

**Habit.** Annual.

**176. **Oryza sativa** L. var. **plena** Prain, Bengal Pl. 2: 1184. 1903 **

*Oryza plena* (Prain) N.P. Chowdhury, Indian Forester 75(11): 497. 1949

**Type.** Bangladesh: Chittagong [Cultivated double rice].

**Distribution.** Andhra Pradesh, Assam, Bihar, Kerala, Madhya Pradesh, Nagaland, Punjab, Tamil Nadu, Tripura, Uttar Pradesh, West Bengal. [Bangladesh]

**Habit.** Annual.

**Remarks.** Records for Assam, Bihar, Kerala, Madhya Pradesh, Punjab, and Uttar Pradesh fide Naithani (1990).

***Zizania* L., Sp. Pl. 2: 991. 1753**

**Lectotype. ***Zizania aquatica* L. LT designated by Nash in Ill. Fl. N. U.S. (Britton & Brown), ed. 2. 1: 168. 1913, affirmed by Hitchcock, Bull. U.S.D.A. 772: 209. 1920

**177. **Zizania latifolia** (Griseb.) Stapf, Bull. Misc. Inform. Kew 1909: 385. 1909 **

*Hydropyrum latifolium* Griseb., Fl. Ross. (Ledeb.) 4: 466. 1853. Type: “Hab. in Davuria in Lacu ad confluentiam fl. Schilka et Argun”; *Turcz. as Zizania latifolia Cat. Baikal no. 1337*.

**Distribution.** Manipur [China, Myanmar].

**Habit.** Perennial.

**Remarks.** Record for Manipur fide Naithani (1990).

### Tribe Streptogyneae C.E. Hubb. ex C.E. Calderón & Soderstr. (1980)

Plants rhizomatous or caespitose. Leaf blades pseudopetiolate, with an abaxial ligule. Spikelets with several flowers, the distal ones reduced, disarticulating between the flowers. *Rachilla internode developing into a hook*. Style branches and stigmas 2 or 3, *elongating and twisting around each other at maturity, becoming indurate*.

***Streptogyna* P. Beauv., Ess. Agrostogr. 80. 1812**

**Type.***Streptogyna crinita* P. Beauv.

**178. **Streptogyna crinita** P. Beauv., Ess. Agrostogr. 80, t. 16. f. 8. 1812 **

*Streptogyna gerontogaea* Hook. f., Handb. Fl. Ceylon 5: 301. 1900 (as “streptogyne”). Type: Sri Lanka; *Thwaites s.n. Ceylon Plant 922*

**Type.** Nigeria; 1786–1788; *Beauvois s.n. *(G).

**Distribution.** Kerala, Tamil Nadu [Sri Lanka].

**Habit.** Perennial.

**Remarks.** Record for Kerala fide Naithani (1990).

### Subfamily Pooideae Benth., 1861

Plants annual or perennial, variously caespitose, rhizomatous or stoloniferous. Culms hollow. Leaf blades without a pseudopetiole, ancestrally lacking auricles; ligules membranous. Spikelets with 1 to many flowers, disarticulating above the glumes. Lodicules 2 not vascularized. Stigmas borne on separate style branches (i.e., style branches not fused).

*Brachyelytrum* is sister to all other Pooideae, with the remainder of the species having inflorescence branches initiating in a two-ranked pattern, the spikelets laterally compressed, the embryo lacking a scutellar cleft, and the embryonic leaf margins overlapping (Grass Phylogeny Working Group 2001; Kellogg et al. 2013; Saarela et al. 2015; Schubert et al. 2019a, 2019b). The next diverging tribe is Nardeae; all members of the large clade sister to Nardeae (i.e., the remainder of the subfamily) lack bicellular microhairs anywhere on the plant (Grass Phylogeny Working Group 2001). Parallel-sided subsidiary cells are synapomorphic for *Brachypodium* plus Bromeae, Triticeae, and Poeae (Grass Phylogeny Working Group 2001). A substantial increase in genome size occurred before the crown node of the clade Bromeae+ Triticeae+ Poeae, and nearly all members of the group have a chromosome base number of x=7 (Avdulov 1931; Bennetzen and Kellogg 1997; Kellogg and Bennetzen 2004).

Taxonomic editor: Robert J. Soreng, Smithsonian Institution, Washington, DC, with additional contributions for Stipeae by Paul M. Peterson, Smithsonian Institution, for *Agrostis* and *Calamagrostis* by Beata Paszko, Polish Academy of Sciences, and for *Poa* sect. *Stenopoa* by Marina V. Olonova, Tomsk State University.

### Tribe Nardeae W. D. J. Koch, 1837

Perennial plants with leathery leaves. Lower glume absent or reduced to a narrow ridge; upper glume absent or minute. *Lodicules absent; stigma one*.

***Lygeum* Loefl. in C. Linnaeus, Gen. Pl. ed. 5. 27. 1754**

**Type.***Lygeum spartum* L.

**179. **Lygeum spartum** L., Gen. Pl. ed. 5, addend. [p.”522”]. 1754 **

**Type.** “Habitat in Hispania.” Lectotype: *Loefling 36a, Herb. Linn. No. 75.1* (LINN). LT designated by Cope in Nasir and Ali (ed.), Fl. Pakistan 143: 39. 1982.

**Distribution.** Jammu and Kashmir [Pakistan].

**Habit.** Perennial.

**Remarks.** Distribution in India fide Naithani (1990). Taxonomic editor Soreng notes that the species is native to the Mediterranean, and was cultivated historically for fiber.

### Tribe Phaenospermateae Renvoize & Clayton, 1985

Leaves with pseudopetiolate and resupinate blades, and long membranous, glabrous ligules. Spikelets 1-flowered, globose, disarticulating below the glumes. Rachilla extension absent.

Soreng et al. (2017), and taxonomic editor of subfamily Pooideae here, recognizes Phaenospermateae as distinct from Duthieeae. The two tribes are sisters in molecular phylogenies and thus would be monophyletic whether combined or kept separate. *Phaenosperma *is morphologically distinctive and the combined tribe has no morphological synapomorphies. In contrast, Kellogg (2015b) combined Phaenospermateae with Duthieeae, noting that they are sister taxa and that Phaenospermateae s.s. is monogeneric; thus recognition of Phaenospermateae offers no phylogenetic information. The decision whether to combine or separate the two tribes is a matter of ranking, for which there are no taxonomic rules.

***Phaenosperma* Munro ex Benth., J. Linn. Soc., Bot. 19: 59. 1881**

**Type.***Phaenosperma globosum* Munro ex Benth. (as “globosa”).

**180. **Phaenosperma globosum** Munro ex Benth., J. Linn. Soc., Bot. 19: 59–60. 1881 (as “globosa”) **

**Type.** “First received from the Jardin des Plantes at Paris where it had been raised from seeds brought from China by the Pere David, but it has since turned up among Shearer’s Kiu-Kaing plants.”

**Distribution.** Arunachal Pradesh, Assam, Sikkim [Bhutan, China].

**Habit.** Perennial.

**Remarks.** Records for Arunachal Pradesh and Sikkim fide Naithani (1990).

### Tribe Duthieeae Röser & Jul. Schneid., Syst. Biodivers. 9(1): 41. 2011.

Leaf blades neither pseudopetiolate, nor resupinate, ligules short and sometimes ciliolate. Spikelets 1 to several flowered, disarticulating above the glumes. Rachilla extension present or absent. Lemma apex deeply 2-lobed, lobes acuminate or setaceous.

None of these morphological characters is synapomorphic.

***Duthiea* Hack., Verh. K.K. Zool.-Bot. Ges. Wien 45: 200. 1896**

**Type. ***Duthiea bromoides* Hack.

**181. **Duthiea bromoides** Hack., Verh. K.K. Zool.-Bot. Ges. Wien 45: 200. 1896 **

**Type. **India: Kashmir, Liddar Valley, alt. 12000 ft.; *J.F. Duthie 13155, 13382.*

**Distribution.** Jammu and Kashmir [Pakistan].

**Habit.** Perennial.

***Pseudodanthonia* Bor & C.E. Hubb., Kew Bull. 1957: 425. 1958**

**Type. ***Pseudodanthonia himalaica* (Hook. f.) Bor & C.E. Hubb. (*Danthonia himalaica* Hook. f.)

**182. **Pseudodanthonia himalaica** (Hook. f.) Bor & Hubbard, Kew Bull.1957: 427. 1958 **

*Danthonia himalaica* Hook. f., Fl. Brit. India (J.D. Hooker). 7(22): 281. 1896. Type: India: Kunawar, Jaunsar, Lokardi Peak, alt. 8000–9000 ft.; *J.F. Duthie 14467 *(K).

**Distribution.** Himachal Pradesh, Uttar Pradesh.

**Habit.** Perennial.

***Sinochasea* Keng, J. Wash. Acad. Sci. 48(4): 115, f. 1. 1958**

**Type.***Sinochasea trigyna* Keng.

**183. **Sinochasea trigyna **Keng, J. Wash. Acad. Sci. 48(4): 115, f. 1. 1958 **

*Trikeraia oreophila* Cope, Kew Bull. 42(2): 350. 1987. Type: Bhutan: Jangothang, 13350 ft [4069 m], 28 Sept. 1979; *Dunbar 18*

**Distribution.** Sikkim [Bhutan, China, Nepal].

**Habit.** Perennial.

**Remarks.** Record for Bhutan fide Naithani (1990).

### Tribe Meliceae Link ex Endl., 1830

Most species perennial. *Leaf sheaths with margins fused up to the collar, the margins often joined by a hyaline membrane*. Glumes usually shorter than the florets. *Lemma margins papery above, the veins not converging at the apex*. *Lodicules thick, truncate, their margins partially or wholly fused*. Style branches 2, free to the base, *the branches persistent in the fruit*.

***Glyceria* R. Br., Prodr. Fl. Nov. Holland. 179. 1810, nom. cons.**

**Type. ***Glyceria fluitans* (L.) R. Br. (*Festuca fluitans* L.).

**184. **Glyceria fluitans** (L.) R. Br., Prodr. Fl. Nov. Holland. 1: 179. 1810 **

*Festuca fluitans* L., Sp. Pl. 1: 75. 1753. Type: “Habitat in Europae fossis & paludibus.” Lectotype: Herb. Linn. No. 92.22 (LINN). LT designated by Kit Tan in Davis (ed.), Fl. Turkey 9: 537. 1985.

**Distribution.** Meghalaya, Tamil Nadu [also in Africa, Europe, North America, South America]. Naturalized; native to Europe.

**Habit. **Perennial.

**Remarks.** Taxonomic editor Soreng notes that *Glyceria fluitans* is sometimes confused with *G. plicata* (= *G. notata* Chevall (Clayton et al. 2006 onwards)) and *G. distans*. Cope (1982) accepts *G. plicata* but this is *G. notata* (Clayton et al. 2006 onwards), and these three are often confused. Based on the discussion and synonym in the Flora of China treatment (Tian 2006), it is likely that this record refers to *G. notata*, not *G. fluitans*.

**185. **Glyceria spicata **Guss., Fl. Sicul. Syn. 2: 784. 1845 **

*Poa spicata* Biv. ex Guss., Fl. Sicul. Syn. 2: 784. 1845, nom. invalid. cited as synonym of *Glyceria spicata*, non L., 1753

**Type.** “In rivulis, stagnis, inundatis in Sicilia.”

**Distribution.** Tamil Nadu. Naturalized; native to southwestern Europe and northwestern Africa.

**Habit.** Perennial.

**186. **Glyceria tonglensis** C.B. Clarke, J. Linn. Soc., Bot. 15: 119. 1876 **

*Glyceria kashmiriensis* Kelso, Rhodora 37: 262. 1935. Type: India: Jammu and Kashmir, Liddar Valley above Palgam, 8000–9000 ft.; September 17, 1893; *J.F. Duthie 13092* (US).

**Type.** India: Himalaya, between Darjeeling and Tonglo, “Mert. Koch. Proxima.”

**Distribution.** Assam, Himachal Pradesh, Jammu and Kashmir, Manipur, Sikkim, Tamil Nadu, Uttar Pradesh [Bhutan, China, Myanmar, Nepal].

**Habit.** Perennial.

***Koordersiochloa* Merr., Philipp. J. Sci., Bot. 12: 67. 1917**

**Type.***Koordersiochloa javanica* Merr.

**187. **Koordersiochloa sanjappae** (Kabeer & V.J. Nair) Veldkamp, Reinwardtia 13(3): 302. 2012 **

*Streblochaete sanjappae* Kabeer & V.J. Nair, Bull. Bot. Surv. India 47(1–4): 137 (-138, fig). 2006 (“2005”). Type: India: Tamil Nadu, Nilgiri District, Ooty, Dodabetta, about 4000 ft.; March, 4, 2002; *K. Althaf Ahamed Kabeer 114021* (MH).

**Distribution.** Tamil Nadu.

**Habit.** Perennial

**Remarks.***Koordersiochloa* Merr. is the correct name for *Streblochaete* Hochst. ex Pilg. (Veldkamp 2012).

***Melica* L., Sp. Pl. 1: 66. 1753**

**Lectotype. ***Melica nutans *L. LT designated by Hitchcock, Bull. U.S.D.A. 772: 69. 1920.

**188. **Melica nutans** L., Sp. Pl. 1: 66. 1753 **

**Type.** “Habitat in Europae frigidioris rupibus.” Proposed Conserved Type: Herb. Linn. No. 86.2 (LINN). Proposed type designated by Hempel in Jarvis (ed.), Taxon 41: 566. 1992.

**Distribution.** Jammu and Kashmir [China, Pakistan].

**Habit.** Perennial.

**Remarks.** Taxonomic editor Soreng notes that *Melica* was recently revised (Hempel 2011).

**189. **Melica onoei** Franch. & Sav., Enum. Pl. Jap. 2: 603. 1878 **

*Melica scaberrima* (Nees) Hook. f. var. *micrantha* Hook. f., Fl. Brit. India (J.D. Hooker). 7(22): 331. 1896. Type: India: Kishtwar, alt. 6000 ft.; *C.B. Clarke s.n.*

**Type.** Japan: unde sine loci indicatione e botanico japonensi ono habuit; *Dr. Savatier s.n.*

**Distribution.** Jammu and Kashmir [China, Pakistan].

**Habit.** Perennial.

**190. **Melica persica** Kunth, Révis. Gramin. 1: 122. 351. t. 89. 1830 **

*Melica cupani* Hook. f. var. *canescens* Regel, Descr. Pl. Nov. fasc. 8: 88. 1880. Type: Turkestan: in angustiis Basmandinsk; *O. Fedtschenko s.n.* (vide Tropicos)

*Melica canescens* (Regel) Lavrenko, Fl. URSS 2: 752. 1934

*Melica cupani* Guss. var. *vestita* (Boiss.) Boiss., Fl. Orient. (Boissier) 5: 591. 1884

*Melica hohenackeri* Boiss., Diagn. Pl. Orient. ser. 1, 1(13): 54. 1854. Type: “Hab. in districtu Talusch prov. transcaucasicarum (Hochstetler).”

*Melica inaequiglumis* Boiss., Diagn. Pl. Orient. ser. 1, 1(7): 124. 1846. Type: Iran: “Hab. ad saxa prope urbem Schiraz, *Kotschy no. 315*.”

*Melica jacquemontii* Decne. in Jacquem., Voy. Inde. 4(3): 174–175. t. 175. 1844. Type: India: Kanum

*Melica kotschyi* Hochst. ex Steud., Syn. Pl. Glumac. 1: 289. 1854. Type: Kurdistan: Persia bor.; *Kotsch. Herb. alepp. no. 366*

*Melica lanata* Hochst. ex Steud., Syn. Pl. Glumac. 1: 289. 1854. Type: “In cacumine montis St. Catharinae Arab. herb. Arab. un. it 104”

*Melica pannosa* Boiss., Diagn. Pl. Orient. ser. 1, 2(13): 55. 1854. Type: “Hab. in rupibus calidis Antilibani ad Souk Wadi Barrada, legi Maio 1846.”

*Melica vestita* Boiss., Diagn. Pl. Orient. ser. 1, 1(7): 125. 1846. Type: “Hab. in rupestribus alpis Kuh-Delu Persiae australis Kotschy no. 414.”

*Melica cupani* auct. non Guss., 1832: Hook. f., Fl. Brit. India (J.D. Hooker). 7(22): 329. 1896, p.p.

**Type.** “Crescit in Persia inter Kermachan et Amadan.”

**Distribution.** Jammu and Kashmir, Uttar Pradesh [Pakistan].

**Habit.** Perennial.

**191. **Melica scaberrima** (Steud.) Hook. f., Fl. Brit. India (J.D. Hooker). 7(22): 330. 1896 **

*Festuca scaberrima* Steud., Syn. Pl. Glumac. 1: 316. 1854. Type: India; *Wight s.n.*

*Glyceria scaberrima* Nees, Syn. Pl. Glumac. 1: 287. 1854. Type: India: *Nees mpt. & Royle herb. no. 376 *

**Distribution.** Himachal Pradesh, Jammu and Kashmir, Tamil Nadu, Uttar Pradesh [China, Nepal, Pakistan].

**Habit.** Perennial.

**192. **Melica secunda** Regel, Trudy Imp. S.-Peterburgsk. Bot. Sada 7(2): 629. 1881 **

*Melica gracilis* Aitch. & Hemsl., J. Linn. Soc., Bot. 19: 192. 1882. Type: Afghanistan: Kuram District, in the Shend-Toi gorge, alt. 9000 ft.

**Type.** “In kokania inter Karakasuk et Schagimardan (O. Fedtschenko), in valle Afghanistaniae Kurrum (Aitchison no. 1257), in trajectu montium Alexander Karakia(A. Regel), in valle fluvii Almatinka majoris prope Wernoje (Kuschakewicz).”

**Distribution.** Jammu and Kashmir [China, Pakistan].

**Habit. **Perennial.

**Remarks.** Record for India fide Naithani (1990).

### Tribe Stipeae Dumort., 1824

Caespitose perennials, the leaves generally stiff and slender. Spikelets one-flowered, the rachilla not extended beyond the flower. Glumes generally longer than the pubescent lemma; lemma generally wrapped around the palea, awned from the apex, the awn straight or twisted and often geniculate. Lodicules 3.

The tribe is monophyletic and the single flowered spikelet could be synapomorphic (Grass Phylogeny Working Group 2001; Kellogg 2015b). Generic limits are the subject of much ongoing research and extensive hybridization is likely (Romaschenko et al. 2008, 2010, 2011, 2012, 2014; Soreng et al. 2017).

***Achnatherum* P. Beauv., Ess. Agrostogr. 19, 146, pl. 6, f. 7. 1812**

**Lectotype.***Agrostis calamagrostis* L. Designated by Niles & Chase, Contr. U.S. Natl. Herb. 24(6): 181 (1925).

**193. **Achnatherum brandisii** (Mez) Z.L. Wu, Acta Phytotax. Sin. 34: 154. 1996 **

*Stipa brandisii* Mez, Repert. Spec. Nov. Regni Veg. 17: 207. 1921. Type: India: [Himachal Pradesh], Kulla; *D. Brandis s.n.* (B?; IT: US-866139 (fragm. ex B).

**Distribution.** Himachal Pradesh, Jammu and Kashmir, Uttar Pradesh [China, Nepal].

**Habit.** Perennial.

**Remarks.** Distribution in India fide Naithani (1990).

**194. **Achnatherum duthiei** (Hook. f.) P.C. Kuo & S.L. Lu, Fl. Reipubl. Popularis Sin. 9(3): 322, pl. 80, f. 9–14. 1987 **

*Stipa duthiei* Hook. f., Fl. Brit. India (J.D. Hooker). 7(21): 232. 1896. Type: Western Himalaya, Garhwal, alt. 12000–13000 ft.; *Duthie 273*

**Distribution.** Jammu and Kashmir, Uttar Pradesh, Uttarakhand [Nepal].

**Habit.** Perennial.

**195. **Achnatherum jacquemontii** (Jaub. & Spach) P.C. Kuo & S.L. Lu, Fl. Reipubl. Popularis Sin. 9(3): 323. 1987 **

*Stipa jacquemontii* Jaub. & Spach, Ill. Pl. Orient. 4: 60, t. 339. 1851. Type: “Ad rupes in excelsis Emodi Cachemyriani (altitudine circiter 9000 pedum) legit Jacquemont Augusto anni 1831 (Herb. Mus. Par.)” Kashmir: arid soils in rock crevices, 6400–14000 ft.; August 1831; *Jacquemont 994 *(P).

*Lasiagrostis jacquemontii* (Jaub. & Spach) Munro ex Aitch., J. Linn. Soc., Bot. 18: 107. 1880

*Lasiagrostis jacquemontii* (Jaub. & Spach) Munro ex Boiss., Fl. Orient. [Boissier] 5: 506. 1884, nom. iso., non (Jaub. & Spach) Munro ex Aitch., 1880

**Distribution.** Himachal Pradesh, Jammu and Kashmir, Uttar Pradesh, Uttarakhand [China, Pakistan].

**Habit.** Perennial.

***Neotrinia* (Tzvelev) M. Nobis, P.D. Gudkova & A. Nowak, Turczaninowia 22(1): 40. 2019.**

**Type.***Neotrinia splendens* (Trin.) M. Nobis, P.D. Gudkova & A. Nowak.

**196. **Neotrinia splendens** (Trin.) M. Nobis, P.D. Gudkova & A. Nowak, Turczaninowia 22(1): 40. 2019. **

*Stipa splendens* Trin., Neue Entdeck. Pflanzenk. 2: 54. 1821. Type: “Habitat in transbaicalensibus Sibirae.”

*Stipa splendens* Trin. var. *gracilis* Bor, Grasses Burma, Ceylon, India and Pakistan 647. 1960. Type: Baltistan, Karakoram; *C.B. Clarke 30097*

*Achnatherum splendens* (Trin.) Ohwi, J. Jap. Bot. 17: 404. 1941

*Lasiagrostis splendens* (Trin.) Kunth, Révis. Gramin. 1: 58. 1829

*Stipa altaica* Trin. ex Ledeb., Fl. Altaic. 1: 80. 1829. Type: “In arenosis et sterilibus subsalsis frequens (L.M.), in montosis apricis ad fl. Ursul. (B).”

*Stipa schlagintweitii* Mez, Repert. Spec. Nov. Regni Veg. 17: 208. 1921. Type: China: Xizang, Balti province “Tibet”; *H. von Schlagintweit 5787*

**Distribution.** Jammu and Kashmir [China, Pakistan].

**Habit. **Perennial.

***Orthoraphium* Nees, Proc. Linn. Soc. London 1: 94. 1841.**

**Type.***Orthoraphium roylei* Nees.

**197. **Orthoraphium roylei** Nees, Proc. Linn. Soc. London 1: 94. 1841. **

*Stipa roylei* (Nees) Mez, Repert. Spec. Nov. Regni Veg. 17: 207. 1921

*Stipa orthoraphium* Steud., Syn. Pl. Glumac. 1: 131. 1854, nom. superf. & illegit.

**Type.** Not given.

**Distribution.** Assam, Himachal Pradesh, Nagaland, Sikkim, Uttar Pradesh, Uttarakhand [China].

**Habit.** Perennial.

***Piptatherum* P. Beauv., Ess. Agrostogr. 17, 173. 1812**

**Lectotype.***P. caerulescens* (Desf.) P. Beauv. (*Milium caerulescens* Desf.) (vide Hitchcock, U. S. Dept. Agric. Bull. 772: 156. 1920).

**198. **Piptatherum aequiglume** (Duthie ex Hook. f.) Munro ex Roshev., Bot. Mater. Gerb. Bot. Inst. Komarova Akad. Nauk S.S.S.R. 14: 113. 1951 **

*Oryzopsis aequiglumis* Duthie ex Hook. f., Fl. Brit. India (J.D. Hooker). 7(22): 234. 1896. Type: Temperate Himalaya: from Kashmir to Sikkim, alt. 6000–10000 ft.; *Duthie s.n.*

*Piptatherum aequiglume* (Duthie ex Hook. f.) Munro ex Roshev. var. *fasciculatum* (Hack.) Freitag, Notes Roy. Bot. Gard. Edinburg 33(3): 379. 1975

*Oryzopsis fasciculata* Hack., Oesterr. Bot. Z. 52: 10. 1902. Lectotype: Pakistan: Kashmir, Astor District, in valle Kamri prope Kalapani, alt. 9000 ft.; August 25, 1892; *Duthie 12644* (W). (vide Notes Roy. Bot. Gard. Edinburg 33(3): 379. 1975)

*Piptatherum fasciculatum* (Hack.) Roshev., Bot. Mater. Gerb. Bot. Inst. Komarova Akad. Nauk S.S.S.R. 14: 114. 1951

**Distribution.** Jammu and Kashmir, Rajasthan, Sikkim [Pakistan].

**Habit.** Perennial.

**199. **Piptatherum gracile** Mez, Repert. Spec. Nov. Regni Veg. 17: 211. 1921 **

*Oryzopsis gracilis* (Mez) Pilg., Notizbl. Bot. Gart. Berlin-Dahlem 14: 347. 1939

*Oryzopsis brachyclada* Pilg., Notizbl. Bot. Gart. Berlin-Dahlem 14: 345. 1939. Type: Kashmir, Nanga-Parbat-Gebiet, Oberes Rakhiot-Tal, Moränen des Ganalo-Gletschers, nach Suden exponierte Hochgras-Matten, 12800 ft.; July 9, 1937; *C. Troll 7774*

*Oryzopsis lateralis* Stapf, Fl. Brit. India (J.D. Hooker). 7(22): 234. 1896, p.p. excluding basionym.

**Type.** China: West Tibet; *Thomson s.n.* (B).

**Distribution.** Jammu and Kashmir [China, Nepal, Pakistan].

**Habit.** Perennial.

**200. **Piptatherum hilariae** Pazij, Notul. Syst. Herb. Inst. Bot. Acad. Sci. Uzbeckistanicae 10: 20. 1948 **

*Oryzopsis humilis* Bor, Kew Bull. 1951: 445. 1952. Type: India: Jaunsar, Rocks north of Deoban, alt. 8000 ft.; June 21, 1897; *J.F. Duthie 19850a* (K).

*Piptatherum humile* (Bor) S. Kumar & Raizada, Indian J. Forest. 9(2): 177. 1986

*Oryzopsis wendelboi* Bor, Nytt Mag. Bot. 1: 16. 1952. Type: Chitral, Barum Gol, Shokor Shal, alt. 11200 ft.; July 14, 1950; *Per Wendelbo s.n. *(K).

**Type.** Asia Centre Pamir Alai.

**Distribution.** Himachal Pradesh, Jammu and Kashmir, Uttar Pradesh, Uttarakhand [China, Pakistan].

**Habit. **Perennial.

**Remarks.** Records for Jammu and Kashmir and Uttar Pradesh fide Naithani (1990). Taxonomic editor Soreng suggests accepting this name, which is older than any of Bor’s taxa listed here in synonymy.

**201. **Piptatherum laterale** (Munro ex Regel) Roshev., Bot. Mater. Gerb. Bot. Inst. Komarova Akad. Nauk S.S.S.R. 14: 117. 1951 **

*Milium laterale* Munro ex Regel, Trudy Imp. S.-Petersburgsk. Bot. Sada 7(2): 645. 1881. Type: “In Afghanistania. Piptatherum laterale Muroe.”

**Distribution.** Jammu and Kashmir [Nepal, Pakistan].

**Habit.** Perennial.

**202. **Piptatherum munroi** (Stapf) Mez, Repert. Spec. Nov. Regni Veg. 17: 212. 1921 **

*Oryzopsis munroi* Stapf, Fl. Brit. India (J.D. Hooker). 7(22): 234. 1896. Type: Western Himalaya: Kashmir to Garhwal, alt. 7000–11000 ft.; *J.D. Hooker & T. Thomson s.n.*

*Oryzopsis stewartiana* Bor, Kew Bull. 1953: 272. 1953. Type: India: Pulga, Parbatti Valley, 6400 ft.; July 22, 1935; *Mohinder Nath 988* (K).

**Distribution.** Himachal Pradesh, Jammu and Kashmir, Uttarakhand [Bhutan, China, Nepal, Pakistan].

**Habit.** Perennial.

**203. **Piptatherum vicarium** (Grig.) Roshev., Bot. Mater. Gerb. Bot. Inst. Komarova Akad. Nauk S.S.S.R. 14: 105. 1951 **

*Oryzopsis vicaria* Grig, Trudy Tadzhikistansk. Bazy 8: 574. 1938. Type: Central deciduous woodland and open vegetation, 1600 ft.-9000 ft.; *Tuturin & Besedin 81* (LE).

*Oryzopsis microcarpa* Pilg., Notizbl. Bot. Gart. Berlin-Dahlem 14: 346. 1939. Type: Kashmir, Nanga-Parbat-Gebiet, Astor Tal, Daschkin, im Artemisien Gesträuch zwischen Büschen und Steinen, 7500 ft.; May 30, 1937; *C. Troll 7272*

**Distribution.** Jammu and Kashmir [Pakistan].

**Habit.** Perennial.

**Remarks.** Record for India fide Naithani (1990).

***Ptilagrostis* Griseb., Fl. Ross. [Ledebour] 4: 447. 1852**

**Type.***Ptilagrostis mongholica* (Turcz.) Griseb. (*Stipa mongholica* Turcz.).

**204. **Ptilagrostis concinna** (Hook. f.) Roshev., Fl. URSS 2: 75. 1934 **

*Stipa concinna* Hook. f., Fl. Brit. India (J.D. Hooker). 7(21): 230. 1896. Type: Sikkim Himalaya, in the Tibetan region, alt. 14000–16000 ft.; *J.D. Hooker s.n.*

**Distribution.** Himachal Pradesh, Jammu and Kashmir, Sikkim [China, Nepal, Pakistan].

**Habit.** Perennial.

**205. **Ptilagrostis dichotoma** Keng ex Tzvelev, Rast. Tsentr. Azii 4: 43. 1968 **

**Type.** China: Gansu/Qinghai border; *Y.C. Wu 478*.

**Distribution.** India; states not recorded. [Bhutan, China, Nepal].

**Habit.** Perennial.

**206. **Ptilagrostis mongholica** (Turcz. ex Trin.) Griseb., Fl. Ross. (Ledeb.) 4: 447. 1852 **

*Stipa mongholica* Turcz. ex Trin., Bull. Sci. Acad. Imp. Sci. Saint-Pétersbourg 1: 67–68. 1836. Type: “Habitat in Sibiria baikalensi in pratis humidis ad torrentem Dschiginai in oxam influentem et ad fl. Tschikoi”; *Turcz s.n.*

*Achnatherum mongholicum* (Turcz. ex Trin.) Ohwi, J. Jap. Bot. 17: 403. 1941

*Lasiagrostis mongholica* (Turcz. ex Trin.) Trin. & Rupr., Mém. Acad. Imp. Sci. Saint-Pétersbourg, Sér. 6, Sci. Math., Seconde Pt. Sci. Nat. 5: 87. 1842

*Stipa tibetica* Mez, Repert. Spec. Nov. Regni Veg. 17: 207. 1921. Type: China: Xizang, Lasiang, Mougholica, Iriu, 14,000 ft. “West Tibet”; *J.D. Hooker & Thomson s.n.*

**Distribution.** Himachal Pradesh, Jammu and Kashmir, Sikkim, Uttar Pradesh [China, Pakistan].

**Habit.** Perennial.

***Stipa* L., Sp. Pl. 1: 78. 1753**

**Lectotype. ***Stipa pennata* L. LT designated by Nash in N.L. Britton et A. Brown, Ill. Fl. N.U.S. ed. 2. 1: 176. 1913; affirmed by Hitchcock, Bull. U.S.D.A. 772: 159. 1920.

**207. **Stipa arabica** Trin. & Rupr., Sp. Gram. Stipac. 77. 1842 **

*Stipa arabica* Trin. & Rupr. var. *szowitsiana* Trin., Mém. Acad. Imp. Sci. Saint-Pétersbourg, Sér. 6, Sci. Math., Seconde Pt. Sci. Nat. 5: 77. 1842. Type: “Persia borealis: in salsas arenosis inter Khoi et Seidkozi prov. Aderbeischan rarius; Junio (Szovitis). Transcaucin collibus aridis lapidosis prope Tatuni tractus Suwant (Hohenacker).”

*Stipa szowitsiana* (Trin.) Griseb., Fl. Ross. (Ledeb.) 4: 450. 1853 (as “szovitsiana”)

**Type.** “Inter lapides ad radices montis Sinai; 15 Majo. (Schimper Un. Itin n. 107).” Israel: Mount Sinai; *G.H.W. Schimper 107*

**Distribution.** India; states not recorded (Northwest Himalaya) [China, Pakistan].

**Habit.** Perennial.

**208. **Stipa breviflora** Griseb., Nachr. Ges. Wiss. Gottingen, Math.-Phys. Kl. 82. 1868 **

*Stipa aliciae* Kanitz, Növényt. Gyujtesek Eredm. Grof Szechenyi Bela Keletazsiai Utjabol 61. t. 7. f. 4. 1891. Type: China: “Hab Prov. Kansu n. 62. Ku-lang-szhien; June 24, 1879.”

**Type.** China: Western Xizang, Tnari Khorsum.

**Distribution.** Jammu and Kashmir, Ladakh [China, Nepal].

**Habit. **Perennial.

**Remarks. **Record for Ladakh fide Naithani (1990).

**209. **Stipa bungeana** Trin. ex Bunge, Enum. Pl. Chin. Bor. 70. 1833 **

**Type.** China: “Hebei, ad radices montium Zui-wey-schan et ad vias prope Ssi-jui-ssi”; *Bunge s.n.*

**Distribution.** India; states not recorded (Eastern Himalaya) [China].

**Habit. **Perennial.

**210. **Stipa capillacea** Keng, Sunyatsenia 6(2): 100–102, pl. 15. 1941 **

**Type.** China: Sichuan: open grass land in rear of Shaowusi Agricultural Station, Taining Xian; *K.L. Chu 7449*

**Distribution.** Arunachal Pradesh, Jammu and Kashmir, Uttarakhand [Bhutan, China, Nepal].

**Habit.** Perennial.

**211. **Stipa capillata** L., Sp. Pl., ed. 2. 1: 116. 1762 **

**Type.** “Habitat in Germania, Gallia.” Lectotype: Herb. Burser I: 127 (UPS). LT designated by Freitag in Notes Roy. Bot. Gard. Edinburgh 42: 453. 1985.

**Distribution.** Himachal Pradesh, Jammu and Kashmir [China, Pakistan].

**Habit.** Perennial.

**212. **Stipa caragana** Trin., Mém. Acad. Imp. Sci. Saint-Pétersbourg, Sér. 6, Sci. Math., Seconde Pt. Sci. Nat. 1: 74. 1830 **

*Lasiagrostis caragana* (Trin.) Trin. & Rupr., Mém. Acad. Imp. Sci. Saint-Pétersbourg, Sér. 6, Sci. Math., Seconde Pt. Sci. Nat. 7: 90. 1843

*Oryzopsis pallescens* Vestberg, Trudy Bot. Sada Imp. Yur’evsk. Univ. 5: 147. 1904. Type: Given in Russian language

**Type.** “V. ssp. e littore oriental. maris Caspici.” Russia: Caspian region; *Eichwald s.n.* (LE).

**Distribution.** India; states not recorded [China, Pakistan].

**Habit.** Perennial.

**213. **Stipa caucasica** Schmalh., Ber. Deutsch. Bot. Ges. 10: 293. 1892 ssp. **caucasica

*Stipa orientalis* Trin var. *grandiflora* Rupr., Mém. Acad. Imp. Sci. St.-Pétersbourg, Sér. 7 14(4): 35. 1869. Type: not given

*Stipa bella* Drob., Repert. Spec. Nov. Regni Veg. 21: 37. 1925, nom. later hom. illeg., non Phil, 1870

**Syntypes. **“Caucasus septentrionalis: Kislowodsk, 24 Julio 1886 (Akinefijew). Daghestania: Czir-Jurt 11 Majo et Tem ir-Chan Schura 6 Majo 1891 (Lipski).” Russia: Caucasus, Kislowodsk; July 24, 1886; *Lipsky s.n.*

**Distribution.** Jammu and Kashmir, Ladakh [China].

**Habit. **Perennial.

**Remarks.** Record for Ladakh fide Naithani (1990).

**214. **Stipa caucasica** Schmalh. ssp. **glareosa** (Smirnow) Tzvelev, Novosti Sist. Nizsh. Rast. 11: 20. 1974 **

*Stipa glareosa* Smirnow, Repert. Spec. Nov. Regni Veg. 26: 266. 1929. Type: “Hab in deserto Gobi et in Altaj locis petrosis V. glareosis.”

**Distribution.** Jammu and Kashmir [also in Mongolia].

**Habit.** Perennial.

**Remarks.** Record for India fide Naithani (1990).

**215. **Stipa chitralensis** Bor, Kew Bull. 1954: 500. 1954 **

**Type.** India Orientalis, Chitral, Guger, alt. 3000; May 18, 1895; *Surgeon Lt. Harris 16800* (K).

**Distribution.** Jammu and Kashmir [Pakistan].

**Habit.** Perennial.

**216. **Stipa consanguinea** Trin. & Rupr., Mém. Acad. Imp. Sci. Saint-Pétersbourg, Sér. 6, Sci. Math., Seconde Pt. Sci. Nat. 5: 78. 1842 **

*Stipa koelzii* R.R. Stewart, Brittonia 5(4): 441. 1945. Type: India: Kashmir, Ladakh, Gya; August 13–14, 1933; *W. Koelz 6432* (US-1607603).

**Type.** “Habitus totus Stipae orientalis, sed pubes adpressa aristarum Stipae holosericeae.” Russia: Siberi, Altai, near Tschuga river; 1832; *Bunge s.n.*

**Distribution.** Himachal Pradesh, Jammu and Kashmir, Ladakh, Sikkim [Bhutan, China, Nepal, Pakistan].

**Habit.** Perennial.

**Remarks. **Records for Himachal Pradesh, Ladakh, and Sikkim fide Naithani (1990).

**217. **Stipa himalaica** Roshev., Bot. Mater. Gerb. Glavn. Bot. Sada RSFSR 5(1): 11. 1924 **

*Stipa pennata* sensu Hook. f., Fl. Brit. India (J.D. Hooker). 7(21): 230. 1896, non L., 1753

**Type.** China: Tibet Province Ladakh; July 1–15, 1856; *Herbarium Schlagintweit 3 gen No Catalogue 1337.*

**Distribution.** Himachal Pradesh, Jammu and Kashmir [China, Pakistan].

**Habit.** Perennial.

**Remarks.** Records for India fide Naithani (1990).

**218. **Stipa orientalis** Trin. ex Ledeb., Fl. Altaic. 1: 83. 1829 **

**Type.** “In rupium fissures ad flum. Tscharysch (L.B.) et n montibus Kurtschum et Arkaul.”

**Distribution.** Jammu and Kashmir, Himachal Pradesh, Uttar Pradesh [China].

**Habit.** Perennial.

**219. **Stipa pennata** L. ssp. **kirghisorum** (Smirnow) Freitag, Notes Roy. Bot. Gard. Edinburgh 42: 438. f. 19. 1985 **

*Stipa kirghisorum* Smirnow, Repert. Spec. Nov. Regni Veg. 21: 232. 1925. Type: “Habitat in montosis et stepposis Turkestaniae et in Mongolia occidentali.”

**Distribution.** Himachal Pradesh, Jammu and Kashmir [China, Pakistan].

**Habit.** Perennial.

**Remarks.** Records for India fide Naithani (1990).

**220. **Stipa purpurea** Griseb., Nachr. Ges. Wiss. Gottingen, Math.-Phys. Kl. 82. 1868 **

*Ptilagrostis purpurea* (Griseb.) Roshev., Fl. URSS 2: 76. 1934

*Lasiagrostis tremula* Rupr., Mém. Acad. Imp. Sci. St.-Pétersbourg, Sér. 7. 14(4): 35. 1869. Type: Not given

*Stipa semenowii* Krassn., Bot. Zap. 2: 22. 1889. Type: “In valle fluminis Sary-Jassy in montibus Thian-Schan non procul ab alpe Chan-tengri et in trajectu Turguen-Aksu non rara.”

**Type.** China: Western Xizang, Tnari Khorsum, 15300–18500 ft.; *Schlagintweit 7116* (LE).

**Distribution.** Jammu and Kashmir [China, Pakistan].

**Habit.** Perennial.

**Remarks. **Taxonomic editors Soreng and Peterson note that this species should continue to be classified in *Stipa*, although it has copies of the ribosomal internal transcribed spacer (ITS) from three genera (Romaschenko et al. 2008). The dominant copy is from *Stipa* as is the chloroplast (Lu et al. 2016).

**221. **Stipa regeliana** Hack., Sitzungsber. Kaiserl. Akad. Wiss., Math.-Naturwiss. Cl., Abt. 1 89: 130. 1884 **

**Type.** Kyrgyzstan: Tien Shan, Issikul Musart pass, alt. 7000–8000 ft.; August, 1877; *A. Regel s.n.* (W).

**Distribution.** Jammu and Kashmir, Ladakh [China].

**Habit.** Perennial.

**Remarks.** Records for India fide Naithani (1990).

**222. **Stipa roborowskyi** Roshev., Bot. Mater. Gerb. Glavn. Bot. Sada RSFSR 1(6): 1. 1920 **

*Stipa basi-plumosa* Munro ex Hook. f. var. *longe-aristata* Munro ex Hook. f., Fl. Brit. India (J.D. Hooker). 7(21): 229. 1896. Type: China: Western Tibet; *Thomson s.n.*

*Stipa purpurea* sensu Bor, Grasses Burma, Ceylon, India and Pakistan 645. 1960, non Griseb., 1868

**Type.** “In montium Kuen Lun jugo Russky, alt. 9700–12200 ft.; July 3, 1890; *Roborowsky s.n.*

**Distribution.** Jammu and Kashmir, Sikkim [China, Pakistan].

**Habit. **Perennial.

**223. **Stipa sibirica** (L.) Lam., Tabl. Encycl. 1: 158. 1791 **

*Avena sibirica* L., Sp. Pl. 1: 79. 1753. Type: “Habitat in Sibiria.” Lectotype: Amman 27, Herb. Linn. No. 95.1 (LINN). LT designated by Scholz in Cafferty et al. (ed.), Taxon 49: 248. 2000.

*Achnatherum sibiricum* (L.) Keng., ex Tzvelev, Probl. Ekol. Geobot. Bot. Geogr. Florist. 140. 1977.

*Stipa confusa* Litw., Izv. Akad. Nauk SSSR, Ser. 7: 53. 1928. Type: Russia: “In Sibiriae prov. Tobolsk, Altai, Semipalatinsk (distr. Ustkamenogorsk), Jenisej (prov. Krasnojarsk. et Minusinsk), Irkutsk, Transbajcalia, Jakutsk (distr. Jakutsk).”

**Distribution.** Himachal Pradesh, Jammu and Kashmir, Uttar Pradesh [China, Malaysia, Myanmar, Pakistan].

**Habit.** Perennial.

**224. **Stipa subsessiliflora** (Rupr.) Roshev., Izv. Imp. Bot. Sada Petra Velikago 14(Suppl. 2): 50. 1915 **

*Lasiagrostis subsessiliflora* Rupr., Mém. Acad. Imp. Sci. St.-Pétersbourg, Sér. 7. 14(4): 35. 1869. Type: Kyrgyzstan: Tian Shan, Toyandyund Sunktu-Thal, 15400–16000 ft.; July 30, 1867; *F. Osten-Sacken s.n.* (LE).

*Stipa basi-plumosa* Munro ex Hook. f., Fl. Brit. India (J.D. Hooker). 7(21): 229. 1896. Type: China: Western Tibet, Nubra, and the Lanak Pass, alt. 15000–17000 ft.; *Thomson s.n.*

**Distribution.** Jammu and Kashmir [China, Pakistan].

**Habit. **Perennial.

**225. **Stipa turkestanica** Hack., Trudy Imp. S.-Peterburgsk. Bot. Sada 26: 59. 1910 ssp. **turkestanica

**Type.** Turkestania: Schugnan, Dschidak in valle fl. Badamdara; July 27, 1904; *B.A. Fedtschenko s.n.*

**Distribution.** Himachal Pradesh, Jammu and Kashmir [also in Asia].

**Habit.** Perennial.

**Remarks.** Records for India fide Naithani (1990).

**226. **Stipa turkestanica** Hack. ssp. **trichoides** (P.A. Smirn.) Tzvelev, Novosti Sist. Vyssh. Rast. 11: 17. 1974 **

*Stipa trichoides* P.A. Smirn., Repert. Spec. Nov. Regni Veg. 21: 233. 1925. Syntypes: Turkmenistan: Ashabad Province, Mt. Ludsha, 6500 ft.; *Litvinov 2222* (LE). Turcomania: Fergana, Alaicum, Langar; *B. Fedtschenko s.n.*

**Distribution.** Jammu and Kashmir [Afghanistan, Pakistan].

**Habit.** Perennial.

**Remarks.** Record for India fide Naithani (1990).

***Stipellula* Röser & H.R. Hamasha, Schlechtendalia 24: 91. 2012**

**227. **Stipellula capensis** (Thunb.) Röser & H.R. Hamasha, Schlechtendalia 24: 92. 2012 **

*Stipa capensis* Thunb., Prodr. Pl. Cap. 19. 1794. Type: South Africa; *Thunberg s.n.* (UPS).

*Stipa tortilis* Desf., Fl. Atlant. 1: 99. t. 31. f. 1. 1798. Type: “Habitat in arvis.”

**Distribution.** Punjab [Pakistan].

**Habit.** Annual.

***Trikeraia* Bor, Kew Bull. 1954: 555. 1955**

**Type. ***Trikeraia hookeri* (Stapf) Bor (*Stipa hookeri* Stapf).

**228. **Trikeraia hookeri** (Stapf) Bor, Kew Bull. 1954: 555. 1955 var. **hookeri

*Stipa hookeri* Stapf, J. Linn. Soc., Bot. 30: 120. 1894. Type: China: Tibet, Probably in the vicinity of Lhasa; July, September, 1891; *Thorold 124* (K).

*Achnatherum hookeri* (Stapf) Keng, Clav. Gen. Sp. Gram. Prim. Sin. 213. 1957

*Timouria aurita* Hitchc., J. Wash. Acad. Sci. 23: 134–136. 1933. Type: India: Western Himalaya, Kashmir, Rupshu, Kugzil, alt. 13700; July 16, 1931; *Walter Koelz 2328*

**Distribution.** Jammu and Kashmir [China, Pakistan].

**Habit.** Perennial.

**229. **Trikeraia hookeri** (Stapf) Bor var. **ramosa** Bor, Kew Bull. 1954(4): 557. 1955 **

*Stipa hookeri* Stapf var.* ramosa* (Bor) S. Karthikeyan et al., Fl. Ind. ser. 4, 1 (Monocotyledon): VI. 1989

**Type.** China: Tibet, Nye, Tsangpo Valley, 12500 ft.; September 2, 1935; *F. Kingdon Ward 12295* (BM).

**Distribution.** India; states not recorded [China].

**Habit.** Perennial.

**Remarks.** Taxonomic editors Soreng and Peterson question whether this species truly occurs in India.

### Tribe Brachypodieae Harz, 1880

Plants annual or perennial. *Inflorescence unbranched, the spikelets on short pedicels*. Spikelets laterally compressed, generally with more than 5 flowers. Glumes shorter than the flowers. Ovary apex pubescent.

***Brachypodium* P. Beauv., Ess. Agrostogr. 100, 155. 1812**

**Lectotype. ***Brachypodium pinnatum* (L.) P. Beauv. (*Bromus pinnatus* L.). LT designated by Niles and Chase, Contr. U.S. Natl. Herb. 24: 196. 1925.

**230. **Brachypodium distachyon** (L.) P. Beauv., Ess. Agrostogr. 156. 1812 (as “distachyos”) **

*Bromus distachyos* L., Fl. Palaest. 13. 1756. Type: “Habitat in H. [= Hasselquist]…Palaestina.” Lectotype: Hasselquist, Herb. Linn. No. 93.48 (LINN). LT designated by Schippmann & Jarvis in Taxon 37: 158. f. 1. 1988.

*Festuca distachyos* (L.) Roth, Catal. Bot. 1: 11. 1797

*Trachynia distachya* (L.) Link, Hort. Berol. [Link] 1: 43. 1827

*Zerna distachyos* Panz. ex B.D. Jacks., Index Kew. 1(4): 1249. 1895

**Distribution.** Jammu and Kashmir, Punjab, Tamil Nadu, Uttar Pradesh [China, Pakistan].

**Habit.** Annual.

**Remarks.** Record for Jammu and Kashmir fide Naithani (1990). Taxonomic editor Soreng notes that *B. distachyon* has been split into three species (Catalán et al. 2012; Lopez-Alvarez et al. 2012). It is unclear which name, or if all 3, would be applicable to material from India. Also, there may be older names for the new species, but this will require checking the types of heterotypic synonyms.

**231. **Brachypodium sylvaticum** (Huds.) P. Beauv., Ess. Agrostogr. 156. 1812 **

*Festuca sylvatica* Huds., Fl. Angl. (Hudson) 38. 1762. Type: “Habitat in sylvis et sepibus frequens.”

*Brachypodium sylvaticum* (Huds.) P. Beauv. var. *khasianum* Hook. f., Fl. Brit. India (J.D. Hooker). 7(22): 363. 1886. Type: India: Khasia Hills

*Brachypodium sylvaticum* (Huds.) P. Beauv. var. *longe-aristatum* Hook. f., Fl. Brit. India (J.D. Hooker). 7(22): 363. 1886. Type: North West Himalaya: Kumaon and Dalhousie, alt. 6000–9000 ft.

*Brachypodium sylvaticum* (Huds.) P. Beauv. var. *pseudo-distachyon* Hook. f., Fl. Brit. India (J.D. Hooker). 7(22): 363. 1886. Type: India: Kashmir and Kumaon

*Brachypodium wattii* C.B. Clarke, J. Linn. Soc., Bot. 25: 90. t. 40. 1889. Type: India: Kohima, alt. 7500 ft.; *C.B. Clarke s.n.* Jakpho, alt. 7500 ft.; *C.B. Clarke s.n.*

*Brachypodium sylvaticum* (Huds.) P. Beauv. var. *luzoniense* (Hack.) H. Hara Bot. Mag. (Tokyo) 52: 228. 1938.

*Brachypodium sylvaticum* (Huds.) P. Beauv. ssp. *luzoniense* Hack., Philipp. J. Sci. 1(Suppl. 4): 269. 1906. Type: Philippines: Luzon Province, Benguet, Pauai to Baguio; November 9, 1905; *Merrill 4698*

**Distribution.** Arunachal Pradesh, Himachal Pradesh, Jammu and Kashmir (C82), Meghalaya, Nagaland, Sikkim, Tamil Nadu, Uttar Pradesh, Uttarakhand [Bhutan, China, Nepal, Pakistan].

**Habit. **Perennial.

### Tribe Bromeae Dumort., 1824

Plants annual or perennial, caespitose or rhizomatous. *Leaf sheaths with fused margins*. Spikelets with 3 or more flowers, disarticulating above the glumes. Lemma entire or bidentate, if awned then the awn forming from the sinus or on the abaxial side just below the apex. *Style branches on the abaxial side of the ovary, the ovary enlarged and lobed above the point of insertion of the style branches*.

This tribe included *Littledalea* in the treatment of Kellogg (2015b), but *Littledalea* is now placed in its own tribe (Soreng et al. 2015) so that *Bromus* is the sole member of Bromeae.

***Bromus* L, Sp. Pl. 1: 76. 1753, nom. cons.**

**Synonyms.***Anisantha* K. Koch, *Bromopsis* (Dumort.) Fourr., *Ceratochloa* P. Beauv.

**Type. ***Bromus secalinus* L. (typ. cons.)

**232. **Bromus arvensis** L., Sp. Pl. 1: 77. 1753 **

**Type.** “Habitat in Europa ad versuras agrorum.” Lectotype: Herb. Linn. No. 93.21 (LINN). LT designated by Smith in Notes Roy. Bot. Gard. Edinburgh 42: 499. 1985.

**Distribution.** Himachal Pradesh, Jammu and Kashmir, Tamil Nadu [China].

**Habit.** Annual.

**Remarks.** Taxonomic editor Soreng cites Cope (1982), saying that this species is not confirmed for Pakistan. “Bor (1960) keys it out but has no distribution for it other than temperate Europe and Asia.”

**233. **Bromus berteroanus** Colla, Herb. Pedem. 6: 68. 1836 **

*Bromus barobalianus* G. Singh, For. Fl. Srinagar 129. 1976

**Distribution.** Jammu and Kashmir. Native to South America.

**Habit.** Annual.

**Remarks.** Records from India fide Naithani (1990).

**234. **Bromus catharticus** Vahl, Symb. Bot. (Vahl) 2: 22. 1791 **

*Bromus unioloides* Kunth, Nov. Gen. Sp. [H.B.K.] 1: 151 (ed. qto.). 1816. Type: “Crescit locis alsis regni Quitensi prope Chillo, Conocoto et Sangolqui, alt. 1340 hexap.” Ecuador: Pichincha, Chillo, Conocoto & Sangolqui, alt. 1340 ft.; *Humboldt & Bonpland 2286* (P).

*Bromus willdenowii* Kunth, Revis. Gramin. 1: 134. 1829. Type:* Willdenow Herb. 2103 *(B).

*Ceratochloa cathartica* (Vahl) Herter, Revista Sudamer. Bot. 6(5–6): 144. 1940

**Type.** “Habitat in Lima.” Lectotype: Peru: Lima; sd; *P. Commerson s.n.* (P-JU, US-865523). LT designated by Pinto-Escobar, Caldasia 11(54): 9–16. 1976.

**Distribution.** Delhi, Himachal Pradesh, Jammu and Kashmir, Meghalaya, Sikkim, Tamil Nadu, West Bengal [China, Nepal]. Naturalized; native to southern South America.

**Habit.** Perennial.

**Remarks.** Records for Himachal Pradesh, Jammu and Kashmir, Meghalaya, Sikkim, and West Bengal fide Naithani (1990).

**235. **Bromus confinis** Nees, Syn. Pl. Glumac. (Steudel) 1: 320. 1854 **

*Bromus inermis* Leyss. var. *confinis *(Nees) Stapf, Fl. Brit. India (J.D. Hooker). 7(22): 358. 1896

**Type.** Nepal; *Royle Herb. no. 325.*

**Distribution.** Himachal Pradesh (B60), Sikkim [China, Pakistan].

**Habit.** Perennial.

**236. **Bromus danthoniae** Trin. ex C.A. Mey., Verz. Pfl. Casp. Meer. (C.A. von Meyer) 24. 1831 **

*Triniusa danthoniae* (Trin. ex C.A. Mey.) Steud., Syn. Pl. Glumac. 1: 328. 1854 (as “danthonia”)

*Bromus macrostachys* Desf. var. *triaristatus* Hack., Flora 62: 155. 1879, in obs. Type: Teheran; *Kotschy s.n.* ?

**Type.** Caucasus: Azerbaijan, Talush; June 22, 1830; *C.A. Meyer s.n. *(LE). “In locis lapidosis aridis montium Talusch prope pagum Swant (alt. 670 Hexap.)”.

**Distribution.** Jammu and Kashmir [China, Pakistan].

**Habit.** Annual.

**237. **Bromus diandrus** Roth, Bot. Abh. Beobacht. 44. 1787 **

*Bromus gussonii* Parl., Pl. Nov. 66. 1842 (as “gussoni”). Type: “Habitat in collibus, ad sepes, et in sylvaticis Siciliae, insulae Capri, aliisque Italiae locis. Obtinui ex Corsica ab amico botanico Le Maire.” Sicilia; *Gussone s.n.* (FI).

**Neotype.** Gr. Bromoides, locustis maximus, lanuginosum, Italicum. Hist. Nat. 261, no. 444, (OXF (Scheuzer Herb.)). NT designated by Sales, Edinb. J. Bot. 50(1): 8–9. 1993.

**Distribution.** Tamil Nadu [also in the Middle East, Africa, Europe, North America, South America]. Naturalized.

**Habit.** Annual.

**238. **Bromus gracillimus** Bunge, Mém. Acad. Imp. Sci. St.-Pétersbourg Divers Savans 7: 527. 1852 **

*Nevskiella gracillima* (Bunge) Krecz. & Vved., Trudy Sredne-Aziatsk. Gosud. Univ., Ser. 8b, Bot.17: 22. 1934

*Bromus crinitus* Boiss. & Hohen. ex Boiss., Diagn. Pl. Orient. ser. 1, 2(13): 64. 1854. Type: “Hab. In monte Elbrus prope pagum Passgala.”

*Deschampsia aralensis* Regel, Bull. Soc. Imp. Naturalistes Moscou 41(2): 300. 1868. Type: “Steppe des Aralses im Gebiete des Syr-Darja, Ende Mai fruchttragende Exemplare; *Borazozow 774.*”

**Type.** “Hab. in der Wuste Karakum bei Kuk-Kabak Zwischen Tschi (Lasiagrostis splendes) 19 Mai 1842 (Deflor. et fr. Maturo).”

**Distribution.** Himachal Pradesh, Jammu and Kashmir [China, Pakistan].

**Habit.** Annual.

**239. **Bromus himalaicus** Stapf, Fl. Brit. India (J.D. Hooker). 7(22): 358. 1896 **

*Zerna himalaica* (Stapf) Henrard, Blumea 4(3): 499. 1941

**Type.** Temperate Himalaya; East Nepal, Sikkim and North Bhutan, alt. 8000–12000 ft.; *J.D. Hooker s.n.*

**Distribution.** Assam, Sikkim, West Bengal [Bhutan, China, Nepal].

**Habit.** Perennial.

**240. **Bromus hordeaceus** L., Sp. Pl. 1: 77. 1753 **

*Bromus mollis* L., Sp. Pl. ed. 2, 1: 112. 1762. Type: “Habitat in Europae australioris siccis.” Lectotype: *Herb. Linn. No. 93.6* (LINN). LT designated by Smith in Cafferty et al. (Ed.), Taxon 49: 248. 2000.

*Bromus hordeaceus* L. ssp. *mollis* (L.) Hyl., Uppsala Univ. Arsskr. 7: 84. 1945

*Serrafalcus mollis* (L.) Parl., Rar. Pl. Sicilia. 2: 11. 1840

**Lectotype.** “Gramen Avenaceum pratense gluma breviore squamosa et villosa” in Morison, Pl. Hist. Univ., 3: 213. s. 8. t. 7. f. 18. 1699. LT designated by Smith in Cafferty et al. (Ed.), Taxon 49: 248. 2000. Epitype: Herb. Linn. No. 93.7 (LINN). Epitype designated by Smith, P.M. in Cafferty et al. (Ed.), Taxon 49: 248. 2000.

**Distribution. **Himachal Pradesh, Jammu and Kashmir [China, Pakistan].

**Habit.** Annual.

**241. **Bromus inermis** Leyss., Fl. Halens. 16. 1761 **

*Schedonorus inermis* (Leyss.) P. Beauv., Ess. Agrostogr. 177. 181

*Zerna inermis* (Leyss.) Lindm., Sv. Fanerogamfl. 101. 1918

*Bromus inermis* Leyss. var. *villosus* (Mert. & W.D. J. Koch) Beck., Fl. Nieder-Oesterreich. 1: 106. 1890

*Festuca inermis* (Leyss.) Lam. & DC. var. *villosus* Mert. & W.D.J. Koch, Deutschl. Fl. (Mertens & W.D.J. Koch), ed. 3, 1: 675. 1823

*Bromus latifolius* Kar. & Kir., Bull. Soc. Imp. Naturalistes Moscou 14: 865. 1841. Type: “Hab. in umbrosis montium Aktschavly ad fl. Karakol.”

**Type.** “In pratis fertilibus succulentis Pomariis in den Pulverweiden im Amstgarten ad Belberg Crollwitz et alibi frequens.”

**Distribution.** Jammu and Kashmir, Uttar Pradesh. Widespread in Eurasia.

**Habit.** Perennial.

**242. **Bromus japonicus** Houtt., Natuurlijke Historie [tweede deel (second part)] 2(13): 315, t. 91, f. 4. Aanwyzing Pl. [2]. 1782 **

*Bromus abolinii* Drobow., Repert. Spec. Nov. Regni Veg. 21: 40. 1925. Syntypes: Turkey: Semi-reczie Province, Almaata District; *Abolin 909,1502, 1578, 1634, 1655, 1721, 1838, 1864, 1866, 1869*

*Bromus cyri* Trin., Verz. Pfl. Casp. Meer. (C.A. von Meyer) 24. 1831. Type: [Caucasus: Azerbaijan: Kura river]; *C.A. Meyer s.n. *(LE).

*Bromus patulus* Mert. & W.D.J. Koch in Röhl., Deutschl. Fl. (Mertens & W.D.J. Koch), ed. 3, 1: 685. 1823

*Bromus patulus* Mert. & W.D.J. Koch var. *microstachya* Stapf, Fl. Brit. India (J.D. Hooker). 7(22): 361. 1896. Type: Northwest India; *Falconer s.n.*

*Serrafalcus patulus* (Mert. & W.D.J. Koch) Parl., Fl. Ital. (Parlatore) 1: 394. 1848

**Type.** Japan; *Thunburg s.n.*

**Distribution.** Jammu and Kashmir, Uttarakhand [China].

**Habit.** Annual.

**243. **Bromus lanceolatus** Roth, Catalect. Bot. 1: 18. 1797 **

**Type.** Europe: Semina benignitati debeo amici aestimatiss cel. Roemeri; *Roemer*.

**Distribution.** Himachal Pradesh, Jammu and Kashmir [Pakistan].

**Habit.** Annual.

**Remarks.** Record for Jammu and Kashmir fide Naithani (1990).

**244. **Bromus moeszii** Penzes, Magyar Bot. Lapok 33: 24, pl. 10. 1934 **

*Bromus sericeus* Drobow, Repert. Spec. Nov. Regni Veg. 21: 39. 1925, non Ten. 1811. Type: Turkey: Syrdarja Province, Taschkent District, Kaplanbek; 1921; *Abolin 7496*

*Bromus tectorum* L. ssp. *lucidus* F. Sales, Fl. & Veg. Mundi 9: 32. 1991, nom. nov. for Bromus sericeus Drobow, non. Ten. 1823

**Type.** Iran: auf Aker und Strassen Graben bei [Daulatabad] Dolitabad; *Fichler 18*.

**Distribution.** Himachal Pradesh, Jammu and Kashmir [China, Pakistan].

**Habit.** Annual.

**245. **Bromus oxyodon** Schrenk, Bull. Sci. Acad. Imp. Sci. Saint-Pétersbourg. 10: 355. 1842 **

*Bromus nototropus* Rupr., Mem. Acad. Imp. Sci. Saint Petersbourg Ser. 7, 14(4): 73. 1869, in adnot. Type: Not given [Mittleren Tian-Schan]

*Bromus lanceolatus* Roth. ssp. *oxyodon* (Schrenk) Tzvelev, Spisok Rastenij Gerbarija Flory SSSR 18: 19. 1970

**Type.** China: Xinjiang: Dzhungaria, desert grasslands, 500–2600 m; *Schrenk s.n.* (LE).

**Distribution.** Jammu and Kashmir [China, Pakistan].

**Habit.** Annual.

**246. **Bromus pectinatus** Thunb., Prodr. Pl. Cap. 1: 22. 1794 **

*Bromus gedrosianus* Penz., Bot. Kozlem. 33: 111. f. 39 (a-d). 48. 1936. Type: Biluchistan: Gedrosia, Killa abdulla; April 10, 1888; *J.F. Duthie 8738*

*Bromus japonicus* Thunb. var. *falconeri* (Stapf) R.R. Stewart, Brittonia 5(4): 413. 1945

*Bromus patulus* Mert. & W.D.J. Koch var. *falconeri* Stapf, Fl. Brit. India (J.D. Hooker). 7(22): 361. 1896. Type: North West India; *Falconer s.n.*

**Type.** South Africa: Cape of Good Hope; *Thunberg 2522* (UPS).

**Distribution.** Jammu and Kashmir, Punjab, Uttar Pradesh [Bhutan, China, Nepal, Pakistan].

**Habit.** Annual.

**247. **Bromus porphyranthos** Cope, Fl. Pakistan 143: 574. 1982 **

*Bromus himalaicus* Stapf var. *grandis* Stapf, Fl. Brit. India (J.D. Hooker). 7(22): 359. 1896. Type: India: Kumaon, Sikkim, alt. 11000–12000 ft.; *R. Strachey & J.E. Winterbottom s.n.*

*Bromusgrandis* (Stapf) Melderis in H. Hara et al., Enum. Fl. Pl. Nepal 1: 125. 1978, non (Shear) Hitchc., 1912

**Distribution.** Sikkim, Uttarakhand [Bhutan, China, Nepal, Pakistan].

**Habit.** Perennial.

**248. **Bromus ramosus** Huds., Fl. Angl. (Hudson) 40. 1762 **

*Zerna ramosa* (Huds.) Lindm., Sv. Fanerogamfl. 101. 1918

*Bromus asper* Murray, Prodr. Strip. Gott. 42. 1770. Type: “Hall. Hist. fl. Helun. 1503. Habitau proxime ad Bromum giganteum accedit, cui vero spiculae quadriflorae. Cl Willich Nordheimi inuenit ego in sylua plessensi.”

**Type.** “Habitat in Sylvis et sepibus frequens.”

**Distribution.** Himachal Pradesh, Jammu and Kashmir, Kerala, Meghalaya, Nagaland, Sikkim, Tamil Nadu, Uttarakhand [China, Pakistan].

**Habit.** Perennial.

**249. **Bromus scoparius** L., Cent. Pl. I 6. 1755 **

*Bromus confertus* M. Bieb., Fl. Taur.-Caucas. 1: 71. 1808. Type: “Habitat in Iberia.” *D. Steuen s.n.*

*Bromus ovatus* Gaertn., Novi Comment. Acad. Sci. Imp. Petrop. 14: 537. t. 19/1. 1770

*Serrafalcus scoparius* (L.) Parl., Rar. Pl. Sicilia. 2: 19. 1840

**Type.** “Habitat in Hispania.” Lectotype: Loefling 81, Herb. Linn. No. 93.32 (LINN). LT designated by Smith in Notes Roy. Bot. Gard. Edinburgh 42: 499. 1985.

**Distribution.** Jammu and Kashmir [China].

**Habit.** Annual.

**250. **Bromus staintonii** Melderis in H. Hara et al., Enum. Fl. Pl. Nepal 1: 125. 1978 var. **staintonii

**Type.** Nepal: Central Nepal, Tukucha, Kali Gandaki, open grassy slopes, 10200 ft.; August 21, 1954; *Stainton, Sykes & Williams 7366* (BM).

**Distribution.** Arunachal Pradesh, Himachal Pradesh, Jammu and Kashmir, Sikkim, Uttar Pradesh [Bhutan, China, Nepal].

**Habit.** Perennial.

**Remarks.** All records for India fide Naithani (1990).

**251. **Bromus tectorum** L., Sp. Pl. 1: 77. 1753 **

*Schedonorus tectorum* (L.) Fr., Bot. Not. 1843(9): 131. 1843

*Zerna tectorum* (L.) Panz. ex B.D. Jacks., Index. Kew. 1(4): 1249. 1895

*Anisantha pontica* C. Koch, Linnaea 21: 394. 1848. Type: Turkey: Coruh, Ispir, alt. 4000 ft.; *Koch s.n.*

*Bromus abortiflorus* St.-Amans, Fl. Agen. 44. 1821. Type: “Fl. E. I. Le. Bord. Des champs sablonneux, a St. Laurent, vis-à-vis le Port-Saint-Marie, a Villeneuve-sur-lot, C. Dans Les Landes, a La Lague, pres Xaintrailles.”

*Bromus setaceus* Buckley, Proc. Acad. Nat. Sci. Philadelphia 14: 98–99. 1862. Lectotype: USA: Northern Texas; *Buckley s.n.* (US-865474 (fragm.)). LT designated by Hitchcock in Man. Grasses U.S. 817. 1935

**Type.** “Habitat in Europae collibus siccis et tectis terrestribus.” Lectotype: *Herb. Linn. No. 93.23* (LINN). LT designated by Smith in Notes Roy. Bot. Gard. Edinburgh 42: 500. 1985.

**Distribution.** Assam, Jammu and Kashmir, Uttar Pradesh [China, Pakistan].

**Habit.** Annual.

### Tribe Triticeae Dumort., 1824

Plants annual or perennial, caespitose or rhizomatous. Leaf sheaths often with prominent auricles. *Inflorescence unbranched*, the spikelets borne crosswise to the rachis. Spikelets 1 to 5 per node, laterally compressed, disarticulating above the glumes. *Lodicules ciliate*. *Endosperm with simple starch grains*.

Generic limits in this tribe are problematical (Barkworth 2007; Stebbins 1956; Stebbins and Snyder 1956; Stebbins, Jr. and Walters 1949). Extensive hybridization and polyploidy mean that the evolutionary history is not tree-like and hence cannot be converted to a hierarchical classification (Kellogg et al. 1996); therefore, all efforts to create monophyletic genera are somewhat arbitrary.

***Aegilops* L., Sp. Pl. 2: 1050. 1753, nom. cons.**

**Type. ***Aegilops triuncialis* L. (type cons.).

**252. **Aegilops tauschii** Coss., Notes Pl. Crit. 2: 69. 1850 (“1849”) **

*Patropyrum tauschii* (Coss.) A. Love, Biol. Zeutralbl. 101(2): 206. 1982

*Triticum aegilops* sensu Hook. f., Fl. Brit. India (J.D. Hooker). 7(22): 367. 1896, non P. Beauv. ex Roem. & Schult., 1817

**Type.** “Habitat in lberia (Buxbaum. Loc. cit, Wilhems in Herb. Gay). In Tauria (Tauch, Loc. cit.). In Graminosis et aridis prope Elisabethpol Georgia Caucasicae (Hohenacker, un. it. 1834).” Lectotype: Ill., pl. 50. f. 1. in Buxbaum, Pl. minus cognit. Centuria 1. 1728. (Illustration). LT designated by van Slageren in Wegen. Agric. Univ. Pap. 94–7: 328. 1994.

**Distribution.** Kashmir [China, Pakistan].

**Habit.** Annual.

**Remarks.** See van Slageren (1994)

***Elymus* L., Sp. Pl. 1: 83. 1753**

**Lectotype. ***Elymus sibiricus* L. LT designated by Hitchcock, Bull. U.S.D.A. 772: 93. 1920.

**Remarks. **Taxonomic editor Soreng cites the major generic synonyms of *Elymus* as *Elytrigia* Desv., *Hystrix* Moench, and *Roegneria* K. Koch. In contrast, he notes, *Anthosachne*, *Pseudoroegneria*, *Stenostachys*, and *Thinopyrum* are generally maintained as separate genera, although Australasian members of *Anthosachne* and *Stenostachys* are not part of those genera as currently understood. See Barkworth et al. (2009).

**253. **Elymus borianus** (Melderis) Cope in E. Nasir & S.I. Ali, Fl. Pakistan 143: 617. 1982 **

*Agropyron borianum* Melderis in Bor, Grasses Burma, Ceylon, India and Pakistan 690. 1960. Type: Swat: Kalam, 7000 ft.; August 24, 1955; *A. Rahmann 229* (K).

**Distribution.** Jammu and Kashmir [Pakistan].

**Habit.** Perennial.

**254. **Elymus burchan-buddae** (Nevski) Tzvelev, Rast. Tsentr. Azii 4: 220. 1968 **

*Agropyron burchan-buddae* Nevski, Izv. Bot. Sada Akad. Nauk SSSR 30: 514. 1932

**Distribution.** India; states not recorded [China, Nepal].

**Habit.** Not recorded.

**255. **Elymus cacuminis **B. Rong Lu & B. Salomon, Nordic J. Bot. 13: 355, f. 1. 1993 **

*Roegneria cacuminis* (B. Rong Lu & B. Salomon) L.B. Cai, Acta Phytotax. Sin. 35(2): 160. 1997

**Distribution.** India; states not recorded [China, Nepal].

**Habit.** Not recorded.

**256. **Elymus caninus** (L.) L., Fl. Suec. ed. 2, (Linnaeus) 39. 1755 **

*Agropyron caninum* (L.) P. Beauv., Ess. Agrostogr. 146. 1812

*Triticum caninum* L., Sp. Pl. 1: 86–87. 1753. Type: “Habitat in Europae sepibus.” Lectotype: *Herb. Linn. No. 100.10* (LINN). LT designated by Cope & van Slageren in Cafferty et al. (ed.), Taxon 49: 258. 2000.

**Distribution.** Jammu and Kashmir. Widespread in Eurasia.

**Habit. **Perennial.

**Remarks.** Record for India fide Naithani (1990).

**257. **Elymus ciliaris** (Trin. ex Bunge) Tzvelev, Novosti Sist. Vyssh. Rast. 9: 61. 1972 **

*Agropyron ciliare* (Trin. ex Bunge) Franch., Nouv. Arch. Mus. Hist. Nat. Ser. 2, 7: 151. 1884

*Triticum ciliare* Trin. ex Bunge, Enum. Pl. Chin. Bor 72. 1833. Type: “Habitat in pratensibus prope Kan-tai.”

**Distribution.** Arunachal Pradesh, Bihar [China].

**Habit.** Perennial.

**Remarks.** Record for Arunachal Pradesh fide Naithani (1990).

**258. **Elymus dahuricus** Turcz. ex Griseb., Fl. Ross. [Ledebour] 4: 331. 1852 **

*Clinelymus dahuricus* (Trucz. ex Griseb.) Nevski, Izv. Bot. Sada Akad. Nauk S.S.S.R 30: 645. 1932

*Elymus dahuricus* Turcz. ex Griseb. var. *micranthus* Melderis in Bor, Grasses Burma, Ceylon, India and Pakistan 697. 1960. Type: Pakistan: Bumbrait; July 22, 1956; *J.H. Norris 25184* (K).

**Type.** “Hab. In sibiria altaica pr. Krasnojarsk”; *Turcz s.n.* “inque Davuria”; *Turcz s.n.*

**Distribution.** Himachal Pradesh, Jammu and Kashmir, Punjab [Bhutan, China, Nepal, Pakistan].

**Habit.** Perennial.

**259. **Elymus dentatus** (Hook. f.) Tzvelev, Novosti Sist. Vyssh. Rast. 10: 21. 1973 ssp. **dentatus

*Agropyron dentatum* Hook. f., Fl. Brit. India (J.D. Hooker). 7(22): 370. 1896. Type: India: Kashmir, alt. 9000–12000 ft.; *Jacquemont s.n.*, *Thomson s.n.* Western Tibet; Karakoram, alt. 14000 ft.; *C.B. Clarke s.n.*

**Distribution.** Jammu and Kashmir [Pakistan].

**Habit.** Perennial.

**260. **Elymus dentatus** (Hook. f.) Tzvelev ssp. **elatus** (Hook. f.) Á. Löve, Feddes Repert. 95: 455. 1984 **

*Agropyron dentatum* Hook. f. var. *elatum* Hook. f., Fl. Brit. India (J.D. Hooker). 7(22): 371. 1896. Type: India: Kashmir, Dras, alt. 12000–13000 ft.; *Thomson s.n.*

*Elymus dentatus* (Hook. f.) Tzvelev var. *elatus* (Hook. f.) G. Singh, J. Econ. Tax. Bot. 8(2): 497. 1986

**Distribution.** Jammu and Kashmir [Pakistan].

**Habit.** Perennial.

**261. **Elymus dentatus** (Hook. f.) Tzvelev ssp. **kashmiricus** (Melderis) Á. Löve, Feddes Repert. 95: 455. 1984 **

*Elymus dentatus* (Hook. f.) Tzvelev var. *kashmiricus* (Melderis) Signh, J. Econ. Tax. Bot. 8: 497. 1986

*Agropyron dentatum* Hook. f. var. *kashmiricum* Melderis in Bor, Grasses Burma, Ceylon, India and Pakistan 690. 1960. Type: India: Kashmir; *W. Munro s.n.* (K).

**Distribution.** Jammu and Kashmir.

**Habit.** Perennial.

**Remarks.** Record for Jammu and Kashmir fide Naithani (1990).

**262. **Elymus dentatus** (Hook. f.) Tzvelev ssp. **scabrus** (Nevski) Á. Löve, Feddes Repert. 95: 455. 1984 **

*Agropyron dentatum* Hook. f. var. *scabrum* Nevski, Izv. Bot. Sada Akad. Nauk S.S.S.R 30: 626. 1932. Type: India: Kashmir, Gilgit, Multur Valley in the bed of stream, 9000–10000 ft.; August 5, 1892; *J.F. Duthie 12423*

**Distribution.** Jammu and Kashmir [Pakistan].

**Habit.** Perennial.

**Remarks.** Record for India fide Naithani (1990).

**263. **Elymus duthiei** (Melderis) G. Singh, Taxon 32(4): 639. 1983 **

*Agropyron duthiei* Melderis, Grasses Burma, Ceylon, India and Pakistan 662, 690. 1960. Type: Western Himalaya: near Simla, 7000–8000 ft.; August 23, 1889; *J.F. Duthie 10123 *(K).

*Elymus longearistatus* (Boiss.) Tzvelev ssp. *duthiei* (Melderis) Á. Löve, Feddes Repert. 95: 468. 1984

**Distribution.** Himachal Pradesh, Uttar Pradesh, Uttarakhand [China, Nepal].

**Habit.** Perennial.

**Remarks.** Records for Himachal Pradesh and Uttar Pradesh fide Naithani (1990).

**264. **Elymus fedtschenkoi** Tzvelev, Novosti Sist. Vyssh. Rast. 10: 21. 1973 **

*Agropyron curvatum* Nevski, Izv. Bot. Sada Akad. Nauk S.S.S.R 30: 629. 1932. Type: “Habitat in Turkestania (Prov. Semipalatinsk, Semireczie, Fergana (Alaj) Kuldsha).”

*Agropyron macrolepis* auct. non Drobow 1925: Bor, Grasses Burma, Ceylon, India and Pakistan 663. 1960

**Distribution.** Jammu and Kashmir [Nepal, Pakistan].

**Habit.** Perennial.

**Remarks.** Record for Jammu and Kashmir fide Naithani (1990).

**265. **Elymus himalayanus** (Nevski) Tzvelev, Novosti Sist. Vyssh. Rast. 9: 61. 1972 **

*Roegneria himalayana* Nevski, Trudy Sredne-Aziatsk. Gosud. Univ., Ser. 8b, Bot. 17: 68. 1934. Type: NW Himalaya: Jihri-Garhwal, Rhudughera; August 19, 1983;* J.F. Duttie 140 *(LE).

*Agropyron himalayanum* (Nevski) Melderis, Grasses Burma, Ceylon, India and Pakistan 662. 1960

**Distribution.** Jammu and Kashmir, Uttar Pradesh, Uttarakhand [China, Pakistan].

**Habit.** Perennial.

**Remarks.** Records for Jammu and Kashmir and Uttar Pradesh fide Naithani (1990).

**266. **Elymus jacquemontii** (Hook. f.) Tzvelev, Rast. Centr. Azii, Mater. Bot. Inst. Komarov 4: 221. 1968 **

*Anthosachne jacquemontii *(Hook. f.) Nevski Fl. URSS 2: 598, pl. 44, f. 20a and b 1934

*Agropyron jacquemontii* Hook. f., Fl. Brit. India (J.D. Hooker). 7(22): 369–370. 1896. Syntypes: Western Tibet, Bekar; *Jacquemont s.n*. Nubra, alt. 17000 ft.; *Thomson s.n.* Kumaon, alt. 15500 ft.; *R. Strachey & J.E. Winterbottom s.n.*

**Distribution.** Uttarakhand [China].

**Habit.** Perennial.

**267. **Elymus longe-aristatus** (Boiss.) Tzvelev ssp. **canaliculatus** (Nevski) Tzvelev, Novosti Sist. Vyssh. Rast.9: 62. 1972 **

*Agropyron canaliculatum* Nevski, Izv. Bot. Sada Akad. Nauk S.S.S.R. 30: 509. 1932. Type: Tajikistan: Darvaz, Peter the Great Range southern slope, Vereshkai glacier, alt. 10500 ft.; July 29, 1899; *V. Lipskii 2500* (LE).

*Roegneria longe-aristata* (Boiss.) Drobow var. *canaliculata* (Nevski) L.B. Cai, Acta Phytotax. Sin. 35(2): 165. 1997

*Agropyron longe-aristatum* auct. non (Boiss.) Boiss., 1884: Hook. f., Fl. Brit. India (J.D. Hooker). 7(22): 368. 1896

*Elymus *x* incertus* H. Hartmann, Candollea 39(2): 519. 1984. Type: Zanskar, Tungri Tal, Doda Rivers, alt. 12800 ft.; *H. Hartmann 2613*

**Distribution.** Himachal Pradesh, Jammu and Kashmir, Uttar Pradesh [China].

**Habit.** Perennial.

**Remarks.** Records for Himachal Pradesh and Jammu and Kashmir fide Naithani (1990). *Anthosachne* Steud. is currently a recognized genus, but restricted to Australasia*. Anthosachne longe-aristatus* (Boiss.) Nevski does not belong to that genus according to Mary Barkworth (pers. comm. Dec 2013).

**268. **Elymus mutabilis** (Drobov) Tzvelev, Pl. As. Cent. 4:217. 1968 **

*Agropyron mutabile* Drobow, Trudy Botaničeskago Muzeja Imperatorskoj Akademii Nauk 16: 88, pl. 9, f. 3–4. 1916. Type: *Drobov 315*.

**Distribution.** Jammu and Kashmir. [Pakistan].

**Habit.** Perennial.

**Remarks.** Record for Jammu and Kashmir fide Naithani (1990).

**269. **Elymus nayarii** Karthik. in S. Karthikeyan et al., Fl. Ind. ser. 4, 1 (Monocotyledon): 213. 1984. Replacement name. **

*Agropyron thomsonii* Hook. f., Fl. Brit. India (J.D. Hooker). 7(22): 370. 1896 (as “thomsoni”). Syntypes: Western Himalaya, alt. 10000–12000 ft. from Kunawur and Piti; *Jacquemont s.n., Thomson s.n.* Garhwal; *R. Strachey & J.E. Winterbottom s.n., Duthie s.n.*

*Elymus thomsonii* (Hook. f.) Melderis in H. Hara et al., Enum. Fl. Pl. Nepal 1: 132. 1978, non Hook. f., 1896

**Distribution.** Uttarakhand.

**Habit. **Perennial.

**Remarks.** Taxonomic editor Soreng notes that Clayton et al. (2006 onwards) cite this as *Agropyron thompsonii*, endemic to India. However, Löve (1984) placed it in the synonymy of *Leymus secalinus*. Evidently, more study is needed.

**270. **Elymus nutans** Griseb., Nachr. Königl. Ges. Wiss. Georg-Augusts-Univ. 72. 1868 var. **nutans

*Clinelymus nutans* (Griseb.) Nevski, Izv. Bot. Sada Akad. Nauk S.S.S.R. 30: 644. 1932

*Elymus nutans* Griseb. var. *elatior* Griseb., Nachr. Königl. Ges. Wiss. Georg-Augusts-Univ. 73. 1868. Type: Himalaya occidentalis: Garhwal, 10000–10600 ft.; *Thomson s.n.*

*Elymus nutans* Griseb. var. *condensatus* Griseb., Nachr. Königl. Ges. Wiss. Georg-Augusts-Univ. 73. 1868. Type: Himalaya occidentalis, Garhwal, 13400–17600 ft.; *T. Nari Khorsum*

*Elymus sibiricus* L. var. *minor* Hack. ex Hook. f., Fl. Brit. India (J.D. Hooker). 7(22): 373. 1896. Type: *Duthie no. 13745*

*Elymus nutans *Griseb. var. *albidus* Melderis in Bor, Grasses Burma, Ceylon, India & Pakistan 697. 1960. Type: West Himalaya: Kunawur, Soognam, circa agros; *V. Jacquemont 1638* (K).

**Type.** “West Himalaya”: Garhval, from Ghastoli North of Badrinath up the Sarsutti Valley do Deo Tal on South foot of Mana Pass; September 3–5, 1855; *Schlagintweit 9158* (LE). Based on Hooker’s misapplication of *E. sibiricus* Hook., non L.

**Distribution.** Himachal Pradesh, Jammu and Kashmir, Uttar Pradesh, Uttarakhand [Bhutan, China, Nepal].

**Habit.** Perennial.

**271. **Elymus repens** (L.) Gould, Madroño 9(4): 127. 1947 **

*Elytrigia repens* (L.) Nevski, Trudy Bot. Inst. Akad. Nauk S.S.S.R., Ser. 1, Fl. Sist. Vyssh. Rast. 1: 14. 1933

*Agropyron repens* (L.) P. Beauv., Ess. Agrostogr. 102. 146, 180, pl. 20. f. 2. 1812

*Triticum repens* L., Sp. Pl. 1: 86. 1753. Type: “Habitat in Europae cultis.” Lectotype: Herb. Linn. No. 104.7 (LINN). LT designated by Bowden in Canad. J. Bot. 43: 1431. 1965.

**Distribution.** Jammu and Kashmir [China, Pakistan].

**Habit.** Perennial.

**272. **Elymus russellii** (Melderis) Cope, Fl. Pakistan 143: 618. 1982 **

*Agropyron russellii* Melderis in Bor, Grasses Burma, Ceylon, India and Pakistan 665, 694. 1960. Type: Karakoram, Hispar Glacier, dry Bank, 12500 ft.; August 1, 1939; *R. Scott Russell 1388* (BM).

**Distribution.** Jammu and Kashmir [Pakistan]

**Habit.** Perennial.

**Remarks.** Taxonomic editor Soreng notes that it is unclear if this species occurs in India or if it is truly endemic only to the Pakistan portion of Kashmir (Baltistan, Gilgit, Markerum).

**273. **Elymus schrenkianus** (Fisch. & C.A. Mey.) Tzvelev, Bot. Mater. Gerb. Bot. Inst. Komarova Akad. Nauk S.S.S.R. 20: 428. 1960 **

*Agropyron schrenkianum* (Fisch. & C.A. Mey.) P. Candargy, Etude Monogr. Tribu des Hordes 1: 22, 41. 1901

*Triticum schrenkianum* Fisch. & C.A. Mey., Bull. Cl. Phys.-Math. Acad. Imp. Sci. Saint-Pétersbourg 3(68): 305. 1845. Type: “In monte Tarbagtai.”

**Distribution.** Jammu and Kashmir, Sikkim [Bhutan, China, Nepal].

**Habit.** Perennial.

**Remarks.** Record for Jammu and Kashmir fide Naithani (1990).

**274. **Elymus schugnanicus** (Nevski) Tzvelev, Nov. Sist. Vyssh. Rast. 9: 62. 1972 **

*Agropyron schugnanicum* Nevski, Izvestiya Botanicheskogo Sada Akademii Nauk SSSR 30: 512. 1932

**Distribution.** Jammu and Kashmir [Pakistan].

**Habit.** Annual.

**Remarks.** Record for Jammu and Kashmir fide Naithani (1990).

**275. **Elymus sibiricus** L., Sp. Pl. 1: 83. 1753 **

*Clinelymus sibiricus* (L.) Nevski, Izv. Bot. Sada Akad. Nauk S.S.S.R. 30: 641. 1932

*Elymus krascheninnikovii* Roshev., Izv. Bot. Sada Akad. Nauk S.S.S.R. 30: 780. 1932 (as “krascheninnikovi”). Type: Mongolia bor. Occidentalis: In valle fl. Dshargalanta (47° lat. Bor., 104–105 ° long. or.) Ad fontem fl. Uber-Dshargalanta; September 11, 1925; *H. Krascheninnikov 967/169*

*Elymus praetervisus* Steud., Syn. Pl. Glumac. 1: 348. 1854. Type: Russia: [South] Urals Mountains, Lake Ilmen; 1832; *Lessing s.n.* (LE).

**Type.** “Habitat in Sibiria.” Proposed Conserved Type: *Herb. Linn. No. 100.2* (LINN). Type designated by Bowden in Canad. J. Bot. 42: 554. 1964.

**Distribution.** Jammu and Kashmir, Sikkim [China, Nepal].

**Habit.** Perennial.

**276. **Elymus sikkimensis** (Melderis) Melderis, En. Fl. Pl. Nepal 1: 132. 1978 **

*Agropyron sikkimense* Melderis, Grasses of Burma, Ceylon, India and Pakistan (excluding Bambuseae) 694. 1960

**Distribution.** Sikkim [Nepal].

**Habit.** Perennial.

**Remarks. **Record for Sikkim fide Naithani (1990).

**277. **Elymus stewartii** (Melderis) Cope, Fl. Pakistan 143: 627. 1982 **

*Agropyron stewartii* Melderis in Bor, Grasses Burma, Ceylon, India and Pakistan 695. 1960. Type: Baltistan: near Kasurmik, Shyok watershed, 9000–10000 ft.; August 15, 1940; *R.R. Stewart & I. D. Stewart 20704* (BM).

*Pseudoroegneria stewartii* (Melderis) A. Löve, Feddes Repert. 95: 447. 1984

**Distribution.** Jammu and Kashmir [Pakistan].

**Habit.** Not recorded but likely perennial.

**Remarks.** Record for Jammu and Kashmir fide Naithani (1990). Taxonomic editor Soreng notes that *Pseudoroegneria* is usually separated from *Elymus*, but it is unclear whether *P. stewartii* is a good member of *Pseudoroegneria*.

**278. **Elymus tschimganicus** (Drobow) Tzvelev, Rasteniia Tsentral’noi Azii 4: 221. 1968 **

*Agropyron tschimganicum* Drobow, Key Fl. Tashkent 1: 40. 1923

**Distribution.** Uttar Pradesh [China].

**Habit.** Perennial.

**Remarks.** Record for India fide Naithani (1990). Taxonomic editor Soreng comments on the orthographic variations in the history of this name. In 1923 Drobow spelled it *tschimganicum*; in 1940 Drobow spelled it *czimganicum*; in Fl. Central Azii, 4: 221 (270 in English ed.) 1968, Tzvelev, comb. nov. the combination was spelled as *Elymus czimganicus*, later corrected to *tschimganicus*.

**279. **Elymus × nothus** (Melderis) G. Singh, Taxon 32(4): 639. 1983 **

*Agropyron *×* nothum* Melderis in Bor, Grasses Burma, Ceylon, India and Pakistan 693. 1960. Type: Karakoram, Makerum, Hispar Glacier, dry bank, 12,500 ft.; August 1, 1939; *R. Scott Russell 1387* (BM).

**Distribution.** Jammu and Kashmir.

**Habit.** Not recorded.

**Remarks.** Taxonomic editor Soreng states that this is the named hybrid *Elymus dentatus* × *E. fedtschenkoi*. Cope (1982) lists it among other putative hybrids but says none have been investigated beyond original characterization.

***Eremopyrum* (Ledeb.) Jaub. & Spach, Ann. Sci. Nat. Bot. ser. 3. 14: 360. 1851**

*Triticum* sect. *Eremopyrum* Ledeb., Fl. Altaica 1: 112. 1829.

**Lectotype. ***Eremopyrum orientale* (L.) Jaub. & Spach (*Secale orientale* L.). LT designated by A. Love in Biol. Zentralbl. 101: 208. 1982.

**280. **Eremopyrum bonaepartis** (Spreng.) Nevski, Trudy Bot. Inst. Akad. Nauk S.S.S.R., Ser. 1, Fl. Sist. Vyssh. Rast. 1: 18. 1933 **

*Triticumbonaepartis* Spreng., Bot. Gart. Halle erster Nachtrag [Suppl. 1]. 40. 1801. Type: “Habitat in Aegypto, Sprengel.” Africa: Egypt, alt. 4200–7000 ft. (B-W-2335.1). (vide Tropicos)

*Agropyron bonaepartis *(Spreng.) T. Durand & Schinz, Consp. Fl. Afr. [T.A. Durand & H. Schinz] 5: 936. 1894 (as “Agropyrum buonapartis”)

*Agropyron orientale* (L.) Roem. & Schult. var. *sublanuginosum* Drobow, Trudy Bot. Muz. Imp. Akad. Nauk 16: 135. 1916. Type: “Prov. Syr-Darja. Circa urb. Petro-Alexandrowsk. In arenosis (Lg. A. Zaregradsky, 1914).”

*Eremopyrum buonapartis* (Spreng.) Nevski var. *sublanuginosum* (Drobow) Melderis, Ark. Bot., n.s. 2: 215, 305. 1952

*Agropyron turkestanicum* Gand., Bull. Soc. Bot. France 60: 420. 1913. Type: Turkestan: Merw province, near Repetek; April 16, 1902; *Androssow in Herb. fl. Ross 1899* (C).

*Hordeum hirsutum* Bertol., Misc. Bot. 1: 11. 1842. Lectotype: Chesney’s Exped. Pl. Sicc. 196, 1837, “Euphr. ex oris Euphratis” (BOLO). LT designated by Frederikden, Nordic J. Bot. 11: 279. 1991.

*Eremopyrum hirsutum* (Bertol.) Nevski, Fl. URSS 2: 663. 1934

*Triticum squarrosum* Roth, Neue Beytr. Bot. 128. 1802, nom. superfl. & illeg. for *Triticum bonaepartis*

**Distribution.** India (Northwest Himalaya) [Pakistan].

**Habit.** Annual.

**Remarks.** Distribution in India not recorded.

**281. **Eremopyrum distans** (K. Koch) Nevski, Trudy Bot. Inst. Akad. Nauk S.S.S.R., Ser. 1, Fl. Sist. Vyssh. Rast. 1: 18. 1933 **

*Agropyron distans* K. Koch, Linnaea 21: 426. 1848 (as “Agropyrum”). Type: “Aus der Provinz Eriwan, c. 3000’ hoch, auf basaltisch-trachytischem Boden.” Russia: Armenia, ultra Araxen fluv. apud Amaranth; 1838; *C. Koch 636 *(LE)

*Agropyron lasianthum* Boiss., Diagn. Pl. Orient. ser. 1, 2(13): 68. 1854 (as “Agropyrum”). Type: “Hab. in Campis Caspiis (Fisch.) in Songaria (Turcz) sub nominee Tr. Orientalis acceptum.”

**Distribution.** India (Western Himalaya; states not recorded) [China, Pakistan].

**Habit.** Annual.

**282. **Eremopyrum orientale** (L.) Jaub. & Spach, Ill. Pl. Orient. 4: 26–27. t. 319. 1851 **

*Secale orientale* L., Sp. Pl. 1: 84. 1753. Type: “Habitat ad Archipelagum.” Neotype: *Herb. Tournefort No. 4939* (P-TOURN). LT designated by Frederiksen in Nordic J. Bot. 11: 277. 1991.

*Agropyrum orientale* (L.) Roem. & Schult., Syst. Veg., ed. 15 bis [Roemer & Schultes] 2: 757. 1817

*Triticum orientale* (L.) M. Bieb., Fl. Taur.-Caucas. 1: 86. 1808

**Distribution.** India (Western Himalaya; states not recorded) [China, Pakistan].

**Habit.** Annual.

***Hordeum* L., Sp. Pl. 1: 84. 1753**

**Lectotype. ***Hordeum vulgare *L. (Designated by Nash in N.L. Britton et A. Brown, Ill. Fl. N.U.S. ed. 2. 1: 286. 7 Jun 1913; affirmed by Hitchcock, Bull. U.S.D.A. 98. 1920).

**283. **Hordeum aegiceras** Royle ex Lindl., Intr. Nat. Syst. Bot., ed. 2. 1836 **

*Critho aegiceras *(Royle ex Lindl.) E. Mey., Index Seminum [Königsberg (Regimontanum)]. 5. 1848

*Hordeum vulgare *L. ssp. *aegiceras *(Royle ex Lindl.) Á. Löve, Feddes Repert. 95(7–8): 435. 1984)

*Hordeum vulgare* L. var.* aegiceras* (Royle ex Lindl.) Aitch., Cat. Pl. Punjab Sindh 171. 1869

**Type.** Kashmir: cultivated at over 3000 m.

**Distribution.** Jammu and Kashmir [China, Pakistan].

**Habit.** Annual.

**Remarks. **Taxonomic editor Soreng notes that this species is sometimes cultivated in Eastern Europe where it is accepted as *Hordeum vulgare* subsp. *aegiceras* (Nees ex Royle) Á. Löve (PlantBase 2005). Clayton et al. (2006 onwards) accept it as a species, and Flora of China (Wu et al. 2006) as a variety, *Hordeum vulgare* var. *trifurcatum* (Schltdl.) Alef.

**284. **Hordeum bogdanii** Wilensky, Trudy Glavn. Bot. Sada 40: 248. 1928 (as “bogdani”) **

*Critesion bogdanii* (Wilensky) Á. Löve, Feddes Repert. 95: 438. 1984

*Hordeum secalinum* sensu Hook. f., Fl. Brit. India (J.D. Hooker). 7(22): 372. 1896, p.p., non Schreb., 1771

**Type. **Russia: Volga region, Lake Elton, near farm Smutnev; March 27, 1917; *D. Wilensky 374a *(LE).

**Distribution.** Jammu and Kashmir [China].

**Habit.** Perennial.

**Remarks.** Record in India fide Naithani (1990).

**285. **Hordeum brevisubulatum** (Trin.) Link, Linnaea 17: 391–392. 1844 ssp. **brevisubulatum

*Hordeum secalinum* Schreb. var. *brevisubulatum* Trin., Sp. Gram. [Trinius] 1(1): t. 4. 1823. Type: “Figura ad speciment ircutense.”

*Hordeum secalinum* sensu Hook. f., Fl. Brit. India (J.D. Hooker). 7(22): 372. 1896, p.p., non Schreb., 1771

**Distribution.** India (Northwest Himalaya; states not recorded) [China, Pakistan].

**Habit.** Perennial.

**286. **Hordeum brevisubulatum** (Trin.) Link ssp. **nevskianum** (Bowden) Tzvelev, Novosti Sist. Vyssh. Rast. 8: 66. 1971 **

*Hordeum nevskianum* Bowden, Canad. J. Genet. Cytol. 7: 396. 1965

**Distribution.** Jammu and Kashmir [China, Nepal, Pakistan].

**Habit.** Perennial

**Remarks.** Records from India and Nepal fide Naithani (1990).

**287. **Hordeum brevisubulatum** (Trin.) Link ssp. **turkestanicum** (Nevski) Tzvelev, Novosti Sist. Vyssh. Rast. 8: 66. 1971 **

*Hordeum turkestanicum* Nevski, Trudy Sredne-Aziatsk. Gosud. Univ., Ser. 8b, Bot. 17: 40. 1934. Type: “Montes meridionales: Tian-schan occidentalis. Ad declivia argilloso-saxosa montis Tschimgan Majoris”; August 10, 1926; *Rajkova s.n.*

*Critesion brevisubulatum* (Trin.) Á. Löve ssp. *turkestanicum* (Nevski) Á. Löve, Feddes Repert. 95: 438. 1984

**Distribution.** Jammu and Kashmir [Nepal].

**Habit.** Perennial

**Remarks.** Records from India and Nepal fide Naithani (1990).

**288. **Hordeum lagunculiforme** Bakhteyer, Kungl. Lantbrukshogskolans Ann. 23: 309, pl. 1. 1957 **

**Distribution.** Jammu and Kashmir [China].

**Habit.** Annual.

**289. **Hordeum leporinum** Link, Linnaea 9: 133. 1834 **

*Hordeum murinum* L. var. *leporinum* (Link) K. Richt., Pl. Eur. 1: 130. 1890

*Hordeum murinum* L. ssp. *leporinum* (Link) Arcang., Comp. Fl. Ital. 805. 1882.

*Critesionmurinum* (L.) Á. Löve ssp. *leporinum *(Link) Á. Löve, Taxon 29(2–3): 350. 1980

*Hordeum pseudomurinum* Tapp. ex W.D.J. Koch., Syn. Fl. Germ. Helv., ed. 2, 955. 1845. Type: “Ad vias in Tyroli australi, (bei Schlanders im Vintschgau, Dr. Tappeiner)”; *Tappeiner s.n.*

*Hordeum ambiguum* Döll, Fl. Bras. (Martius) 2(3): 231. 1880. Type: “Habitat ad Monte video, sello d. n. 755.” Uruguay: Montevideo; *Sellow 755*

*Hordeum rubens* Willk., Oesterr. Bot. Z. 25: 109. 1875. Type: “Mallorea: in cultis, muris, ruderatis, hortis in oppido Soller. Die 6 Maji Jam fere defloratum.”

**Type.** “Frequens in Graecia.”

**Distribution.** India (Northwest Himalaya) [also in Europe].

**Habit.** Annual.

**Remarks.** Distribution in India not recorded. Taxonomic editor Soreng notes that this species may not occur in India. It is native across the Mid-east to Pakistan including Kashmir. While Cope (1982) cites it as occurring in China, it is not listed by Wu et al. (2006).

**290. **Hordeum marinum** Huds. ssp. **gussoneanum** (Parl.) Asch. & Graebn., Syn. Mitteleur. Fl. [Ascherson & Graebner]. 2: 737. 1902 **

*Hordeum gussoneanum* Parl., Fl. Palerm. 1: 246. 1845, in obs. Type: Italy: Sicily; *Gussone s.n.*

*Critesion marinum* (Huds.) Á. Löve ssp. *gussoneanum *(Parl.) Barkworth & D.R. Dewey, Amer. J. Bot. 72(5): 772. 1985

**Distribution.** Jammu and Kashmir [Pakistan]. Naturalized; native from Europe to central Asia.

**Habit.** Annual.

**Remarks.** Record in India fide Naithani (1990). Taxonomic editor Soreng notes that if this actually occurs in India, it is probably naturalized. Cope (1982) records it as occurring in Europe to central Asia, and cites one record from Pakistan.

**291. **Hordeum murinum** L., Sp. Pl. 1: 85. 1753 ssp. **murinum

*Zeocriton murinum* (L.) P. Beauv., Ess. Agrostogr. 182. 1812

*Critesion murinum* (L.) Á. Löve, Taxon 29(2–3): 350. 1980

*Triticum murale* Salisb., Prodr. Stirp. Chap. Allerton 27. 1796, nom. superfl. & illeg.

**Type. **“Habitat in Europae locis ruderatis.” Lectotype: *Herb. Clifford: 24, Hordeum 3 *(BM-000557673). LT designated by Baum & Jarvis in Taxon 34: 529. f. 1. 1985.

**Distribution.** Jammu and Kashmir [Afghanistan].

**Habit. **Annual.

**292. **Hordeum murinum** L. ssp. **glaucum** (Steud.) Tzvelev, Novosti Sist. Vyssh. Rast. 8: 67. 1971 **

*Hordeum glaucum* Steud., Syn. Pl. Glumac. 1: 352. 1854. Type: Saudi Arabia: Mons. Sinai; *Schimper 383* (P).

*Critesion glaucum* (Steud.) Á. Löve, Feddes Repert. 95: 440. 1984

*Hordeum stebbinsii *Covas, Madrono 10(1): 17–19, pl. 2. F. 8, 12[left], 13. 1949. Type: USA: California: Lake County, Middletown; May 9, 1948; *G.L. Stebbins & G. Covas 3927* (UC-754601).

**Distribution.** Jammu and Kashmir [Eurasia].

**Habit. **Annual.

**Remarks.** Record from India fide Naithani (1990).

**293. **Hordeum spontaneum** C. Koch, Linnaea 21: 430–431. 1848 **

*Hordeum ithaburense* Boiss., Diagn. Pl. Orient. Ser. 1, 2(13): 70. 1854. Type: “Hab. In declivibus siccis montis Ithaburis in latere meridionali supra Daburieh.”

*Hordeum vulgare* L. var. *spontaneum* (C. Koch) Asch. & Graebn., Syn. Mitteleur. Fl. [Ascherson & Graebner]. 2(1): 723. 1902 [cited as synonym]

**Type.** “Auf den steppenartigen Matten des schirwanschen Theiles des Kaukasus, 500–1000 ft.” Russia: Caucasus, 500–1000 ft.; *C. Koch s.n.*

**Distribution.** India (Northwest; states not recorded) [China].

**Habit.** Perennial.

**294. **Hordeum vulgare** L., Sp. Pl. 1: 84–85. 1753 **

*Hordeum distichon* L., Sp. Pl. 1: 85. 1753. Neotype: France. “Pl. de l’Ouest de la France”; June 29, 1879; *Guadeceau s.n.* (BM-000576277). LT designated by Cope in Cafferty et al. (ed.), Taxon 49: 251. 2000.

*Hordeum vulgare* L. ssp. *agriocrithon* (Åberg) Á. Löve, Feddes Repert. 95: 435. 1984

*Hordeum agriocrithon* Åberg, Ann. Agri. Coll. Sweden 6: 159. 1938. Type: China: Xizang, Taofu (Dawo), 9600 ft. (“two specimens grown from the seed collection no. 13064”); *H. Smith*.

**Lectotype.** Herb. Linn. No. 103.1 (LINN). LT designated by Bowden in Canad. J. Bot. 37: 679. 1959.

**Distribution.** Assam, Bihar, Delhi, Himachal Pradesh, Jammu and Kashmir, Jharkhand, Karnataka, Madhya Pradesh, Maharashtra, Punjab, Rajasthan, Tamil Nadu, Uttar Pradesh, Uttarakhand, West Bengal [China].

**Habit.** Annual.

**Remarks. **Taxonomic editor Soreng notes that this species is cultivated throughout semi-arid regions of Eurasia.

***Kengyilia* C. Yen & J.L. Yang, Canad. J. Bot. 68: 1897. 1990**

**Type. ***Kengyilia gobicola* C. Yen & J.L. Yang.

**295. **Kengyilia thoroldiana** (Oliv.) J.L. Yang, C. Yen & B.R. Baum, Hereditas (Lund) 116(1–2): 27. 1992 **

*Agropyron thoroldianum* Oliv., Hooker’s Icon. Pl. 23(3): t. 2262. 1893

*Elymus thoroldianus* (Oliv.) G. Singh, Taxon 32(4): 640. 1983

*Roegneria thoroldiana* (Oliv.) Keng, Acta Univ. Nankin. Sci. Nat. 1963(3): 79. 1963

**Distribution.** Sikkim [China].

**Habit.** Perennial.

***Leymus* Hochst., Flora 31: 118. 1848**

**Type. ***Leymus arenarius* (L.) Hochst. (*Elymus arenarius* L.).

**296. **Leymus secalinus** (Georgi) Tzvelev, 4: 209. 1968 **

*Triticum secalinum* Georgi, Bemerk. Reise Russ. Reich 1: 198. 1775. Type: Russia, 2600–5000m.

*Elymus dasystachys* Trin., Fl. Altaic. (Ledebour). 1: 120. 1829

*Elymus thomsonii* Hook. f., Fl. Brit. India (J.D. Hooker). 7(22): 374. 1896 (as “thomsoni”). Type: China: Western Tibet, Xizang, Danskar, Piti, alt. 13000 ft.; *T. Thomson s.n.*

**Distribution.** Uttar Pradesh, Uttarakhand [China, Pakistan].

**Habit.** Perennial.

**297. **Leymus duthiei** (Stapf ex Hook. f.), J. Syst. Evol. 47: 83. 2009 **

*Asperella duthiei* Stapf ex Hook. f., Fl. Brit. India (J.D. Hooker). 7(22): 375. 1896. Type: India: Western Himalaya, Tihri-Garhwal, alt. 7000–8000 ft.; *J.F. Duthie 14564* (K).

*Hystrix duthiei* (Stapf ex Hook. f.) Bor, Indian Forester 66(9): 544. 1940

*Macrohystrix duthiei* (Stapf ex Hook. f.) Tzvelev & Prob., Bot. Zhurn. (Moscow and Leningrad) 95: 858. 2010.

**Distribution. **Western Himalaya.

**Habit. **Perennial.

***Pseudoroegneria* (Nevski) Á. Löve, Taxon 29: 168. 1980**

*Elytrigia* sect. *Pseudoroegneria* Nevski, Trudy Sredne-Aziatsk. Gosud. Univ., Ser. 8b, Bot. 17: 60. 1934

**Type. ***Pseudoroegneria strigosa* (M. Bieb.) Á. Löve (*Bromus strigosus* M. Bieb.).

**298. **Pseudoroegneria cognata** (Hack.) A. Löve, Feddes Repert. 95: 446. 1984 **

*Agropyron cognatum* Hack., Allg. Bot. Z. Syst. 10: 22. 1904. Type: India: Kashmir; *J.F. Duthie 11895* (W).

*Elymus cognatus* (Hack.) Cope, Fl. Pakistan 143: 628. 1982

*Agropyron cognatum* Hack. var. *shingoense* Melderis in Bor, Grasses Burma, Ceylon, India and Pakistan 660, 690. 1960. Type: Baltistan: Kashmir, Shingo Valley, 10000–11000 ft.; July 7, 1893; *J.F. Duthie 11895* (K).

**Distribution.** Jammu and Kashmir, Ladakh [China, Pakistan].

**Habit.** Perennial.

**Remarks. **Records for India fide Naithani (1990).

***Secale* L., Sp. Pl. 1: 84. 1753**

**Lectotype. ***Secale cereale* L. LT designated by Designated by Hitchcock in U.S.D.A. Bull. 772: 91. 1920.

**299. **Secale cereale** L., Sp. Pl. 1: 84. 1753 **

*Triticum cereale* (L.) Salisb., Prodr. Stirp. Chap. Allerton 27. 1796

**Type. **“Habitat - - - -” Lectotype: Herb. Linn. No. 102.1 (LINN). LT designated by Bowden in Canad. J. Bot. 40: 1704. 1962.

**Distribution.** Himachal Pradesh, Madhya Pradesh, Meghalaya, Uttar Pradesh, Uttarakhand.

**Habit.** Annual.

**Remarks.** Native to Eurasia; widely cultivated.

**300. **Secale segetale** (Zhuk.) Roshev., Trudy Bot. Inst. Akad. Nauk S.S.S.R., Ser. 1, Fl. Sist. Vyssh. Rast. 6: 143. 1947 **

*Secale cereale* L. ssp. *segetale* Zhuk., Trudy Prikl. Bot. 19(2): 56. 1928

*Secale afghanicum* (Vavilov) Roshev., Trudy Bot. Inst. Akad. Nauk S.S.S.R., Ser. 1, Fl. Sist. Vyssh. Rast. 6: 139. 1947

*Secale cereale* L. var. *afghanicum* Vavilov, Trudy Prikl. Bot. 16(2): 77. 1926

**Distribution.** Jammu and Kashmir [China].

**Habit.** Annual.

**Remarks.** Record for India fide Naithani (1990).

***Triticum* L., Sp. Pl. 1: 85. 1753**

**Lectotype. ***Triticum aestivum* L. LT designated by Hitchcock in Amer. J. Bot. 10: 513. 1923.

**301. **Triticum aestivum** L., Sp. Pl. 1: 85. 1753, nom. cons. **

*Triticum compositum* L., Syst. Veg., ed. 13. 108. 1774. Neotype : Cultivated. “Triticum compositum L. Introdotto nella coltura del basso Egitto,” Herb. Figari (K). Neotype designated by Cope in Cafferty et al. (ed.), Taxon 49: 258. 2000.

*Triticum hybernum* L., Sp. Pl. 1: 86. 1753. Type: “Habitat - - - -” Lectotype: Herb. Clifford: 24, Triticum 2 (BM-000557669). LT designated by Hanelt et al. in Taxon 32: 496, f. 2. 1983.

*Triticum sativum* Lam., Fl. Franc. (Lamarck). 3: 625. 1779 (“1778”), nom. superfl. & illeg. for *Triticum turgidum*

*Triticum vulgare* Vill., Hist. Pl. Dauphiné (Villars) 2: 153. 1787. Type: “N. Froment Tourn. Tab. 293.”

**Type.** “Habitat - - - -” Lectotype: Herb. Clifford: 24, Triticum 3 (BM-000557670). LT designated by Bowden in Canad. J. Bot. 37: 674. 1959.

**Distribution.** Andhra Pradesh, Assam, Bihar, Delhi, Gujarat, Haryana, Himachal Pradesh, Karnataka, Madhya Pradesh, Maharashtra, Punjab, Rajasthan, Tamil Nadu, Tripura, Uttar Pradesh, Uttarakhand, West Bengal [China, Myanmar, Pakistan, Sri Lanka].

**Habit. **Annual.

**Remarks.** Taxonomic editor Soreng notes that this species is cultivated. It sporadically germinates on roadsides and pastures and market areas due to spillage of grain, but it would not persist on its own. See van Slageren (1994).

**302. **Triticum dicoccum** (Schrank) Schübl., Diss. Char. Descr. Cereal. 29. 1818 **

*Triticum spelta* L. var. *dicoccum* Schrank, Baier. Fl. 1: 389. 1789, sphalm. Type: Germany: Bavaria, Stuttgart; *H.R. Kerner s.n. *“ult. die vom H.R. Kerner von Stuttgart”; *H.R. Kerner s.n.*

*Triticum spelta* Host, Icon. Descr. Gram. Austriac. 3: 21, t. 30. 1805, non L., 1753

**Distribution.** Andhra Pradesh, Bihar, Madhya Pradesh, Maharashtra, Tamil Nadu, Uttar Pradesh, Uttarakhand [China].

**Habit.** Annual.

**Remarks.** Taxonomic editor Soreng notes that this species is cultivated. It sporadically germinates on roadsides and pastures and market areas due to spillage of grain, but it would not persist on its own.

**303. **Triticum durum** Desf., Fl. Atlant. 1: 114. 1798 **

*Triticum polonicum* L. ssp. *durum* (Desf.) A. Löve, Feddes Repert. 95: 497. 1984

**Type.** “Colitur in Barbaria.”

**Distribution.** Madhya Pradesh, Maharashtra, Tamil Nadu, Uttar Pradesh, Uttarakhand.

**Habit.** Annual.

**Remarks.** Taxonomic editor Soreng notes that this species is cultivated. It sporadically germinates on roadsides and pastures and market areas due to spillage of grain, but it would not persist on its own.

**304. **Triticum monococcum** L., Sp. Pl. 1: 86. 1753 **

*Crithodium monococcum* (L.) Á. Löve, Feddes Repert. 95: 490. 1984

**Lectotype.***Herb. Linn. No. 104.4* (LINN). LT designated by Bowden in Canad. J. Bot. 37: 664. 1959.

**Distribution.** India; states not recorded [China].

**Habit. **Annual.

**Remarks.** Taxonomic editor Soreng notes that this species is cultivated. It sporadically germinates on roadsides and pastures and market areas due to spillage of grain, but it would not persist on its own. Editor Kellogg questions whether this is truly cultivated in India.

**305. **Triticum polonicum** L., Sp. Pl., ed. 2. 1: 127. 1762 **

*Triticum glaucum* Moench, Methodus (Moench) 174. 1794, nom. superfl. & illeg. for *T. polonicum*

*Triticum levissimum* Hall., Stirp. Ind. Helv. 209. 1768

**Type.** “Habitat - - - -” Lectotype: Herb. Linn. No. 104.3 (LINN). LT designated by Bowden in Canad. J. Bot. 37: 670. 1959.

**Distribution.** Assam [China].

**Habit.** Annual.

**Remarks.** Taxonomic editor Soreng notes that this species is cultivated. It sporadically germinates on roadsides and pastures and market areas due to spillage of grain, but it would not persist on its own. The Linnaean typification project assigns the name *Gigachilon polonicum* (L.) Seidl ssp. *polonicum* to this taxon (http://www.nhm.ac.uk/our-science/data/linnaean-typification/), but Soreng notes that *Gigachilon* is invalid.

**306. **Triticum spelta** L., Sp. Pl. 1: 86. 1753 **

*Triticum aestivum* L. ssp. *spelta* (L.) Thell., Mitt. Naturwiss. Ges. Winterthur 12: 471. 1918

*Triticum zea* Host, Icon. Descr. Gram. Austriac. 3: 20. t. 29. 1805. Type: Seritur

**Type.** “Habitat - - -” Lectotype: Herb. Linn. No. 104.1 (LINN). LT designated by Morrison in Taxon 47: 709. 1998.

**Distribution.** India; states not recorded [China].

**Habit.** Annual.

**Remarks.** Taxonomic editor Soreng notes that this species is cultivated. It sporadically germinates on roadsides and pastures and market areas due to spillage of grain, but it would not persist on its own.

**307. **Triticum sphaerococcum** Percival, Wheat Pl. Monogr. 321. 1921 **

*Triticum aestivum* L. ssp. *sphaerococcum* (Percival) Mackey, Svensk. Bot. Tidskr. 48: 586. 1954

**Type.** India: parts of Persia; *Howard s.n.*

**Distribution.** Madhya Pradesh, Punjab.

**Habit. **Annual.

**Remarks. **Taxonomic editor Soreng notes that this species is cultivated. It sporadically germinates on roadsides and pastures and market areas due to spillage of grain, but it would not persist on its own.

**308. **Triticum turgidum** L., Sp. Pl. 1: 86. 1753 **

*Triticum polonicum* L. ssp. *turgidum* (L.) A. Löve, Feddes Repert. 95: 497. 1984

**Type.** “Habitat - - - -” Lectotype: Herb. A. van Royen No. 913.62–257 (L). LT designated by Morrison in Taxon 47: 707. 1998.

**Distribution.** Northwest India [China].

**Habit.** Annual.

**Remarks. **Taxonomic editor Soreng notes that this species is cultivated. It sporadically germinates on roadsides and pastures and market areas due to spillage of grain, but it would not persist on its own. The Linnaean typification project assigns the name *Gigachilon polonicum* (L.) Seidl ssp. *turgidum* (L.) Á. Löve to this taxon (Cafferty et al. 2000), but Soreng notes that *Gigachilon* is invalid.

### Tribe Poeae R. Br., 1814

Plants annual or perennial, rhizomatous or caespitose, inflorescence and spikelets variable; spikelets laterally compressed, disarticulating above the glumes. The *punctate hilum* may be synapomorphic; having an *endosperm with lipid* may also be derived here although this character is sparsely sampled.

**×**Agropogon** P. Fourn., Quatre Fl. France (ed. 1) 50. 1934 (parent: **Agrostis** L., 1753 × **Polypogon** Desf. 1798) **

**309. ×**Agropogon lutosus** (Poir.) P. Fourn., Quatre Fl. France (ed. 1) 50. 1934 **

[*Agrostis stolonifera* × *Polypogon monspeliensis*]

*Agrostis lutosa* Poir., Encycl. (Lamarck) Suppl. 1: 249. 1810, nom. nov. for *Agrostis littoralis* With.

*Agrostis subaristata* Aitch. & Hemsl., J. Linn. Soc., Bot. 19: 192. pl. 29. 1881. Type: Afghanistan: Kuram District, on margins of fields, alt. 7000 ft.; *J.E.T. Aitchison s.n.* (K).

*Polypogon subaristatus* (Aitch. ex Hemsl.) Bhattacharya & S.K. Jain, Curr. Sci. 49(11): 444. 1980

*Polypogon littoralis* Sm., Comp. Fl. Brit. 13. 1816, nom. nov.

*Agrostis littoralis* With., Arr. Brit. Pl., ed. 3. 2: 129. t. 23. 1796, non Lam., 1791. Type: England: Specimens from Wells, on the Norfolk coast

**Distribution.** Assam, Jammu and Kashmir [Afghanistan, China, Pakistan].

**Habit.** Perennial (short-lived).

**Remarks.** Widespread, sporadic across Eurasia, sterile.

***Agrostis* L., Sp. Pl. 1: 61. 1753, nom. cons.**

**Type. ***Agrostis canina* L. (typ. cons*.*).

Taxonomic editor Soreng notes that generic boundaries among *Agrostis*, *Calamagrostis* and *Deyeuxia* are confusing. No molecular studies have yet resolved the problem of how to divide these genera or determined if it is even possible to divide them. See also discussion under *Calamagrostis*.

**310. **Agrostis brachiata** Munro ex Hook. f., Fl. Brit. India (J.D. Hooker). 7(22): 256. 1896 **

**Type.** India: Bihar (“Behar”), Monghyr; *Wallich *(Numer. List [Wallich] no. 3769B).

**Distribution.** Bihar [Bhutan, China, Nepal].

**Habit.** Perennial.

**311. **Agrostis canina **L., Sp. Pl. 1: 62. 1753, nom. cons. **

*Agraulus caninus* (L.) P. Beauv., Ess. Agrostogr. 5, 147. t. 4. f. 7. 1812

*Trichodium caninum* (L.) Schrad., Fl. Germ. 1: 198. 1806

*Agrostis wightii* Nees, Syn. Pl. Glumac. (Steudel) 1: 168. 1854. Type: *Wight Herb. no. 78 *as* Vilfa vulgaris*

**Type.** “Habitat in Europae pascuis humidiusculis.” Conserved Type: Herb. Burser I: 3 (UPS). Type designated by Widén in Fl. Fennica 5 : 29. f. 25. 1971.

**Distribution.** Jammu and Kashmir, Tamil Nadu [China].

**Habit.** Perennial.

**312. **Agrostis capillaris** L., Sp. Pl. 1: 62 1753 **

*Agrostis tenuis* Sibth., Fl. Oxon. 36. 1794

*Agrostis alba* L. var. *tenuis* (Sibth.) Fiori, Nuov. Fl. Italia. 1: 97. 1923

*Agrostis hispida* Willd., Sp. Pl., ed. 4 [Willdenow] 1: 370. 1797. Type: “Habitat in Europae parties, pascuis et arvis.”

*Agrostis vulgaris* With. var. *hispida* (Willd.) Gaudin, Fl. Helv. 1: 191. 1828

*Agrostis stolonifera* L. fo. *hispida* (Willd.) Farw., Rep. (Annual) Michigan Acad. Sci. 21: 351. 1920

*Agrostis stolonifera* L. var. *vulgaris* (With.) Celak., Prodr. Fl. Bohmen. 4: 710. 1881 (as “1880”)

*Agrostis vulgaris* With., Arr. Brit. Pl., ed. 3. 2: 132. 1796. Type: England: very common but grows chiefly on poor dry and sandy soil

*Decandolia vulgaris* (With.) Bastard, Essai Fl. Maine-et-Loire 28. 1809

*Vilfa vulgaris* (With.) Gray, Nat. Arr. Brit. Pl. 2: 146. 1821

*Agrostis vulgaris* With. var. *mutica* G. Sinclair, Hort. Gram. Woburn. 269. 1824. Type: Germany

*Agrostis vulgaris* With. var. *plena* G. Mey., Fl. Kon. Hanov. 3, sig. 22. 1842

*Agrostis capillaris* Huds., Fl. Angl. (Hudson), ed. 2. 1: 27. 1778, non L., 1753

*Agrostis alba* L. var. *vulgaris* (With.) Plues, Brit. Grass. 151. 1867, non G. Mey., 1824

**Type.** ”Habitat in Europae pratis” LT: Herb. A. van Royen (L-912.356–69 (left-hand specimen); ILT: L); designated by Widen, Fl. Fenn. 5: ? (1971).

**Distribution.** Arunachal Pradesh, Sikkim, Uttar Pradesh.

**Habit.** Perennial.

**313. **Agrostis filipes** Hook. f., Fl. Brit. India (J.D. Hooker). 7(22): 256. 1896 **

**Type.** Unresolved. Several syntypes: “Khasia Hills, alt. 5–6000 ft., in several places, *Clarke*”*.*

**Distribution.** Assam, Bihar, Meghalaya [Pakistan].

**Habit.** Perennial.

**314. **Agrostis gigantea** Roth, Tent. Fl. Germ. 1: 31–32. 1788 **

*Agrostis stolonifera* L. var. *ramosa* (Gray) Veldkamp, Blumea 28(1): 223–225. 1982. Type: “Not indicated and according to Philipson not preserved. The type of the next name might be appointed as lectotype.”

*Vilfa alba* (L.) P. Beauv. var. *ramosa* Gray, Nat. Arr. Brit. Pl. 2: 145. 1821. Type: Marshes and Woods

**Type.** Europe: Germany; *Roth s.n.* (B). “Habitat inter arundinum et salices ad ripas Visurgi Ducatus Bremensis.”

**Distribution.** Jammu and Kashmir, Sikkim [Afghanistan, Nepal, Pakistan].

**Habit.** Perennial.

**315. **Agrostis griffithiana** (Hook. f.) Bor, Grasses Burma, Ceylon, India & Pakistan 387. 1960 **

*Calamagrostis griffithiana* Hook. f., Fl. Brit. India (J.D. Hooker). 7(22): 263. 1896. Syntypes: Western Himalaya: Kumaon, alt. 5500 ft.; *R. Strachey & J.E. Winterbottom s.n. *Khasia Hills, alt. 5000 ft.; *Griffith s.n., J.D. Hooker & Thomson s.n.*

*Agrostis wardii* Bor, Kew Bull. 1949: 444. 1949. Type: India: Manipur State, Sirhoi Kashong, 9400 ft.; 8.10.1948; *Kingdon-Ward 18145* (K).

**Distribution.** Assam, Manipur, Uttar Pradesh.

**Habit.** Perennial.

**Remarks.** See (Paszko and Pendry 2013).

**316. **Agrostis hissarica** Roshev., Bot. Mater. Gerb. Glavn. Bot. Sada RSFSR 4: 93. 1923 **

*Polypogon hissaricus* (Roshev.) Bor, Fl. Iranica 70: 307. 1970

*Agrostis stewartii* Bor, Kew Bull. 1956: 255. 1956. Type: India: Swat State, Kalam, alt. 8000 ft.; August 23, 1952; *R.R. Stewart 24733* (K).

**Syntypes.** Turkestan: Buchara, Hissar District, Sorbo. 8000 ft.; July 23, 1896; *W. Lipsky 3511*. Prov. Samerkand Utsch-Chada; July 20, 1915; *Balabajew s.n. *Minkwitz; *O. Knorring s.n. *Prov. Fergana, Distr. Andishan, Otusart, sub trajectu Taldy bel.; August 21, 1911; *Z. Minkwitz & O. Knorring 1791.*

**Distribution.** Jammu and Kashmir [Afghanistan, Pakistan].

**Habit. **Perennial.

**Remarks.** Taxonomic editor Soreng notes that *Polypogon hissaricus* (Roshev.) Bor is the accepted name in Lu and Phillips (2006) and Clayton et al. (2006 onwards). Distribution in India fide Naithani (1990).

**317. **Agrostis hookeriana** C.B. Clarke ex Hook. f., Fl. Brit. India (J.D. Hooker). 7(22): 256. 1896 **

**Type.** India: Sikkim Himalaya, in meadow, alt. 9000–11000 ft.; *J.D. Hooker s.n.*

**Distribution.** Sikkim [Bhutan, China, Nepal].

**Habit. **Annual.

**318. **Agrostis inaequiglumis** Griseb., Nachr. Königl. Ges. Wiss. Georg-Augusts-Univ. 80. 1868 **

**Type.** India: Sikkim, 12000–15000 ft.; *J.D. Hooker s.n.*

**Distribution.** Arunachal Pradesh, Sikkim, N West Bengal. [Bhutan, China, Nepal].

**Habit. **Annual.

**319. **Agrostis micrantha** Steud., Syn. Pl. Glumac. 1: 170. 1854 **

*Agrostis myriantha* Hook. f., Fl. Brit. India (J.D. Hooker). 7(22): 257. 1896. Lectotype: India: Sikkim, Lachen, 9000–10000 ft.; August 2, 1849; *J.D. Hooker & Thomson, Agrostis 7* (K). LT designated by H.J. Noltie in Edinburgh J. Bot. 56(3): 390. 1999.

*Agrostis myriantha* Hook. f. var. *khasiana* Hook. f., Fl. Brit. India (J.D. Hooker). 7(22): 257. 1896. Lectotype: India: Khasia; *J.D. Hooker & Thomson, Agrostis 8 *(part) (K). LT designated by H.J. Noltie in Edinburgh J. Bot. 56(3): 390. 1999.

*Agrostis myriantha* Hook. f. var. *sikkimensis* Hook. f., Fl. Brit. India (J.D. Hooker). 7(22): 257. 1896 (nom. superfl. for var. *myriantha* by lectotypification; H.J. Noltie in Edinburgh J. Bot. 56(3): 390. 1999).

*Agrostis platyphylla* Mez, Repert. Spec. Nov. Regni Veg. 17: 302. 1921. Holotype: J.D. Hooker & T. Thomson, Agrostis 8 (in part); no date; India: Khasia (B (photo, K); IT: US-73398 (fragm. ex B)) B destroyed

*Agrostis himalayana *Bor, Kew Bull. 1953: 269. 1953. Type: Assam: Balipara Frontier Tract, Nyukmadung, 6400 ft.; *F. Kingdon-Ward 11538* (BM).

**Type.** Nepal; *Wallich* (Numer. List [Wallich] no. 3776) (P).

**Distribution.** Arunachal Pradesh, Assam, Himachal Pradesh, Jammu and Kashmir, Meghalaya, Sikkim, Tamil Nadu, Uttar Pradesh, West Bengal [Bhutan, China, Myanmar, Nepal].

**Habit.** Perennial.

**320. **Agrostis munroana** Aitch. & Hemsl., J. Linn. Soc., Bot. 19: 192. 1882 ssp. **munroana

*Calamagrostis munroana* (Aitch. & Hemsl.) Boiss., Fl. Orient. (Boissier) 5: 523. 1884

*Calamagrostis munroana* (Aitch. & Hemsl.) Boiss. var. *stricta* Hook. f., Fl. Brit. India (J.D. Hooker). 7(22): 263. 1896. Type: Common at altitudes of 11000–15000 ft.; *R. Strachey & J.E. Winterbottom, Agrostis no 4*

**Type.** Afghanistan: Kuram District, Shend-toi gorge, alt. 10000–11000 ft.; *J.E.T. Aitchison s.n.*

**Distribution.** Himachal Pradesh, Jammu and Kashmir, Uttar Pradesh, Uttarakhand [Afghanistan, China, Nepal, Pakistan].

**Habit.** Annual.

**321. **Agrostis munroana** Aitch. & Hemsl. ssp. **indica** Bhattacharya & S.K. Jain, Bull. Bot. Surv. India 25(1–4): 205. f. 1. 1985 (“1983”) **

**Type.** India: Himachal Pradesh, Karcham-Brue Road; August 27, 1973; *Janardhanan 52862A* (CAL).

**Distribution.** Himachal Pradesh, Jammu and Kashmir, Uttar Pradesh.

**Habit.** Annual.

**Remarks.** Records for India fide Naithani (1990).

**322. **Agrostis nervosa** Nees ex Trin., Mém. Acad. Imp. Sci. Saint-Pétersbourg, Sér. 6, Sci. Math., Seconde Pt. Sci. Nat. 6, 4(3–4): 328. 1841 **

*Agrostis clarkei* Hook. f., Fl. Brit. India (J.D. Hooker). 7(22): 257. 1896. Type: India: Sikkim, alt. 9000–11000 ft.; *J.D. Hooker s.n.*

*Agrostis sikkimensis* Bor, Kew Bull. 1954: 502. 1954

*Agrostis divaricata* Griseb., Nachr. Königl. Ges. Wiss. Georg-Augusts-Univ. 81. 1868, non Hoffman, 1800. Type: Himalaya orientalis: Sikkim, alt. 9000–12000; *J.D. Hooker Agrostis no. 11*

**Type.** India; *Royle s.n.* (LE).

**Distribution.** Sikkim, Uttar Pradesh, Uttarakhand [Bhutan, China, Myanmar, Nepal].

**Habit.** Perennial.

**Remarks.** Taxonomic editor Soreng notes that Hooker cited four specimens for *A. clarkei*, which are syntypes. A lectotype appears not to have been designated yet.

**323. **Agrostis peninsularis** Hook. f., Fl. Brit. India (J.D. Hooker). 7(22): 255. 1896 **

**Syntypes.** India: Deccan Peninsula; *Wight Cat no. 1746*. Pulney Hills; *Wight s.n.*

**Distribution.** Kerala, Tamil Nadu [Afghanistan].

**Habit.** Annual.

**324. **Agrostis pilosula** Trin., Mém. Acad. Imp. Sci. Saint-Pétersbourg, Sér. 6, Sci. Math., Seconde Pt. Sci. Nat. 6, 4(3–4): 372. 1841 var. **pilosula

*Agrostis pilosula* Trin. var. *wallichiana* (Steud.) Bor, Kew Bull. 1954: 459. 1954

*Agrostis pilosula* Trin. f. *wallichiana* (Steud.) Bhattacharya & S.K. Jain, Bull. Bot. Surv. India 25(1–4): 210. 1985 (“1983”)

*Calamagrostis pilosula* (Trin.) Hook. f. var. *wallichiana* (Steud.) Hook. f., Fl. Brit. India (J.D. Hooker). 7(22): 264. 1896

*Agrostis wallichiana* Steud., Syn. Pl. Glumac. 1: 174. 1854. Type: Himalaya; *Wallich* (Numer. List [Wallich] no. 3775a)

*Agrostis royleana* Trin., Mém. Acad. Imp. Sci. Saint-Pétersbourg, Sér. 6, Sci. Math., Seconde Pt. Sci. Nat. 6,4(3–4): 371. 1841. Type: India; *Royle s.n. *(LE).

*Agrostis ciliata* Trin., Mém. Acad. Imp. Sci. Saint-Pétersbourg, Sér. 6, Sci. Math., Seconde Pt. Sci. Nat. 6, 4(3–4): 373. 1841. Type: India

*Calamagrostis ciliata* Nees ex Steud., Syn. Pl. Glumac. 1: 193. 1854. Type: Nepal; *Royle 67, 71 & 303*

*Calamagrostis hookeriana* Steud., Syn. Pl. Glumac. 1: 192. 1854. Type: Sri Lanka

*Calamagrostis jacquemontii* Hook. f., Fl. Brit. India (J.D. Hooker). 7(22): 265. 1896. Type: India: Kashmir; moist places on the Pyr Panjal; *Jacquemont no. 97*

*Calamagrostis neesii* Steud., Syn. Pl. Glumac. 1: 193. 1854. Type: Nepal; *Royle 72*

*Agrostis pilosula* Trin. var. *alpestris* (Hook. f.) Veldkamp, Blumea 28(1): 203. 1982

*Calamagrostis pilosula* (Trin.) Hook. f. var. *alpestris* Hook. f., Fl. Brit. India (J.D. Hooker). 7(22): 264. 1896. Type: Kashmir to Sikkim, alt. 11000–15000 ft.

*Calamagrostis pilosula* (Trin.) Hook. f., Fl. Brit. India (J.D. Hooker). 7(22): 263. 1896

*Calamagrostis pilosula* (Trin.) Hook. f. var. *ciliata* (Trin.) Hook. f., Fl. Brit. India (J.D. Hooker). 7(22): 264. 1896

*Lachnagrostis ciliata* Trin., Mém. Acad. Imp. Sci. Saint-Pétersbourg, Sér. 6, Sci. Math., Seconde Pt. Sci. Nat. 6, 4(3–4): 373. 1841, pro. syn., nom. nud. Type: “Ind. orient.”

*Calamagrostis pilosula* (Trin.) Hook. f. var. *scabra* Hook. f., Fl. Brit. India (J.D. Hooker). 7(22): 264. 1896. Type: India: in woods at about 7000–10000 ft., Kashmir to Sikkim. Hack. in *Herb. Duthie n. 10,088*, as *Agrostis bicuspidata* Hack. in *Herb. Duthie 7583*, as *Agrostisroyleana* Nees in Herb. *Royle s.n.*, as *Lachnagrostis scabra*.

*Calamagrostis roylei* Nees ex Steud., Syn. Pl. Glumac. 1: 193. 1854. Type: Nepal

*Deyeuxia royleana* (Trin.) Trimen, Syst. Cat. Fl. Pl. Ceylon 108. 1885

*Lachnagrostis royleana* Trin., Mém. Acad. Imp. Sci. Saint-Pétersbourg, Sér. 6, Sci. Math., Seconde Pt. Sci. Nat. 6, 4(3–4): 371. 1841, pro. syn., nom. nud. Type: “In Royle Herb. Ind. reg. mont. sup. no. 266”

*Lachnagrostis scabra* Nees ex Trin., Mém. Acad. Imp. Sci. Saint-Pétersbourg, Sér. 6, Sci. Math., Seconde Pt. Sci. Nat. 6, 4(3–4): 372. 1841, pro. syn., nom. nud. Type: “Ind. orient. reg. mont. super”; *Royle s.n.*

*Agrostis pilosula* Trin. var. *royleana* (Trin.) Bor, Kew Bull. 1954: 459. 1954, nom. illeg.

*Agrostis tungnathii* Bhattacharya & S.K. Jain, Bull. Bot. Surv. India 25(1–4): 204–205, f. 1. 1985 (“1983”). Type: India: Uttar Pradesh, Chamoli, on way to Tungnath, 12800 ft.; August 29, 1978; *Bhattacharya 1524A* (CAL).

**Type.** India; *Royle 72* (LE).

**Distribution.** Bihar, Himachal Pradesh, Jammu and Kashmir, Karnataka, Kerala, Sikkim, Tamil Nadu, Uttar Pradesh, Uttarakhand [Bhutan, China, Nepal, Pakistan, Sri Lanka].

**Habit.** Annual.

**Remarks. **Clayton et al. (2006 onwards) places *A. tungnathii* in synonymy here.

**325. **Agrostis pilosula** Trin. var. **filifolia** Bor, Kew Bull. 1954: 459. 1954 **

*Agrostis pilosula* Trin. f. *filiifolia* (Bor) Bhattacharya & S.K. Jain, Bull. Bot. Surv. India 25(1–4): 210. 1985 (“1983”)

**Syntypes. **India: Madras, Silva cascade, Riverside Pulneys; June 1, 1898; *A.G. Bourne 1022*. Lidcot Valley, Kodaikanal, Pulney; July 10, 1898; *A.G. Bourne 3143*. Bear Shola Valley, Pulneys; June 21, 1901; *A.G. Bourne 1486*.

**Distribution.** Himachal Pradesh, Jammu and Kashmir, Tamil Nadu.

**Habit.** Annual.

**326. **Agrostis pleiophylla** Mez, Repert. Spec. Nov. Regni Veg. 17: 301. 1921. **

**Lectotype.** C.B. Clarke 26852; 31 Jul 1875; Sikkim: Darjeeling, 2280 m (B100448371) LT designated by B. Paszko and Soreng 2013, to supersede that by Noltie.

**Distribution. **Assam, Khasa Hills, Meghalaya, Sikkim.

**Habit.** Perennial.

**Remarks.** See Paszko and Soreng (2013) for a discussion of typification of this species. Taxonomic editor Soreng notes that this is not a synonym of *A. zenkeri*, but rather is a good taxon on its own.

**327. **Agrostis schmidii** (Hook. f.) C.E.C. Fisch., Fl. Madras 3(10): 1810. 1934 **

*Calamagrostis schmidii* Hook. f., Fl. Brit. India (J.D. Hooker). 7(22): 264. 1896. Type: India: Tamil Nadu, Nilghiri Hills, Ootacamund; *Schmid s.n.*

**Distribution.** Tamil Nadu.

**Habit.** Perennial.

**328. **Agrostis stolonifera** L., Sp. Pl. 1: 62. 1753 **

*Agrostis alba* sensu Hook. f., Fl. Brit. India (J.D. Hooker). 7(22): 254. 1896, non L., 1753

**Type.** “Habitat in Europa.” Lectotype: *Herb. A. van Royen, sheet no. 912.356–55* (L). LT designated by Widén in Fl. Fennica 5: 77. f. 28. 1971.

**Distribution. **Andhra Pradesh, Himachal Pradesh, Jammu and Kashmir, Kerala, Meghalaya, Punjab, Sikkim, Tamil Nadu, Uttar Pradesh [Bhutan, Myanmar, Nepal, Sri Lanka].

**Habit.** Perennial.

**329. **Agrostis tripilifera** (Hook. f.) Forsan, ined. **

*Agrostis triaristata* (Hook. f.) Bor, Grasses Burma, Ceylon, India and Pakistan 391. 1960, nom. later hom., non Knapp, 1804

*Calamagrostis tripilifera* Hook. f., Fl. Brit. India (J.D. Hooker). 7(22): 262. 1896. Syntypes. India: Sikkim, in the drier interior, 10000–13000 ft.; *J.D. Hooker s.n.; Clarke s.n, King’s Collector*

*Deyeuxia triaristata* Hook. f., Fl. Brit. India (J.D. Hooker). 7(22): 266. 1896. Type: Sikkim Himalaya: in woods, Yeumtong, alt. 12000 ft.; *J.D. Hooker s.n.*

*Deyeuxia tripilifera* (Hook. f.) Keng, Sunyatsenia 6(1): 68. 1941

**Distribution.** Sikkim.

**Habit.** Perennial.

**Remarks.** This combination is accepted by Clayton et al. (2006 onwards) but appears not to be published. See also Paszko (2012b).

**330. **Agrostis vinealis** Schreb., Spic. Fl. Lips. 47. 1771 **

*Agrostis canina* sensu Hook. f., Fl. Brit. India (J.D. Hooker). 7(22): 255. 1896, non L.,1753

**Type. **Germany: “In siccioribus ad Schoenfeld templum S. Theclae.”

**Distribution. **Tamil Nadu, Uttar Pradesh, Uttarakhand [Pakistan].

**Habit.** Perennial.

**331. **Agrostis zenkeri** Trin., Mém. Acad. Imp. Sci. Saint-Pétersbourg, Sér. 6, Sci. Math., Seconde Pt. Sci. Nat. 6, 4(3–4): 363. 1841 **

*Deyeuxia zenkeri* (Trin.) Veldkamp, The Gardens’ Bulletin Singapore 37(2): 219. 1984[1985].

*Calamagrostis zenkeri* (Trin.) Davidse, in M.D. Dassanayake et al. (eds.), Revised Handb. Fl. Ceylon 8: 107. 1994

**Type.** India: Tamil Nadu, Nilagiri;* Zenker s.n.* (LE-TRIN-1669.01).

**Distribution.** Tamil Nadu.

**Habit.** Perennial (perhaps annual?).

**Remarks.** Paszko argues that this species is properly placed in *Agrostis* (Paszko and Soreng 2013)

***Aira* L., Sp. Pl. 1: 63. 1753, nom. cons.**

**Type. ***Aira praecox* L. (typ. cons.)

**332. **Aira caryophyllea** L., Sp. Pl. 1: 66. 1753 **

**Type.** “Habitat in Angliae, Germaniae, Galliae glareosis.” Lectotype: *Herb. Linn. No. 85.22 *(LINN). LT designated by Clayton in Milne-Redhead and Polhill (ed.), Fl. Trop. E. Africa, Gramineae 1: 84. 1970.

**Distribution.** Himachal Pradesh [China].

**Habit.** Annual.

***Alopecurus* L., Sp. Pl. 1: 60. 1753**

**Lectotype. ***Alopecurus pratensis* L. LT designated by Nash in N.L. Britton and A. Brown, Ill. Fl. N. U.S. ed. 2. 1: 191. 1913; affirmed by Hitchcock in Bull. U.S.D.A 772: 137. 1920.

**333. **Alopecurus aequalis** Sobol., Fl. Petrop. 16. 1799 **

*Alopecurus caespitosus* Trin., Sp. Gram. [Trinius] 3(21): t. 241. 1829. Type: “Figura ad specimen Boreali-Americanum.” NW America: Douglas; *J. Lindley s.n. *(LE-TRIN-1537.01).

*Alopecurus diandrus* Griff., Not. Pl. Asiat. 3: 11. 1851. Type: India: “Assam in ripas arenosis Brahmaputra fluminis rarus March 1836. It. Ass. 45.”

*Alopecurus aristulatus* Michx., Fl. Bor.-Amer. (Michaux) 1: 43. 1803. Type: “Hab. in Paludosis Canadae”; *H. Pittier, T.A. Durand s.n.*

*Alopecurus fulvus* Sm., Engl. Bot. 21: t. 1467. 1805. Type: England: Swainsthorpe, four miles south of Norwich; *Stone s.n.*

*Alopecurus subaristatus* Pers., Syn. Pl. (Persoon) 1: 80. 1805, nom. superfl & illeg.

**Type.** “Habitat in locis uliginosis cum Alopecurus geniculatus, in lacubus natans est”; *Sobolewski s.n.* (LE).

**Distribution.** Assam, Jammu and Kashmir, Nagaland, Uttar Pradesh [Afghanistan, China, Nepal, Pakistan].

**Habit.** Annual or perennial.

**Remarks.** Records for Assam, Jammu and Kashmir, and Nagaland fide Naithani (1990).

**334. **Alopecurus arundinaceus** Poir., Encycl. (Lamarck) 8: 776. 1808 **

*Alopecurus ventricosus* Pers., Syn. Pl. (Persoon) 1: 80. 1805, non Huds. 1778. Type: “Hab. in Gallia.”

**Type.** “Cette plante est cultivate au Jardin des Plantes de Paris Jignore son lieu natal. (herb. Desfont.)”

**Distribution.** Jammu and Kashmir, Uttar Pradesh [China, Pakistan].

**Habit.** Perennial.

**335. **Alopecurus geniculatus** L., Sp. Pl. 1: 60. 1753 **

**Type.** “Habitat in Europae uliginosis.” Lectotype: *Herb. Burser I: 26* (UPS). LT designated by Cope in Cafferty et al. (ed.), Taxon 49: 245. 2000.

**Distribution.** Uttar Pradesh, Uttarakhand [Afghanistan, Nepal].

**Habit.** Perennial.

**336. **Alopecurus himalaicus** Hook. f., Fl. Brit. India (J.D. Hooker). 7(22): 238. 1896 **

**Type.** India: Kashmir and Dras, alt.10000–14500 ft.; *Falconer s.n.* (K).

**Distribution.** Jammu and Kashmir [Afghanistan, China, Pakistan].

**Habit.** Perennial.

**337. **Alopecurus myosuroides** Huds., Fl. Angl. (Hudson) 23. 1762 **

*Alopecurus agrestis* L., Sp. Pl., ed. 2. 1: 89. 1762. Type: “Habitat in Europa australi.” Lectotype: *Hudson 29, Herb. Linn. No. 82.2* (LINN). LT designated by Cope in Cafferty et al. (ed.), Taxon 49: 245. 2000.

**Type.** “Habitat in arvis, et ad vias”; *Hudson s.n.*

**Distribution.** Jammu and Kashmir, Nagaland [Afghanistan, Pakistan].

**Habit.** Annual.

**338. **Alopecurus nepalensis** Trin. ex Steud., Syn. Pl. Glumac. 1: 148. 1854 **

*Alopecurusborii* Tzvelev, Vasailchenko, Nov. Syst. Plant Vasc. 8: 21. 1971. Type: Turcomania, in valley fl. Amu-Darja, 15 km ad astros versus urb. Czardzhou; 8 May 1958, *T. Nadezhina s.n.* (LE)

**Type.** Nepal; *Wallich *(Numer. List [Wallich] no. 3780).

**Distribution.** Jammu and Kashmir, Uttar Pradesh [Nepal, Pakistan].

**Habit.** Annual.

**Remarks.** Taxonomic editor Soreng notes that Doğan (1999) places *A. borii* in the synonymy of *A. nepalensis*.

***Aniselytron* Merr., Philipp. J. Sci., C. 5: 328. 1910**

**Type. ***Aniselytron agrostoides* Merr.

**339. **Aniselytron treutleri** (Kuntze) Soják, Cas. Nar. Muz. Praze, Rada Prir. 148(3–4): 202. 1980 **

*Milium treutleri* Kuntze, Revis. Gen. Pl. 780. 1891. Type: India: Sikkim: Tanglo, beim Dorf Shimong, 10500 ft.; August 5, 1874; *Von Treutler 486* (K).

*Calamagrostis treutleri* (Kuntze) U. Shukla, non Govaerts. Grasses N.E. India 51. 1996.

*Deyeuxia treutleri* (Kuntze) Stapf, non Rauschert. Hooker’s Icones Plantarum 24(4): pl. 2396. 1895.

*Neoaulacolepis treutleri* (Kuntze) Rauschert. Taxon 31(3): 561. 1982.

*Aniselytron treutleri* (Kuntze) Bennet & Raizada, Indian Forester 107(7): 434. 1981, isonym, nom. inval.

**Distribution.** Sikkim, West Bengal [Bhutan, China, Myanmar].

**Habit.** Perennial.

***Anthoxanthum* L., Sp. Pl. 1: 28. 1753**

**Lectotype. ***Anthoxanthum odoratum* L. LT designated by Nash in N.L. Britton et A. Brown, Ill. Fl. N. U.S. ed. 2. 1: 171. 1913; affirmed by Hitchcock, Bull. U.S.D.A 772: 201. 1920.

**340. **Anthoxanthum borii **S.K. Jain & D.C. Pal, J. Bombay Nat. Hist. Soc. 72: 92. 1975 **

**Type.** India: Tamil Nadu, Palnis, Pambar Stream near Shenthadikanal; December 6, 1898; *Bourne 1954* (CAL).

**Distribution.** Kerala, Tamil Nadu.

**Habit.** Perennial.

**Remarks.** Record from Tamil Nadu fide Naithani (1990).

**341. **Anthoxanthum flexuosum** (Hook. f.) Veldkamp, Blumea 30(2): 347. 1985 **

*Hierochloe flexuosa* Hook. f., Fl. Brit. India (J.D. Hooker). 7(21): 222. 1896. Type: India: Sikkim Himalaya, Bijean; *King’s collector s.n.* (K).

**Distribution.** Sikkim [Bhutan, China].

**Habit.** Perennial.

**342. **Anthoxanthum hookeri** (Griseb.) Rendle, J. Linn. Soc., Bot. 36: 380. 1904 **

*Ataxia hookeri* Griseb., Nachr. Königl. Ges. Wiss. Georg-Augusts-Univ. 77. 1868. Type: India: Sikkim, 9000–12000 ft.; *J.D. Hooker Ataxia no. 2*

*Hierochloe hookeri* (Griseb.) Maxim., Bull. Acad. Imp. Sci. Saint-Pétersbourg 32: 627. 1888

*Hierochloe hookeri* (Griseb.) C.B. Clarke ex Hook. f., Fl. Brit. India (J.D. Hooker). 7(21): 223. 1896, isonym, non (Griseb) Maxim., 1888

**Distribution.** Tamil Nadu, Sikkim [Bhutan, China, Myanmar, Nepal].

**Habit.** Perennial.

**343. **Anthoxanthum horsfieldii** (Kunth ex Bennett) Mez, Repert. Spec. Nov. Regni Veg. 17: 291. 1921 **

*Ataxia horsfieldii* Kunth ex Bennett, Pl. Jav. Rar. (Bennett) 8, pl. 3. 1838

*Hierochloe horsfieldii* (Kunth ex Benn.) Maxim., Diagn. Pl. Nov. Asiat. 7: 930. 1888

*Anthoxanthum formosanum* Honda, Bot. Mag. (Tokyo) 40(474): 318. 1926

*Anthoxanthum horsfieldii* var. *formosanum* (Honda) Veldkamp, J. Jap. Bot. 68(6): 344. 1993

*Anthoxanthum horsfieldii* var. *luzoniense* (Merr.) Y. Schouten, Blumea 30(2): 337. 1985

*Anthoxanthum japonicum* ssp. *luzoniense* (Merr.) T. Koyama, Grass. Jap. Neighb. Reg. 486. 1987

*Anthoxanthum luzoniense* Merr., Philipp. J. Sci. 1(Suppl.): 178. 1906

*Anthoxanthum horsfieldii* var. *viridescens* (Honda) Veldkamp, J. Jap. Bot. 68(6): 344. 1993

*Anthoxanthum viridescens* Honda, Bot. Mag. (Tokyo) 41: 379. 1927

*Anthoxanthum clarkei* (Hook. f.) Ohwi, Bull. Tokyo Sci. Mus. 18: 8. 1947

*Hierochloe clarkei* Hook. f., Fl. Brit. India (J.D. Hooker). 7(21): 223. 1896. Type: India: Khasia Hills, Lailan Kote, alt. 5500; *C.B. Clarke s.n.*

**Distribution.** Assam, Meghalaya, Sikkim [China].

**Habit.** Perennial.

**344. **Anthoxanthum khasiana** (C.B. Clarke) Ohwi, Bulletin of the Tokyo Science Museum 18: 8. 1947 **

*Hierochloe khasiana* C.B. Clarke ex Hook. f., Fl. Brit. India (J.D. Hooker). 7(21): 223. 1896. Type: India: Khasia Hills, alt. 4500–6000 ft.; *C.B. Clarke s.n.*

**Distribution.** Meghalaya.

**Habit.** Perennial.

**345. **Anthoxanthum laxum** (R. Br. ex Hook. f.) Veldkamp, Blumea 30(2): 348. 1985 **

*Hierochloe laxa* R. Br. ex Hook. f., Fl. Brit. India (J.D. Hooker). 7(21): 222. 1896. Type: Western Himalaya, Kashmir, from alt. 10000–12000 ft. to Kumaon, alt. 12000–16000 ft.; *R. Brown* (Numer. List [Wallich] no. 3796)

**Distribution.** Jammu and Kashmir, Uttar Pradesh [Pakistan].

**Habit.** Perennial.

**346. **Anthoxanthum odoratum** L., Sp. Pl. 1: 28. 1753 **

**Type. **“Habitat in Europae pratis.” Lectotype: *Herb. Linn. No. 46.1* (LINN). LT designated by Cope in Jarvis et al. (ed.), Regnum Veg. 127: 19. 1993.

**Distribution.** Himachal Pradesh, Meghalaya, Tamil Nadu, Uttar Pradesh, Uttarakhand [China, Pakistan].

**Habit.** Perennial.

**347. **Anthoxanthum sikkimense** (Maxim.) Ohwi, Bull. Tokyo Sci. Mus. 18: 8. 1947 **

*Hierochloe sikkimensis* Maxim., Bull. Acad. Imp. Sci. Saint-Pétersbourg 32: 626. 1888. Type: India: Sikkim; *J.D. Hooker & T. Thomson Ataxia 1*

*Anthoxanthum gracillimum* (Hook. f.) Mez, Repert. Spec. Nov. Regni Veg. 17: 291. 1921

*Hierochloe gracillima* Hook. f., Fl. Brit. India (J.D. Hooker). 7(21): 223. 1896. Type: India: Sikkim, alt. 10000–11000 ft.; *J.D. Hooker s.n.*

**Distribution.** Sikkim [China, Nepal].

**Habit.** Perennial.

**348. **Anthoxanthum tibeticum** (Bor) Veldkamp, Blumea 30(2): 350. 1985 **

*Hierochloe tibetica* Bor, Kew Bull. 1953: 271. 1953

**Distribution.** Sikkim. [China].

**Habit.** Perennial.

**Remarks.** Record in India fide Naithani (1990).

***Arctopoa* (Griseb.) Prob., Novosti Sist. Vyssh. Rast. 11: 49. 1974**

*Glyceria* sect. *Arctopoa* Griseb., in Ledeb., Fl. Ross. 4(13): 392 1852

**Type.***Glyceria glumaris* (Trin.) Griseb. ==*Arctopoa eminens* (J. Presl) Prob.

**349. **Arctopoa tibetica** (Munro ex Stapf) Prob., Novosti Sist. Vyssh. Rast. 11: 52 1974 **

*Poa tibetica* Munro ex Stapf, Fl. Brit. India (J.D. Hooker). 7(22): 339. 1896 var. tibetica. Syntypes: China: Western Tibet, Piti, alt. 9000–16000 ft.; *Jacquemont s.n.* Ladakh; *Thomson s.n.* Plains of Tibet north of Kumaon, alt. 15000 ft.; *R. Strachey & J.E. Winterbottom s.n.*

**Distribution.** Himachal Pradesh, Jammu and Kashmir, Uttar Pradesh [China, Nepal, Pakistan].

**Habit.** Perennial.

**Remarks.** Taxonomic editor Soreng notes that *Arctopoa* has been demonstrated be of old reticulate origin (Gillespie et al. 2008), derived from an extinct parent (chloroplast donor) from an early lineage of *Poa* crossed with a parent from a lineage outside *Poa* but related to *Cinna* and *Arctagrostis* (nrDNA donor). The genus has diversified and now includes at least three well marked and stable species, although Probatova (2003) recognizes eight.

**350. **Arctopoa tibetica** (Munro ex Stapf) Prob. var. **aristulata** (Stapf) E.A. Kellogg, comb. nov. **

urn:lsid:ipni.org:names:77212054-1

*Poa tibetica* Munro ex Stapf var. *aristulata* Stapf, Fl. Brit. India (J.D. Hooker). 7(22): 339. 1896. Type: Eastern Tibet, North of Sikkim, alt. 16000–17000 ft.; *J.D. Hooker s.n.*

*Poa pseudotibetica* Noltie, Edinburgh Journal of Botany 57(2): 279–281, f. 1A–D. 2000. (Edinburgh J. Bot.). Type: Sikkim; Chholhamoo, marshy meadows, 17820 ft, 6 Aug. 1972, *Pradham, Norbu & Naku 206* (HT: E00393981)

**Distribution.** Sikkim [China, Pakistan].

**Habit.** Perennial.

**Remarks.** Taxonomic editor Soreng notes that recognizing this variety requires a new combination in *Arctopoa*, which we make here. It could also be recognized as a separate species but he does not feel that this is warranted.

***Avena* L., Sp. Pl. 1: 79. 1753**

**Lectotype. ***Avena sativa* L. LT designated by Nash in N.L. Britton et A. Brown, Ill. Fl. N. U.S. ed. 2. 1: 218. 1913; affirmed by Hitchcock, Bull. U.S.D.A. 772: 110. 1920.

**351. **Avena barbata** Pott ex Link in Schrader, J. Bot. (Schrader) 2: 315. 1799 **

**Lectotype.** Lusitania, ex cult. mea; July 1796; *Pott s.n.* (LE).

**Distribution.** Tamil Nadu, Uttar Pradesh [Nepal, Pakistan].

**Habit.** Annual.

**352. **Avena byzantina** C. Koch, Linnaea 21: 392. 1848 **

**Type.** “Ich erhielt sie auch unter den Thirkenschen aus der Umgegend von Brussa gesammelten Pflanzen.”

**Distribution.** Bihar, Tamil Nadu, Himachal Pradesh [Pakistan].

**Habit.** Annual.

**Remarks.** Taxonomic editor Soreng notes that this is a Mediterranean taxon, presumably introduced to India in cultivation. It was placed in *Avena sativa* by Baum (1977). This species does grow outside of cultivation as a weed in other crops and disturbed places around where it is cultivated. It is recognized as a separate species by some taxonomists (e.g. by Chiapella and Amarilla (2012)), based on its slender, non-geniculate awn on both florets. Other authors (e.g. http://www.emplantbase.org/home.html) consider it as a subspecies – *Avena sativa* subsp. *byzantina* (K. Koch) Romero Zarco (Euro+Med Plantbase on-line). The latter treatment seems preferable.

**353. **Avena fatua** L., Sp. Pl. 1: 80. 1753 **

*Avena fatua* L. var. *pilosissima* Gray, Nat. Arr. Brit. Pl. 2: 131. 1821. Type: Corn fields

*Avena fatua* L. var. *glabrata* Peterm., Fl. Bienitz. 13. 1841

*Avena sativa* L. var. *sericea* Hook. f., Fl. Brit. India (J.D. Hooker). 7(22): 275. 1896. Type: Bhutan; *Griffith s.n.*

*Avena fatua* L. var. *pilosa* Syme, Rep. Bot. Exch. Club 1871: 20. 1872. Type: Beanfield Claygate, Surrey; *H.C. Watson s.n.*

*Avena fatua* L. ssp. *meridionalis* Malzev, Bull. Appl. Bot. Gen. Pl. Br., Suppl. 38: 304. 1930

*Avena meridionalis* (Malzev) Roshev., Fl. Turkmen. 1: 105. 1932

**Type.** “Habitat in Europae agris inter segetes.” Lectotype: *Herb. Linn. No. 95.9* (LINN). LT designated by Hubbard in Milne-Redhead and Polhill (ed.), Fl. Trop. E. Africa, Gramineae 1: 82. 1970.

**Distribution.** Arunachal Pradesh, Assam, Himachal Pradesh, Jammu and Kashmir, Meghalaya, Punjab, Rajasthan, Sikkim, Tamil Nadu, Uttar Pradesh, Uttarakhand, West Bengal [Afghanistan, Bhutan, China, Nepal, Pakistan].

**Habit.** Annual.

**Remarks.** Record from Jammu and Kashmir fide Naithani (1990).

**354. **Avena sativa** L., Sp. Pl. 1: 79. 1753 **

**Lectotype.***Herb. Clifford: 25, Avena 1* (BM-000557678). LT designated by Baum in Taxon 23: 579. f. 1. 1974.

**Distribution.** Bihar, Himachal Pradesh, Maharashtra, Madhya Pradesh, Punjab,

Odisha, Sikkim, Tamil Nadu, Uttar Pradesh, West Bengal [China].

**Habit.** Annual.

**Remarks.** It is unclear whether this species is naturalized or only cultivated.

**355. **Avena sterilis** L., Sp. Pl., ed. 2. 1: 118. 1762 var. **sterilis

**Type.** “Habitat in Hispania. Alströmer.” Lectotype: Alströmer 7, Herb. Linn. No. 95.12 (LINN). LT designated by Hubbard in Milne-Redhead and Polhill (ed.), Fl. Trop. E. Africa, Gramineae 1 : 84. 1970.

**Distribution.** Maharashtra, Tamil Nadu, Uttar Pradesh, Uttarakhand [Pakistan].

**Habit.** Annual.

**356. **Avena sterilis** L. var. **ludoviciana** (Durieu) Gillet & Magne, Nouv. Fl. Franc., ed. 3, 532. 1875 **

*Avena ludoviciana* Durieu, Actes Soc. Linn. Bordeaux 20: 41. 1855. Type: “Cette espece est assez frequente dans les environs de Bordeaux, aux lieux deja cites, elle semble preferer les terrain calcaires aux siliceux, quand I’A.” France: Bordeaux; May 27, 1855; *G. Lespinasse s.n.* (LE). [France]: de la Garonne: Bordeaux; July 6 1855; *F. Schultz 386 herbarium normale. Cent. 4.* (LE).

**Distribution.** Delhi, Jammu and Kashmir, Rajasthan, Tamil Nadu, Uttar Pradesh [Pakistan].

**Habit.** Annual.

**Remarks.** Records for Jammu and Kashmir and Uttar Pradesh fide Naithani (1990).

***Briza* L., Sp. Pl. 1: 70. 1753**

*Macrobriza* (Tzvelev) Tzvelev Komarovskie Čtenija (Moscow and Leningrad) 37: 32 1987

**Lectotype. ***Briza media *L. LT designated by Hitchcock, Bull. U.S.D.A. 772: 45. 1920.

**357. **Briza maxima** L., Sp. Pl. 1: 70. 1753 **

*Macrobriza maxima* (L.) Tzvelev., Bot. Žhurn. (Moscow and Leningrad) 78(10): 91 1993

**Type.** “Habitat in Italia, Lusitania.” Lectotype: *Seguier, Herb. Linn. No. 88.6* (LINN). LT designated by Hubbard in Milne-Redhead and Polhill (ed.), Fl. Trop. E. Africa, Gramineae 1: 53. 1970.

**Distribution.** Karnataka, Meghalaya, Tamil Nadu [China, Pakistan].

**Habit.** Annual.

**358. **Briza media** L., Sp. Pl. 1: 70. 1753 **

**Type.** “Habitat in Europae partis [sic] siccioribus.” Lectotype: *Herb. Linn. No. 88.5* (LINN). LT designated by Meikle in Fl. Cyprus 2: 1720. 1985.

**Distribution.** Himachal Pradesh, Jammu and Kashmir, Tamil Nadu, Sikkim, Uttar Pradesh [Bhutan, Nepal, Pakistan].

**Habit.** Perennial.

**359. **Briza minor** L., Sp. Pl. 1: 70. 1753 **

**Type.** “Habitat in Helvetia, Italia.” Lectotype: *Herb. Linn. No. 88.1* (LINN). LT designated by Hubbard in Milne-Redhead and Polhill (ed.), Fl. Trop. E. Africa, Gramineae 1: 53. 1970.

**Distribution.** Himachal Pradesh, Tamil Nadu. Perhaps naturalized. [China].

**Habit.** Annual.

**Remarks.** Possibly naturalized. Records for India fide Naithani (1990).

***Calamagrostis* Adans., Fam. Pl. (Adanson) 2: 31, 530. 1763**

*Deyeuxia* Clarion ex P. Beauv., Ess. Agrostogr. 43. 1812. LT: *Deyeuxia montana* P. Beauv. (*D*. *pyramidalis* (Host.) Veldkamp), designated by Niles & Chase, Contr. US Natl. Herb. 24: 169. 1925.

**Type.***Calamagrostis lanceolata* Roth (Tent. Fl. Germ. 2: 34. 1789) (*Arundo calamagrostis* L.).

**Notes.** The treatment here of *Calamagrostis* and *Deyeuxia* should be considered provisional as the generic boundaries are unclear. The two are considered distinct by Lu et al. (2006) and Kellogg (2015b). Taxonomic editor Soreng notes that *Calamagrostis* is deeply polyphyletic (Saarela et al. 2010). See also Paszko and Chen (2013), and Paszko and Ma (2011).

**360. **Calamagrostis arundinacea** (L.) Roth, Tent. Fl. Germ. 1: 33. 1788 **

*Agrostis arundinacea* L., Sp. Pl. 1: 61. 1753. Type: “Habitat in Europae monticulis sylvosis glareosis juniperitis.” Lectotype: *Amman 26, Herb. Linn. No. 84.7 *(LINN). LT designated by Veldkamp in Cafferty et al. (ed.), Taxon 49: 243. 2000.

*Deyeuxia sylvatica* (Schrad.) Kunth, Révis. Gramin. 1: 77. 1829

*Calamagrostis pyramidalis* Host., Icones et Descriptiones Graminum Austriacorum 4: 28, pl. 49. 1809. (Icon. Descr. Gram. Austriac.). Type: In sylvis Pannoniae...Bruck an der Leitha...Junio, Julio, *Anon. s.n*. (W?)

*Deyeuxia pyramidalis* (Host.) Veldkamp, Blumea 37(1): 230. 1992

*Deyeuxia montana* P. Beauv., Ess. Agrostogr. 43. 1812.

*Arundo montana *Gaudin, nom. illeg. superfl. for* Agrostis arundinacea* L.

*Arundo sylvatica* Schrad., Fl. Germ. (Schrader) 1: 218. t. 4. f. 7. 1806 (as “Siluativa”), nom. superfl. & illeg.

*Deyeuxia arundinacea* (L.) Jansen, Acta Bot. Neerl. 1: 470. 1952, nom. illeg. hom., non P. Beauv., 1812

**Distribution.** Jammu and Kashmir [China, Pakistan].

**Habit.** Perennial.

**Remarks.** Taxonomic editor Soreng notes that *Deyeuxia arundinacea* P. Beauv. has no connection with any Indian species and is equal to *Ampelodesmos mauritanicus* (Poir.) T. Durand & Schinz.

**361. **Calamagrostis canadensis** (Michx.) Beauv., Ess. Agrost. 15: 157. 1812 **

*Calamagrostis cinnoides* Barton, Compend. Fl. Phila. 1:45. 1818.

**Distribution.** Jammu and Kashmir, Uttar Pradesh.

**Habit.** Perennial.

**Remarks. **Distribution in India fide Naithani (1990). However, these records are dubious. Taxonomic editor Soreng notes that the only correct name for this as misapplied is *Calamagrostisnuttaliana* Steud., but he remains skeptical that this should be in Asia at all.

**362. **Calamagrostis debilis** Hook. f., Fl. Brit. India (J.D. Hooker). 7(22): 262. 1896 **

*Deyeuxia debilis* (Hook. f.) Veldkamp, Gard. Bull. Singapore 37(2): 220. 1985

*Agrostis debilis* (Hook. f.) Bor, Grasses Burma, Ceylon, India and Pakistan. 387. 1960, non Poir, 1810

*Agrostis neodebilis* Bennet & Raizada, Indian Forester 107(7): 433. 1981

**Type.** India: Sikkim: Chola, 9800 ft.; *J.D. Hooker s.n. *(K).

**Distribution.** Sikkim [China].

**Habit.** Perennial.

**363. **Calamagrostis decora** Hook. f., Fl. Brit. India (J.D. Hooker). 7(22): 260. 1896 **

**Type.** India: Kashmir, Astor Valley, alt. 9000–10000 ft.; *J.F. Duthie no. 12600.*

**Distribution.** Jammu and Kashmir [Pakistan].

**Habit. **Perennial.

**364. **Calamagrostis diffusa** (Keng) Keng f., Bull. Bot. Res., Harbin 4(3): 195–196. 1984 **

*Deyeuxia diffusa* Keng, Sunyatsenia 6(2): 94–95. 1941. Type: China: Yunnan: 1900–2750 m, *E.E. Maire 6863* p.p.

*Agrostis nagensis* Bor, Kew Bull. 1954: 497. 1954. Type: India: Assam, Naga Hills, Japvo, alt. 7500 ft.; September 28, 1935; *N.L. Bor 6449* (K).

*Calamagrostis nagensis* (Bor) Uniyal, J. Econ. Taxon. Bot. 8(1): 235. 1986

*Deyeuxia nagensis* (Bor) Veldkamp, J. Econ. Taxon. Bot. 13(1): 74. 1989

*Aulacolepis petelotii* Hitchc., J. Wash. Acad. Sci. 24(7): 291. 1934 (= *Agrostis abnormis* Munro ex Hook. f, Fl. Brit. India 7(22): 268. 1897[1896]; nom. inval., as syn. *Deyeuxia abnormis* Munro ex Hook. f.)

*Agrostis petelotii* (Hitchc.) Noltie, Edinburgh J. Bot. 56(3): 386–387. 1999

*Calamagrostis petelotii* (Hitchc.) Govaerts, World Checkl. Seed Pl. 3(1): 11. 1999

*Deyeuxia petelotii* (Hitchc.) S.M. Phillips & W.L. Chen, Novon 13(3): 319. 2003

*Aniselytron petelotii* (Hitchc.) Soják, Cas. Nar. Muz. Praze, Rada Prir. 148(3–4): 202. 1980

*Neoaulacolepis petelotii* (Hitchc.) Rauschert, Taxon 31(3): 561. 1982

*Deyeuxia abnormis* Hook. f., Fl. Brit. India (J.D. Hooker). 7(22): 268. 1896. Syntypes: Sikkim Himalaya: Lachong Valley, alt. 9000 ft.; *J.D. Hooker s.n.* 5000–5500 ft.; *Griffith s.n. *

*Agrostis continentalis* Hand.-Mazz., Symb. Sin. 7(5): 1297, pl. 40, f. 2. 1936

*Anisachne gracilis* Keng, J. Wash. Acad. Sci. 48(4): 117–118, f. 2. 1958

*Deyeuxia borii* Bhattacharya & S.K. Jain, Bull. Bot. Surv. India 25(1–4): 208. 1985 (“1983”), nom. superfl. & illeg.

**Distribution.** Meghalaya, Nagaland [Bhutan, China, Nepal].

**Habit.** Perennial.

**Remarks.** Record for Nagaland fide Naithani (1990). See Paszko and Ma (2011) for discussion of *Deyeuxia abnormis* and its synonyms.

**365. **Calamagrostis emodensis** Griseb., Nachr. Königl. Ges. Wiss. Georg-Augusts-Univ. 80. 1868 **

**Type. **India: Garhwal, alt. 9000–15400 ft., Sikkim, 6000–9000 ft.; *J.D. Hooker s.n.*

**Distribution.** Jammu and Kashmir, Uttar Pradesh, Uttarakhand [Bhutan, China, Pakistan].

**Habit.** Perennial.

**366. **Calamagrostis epigejos** (L.) Roth, Tent. Fl. Germ. 1: 34. 1788 **

*Arundo epigejos* L., Sp. Pl. 1: 81. 1753. Type: “Habitat in Europae collibus aridis.” Lectotype: *Herb. Linn. No. 97.11* (LINN). LT designated by Hubbard in Milne-Redhead and Polhill (ed.), Fl. Trop. E. Africa, Gramineae 1: 102. 1970.

*Calamagrostis macrolepis* Litv., Bot. Mater. Gerb. Glavn. Bot. Sada RSFSR 2: 125. 1921. Type: Turkestania, Dominium Buchara, Schugnan District, Anderob Province; *S. Korschinsky s.n.*

*Calamagrostis epigejos* (L.) Roth ssp. *macrolepis* (Litv.) Tzvelev

*Calamagrostis gigantea* Roshev., Izv. Bot. Sada Akad. Nauk S.S.S.R*.* 30: 294. 1932, nom. illeg. hom., non Nutt., 1835. Type: Province Semiretschensk, distr. Lepsinsk in parties ad ripam lacus Alakul prope vicum Ksyl- agatch; July 10, 1928; *N. Pavlov 671*, based on *Calamagrostisepigeios* var. *gigantea* Roshev.

**Distribution.** Jammu and Kashmir, Sikkim, Uttar Pradesh, West Bengal [China].

**Remarks.** Records for Sikkim, Uttar Pradesh, and West Bengal fide Naithani (1990).

**367. **Calamagrostis gamblei** Paszko, Polish Bot. J. 57: 327, 2012 **

**Type. **Uttarakhand prov., [Dehradun distr.], Jaunsar, Mundali, 7000 ft; Oct 1894, *J.S. Gamble 25249* (K; IT: CAL).

**Distribution.** Uttarakhand.

**Habit.** Perennial.

**Remarks.** See discussion in Paszko (2012a).

**368. **Calamagrostis garhwalensis** C.E. Hubb. & Bor, Indian Forester 68(7): 355. 1942 **

**Type. **Himalaya, Kedarnath, alt. 10000–11000 ft.; October 19, 1938; *C.E. Hubbard & Bor 8945? *(DD).

**Distribution.** Himachal Pradesh, Uttar Pradesh, Uttarakhand [Nepal, Pakistan].

**Habit.** Perennial.

**Remarks. **Records from Himachal Pradesh, Uttar Pradesh, and Nepal fide Naithani (1990).

**369. **Calamagrostis holciformis** Jaub. & Spach, Ill. Pl. Orient. 4: 61. t. 340. 1851 **

*Deyeuxia holciformis* (Jaub. & Spach) Bor, Grasses Burma, Ceylon, India and Pakistan 398. 1960

*Deyeuxia compacta* Munro ex Hook. f., Fl. Brit. India (J.D. Hooker). 7(22): 267. 1896. Type: Western Tibet; *Jacquemont s.n.* Nubra Valley, alt. 14000–16000 ft.; *Thomson s.n*. Kumaon, alt. 15000 ft.; *R. Strachey& J.E. Winterbottom Deyeuxia no. 1*

*Calamagrostis compacta* (Munro) Hack. ex Paulsen, Vidensk. Meddel. Dansk Naturhist. Foren. Kjobenhavn 167. 1903

*Calamagrostis tianschanica* Rupr., Sert. Tianschan. 34. 1869. Type: Not given.

**Type.** “In humidis altissimis Emodi Thibetani legit Jacquemont August auni 1830.” (P).

**Distribution.** Jammu and Kashmir [Afghanistan, Pakistan].

**Habit.** Perennial.

**370. **Calamagrostis kashmiriana** (Bor) Govaerts, World Checklist of Seed Plants 3(1): 10. 1999 **

*Deyeuxia kashmiriana* Bor, Kew Bull. 1954: 558. 1955

*Deyeuxia hackelii* Bor, Kew Bull. 1954: 497. 1954, nom. later hom., non (Lillo ex Stuck.) Parodi, 1953. Type: India: Kashmir, Astor Valley, above Doian, alt. 10500- 11600 ft.; August 14, 1892; *J.F. Duthie 12454* (K).

**Distribution.** Jammu and Kashmir.

**Habit.** Perennial.

**371. **Calamagrostis lahulensis** G. Singh, Taxon 33(1): 94. 1984 **

*Deyeuxia pulchella* Hook. f., Fl. Brit. India (J.D. Hooker). 7(22): 268. 1896. Type: Himalaya: Kumaon and Garhwal, alt. 10000–15000 ft., Sikkim, alt. 14000–16000 ft.; *J.D. Hooker Deyeuxia no. 10*

*Calamagrostis pulchella* Griseb., Nachr. Königl. Ges. Wiss. Georg-Augusts-Univ. 78. 1868, nom. illeg., non Saut. ex Rchb., 1830

**Distribution.** Himachal Pradesh, Jammu and Kashmir, Sikkim, Uttar Pradesh, Uttarakhand [Bhutan, China, Nepal].

**Habit.** Perennial.

**372. **Calamagrostis nagarum** (Bor) G. Singh, Taxon 33(1): 94. 1984 **

*Deyeuxia nagarum* Bor, Indian Forest Rec., Bot. n. s. 1(3): 69. 1938. Type: India: Assam, Naga Hills, Japvo, alt. 9500 ft.; *N.L. Bor s.n.* (K).

**Distribution.** Assam, Nagaland.

**Habit.** Perennial.

**Remarks.** Record from Nagaland fide Naithani (1990).

**373. **Calamagrostis nivicola** (Hook. f.) Hand.-Mazz., Symbolae Sinicae 7(5): 1299. 1936 **

*Deyeuxia nivicola* Hook. f., Fl. Brit. India (J.D. Hooker). 7(22): 267. 1896. Type: India: Sikkim, Momay, 15000 ft.; *J.D. Hooker & Thomson Deyeuxia no. 4*

**Distribution.** Sikkim [Bhutan, China, Nepal].

**Habit. **Perennial.

**374. **Calamagrostis pseudophragmites** (Haller f.) Koeler, Descr. Gram. (Koeler) 106. 1802 var. **pseudophragmites

*Arundo pseudophragmites* Haller f., Arch. Bot. (Leipzig) 1, 2: 11. 1797. Type: “ubi vicina Saxifragae mutatae habitabat.”

*Arundo littorea* Schrad., Fl. Germ. (Schrader) 1: 212. t. 4. f. 2. 1806 (as “litorea”). Type: [Austria]: In arenosis litoralis austriaci litoribus. *Wulfen s.n.* [Austria]:et similibus locis in insulis Danubialibus; *Flugge s.n.*

*Calamagrostis littorea* (Schrad.) P. Beauv., Ess. Agrostogr. 15: 157. 1812

*Calamagrostis laxa* Host, Icon. Descr. Gram. Austriac. 4: 25. t. 43. 1809. Type: “In arenosis, praecipue ad Danubii, aliorumque fluviorum ripas.”

*Calamagrostis nepalensis* Nees ex Steud., Syn. Pl. Glumac. 1: 193. 1854. Type: Nepal; *Royle 288*

*Calamagrostis onoei* Franch. & Sav., Enum. Pl. Jap. 2: 598. 1879. Syntypes: Japan: in provincial Senano; *Dr. Rein s.n.* Sine loci indicatione e botanico japonensi Ono primum habuit; *Dr. Savatier s.n.*

*Calamagrostis lanceolata* sensu Aitch., J. Linn. Soc., Bot. 18: 107. 1880, non Roth, 1788

**Distribution.** Himachal Pradesh, Jammu and Kashmir [Bhutan, China, Nepal, Pakistan].

**Habit.** Perennial.

**375. **Calamagrostis pseudophragmites** (Hall.f.) Koeler ssp. **tartarica** (Hook. f.) Tzvelev, Novosti Sist. Vyssh. Rast. 2: 42. 1965 **

*Calamagrostis littorea* (Schrad.) P. Beauv. var. *tartarica *Hook. f., Fl. Brit. India (J.D. Hooker). 7(22): 261. 1896. Type: Western Tibet; *J.D. Hooker & Thomson s.n.*

*Calamagrostis pseudophragmites* (Hall.f.) Koeler var. *tartarica* (Hook. f.) Bor, Grasses Burma, Ceylon, India and Pakistan 396. 1960

*Calamagrostis hedinii* Pilg., S. Tibet, Bot. 6(3): 93. 1922

**Distribution.** Himachal Pradesh, Jammu and Kashmir, Uttar Pradesh [Russia].

**Habit. **Perennial.

**376. **Calamagrostis scabrescens** Griseb., Nachr. Königl. Ges. Wiss. Georg-Augusts-Univ. 79. 1868 var. **scabrescens

*Deyeuxia scabrescens* (Griseb.) Munro ex Duthie in Atkins., Bot. Himalayan Distr. N. W. Prov. 623. 1882

*Calamagrostis filiformis* Griseb., Nachr. Königl. Ges. Wiss. Georg-Augusts-Univ. 79. 1868. Type: India: “’H. or Sikkim 11–12000’, H.[Hooker]: D[eyeuxia] nr. 3”

*Deyeuxia filiformis* (Griseb.) Hook. f., Fl. Brit. India (J.D. Hooker). 7(22): 268. 1896. Type: India: Sikkim, Lachen Valley, alt. 10000–12000 ft.; *J.D. Hooker s.n.*

*Calamagrostis scabrescens* Griseb. var. *humilis* Griseb., Nachr. Königl. Ges. Wiss. Georg-Augusts-Univ. 79. 1868. Type: India: Sikkim, Lachen Valley, alt. 10000–12000 ft.; *J.D. Hooker & Thomson Deyeuxia no. 2*

*Deyeuxia scabrescens* (Griseb.) Munro ex Duthie var. *humilis* (Griseb.) Hook. f., Fl. Brit. India (J.D. Hooker). 7(22): 268. 1896

*Deyeuxia scabrescens* (Griseb.) Munro ex C.B. Clarke, J. Linn. Soc., Bot. 25: 89, t. 39. 1889, non (Griseb.) Munro ex Duthie, 1882, isonym

**Type.** India: Sikkim, Lachen Valley, 10000–12000 ft.; *J.D. Hooker s.n.*

**Distribution.** Assam, Jammu and Kashmir, Sikkim, Manipur, Nagaland, Uttar Pradesh [Bhutan, China, Myanmar, Nepal, Pakistan].

**Habit.** Perennial.

**377. **Calamagrostis scabrescens** Griseb. var. **elatior** Griseb., Nachr. Königl. Ges. Wiss. Georg-Augusts-Univ. 79. 1868 **

*Deyeuxia elatior* (Griseb.) Hook. f., Fl. Brit. India (J.D. Hooker). 7(22): 266. 1896

**Type. **India: Khasia Hills, alt. 5000–5500 ft.; *J.D. Hooker Deyuxia no. 9.*

**Distribution.** Meghalaya.

**Habit.** Perennial.

**378. **Calamagrostis simlensis** (Bor) G. Singh, Taxon 33(1): 94. 1984 **

*Deyeuxia simlensis* Bor, Indian Forest Rec., Bot. n. s. 3: 149. 1941. Type: India: Simla; *D.M. Strong s.n. *(DD).

**Distribution.** Himachal Pradesh.

**Habit.** Perennial.

**Remarks.** Record for Himachal Pradesh fide Naithani (1990).

**379. **Calamagrostis stoliczkae** Hook. f., Fl. Brit. India (J.D. Hooker). 7(22): 262. 1896 **

**Type.** Western Tibet: Zanskar, on the Pensi-la, alt. 12000–15000 ft.; *Stoliczka s.n.*

**Distribution.** Jammu and Kashmir, Uttar Pradesh [China]

**Habit.** Perennial.

**380. **Calamagrostis tibetica** (Bor) Tzvelev, Rast. Tsentral. Azii 4: 86. 1968 **

*Deyeuxia tibetica* Bor, Kew Bull. 1949: 66. 1949. Type: India: Sikkim, Chakalunga, alt. 16000 ft.; June 1. 1915; *Rohmoo Lepcha s.n.* (K).

**Distribution.** Sikkim [China].

**Habit.** Perennial.

***Castellia* Tineo, Pl. Rar. Sicil. 2: 17. 1846**

**Type. ***Castellia tuberculata* Tineo.

**381. **Castellia tuberculosa** (Moris) Bor, Indian Forester 74(3): 90. 1948 **

*Catapodium tuberculosum* Moris, Atti Riunione Sci. Ital. 481. 1841. Type: “Appartiene questa specie alla Sardegna occidentale, e vi fiorisce in April e in Maggio.” Italy: South Sardinia; *Moris s.n.*

*Desmazeria tuberculosa* (Moris) Batt. & Trab., Fl. Alger (Battandier & Trabut) 100. 1884

*Festuca tuberculosa* (Moris) Coss. & Durieu., Expl. Sci. Algerie 2: 189, t. 14. 1856

*Micropyrum tuberculosum* (Moris) Pilg., Bot. Jahrb. Syst. 74(4): 567. 1949

*Castellia tuberculata* Tineo, Pl. Rar. Sicil. 2: 18. 1846. Type: “In arenosis vulcanicis. Linosa.”

*Festuca tuberculata* (Tineo) Benth., J. Linn. Soc., Bot. 19: 128. 1881, isonym, non (Moris) Coss. & Durieu., 1856

**Distribution.** India [Pakistan].

**Habit.** Annual.

**Remarks.** Localities within India not recorded.

***Catabrosa* P. Beauv., Ess. Agrostogr. 97. 1812**

**Lectotype. ***Catabrosa aquatica* (L.) P. Beauv. (*Aira aquatica* L.) LT. designated by Nash in N.L. Britton et A. Brown, Ill. Fl. N. U.S. ed. 2. 1: 245. 1913; affirmed by Hitchcock, Bull. U.S.D.A. 772: 47. 1920.

**382. **Catabrosa aquatica** (L.) P. Beauv., Ess. Agrostogr. 97, 149. t. 19. f. 8. 1812 var. **aquatica

*Aira aquatica* L., Sp. Pl. 1: 64. 1753. Type: “Habitat in Europae pascuis aquosis.” Lectotype: *Herb. Linn. No. 85.6 *(LINN). LT designated by Sherif & Siddiqi in El-Gadi (ed.), Fl. Libya 145: 84. 1988.

*Colpodium aquaticum* (L.) Trin., Mém. Acad. Imp. Sci. St.-Pétersbourg, Sér. 6, Sci. Math. 1(4): 395. 1830

*Glyceria aquatica* (L.) J. Presl. & C. Presl., Fl. Čech. 25. 1819

*Poa airoides* Koeler, Descr. Gram. (Koeler) 194. 1802, nom. superfl & illeg. for *Aira aquatica* L.

**Distribution.** Jammu and Kashmir [Afghanistan, Pakistan].

**Habit.** Perennial.

**383. **Catabrosa aquatica** (L.) P. Beauv. var. **angusta** Stapf, Fl. Brit. India (J.D. Hooker). 7(22): 311. 1896 **

**Type.** Western Tibet, Lanak Pass, alt. 15000–16000 ft.; *Thomson s.n.*

**Distribution.** Jammu and Kashmir [China, Pakistan].

**Habit.** Perennial.

**384. **Catabrosa sikkimensis** Stapf, Fl. Brit. India (J.D. Hooker). 7(22): 311. 1896 **

**Type.** India: alpine Sikkim, alt. 17000 ft.; *Gammie s.n.*

**Distribution.** Jammu and Kashmir, Sikkim.

**Habit.** Presumed perennial.

**Remarks. **Taxonomic editor Soreng notes that Dickoré (1995) treats this as a synonym of *C. aquatica*.

***Catabrosella* (Tzvelev) Tzvelev, Bot. Zhurn. (Moscow & Leningrad) 50: 1320 (in obs.). 1965**

*Colpodium* subg. *Catabrosella* Tzvelev, Nov. Syst. Pl. Vasc. 1964: 12, 1964.

**Type. ***Catabrosella humilis* (M. Bieb.) Tzvelev (*Aira humilis* M. Bieb.).

**385. **Catabrosella himalaica** (Hook. f.) Tzvelev, Novosti Sist. Vyssh. Rast. 1966: 32. 1966 **

*Phippsia himalaica* Hook. f., Fl. Brit. India (J.D. Hooker). 7(22): 240. 1896. Type: Alpine Western Himalaya: Kumaon, Barji-Khang Pass, alt. 14500 ft.; *R. Strachey & J.E. Winterbottom s.n.* Garhwal, Duder Glacier, alt. 13000–14000 ft.; *Duthie s.n.*

*Catabrosa himalaica* (Hook. f.) Stapf, Fl. Brit. India (J.D. Hooker). 7(22): 311. 1896

*Colpodium himalaicum* (Hook. f.) Bor, Grasses Burma, Ceylon, India and Pakistan 529. 1960

**Distribution.** Jammu and Kashmir, Uttar Pradesh, Uttarakhand [Pakistan].

**Habit.** Perennial.

**Remarks.** Taxonomic editor Soreng notes that *Colpodium trollii* Pilg. was placed in *Catabrosella himalaica* by Alexeev (1988) with some doubt. However, the species was described as 3(4)-flowered, and as no lemma vestiture was mentioned, presumably the type (if it is extant) has glabrous lemmas, so it probably is not *C. himalaica*. This also casts doubt on the synonymy of *Catabrosa trollii* (Pilg.) R.R. Stewart. Neither name is included here.

***Catapodium* Link, Hortus Berol. 1: 44 (“Catopodium”), 380. 1827**

**Type. ***Catapodium loliaceum* (Huds.) Link (*Poa loliacea* Huds.).

**386. **Catapodium rigidum** (L.) C.E. Hubbard in Dony, Fl. Bedfordshire 437. 1953 **

*Poa rigida* L., Cent. Pl. I 5. 1755. Type: “Habitat in Gallia, Anglia.” Lectotype: “Gramen panicula multiplici” in Bauhin, Prodr. Theatri Bot. 6. 6. 1620. LT designated by Stace in Cafferty et al. (ed.) Taxon 49: 256. 2000.

*Desmazeria rigida* (L.) Tutin & Stace in Tutin et al., Fl. Eur. 5: 158. 1980.

*Festuca rigida* (L.) Kunth Révis. Gramin. 2: 392. 1831, non Roth, 1797

*Scleropoa rigida* (L.) Griseb., Sp. Fl. Rumel 2: 431. 1845

**Distribution.** Jammu and Kashmir. Possibly naturalized; native to Europe.

**Habit.** Annual.

**Remarks.** Record for India fide Naithani (1990).

***Cyathopus* Stapf, Hooker’s Icon. Pl. 24: sub t. 2395. 1895**

**Type. ***Cyathopus sikkimensis* Stapf.

**387. **Cyathopus sikkimensis** Stapf, Hooker’s Icon. Pl. 24: t. 2395. 1895 **

**Type.** India: North Sikkim, Lachoong Valley in woods, alt. 11000 ft.; *J.D. Hooker s.n.*

**Distribution.** Sikkim [Bhutan, China].

**Habit.** Perennial.

***Cynosurus* L., Sp. Pl. 1: 72. 1753**

**Lectotype. ***Cynosurus cristatus *L. (Designated by Nash in N.L. Britton & A. Brown, Ill. Fl. N. U.S. ed. 2. 1: 251. 1913; affirmed by Hitchcock, Bull. U.S.D.A. 772: 67. 1920).

**388. **Cynosurus cristatus** L., Sp. Pl. 1: 72. 1753 **

**Type.** “Habitat in Europa pratis.” Lectotype: *Herb. Linn. No. 91.1* (LINN). LT designated by Cope in Jarvis et al. (ed.), Regnum Veg. 127: 41. 1993

**Distribution.** Meghalaya [China].

**Habit.** Perennial.

**389. **Cynosurus echinatus** L., Sp. Pl. 1: 72. 1753 **

*Phalona echinata* (L.) Dumort., Observ. Gramin. Belg. 114. 1824 (“1823”)

**Type.** “Habitat in Europa australiori.” Lectotype: *Herb. A. van Royen, sheet no. 913.62–79* (L). LT designated by Veldkamp in Cafferty et al. (ed.), Taxon 49: 249. 2000.

**Distribution.** India [also in Africa, Europe, North America, South America]. Possibly naturalized.

**Habit.** Annual.

**Remarks.** Specific states in India not recorded.

***Dactylis* L., Sp. Pl. 1: 71. 1753**

**Lectotype. ***Dactylis glomerata* L. (“glomeratus”). (Designated by Nash in N.L. Britton et A. Brown, Ill. Fl. N. U.S. ed 2. 1: 251. 1913; affirmed by Hitchcock, Bull. U.S.D.A. 772: 67. 1920).

**390. **Dactylis glomerata** L., Sp. Pl. 1: 71. 1753 (as “glomeratus”) **

*Bromus glomeratus* (L.) Scop., Fl. Carniol., ed. 2. 1: 76. 1771

*Festuca glomerata* (L.) All., Fl. Pedem. 2: 252. 1785

*Trachypoa vulgaris* Bubani, Fl. Pyren. (Bubani) 4: 359. 1901, nom. superfl. & illeg.

**Type.** “Habitat in Europae cultis ruderatis.” Lectotype: *Herb. Linn. No. 90.3* (LINN). LT designated by Clayton in Milne-Redhead and Polhill (ed.), Fl. Trop. E. Africa, Gramineae 1: 43. 1970.

**Distribution.** Arunachal Pradesh, Himachal Pradesh, Jammu and Kashmir, Meghalaya, Punjab, Tamil Nadu, Uttar Pradesh, Uttarakhand, West Bengal [Bhutan, China, Nepal].

**Habit.** Perennial.

***Deschampsia* P. Beauv., Ess. Agrostogr. 91. 1812**

**Lectotype. ***Deschampsia cespitosa* (L.) P. Beauv. (“caespitosa”) (*Aira cespitosa* L.). LT designated by Nash in N.L. Britton & A. Brown, Ill. Fl. N. U.S. ed. 2., 1: 215. 1913; affirmed by Hitchcock, Bull. U.S.D.A. 772: 116. 1920.

**391. **Deschampsia cespitosa** (L.) P. Beauv., Ess. Agrostogr. 149, 160. 1812 (as “caespitosa”) **

*Aira cespitosa* L., Sp. Pl. 1: 64–65. 1753. Type: “Habitat in Europae partis cultis & fertilibus.” Lectotype: *Herb. Linn. No. 85.8* (LINN). LT designated by Clayton in Milne-Redhead and Polhill (ed.), Fl. Trop. E. Africa, Gramineae 1: 92. 1970.

*Agrostis cespitosa* (L.) Salisb., Prodr. Stirp. Chap. Allerton 25. 1796

**Distribution.** Jammu and Kashmir, Sikkim, Uttarakhand [Bhutan, China, Pakistan].

**Habit.** Perennial.

**392. **Deschampsia koelerioides** Regel, Bull. Soc. Imp. Naturalistes Moscou 41(2): 299. 1868 **

**Type.** “Eine ausgezeichnete neue Art, die in ihrer Tracht an eine Koeleria oder eine Hierochloe erinnert. Am Kok-djar im Thian-Shan bei 8000 Fuss Hohe vom H. v. Semenow auf Alpenwiesen gesammelt.” “cis- et Transsiliensibus”; 1857; *P.P. Semenov-Tjan-Schansky 1197* (LE).

**Distribution.** Jammu and Kashmir [Afghanistan, China, Pakistan].

**Habit. **Perennial.

***Festuca* L., Sp. Pl. 1: 73. 1753**

**Lectotype. ***Festuca ovina *L. LT designated by Nash in N.L. Britton and A. Brown, Ill. Fl. N. U.S. ed. 2., 1: 269. 1913; affirmed by Hitchcock, Bull. U.S.D.A. 772: 28. 1920.

**393. **Festuca alaica** Drobow, Trudy Bot. Muz. Imp. Akad. Nauk 16: 134, t. 17/5. 1916 **

**Type.** Turkestan: Fergana Prov., Skobelev District; *Margelan s.n.* “In pratis alpinis ad fl. Dugova, circa pag. Jordan, alt. 14000 ft. (*Drobov 303*).”

**Distribution.** Jammu and Kashmir [Afghanistan, China, Pakistan].

**Habit.** Perennial.

**Remarks.** Record for India fide Naithani (1990).

**394. **Festuca alatavica** (St.-Yves) Roshev., Fl. URSS. 2: 528. 1934 **

*Festuca rubra* L. ssp. *alatavica* St.-Yves, Candollea 3: 393. 1928. Type: Turkestan: Chaine de l’Alatau, Kokoirak, aux sources du Kabir;* Brotherus no. 784* (B). Kokbulak, aux sources du Naryn; *Brotherus s.n.* (B).

*Festuca altaica* auct. non Trin., 1829: Stapf, Fl. Brit. India (J.D. Hooker). 7(22): 351. 1896

**Distribution.** Jammu and Kashmir [China, Pakistan].

**Habit.** Perennial.

**395. **Festuca altaica** Trin., Fl. Altaic. [Ledebour]. 1: 109. 1829 **

**Type.** USSR: Siberia “In summa alpe ad fontem fl. Acjulac rarissima”; *Bunge s.n.*

**Distribution.** India (Northwest Himalaya) [China, Pakistan].

**Habit.** Perennial.

**Remarks.** States within India not recorded.

**396. **Festuca asthenica** Hook. f., Fl. Brit. India (J.D. Hooker). 7(22): 354. 1896 **

**Type.** India: Kashmir, alt. 7000–9000 ft.; *C.B. Clarke s.n., Duthie s.n.*

**Distribution.** Jammu and Kashmir [Pakistan].

**Habit.** Perennial.

**397. **Festuca bromoides** L., Sp. Pl. 1: 75. 1753 **

*Vulpia bromoides* (L.) Gray, Nat. Arr. Brit. Pl. 2: 124. 1821

**Type.** “Habitat in Anglia, Gallia.” Lectotype: Herb. A. van Royen No. 912.356–219 (L). LT designated by Stace & Jarvis in Bot. J. Linn. Soc. 91: 436. 1985.

**Distribution.** Tamil Nadu [Bhutan, Sri Lanka].

**Habit.** Annual.

**398. **Festuca coelestis** (St.-Yves) Krecz. & Bobrov, Fl. URSS 2: 514. t. 50. f. 12. 1934 **

*Festuca ovina* L. ssp. *coelestis* St.-Yves, Candollea 3: 376. 1928. Type: Thian-schan Mount celestes, vallee de Djoukoutchiak, alt. 6300–7200 ft.; *Brocherel 392 *(Herb. Deless.) and many syntypes.

**Distribution.** Jammu and Kashmir [China, Pakistan].

**Habit.** Perennial.

**Remarks.** Record for India fide Naithani (1990).

**399. **Festuca cumminsii** Stapf, Fl. Brit. India (J.D. Hooker). 7(22): 349. 1896 **

**Syntypes.** India: Sikkim, alt. 11000–12000 ft.; *J.D. Hooker s.n.* Bhutan; *Cummins s.n.*

**Distribution.** Jammu and Kashmir, Sikkim [Bhutan, Pakistan].

**Habit.** Perennial.

**400. **Festuca danthonii** Asch. & Graebn., Syn. Mitteleur. Fl. 2: 551. 1901 **

*Festuca ciliata* Danthoine ex Lam. & DC. , 1805, Flore Française. Troisième Édition 3: 55. 1805, non. Gouan, 1762. Type. France: près Montepellier, et dans les îles sablonneuses de la Durance

*Vulpia ciliata* Dumort., Observ. Gramin. Belg. 100. 1824. Type: Yugoslavia: Dalmatiae arenosis, herbidis ad mare: copiosae in insula Vergada, Vaena.

**Distribution.** Himachal Pradesh, Jammu and Kashmir [Pakistan].

**Habit.** Annual.

**Remarks.** Records for India fide Naithani (1990).

**401. **Festuca debilis** (Stapf) E.B. Alexeev, Byull. Moskovsk. Obshch. Isp. Prir. Otd. Biol. 83(4): 109. 1978 **

*Festuca kashmiriana* Stapf var. *debilis* Stapf, Fl. Brit. India (J.D. Hooker). 7(22): 351. 1896. Lectotype: India: Kashmir, Lidder Valley, above Kainmul, alt. 11000–12000 ft.; July 21, 1893; *J.F. Duthie 13139* (K). LT designated by E.B. Alexeev in Byull. Moskovsk. Obshch. Isp. Prir. Otd. Biol. 83(4): 109. 1978.

**Distribution.** Jammu and Kashmir [Pakistan].

**Habit.** Perennial.

**402. **Festuca hartmannii** (Markgr.-Dann.) E.B.Alexeev, Byull. Moskovsk. Obshch. Isp. Prir. Otd. Biol. 83(4): 121. 1978 **

*Festuca ovina* L. var. *hartmannii* Markgr.-Dann., Bot. Jahrb. Syst. 85(3): 376. 1966. Type: Karakorum Central: Baintha, alt. 12800 ft.; July, 1962; *H. Hartmann s.n.*

**Distribution.** Jammu and Kashmir [Pakistan].

**Habit.** Perennial.

**Remarks.** Record for India fide Naithani (1990).

**403. **Festuca kashmiriana** Stapf, Fl. Brit. India (J.D. Hooker). 7(22): 351. 1896 **

*Festuca kashmiriana* Stapf var. *ligulata* Stapf, Fl. Brit. India (J.D. Hooker). 7(22): 351. 1896. Type: India: Kashmir, alt. 11000–13000 ft.; *Duthie s.n.*

*Festuca rubra* L. ssp. *kashmiriana* (Stapf) St.-Yves, Candollea 3: 395. 1928

**Type.** Temperate and Alpine Himalaya, from Kashmir to Kumaon, alt. 8000–14000 ft.; *Royle s.n.*

**Distribution.** Jammu and Kashmir, Uttar Pradesh [China, Pakistan].

**Habit.** Perennial.

**404. **Festuca leptopogon** Stapf, Fl. Brit. India (J.D. Hooker). 7(22): 354. 1896 **

*Festuca subulata* Trin. var. *leptopogon* (Stapf) St.-Yves, Candollea 3: 450–451. 1928

**Type.** India: Sikkim Himalaya, alt. 8000–11000 ft.; *J.D. Hooker s.n.* Khasia Hills, alt. 5000–6000 ft.; *J.D. Hooker & Thomson, C.B. Clarke s.n.*

**Distribution.** Assam, Meghalaya, Sikkim [Bhutan, China, Nepal].

**Habit.** Perennial.

**405. **Festuca levingei** Stapf, Fl. Brit. India (J.D. Hooker). 7(22): 352. 1896 **

**Type.** India: Kashmir; *Levinge 27394 *(K).

**Distribution.** Jammu and Kashmir [Pakistan].

**Habit.** Perennial.

**406. **Festuca lucida** Stapf, Fl. Brit. India (J.D. Hooker). 7(22): 355. 1896 **

**Type.** India: NW Himalaya, Jaunsar, Karambar Peak, alt. 9000 ft.; May 1, 1894; *J.F. Duthie 14481* (LE).

**Distribution. **Himachal Pradesh, Jammu and Kashmir, Uttar Pradesh, Uttarakhand.

**Habit.** Perennial.

**407. **Festuca maritima** L., Sp. Pl. 1: 75. 1753 **

*Nardurus maritimus* (L.) Murb., Acta Univ. Lund. 4: 25. 1900

*Vulpia unilateralis* (L.) Stace, Bot. J. Linn. Soc. 76: 350. 1978

*Agropyron unilaterale* (L.) P. Beauv., Ess. Agrostogr. 102. 1812, alternative name to *Brachypodium unilaterale*

*Brachypodium unilaterale* (L.) P. Beauv., Ess. Agrostogr. 155. 1812, alternative name to Agropyron* unilaterale*

*Nardurus unilateralis* (L.) Boiss., Voy. Bot. Espagne 2: 667. 1844

*Triticum unilaterale* L., Mant. Pl. 35. 1767. Type: “Habitat in maritimis Italiae, Galliae australis.” Lectotype: Herb. Linn. No. 104.15 (LINN). LT designated by Stace & Jarvis in Bot. J. Linn. Soc. 91: 442. 1985

*Festuca tenuifolia* Schrad., Fl. Germ. (Schrader) 1: 345. 1806, non Sibth, 1794. Type: “In iftriae herbidis.”

**Type.** “Habitat in Hispania.” Lectotype: Herb. Linn. No. 104.17 (LINN). LT designated by Hubbard in Proc. Linn. Soc. London 148: 110. 1936.

**Distribution.** India (Northwest Himalaya) [Asia: Saudi Arabia; Africa; Europe: Germany, Italy, Spain].

**Habit.** Annual.

**Remarks.** States in India not recorded.

**408. **Festuca megalura** Nutt., Proc. Acad. Nat. Sci. Philadelphia 4: 24. 1848; J. Acad. Nat. Sci. Philadelphia ser. 2, 1: 188. 1848 **

*Vulpia megalura* (Nutt.) Rydb., Bull. Torrey Bot. Club 36: 538. 1909

*Vulpia myuros *var*. megalura *(Nutt.) Auquier, Bulletin du Jardin Botanique National de Belgique 47(1–2): 123. 1977

*Festuca commutata* Steud., Syn. Pl. Glumac. 1: 304. 1854. Type: “Chili, Hrbr Bertero nr. 274 et 1002 ex parte, mixta cum praecedente.”

**Type.** USA: California, Hab. Santa Barbara, Upper California; *Wm. Gambel s.n.* (PH).

**Distribution.** Tamil Nadu. Naturalized; native to western North and South America.

**Habit.** Annual.

**409. **Festuca modesta** Steud., Syn. Pl. Glumac. 1: 316. 1854 **

*Schedonorus modestus* Nees ex Steud., Syn. Pl. Glumac. 1: 316. 1854, nom. nud. pro. syn.

**Type.** Nepal; *Royle Herb. 161.*

**Distribution.** Jammu and Kashmir, Uttar Pradesh [China, Nepal].

**Habit.** Perennial.

**Remarks.** Taxonomic editor Soreng notes that this taxon belongs to the broad-leaved lineage of *Festuca* s.l., and possibly should be transferred to *Lolium*.

**410. **Festuca muralis** Kunth, Syn. Pl. (Kunth) 1: 218. 1822 **

*Vulpia muralis* (Kunth) Nees, Linnaea 19: 694. 1847

*Vulpia muralis* (Kunth) Henrard, Blumea 2: 319. 1937

**Type.** “Crescit in muris, quibus horti circumsepti sunt, prope Conocoto Quitensium, alt. 1350 hex., Humboldt & Bonpland s.n.” Ecuador; *F.W.H. Humboldt & A.J.A. Bonpland s.n.*(P).

**Distribution.** India, presumably naturalized; native to South America.

**Habit.** Annual.

**Remarks.** States in India not recorded.

**411. **Festuca myuros** L., Sp. Pl. 1: 74. 1753 **

*Vulpia myuros* (L.) C.C. Gmel., Fl. Bad. 1: 8. 1805

*Zerna myuros* Panz. ex Jacks., Index Kew. 2: 1249. 1895, pro syn.

*Avena muralis* Salisb., Prodr. Stirp. Chap. Allerton 22. 1796, nom. superfl. & illeg.

**Type.** “Habitat in Anglia, Italia.” Lectotype: Herb. A. van Royen No. 912.356–218 (L). LT designated by Stace & Jarvis in Bot. J. Linn. Soc. 91: 436. 1985.

**Distribution.** Arunachal Pradesh, Assam, Himachal Pradesh, Jammu and Kashmir, Karnataka, Tamil Nadu, Uttar Pradesh [China, Pakistan].

**Habit.** Annual.

**412. **Festuca nandadevica** Hajra, Indian J. Forest. 6(1): 79–80. f. 1. 1983 **

**Type.** India: Uttar Pradesh, Chamoli, Nandadevi National Park, Deodi-Ramani; *P.K. Hajra 73285A* (CAL).

**Distribution.** Uttar Pradesh, Uttarakhand.

**Habit.** Perennial.

**Remarks.** Record for Uttar Pradesh fide Naithani (1990).

**413. **Festuca nitidula** Stapf, Fl. Brit. India (J.D. Hooker). 7(22): 350. 1896 **

**Syntypes.** Western Tibet, Nubra, alt. 12000–14000 ft.; *Thomson s.n.* India: North of Kumaon, alt. 15000 ft.; *R. Strachey & J.E. Winterbottom s.n.*

**Distribution.** Jammu and Kashmir, Uttar Pradesh, Uttarakhand [China, Nepal, Pakistan].

**Habit.** Perennial.

**414. **Festuca octoflora** Walter, Fl. Carol. 81. 1788 **

*Vulpia octoflora* (Walter) Rydb., Bull. Torrey Bot. Club 36: 538. 1909

*Festuca tenella* Willd., Sp. Pl., ed. 4 [Willdenow] 1(1): 419. 1797. Type: “Habitat in America boreali.”

**Type.** USA: South Carolina, Santee Valley; *Walter s.n.* (BM?). According to Hitchcock, this specimen is not at BM; see Lonard and Gould, Madroño 22: 221. 1974.

**Distribution. **Uttar Pradesh. Naturalized. Native to North America.

**Habit.** Annual.

**Remarks.** Record for India fide Naithani (1990).

**415. **Festuca ovina** L., Sp. Pl. 1: 73–74. 1753 **

**Type. **“Habitat in Alpibus Lapponicae, Helvetiae, Scotiae.” Lectotype: *Herb. Linn. No. 92.1; Lapland Herb. No. 55* (LINN). LT designated by Kerguélen in Lejeunia, n.s. 75: 150. 1975.

**Distribution.** Sikkim, Tamil Nadu, Uttar Pradesh [China, Nepal].

**Habit.** Perennial.

**416. **Festuca pamirica** Tzvelev, Bot. Mater. Gerb. Bot. Inst. Komarova Akad. Nauk S.S.S.R. 20: 422. 1960 **

*Festuca rubra* L. ssp. *schlagintweitii* St.- Yves, Candollea 3: 389. 1928, p.p.

**Type.** Tadzhikistania, Pamir Boreali-occidentalis, in parties lapidosis in fontibus fluminis tachta-Korum prope trajectum Tachtakorum, alt. 13000 ft.; July 30, 1958; *N. Tzvelev. 1008* (LE).

**Distribution.** Jammu and Kashmir [China, Pakistan].

**Habit.** Perennial.

**Remarks.** Record for India fide Naithani (1990).

**417. **Festuca parvigluma** Steud., Syn. Pl. Glumac. 1: 305. 1854 **

**Type.** “Ex Hrbo. Mus. Lugd. Batav. Japonia.”

**Distribution.** Sikkim [China, Nepal].

**Habit.** Perennial.

**Remarks.** Records for Sikkim and Nepal fide Naithani (1990).

**418. **Festuca polycolea** Stapf, Fl. Brit. India (J.D. Hooker). 7(22): 349. 1896 var. **polycolea

*Festuca ovina* L. ssp. *polycolea* (Stapf) St.-Yves, Candollea 3: 373. 1928

**Syntypes.** Nepal; *Wallich s.n.* India: Sikkim, alt. 12000–16000 ft.; *J.D. Hooker &c.*

**Distribution.** Sikkim, Uttar Pradesh, Uttarakhand [Bhutan, Nepal].

**Habit.** Perennial.

**419. **Festuca polycolea** Stapf var. **brevia** Stapf, Fl. Brit. India (J.D. Hooker). 7(22): 350. 1896 **

**Type.** India: Sikkim Himalaya, alt. 11000–13000 ft.

**Distribution.** Sikkim, Uttar Pradesh.

**Habit.** Perennial.

**420. **Festuca rubra** L., Sp. Pl. 1: 74. 1753 var. **rubra

**Type.** “Habitat in Europae sterilibus siccis.” Lectotype: *Linnaeus s.n.* (GB). LT designated by Jarvis et al. in Watsonia 16: 302. pl. 2B. 1987.

**Distribution.** Jammu and Kashmir, Himachal Pradesh, Meghalaya, Uttar Pradesh,

Uttarakhand [China].

**Habit.** Perennial.

**421. **Festuca rubra** L. ssp. **arctica** (Hack.) Govor., Fl. Ural. 127. 1937 **

*Festuca rubra* f. *arctica* Hack., Monogr. Festuc. Eur.: 138. 1882. Type not cited.

*Festuca richardsonii* Hook., Fl. Bor.-Amer. 2: 250, t. 230. 1840. Type: Arctic Sea-coast. *Dr. Richardson s.n., s.d.*

**Distribution.** Jammu and Kashmir [China, Pakistan].

**Habit.** Perennial.

**Remarks.** Record for India fide Naithani (1990).

**422. **Festuca rubra** L. ssp. **clarkei** (Stapf) St.-Yves, Candollea 3: 398. 1928 **

*Festuca rubra* L. var. *clarkei *Stapf, Fl. Brit. India (J.D. Hooker). 7(22): 353. 1896. Syntypes: India: Kangara & Dalhousie, alt. 7000–8000 ft.; *C.B. Clarke s.n.* Khasia Hills, alt. 4000–6000 ft.; *C.B. Clarke s.n.*

*Festuca rubra* L. var. *stapfiana* St.-Yves, Candollea 3: 398. 1928

**Distribution.** Assam, Himachal Pradesh, Jammu and Kashmir, Meghalaya [Bhutan, China, Pakistan].

**Habit.** Perennial.

**Remarks.** Record for Meghalaya fide Naithani (1990).

**423. **Festuca rubra** L. var. **villosa** Mert. & W.D.J. Koch, Deutschl. Fl. (Roehling), ed. 3. 1: 654. 1823 **

**Type.** [Europe], Not Spenner, 1825.

**Distribution.** Himachal Pradesh. Also in Europe.

**Habit.** Perennial.

**Remarks.** Record for India fide Naithani (1990).

**424. **Festuca sanjappae** Chandra Sek. & S.K. Srivast., J. Jap. Bot. 80(2): 72–75. f. 1. 2005 **

**Type.** India: Himachal Pradesh, Pin Valley National Park, Chhohem, 12800–13100 ft.; *K. Chandra Sekar 103271* (CAL).

**Distribution. **Himachal Pradesh.

**Habit.** Perennial.

**425. **Festuca simlensis** (Stapf) E.B. Alexeev, Byull. Moskovsk. Obshch. Isp. Prir. Otd. Biol. 83(4): 110. 1978 **

*Festuca kashmiriana* Stapf var. *simlensis* Stapf, Fl. Brit. India (J.D. Hooker). 7(22): 351. 1896. Lectotype: India: Simla, alt. 7000–8000 ft.; *J.D. Hooker & Thomson s.n.* (K). LT designated by E.B. Alexeev in Byull. Moskovsk. Obshch. Isp. Prir. Otd. Biol. 83(4): 110. 1978.

**Distribution.** Himachal Pradesh [Pakistan].

**Habit.** Perennial.

**426. **Festuca stapfii** E.B. Alexeev, Byull. Moskovsk. Obshch. Isp. Prir. Otd. Biol. 83(4): 115 1978 **

*Festuca undata* Stapf var. *aristata* Stapf, Fl. Brit. India (J.D. Hooker). 7(22): 351. 1896. Type: India: Sikkim, alt. 11,000 ft.; *J.D. Hooker & T. Thomson s.n.*

**Distribution.** Sikkim, Tamil Nadu, West Bengal [Bhutan, China, Nepal].

**Habit.** Perennial.

**427. **Festuca tibetica** (Stapf) E.B. Alexeev, Byull. Moskovsk. Obshch. Isp. Prir. Otd. Biol. 83(4): 118. 1978 **

*Festuca valesiaca* Schlecht. ex Gaudin var. *tibetica* Stapf, Fl. Brit. India (J.D. Hooker). 7(22): 349. 1896. Lectotype: Tibet Orientalis, region alpine, alt. 14000 ft.; *J.D. Hooker & T. Thomson s.n.* (K). LT designated by E.B. Alexeev in Byull. Moskovsk. Obshch. Isp. Prir. Otd. Biol. 83(4): 118. 1978

**Distribution.** Sikkim [Bhutan, China, Nepal, Pakistan].

**Habit.** Perennial.

**428. **Festuca undata** Stapf, Fl. Brit. India (J.D. Hooker). 7(22): 350. 1896 **

**Syntypes.** India: Sikkim, alt. 12000 ft.; *J.D. Hooker s.n.*, Herb. Ind. Or.; *J.D. Hooker & ThomsonBromusno. 16.*

**Distribution.** Sikkim, Tamil Nadu [China, Nepal].

**Habit.** Perennial.

**429. **Festuca valesiaca** Schlecht. ex Gaudin, Agrost. Helv. 1: 242. 1811 var. **valesiaca

*Festuca ovina* L. var. *valesiaca* (Schlecht. ex Gaudin) W.D.J. Koch, Syn. Fl. Germ. Helv. 812. 1837, *isonym*, non *Festuca ovina* L. var. *valesiaca* (Schleich. ex Gaudin) Link, Hort. Berol. [Link] 2: 267. 1833.

**Type.** “Habitat in locis arenosis Valesiae. Circa Branson copiose Perennis.”

**Distribution.** Assam, Himachal Pradesh, Jammu and Kashmir, Sikkim, Uttar Pradesh, Uttarakhand [China].

**Habit.** Perennial.

**430. **Festuca wallichiana** E.B. Alexeev, Byull. Moskovsk. Obshch. Isp. Prir., Otd. Biol. 83(4): 120. 1978 **

**Distribution.** Arunachal Pradesh [Bhutan, China, Nepal].

**Habit.** Perennial.

***Helictochloa* Romero Zarco, Candollea 66(1): 96. 2011**

**Type.***Helictochloa bromoides* (Gouan) Romero Zarco (*Avena bromoides* Gouan).

**431. **Helictochloa pratensis** (L.) Romero Zarco, Candollea 66(1): 103. 2011 **

*Avena pratensis* L., Sp. Pl. 1: 80. 1753. Type: “Habitat in Europae pascuis siccis apricis.” Lectotype: *Herb. Linn. No. 44.5 *(S). LT designated by Röser in Taxon 44: 397. 1995.

*Avena alpina* Sm., Trans. Linn. Soc. London 10: 335. 1811, non Honck., 1782? Type: “Discovered in 1807, on rocks upon the summits of the highest Mountains of Clova, Angusshire.”

*Heuffelia pratensis* (L.) Schur, Enum. Pl. Transsilv. 762. 1866

*Helictotrichon pratense* (L.) Pilg., Repert. Spec. Nov. Regni Veg. 45: 6. 1938

**Distribution.** Uttarakhand [Pakistan].

**Habit. **Perennial.

**Remarks.** Dr. Romero Zarco (pers. comm.) speculates that this species could have been introduced into India as a pasture grass.

***Holcus* L., Sp. Pl. 2: 1047. 1753, nom. cons.**

**Type. ***Holcus lanatus* L. (typ. cons.).

**432. **Holcus lanatus** L., Sp. Pl. 2: 1048. 1753 **

*Avena lanata* (L.) Koeler, Descr. Gram. (Koeler) 303. 1802

*Notholcus lanatus* (L.) Nash ex Hitchc. in Jepson, Fl. Calif. 1: 126. 1912

*Ginannia lanata* (L.) F.T. Hubb., Rhodora 18: 234. 1916, nom. superfl. & illeg. for *H. lanatus* L.

*Ginannia pubescens* Bubani, Fl. Pyren. (Bubani) 4: 321. 1901, nom. superfl. & illeg. for *H. lanatus* L.

*Aira holcus-lanatus* Vill., Hist. Pl. Dauphiné (Villars) 2: 87. 1787, nom. superfl. & illeg. for *H*. *lanatus* L.

*Avena pallida* Salisb., Prodr. Stirp. Chap. Allerton 24. 1796, nom. superfl. & illeg. for *H. lanatus* L.

**Type.** “Habitat in Europae pascuis arenosis.” Lectotype: *Herb. Linn. No. 1212.10* (LINN). LT designated by Cope in Jarvis et al. (ed.), Regnum Veg. 127: 54. 1993.

**Distribution.** Sikkim, West Bengal [China].

**Habit.** Perennial.

**433. **Holcus mollis** L., Syst. Nat., ed. 10. 2: 1305. 1759 **

*Aira mollis* (L.) Schreb., Spic. Fl. Lips. 51. 1771

*Avena mollis* (L.) Koeler, Descr. Gram. (Koeler) 300. 1802

*Avena sylvatica* Salisb., Prodr. Stirp. Chap. Allerton 24. 1796, nom. superfl. & illegit.

*Ginannia mollis* (L.) Bubani, Fl. Pyren. (Bubani) 4: 322. 1901

*Notholcus mollis* (L.) Hitchc., Amer. J. Bot. 2(6): 304. 1915

**Type.** “Habitat [in Europa: in Hallandia. [sic] Osbeck.] Sp. Pl., ed. 2. 2: 1485. 1763.” Lectotype: Herb. Linn. No. 1212.9 (LINN). LT designated by Cope in Cafferty et al. (ed.), Taxon 49: 251. 2000.

**Distribution.** Meghalaya [also in Europe, North America]. Naturalized. Native to Europe.

**Habit.** Perennial.

***Hyalopoa* (Tzvelev) Tzvelev, Bot. Zhurn. (Moscow & Leningrad) 50(9): 1320. 1965**

*Colpodium* subg. *Hyalopoa* Tzvelev, Nov. Syst. Pl. Vasc. 1964: 6. 1964.

**Type. ***H. pontica* (Balansa) Tzvelev (*Catabrosa pontica* Balansa).

**434. **Hyalopoa nutans** (Stapf) E.B. Alexeev ex Cope, Fl. Pakistan 143: 423. 1982 **

*Catabrosa nutans* Stapf, Fl. Brit. India (J.D. Hooker). 7(22): 312. 1896. Type: Alpine Himalaya: from Kashmir to Garhwal, alt. 10000–14000 ft.; *Jacquemont s.n.*

*Colpodium nutans* (Stapf) Bor, Fl. Iranica 70: 54. 1970

**Distribution.** Jammu and Kashmir [Pakistan].

**Habit.** Perennial.

***Koeleria* Pers., Syn. Pl. (Persoon) 1: 97. 1805**

**Lectotype.***Aira cristata* L. Designated by Hitchcock, Bull. U.S.D.A. 772: 107. 1920.

**Notes.** The generic limits of *Koeleria*, *Trisetum* and related genera have been the subject of much recent taxonomic work. See comments under *Trisetum*.

**435. **Koeleria argentea** Griseb., Nachr. Königl. Ges. Wiss. Georg-Augusts-Univ. 77. 1868 var. **argentea

*Koeleria litvinowii* ssp. *argentea* (Griseb.) S.M. Phillips & Z.L. Wu, Fl. China 22: 331 2006

**Type.** India: “T. Nubra, Ladak pr. Leh, Nari Khorsum.”

**Distribution.** Himachal Pradesh.

**Habit.** Perennial.

**436. **Koeleria argentea** Griseb. var. **nepalensis** Domin, Biblioth. Bot. 14(Heft. 65): 115. t. 7. f. 6. 1907 **

**Type.** “Herb. Schlaginweit from India and High Asia 1. Gen, Nro. Of Catal 4118 K. nepalensis Griseb. (Teste Grisebach) (1856).”

**Distribution.** Himachal Pradesh [Nepal].

**Habit.** Perennial.

**Remarks.** Record for India fide Naithani (1990).

**437. **Koeleria hookeri** Barberá, Quintanar, Soreng, & P.M. Peterson, Phytoneuron 2019–46: 4. 2019. Name changed to avoid confusion with **Koeleria clarkeana** Domin. **

*Avena clarkei* Hook. f., Fl. Brit. India (J.D. Hooker). 7(21): 278. 1896. Type: India: Kashmir, at Laka and Badrawur, alt. 10000–11000 ft. Chamba, Dalhousie, alt. 8700 ft.; *C.B. Clarke s.n.*

*Trisetum clarkei* (Hook. f.) R.R. Stewart, Brittonia 5(4): 431. 1945

**Distribution.** Jammu and Kashmir, Himachal Pradesh, Uttar Pradesh, Uttarakhand [Afghanistan, China, Nepal, Pakistan].

**Habit.** Perennial.

**438. **Koeleria litvinowii** Domin, Biblioth. Bot. 14(65): 116. 1907 **

**Distribution.** Jammu and Kashmir [Afghanistan, China].

**Habit.** Perennial.

**439. **Koeleria micans** (Hook. f.) Barberá, Quintanar, Soreng, & P.M. Peterson, Phytoneuron 2019–46: 5. 2019 **

*Avena micans* Hook. f., Fl. Brit. India (J.D. Hooker). 7(22): 279. 1896. Type: Western Himalaya: Garhwal, banks of the Ganges, alt. 8000–10000 ft.; *Duthie s.n.*

*Trisetum micans* (Hook. f.) Bor, Grasses Burma, Ceylon, India and Pakistan 448. 1960. Type: Teri Garhwal, alt. 8600- 9600 ft.; *J.F. Duthie 46*

**Distribution.** Uttar Pradesh, Uttarakhand.

**Habit. **Perennial.

**440. **Koeleria pyramidata** (Lam.) P. Beauv., Ess. Agrostogr. 84, 166, 175. 1812 **

*Poa pyramidata* Lam., Tabl. Encycl 1: 183. 1791

*Koeleria cristata* Pers., Syn. Pl. (Persoon) 1: 97. 1805, nom. illeg. superfl.; [Persoon included Lamarck’s older name as a variety (*K. cristata* var. *pyramidata* (Lam.) Pers.) within his new *Koeleria cristata* Pers. (non. L.; often erroneously cited as *K. cristata* (L.) Pers.)]

*Aira cristata* L., Sp. Pl. 1: 63. 1753, p.p. Type: “Habitat in Angliae, Galliae, Helvetiae siccioribus.” Lectotype: Herb. A. van Royen, sheet no. 913.62–99 (L). LT designated by Humphries in Cafferty et al. (ed.), Taxon 49: 244. 2000. Epitype: France. Jura, Bas-Bugey, Montagny de Ste Claire sur Brioguier, 400–500 m; 3 Jun 1929; *Briquet 6737* (BM). Epitype designated by Humphries, C.J. in Cafferty et al. (ed.), Taxon 49: 244. 2000.

*Airochloa cristata* (L.) Link, Hort. Berol. [Link] 1: 127. 1827

*Dactylis cristata* (L.) M. Bieb., Fl. Taur.-Caucas. 1: 67. 1808

*Festuca cristata* (L.) Vill., Hist. Pl. Dauphiné (Villars) 2: 93. 1787, non L. (1753)

*Poa nitida* Lam., Tabl. Encycl. 1: 182. 1791. Type: France: “Ex Galliae siccis & montosis Leer S.T. 5. f. 6.”

*Aira gracilis* Trin., Fund. Agrost. (Trinius) 144. 1820

*Airochloa gracilis* Link, Hort. Berol. [Link] 2: 276. 1833

*Koeleria gracilis* Pers., Syn. Pl. (Persoon) 1: 97. 1805, nom. superfl. & illeg.

*Koeleria cristata* Pers., Syn. Pl. (Persoon) 1: 97. 1805, nom. superfl. & illeg.

*Koeleria macrantha* (Ledeb.) Schult., Mant. 2 (Schultes) 345. 1824

*Aira macrantha* Ledeb., Mém. Acad. Imp. Sci. Saint Pétersbourg, Sér. 7, 5: 515–516. 1812. Type: “Hab. in jugo montium jablonnoi-Chrebet.”

*Koeleria cristata* auct. non (L.) Pers., 1805: Hook. f., Fl. Brit. India (J.D. Hooker). 7(22): 308. 1896

**Distribution.** Himachal Pradesh, Jammu and Kashmir, Rajasthan, Uttar Pradesh, Uttarakhand [China, Nepal, Pakistan].

**Habit. **Perennial.

**Remarks.** Taxonomic editor Soreng notes that according to the Linnaean typification website, the current name for *Aira cristata* L. is *Koeleria macrantha* (Ledeb.) Schult., Mant. 2 (Schultes) 2: 345. 1824. *Koeleria cristata* might not be made on the basis of *Aira cristata*. *Koeleria cristata* is treated as a synonym of *Koeleria macrantha* in the Flora of Pakistan, and according to Quintanar *Koeleria macrantha* is a taxonomic synonym of *K. pyramidata*. There is no morphological discontinuity among these taxa. Thus, our distribution information and synonymy reflect the merging of these taxa, with the understanding that (as above), *Koeleria cristata* might not really belong here. See also comments in IPNI (2020).

**441. **Koeleria spicata** (L.) Barberá & al. subsp. **virescens** (Regel) Barberá, A. Quintanar, Soreng & P.M. Peterson, Phytoneuron 2019–46: 9. 2019 **

*Avena flavescens *L. var. *virescens *Regel, Bull. Soc. Imp. Naturalistes Moscou 41(2): 299. 1868

*Trisetum spicatum* (L.) Richt. ssp. *virescens* (Regel) Tzvelev, Novosti Sistematiki Vysshchikh Rastenii 7: 65. 1971. 1970

*Aira spicata* L., Sp. Pl. 1: 64. 1753 [applies to ssp. spicatum] Type: “Habitat in Lapponiae alpibus.” Lectotype = “Gramen Avenaceum paniculatum Alp. humile, locustis in spicam collectis varicoloribus aristatis” in Scheuchzer, Agrostographia Helv. Prodr., 24, t. 6, 1708. LT designated by Louis-Marie in Rhodora 30: 238. 1928.

*Avena virescens* (Regel) Regel, Trudy Imp. S.-Peterburgsk. Bot. Sada 7: 635. 1880

*Avena flavescens* L. var. *virescens* Regel, Bull. Soc. Imp. Naturalistes Moscou 41(2): 299. 1868. Type: “Tschilik und Djenischke. Wasserscheide im Altau transiliensis, auf Alpenwiesen bei 8000 fuss Hohe (Semenow).”

*Trisetum pubiflorum* Hack., Oesterr. Bot. Z. 52: 187. 1902. Type: India: Kashmir, Sangam Valley, in rupibus, circ. 13500 ft.; *Duthie 13543*

*Trisetum subspicatum* (L.) P. Beauv., Ess. Agrostogr. 88, 180. 1812, Basionym is nom. superfl.

*Trisetum virescens* (Regel) B. Fedtsch., Izv. Imp. Bot. Sada Petra Velikago 14 (Suppl. 2): 64. 1915, non Nees ex Steud., 1854

*Aira subspicata* L., Syst. Nat., ed. 10. 2: 873. 1759, nom. superfl. & illeg.

*Avena airoides* Koeler, Descr. Gram. (Koeler) 298. 1802 non *Avena spicata* L., 1753

*Trisetum spicatum* (L.) Richt. ssp. *himalaicum* Hulten, Svensk. Bot. Tidskr. 53: 213. 1959, nom. inval., type not indicated (many specimens cited, none indicated as type)

*Trisetum spicatum* (L.) Richt. ssp. *himalaicum* Hulten ex Veldkamp, The Gardens’ Bulletin Singapore 36(1): 135. 1983. Holotype, as lectotype: Nepal: Chilime Kharka, 4570 m, July 1949, *Polunin 1175* (S; IT: BM).

*Koeleria spicata* subsp. *himalaica* (Hultén ex Veldkamp) Barberá, A. Quintanar, Soreng & P.M. Peterson, Phytoneuron 2019–46: 8. 2019

**Distribution.** Arunachal Pradesh, Himachal Pradesh, Jammu and Kashmir, Uttar Pradesh, Uttarakhand [Afghanistan, Bhutan, China, Nepal, Pakistan].

**Habit.** Perennial.

**Remarks.** Records for Arunachal Pradesh, Himachal Pradesh, Jammu and Kashmir, and Uttar Pradesh fide Naithani (1990). Because *Trisetum spicatum* ssp. *himalaicum*, ssp. *spicatum* and ssp. *virescens* may be lumped together in the literature, it is not clear if all subspecies are in India and, if so, what their ranges are.

***Lagurus* L., Sp. Pl. 1: 81. 1753**

**Type. ***Lagurus ovatus* L.

**442. **Lagurus ovatus** L., Sp. Pl. 1: 81. 1753 **

**Type.** “Habitat in Italia, Gallia, Sicilia, Lusitania.” Lectotype: *Herb. Linn. No. 96.1* (LINN). LT designated by Meikle in Fl. Cyprus 2: 1788. 1985.

**Distribution.** Jammu and Kashmir [China, Pakistan].

**Habit.** Annual.

**Remarks.** Record for India fide Naithani (1990).

***Lamarckia* Moench, Methodus (Moench) 201. 1794 (“Lamarkia”), nom. et orth. cons.**

**Type. ***Lamarckia aurea* (L.) Moench (*Cynosurus aureus* L.).

**443. **Lamarckia aurea** (L.) Moench, Methodus (Moench) 201. 1794 **

*Cynosurus aureus* L., Sp. Pl. 1: 73. 1753. Type: “Habitat in Europa australi.” Lectotype: *Herb. Linn. No. 91.19 *(LINN). LT designated by Scholz in Cafferty et al. (ed.), Taxon 49: 249. 2000.

*Achyrodes aureum* (L.) Kuntze, Revis. Gen. Pl. 2: 758. 1891

*Chrysurus aureus* (L.) P. Beauv. ex Spreng., Syst. Veg. (ed. 16) [Sprengel] 1: 296. 1824 (“1825”), non (L.) Besser, 1810 c. f. Taxon 31(1): 70. 1982, Isonym

*Chrysurus cynosuroides* Pers., Syn. Pl. (Persoon) 1: 80. 1805, nom. superfl. & illeg. for *C. aureus *

**Distribution.** Himachal Pradesh, Jammu and Kashmir, Uttar Pradesh [China, Pakistan].

**Habit.** Annual.

***Leucopoa* Griseb. in Ledebour, Fl. Ross. 4(13): 383 1852**

**Type.***Leucopoa sibirica* Griseb. (*Leucopoa albida* (Turcz. ex Trin.) Krecz. & Bobrov).

**444. **Leucopoa albida** (Turcz. ex Trin.) Krecz. & Bobrov, Fl. URSS 2: 495. 1934 **

*Poa albida* Turcz. ex Trin., Mém. Acad. Imp. Sci. St.-Pétersbourg, Sér. 6, Sci. Math. 1: 387. 1830, non *Festuca albida* Lowe, 1831. Lectotype: [Siberia]: Lk. Baikal, mineral springs Turkinskije; 1829; *Turczaninow s.n.* (LE). LT orig. label: “In arenosis ad Baicalem prope thermes Turkenses.” (vide TROPICOS)

*Leucopoa sibirica* Griseb. in Ledebour, Fl. Ross. 4(13): 383. 1852, nom. superfl. for *Poa albida* Turcz. ex Trin.

*Festuca sibirica* Hack. ex Boiss., Fl. Orient. (Boissier) 5: 626. 1884, nom. nov. for *Poa albida* Turcz. ex Trin.

**Distribution.** Reported from Jammu and Kashmir, but probably not in India [Afghanistan, China, Pakistan]. See comments under *Leucopoa olgae*.

**Habit.** Perennial.

**445. **Leucopoa karatavica** (Bunge) Krecz. & Bobrov, Fl. URSS 2: 496. t. 39. f. 2 (a–f). 1934 **

*Poa karatavica* Bunge, Mém. Acad. Imp. Sci. St.-Pétersbourg Divers Savans 7: 525. 1851. Type: “Hab. Auf den Alpen des Karatau 12 Septbr. 1841 (specima duo deflorata sine fructu)”; *Alexandrio Lehmann s.n.*

*Festuca karatavica* (Bunge) B. Fedtsch., Rastitel’n. Turkestana 136. 1915

*Xanthochloa karatavica* (Bunge) Tzvelev, Bot. Zhurn. (Moscow and Leningrad) 91(2): 275. 2006.

**Distribution.** India (Northwest Himalaya) [also in Russia].

**Habit.** Not recorded.

**Remarks.** Distribution in India not recorded.

**446. **Leucopoa olgae** (Regel) Krecz. & Bobrov, Flora URSS 2: 495. 1934 **

*Molinia olgae* Regel, Trudy Imp. S.-Peterburgsk. Bot. Sada 7(2): 625. 1881. Type: “In Kokaniae montibus Alai”; *O. Fedtschenko s.n.*

*Festuca olgae* (Regel) Krivot., Bot. Mater. Gerb. Bot. Inst. Komarova Akad. Nauk S.S.S.R. 20: 56. 1960

**Distribution.** Jammu and Kashmir [Afghanistan, China, Pakistan].

**Habit.** Perennial.

**Remarks.** Although *Leucopoa albida* (Turcz. ex Trin.) Krecz. & Bobrov has been reported from India this is most likely an error, confusing *L. albida* with *L. olgae*. See Dickoré (1995).

***Lolium* L., Sp. Pl. 1: 83. 1753**

**Lectotype. ***Lolium perenne* L. LT designated by Nash in N.L. Britton & A. Brown, Ill. Fl. N. U.S. ed. 2. 1: 281. 1913; affirmed by Hitchcock, Bull. U.S.D.A. 772: 103. 1920.

**447. **Lolium arundinaceum** (Schreb.) Darbysh., Novon 3(3): 241. 1993 **

*Festuca arundinacea* Schreb., Spic. Fl. Lips. 57. 1771. Type: “In prato acclivi hinter dem Biniz, loco humido” Lectotype: Scheuzer, Agrostographia, t. 5. f. 18. 1719. LT designated by Reveal, Terrell, Wiersema & Scholz in Taxon 40: 136. 1991.

*Bromus arundinaceus* (Schreb.) Roth, Tent. Fl. Germ. 2(1): 141. 1789

**Distribution.** Himachal Pradesh [native to Europe].

**Habit.** Perennial.

**Remarks.** Distribution in India fide Naithani (1990). It is unclear where this is naturalized there or not.

**448. **Lolium giganteum** (L.) Darbysh., Novon 3(3): 241. 1993 **

*Bromus giganteus* L., Sp. Pl. 1: 77–78. 1753. Type: “Habitat in Europae sylvis siccis.” Lectotype: *Herb. A. van Royen, sheet no. 913.62–78* (L). LT designated by Darbyshire in Cafferty et al. (ed.), Taxon 49: 248. 2000.

*Festuca gigantea* (L.) Vill., Hist. Pl. Dauphiné (Villars) 2: 110. 1787

*Zerna gigantea* (L.) Panz. ex B.D. Jacks., Index Kew. 1(4): 1249. 1895

*Trisetum flaccidum* (Hack. ex Hook. f.) R.R. Stewart, Brittonia 5(4): 431. 1945

*Avena flaccida* Hack. ex Hook. f., Fl. Brit. India (J.D. Hooker). 7(22): 280. 1896. Type: India: Punjab, Black Mountains, alt. 8000–9000 ft.; *J.F. Duthie 7609.*

**Distribution. **Assam, Himachal Pradesh, Jammu and Kashmir, Meghalaya, Punjab, Uttar Pradesh, Uttarakhand [Bhutan, China, Nepal, Pakistan].

**Habit.** Perennial.

**Remarks.**Bor (1960, p. 448) notes that *Trisetum flaccidum* is a species of *Festuca*, possibly *F. gigantea*. This species is now *Lolium giganteum*.

**449. **Lolium perenne** L., Sp. Pl. 1: 83. 1753 **

*Lolium brasilianum* Nees, Fl. Bras. Enum. Pl. 2(1): 443. 1829. Type: Uruguay: Montevideo; *Sellow s.n.* (B).

**Type.** “Habitat in Europa ad agrorum versuras solo fertili.” Lectotype: *Herb. Linn. No. 99.1 *(LINN). LT designated by Terrell in Taxon. Revis. Genus Lolium 7. 1968.

**Distribution.** Bihar, Himachal Pradesh, Jammu and Kashmir, Meghalaya, Sikkim, Tamil Nadu, Uttar Pradesh [China, Pakistan].

**Habit.** Perennial.

**450. **Lolium persicum** Boiss. & Hohen., Diagn. Pl. Orient. ser. 1, 2(13): 66. 1854 **

*Lolium rigidum* Gaudin var. *duthiei* Hack. ex Hook. f., Fl. Brit. India (J.D. Hooker). 7(22): 364. 1896. Type: India: Kashmir, Sirinagur, alt. 5000–6000 ft.; *Duthie no. 10846*

**Type.** Iran: “Hab. in uliginosis montis Elbrus prope Derbend; June 9, 1943; *Kotschy 278.*” (G).

**Distribution.** Jammu and Kashmir [Afghanistan, Pakistan].

**Habit.** Annual.

**Remarks.** Distribution in India fide Naithani (1990).

**451. **Lolium remotum** Schrank, Baier. Fl. 1: 382. 1789 var. **remotum

**Type.** Germany: Bavaria, “um Burghausen”, type not known according to Terrell (1968).

**Distribution.** Northwest India [Afghanistan, China].

**Habit.** Annual.

**Remarks.** States in India not recorded.

**452. **Lolium remotum** Schrank var. **aristatum** (Doell) Asch., Fl. Brandenburg 1(2): 876. 1864 **

*Lolium linicolum* A. Braun var. *aristatum* Doell, Fl. Baden [Doell] 1: 113. 1857. Type: *Herbarium Doell*

**Distribution.** Himachal Pradesh, Uttar Pradesh [China].

**Habit.** Annual.

**Remarks.** Distribution in India fide Naithani (1990).

**453. **Lolium rigidum** Gaudin, Agrost. Helv. 1: 334–335. 1811 **

*Lolium loliaceum* (Bory & Chaub.) Hand.-Mazz., Ann. K. K. Naturhist. Hofmus. 28(1–2): 32. 1914

*Rottboellia loliacea* Bory & Chaub., Exp. Sci. Moree Bot. 3: 46. t. 3. f. 2. 1832

**Type.** “Augustae Praetoriae ad vias apricas anno 1809 inveni.” *Gaudin s.n.* (LAU).

**Distribution.** Jammu and Kashmir [Afghanistan, Pakistan].

**Habit.** Annual.

**454. **Lolium temulentum** L., Sp. Pl. 1: 83. 1753 var. **temulentum

**Type.** “Habitat in Europae agris inter Hordeum, Linum.” Lectotype: *Herb. Burser I: 113* (UPS). LT designated by Loos & Jarvis in Bot. J. Linn. Soc. 108: 408. 1992.

**Distribution.** Assam, Bihar, Delhi, Himachal Pradesh, Jammu and Kashmir, Kerala, Madhya Pradesh, Maharashtra, Meghalaya, Odisha, Punjab, Rajasthan, Tamil Nadu, Uttar Pradesh, Uttarakhand [China, Pakistan].

**Habit.** Annual.

**455. **Lolium temulentum** L. var. **arvense** (With.) Lilj., Utkast Sv. Fl., ed. 3 80. 1816 **

*Lolium arvense* With., Arr. Brit. Pl., ed. 3. 2: 168. 1796. Type: United Kingdom and cited many syntypes.

**Distribution.** Assam, Delhi, Himachal Pradesh, Jammu and Kashmir, Kerala, Madhya Pradesh, Maharashtra, Meghalaya, Punjab, Rajasthan, Tamil Nadu, Uttar Pradesh, Uttarakhand [also in Europe].

**Habit.** Annual.

**Remarks.** Record for Himachal Pradesh fide Naithani (1990).

***Milium* L., Sp. Pl. 1: 61. 1753**

**Lectotype. ***Milium effusum* L. LT designated by Nash in N.L. Britton & A. Brown, Ill. Fl. N. U.S. ed. 2. 1: 173. 1913; affirmed by Hitchcock, Bull. U.S.D.A. 772: 156. 1920.

**456. **Milium effusum** L., Sp. Pl. 1: 61. 1753 **

*Melica effusa* (L.) Salisb., Prodr. Stirp. Chap. Allerton 20. 1796

*Miliarium effusum* (L.) Moench, Methodus (Moench) 204. 1794

*Paspalum effusum* (L.) Raspail, Ann. Sci. Nat. (Paris) 5: 301. 1825

**Type.** “Habitat in Europae nemoribus umbrosis.” Lectotype: *Herb. Linn. No. 83.3* (LINN). LT designated by Cope in Jarvis et al. (ed.), Regnum Veg. 127: 66. 1993.

**Distribution.** Himachal Pradesh, Jammu and Kashmir, Uttar Pradesh [Afghanistan, Bhutan, China, Pakistan].

**Habit.** Perennial.

***Paracolpodium* (Tzvelev) Tzvelev, Bot. Zhurn. (Moscow & Leningrad) 50: 1320. 1965**

*Colpodium* subg. *Paracolpodium* Tzvelev, Nov. Syst. Pl. Vasc. 1964: 9. 1964.

**Type. ***Paracolpodium altaicum* (Trin.) Tzvelev (*Colpodium altaicum* Trin.).

**457. **Paracolpodium altaicum** (Trin.) Tzvelev ssp. **leucolepis** (Nevski) Tzvelev, Novosti Sist. Vyssh. Rast. 3: 33. 1966 **

*Colpodium leucolepis* Nevski, Byull. Moskovsk. Obshch. Isp. Prir. Otd. Biol. 43: 224. 1934. Type: Tajikistan: Pamir, Fontes fl. Aksu (Murgab), in declivitate NW faucium Beik, in herbosis; July 18, 1901; *Th. Alexeenko 2120* (LE).

**Distribution.** Jammu and Kashmir [also in Tajikistan].

**Habit.** Perennial.

**Remarks.** Distribution in India fide Naithani (1990).

**458. **Paracolpodium wallichii** (Hook. f. ex Stapf) E.B. Alexeev, Novosti Sist. Vyssh. Rast. 18: 94. 1981 **

*Colpodium wallichii* (Hook. f. ex Stapf) Bor, Kew Bull. 1953: 270. 1953

*Catabrosa wallichii* Hook. f. ex Stapf, Fl. Brit. India (J.D. Hooker). 7(22): 312. 1896. Syntypes: Nepal; 1821; Numer. List [Wallich] no. 8907. India: Sikkim, Kankola Pass, alt. 14000–15000 ft.; *J.D. Hooker s.n.*

**Distribution.** Sikkim [Bhutan, China, Nepal].

**Habit.** Perennial.

***Parapholis* C.E. Hubb., Blumea Suppl. 3: 14. 1946**

**Lectotype. ***Parapholis incurva *(L.) C.E. Hubb. **(***Aegilops incurva *L.) Probably designated by A. Chase, Misc. Publ. U.S.D.A. 200: 997. 1950.

**459. **Parapholis incurva** (L.) C.E. Hubb., Blumea, Suppl. 3: 14. 1946 **

*Aegilops incurva* L., Sp. Pl. 2: 1051. 1753. Type: “Habitat in Oriente. Tournefort.” Neotype: *Herb. Linn. No. 1218.11* (LINN). LT designated by Cuccuini in Cafferty et al. (ed.), Taxon 49: 242. 2000.

*Lepturus incurvus* (L.) Trin., Fund. Agrost. (Trinius) 123. t. 8. 1820 (as “incurvatus”)

*Pholiurus incurvus* (L.) Hitch., Bull. U.S.D.A. 772: 106. 1920 (as “incurvatus”)

**Distribution.** Northwest India [China].

**Habit.** Annual.

**Remarks.** States in India not recorded.

***Phalaris* L., Sp. Pl. 54. 1753**

**Lectotype. ***Phalaris canariensis* L. LT designated by Hitchcock, Amer. J. Bot. 5: 252. 1918.

**460. **Phalaris aquatica** L., Cent. Pl. I. 4. 1755 **

*Phalaris tuberosa* L., Mant. Pl. Altera 557. 1771. Type: “Habitat in Europae australi.” Lectotype: *Herb. Linn. No. 78.3* (LINN). LT designated by Anderson in Iowa State J. Sci. 36: 43. 1961.

**Type.** “Habitat in Aegypto & ad Tyberia.” Lectotype: Hasselquist, Herb. Linn. No. 78.4 (LINN). LT designated by Anderson in Iowa State J. Sci. 36: 43. 1961.

**Distribution.** Tamil Nadu [China].

**Habit. **Perennial.

**461. **Phalaris arundinacea** L., Sp. Pl. 1: 55. 1753 **

*Digraphis arundinacea *(L.) Trin., Fund. Agrost. (Trinius) 127. 1820

*Phalaroides arundinacea* (L.) Rauschert, Feddes Repert. 79: 409. 1969

*Typhoides arundinacea* (L.) Moench, Methodus (Moench) 202. 1794

*Arundo colorata* Aiton, Hortus. Kew. (W. Aiton) 1: 116. 1789. Type: Britain

*Calamagrostis variegata* With., Arr. Brit. Pl., ed. 3. 2: 124. 1796

*Arundo riparia* Salisb., Prodr. Stirp. Chap. Allerton 24. 1796, nom. superfl. & illeg.

**Type.** “Habitat in Europae subhumidis adripas lacuum.” Lectotype: Herb. Linn. No. 78.7 (LINN). LT designated by Anderson in Iowa State J. Sci. 36: 37. 1961.

**Distribution.** Bihar, Jammu and Kashmir, Tamil Nadu [China, Pakistan].

**Habit.** Perennial.

**462. **Phalaris canariensis** L., Sp. Pl. 1: 54. 1753 **

*Phalaris avicularis* Salisb., Prodr. Stirp. Chap. Allerton 17. 1796, nom. superfl. & illeg.

**Type.** “Habitat in Europa australi, Canariis.” Proposed Conserved Type: Herb. Clifford: 23, Phalaris 1 (BM-000557659). Type designated by Baldini & Jarvis in Taxon 40: 482. 1991.

**Distribution.** India [China, Pakistan]. Naturalized, native to Mediterranean region.

**Habit.** Annual.

**Remarks.** States in India not recorded.

**463. **Phalaris minor** Retz., Observ. Bot. (Retzius) 3: 8. 1783 var. **minor

**Lectotype.** LINN 89/31.1962. LT designated by R.M. Baldini in Webbia 47(1): 22. 1993.

**Distribution.** Andhra Pradesh, Assam, Bihar, Delhi, Himachal Pradesh, Jammu and Kashmir, Madhya Pradesh, Maharashtra, Manipur, Nagaland, Odisha, Rajasthan, Tamil Nadu, Uttar Pradesh, Uttarakhand [Bhutan, China, Pakistan].

**Habit.** Annual.

**464. **Phalaris minor** Retz. var. **nepalensis** (Trin.) Bor, Grasses Burma, Ceylon, India & Pakistan 616. 1960 **

*Phalaris nepalensis* Trin., Sp. Gram. [Trinius] 1(7): t. 80. 1827. Type: “Figura as specimen nepalense.” Lectotype: *Trinius, Sp. Gram. Icon. t. 80*. 1827. LT designated by Baldini in Webbia 49(2): 279. 1995.

**Distribution.** Bihar, Maharashtra, Uttar Pradesh, Uttarakhand [Nepal].

**Habit.** Annual.

**465. **Phalaris paradoxa** L., Sp. Pl., ed. 2. 2: 1665. 1763 **

**Type.** “Habitat in Oriente. P. Forskåhl.” Lectotype: *Herb. Linn. No. 78.6* (LINN). LT designated by Anderson in Iowa State J. Sci. 36: 22. 1961.

**Distribution.** Northwest India [China, Pakistan].

**Habit.** Annual.

**Remarks.** States in India not recorded.

***Phippsia* (Trin.) R. Br., Chlor. Melvill. 27. 1823**

*Vilfa* subg. *Phippsia* Trin. in K.P.J. Sprengel, Neue Entdeck. Pflanzenk. 2: 37. 1820 (as “*Phipsia*”)

**Type. ***Phippsia algida* (Sol.) R. Br. (*Agrostis algida* Sol.).

**466. **Phippsia algida** (Sol.) R. Br., Chlor. Melvill. 27. 1823 **

*Agrostis algida* Sol., Voy. North Pole 200. 1774. Type: Arctic: “voyage toward the North Pole.” [syntype (BM): Capt. Phipps, Spitsbergen]

**Distribution.** Uttar Pradesh [also in Europe, North America].

**Habit.** Perennial.

**Remarks.** Distribution in India fide Naithani (1990). Taxonomic editor Soreng comments that it is doubtful that this species actually occurs in India.

***Phleum* L., Sp. Pl. 1: 59. 1753**

**Lectotype. ***Phleum pratense* L. LT designated by Nash in N.L. Britton & A. Brown, Ill. Fl. N. U.S. ed. 2. 1: 190. 1913; affirmed by Hitchcock Bull. U.S.D.A. 772: 140. 1920.

**467. **Phleum alpinum** L., Sp. Pl. 1: 59. 1753  **

*Phleum commutatum* Gaudin, Alpina 3: 4. 1808. Type: “Auf den hoheren Alpen im Wallis… z.b. auf den Bernhard und auf den Alpen des Visperthals. Ludw. Thomas, Schleicher.” European Alps, alt. 6400–13000 ft.

**Type.** “Habitat in Alpibus.” Lectotype: *Herb. Linn. No. 81.4 *(LINN). LT designated by Bowden in Canad. J. Bot. 43: 286. 1965.

**Distribution.** Jammu and Kashmir, Uttar Pradesh, Uttarakhand [Afghanistan, Bhutan, China, Pakistan].

**Habit.** Perennial.

**468. **Phleum himalaicum** Mez, Repert. Spec. Nov. Regni Veg. Beih. 17: 293. 1921 **

*Phleum arenarium* L. var. *thomsonii* Griseb., Nachr. Königl. Ges. Wiss. Georg-Augusts-Univ. 83. 1868. Type: Himalaya occidentalis, 6000–8000 ft.; *Thomson s.n.*

*Phleum arenarium* sensu Hook. f., Fl. Brit. India (J.D. Hooker). 7(22): 237. 1896, non L., 1753

**Syntypes.** Afghanistan; *Griffith s.n.* Northwest Himalaya; *Thomson s.n.* Kashmir; *Meebold s.n.*

**Distribution.** Himachal Pradesh, Jammu and Kashmir [Afghanistan, Pakistan].

**Habit.** Annual.

**469. **Phleum paniculatum** Huds., Fl. Angl. (Hudson) 23. 1762 **

*Phleum asperum* Jacq., Collectanea [Jacquin] 1: 110. 1786. Type: “Gramen hoc quibusdam in Europe provincis sponte obvium.”

**Type.** “Habitat in partes infra King’s Waston prope Bristolium.”

**Distribution.** Himachal Pradesh, Jammu and Kashmir [Afghanistan, China, Pakistan].

**Habit.** Annual.

**470. **Phleum pratense** L. var. **pratense**, Sp. Pl. 1: 59. 1753 **

**Type.** “Habitat in Europae versuris & pratis.” Lectotype: *Herb. Linn. No. 26* (LAPP). LT designated by Humphries in Jarvis et al. (ed.), Regnum Veg. 127: 75. 1993.

**Distribution.** Assam [China].

**Habit.** Perennial.

**471. **Phleum pratense** L. var. **nodosum** (L.) Arcang., Compend. Fl. Ital. 757. 1882 **

*Phleum nodosum* L., Syst. Nat. ed. 10:871. 1759.

**Distribution.** Nagaland.

**Habit.** Perennial.

**Remarks.** Distribution in India fide Naithani (1990). It is unclear if this species is naturalized in India.

***Poa* L., Sp. Pl. 1: 67. 1753**

**Lectotype. ***Poa pratensis* L. LT designated by Nash in N.L. Britton & A. Brown, Ill. Fl. N. U.S. ed. 2. 1: 252. 1913; affirmed by Hitchcock Bull. U.S.D.A. 772: 41. 1920.

**472. **Poa aitchisonii** Boiss., Fl. Orient. (Boissier) 5: 602. 1884 (as “aitchisoni”) **

**Type.** Afghanistan; Kurum Valley; *Aitchinson 405, 497.*

**Distribution.** Northwest India [Afghanistan].

**Habit.** Perennial.

**Remarks.** Records from Bor (1960) and Zhu et al. (2006).

**473. **Poa alpina** L., Sp. Pl. 1: 67. 1753 **

**Type.** “Habitat in alpibus Lapponicis, Helveticis.” Lectotype: *Herb. Linn. No. 87.2* (LINN). LT designated by Soreng in Cafferty et al. (ed.), Taxon 49: 254. 2000.

**Distribution.** Assam, Jammu and Kashmir, Himachal Pradesh, Uttar Pradesh, Uttarakhand [Afghanistan, China, Nepal, Pakistan].

**Habit.** Perennial.

**Remarks.** Records from Bor (1960) and Zhu et al. (2006).

**474. **Poa annua** L., Sp. Pl. 1: 68. 1753 **

**Type.** “Habitat in Europa ad vias.” Lectotype: *Herb. Linn. No. 87.17, right specimen* (LINN). LT designated by Soreng in Cafferty et al. (ed.), Taxon 49: 254. 2000.

**Distribution.** Assam, Bihar, Delhi, Himachal Pradesh, Jammu and Kashmir, Karnataka, Kerala, Meghalaya, Punjab, Rajasthan, Tamil Nadu, Uttar Pradesh, Uttarakhand [Afghanistan, Bhutan, China, Myanmar, Nepal, Pakistan, Sri Lanka]. Native to Eurasia and weedy elsewhere around the world.

**Habit.** Annual.

**Remarks.** Records from Bor (1960) and Zhu et al. (2006).

**475. **Poa arnoldii** Melderis, Enum. Fl. Pl. Nepal 1: 142. 1978. **

*Poa albertii* ssp. *arnoldii* (Melderis) Olonova & G.H. Zhu, Fl. China 22: 308. 2006.

*Poa mustangensis* Rajbh., Acta Phytotax. Geobot. 39(1–3): 61. 1988. Type: Nepal: Mustang Distr., 4900 m, in open place, 25 July 1983; *Rajbhandari 8352* (TI, KATH)

**Type.** Nepal: west, 5 mi NE of Saipal, scree, 5600 m, 25 Aug. 1954, *J.E.M. Arnold 226* (BM).

**Distribution.** Uttarakhand [Nepal].

**Habit.** Perennial.

**Remarks.** Specimen records for Uttarakhand listed by Nautiyal and Gaur (2017). Zhu et al. (2006) place this as a subspecies of *P. albertii* and also consider *P. mustangensis* as a synonym.

**476. **Poa asperifolia** Bor, Kew Bull.1952: 130. 1952 **

**Type.** China: Tibet, Pembu La, 10–15 miles north from Lhasa; September, 1904; *H.J. Walton s.n. *(K).

**Distribution.** Sikkim [Bhutan, China].

**Habit.** Perennial.

**Remarks.** Record for Sikkim fide Naithani (1990).

**477. **Poa attenuata** Trin., Mém. Acad. Imp. Sci. St.-Pétersbourg Divers Savans 2: 527. 1835 **

**Type.** Russia: Siberia, Altai Mountain, “in montosis ad fontem fluvii”; July, 1833; *D. Bunge s.n.* (LE-TRIN-2584.01, Isotype).

**Distribution.** India (Northwest Himalaya) [Pakistan].

**Habit.** Perennial.

**Remarks.** Records from Bor (1960) and Zhu et al. (2006). Taxonomic editor Soreng notes that Olonova (pers. comm.) doubts that this species, at least in its strict sense, occurs in India.

**478. **Poa bactriana** Roshev., Bot. Mater. Gerb. Glavn. Bot. Sada RSFSR 4: 93. 1923 **

*Poa bactriana* Roshev. ssp. *glabriflora* (Roshev. ex Ovcz.) Tzvelev, Novosti Sist. Vyssh. Rast. 10: 96. 1973

*Poa bulbosa* var. *glabriflora* Roshev. ex Ovcz., Fl. Turkmen. 1(2): 143. 1932 Lectotype: N. Desyatova [N. Dessinatoff 1398]; 25 Jun 1913; [Tadjikistan]: Iter ad distr. Margelan, Alai valley, Valley of Dara Rv., from Izmail burial ground to Aran-Kungei winter quarters, soft hillocks (LE). ILT: LE.

**Lectotype.** V. Lipsky; ; Turkmenistan: Gissar Range southern slope, Pyanjkhan, 7800 ft (LE; ILT: K-969/64, LE) LT cited by Tzvelev, Zlaki SSSR 450 (1976).

**Distribution.** Himachal Pradesh, Jammu and Kashmir [Afghanistan, China, Pakistan].

**Habit.** Perennial.

**Remarks.** Records from Bor (1960) and Zhu et al. (2006). Taxonomic editor Soreng has investigated the lectotypification of *P. bulbosa* var. *glabriflora*. He notes that Tzvelev (Zlaki SSSR p 450, 1976), cited the Desyatova specimen as the holotype of *P. glabriflora* Roshev. ex Ovcz., and therefore he (Soreng) takes this to be the lectotype. Roshevits did not cite any material. OM: S. Nevski 464; 27 Jun 1931; [Uzbekistan] Turkomania orientalis: In regione subalpina montium Kuhitang supra pagum Chodsha-i-fil (K-67, K-68) K-67 was annotated as *P. glabriflora* by Roshevits in 1931, but this specimen keys to *P. bactriana* subsp. *drobovi* in Tzvelev (1976); fide RJS 2004.

**479. **Poa bucharica** Roshev., Bot. Mater. Gerb. Glavn. Bot. Sada RSFSR 4: 94. 1923 ssp. **bucharica

**Type.** LT: V. Lipskii; 2 Aug 1896; Afghanistan: Bukhara, southern slope of Gissar range, upper reaches of Sio River, 10000 ft (LE; ILT: K). LT: cited by Tzvelev, Zlaki SSSR p. 462 (1976).

**Distribution.** Jammu and Kashmir [Afghanistan, China].

**Habit.** Perennial.

**Remarks.** Records from Bor (1960) and Zhu et al. (2006).

**480. **Poa bucharica** Roshev. ssp. **karateginensis** (Roshev. ex Ovcz.) Tzvelev, Novosti Sist. Vyss. Rast. 11: 28. 1974 **

*Poa karateginensis* Roshev. ex Ovcz., Izv. Tadzh. Bazy Akad. Nauk. SSSR 1(1): 12, 26. 1933. Type: Tajikistan: Buchara, Nusratabadskij Range, Karategin, Karashibet glacier, 3000 m, 3–15 Aug 1896, V. Lipski. HT: LE; IT: K, LE

*Poa suruana* H. Hartmann, Candollea 39(6): 516–519. f. 1. 1984. Type: India: Karakorum, Skardu, Suru, Sanmodagsa (Gulmatungo) 12500 ft.; August 27; 1976; *H. Hartmann 2873* (G).

**Distribution.** Jammu and Kashmir [China].

**Habit.** Perennial.

**481. **Poa bulbosa** L. var. **vivipara** Koeler, Descr. Gram. 189. 1802 **

*Poa bulbosa* L. ssp. *vivipara* (Koeler) Arcang., Comp. Fl. Ital. 785. 1882

*Paneion bulbosum* var. *viviparum* (Koeler) Lunell, Amer. Midl. Naturalist 4: 222. 1915

*Poa bulbosa* L. forma *vivipara* (Koeler) Maire, Fl. Afrique N. 3: 86. 1855

**Type.** Prope Moguntiam [Mainz, Germany] in arenosis.

**Distribution.** Himachal Pradesh, Jammu and Kashmir [Afghanistan, China, Nepal, Pakistan].

**Habit.** Perennial.

**Remarks. **Records from Bor (1960) and Zhu et al. (2006). Taxonomic editor Soreng notes that possibly all collections in India are more or less viviparous, and doubts that any representatives of *P. bulbosa* var. *bulbosa* occur there. Bor (1960, p. 556) also indicates “almost invariably found as var. *vivipara*”.

**482. **Poa burmanica** Bor, Kew Bull. 3: 141. 1948. **

**Type.** Myanmar: Kachin State, Myitkyina Distr., Hpimaw Pass, 11,000 ft, 6 Aug. 1929, *Sukoe10074* (K).

**Distribution.** Arunachal Pradesh, Sikkim, Uttarakhand [Bhutan, China, Myanmar].

**Habit.** Perennial.

**Remarks.** Specimen records for India listed by Nautiyal and Gaur (2017). This species is also accepted by Zhu et al. (2006) in the *Flora of China*.

**483. **Poa calliopsis** Litw. ex Ovcz., Izv. Tadzhik. Bazy Bot. 1: 11, 18. 1933 **

**Lectotype.** Th. Alexeenko 3023/1451; 26 July 1901; Tajikistan: Pamir in angustiis ad fauces Chargosch. N. versus in pratis, 4300–5500 m (LE) LT designated (as holotype) by Roshevits, Fl. SSSR 2: 755 (1934).

**Distribution.** Himachal Pradesh, Jammu and Kashmir [Bhutan, China, Nepal, Pakistan].

**Habit.** Perennial.

**Remarks.** Records from Bor (1960) and Zhu et al. (2006).

**484. **Poa compressa** L., Sp. Pl. 1: 69. 1753 **

**Type.** “Habitat in Europae & Americae septentrionalis siccis. muris, tectis.” Lectotype: *Herb. Linn. No. 87.41* (LINN). LT designated by Soreng in Cafferty et al. (ed.), Taxon 49: 255. 2000.

**Distribution.** Himachal Pradesh, Jammu and Kashmir [China].

**Habit.** Perennial.

**Remarks.** Records from Bor (1960) and Zhu et al. (2006).

**485. **Poa diaphora** Trin., Bull. Sci. Acad. Imp. Sci. Saint-Pétersbourg 1: 69–70. 1836 **

*Poa persica* var. *diaphora* (Trin.) Asch. & Graebn., Syn. Mitteleur. Fl. [Ascherson & Graebner 2: 437. 1900

*Aira altaica* Trin., Mém. Acad. Imp. Sci. St.-Pétersbourg Divers Savans 2: 526. 1835

*Eremopoa altaica* (Trin.) Roshev., Fl. URSS 2: 431. t. 32. f. 13. 1934

**Distribution.** Himachal Pradesh, Jammu and Kashmir [Afghanistan, China, Pakistan].

**Habit.** Annual.

**486. **Poa diaphora** Trin. var. **songarica** (Schrenk ex Fisch. & C.A. Mey.) Soreng, Cabi & L.J. Gillespie, PhytoKeys 111: 85. 2018. **

*Glyceria songarica* Schrenk ex Fisch. & C.A. Mey., Enum. Pl. Nov. 1: 1. 1841. Type: Kazakhstan: “ad fl. Karatai versus montes Karatau”; June 13, 1840; *H. Schrenk s.n.* (LE).

*Poa songarica* (Schrenk ex Fisch. & C.A. Mey.) Boiss., Fl. Orient. (Boissier) 5: 611. 1884 (as “soongarica”)

*Eremopoa songarica* (Schrenk ex Fisch. & C.A. Mey.) Roshev., Fl. URSS 2: 431. t. 32. f. 11. 1937 (as “soongorica”)

*Poa persica* Trin. var. *songarica* (Schrenk ex Fisch. & C.A. Mey.) Stapf, Fl. Brit. India (J.D. Hooker)

*Eremopoa persica* (Trin.) Roshev. var. *songarica* (Schrenk ex Fisch. & C.A. Mey.) Bor, Grasses Burma, Ceylon, India & Pakistan 532. 1960

*Eremopoa altaica* (Trin.) Roshev. var. *songarica* (Schrenk ex Fisch. & C.A. Mey..) Tzvelev, Poaceae URSS 479. 1976

*Eremopoaaltaica* subsp. *songarica* (Schrenk ex Fisch. & C.A. Mey.) Tzvelev, Botanicheskii Zhurnal (Moscow & Leningrad) 51(8): 1104. 1966

*Nephelochloa songarica* (Schrenk ex Fisch. & C.A. Mey.) Griseb., Fl. Ross. [Ledebour] 4: 367. 1852 (as “soongorica”)

**Distribution.** Jammu and Kashmir, Uttar Pradesh [Afghanistan, Pakistan].

**Habit.** Annual.

**Remarks.** Records from Bor (1960) and Zhu et al. (2006). Taxonomic editor Soreng places *P. songarica* as a variety of *P. diaphora*.

**487. **Poa dzongicola** Noltie, Edinburgh J. Bot. 57(2): 283, f. 1M-P. 2000 **

*Poa yakiangensis* L. Liou, Fl. Reipubl. Popularis Sin. 9(2): 405. 2002

**Type.** HT: I.W.J. Sinclair & D. G. Long 5396; 28 Sep 1984; Bhutan: Upper Mo Chu District, Lingshi Dzong, 27°55', 89°27', on wall of dzong, ca. 4100m (E).

**Distribution.** Sikkim [Bhutan, China].

**Habit.** Annual.

**Remarks.** Records from Bor (1960) and Zhu et al. (2006).

**488. **Poa eleanorae** Bor, Kew Bull. 1948: 142. 1948 **

**Type.** H.A. Cummins; “Laid in” 13 Jun 1894; India: Northeast Sikkim. K000789631.

**Distribution.** Sikkim, Uttarakhand [Bhutan, Nepal].

**Habit.** Perennial.

**Remarks.** Records from Bor (1960), and Nautiyal and Gaur (2017).

**489. **Poa falconeri** Hook. f. ex Stapf, Fl. Brit. India (J.D. Hooker). 7(22): 342. 1896 **

**Syntypes.***Falconer*; 10 Aug. 1870–1871; Garwahl, 12–13000 ft (K000789637; IST: K000789636) [Jumnotri to Kasauli, Uttar Pradesh to Himachal Pradesh]; *Duthie 288*; 16 Aug. 1983; India: Uttar Pradesh: Tihri-Garwhal, in Nila Valley 12–13,000 ft (K000789635).

**Distribution.** Himachal Pradesh, Jammu and Kashmir, Uttar Pradesh, Uttarakhand [Pakistan, Nepal].

**Habit.** Perennial.

**490. **Poa gamblei** Bor, Kew Bull. 1948: 144. 1948 **

**Type.** India: Madras, Nilgiris Distr. Ootacamund, 7000 ft, Sep 1886, *J.S. Gamble 18129* (K000789628).

**Distribution.** Himachal Pradesh, Tamil Nadu.

**Habit.** Perennial.

**Remarks.** Records from Bor (1960).

**491. **Poa gammieana** Hook. f. ex Stapf, Fl. Brit. India (J.D. Hooker). 7(22): 345. 1896 **

**Type.** India: Sikkim, Tankra Mountains, alt. 12000 ft.; August 5, 1892; *G.A. Gammie 641* (HT: (K000789632).

**Distribution.** Assam, Sikkim, Uttarakhand [Bhutan].

**Habit.** Perennial.

**Remarks.** Records from Bor (1960), and Nautiyal and Gaur (2017).

**492. **Poa garhwalensis** D.C. Nautiyal & R.D. Gaur, J. Bombay Nat. Hist. Soc. 96(2): 285. 1999. **

**Type.** Jammu-Kashmir: NW Himalaya, Leptal, 4000m, 4 Aug 1996; *D.C. Nautiyal* (GUH-13501A).

**Distribution.** Jammu and Kashmir, Uttarakhand.

**Habit.** Perennial.

**493. **Poa glauca** Vahl, Fl. Dan. 6(17): 3, pl. 964. 1790. **

*Poa araratica* s. auct., non Trautv.

*Poa sterilis* M. Bieb., Fl. Taur.-Caucas. 1: 62. 1808. Type: “Habitat in tauriae collibus apricis sterilibus [Crimea].” (LE).

**Type.** “Legi tantummodo in paroecia Vang (Valders) ad pedes montium, in Finmarkia non frequens.”

**Distribution.** Arunachal Pradesh, Assam, Bihar, Himachal Pradesh, Jammu and Kashmir, Uttarakhand [China, Nepal, Pakistan].

**Habit.** Perennial.

**Remarks.** Records from Bor (1960) and Zhu et al. (2006). Taxonomic editor Soreng notes that the specialist in this group of *Poa* (sect. *Stenopoa*), M.V. Olonova, now considers *P. araratica* to be a taxonomic synonym of *Poa glauca* Vahl. but probably does not extend to India. The Cenral Asian plants may be better treated as *Poa glauciculmis* Ovcz. More research is needed. Certainly the taxonomy of this section is complex; see (Zhu et al. 2006). *Poa glauca* is circumboreal and also occurs in S America. *Poa sterilis* was accepted as a separate species by Bor (1960) and Zhu et al. (2006), but now is considered a synonym of *P. glauca* s.l. or possibly *P. nemoraliformis* and almost certainly does not occur in India.

**494. **Poa harae** Rajbh., Acta Phytotax. Geobot. 39(1–3): 55. 1988 **

**Type.** Nepal: Sagarmantha Zone, Solukhumbu Distr., Denikharka (Dambuk) – Yurigolcha, 4600m, on grassy slope, 23 Aug 1985; *Rajbhandari 61665* (TI, KATH).

**Distribution.** Uttarakhand [Nepal].

**Habit.** Perennial.

**Remarks.** Not listed for China by Zhu et al. (2006).

**495. **Poa himalayana** Nees, Syn. Pl. Glumac. (Steudel) 1: 256. 1854 **

*Poa stewartiana* Bor, Kew Bull.1951: 185. 1951. Type: India: Jaunsar District, Mandali, 6400 ft.; May 5, 1897; *J.F. Duthie 19777* (K).

**Type.** Nepal, Lectotype: India: Uttar Pradesh, Mussooree, Shalma; *Royle 163 on 187/163* (LIV-12514). LT designated by Noltie in Edinburgh J. Bot. 57(2): 289. 2000.

**Distribution.** Himachal Pradesh, Jammu and Kashmir, Uttarakhand, Uttar Pradesh [Pakistan, Nepal, Tibet, China].

**Habit.** Perennial.

**Remarks.** Records from Bor (1960) and Nautiyal and Gaur (2017). Taxonomic editor Soreng notes that *Poa himalayana *sensu Bor is *Poa rajbhandari*; see Noltie (2000). *Poa stewartiana* Bor is *Poa himalayana*.

**496. **Poa hirtiglumis** Hook. f. ex Stapf, Fl. Brit. India (J.D. Hooker). 7(22): 343–344. 1896 **

**Type.** India: Sikkim: Donkaih Pass, alt. 18000 ft.; September 9, 1849; *Herb. Ind. Or. J.D. Hooker & Thomson s.n. *(K).

**Distribution.** Assam, Sikkim, Uttarakhand [Bhutan, Nepal].

**Habit.** Perennial.

**Remarks.** Records from Bor (1960) and Nautiyal and Gaur (2017).

**497. **Poa hylobates** Bor, Bull. Bot. Surv. Ind. 7: 132. 1965 **

**Type.** Nepal: near Tarakoti, Bheri River, 10500 ft, grassy clearings in mixed forest, 13 July 1952; *Polunin, Sykes & Williams 2445* (K, BM).

**Distribution.** Uttarakhand [China, Nepal].

**Habit.** Annual.

**Remarks.** This species is also reported for China by Zhu et al. (2006).

**498. **Poa infirma** Kunth, Nov. Gen. Sp. [H.B.K.] 1: 158 (ed. qto.). 1816 **

*Catabrosa thomsonii* Stapf ex Hook. f., Fl. Brit. India (J.D. Hooker). 7(22): 311. 1896 (as “thomsoni”). Type: China: Xizang, Nubra Valley, alt. 10000–11000 ft.; *T. Thomson s.n.*

*Poa annua* L. ssp. *exilis* Tomm. ex Freyn, Verh. K.K. Zool.-Bot. Ges. Wien 27: 469. 1878 (“1877”). Type: Langs der Kust von Fasana bis Medolino; auch auf S. Marina; 1872; *Tommasini s.n.*

*Poa exilis* (Tomm. ex Freyn) Murb., Syn. Mitteleur. Fl. [Ascherson & Graebner]. 2(1): 389. 1900

*Poa remotiflora* (Hack.) Murb., Acta Univ. Lund. 36(2): 22. 1900, non Rupr., 1845.

*Poa annua* L. forma *remotiflora* Hack., Flore de l’Algérie 2: 206. 1895. (Fl. Algérie). Type: Algeria: Lieux humides et region Montagneuse, Rouiba, Teniet-el-Haad.

**Type.** Colombia; August, 1801; *Humboldt & Bonpland 134* (P). “Crescit in frigidis regni Novogranatensis, inter Fonibon, Suba et Santa Fe de Bogota, 1360 hexap.”

**Distribution.** Bilaspur, Uttarakhand. [Introduced. Widespread in Europé and North America].

**Habit.** Annual.

**Remarks.** Records from Bor (1960) and Nautiyal and Gaur (2017).

**499. **Poa kanaii** Rajbh., Acta Phytotax. Geobot. 39(1–3): 58. 1988 **

**Type. **Nepal: Mustang Distr., Thourungse, 4600 m, in open place, 25 Jul 1983; Rajbhandari 8356 (TI, KATH).

**Distribution. **Uttarakhand [Nepal].

**Habit. **Perennial.

**Remarks.** Not listed for China by Zhu et al. (2006).

**500. **Poa khasiana** Stapf, Fl. Brit. India (J.D. Hooker). 7(22): 343. 1896 **

**Lectotype.** Designated by Bor, Bombay Natur. Hist. Soc. J. 50: 831 (1952), without indication of herb. As in the protolog, this was originally det as “*P. himalayana* Nees”. Stapf’s figures pinned on illustrate the LT, K000789598.

**Distribution.** Arunachal Pradesh, Assam, Meghalaya, Nagaland, Sikkim, Uttarakhand, West Bengal [China, Myanmar, Nepal].

**Habit.** Perennial.

**Remarks.** See also Nautiyal and Gaur (2017) for additional discussion of this species.

**501. **Poa koelzii** Bor, Kew Bull. 1948: 139–140. 1948 **

*Poa albertii* Regel ssp. *kunlunensis* (N.R. Cui) Olonova & G.H. Zhu, Flora of China 22: 308. 2006

*Poa festucoides* Lam. ssp. *kunluensis* N.R. Cui, Acta Botanica Boreali-Occidentalia Sinica 7(2): 97, f. 8. 1987, nom. hom. illeg.

**Type.** India: Kashmir, Tsakzhun Tso, Ladak, alt. 15000 ft.; July 20, 1931; *Walter Koelz 2385* (holotype: K000789620).

**Distribution.** Himachal Pradesh, Jammu and Kashmir, Ladakh, Uttar Pradesh, Uttarakhand [China, Pakistan].

**Habit.** Perennial.

**Remarks.** See discussion in Nautiyal and Gaur (2017).

**502. **Poa lahulensis** Bor, Kew Bull. 1948: 138. 1948 **

*Poa albertii* Regel ssp. *lahulensis* (Bor) Olonova & G.H. Zhu, Fl. China 22: 309. 2006

**Type.** Kashmir: Lingti, Lahul, alt. 12000 ft.; June 29, 1941; *N.L. Bor 15024* (K).

**Distribution.** Himachal Pradesh, Jammu and Kashmir, Uttarakhand [China, Pakistan].

**Habit.** Perennial.

**Remarks.** Records for India confirmed by M. Olonova. Specimens for Uttarakhand cited by Nautiyal and Gaur (2017).

**503. **Poa lhasaensis** Bor, Bull. Bot. Surv. India 7: 132. 1965 **

*Poa jaunsarensis* Bor, Kew Bull. 1948: 143. 1948. Type: India: Kumaon, Kutti Yangti Valley, Byans, 12000–13000 ft.; July 30, 1886; *J.F. Duthie 6224* (K000789644).

**Type.** China: Xizang: Lhasa, 10000 ft,, Sep 1904 Collector and Number: Walton s.n. (K).

**Distribution.** Himachal Pradesh, Jammu and Kashmir, Uttar Pradesh, Uttarakhand [Pakistan, China, Nepal].

**Habit.** Perennial.

**Remarks.** Records for Uttar Pradesh and Nepal fide Naithani (1990). Taxonomic editor Soreng notes that *Poa jaunsarensis* was accepted as *Poa lhasaensis* Bor by Bor (1960) and Zhu et al. (2006), but this is considered tentative.

**504. **Poa litvinoviana** Ovcz., Izv. Tadzhik. Bazy Bot. 1: 22. 1933   **

*Poa glauca* Vahl ssp. *litwinowiana* (Ovcz.) Tzvelev, Novosti Sist. Vyssh. Rast. 11: 32. 1974. Type: USSR, Tadzhikistan; Tajikistan: Pamiro-Alaj, Tian Shan, distr. Matscha, inter rupibus in montibus Zeravschanicis pr. glaciem zeravschanicum, 4500–4700 m, 16 July 1927, *Drobov 354 *(LE).

**Distribution.** Jammu and Kashmir [China, Pakistan].

**Habit.** Perennial.

**505. **Poa mairei **Hack., Repert. Spec. Nov. Regni Veg. 12(333–335): 387. 1913 **

*Poa pseudo-pratensis* Hook. f., Fl. Brit. India (J.D. Hooker). 7(22): 340. 1896, non Beyer, 1891 nec Scribn. & Rydb., 1896. Syntypes: Nepal; *Wallich s.n.* Sikkim and Western Bhutan, alt. 10000–12000 ft.; *J.D. Hooker s.n. *Sine loc; *Cummins s.n.*

*Poa patens *Keng ex Keng f., Acta Botanica Yunnanica 4(3): 276–277. 1982. Type: China: Yunnan:, Yulong Shan, eastern slopes of mount Dyinaloko, northern peak of Likang Snow Range, 1923, *J.F. Rock 10427* (HT: US-1214301)

*Poa ludens* R.R. Stewart, Brittonia 5(4): 420. 1945, nom. nov. for *Poa pseudo-pratensis* Hook. f.

**Type.** China; Yunnan, pr. Tong-Tchouan, 1910, *R.P. Maire 6992, ser. B* (HT: W).

**Distribution.** Assam, Jammu and Kashmir, Punjab, Sikkim [Bhutan, China, Nepal].

**Habit.** Perennial.

**Remarks.** Records from Bor (1960) and Zhu et al. (2006).

**506. **Poa nemoraliformis** Roshev., Bot. Mater. Gerb. Bot. Inst. Komarova Akad. Nauk SSSR 11: 30. 1949. **

**Syntypes.** India, Himachal Pradesh, Bor. or *Poa relaxa* Ovcz., Izv. Tadzh. Bazy Akad. Nauk. SSSR 1(1): 20, f. 2. 1933. Type: Tajikistan: western Zeravshan range, near Marguzarsky Lake, stony screes along NW slope of Mun-Kua Mt., 2900 m, 20 Aug. 1932. P. Ovchinnikov & A. Slobodov 1257.

**Distribution.** West Himalaya [China].

**Habit.** Perennial.

**507. **Poa nemoralis** L., Sp. Pl. 1: 69. 1753 **

**Type.** “Habitat in Europa ad radices montium umbrosas.” Lectotype = “Gramen paniculatum angusti-folium Alpin. Locustis rarioribus et angustioribus non aristatis” in Scheuchzer, Agrostographia Helv. Prodr., 18. t. 2. 1708. LT designated by Soreng in Cafferty et al. (ed.), Taxon 49: 255. 2000. Epitype: Sweden. Uppland: Danmark Parish, Linnés Hammarby; June 14, 1933; *Hylander s.n.* (BM). Epitype designated by Soreng, R. & Edmondson, J. in Cafferty et al. (ed.), Taxon 49: 255. 2000.

**Distribution.** Arunachal Pradesh, Himachal Pradesh, Jammu and Kashmir, Sikkim, Tamil Nadu, Uttar Pradesh, Uttarakhand, West Bengal [Bhutan, China, Nepal, Pakistan].

**Habit.** Perennial.

**508. **Poa nepalensis** (Wall. ex Griseb.) Wall. ex Duthie, List Grasses N.W. India 40. 1883 var. **nepalensis

*Poa annua* L. var. *nepalensis* Wall. ex Griseb., Nachr. Königl. Ges. Wiss. Georg-Augusts-Univ. 75. 1868. Lectotype: *Strachey & Winterbottom 13*, India orientali: Kumaon, Binsar, 2300 m (K; ILT: BM)

*Poa nephelophila* Bor, Kew Bull. 1948: 140. 1948

**Distribution.** Himachal Pradesh, Sikkim, Uttar Pradesh, Uttarakhand [Bhutan, China, Myanmar, Nepal, Pakistan].

**Habit.** Annual.

**Remarks.** Taxonomic editor Soreng notes that the lectotype of *P. annua* var. *nepalensis* was designated by Bor (1952) without indication of herbarium. The K specimen was cited as the holotype by Cope (1982) and by Rajbhandari (1991). Lectotype here designated (refined), K000789542 = p.p. “a”, by R.J. Soreng per annotation 2010 on specimen. (p.p. “b”, is *Poa setulosa *Bor.) The placement of *P. nephelophila* in synonymy here follows Bor (1960) and Nautiyal and Gaur (2017).

**509. **Poa nitide-spiculata** Bor, Kew Bull. 1948: 139. 1948 **

**Type.** India: Sikkim Himalaya, Valley running into Teesta from West, Tangu, alt. 13500–14000 ft.; July 13, 1903; *F.E. Younghusband s.n.* (K000789633).

**Distribution.** Sikkim [China, Nepal].

**Habit.** Perennial.

**Remarks.** Records for Sikkim and Nepal fide Naithani (1990).

**510. **Poa pagophila** Bor, Fl. Iranica 70: 38. 1970 **

*Poa pagophila* Bor, Kew Bull. 1949: 239. 1949, nom. inval., without Latin. Type: Sikkim: Yeumtang, 4600 m, 6 Sept. 1849, *Hooker f. s.n. *(HT: K000789639; IT: GOET-006888)

*Poa flexuosa* sensu Stapf, Fl. Brit. India (J.D. Hooker). 7(22): 342. 1896, non Sm., 1800, nec Wahlb., 1814

**Distribution.** Jammu and Kashmir, Sikkim, Uttar Pradesh, Uttarakhand [Bhutan, China, Nepal, Pakistan].

**Habit.** Perennial.

**Remarks.** Taxonomic editor Soreng notes that Bor’s 1949 publication of *P. pagophila* was invalid, lacking a Latin description. Bor cited only the English description of *P. flexuosa* sensu Stapf. The Latin description was provided by Bor (1970), p. 38. Stapf clearly attributed *P. flexuosa* to Wahlberg.

**511. **Poa palustris** L., Syst. Nat., ed. 10. 2: 874. 1759 **

*Poa serotina* Ehrh., Beitr. Naturk. (Ehrhart) 6: 83. 1791, nom. nud.; Ehrh. ex Hoffm., Fl. Germ. 1: 299. 1806. Type: Upsaliae

**Type. **“Habitat [in Helvetiae, Italiae paludibus.] Sp. Pl., ed. 2. 1: 99. 1762.” Lectotype: Herb. Linn. No. 87.21 (LINN). LT designated by Soreng in Cafferty et al. (ed.), Taxon 49: 256. 2000.

**Distribution.** Jammu and Kashmir, Uttarakhand [China, Pakistan]. Introduced; native to temperate regions of the Northern Hemisphere.

**Habit.** Perennial.

**512. **Poa persica** Trin., Mém. Acad. Imp. Sci. St.-Pétersbourg, Sér. 6, Sci. Math. 1: 373. 1830 var. **persica

*Eremopoa persica* (Trin.) Roshev., Fl. URSS 2: 430. t. 32. f. 8. 1934

*Festuca persica* (Trin.) C. Koch, Linnaea 21: 410. 1848

*Nephelochloa persica* (Trin.) Griseb., Fl. Ross. [Ledebour] 4: 366–367. 1852

*Poa heptantha* (C. Koch) Steud., Syn. Pl. Glumac. 1: 255. 1854

*Festuca heptantha* C. Koch, Linnaea 21: 410. 1848. Type: “Im Hochgebirge, auf sumpfigen Wiesen, auf Urgestein, 5500 ft.” (B).

*Festuca polygama* C. Koch, Linnaea 21: 409. 1848. Type: “aus dem Wilhelm’schen herbar als.” Caucasus?; *Wilhelms s.n.* (B).

**Lectotype.** “V. ssp. Pers.” [Caucasus; Azerbaijan]: in collibus ad Akar-Tschai prob, Karabagh; May 27, 1829; *Szowits 246* (LE). LT designated by Tzvelev in Zlaki SSSR. 479. 1976.

**Distribution.** Himachal Pradesh, Jammu and Kashmir, Uttar Pradesh, Uttarakhand [Afghanistan, Pakistan].

**Habit.** Annual.

**Remarks.** See also Gillespie et al. (2018). Records from Bor (1960) and Zhu et al. (2006).

**513. **Poa polycolea** Stapf, Fl. Brit. India (J.D. Hooker). 7(22): 342. 1896 **

*Poa chalarantha* Keng ex L. Liu, Fl. Reipubl. Popularis Sin. 9(2): 390 2002. Type: *K.L. Chu 7012*, China: Sichuan: Yulinkun, ad declivitatem saxosam umbrosam, 1 April 1940 (NAS).

*Poa gilgitica *Dickoré, Stapfia 39: 169 1995. Type: India: Karakorum: Gilgit, Shinghai Gah - Pahot Gali 35°48'–55'N, 74°10'–17'E, *Picea smithiana* forest on NW-facing slope, veg. rec. 37, 3100m; 26 Jul 1990, *G. & S. Miehe 796 *(HT: K; IT: GOET).

*Poa lithophila *Keng ex L. Liu, Fl. Reipubl. Popularis Sin. 9(2): 397, 2002. Type: China: Sichuan: Dêgê [ca 31.8°N 98.6°E], alt. 2800–4300 m, *Y.W. Tsui 4413* (HT: N).

*Poa maerkangica *L. Liu, Fl. Reipubl. Popularis Sin. 9(2): 399, 2002. Type: China: Sichuan: Maerkang, in locis gramineis declivitatis, 2800 m, 11 May 1959, *Li Xin 70629* (HT herbarium unknown).

*Poa triglumis* Keng ex L. Liu, Fl. Reipubl. Popularis Sin. 9(2): 404, 2002. Type: 10 Jun 1937; Sichuan: Mu-ii, Wa-chin, bank of mt. stream, 2800m, *FIB Yunnan exp. T.T. Yu 6032* (HT: NAS (not found 2001 or 2004); IT: KUN-388592, KUN-388590, PE-308548)

**Lectotype.***J.D. Hooker & Thomson, Herb. Ind. Or. Hf. & T. 15*, West Himalaya: Kashmire, Valley north of Chamba, 3–3500 m (K000789594; ILT; BM) LT designated by Bor B.N.H.S.J. 50: 835 (1952), without indication of herb.

**Syntype.** India: Kumaon, Dugli, alt. 10500 ft.; *R. Strachey & J.E. Winterbottom 11* (as *P. serotina*) (K; IST: BM).

**Distribution.** Jammu and Kashmir, Uttar Pradesh, Uttarakhand [Afghanistan, Bhutan, China, Nepal, Pakistan].

**Habit.** Perennial.

**514. **Poa polyneuron** Bor, Kew Bull.1952: 223. 1952 **

**Type.** India: Sikkim, Natu La, 12800 ft.; June 23, 1945; *N.L. Bor & Kiratram 20685* (K000789638).

**Distribution.** Sikkim [China].

**Habit.** Perennial.

**Remarks.** Records from Bor (1960) and Zhu et al. (2006).

**515. **Poa poophagorum** Bor, Kew Bull. 1948: 143. 1948 (as “poiphagorum”) **

*Poa albertii* Regel ssp. *poophagorum* (Bor) Olonova & G.H. Zhu, Fl. China 22: 308. 2006

**Type.** India: Sikkim, Temu La, alt. 16000 ft.; 1912; *Rohmoo Lepcha 374* (K).

**Distribution.** Sikkim, Uttarakhand [China, Nepal].

**Habit.** Not reported.

**Remarks.** Taxonomic editor Soreng notes that this species was recognized at the subspecific level by Bor (1960) and Zhu et al. (2006) as *P. albertii* ssp. *poophagorum*, but more recently has been accepted as a species.

**516. **Poa pratensis** L., Sp. Pl. 1: 67–68. 1753 ssp. **pratensis**, nom. cons. **

**Type.** “Habitat in Europae pratis fertilissimis.” Conserved Type: Russia. Prov. Sanct-Petersburg, 5 km australi-occidentem versus a st. viae ferr. Mga, pratulum ad ripam dextram fl. Mga, 26 Jun 1997, Tzvelev N-257 (BM-000576302). Type designated by Soreng & Barrie in Taxon 48: 157. 1999.

**Distribution.** Arunachal Pradesh, Assam, Himachal Pradesh, Jammu and Kashmir, Meghalaya [Afghanistan, Bhutan, China, Myanmar, Nepal, Pakistan, Sri Lanka].

**Habit.** Perennial.

**Remarks.** Taxonomic editor Soreng notes that this species is widely seeded for lawns and pastures in cool temperate regions. It is presumably naturalized in India.

**517. **Poa pratensis** L. ssp. **alpigena** (Lindm.) Hiitonen, Suom. Kasvio 205. 1933 **

*Poa alpigena* Lindm., Svensk Fanerogamflora 91. 1918. Based on “*P. pratensis var. alpigena*” Fr. ex Blytt, nom. illeg. superfl. for “*P. pratensis var. iantha*” Laest.

*Poa pratensis* L. var. *alpigena* Fr. ex Blytt, Norg. Flora 130. 1861. Type: “P. prat. Alpigena Fries Herbar. Norm. Fasc. 9.n. 93.1.p. prat. Alpestris Fries Summa Veget. P. 76. P. Prat. δ. Iantha Blytt l.c.”

*Poa pratensis *L. var. *iantha* Laest., Kongl[iga]. Vetenskaps Academiens Handlingar 1822: 329. 1822

*Poa poophagorum* Bor var. *lanata* Bor, Kew Bull. 1948: 143. 1948. Type: China: Tibet, Rongshar Valley, alt. 12000 ft.; *Hingston s.n.* (K).

**Distribution.** Sikkim [China, Nepal (in Naithani as ssp. staintonii)].

**Habit. **Perennial.

**Remarks.** Records from Bor (1960) and Zhu et al. (2006).alpina

**518. **Poa pratensis** L. ssp. **angustifolia** (L.) Gaudin, Agrost. Helv. 1: 214. 1811 **

*Poa angustifolia* L., Sp. Pl. 1: 67. 1753. Type: “Habitat in Europa ad agrorum versuras.” Lectotype: Herb. Linn. No. 87.12, excl. second culm from left (LINN). LT designated by Soreng in Cafferty et al. (ed.), Taxon 49: 254. 2000.

*Poa pratensis* L. var. *angustifolia* (L.) Sm., Fl. Brit. 105. 1800

**Distribution.** India [Afghanistan, Bhutan, China, Nepal, Pakistan]. Naturalized and possibly native; native to Europe and west and central Asia.

**Habit.** Perennial.

**Remarks.** Taxonomic editor Soreng notes that this name is widely misapplied to *Poa pratensis* ssp. *pratensis*; it could be in India, but the identifications should be checked.

**519. **Poa pratensis** L. ssp. **irrigata** Lindb., Schedae Plantae Findlandiae Exsiccatae 2: 20. 1916 **

*Poa irrigata* Lindm., Botaniska Notiser 1905: 88, f. 1. 1905. (Bot. Not.), nom. incorrect as applied by the author, taxonomically superfl.; *Poa rigens* Hartm. 1820 cited as fo. within, see Art. 52 and 52.3. Isotypes: Ehrhart; Scandinavia: Upsaliae (LE) equals *P. humilis* Ehrh., nom. nud., *P. humilis* Ehrh. ex Hoffm. Ehrhart spec. B; 1 Oct 1893; Scandinavia: Upsala (LE)

*Poa subcaerulea* Sm., Engl. Bot. 14: t. 1004. 1802. Type: England: Anglesea (on the Mountains of Westmoreland and Cumberland); *Rev. H. Davies s.n. *

**Distribution.** Jammu and Kashmir. Naturalized; native to Eurasia.

**Habit.** Perennial.

**Remarks.** Records from Bor (1960) and Zhu et al. (2006). Taxonomic editor Soreng notes that this is a common lawn grass and sometimes pasture grass, and is widely seeded. This is the currently accepted name for *Poa humilis*, and for *Poa subcaerulea* as when treated as subspecies of *Poapratensis* L.

**520. **Poa pratensis** L. ssp. **pruinosa** (Korotky) Dickoré, Stapfia 39: 173. 1995 **

*Poa pruinosa* Korotky, Repertorium Specierum Novarum Regni Vegetabilis 13: 291. 1914. (Repert. Spec. Nov. Regni Veg.) Type: *M. Korotky et al. s.n.*; 4 Jul 1912; Russia, Transbaical district, Regio lacorum Eravinensis, pr. p. Konstantinowka, pr. ripam fl. Choloj et lacos Brarun-Charga and Syntyr, in arenosis (Lectotype: LE) LT cited in Tzvelev, Zlaki SSSR 459 (1976).

*Poa markgrafii* H. Hartmann, Candollea 39(2): 514. f. 2. 1984. Type: India: Karakorum, Suru, Chellong Nalla, W Panikhar, 12000 ft.; *H. Hartmann 2380* (G).

**Distribution.** Jammu and Kashmir [China, Pakistan].

**Habit.** Perennial.

**Remarks.** Records from Bor (1960) and Zhu et al. (2006).

**521. **Poa pseudamoena** Bor, Kew Bull. 1953: 276. 1953 **

*Poa amoena* Bor, Kew Bull. 1948: 140. 1949, nom. later hom., non (Presl) Kunth, 1833. Type: India: Kumaon; *R. Strachey 26/2* (K000789626).

**Distribution.** Uttar Pradesh, Uttarakhand [China].

**Habit.** Annual or short-lived perennial.

**Remarks.** Record for Uttar Pradesh fide Naithani (1990), and for Uttarakhand fide Nautiyal and Gaur (2017).

**522. **Poa rajbhandarii** Noltie, Edinburgh J. Bot. 57(2): 288–289, f. 2M-P. 2000 **

**Type.** Sikkim: Phedang to Tsoka, S of Dzongri, 27°26'N, 88°10'E, 3500 m, 26 July 1992, Edinburgh Expedition to Sikkim and Darjeeling (ESIK) 748 (E00393984).

**Distribution.** Assam, Sikkim, West Bengal [Bhutan, China, Nepal].

**Habit.** Annual.

**Remarks.***Poa himalayana *sensu Bor is *Poa rajbhandari *(see Noltie 2000). Taxonomic editor Soreng also notes that the situation is further confused by Nautiyal and Gaur (2017) accepting *P. stewartiana*.

**523. **Poa royleana** Nees, Syn. Pl. Glumac. (Steudel) 1: 256. 1854 **

**Type.** 187/140; Mussooree (LIV (12941-I and 12941-II)[upper culm and base mounted separately] (photo K-969/176)).

**Distribution.** Uttar Pradesh, Uttarakhand.

**Habit.** Perennial.

**Remarks.** This species is sometimes placed as a synonym of *Poa annua*, but taxonomic editor Soreng notes that *Poa royleana* Nees ex Steud. is not at all like *P. annua*. It is a robust perennial, and a very odd one at that; it is apparently very rare. See also Rajbhandari and Edmondson (1990). Regarding the type specimen Soreng observes that the K-969/176 photo shows that both parts I and II were originally mounted on one sheet belonging to Merseyside County Museums Herbarium LIVCM 1952-121-128H with the 187/140 *Poa royleana* - Mussooree tag and the *PoaRoyleana* N. ab. E.-- tag. The spikelets are shaped like *Glyceria striata*; the lemmas are (5)7-(9)veined, glabrous; the rachilla is round, smooth, glabrous; the lodicules have stiff hairs at the apex; the paleas are peculiar -- flanges appressed hispidulous, keels densely hispidulous, and between keels densely hispidulous; the caryopsis is glabrous, with a short round to ovate hilum; sheaths mostly closed ca. 1/2 the length.

**524. **Poa setulosa** Bor, Kew Bull. 1948: 142. 1948 **

*Poa rhadina* Bor, Kew Bull. 1948: 138. 1948. Type: Tehri-Garwahal, Jaulea bah, Srikanta, alt. 12000–13000 ft.; *J.F. Duthie 265* (K000789593).

**Type.** India: Lower Kunawar; August 15, 1847; *T. Thomson s.n.* (K000789592).

**Distribution.** Himachal Pradesh, Jammu and Kashmir, Uttar Pradesh, Uttarakhand [Pakistan].

**Habit.** Annual or perennial.

**Remarks.** Records for Himachal Pradesh, Jammu and Kashmir, and Uttar Pradesh fide Naithani (1990). Nautiyal and Gaur (2017) separate *P. rhadina* from *P. setulosa*, and cite localities in Uttarakhand. Bor (1960) and Zhu et al. (2006) place *P. rhadina* in synonymy as done here.

**525. **Poa sikkimensis** (Stapf) Bor, Kew Bull. 1952: 130. 1952 **

*Poa annua* L. var. *sikkimensis* Stapf, Fl. Brit. India (J.D. Hooker). 7(22): 346. 1896. Lectotype: India: Sikkim, Wallanchoon [Walungchung], 10000–12000 ft.; *J.D. Hooker s.n.* (K000808543). LT (called type) designated by N.L. Bor in Kew Bull. 1952: 130. 1952. (Accepted as Holotype by Rajbhandhari (1991), and as lectotype by Soreng annotation 2010).

**Distribution.** Assam, Jammu and Kashmir, Sikkim, Uttarakhand [Bhutan, China].

**Habit.** Perennial.

**Remarks.** Taxonomic editor Soreng notes that Bor annotated a different specimen as type in 1947 (K000808542), but later published the Wallanchoon specimen as the Type (Bor 1952). The latter was accepted as the holotype by Rajbhandhari (1991), and by Soreng in annotation.

**526. **Poa sinaica** Steud., Syn. Pl. Glumac. 1: 256. 1854 **

**Isotypes.** In cacuminae montis St. Catharinae Arab.; May 19, 1835; *Schimper Unio itiner. 326* (BM, K, S).

**Distribution.** India (West Himalaya) [Afghanistan, Pakistan].

**Habit.** Perennial.

**Remarks.** Records from Bor (1960) and Zhu et al. (2006). Taxonomic editor Soreng notes that this species apparently reaches to Pakistan, it is mostly viviparous over much of its range, especially eastward, and is thus difficult to separate from *Poa bulbosa* subsp. *vivipara*. It has more elongated bulbs at the base of shoots, and longer ligules than *P. bulbosa*.

**527. **Poa stapfiana** Bor, Kew Bull. 1949: 239. 1949 **

*Poa tremula* Stapf, Fl. Brit. India (J.D. Hooker). 7(22): 344. 1896, non Lam., 1791. Syntypes: Temperate and Alpine Himalaya, from Kashmir, alt. 8000–15000 ft.; *Jacquemont s.n.* to Garhwal; *Duthie s.n.* Western Tibet, Ladakh; *Thomson s.n., Schlagintweit s.n.*

*Poa stapfiana* Bor var. *micranthera* (Stapf) Bor, J. Bombay Nat. Hist. Soc. 50: 827. 1952

*Poa tremula* Stapf var. *micranthera* Stapf, Fl. Brit. India (J.D. Hooker). 7(22): 345. 1896. Syntypes: India: Kashmir, Palgam, 12000–13000 ft.; September 4, 1876; *C.B. Clarke s.n., Duthie s.n.*

**Distribution.** Himachal Pradesh, Jammu and Kashmir, Sikkim, Uttarakhand [China, Nepal, Pakistan].

**Habit.** Perennial.

**Remarks.** Taxonomic editor Soreng observes that there are no worthy varieties here; all is one species. However, the Jacquemont collection, listed as a syntype of *P. tremula* is actually *P. hirtiglumis*. The lectotype designated by Rajbhandari (1991) is rejected for *P. tremula*, as it was only cited by Stapf as syntype of the var. *micranthera.*

**528. **Poa supina** Schrad., Fl. Germ. (Schrader) 1: 289. 1806 **

*Poa variegata* Haller f., Cat. Pl. Helv. 38. 1800, nom. illeg., non Lam., 1791

*Poa ustulata* S.E. Fröhner, Botanische Jahrbücher für Systematik, Pflanzengeschichte und Pflanzengeographie 88: 437. 1968. (Bot. Jahrb. Syst.). Type: Russia: Siberia: Pamiro-Alaj mountains, Alajica valley, Tengiz-bai pass, alpine swales, slope toward Darant-Kurgan, 23 June 1931, *S. Lipschitz 122* (HT: MW; IT: LE)

*Poa supina* Schrad. ssp. *ustulata* (S.E. Fröhner) Á. Löve & D. Löve, Botaniska Notiser 128(4): 498. 1975[1976]. (Bot. Not.)

**Type.** In summis alpibus Salisburgensibus; *Mielichhofer s.n.*

**Distribution.** Himachal Pradesh, Jammu and Kashmir, Uttar Pradesh, Uttarakhand [Afghanistan, China, Nepal, Pakistan].

**Habit.** Perennial.

**Remarks.** Records for Himachal Pradesh and Jammu and Kashmir fide Naithani (1990). Nautiyal and Gaur (2017) document specimen for Uttarakhand.

**529. **Poa szechuensis** Rendle, J. Bot. 46: 173. 1908 var. **szechuensis

*Poa gracillima* Rendle, Journal of the Linnean Society, Botany 36(254): 424–425. 1904, nom. illeg. hom., non Vasey 1893. Type: China; Sichuan, Emei Shan, *Faber 1185* (HT: K000789505)

*Poa tibeticola* Bor, Kew Bull. 1948: 139. 1948. Type: China: Tibet, Khambajong; September 7, 1903; *F.E. Younghusband s.n. *(K000789482).

*Poa chumbiensis* Noltie, Edinburgh Journal of Botany 57(2): 282, f. 1I–L. 2000. Type: China: Xizang: Chumbi Valley, Yatung, 10000 ft, 18 April 1945, *Bor & Kirat Ram 20148* (K)

**Distribution.** Sikkim, Uttarakhand [Bhutan, China, Nepal].

**Habit.** Annual.

**Remarks.** Records from Bor (1960) and Zhu et al. (2006).

**530. **Poa szechuensis** Rendle var. **rossbergiana** (K.S. Hao) Soreng & G.H. Zhu, Fl. China 22: 295. 2006 **

*Poa rossbergiana* K. S. Hao, Bot. Jahrb. Syst. 68(5): 581. 1938. Type: China; Qinghai, in der Nähe des Klosters Ta-schiu-sze, 4200 m, im Ja-he-mari-Gebirge, westlich von Tsi-gi-gan-ba, 9 Sep 1930, *K.S. Hao 1197* (IT: NAS)

*Poa rohmooana* Noltie, Edinburgh Journal of Botany 57(2): 281–282, f. 1E–H. 2000. (Edinburgh J. Bot.). Type: Sikkim: Chugya, 15000 ft, 12 Nov. 1912, *Rohmoo Lepcha 284* (HT: E)

**Distribution.** Sikkim [China].

**Habit.** Annual.

**531. **Poa trivialis** L., Sp. Pl. 1: 67. 1753 ssp. **trivialis

**Type.** “Habitat in Europae pascuis.” Neotype: *Hudson 16, Herb. Linn. No. 87.9* (LINN). Neotype designated by Soreng in Cafferty et al. (ed.), Taxon 49: 256. 2000.

**Distribution.** Himachal Pradesh, Jammu and Kashmir, Tamil Nadu [Afghanistan, Bhutan, China, Pakistan].

**Habit.** Perennial.

**Remarks.** All records for India fide Naithani (1990).

**532. **Poa trivialis** L. var. **glabra** Döll, Rhein. Fl. 92. 1843. **

**Type.** Not found.

**Distribution.** Jammu and Kashmir.

**Habit.** Perennial.

**Remarks.** Record in India fide Naithani (1990). Taxonomic editor Soreng notes that Bor (1952, 1960) recognized this species, and Tzvelev (1983) says it is sometimes recognized. It has smoother sheaths than the typical variety.

**533. **Poa wardiana** Bor, Kew Bull. 1948: 143. 1948 **

**Type.** India: Assam, Balipara Frontier Tract, Poshing La, 10000–12000 ft.; July 21, 1938; *F. Kingdon-Ward 13990* (K000789597, ex DD).

**Distribution.** Arunachal Pradesh, Assam [China].

**Habit.** Perennial.

**Remarks.** Record for India fide Naithani (1990).

***Polypogon* Desf., Fl. Atlant. 1: 66. 1798 (“1800”)**

**Type. ***Polypogon monspeliensis* (L.) Desf. (as “monspeliense”) (*Alopecurus monspeliensis *L.).

**534. **Polypogon fugax** Nees, Syn. Pl. Glumac. (Steudel) 1: 184. 1854 **

*Polypogon higegaweri* Steud., Syn. Pl. Glumac. 1: 422. 1854. Type: Japonica

*Polypogon littoralis* Sm. var. *higegaweri* (Steud.) Hook. f., Fl. Brit. India (J.D. Hooker). 7(22): 246. 1896

**Type.** Nepal.

**Distribution.** Arunachal Pradesh, Assam, Bihar, Himachal Pradesh, Jammu and Kashmir, Manipur, Meghalaya, Nagaland, Punjab, Tamil Nadu, Uttar Pradesh, Uttarakhand [Afghanistan, Bhutan, China, Myanmar, Nepal, Pakistan].

**Habit.** Annual.

**535. **Polypogon monspeliensis** (L.) Desf., Fl. Atlant. 1: 67. 1798 var. **monspeliensis

*Alopecurus monspeliensis* L., Sp. Pl. 1: 61. 1753. Type: “Habitat Monspelii.” Lectotype: Herb. Linn. No. 82.6 (LINN). LT designated by Scholz in Cafferty et al. (ed.), Taxon 49: 245. 2000.

*Phleum monspeliense* (L.) Koeler, Descr. Gram. (Koeler) 57. 1802

*Santia monspeliensis* (L.) Parl., Fl. Palerm. 1: 73. 1845

*Agrostis alopecuroides* Lam., Tabl. Encycl. 1: 160. 1791. Type: “Ex Gallia & Europa australi.”

*Phleum crinitum* Schreb., Beschr. Graes. 1: 151. 1768

*Polypogon flavescens* J. Presl in C. Presl, Reliq. Haenk. 1(4–5): 234. 1830. Type: “Habitat in Peruvia.” Peru; *Haenke s.n. *(PR)

**Distribution.** Assam, Bihar, Chhattisgarh, Delhi, Gujarat, Himachal Pradesh, Jammu and Kashmir, Jharkhand, Karnataka, Madhya Pradesh, Maharashtra, Manipur, Meghalaya, Nagaland, Rajasthan, Tamil Nadu, Uttar Pradesh, Uttarakhand, West Bengal [China, Pakistan, Sri Lanka].

**Habit.** Annual.

**536. **Polypogon monspeliensis** (L.) Desf. var. **indicus** Bhattacharya & S.K. Jain, Bull. Bot. Surv. India 25(1–4): 208. f. 1. 1985 (“1983”) **

**Type.** India: Punjab, Ferozepore; *S.l. s.n.* (CAL).

**Distribution.** Assam, Bihar, Delhi, Himachal Pradesh, Jammu and Kashmir, Karnataka, Madhya Pradesh, Maharashtra, Nagaland, Punjab, Rajasthan, Tamil Nadu, Uttar Pradesh, West Bengal [Afghanistan, Sri Lanka].

**Habit.** Annual.

**Remarks.** Record for Punjab fide Naithani (1990).

**537. **Polypogon nilgiricus** Kabeer & V.J. Nair, Nord. J. Bot. 25(1–2): 9, fig. 1. 2008 **

**Type.** India: Tamil Nadu, Nilgiri Hills, Manjakambai, alt. 3000 ft.; March 3, 2004; *K. Althaf Ahamed Kabeer 117749* (CAL).

**Distribution.** Tamil Nadu.

**Habit.** Perennial.

**538. **Polypogon viridis** (Gouan) Breistr., Bull. Soc. Bot. France 110 (Sess. Extraord. 89): 56. 1966 (“1963”) **

*Agrostis viridis* Gouan, Hortus Monsp. 546. 1762. Type: France “Habitat au mont Saint Loup. Cum agrost. Ventricosa Altitudo prioris.” (P).

*Agrostis semiverticillata* (Forssk.) C. Chr., Dansk Bot. Ark. 4(3): 12. 1922

*Phalaris semiverticillata* Forssk., Fl. Aegypt.-Arab. 17. 1775. Type: “Rofettae & Kahirae frequens, Aprile ineunre florens, Naaejm.”Egypt: Rosetta and Cairo; April, 1762; *Forsskal 63* (C).

*Agrostis verticillata* Vill., Prosp. Hist. Pl. Dauphiné (Villars) 16. 1779. Type: France.

*Polypogon littoralis* Sm. var. *muticus* Hook. f., Fl. Brit. India (J.D. Hooker). 7(22): 246. 1896. Type: India: Kunawur; *Thomson s.n.*

**Distribution.** Uttar Pradesh [Pakistan].

**Habit.** Perennial.

***Puccinellia* Parl., Fl. Ital. 1: 366. 1848, nom. cons.**

**Type. ***Puccinellia distans* (Jacq.) Parl. (*Poa distans* Jacq.) (type cons*.*).

For additional information see Liu et al. (2006) and Dickoré (1995). Dickoré notes several poorly known taxa from Karakorum that are not mentioned here.

**539. **Puccinellia distans** (Jacq.) Parl., Fl. Ital. (Parlatore) 1: 367. 1848 **

*Poa distans* Jacq., Observ. Bot. (Jacquin) 1: 42. 1764, Type: “Habitat in Austria. D. Jacquin.” Lectotype: Austria. “Poa distans. triandra digyn. Crescit in fossis aquosis et locis humidis per Austriam.” *Herb. Jacquin fil.* (W). LT designated by Cope in Cafferty et al. (ed.), Taxon 49: 255. 2000.

*Atropis distans* (Jacq.) Griseb., Fl. Ross. [Ledebour] 4: 388. 1852

*Glyceria distans* (Jacq.) Wahlenb., Fl. Upsal. 36. 1820

**Distribution.** Jammu and Kashmir [China, Pakistan].

**Habit.** Perennial.

**540. **Puccinellia glauca** (Regel) V.I. Krecz., Flora URSS 2: 484, pl. 36, f. 19. 1934 **

*Atropis distans* var. *glauca* Regel, Trudy Imperatorskago S.-Peterburgskago Botaničeskago Sada 7(2): 623. 1881. (Trudy Imp. S.-Peterburgsk. Bot. Sada). Type: *P. Fedchenko s.n.*, 29 May 1869, Environs of Kalta-Kurgon, along the irrigation channel Narupai (LE)

*Puccinellia distans* Parl. ssp. *glauca* (Regel) Tzvelev, Zlaki SSSR 507. 1976

**Distribution.** Jammu and Kashmir [Afghanistan, China, Pakistan].

**Habit.** Perennial.

**541. **Puccinellia himalaica** Tzvelev, Bot. Mater. Gerb. Bot. Inst. Komarova Akad. Nauk S.S.S.R. 17: 66. 1955 **

**Type.** India: Kashmir, Soo Morari Peldo, Rupshu, alt. 12800 ft.; July 8, 1931; *W. Koeltz 2216* (LE).

**Distribution.** Himachal Pradesh, Jammu and Kashmir [Afghanistan, China, Pakistan].

**Habit.** Perennial.

**Remarks.** Records for India fide Naithani (1990).

**542. **Puccinellia ladakhensis** (H. Hartmann) Dickoré, Stapfia 39: 182. 1995 **

*Poa ladakhensis* H. Hartmann, Candollea 39(2): 510. f. 1. 1984. Type: India: Kashmir, Stokphu, alt. 14500 ft.; *H. Hartmann 2614* (G).

**Distribution.** Jammu and Kashmir.

**Habit.** Perennial.

**543. **Puccinellia minuta** Bor, Nytt Mag. Bot., Oslo 1: 19, f. 8. 1952. **

*Puccinellia kashmiriana* Bor, Kew Bull. 1953: 270–271. 1953. Type: India: Kashmir, Kamri Valley, near Kalapani, alt. 11200–12000 ft.; August 25, 1893; *J.F. Duthie 12543* (K).

**Type.** Pakistan: Chitral distr.: Barum Gol, S. Barum Glacier, ca. 4500 m, 27 July 1950, *P*. *Wendelbo s.n.* (K).

**Distribution.** Himachal Pradesh, Jammu and Kashmir [Afghanistan, China, Pakistan].

**Habit.** Perennial.

**Remarks.** Records for India fide Naithani (1990). Taxonomic editor Soreng notes that Dickoré (1995) recognized *P*. *minuta* with *P. kashmiriana* Bor as a synonym, a structure that we follow here. Liu et al. (2006) recognize *P. minuta* but do not mention *P. kashmiriana*.

**544. **Puccinellia stapfiana** R.R. Stewart, Brittonia 5(4): 418. 1945 **

*Glyceria pooides* Stapf, Fl. Brit. India (J.D. Hooker). 7(22): 348. 1896 (as “poaeoides”), non *Puccinellia pooides* Keng, 1938. Type: China: Xizang, Rupchu, grassy plains at the head of Salt Lake; *T. Thomson s.n.*, non *Puccinellia pooides* Keng, 1938

**Distribution.** Jammu and Kashmir [China, Pakistan].

**Habit. **Perennial.

**545. **Puccinellia tenuiflora** (Griseb.) Scribn. & Merr., Contr. U.S. Natl. Herb. 13: 78. 1910 **

*Atropis tenuiflora* Griseb., Fl. Ross. [Ledebour] 4(13): 389. 1852. Type: Russia: Siberia, Transbaical; *Turczaninov s.n. *(LE).

**Distribution.** India (West Himalaya) [China, Pakistan].

**Habit.** Perennial.

**Remarks.** States in India not recorded. Taxonomic editor Soreng notes that Liu et al. (2006) include Iran but not Pakistan in the distribution of this species.

**546. **Puccinellia thomsonii** (Stapf) R.R. Stewart, Brittonia 5(4): 418. 1945 (as “thomsoni”) **

*Glyceria thomsonii* Stapf, Fl. Brit. India (J.D. Hooker). 7(22): 347. 1896. Type: China: Xizang, Rupchu above Pugha, alt. 15500 ft.; *T. Thomson s.n.*

*Atropis thomsonii* (Stapf) Pamp., Fl. Caracorum 77. 1930

**Distribution.** Jammu and Kashmir [China, Pakistan].

**Habit.** Perennial.

***Rostraria* Trin., Fund. Agrost. (Trinius) 149. 1820**

**Lectotype. ***Rostraria pubescens* Trin., nom. illeg. (*Bromus dactyloides* Roth) (designated by Pfeiffer, Nomenclat. Bot. 2: 991. 1874).

**547. **Rostraria clarkeana** (Domin) Holub, Folia Geobot. Phytotax. 9(3): 271. 1974 **

*Koeleria clarkeana* Domin, Biblioth. Bot. 14(Heft 65): 272. 1907. Type: India: Kashmir, Naosheva; 1876; *C.B. Clarke 28155* (K).

*Lophochloa clarkeana* (Domin) Bor, Grasses Burma, Ceylon, India and Pakistan 445. 1960

**Distribution.** Jammu and Kashmir [Pakistan].

**Habit. **Annual.

**Remarks.** Record for Jammu and Kashmir fide Naithani (1990).

**548. **Rostraria cristata** (L.) Tzvelev, Novosti Sist. Vyssh. Rast. 7: 47. 1971 (“1970”) **

*Festuca cristata* L., Sp. Pl. 1: 76. 1753. Type: “Habitat in Lusitanae collibus sterilibus.” Lectotype: *Herb. Linn. No. 92.24* (LINN). LT designated by Sherif & Siddiqi in El-Gadi (ed.), Fl. Libya 145: 167. 1988.

**Distribution.** Jammu and Kashmir [Pakistan].

**Habit. **Annual.

**549. **Rostraria pumila** (Desf.) Tzvelev, Novosti Sist. Vyssh. Rast. 7: 48. 1971(“1970”) **

*Avena pumila* Desf., Fl. Atlant. 1: 103. 1798. Lectotype: Morocco; 1806; *Broussonet 296 *(LE). (Vide Henderson & Schäfer, Bot. J. Linn Soc. 141: 130. 2003.)

*Lophochloa pumila* (Desf.) Bor, Grasses Burma, Ceylon, India and Pakistan 445. 1960. Type: Pakistan: Rawalpindi; *R.R. Stewart 23443*

**Distribution.** Rajasthan [Pakistan].

**Habit.** Perennial.

***Sclerochloa* P. Beauv., Ess. Agrostogr. 97, 177. 1812**

**Lectotype. ***Sclerochloa dura* (L.) P. Beauv. (*Cynosurus durus* L.). (vide Pfeiffer, Nomenclat. Bot. 2: 1103. 1874).

**550. **Sclerochloa dura** (L.) P. Beauv., Ess. Agrostogr. 98, 177. t. 19. f. 4. 1812 **

*Cynosurus durus* L., Sp. Pl. 1: 72. 1753. Type: “Habitat in Europa australi.” Lectotype = “Gramen arvense, Polypodii panicula crassiore” in Barrelier, Pl. Galliam, 111, t. 50, 1714. LT designated by Stace & Jarvis in Bot. J. Linn. Soc. 91: 438. 1985.

*Eleusine dura* (L.) Lam., Tabl. Encycl. 1: 203. 1791

*Festuca dura* (L.) Vill., Hist. Pl. Dauphiné (Villars) 2: 94. 1787

*Poa dura* (L.) Scop., Fl. Carniol., ed. 2. 1: 70. 1772. Type: “Habitat Tergesti, iuxta vias, in aridis.”

*Sesleria dura* (L.) Kunth, Révis. Gramin. 1: 110. 1829

**Distribution. **Jammu and Kashmir [China, Pakistan].

**Habit. **Annual.

***Sibirotrisetum* Barberá, Soreng, Romasch., A. Quintanar, & P.M. Peterson, J. Syst. Evol.: 8. 2019**

**Type.***Sibirotrisetum sibiricum* (Rupr.) Barberá.

**551. **Sibirotrisetum aeneum** (Hook. f.) Barberá, J. Syst. Evol.: 8. 2019. **

*Avena aenea* Hook. f., Fl. Brit. India (J.D. Hooker). 7(22): 279. 1896. Type: Western Himalaya, alt. 11000–12000 ft., from Kashmir to Kumaon.

*Trisetum aeneum* (Hook. f.) R.R. Stewart, Brittonia 5(4): 431. 1945

*Trisetum aureum* Nees, Syn. Pl. Glumac. (Steudel) 1: 225. 1854, non Ten., 1820. Type: Nepal; *Royle 44*

**Distribution.** Himachal Pradesh, Jammu and Kashmir, Uttar Pradesh, Uttarakhand [Pakistan].

**Habit.** Perennial.

**552. **Sibirotrisetum scitulum** (Chrtek ex Barberá) Barberá, J. Syst. Evol. 2019 (doi: 10.1111/jse.12523): 8. **

*Trisetum scitulum* Bor, Kew Bull. 1956: 212. 1956. Type: India: Sikkim; *J.D. Hooker s.n.*

*Avena flavescens* sensu Hook. f., Fl. Brit. India (J.D. Hooker). 7(22): 279. 1896, non L., 1753

**Distribution.** Uttarakhand, Sikkim [Bhutan, China, Nepal].

**Habit.** Perennial.

**553. **Sibirotrisetum sibiricum** (Rupr.) Barberá subsp. **sibiricum**, J. Syst. Evol. 8. 2019. **

*Trisetum sibiricum* Rupr., Beitr. Pflanzenk. Russ. Reiches 2: 65. 1845

*Trisetum sikkimense* (Hook. f.) Chrtek, Acta Univ. Carol., Biol. 1966: 92. 1967 [page 1967: 104?]

*Avena sikkimensis* Hook. f., Fl. Brit. India (J.D. Hooker). 7(22): 280. 1896. Type: Sikkim Himalaya, Lachen and Lachoong Valleys, alt. 10000–11000 ft.; *J.D. Hooker s.n.*

**Distribution.** Sikkim.

**Habit.** Perennial.

***Trisetaria* Forssk., Fl. Aegypt.-Arab. 27. 1775**

**Type. ***Trisetaria linearis* Forssk.

**554. **Trisetaria loeflingiana** (L.) Paunero, Anales Jard. Bot. Madrid 9: 527. 1950 **

*Avena loeflingiana* L., Sp. Pl. 1: 79. 1753. Type: “Habitat in Hispania.” Lectotype: Löfling s.n., Herb. Linn. No. 95.4, upper row of specimens (excl. specimen at bottom left) (LINN). LT designated by Scholz in Cafferty et al. (ed.), Taxon 49: 247. 2000.

*Lophochloa cavanillesii* (Trin.) Bor, Grasses Burma, Ceylon, India and Pakistan 445. 1960

*Trisetaria cavanillesii* (Trin.) Maire, Fl. Afrique N. 2: 251. 1953

*Trisetum cavanillesii* Trin., Mém. Acad. Imp. Sci. St.-Pétersbourg, Sér. 6, Sci. Math. 1: 63. 1830 (1831)

**Distribution.** Northwest India [Pakistan].

**Habit.** Annual.

**Remarks.** States in India not recorded. Taxonomic editor Soreng cites unpublished phylogenetic data suggesting that this species may be misplaced in *Trisetaria*.

***Trisetopsis* Röser & A. Wölk, Schlechtendalia 25: 57. 2013**

**Type.***Trisetopsis elongata* (Hochst. Ex A. Rich.) Röser & A. Wölk (*Danthonia elongata *Hochst. ex A.Rich.).

**Notes.** Taxonomic editor Soreng notes that *Trisetopsis* Röser & A. Wölk (2013) and *Tzveleviochloa* Röser & A. Wölk (2017) were described to accommodate 28 and 3 species formerly treated as *Helictotrichon*. There is a confusing array of named species along the Himalayan front and extending into the Hengduan Shan of China, for which (in his opinion) there is no adequate taxonomic account. *Helictotrichon schmidtii* and *H. polyneurum* belong to this group, but there is no newer combination or synonymy for these two, although Röser may have placed them in synonymy of others. These are all species with lax leaves unlike *Helictotrichon* s.s. There are no species of *Helictotrichon* s.s. in India. See also discussions in Wölk and Röser (2013, 2014, 2017) and Wölk et al. (2015).

**555. **Trisetopsis aspera** (Munro ex Thwaites) Röser & A. Wölk, Taxon 66(1): 38. 2017 **

*Avena aspera* Munro ex Thwaites, Enum. Pl. Zeyl. 372. 1864. Type: Sri Lanka: Newera Ellia and Other of the more elevated parts of the Island;* Munro Ceylon Plant 916*

*Avenastrum asperum* (Munro ex Thwaites) Vierh. var. *polyneuron* (Hook. f.) C.E.C. Fisch., Fl. Madras 3(10): 1803. 1934

*Avenastrum asperum* (Munro ex Thwaites) Vierh., Verh. Ges. Deutsch. Naturf. 85(2): 672. 1914

*Helictotrichon asperum* (Munro ex Thwaites) Bor, Indian Forest Rec., Bot. n.s. 1(3): 68. 1938

*Avena aspera* Munro ex Thwaites var. *roylei* Hook. f., Fl. Brit. India (J.D. Hooker). 7(22): 277. 1896. Type: Kashmir to Nepal, alt. 6000–12000 ft.

*Avena polyneura* Hook. f., Fl. Brit. India (J.D. Hooker). 7(22): 277–278. 1896. Type: India: Nilghiri Hills, Dodabetta, alt. 8000 ft.; *Gamble s.n.*

*Helictotrichon polyneurum* (Hook. f.) Henrard, Blumea 3(3): 425. 1940

*Helictotrichon roylei* (Hook. f.) Keng, Clav. Gen. Sp. Gram. Prim. Sin. 200. 1957

**Distribution.** Tamil Nadu.

**Habit.** Perennial.

**Remarks.** Note that *Avena polyneura* Hook. f. has never been formally transferred to *Trisetopsis*.

**556. **Trisetopsis junghuhnii** (Buse) Röser & A. Wölk, Taxon 66(1): 38. 2017 **

*Avena junghuhnii* Buse, Pl. Jungh. 345. 1854. Type: Indonesia: Java, Kedu, in planitie montis Dieng, ubi una cum Bromo insigni Buse prope pagum Parre kesit; *Junghuhn s.n. *(L).

*Helictotrichon junghuhnii* (Buse) Henrard, Blumea 3(3): 425. 1940

**Distribution.** Assam, Himachal Pradesh, Jammu and Kashmir, Kerala, Meghalaya, Nagaland, Sikkim, Tamil Nadu, Uttar Pradesh [Bhutan, China, Myanmar, Nepal, Pakistan, Sri Lanka].

**Habit. **Perennial.

**557. **Trisetopsis virescens** (Nees) Röser & Wölk, Taxon 66(1): 38. 2017 **

*Trisetum virescens* Nees, Syn. Pl. Glumac. (Steudel) 1: 226. 1854. Type: India; *Royle Herb. no. 137, 138*

*Helictotrichon virescens* (Nees) Henrard, Blumea 3(3): 425. 1940

**Distribution.** Himachal Pradesh, Jammu and Kashmir, Kerala, Tamil Nadu, Uttarakhand [China, Myanmar, Nepal, Pakistan, Sri Lanka].

**Habit.** Perennial.

***Trisetum* Pers., Syn. Pl. (Persoon) 1: 97. 1805**

**Lectotype**. *Avena flavescens* L. LT designated by Hitchcock, U.S.D.A. Bull. 772: 108. 1920.

**Notes.** Generic limits in *Koeleria*, *Trisetum* and related genera have been the subject of much recent work. See Barberá et al. (2017a, 2017b, 2018, 2019a, 2019b) and Peterson et al. (2019).

**558. **Trisetum flavescens** (L.) P. Beauv., Ess. Agrostogr. 88, 153, 180. 1812 **

*Avena flavescens* L., Sp. Pl. 1: 80. 1753. Type: “Habitat in Germania, Anglia, Gallia.” Lectotype: Herb. A. van Royen No. 913.7–458 (L). LT designated by Cope in Cafferty et al. (ed.), Taxon 49: 247. 2000.

*Trisetaria flavescens* (L.) Baumg., Enum. Stirp. Transsilv. 3: 263. 1816

*Rebentischia flavescens* (L.) Opiz, Lotos 4: 104. 1854, nom. inval. pro syn. of *Trisetum flavescens* (L.) P. Beauv., 1812

*Trisetum pratense* Pers., Syn. Pl. (Persoon) 1: 97. 1805, nom. superfl. & illeg. for *Avena flavescens*

**Distribution.** Meghalaya, Sikkim [also in Europe, North America, South America]. Widespread and native in Eurasia, but not in China.

**Habit.** Perennial.

**559. **Trisetum spicatum** (L.) Richt. ssp. **mongolicum** Hultén ex Veldkamp, Gard. Bull. Singapore 36(1): 135. 1983 **

*Trisetum mongolicum* (Hultén ex Veldkamp) Peshkova, Fl. Centr. Sibir. 1: 97. 1979

**Type.** China: Xizang: Kuenlun, Ulan Bulak, 5 July 1894, *Roborovsky s.n.* (HT: S).

**Distribution.** Sikkim [Bhutan, China].

**Habit.** Perennial.

***Tzveleviochloa* Röser & A. Wölk, Taxon 66(1): 38. 2017.**

**Type.***Tzveleviochloa parviflora* (Hook. f.) Röser & A. Wölk, Taxon 66(1): 38. 2017. (*Avena aspera *Munro ex Thwaites var. *parviflora *Hook. f.).

**Notes.** See comments under *Trisetopsis*, and also discussions in Wölk and Röser (2013, 2014, 2015, 2017).

**560. **Tzveleviochloa parviflora** (Hook. f.) Röser & A. Wölk, Taxon 66(1): 38. 2017 **

*Avena aspera* Munro ex Thwaites var. *parviflora* Hook. f., Fl. Brit. India (J.D. Hooker). 7(22): 277. 1896. Type: India: Sikkim, Singalela, alt. 11000 ft.; *C.B. Clarke s.n.*

*Helictotrichon parviflorum* (Hook. f.) Bor, Kew Bull. 1951: 445. 1952

**Distribution.** Sikkim.

**Habit.** Perennial.

**561. **Tzveleviochloa schmidii** (Hook. f.) E. A. Kellogg, comb. nov. **

urn:lsid:ipni.org:names:77212057-1

*Helictotrichon schmidii* (Hook. f.) Henrard, Blumea 3(3): 427. 1940

*Avena aspera* Munro ex Thwaites var. *schmidii* Hook. f., Fl. Brit. India (J.D. Hooker). 7(22): 277. 1896. Syntypes: India: Nilghiri Hills; *Schmid s.n.* Ootacamund, alt. 7500–8000 ft.; *C.B. Clarke s.n.*

*Avenastrum asperum* (Munro ex Thwaites) Vierh. var. *schmidii* (Hook. f.) C.E.C. Fisch., Fl. Madras 3(10): 1802. 1934

**Distribution.** Tamil Nadu [China].

**Habit.** Perennial.

**Remarks.** Taxonomic editor Soreng suggests placing *Helictotrichon schmidii* (Hook. f.) Henrard in *Tzveleviochloa* so the combination is made here.

### Subfamily Aristidoideae Caro, 1982

Annuals or perennials, generally with slender leaves. The spikelets have one flower subtended by glumes that are generally longer than the flower, and may be cylindrical or laterally compressed. Lemmas are terete and generally leathery, bearing three awns; the latter condition is synapomorphic for the subfamily. Awns may be free or fused.

While plants in Aristidoideae appear similar to Stipeae, many morphological characters keep them out of the Pooideae (e.g., presence of mcirohairs, lack of an epiblast in the embryo, C4 photosynthesis in two of the genera (Kellogg 2015b; Kellogg and Campbell 1987)). Molecular data place the subfamily firmly in the PACMAD clade (Barker et al. 1995; Clark et al. 1995; Grass Phylogeny Working Group 2001; Grass Phylogeny Working Group II 2012). The position of the subfamily within PACMAD is unclear. While GPWG II (Grass Phylogeny Working Group II 2012) placed it sister to all the remaining PACMAD subfamilies (i.e. PCMAD), Cotton et al. (2015) place it sister to the clade comprised of Chloridoideeae+Danthonioideae+Arundinoideae+Micrairoideae. Teisher et al. (2017) and Saarela et al. (2018) find that placement of the clade is ambiguous, with nearly equal support for placement sister to PCMAD, sister to CMAD, or sister to Panicoideae.

The three genera of Aristidoideae are each monophyletic, with *Sartidia* sister to *Stipagrostis* and *Aristida* sister to the two together (Cerros Tlatilpa et al. 2011).

***Aristida* L., Sp. Pl. 1: 82. 1753**

**Type. ***Aristida adscensionis* L.

**562. **Aristida adscensionis** L., Sp. Pl. 1: 82. 1753 **

*Aristida divaricata* J. Jacq., Ecl. Gram. Rar. 7. t. 6. 1813

*Chaetaria adscensionis* (L.) P. Beauv., Ess. Agrostogr. 151, 158. 1812 (as “ascensionis”)

*Aristida adscensionis* L. var. *pumila* (Decne.) Coss. & Durieu, Expl. Sci. Algerie 84. 1855

*Aristida pumila* Decne. Ann. Sci. Nat., Bot. Ser. 2, 4: 85. 1835. Type: “Hab. l’Hedjas” (P).

*Aristida bromoides* Kunth, Nov. Gen. Sp. [H.B.K.] 1: 122 (ed. qto.). 1816. Type: “Crescit in montanis regni Quitensis, Juxta Tambo de Guamote et Lianos de Tiocaxas, alt. 1600 hexap.” Ecuador: Chimborazo, Guamote; *Humboldt & Bonpland 3020 *(P).

*Chaetaria bromoides* (Kunth) Roem. & Schult., Syst. Veg., ed. 15 bis [Roemer & Schultes] 2: 396. 1817

*Aristida canariensis* Willd., Enum. Pl. (Willdenow) 1: 99. 1809. Type: “Habitat in Canariis.” Canary Islands: Habitat in Teneriffe; *Broussonet s.n.*

*Aristida coarctata *Kunth, Nov. Gen. Sp. [H.B.K.] 1: 122 (ed. qto.). 1816. Type: “Crescit in alto planitie Mexicana, inter Burras et Guanaxuato, alt. 1060 hexap.” Lectotype: Mexico: Guannajuato, between Burras and Guanaxuato; *Humbold & Bonpland s.n.* (P-Bonpl. (left-hand plant). LT designated by Hitchcock in Contr. U.S. Natl. Herb. 22: 542. 1924.

*Aristida curvata* Nees ex A. Rich. var. *abyssinica* A. Rich., Tent. Fl. Abyss. 2: 392. 1850–1851. Type: “Crescit in montibus prope Tchelatchranne et in provincial Chire mense October (Schimper).”

*Aristida debilis* Mez, Repert. Spec. Nov. Regni Veg*.* 17: 151. 1921. Lectotype: Jamaica; *Macnab s.n. *(B; ILT: US-81060 (fragm. ex B)). LT designated by Hitchcock in Contr. U.S. Natl. Herb. 22: 543. 1924.

*Aristida depressa* Retz., Observ. Bot. (Retzius) 4: 22. 1786–1787. Type: “Habitantem invenit honor, Koening in fterilioribus Malabariae cum priori.” India, Malabariae; *Koenig s.n.* (S).

*Aristida fasciculata* Torr., Ann. Lyceum Nat. Hist. New York 1(1): 154–155. 1824. Type: “Habitat in the forests of the Canadian river.” USA: New Mexico, in forests of the Canadian river; 1819–1820; *James s.n. *(NY).

*Chaetaria fasciculata* (Torr.) Schult. & Schult. f., Mant. 3 (Schultes & Schultes f.) Add. I 578. 1827

*Aristida grisebachiana* E. Fourn., Mexic. Pl. 2: 78. 1886. Lectotype: Mexico: Veracruz, Mirador; *Schaffner 175 (pl. ed. Hohen. 175) pro parte* (P). LT designated by Hitchcock in Contr. U.S. Natl. Herb. 22: 543. 1924.

*Aristida heymannii* Regel, Trudy Imp. S.-Peterburgsk. Bot. Sada 7: 649. 1881 (as “heymanni”). Type: “In valle fluvii Iii in angustiis Koibin.”

*Aristida interrupta* Cav., Icon. (Cavanilles) 5(1): 45. t. 471. f. 2. 1799. Type: “Habitat prope oppidium Chalma, Regni mexicani. Floret Augusto. Vidi siccam in laudato herbario.”

*Aristida maritima* Steud., Syn. Pl. Glumac. 1: 137. 1854. Type: Guadeloupe: sabulosis aridis maritimis; 1852; *E.P. de F. Duchassaing s.n*. (P).

*Aristida mauritiana* Hochst. ex A. Rich., Tent. Fl. Abyss. 2: 392. 1850–1851. Type: “Crescit ad pagum Aitet, in provincial Medat, mense Martio (Schimper).” Lectotype: Mauritius:* Anonymous s.n.* [Insula franciae ex Museo Paris 1820] (B). LT designated by Henrard in Meded. Rijks-Herb. 54(A): 338. 1927.

*Aristida modatica* Steud., Syn. Pl. Glumac. 1: 139. 1854. Type: Provincia Modat Abyss.; *Hochstetter herb. Abyss. un. it. nr. 1047*

*Aristida vulgaris *Trin. & Rupr.* var. mongholica* Trin. & Rupr., Sp. Gram. Stipac. 133. 1842. Type: “In arenosis et plnitiebus deserti totius Mongholiae mediae.”

*Aristida nana* (Trin. & Rupr.) Steud., Syn. Pl. Glumac. 1: 137. 1854

*Aristida dispersa* Trin. & Rupr. var. *nana* Trin. & Rupr., Sp. Gram. Stipac. 129. 1842. Type: Chile: Valparaiso, in declivibus apricis collium Quillota; Sepember, 1829; *C.G. Bertero 994* (LE). LT designated by Hitchcock in Contr. U.S. Natl. Herb. 22: 542. 1924.

*Aristida nigrescens* J. Presl in C. Presl, Reliq. Haenk. 1(4–5): 223. 1830. Type: “Hab. in Mexico” Mexico; 1836; *Haenke s.n.* (PR).

*Aristida schaffneri* E. Fourn., Mexic. Pl. 2: 78. 1886. Lectotype: Mexico: Veracruz, Orizaba; *J.G. Schaffner 181*(P). LT designated by Hitchcock in Contr. U.S. Natl. Herb. 22: 543. 1924.

*Aristida teneriffae* Steud., Syn. Pl. Glumac. 1: 420. 1854. Type: Canary Islands: Teneriffe; *Boivin 222*.

*Aristida vulgaris* Trin. & Rupr., Sp. Gram. Stipac. 131. 1842. Type: Canary Islands, Teneriffe; *Broussonet s.n. *(B-W).

**Lectotype. ***Herb. Linn. No. 98.1* (LINN). LT designated by Hitchcock in Contr. U.S. Natl. Herb. 22: 541. 1924.

**Distribution.** Andhra Pradesh, Bihar, Daman and Diu, Goa, Gujarat, Karnataka, Kerala, Maharashtra, Madhya Pradesh, Odisha, Rajasthan, Tamil Nadu, Uttar Pradesh, Uttarakhand [Pantropical, nearly worldwide in tropic, subtropic and warm temperate regions].

**Habit.** Annual.

**563. **Aristida cumingiana** Trin. & Rupr., Sp. Gram. Stipac. 141. 1842 **

*Aristida delicatula* Hochst. ex A. Rich., Tent. Fl. Abyss. 2: 393. 1850–1851. Syntypes: “Crescit in locis arenosis et graminosis prope Kouaieta, in provincial Chire (Quartin Dillon et Schimper).” Ethiopia; *Quartin Dillon s.n. *(P). Ethiopia: Schire; October 16, 1840; *Schimper 1830* (P).

*Aristida trichodes* (Nees ex Lindl.) Walpers, Ann. Bot. (Oxford) 3: 753. 1853

*Chaetaria trichodes* Nees ex Lindl., Hooker’s J. Bot. Kew Gard. Misc*.* 2: 101. 1850. Type: Insulae Philippinae; *H. Cuming 671* (B).

*Aristida capillacea* sensu Cav., Icon. (Cavanilles) 5(1): 43. t. 468. f. 1. 1799, non Lam., 1791.

**Type.** Philippine Islands: Luzon; *H. Cuming 671* (LE).

**Distribution.** Bihar, Chhattisgarh, Jharkhand, Madhya Pradesh, Odisha, West Bengal [China, Myanmar, Nepal.

**Habit.** Annual.

**564. **Aristida cyanantha** Nees ex Steud., Syn. Pl. Glumac. 1: 141. 1854 **

**Type.** Nepal; *Royle 64* (LE).

**Distribution.** Jammu and Kashmir, Punjab, Madhya Pradesh, Uttar Pradesh, Uttarakhand [Afghanistan, Nepal, Pakistan].

**Habit.** Perennial.

**565. **Aristida funiculata** Trin. & Rupr., Sp. Gram. Stipac. 159. 1842. **

*Aristida funiculata* Trin. & Rupr. var. *mallica* (Edgew.) Henrard, Mon. Aristida 90. 1929

*Aristida mallica* Edgew., J. Proc. Linn. Soc., Bot*.* 6: 209. 1862. Type: Multan, not cited.

*Aristida funiculata* Trin. & Rupr. var. *royleana* (Trin. & Rupr.) Hook. f., Fl. Brit. India (J.D. Hooker). 7(22): 227. 1896

*Aristida royleana* Trin. & Rupr., Sp. Gram. Stipac. 160. 1842. Type: India orientalis; 1836; *Royle s.n.* [Royle herb. Ind. Mont. (Nees herb.)] (LE-TRIN-1341.01).

*Aristida macrathera* A. Rich., Tent. Fl. Abyss. 2: 393. 1850–1851. Type: “Crescit in locis arenosis provinciae montosae Chire, mense Octobre (Quartin Dillon).” Ethiopia: Schire; *Quartin Dillon s.n.* (P).

*Aristida kotschyi* Hochst. ex Steud., Syn. Pl. Glumac. 1: 142. 1854, pro. syn. nom. nud. Type: Sudan: Kordofan, Abu-Gerad; September 21, 1839; *Kotschy 31*(WAG).

*Aristida stipacea* Ehrenb. & Hempr. ex Trin. & Rupr., Sp. Gram. Stipac. 159. 1842, nom. nud., cited as synonym of *Aristida funiculata*

*Aristida funicularis* Trin. ex Steud., Nomencl. Bot., ed. 2 (Steudel) 1: 131. 1840, nom. nud.

**Syntypes.** Africa: Senegal, in sabulosis prope Walo; *Leprieur s.n.* Sudan: ad pagum Cordofanum Abu-Gerard locis demissis arenosis siccis; *Kotschy 34 *(LE). Sudan: in planitie arenosa Accabae; *Kotschy 98 *(LE). Arabia: prope Gidon; *Ehrenberg s.n. *(LE).

**Distribution.** Andhra Pradesh, Bihar, Daman and Diu, Goa, Gujarat, Karnataka, Maharashtra, Madhya Pradesh, Odisha, Punjab, Rajasthan, Tamil Nadu, Uttar Pradesh [Myanmar, Pakistan].

**Habit.** Annual.

**566. **Aristida hystricula** Edgew., J. Proc. Linn. Soc., Bot. 6: 208. 1862 **

**Type.** India: Sindh and Multan; *Stocks 187*, p.p.

**Distribution.** Gujarat, Punjab, Rajasthan [Pakistan].

**Habit.** Annual.

**567. **Aristida hystrix** L.f., Suppl. Pl. 113. 1782 (“1781”) **

*Chaetaria hystrix* (L.f.) P. Beauv., Ess. Agrostogr. 152, 158. 1812

*Aristida rigescens* Roem. & Schult., Syst. Veg., ed. 15 bis [Roemer & Schultes] 2: 400. 1817. Type: India Orientalis; *Heyne s.n.*

*Aristida rigida* Roth, Nov. Pl. Sp. 42. 1821, non Cav., 1799. Type: “In collectione veneratiss, Benjamin Heyne innominata aderat.”

**Type.** “Habitat in Malabaria”; *Koenig s.n.*

**Distribution.** Andhra Pradesh, Bihar, Daman and Diu, Goa, Gujarat, Karnataka, Kerala, Madhya Pradesh, Maharashtra, Odisha, Rajasthan, Tamil Nadu [Myanmar, Sri Lanka].

**Habit.** Perennial.

**568. **Aristida mutabilis** Trin. & Rupr., Sp. Gram. Stipac. 150–151. 1842 **

*Aristida articulata* Edgew, J. Proc. Linn. Soc., Bot. 6: 209. 1862. Type: “The habit is that of rigescens (R.S., Steud. Gr. no. 100, p. 141).”

*Aristida longeradiata* Steud., Syn. Pl. Glumac. 1: 140. 1854. Type: Senegal; 1830; *Leprieur 92* (P).

**Type.** Africa: Sudan, “Cordofan, in arenae saxis graniticis delitescantibus formata”; *Kotschy 103* (LE).

**Distribution. **Andhra Pradesh, Himachal Pradesh, Karnataka, Maharashtra, Punjab, Rajasthan, Tamil Nadu [Pakistan].

**Habit. **Annual.

**569. **Aristida redacta** Stapf, Bull. Misc. Inform. Kew 1892: 85–86. 1892 **

*Stipa aristoides* Stapf ex Lisboa, J. Bombay Nat. Hist. Soc. 7: 358. 1893. Type: India: Deccan, Burdwan; *Lisboa s.n.?*

**Type.** India: “Bombay Presidency, Western Ghats, Eastern Chuttanagpur”, Deccan; May 1880; *G.M. Woodrow 19=124*(K (photo, US-1447177)). LT designated by Henrard in Meded. Rijks-Herb. 54(B): 505. 1928.

**Distribution.** Andhra Pradesh, Bihar, Chhattisgarh, Gujarat, Jharkhand, Karnataka, Maharashtra, Madhya Pradesh, Odisha, Rajasthan, Tamil Nadu, West Bengal.

**Habit.** Annual.

**570. **Aristida setacea **Retz., Observ. Bot. (Retzius) 4: 22. 1786–1787 **

*Chaetaria setacea* (Retz.) P. Beauv., Ess. Agrostogr. 152, 158. 1812

*Aristida quinqueseta* Steud., Syn. Pl. Glumac. 1: 420. 1855. Type: Insula Mauritii; *Boivin s.n.*

*Aristida coerulescens* sensu Thwaites, Enum. Pl. Zeyl. (Thwaites). 370. 1864, non Desf., 1798

**Type.** “In aridis sterilioribus Malabaricae cel. Koenig, unde sub nomine aristidae arundinaceae misit.” India: Malabaricae; *Koenig s.n. *(LD).

**Distribution.** Andhra Pradesh, Bihar, Chhattisgarh, Delhi, Gujarat, Himachal Pradesh, Karnataka, Kerala, Madhya Pradesh, Maharashtra, Odisha, Rajasthan, Tamil Nadu, Uttar Pradesh, West Bengal [Myanmar, Sri Lanka].

**Habit.** Perennial.

**571. **Aristida stocksii** (Hook. f.) Domin., Biblioth. Bot. 20 (Heft. 85): 338. 1915 **

*Aristida funiculata* Trin. & Rupr. var. *stocksii* Hook. f., Fl. Brit. India (J.D. Hooker). 7(22): 227. 1896. Type: India: Concan; *J.E. Stocks s.n.* (K).

**Distribution.** Bihar, Karnataka, Maharashtra.

**Habit.** Annual.

***Stipagrostis* Nees, Linnaea 7: 290. 1832**

**Type. ***Stipagrostis capensis* Nees.

**572. **Stipagrostis hirtigluma** (Steud. ex Trin. & Rupr.) De Winter, Kirkia 3: 134. 1963 **

*Aristida hirtigluma* Steud. ex Trin. & Rupr., Mém. Acad. Imp. Sci. Saint-Pétersbourg, Sér. 6, Sci. Math., Seconde Pt. Sci. Nat. 5: 171. 1842. Type: “In rupibus Wadi Hebran Arabiae petraeae m. April (Schimper Un. it. n. 165).” Lectotype: *Schimper 165* (B). LT designated by Henrard in Meded. Rijks-Herb. 54: 232. 1927.

**Distribution.** Rajasthan [also in Saudi Arabia, Africa]. Native or naturalized?

**Habit.** Annual or perennial.

**573. **Stipagrostis paradisea** (Edgew.) De Winter, Kirkia 3: 135. 1963 **

*Aristida paradisea* Edgew., J. Asiat. Soc. Bengal 16: 1219. 1847. Type: “Aden” Arabia.

**Distribution.** Rajasthan [also in the Middle East, Africa]. Native or naturalized?

**Habit.** Perennial.

**574. **Stipagrostis plumosa** (L.) Munro ex T. Anderson, J. Linn. Soc., Bot. 5(Suppl.)1: 40. 1860 **

*Aristida plumosa* L., Sp. Pl., ed. 2. 2: App. 1666. 1763. Type: “Habitat in America. D. Schreber.” Lectotype: Schreber, Herb. Linn. No. 98.6 (LINN). LT designated by Henrard in Meded. Rijks-Herb. 54A: 451. 1927.

**Distribution.** Rajasthan [Afghanistan, Pakistan].

**Habit.** Perennial.

**575. **Stipagrostis pogonoptila** (Jaub. & Spach) De Winter, Kirkia 3: 135. 1963 **

*Arthratherum pogonoptilum* Jaub. & Spach, Ill. Pl. Orient. 4: 56. 1851. Type: “Pentapotamide (collibus inter Jalalpur et Darapour).” India; April, 1831; *Jacquemont s.n.* (P).

*Aristida pogonoptila* (Jaub. & Spach) Boiss., Fl. Orient. [Boissier] 5: 496. 1884

**Distribution.** Haryana, Meghalaya, Punjab, Rajasthan [Pakistan].

**Habit. **Not reported.

**Remarks.** Records from Haryana and Punjab fide Naithani (1990).

**576. **Stipagrostis pungens** (Desf.) De Winter, Kirkia 3: 135. 1963 **

*Aristida pungens* Desf., Fl. Atlant. 1: 109. t. 35. 1798. Type: “Habitat in arenas humidis prope Sfax et in deserto.”

**Distribution.** India [Africa]. Possibly naturalized; state localities not reported.

**Habit.** Perennial.

### Subfamily Panicoideae A. Braun, 1864

Annuals or perennials, caespitose, rhizomatous, or stoloniferous, often with branched culms. Leaves, inflorescences, and spikelets variable. Lodicules 2, thick, fleshy. *Caryopsis with a punctate hilum*. Embryo with a scutellar cleft and *with the embryonic leaf margins overlapping*.

This large subfamily includes more than a quarter of the species in the family and in its current circumscription encompasses a broad range of variation. Much of this variability is due to the diverse morphology of the tribes Centotheceae, Chasmanthieae, and Tristachyideae, which have only recently been included as part of Panicoideae (Morrone et al. 2012; Sánchez-Ken and Clark 2010). In contrast, the core Panicoideae includes Paspaleae, Andropogoneae, and Paniceae; together this group has been recognized since the work of Robert Brown (1810; 1814). Members of the core all have two flowered spikelets which are dorsiventrally compressed. The distal flower is generally bisexual and the proximal one is either staminate or sterile. Disarticulation is below the glumes. All these spikelet characters appear to be synapomorphic for the clade comprising these three large tribes (Kellogg 2015b).

Taxonomic editor: Fernando Zuloaga for all except Andropogoneae

### Incertae sedis:

***Dichaetaria* Nees, Syn. Pl. Glumac. (Steudel) 1: 145. 1854**

**Type. ***Dichaetariawightii* Nees.

**577. **Dichaetaria wightii** Nees, Syn. Pl. Glumac. (Steudel) 1: 145–146. 1854 **

*Gymnopogon wightii* (Nees) Koord., Exkurs.-Fl. Java 1: 152. 1911

*Gymnopogon rigidus* Thwaites, Enum. Pl. Zeyl. (Thwaites). 372. 1864. Type: Sri Lanka: Hotter and drier parts of the Island; *Thwaites Ceylon Plant 914*

**Type.** India; *Wight Herb. no. 1035.*

**Distribution.** Kerala, Tamil Nadu [Sri Lanka].

**Habit.** Perennial.

### Tribe Centotheceae Ridl., 1907

Plants annual or perennial. Spikelets laterally compressed, with few to many flowers, the reduced flowers either proximal or distal to the fertile ones.

Members of this tribe have no obvious morphological features in common (Kellogg 2015b), but the group is strongly supported as monophyletic in molecular phylogenies (Burke et al. 2016; Grass Phylogeny Working Group II 2012; Sánchez-Ken and Clark 2007).

***Centotheca* Desv., Nouv. Bull. Sci. Soc. Philom. Paris 2: 189. 1810 (“Centosteca”), nom. & orth. cons.**

**Type. ***Centotheca lappacea* (L.) Desv. (*Cenchrus lappaceus* L.).

**578. **Centotheca ganeshaiahiana** M.V. Ramana, Chorghe, Prasanna, & Sanjappa, Nordic J. Bot. 32: 559. 2014. **

**Type.** India, north Andaman Islands Saddle Peak National Park. Open scrub forest (13°9'35.4"N, 93°11'09.3"E), 250–400 m a.s.l., 19 Feb 2011, *M.V. Ramana 0078* (holotype: CAL, isotypes: BSID, PBL).

**Distribution.** Andaman and Nicobar Islands.

**Habit.** Annual.

**579. **Centotheca lappacea** (L.) Desv., Nouv. Bull. Sci. Soc. Philom. Paris. 2: 189. 1810 var. **lappacea

*Cenchrus lappaceus* L., Sp. Pl., ed. 2. 2: 1488. 1763. Type: “Habitat in India.” Neotype: *Herb. Linn. No. 1212.15* (LINN). LT designated by Monod de Froideville in Blumea 19: 59. 1971.

*Melica lappacea* (L.) Raspail, Ann. Sci. Nat. (Paris) 5: 443. 1825

*Uniola lappacea* (L.) Trin., Mém. Acad. Imp. Sci. St.-Pétersbourg, Sér. 6, Sci. Math. 1(4): 358. 1830

*Centotheca latifolia* Trin., Fund. Agrost. (Trinius) 141. 1820, nom. superfl. & illeg. for *Cenchrus lappaceus *L.

*Centotheca parviflora* Andersson, Naturw. Reise Mossambique 560. 1864. Type: “Standort Auf feuchtem, sandigem und morastigem Wiesengrunde in Boror (und am Zambeze) in Marz.”

*Festuca blepharophora* Roem. & Schult., Syst. Veg., ed. 15 bis [Roemer & Schultes] 2: 728. 1817. Type: India orientalis.

*Holcus latifolius* Osbeck, Dagb. Ostind. Resa 247. 1757. Type: China.

*Torresia latifolia* (Osbeck) P.Beauv. ex Roem. & Schult., Syst. Veg., ed. 15 bis [Roemer & Schultes] 2: 515. 1817.

*Melica diandra* Roxb., Fl. Ind. (Carey & Wallich ed.) 1: 329. 1820. Type: “A Native of Mountains on the Coromandel Coast.”

*Melica refracta* Roxb., Fl. Ind. (Carey & Wallich ed.) 1: 329. 1820. Type: “Native of Moluccas and from thence introduced amongst other plants, into the Botanical Garden.”

*Poa latifolia* G. Forst., Fl. Ins. Austr. 8. 1786. Type: Society Islands; 1773 or 1774; *Forster s.n.*

*Torresia biflora* Roem. & Schult., Syst. Veg., ed. 15 bis [Roemer & Schultes] 2: 515. 1817, nom. nud., pro syn. as “Torresiam biflorum”.

*Panicum festuciforme* Hochst. ex Hook. f., Fl. Brit. India (J.D. Hooker). 7(22): 332. 1896, nom. nud.

*Festuca ciliaris* Heyne ex Roem. & Schult., Syst. Veg., ed. 15 bis [Roemer & Schultes] 2: 728. 1817, nom. nud., pro. syn.

*Festuca latifolia* Roth, Nov. Pl. Sp. 75. 1821, non DC., 1813

**Distribution. **Andaman and Nicobar Islands, Andhra Pradesh, Arunachal Pradesh, Assam, Bihar, Goa, Karnataka, Kerala, Madhya Pradesh, Maharashtra, Manipur, Meghalaya, Nagaland, Odisha, Punjab, Rajasthan, Sikkim, Tamil Nadu, Tripura, Uttar Pradesh, West Bengal [Bhutan, China, Myanmar, Nepal, Sri Lanka].

**Habit.** Perennial.

**580. **Centotheca lappacea** (L.) Desv. var. **longilamina** (Ohwi) Bor, Grasses Burma, Ceylon, India & Pakistan 459. 1960 **

*Centotheca longilamina* Ohwi, Bull. Tokyo Sci. Mus. 18: 10. 1947. Type: Indonesia: Java, Pasir Kiara Djongkang in Res. Batavia; June 8, 1924; *Bakhuizen van der Brink 3312* (BO).

**Distribution.** Andaman and Nicobar Islands (Naithani), Assam, Goa, Kerala, Maharashtra, Manipur, Meghalaya, Rajasthan, Sikkim, Tamil Nadu, West Bengal [China, Myanmar, Sri Lanka].

**Habit.** Perennial.

**Remarks.** Record from Andaman and Nicobar Islands fide Naithani (1990). Reports from Andhra Pradesh and Karnataka are erroneous.

***Thysanolaena* Nees, Edinburgh New. Philos. J. 18: 180. 1835**

**Type. ***Thysanolaena agrostis* Nees, nom. illeg. (*Agrostis maxima* Roxb., *Thysanolaena maxima* (Roxb.) Kuntze).

**581. **Thysanolaena latifolia** (Roxb. ex Hornem.) Honda, J. Fac. Sci. Univ. Tokyo, Sect. 3, Bot. 3: 312. 1930 **

*Melica latifolia* Roxb. ex Hornem., Suppl. Hort. Bot. Hafn. 117. 1819. Type: Habitat in Ind, orient. C. intr. 1818. Neotype: J*.O. Voigt - s.n*. (C/BC:C-10017246) [vide tropicos].

*Agrostis maxima* Roxb., Fl. Ind. (Carey & Wallich ed.) 1: 319. 1820. Type: A native of hedges, amongst the Mountains India, 1900–4000 ft.; *W. Roxburgh s.n.*

*Thysanolaena maxima* (Roxb. ex Hornem) Kuntze, Revis. Gen. Pl. 2: 794. 1891.

*Melica latifolia* Roxb. ex Hornem, Suppl. Hort. Bot. Hafn. 117. 1819; Roxb., Fl. Ind. (Carey & Wallich ed.) 1: 330. 1820. Type: “A native of the Garrow Hills, from thence it was brought to the Botanic Garden, by Mr. Robert Kyd.”

*Myriachaeta glauca* Moritzi ex Steud., Syn. Pl. Glumac. 1: 404. 1854. Type: Malaysia: Ins. Sümbaura; *Hrbo. Zolling. Nr. 1769*.

*Panicum acariferum* Trin., Sp. Gram. [Trinius] 1: t. 87. 1827.

*Thysanolaena assamensis* Gdger., Bull. Soc. Bot. France 66: 303. 1919. Type: Assam, Lashai Lungleh; *Gage 281*.

*Thysanolaena birmanica* Gdger., Bull. Soc. Bot. France 66: 303. 1919. Type: Birmania, ad Makhaye; *King 73*.

*Thysanolaena sikkimensis* Gdger., Bull. Soc. Bot. France 66: 303. 1919. Type: Himalaya: Sikkim, Labdah;* Prian s.n., Ribu s.n.*

*Thysanolaena agrostis* Nees, Edinburgh New Philos. J. 18: 180. 1835, nom. superfl. & illeg. for *Agrostis maxima* Roxb.

*Myriachaeta arundinacea* Zoll. & Moritz, Syst. Verz. (Moritzi et al.) 101. 1846, nom. superfl. & illeg. for *Myriachaeta glauca* Moritzi ex Steud.

**Distribution.** Andaman and Nicobar Islands, Arunachal Pradesh, Assam, Bihar, Himachal Pradesh, Jammu and Kashmir, Madhya Pradesh, Maharashtra, Manipur, Meghalaya, Mizoram, Nagaland, Odisha, Punjab, Tamil Nadu, Tripura, Uttarakhand, West Bengal [Bangladesh, Bhutan, China, Myanmar, Nepal, Sri Lanka].

**Habit.** Perennial.

### Tribe Chasmanthieae W.V. Br. & B.N. Smith ex Sánchez-Ken & L.G. Clark, 2010

Plants perennial, caespitose, rhizomatous or stoloniferous. Leaf blades broad, pseudopetiolate in some species. Spikelets laterally compressed, with one to many flowers. Stamens 1, 2, or 3.

Members of this tribe share no obvious morphological characters (Kellogg 2015b) but are supported as forming a clade in molecular phylogenies (Burke et al. 2016; Sánchez-Ken and Clark 2007; 2010).

***Lophatherum* Brongn., Voy. Monde, Phan. 49. 1831 (“1829”)**

**Type. ***Lophatherum gracile* Brongn.

**582. **Lophatherum gracile** Brongn., Voy. Monde, Phan. 2(2): 50, t. 8. 1831 var. **gracile

*Acroelytrum japonicum* Steud., Flora 29: 21. 1846. Type: “Ab omnibus Bambusaceis valvulis aristatis differt.”

*Allelotheca urvillei* Steud., Syn. Pl. Glumac. 1: 117. 1854. Type: “Ex herbario Urville sine addito loco natali. An. N. Holl.”

*Lophatherum annulatum* Franch. & Sav., Enum. Pl. Jap. 2: 605. 1879. Type: not given

*Lophatherum dubium* Steud., Syn. Pl. Glumac. 1: 300. 1854. Type: India; *Wight Herb. no. 1090*

*Lophatherum geminatum* Baker, J. Linn. Soc., Bot. 20: 300. 1883. Type: Central Madagascar; *Baron 1061 *(K).

*Lophatherum gracile* Brongn. var. *zeylanicum* (Hook. f.) Bor, Grasses Burma, Ceylon, India ad Pakistan 460. 1960

*Lophatherum zeylanicum* Hook. f. in Trimen, Handb. Fl. Ceylon 5: 303. 1900. Syntypes: Sri Lanka; *Thwaites Ceylon Plant 920*. Sri Lanka; *Walker s.n.* Pasdum Korale; *Gardner s.n.*

*Lophatherum humile* Miq., Prolus. Fl. Japon. 170. 1867. Type: Japan: Prope Nagasaki; 1862; *R. Oldham 948* (L). (vide Tropicos)

*Lophatherum lehmannii* Nees, Syn. Pl. Glumac. (Steudel) 1: 300. 1854. Type: Nepal; *Lehman s.n.*

*Lophatherum multiflorum* Steud., Syn. Pl. Glumac. 1: 300. 1854. Type: Java

*Lophatherum pilosulum* Steud., Syn. Pl. Glumac. 1: 428. 1855. Type: Japan; *I. Keiske s.n.* (L).

**Type.** “Amboine, dans les Moluques.”

**Distribution.** Arunachal Pradesh, Assam, Kerala, Meghalaya, Sikkim, Tamil Nadu [Bhutan, China, Myanmar, Nepal, Sri Lanka].

**Habit.** Perennial.

### Tribe Paspaleae J. Presl, 1830

Members of this tribe are morphologically disparate, with no obvious shared morphological character (Kellogg 2015b). Most species have a chromosome base number of *x *= 10 (Giussani et al. 2001).

***Axonopus* P. Beauv., Ess. Agrostogr. 12, 154. 1812**

**Lectotype. ***Axonopus compressus* (Sw.) P. Beauv. (*Milium compressum* Sw.). LT designated by Hitchcock in Contr. U.S. Natl. Herb. 12: 142. 1908; Chase, Proc. Biol. Soc. Wash. 24: 129. 1911.

**583. **Axonopus compressus** (Sw.) P. Beauv., Ess. Agrostogr. 154, 167. 1812 **

*Milium compressum* Sw., Prodr. (Swartz). 24. 1788. Type: Jamaica; *Swartz s.n.* India orientalis

*Paspalum compressum* (Sw.) Raspail, Ann. Sci. Nat. (Paris) 5: 301. 1825

*Paspalum platyculmum* Thouars ex Nees, Fl. Bras. Enum. Pl. 2(1): 24. 1829. Type: “Habitat in insula S. Mauritii.”

*Paspalum depressum* Steud., Syn. Pl. Glumac. 1: 20. 1853. Type: USA: Louisiana; *F.X. von Hartmann 51*(P).

*Paspalum filostachyum* A. Rich. ex Steud., Syn. Pl. Glumac. 1: 20. 1853. Type: West Indies; *F.W. Sieber 365* (P).

*Paspalum guadaloupense* Steud., Syn. Pl. Glumac. 1: 18. 1853. Type: Guadeloupe; *Duchaissing s.n*. (P).

*Paspalum platicaulon* Poir., Encycl. (Lamarck) 5: 34. 1804 [as “platycaule” in IPNI]

Type: “Cette espece a été recueillie a Porto Ricco, par le citoyen Ledru.” Puerto Rico; *A.P. Ledrú s.n.* (P-LAM).

*Anastrophus platycaulis* (Poir.) Nash, Fl. S.E. U.S. [Small]. 79, 1327. 1903

*Digitaria platicaulis* (Poir) Desv., Mém. Soc. Agric. Angers 62. 1831

*Panicum platicaulon* (Poir.) Kuntze, Revis. Gen. Pl. 3: 363. 1898 (as “Platycaulon”)

*Paspalum raunkiaerii* Mez, Repert. Spec. Nov. Regni Veg. 15: 60. 1917. Type: Antillarum insula St. Jan;* Raunkiaer 1313*

*Paspalum tristachyon* Lam., Tabl. Encycl. 1: 176. 1791. Type: “Ex America merid. Communic. D. Richard.” South America; *L.C.M. Richard s.n.* (P-LAM).

*Paspalum laticulmum* Spreng., Syst. Veg. (ed. 16) [Sprengel] 1: 245. 1824 (“1825”), nom. superfl. & illeg. for *Paspalum tristachyon.*

*Agrostis compressa* (Sw.) Poir., Encycl. (Lamarck) Suppl. 1: 259. 1810, non *Axonopus compressa* Willd., 1790

*Digitaria domingensis* Desv. ex Kunth, Enum. Pl. (Kunth) 1: 49. 1833, non Roem. & Schult., 1817

*Anastrophus compressus* sensu Schltr. ex Döll., Fl. Bras. (Martius) 2(2): 102. 1877, non Schltdl., 1850

**Distribution.** Andaman and Nicobar Islands, Andhra Pradesh, Arunachal Pradesh, Assam, Bihar, Himachal Pradesh, Karnataka, Kerala, Maharashtra, Meghalaya, Tamil Nadu, Uttarakhand, Uttar Pradesh, West Bengal [China, Pakistan].

**Habit.** Perennial.

**Remarks.** Records for Arunachal Pradesh, Assam, Kerala and Uttar Pradesh fide Naithani (1990).

**584. **Axonopus fissifolius **(Raddi) Kuhlm., Comiss. Linhas Telegr. Estratég. Matto Grosso Amazonas 67(11): 87. 1922 **

*Paspalum fissifolium *Raddi, Agrostogr. Bras. [Raddi] 26. 1823 (as ‘‘Paspalus fissifolius’’). Type: Brazil. Rio de Janeiro: Guanabara, s.d., *G. Raddi s.n.* (holotype, PI; isotypes, BM 678791, FI, FI fragm. in BAA 313, G, PI fragm. in US 2766195).

*Axonopus affinis* A. Chase, J. Wash. Acad. Sci. 28: 180. f. 1–2. 1938. Type: USA: Mississippi: Waynesboro, in low moist ground, 2 Oct. 1896, *T.H. Kearney 175.*

**Distribution.** Uttar Pradesh. Possibly naturalized; native to North America.

**Habit.** Perennial.

**Remarks.** Record for Uttar Pradesh fide Naithani (1990).

***Hildaea* C. Silva & R.P. Oliveira, Molec. Phylog. Evol. 93: 229. 2015**

**Type. ***Hildaea pallens* (Sw.) C. Silva & R.P. Oliveira (*Panicum pallens* Sw.).

**585. **Hildaea pallens** (Sw.) C. Silva & R.P. Oliveira, Molec. Phylog. Evol. 93: 229. 2015. **

*Panicum pallens* Sw., Prodr. (Swartz) 23. 1788. Lectotype: O.P. Swartz s.n.; Jamaica, designated by Stieber, Syst. Bot. 12: 203 (1987)

*Ichnanthus pallens* (Sw.) Munro ex Benth., Fl. Hongk. 414. 1861

*Panicum vicinum* F.M. Bailey, Syn. Queensl. Fl. Suppl. 3: 82. 1890. Type: Australia: Queensland, Harvey Creek, headwaters of Russell River; *Bailey 22* (BRI). Australia, Queensland, Harveys’s Creek, Russell River, on rich scrubland; *Bailey s.n*. (BRI).

*Ichnanthus vicinus* (F.M. Bailey) Merr., Philipp. J. Sci. 20(4): 367–368. 1922

*Panicum nitens* Merr., Publ. Bur. Sci. Gov. Lab. 17: 8. 1904, non K. Schum., 1901. Type: Philippines: Province of Bataan, Luzon, Mount Mariveles; January 1, 1904; *Merrill 3756*

**Distribution.** Andaman and Nicobar Islands, Andhra Pradesh, Assam, Delhi, Kerala, Nagaland, Odisha, Tamil Nadu [Sri Lanka], Andaman and Nicobar Islands. Pantropical.

**Habit.** Perennial.

**Remarks.**Chorge et al. (2013) indicate that *Hildaea. pallens *var*. majus* (Nees) Stieber (as *Ichnanthus pallens*) is widely distributed in India and is synonymous with *I. vicinus*. *Hildaea. pallens* var. *pallens* is only recorded from the Andaman Islands; this variety is primarily neotropical with sporadic records from Southeast Asia.

***Hymenachne* P. Beauv., Ess. Agrostogr. 48, 165. 1812**

**Lectotype. ***Hymenachne myuros* (Lam.) P. Beauv. (*Panicum myuros* Lam. (“myruos”)) LT designated by L. K. G. Pfeiffer, Nom. 1: 1702. 1873.

**586. **Hymenachne amplexicaulis** (Rudge) Nees, Fl. Bras. Enum. Pl. 2(1): 276. 1829 **

*Panicum amplexicaule* Rudge, Pl. Guian. 1: 21. t. 27. 1805. Type: Guianas; *Rudge s.n.* French Guiana; *Martin s.n.*

*Hymenachne pseudointerrupta* Müll. Hal., Bot. Zeitung (Berlin) 19(45): 333. 1861. Type: India orientalis, Bengalia et Malacca; *Griffith s.n.*

*Panicum myuros* sensu Hook. f., Fl. Brit. India (J.D. Hooker). 7(21): 39. 1896, non Lam., 1791

*Hymenachne acutigluma* (Steud.) Gilliland, Gard. Bull. Singapore 20(4): 314. 1964

*Panicum acutiglumum* Steud., Syn. Pl. Glumac. 1: 66. 1853. Type: Philippine Islands; *Cuming 2287* (P).

**Distribution.** Andhra Pradesh, Assam, Bihar, Karnataka, Kerala, Maharashtra, Meghalaya, Odisha, Rajasthan, Tripura, Uttar Pradesh, West Bengal [China, Myanmar]. Pantropical, possibly native to the Americas.

**Habit.** Perennial.

**587. **Hymenachne assamica** (Hook. f.) Hitchc., Lingnan Sci. J. 7: 222. 1931 (as “assamicum”) **

*Panicum assamicum* Hook. f., Fl. Brit. India (J.D. Hooker). 7(21): 40. 1896. Syntypes: India: Assam;* Masters s.n., Griffith s.n.* Dacca; *C.B. Clarke s.n.*

**Distribution.** Assam [China].

**Habit.** Perennial.

**Remarks.** Taxonomic editor Zuloaga questions whether this species actually occurs in India.

**588. **Hymenachne aurita** (J. Presl. ex Nees) Balansa, J. Bot. (Morot) 4: 144. 1890 **

*Panicum auritum* J. Presl. ex Nees, Fl. Bras. Enum. Pl. 2(1): 176. 1829. Lectotype: Philippines: “Luzon”; *Haenke s.n. *(PR). LT designated by Veldkamp in Blumea 41(1): 187. 1996

*Sacciolepis aurita* (J. Presl. ex Nees) E.G. Camus & A. Camus, Fl. Indo-Chine [P.H. Lecomte et al.] 7: 459. 1922

*Panicum insulicolum* Steud., Syn. Pl. Glumac. 1: 78. 1853 (as “insulicola”). Type: Indonesia: Java, Sri Lanka

*Sacciolepis insulicola* (Steud.) Ohwi, Bull. Tokyo Sci. Mus. 18: 3. 1947

*Panicum javanum* Nees ex Buse, Pl. Jungh. 376. 1854, non poir, 1816. Lectotype: *Junghuhn s.n.(*L-sh908.91-2190). LT designated by Veldkamp in Blumea 41(1): 187. 1996.

**Distribution.** Andaman and Nicobar Islands, Assam, Bihar, Karnataka, Kerala, Nagaland, Sikkim, Tripura, West Bengal [China, Myanmar].

**Habit.** Perennial.

**589. **Hymenachne panduranganii** J. Remya & Geethakum., Nordic. J. Bot. 35(2): 201–204, f. 1–2. 2016. **

**Type.** India, Kerala, Wayanad district, Thirunelli, at 900 m a.s.l., 11 November 2014, Remya J 83272 (holotype: TBGT, isotypes: MH, CAL).

**Distribution.** Kerala.

**Habit.** Perennial

***Paspalum* L., Syst. Nat., ed. 10. 2: 855, 1359. 1759**

**Lectotype. ***Paspalum dimidiatum* L., nom. illeg. (*Panicum dissectum* L., *Paspalum dissectum* (L.) L.). Designated by Nash in N. Amer. Fl. 17: 165. 1912; affirmed by Hitchcock, Bull. U.S.D.A. 772: 225. 1920.

**590. **Paspalum canarae** (Steud.) Veldkamp, Blumea 21(1): 72. 1973 var. **canarae

*Panicum canarae* Steud., Syn. Pl. Glumac. 1: 58. 1853. Type: India: Canara Province; *R.F. Hohenacker 635*

*Panicum compactum* auct. non Roth ex Roem. & Schult., 1817: Hook. f., Fl. Brit. India (J.D. Hooker). 7(21): 12. 1896

**Distribution.** Andhra Pradesh, Karnataka, Kerala, Madhya Pradesh, Maharashtra, Odisha, Tamil Nadu.

**Habit.** Annual.

**591. **Paspalum canarae** (Steud.) Veldkamp var. **fimbriatum** (Bor) Veldkamp, Blumea 21(1): 72. 1973 **

*Paspalum compactum* Roth ex Roem. & Schult. var. *fimbriatum* Bor, Grasses Burma, Ceylon, India and Pakistan 336. 1960. Type: India: Maharashtra, Bombay; *Bole 305* (K).

**Distribution.** Karnataka, Kerala, Maharashtra.

**Habit.** Annual.

**Remarks.** Distribution in India fide Naithani (1990).

**592. **Paspalum conjugatum** P. J. Bergius, Acta Helv. Phys.-Math. 7: 129, t. 8. 1762 **

*Paspalum ciliatum* Lam., Tabl. Encycl. 1: 175. 1791. Type: French Guiana; *D. LeBlond s.n.* (P-LAM).

*Paspalum longissimum* Hochst. ex Steud., Syn. Pl. Glumac. 1: 19. 1853. Type: Surinam: in districtu Surinamense Para; February-April, 1844; *A. Kappler 1556 *(P).

*Paspalum renggeri* Steud., Syn. Pl. Glumac. 1: 17. 1853. Type: Paraguay; *J.R. Rengger s.n. *(P).

*Paspalum bicrurum* Saltzm. ex Döll, Fl. Bras. (Martius) 2(2): 55. 1877, nom. nud. cited as synonym of *Paspalum conjugatum*.

*Paspalum ciliatifolium* sensu Trin., Mém. Acad. Imp. Sci. Saint-Pétersbourg, Sér. 6, Sci. Math. Seconde Pt. Sci. Nat. 3, 1(2–3): 340. 1834, non Michx., 1803.

*Paspalum hirsutum* sensu Poir., Encycl. (Lamarck) 5: 28. 1804, non Retz. 1781.

**Type.** “Habitat in Surinamo.” France: Surinam; *Rolander in Herb. Bergius 36*(SBT). LT designated by de Koning & Sosef, Blumea 30: 290. 1985

**Distribution.** Andaman and Nicobar Islands, Andhra Pradesh, Arunachal Pradesh, Assam, Karnataka, Kerala, Maharashtra, Meghalaya, Odisha, Sikkim, Tamil Nadu, Tripura, Uttar Pradesh, West Bengal [China, Sri Lanka].

**Habit.** Perennial.

**593. **Paspalum dilat**atum Poir., Encycl. (Lamarck) 5: 35. 1804 **

**Type. **“Cette plante a été recueillie a Buenos-Ayres par Commerson.” Argentina: Buenos Aires; *P. Commerson s.n. *(P-LAM).

**Distribution.** Assam, Himachal Pradesh, Kerala, Maharashtra, Meghalaya, Nagaland, Rajasthan, Sikkim, Tamil Nadu, Uttar Pradesh, Uttarakhand, West Bengal [China, Sri Lanka]. Native to South America, introduced to North America, Europe and Aisa.

**Habit.** Perennial.

**Remarks.** Records for Meghalaya, Nagaland, and Uttar Pradesh fide Naithani (1990).

**594. **Paspalum distichum** L., Syst. Nat., ed. 10, 2: 855. 1759. **

*Paspalum paspalodes* (Michx.) Scribn., Mem. Torrey. Bot. Club 5(3): 29. 1894 (as “paspaloides”)

*Digitaria paspalodes* Michx., Fl. Bor.-Amer. (Michaux) 1: 46. 1803. Type: USA: “in pascuis aridis juxta Charslton”; *Michaux s.n.*(P-MICH).

**Type.** “Habitat [in Jamaica.]” LT: *P. Browne s.n*.; ; Jamaica (LINN-75.9, second fertile culm from the left) LT designated by Guedes, Taxon 25: 513 (1976), and earlier discussed by Jovet & Guedes, Taxon 21: 546 (1972); formally ruled on by Nom. Comm., Taxon 32: 281 (1983).

**Distribution.** Andaman and Nicobar Islands, Andhra Pradesh, Assam, Bihar, Gujarat, Haryana, Himachal Pradesh, Jammu and Kashmir, Karnataka, Kerala, Madhya Pradesh, Maharashtra, Meghalaya, Odisha, Punjab, Rajasthan, Tamil Nadu, Uttar Pradesh, West Bengal [Pakistan]. Pantropical.

**Habit.** Perennial.

**Remarks.** This species is often confused with *P. vaginatum*. Taxonomic editor Zuloaga notes that “Bor (1960) cited *P. distichum* in India and Pakistan, and included in the species several synonyms. Also he correctly differentiated *P. distichum* from *P. vaginatum* by a hairy upper glume in the former species.” Zuloaga concludes that it is likely that *P. distichum* is present in India.

**595. **Paspalum longifolium** Roxb., Fl. Ind. (Carey & Wallich ed.) 1: 283. 1820 var. **longifolium

*Paspalum scrobiculatum* var. *longifolium* (Roxb.) Domin, Biblioth. Bot. 85: 288. 1915

**Type.** “Of what country this is a native is uncertain. It appeared in the Botanic Garden in 1807.” India, Cult.; *Roxburgh s.n. *(BM).

**Distribution.** Andaman and Nicobar Islands, Assam, Bihar, Gujarat, Haryana, Kerala, Madhya Pradesh, Nagaland, Odisha, Tamil Nadu, Tripura, Uttar Pradesh, West Bengal [Bhutan, Myanmar, Nepal, Sri Lanka].

**Habit.** Perennial.

**Remarks.** Records from Assam, Gujarat, Haryana, Kerala, Madhya Pradesh, Nagaland, Uttar Pradesh, and Nepal fide Naithani (1990).

**596. **Paspalum longifolium** Roxb. var. **lorirhachis** Bor, Fl. Assam 5: 253. 1940 **

**Type.** India: Assam, Bhomraguri, Darrang; *N.L. Bor 78485* (DD).

**Distribution.** Assam.

**Habit.** Perennial.

**Remarks.** Record for Assam fide Naithani (1990).

**597. **Paspalum notatum** Fluegge, Gram. Monogr., Paspalum 106. 1810 (as “Paspalus notatus”) **

*Paspalum taphrophyllum* Steud., Syn. Pl. Glumac. 1: 19. 1853. Type: Martinica; *F.W. Sieber 365* (L).

**Type.** West Indies: Virgin Islands, St. Thomas; 1802; *Ventenat s.n.* (B).

**Distribution. **India. Native to North and South America.

**Habit.** Perennial.

**Remarks.** Distribution in India not recorded.

**598. **Paspalum plicatulum** Michx., Fl. Bor.-Amer. (Michaux) 1: 45. 1803 **

*Panicum plicatulum* (Michx.) Kuntze, Revis. Gen. Pl. 3: 363. 1898

*Paspalum antillense* Husn., Bull. Soc. Linn. Normandie ser. 2, 5: 260. 1870. Type: “Alt. 640–1300 ft.-Bords des chemins-Route de la Basse-Terre au camp Jacob (Gaud.)” Guadeloupe; *P.T. Husnot 76* (US-2854152 (fragm. ex BR)).

*Paspalum atrocarpum* Steud., Syn. Pl. Glumac. 1: 25. 1853. Type: “Ex. Hrbo. Urville sine loco natali.”

*Paspalum montevidense* Spreng., Syst. Veg. (ed. 16) [Sprengel] 1: 246. 1824 (“1825”). Type: Uruguay: Montevideo;* F. Sellow 670*

*Paspalum multiflorum* Desv., Opusc. Sci. Phys. Nat. 58. 1831.Type: “Crescit in Brasilio.” (P).

*Paspalum tenue* Kunth, Révis. Gramin. 1: 26. 1829

*Paspalum undulatum* Poir., Encycl. (Lamarck) 5: 29. 1804. Type: “Cette plante croit a Porto-Ricco per le citoyen Ledru.” Puerto Rico; *A.P. Ledrú s.n.* (P-LAM).

*Paspalum campestre* Schlecht., Linnaea 26: 131. 1853, non Trin., 1834

*Paspalum gracile* Leconte, J. Phys. Chim. Hist. Nat. Arts 91: 285. 1820, non Rudge, 1805. Type: America: Georgia

*Paspalum plicatum* sensu Pers., Syn. Pl. (Persoon) 1: 86. 1805 Michx., 1803

*Paspalum marginatum* sensu Spreng. ex Steud., Nomencl. Bot., ed. 2 (Steudel) 2: 272. 1841, non Trin., 1826

**Type.** USA: “Hab. in Georgia et Florida”; *Michaux s.n.* (P).

**Distribution.** India [China]. Naturalized. Native to tropical North and South America.

**Habit.** Perennial.

**Remarks.** Distribution in India not recorded.

**599. **Paspalum scrobiculatum** L., Mant. Pl. 29. 1767 var. **scrobiculatum

*Paspalum cartilagineum* J. Presl in C. Presl, Reliq. Haenk. 1(4–5): 216. 1830. Type: “Habitat in insula Luzonia ad Sorzogon, in Marianis” Lectotype: Philippines: Luzon, Sorzogon; 1792; *Haenke s.n. *(PR). (vide de Koning & Sosef, Blumea 30 305. 1985).

*Paspalum commersonii* Lam., Tabl. Encycl. 1: 175. t. 43. f. 1. 1791 (as “commerson”). Type: “Lieu nat. I’Isle de France.” Mauritius; *P. Commerson s.n.* (P-LAM).

*Paspalum orbiculare* G. Forst., Fl. Ins. Austr. 7. 1786. Type: Society Islands; *Forster s.n.*(GOET).

*Paspalum kora* Willd., Sp. Pl., ed. 4 [Willdenow] 1: 332. 1797, nom. superfl. & illeg. for *P. orbiculare*

*Paspalum scrobiculatum* L. var. *bispicatum* Hack., Allg. Bot. Zeitschr. 20:146. 1914.

**Type. **“Habitat in India orientali.” Lectotype: *Herb. Linn. No. 79.4* (LINN). LT designated by Clayton in Kew Bull. 30: 101. 1975.

**Distribution.** Andhra Pradesh, Assam, Chhattisgarh, Karnataka, Kerala, Madhya Pradesh, Maharashtra, Odisha, Punjab, Rajasthan, Tamil Nadu [Sri Lanka].

**Habit.** Perennial.

**Remarks.** Records for Assam and Maharashtra fide Naithani (1990).

**600. **Paspalum scrobiculatum** L. var. **auriculatum** (J. Presl) Merr., Philipp. J. Sci. 1(Suppl. 5): 345. 1906 **

*Paspalum auriculatum* J. Presl in C. Presl, Reliq. Haenk. 1(4–5): 217. 1830. Type: “Habitat in Luzonia ad Sorzogon” Lectotype: Philippines: Luzon, Sorzogon; *Haenke s.n.* (the 3 right-hand side specimens) (PR). (vide de Koning & Sosef, Blumea 30: 304. 1985).

**Distribution.** Andhra Pradesh, Karnataka, Tamil Nadu [also in the Philippines].

**Habit.** Perennial.

**Remarks. **Taxonomic editor Zuloaga notes that this taxon is native in other parts of Asia but might be naturalized or only cultivated in parts of India.

**601. **Paspalum scrobiculatum** L. var. **horneri** (Henrard) de Konnig & Sosef, Blumea 30(2): 307. f. 7B. 1985 **

*Paspalum horneri* Henrard, Blumea 1(2): 306. f. 2. 1935. Type: Sumatra: Prairien; *Horner s.n. *(L).

**Distribution.** Andhra Pradesh, Assam [also in Indonesia].

**Habit.** Perennial.

**Remarks.** Record for Assam fide Naithani (1990).

**602. **Paspalum thunbergii **Kunth, Révis. Gramin. 1(2): 25, no. 80. 1829 **

*Paspalum scrobiculatum* var. *thunbergii* (Kunth) Makino, Bot. Mag. (Tokyo) 10: 60. 1896

*Paspalum dissectum* sensu Thunb., Fl. Jap. (Thunberg) 45. 1784, non. (L.) L., 1762

*Paspalum thunbergii* Kunth ex Steud., Nomencl. Bot., ed. 2 (Steudel) 2(2): 273. 1841, isonym

**Distribution.** Sikkim, West Bengal [Bhutan, China].

**Habit.** Perennial.

**603. **Paspalum urvillei** Steud., Syn. Pl. Glumac. 1: 24. 1853 **

*Paspalum dilatatum *Poir. var. *parviflorum* Döll, Fl. Bras. (Martius) 2(2): 64. 1877. Syntypes: Brazil: Lagoa Santa; *E. Warming s.n.* (BR). Brazil: prope Pernambuco; *Forsell s.n.* Uruguay: Montevideo; *Sello s.n.* Rio de Janerio; *Glaziou 477*

*Paspalum larranagae* Arechav., Ann. Mus. Nac. Montevideo 1: 60 (reprint 68), t. 2. 1894 (as “larranagai”). Type: Uruguay: Salto, terrenos bajos humedos, viñedo de Harrigue, y en la Vitícola Saltena; March, 1894; *Arechavaleta 207 *(MVM)

*Paspalum ovatum* Nees ex Trin. var. *parviflorum* Nees, Fl. Bras. Enum. Pl. 2(1): 43. 1829 (as “parviflorus”). Type: Uruguay: Montevideo; *Sellow s.n.*

*Paspalum velutinum* Trin. ex Nees, Fl. Bras. Enum. Pl. 2(1): 43. 1829, nom. nud., pro syn.

**Type.** Brazil; 1825; *J.S.C. Dumont de Urville s.n.* (P).

**Distribution.** India [China, Sri Lanka]. Native to South America; naturalized in North America, Europe, and Asia.

**Habit.** Perennial.

**Remarks.** Distribution in India not recorded.

**604. **Paspalum vaginatum** Sw., Prodr. (Swartz) 21. 1788 (as “Paspatum”) **

*Paspalum distichum* L. var. *vaginatum* (Sw.) Griseb., Fl. Brit. W.I. (Grisebach) 541. 1864

*Sanguinaria vaginata* (Sw.) Bubani, Fl. Pyren. (Bubani) 4: 258. 1901

*Digitaria foliosa* Lag., Gen. Sp. Pl. (Lagasca) 4. 1816. Type: Cuba: “Habitat in Havana, ubi legit *D. Balth. Boldo*” (MA).

*Paspalum foliosum* (Lag.) Kunth, Révis. Gramin. 1: 25. 1829

*Paspalum inflatum* A. Rich. in Sagra, Hist. Fis. Cuba 11: 298. 1850. Type: Cuba: La Habana; *R. de la Sagra s.n. *(P).

*Paspalum brachiatum* Trin. ex Nees, Fl. Bras. Enum. Pl. 2(1): 62. 1829, nom. nud. pro. syn.

**Type.** Jamaica; *Swartz s.n.* (S).

**Distribution.** Andaman and Nicobar Islands, Bihar, Gujarat, Karnataka, Kerala, Madhya Pradesh, Rajasthan, Odisha, Tamil Nadu, Uttar Pradesh, West Bengal [China, Sri Lanka]. Pantropical.

**Habit.** Perennial.

### Tribe Paniceae R. Br., 1814

Members of this tribe are morphologically disparate, with no obvious shared morphological character (Kellogg 2015b; Zuloaga 1987; Zuloaga and Soderstom 1985). Most species have a chromosome base number of *x*=9 (Giussani et al. 2001).

Taxonomic editors for Paniceae: Fernando Zuloaga, P. (Parigi) Venkateswara Prasanna and Alok R. Chorghe.

***Acroceras* Stapf, Fl. Trop. Africa 9: 621. 1920**

**Lectotype. ***Acroceras oryzoides* (Sw.) Stapf (*Panicum oryzoides* Sw. 1788, non Arduino 1764), = *Acroceras zizanioides*. LT designated by E. Phillips in Gen. S. Afr. Fl. Pl. ed. 2., 103. 1951.

**605. **Acroceras munroanum** (Balansa) Henrard, Blumea 3(3): 444–445. 1940 **

*Panicum munroanum* Balansa, J. Bot. (Morot) 4(7): 140. 1890. Type: Sri Lanka; 1857; *Thwaites Ceylon Plant 3244*

*Panicum crassiapiculatum* Merr., Philipp. J. Sci. 1(Suppl. 5): 356. 1906. Type: Malaya Peninsula, Goping; *King’s Collector s.n.*

*Acroceras crassiapiculatum* (Merr.) Alston in Trimen, Handb. Fl. Ceylon 6: 324. 1931

*Panicum ridleyi* Hack. ex Ridl., Trans. Linn. Soc. London, Bot. Ser. 2. 3: 400. 1893, nom. nud.

*Panicum latifolium* sensu Hook. f., Fl. Brit. India (J.D. Hooker). 7(21): 39. 1896, non L., 1753

**Distribution.** Karnataka, Kerala [China, Myanmar, Sri Lanka].

**Habit. **Perennial.

**606. **Acroceras tonkinense** (Balansa) C.E. Hubb. ex Bor, Indian Forest Rec., Bot. n.s. 1(3): 78. 1938 **

*Panicum tonkinense* Balansa, J. Bot. (Morot) 4(7): 140. 1890. Syntypes: Vietnam: Tu-Phap, Ouonbi; May, 1887; *Balansa 1646*. Vietnam; Sepember 12, 1885; *Balansa 442*

*Neohusnotia tonkinensis* (Balansa) A. Camus, Bull. Mus. Natl. Hist. Nat. 26: 664. 1920

*Panicum latifolium* L. var. *majus* Hook. f., Fl. Brit. India (J.D. Hooker). 7(21): 39. 1896. Type: Malay Peninsula: Goping; *King’s Collector s.n.*

**Distribution.** Assam [Myanmar].

**Habit.** Perennial.

**607. **Acroceras zizanioides** (Kunth) Dandy, J. Bot. 69: 54. 1931 **

*Panicum zizanioides* Kunth, Nov. Gen. Sp. [H.B.K.] 1: 100 (ed. qto.). 1816. Type: Colombia; *Humboldt & Bonpland 1606* (B). “Crescit in calidissimis regni Novogranatensis, in ripa fluminis Magdalenae, inter Borjorque et Los Paxarales de Sogamozo, alt. 130 hexap.”

*Acroceras oryzoides* Stapf, Fl. Trop. Afr. [Oliver et al.] 9: 622. 1920

*Panicum balbisianum* Schult., Mant. 2 (Schultes) 254. 1824. Type: “In S. Domingo. D. Bertero, Panicum aturense Herb. Balbis n. 2578.”

*Panicum lutetense* K. Schum., Bot. Jahrb. Syst. 24: 332. 1897. Type: Zaire: Congogebiet, Lutete, Gebusch, Bachufern, alt. 16000 ft.; *Hens 194* (K).

*Panicum ogowense* Franch., Bull. Soc. Hist. Nat. Autun 8: 344–345. 1895. Syntypes: Congo Francais: “Region de l’Ogooué; *Thollon 727*, *Thollon 832.*” Congo belge: à Lutèté; *Fr. Hens 194*

*Panicum pseudoryzoides* Steud., Syn. Pl. Glumac. 1: 75. 1853. Type: Bahia

*Panicum oryzoides* Sw., Prodr. (Swartz) 23. 1788, non Ard., 1764. Type: Jamaica; *Swartz s.n.*

*Panicum latifolium* sensu Hook. f., Fl. Brit. India (J.D. Hooker). 7(21): 39. 1896, p.p., non L., 1753

**Distribution.** Assam [China].

**Habit.** Perennial.

**Remarks. **The taxonomic editors note that it is unclear if this species is naturalized (given the large number of New World species in the synonymy), cultivated, or native.

Alloteropsis **J. Presl, Reliq. Haenk. 1: 343. 1830 **

**Type.***Alloteropsis distachya* J. Presl.

**608. **Alloteropsis cimicina** (L.) Stapf, Fl. Trop. Afr. [Oliver et al.] 9: 487. 1919 **

*Milium cimicinum* L., Mant. Pl. Altera 184. 1771. Type: “Habitat in Malabariae et oppidi Johannis plateis. Koenig. 55.” Neotype: *Herb. Linn. No. 83.2 *(LINN). LT designated by Clayton & Renvoize in Polhill (ed.), Fl. Trop. E. Africa, Gramineae 3: 617. 1982.

*Axonopus cimicinus* (L.) P. Beauv., Ess. Agrostogr. 154. 1812.

*Panicum cimicinum* (L.) Retz., Observ. Bot. (Retzius) 3: 9. 1783.

*Urochloa cimicina* (L.) Kunth, Révis. Gramin. 1: 31. 1829

*Coridochloa cimicina* (L.) Nees ex A. Chase, Proc. Biol. Soc. Washington 24:129. 1911

**Distribution.** Andaman and Nicobar Islands, Andhra Pradesh, Bihar, Daman and Diu, Delhi, Goa, Gujarat, Haryana, Himachal Pradesh, Jammu and Kashmir, Karnataka, Kerala, Madhya Pradesh, Maharashtra, Odisha, Rajasthan, Tamil Nadu, Tripura, Uttar Pradesh, West Bengal [China, Myanmar, Sri Lanka].

**Habit.** Annual.

**609. **Alloteropsis semialata** (R. Br.) Hitchc., Contr. U.S. Natl. Herb. 12: 210. 1909 var. **semialata

*Panicum semialatum* R. Br., Prodr. Fl. Nov. Holland. 1: 192. 1810. Type: Australia: “Littora Novae Hollandiae intra tropicum”; *R. Brown 6101*(BM)

*Oplismenus semialatus* (R. Br.) Desv., Mém. Soc. Agric. Angers 1: 185. 1831.

*Urochloa semialata* (R. Br.) Kunth, Révis. Gramin. 1: 31. 1829

*Axonopus semialatus* (R. Br.) Hook. f., Fl. Brit. India (J.D. Hooker). 7(22): 64. 1896, p.p.

*Arundinella schultzii* Benth., Fl. Austral. 7: 545. 1878. Type: Australia, North Australia, Port Darwin; *Schultz 31*

*Holosetum philippicum* Steud., Syn. Pl. Glumac. 1: 118. 1854. Type: Philippines: Luzon, Cagayan, Cagayan; *H. Cuming 1363* (L). Philippines: Luzon: Nueva Ecija, Nueva Ecija; *H. Cuming 1414* (L).

**Distribution. **Andhra Pradesh, Assam, Bihar, Karnataka, Kerala, Manipur, Meghalaya, Mizoram, Nagaland, Odisha, Tamil Nadu, Uttar Pradesh [Myanmar, Sri Lanka].

**Habit.** Perennial.

**610. **Alloteropsis semialata** (R. Br.) Hitchc. var. **eckloniana** (Nees) C.E. Hubb. ex Bor, Grasses Burma, Ceylon, India & Pakistan 277. 1960 **

*Panicum semialatum* R. Br. var. *ecklonianum* (Nees) Hack. ex T. Durand. & Schinz, Consp. Fl. Afr. [T.A. Durand & H. Schinz] 5: 764. 1894

*Axonopus semialatus* (R. Br.) Hook. f. var. *ecklonii* (Nees) Stapf, Fl. Cap. (Harvey) 7: 418. 1899.

*Bluffia eckloniana* Nees, Del. Sem. Hort. Hamburg. 1834: 8. 1834. Type: “Semina ex America septentrionali sine nomine accepimus.”

*Alloteropsis eckloniana* (Nees) Hitchc., Proc. Biol. Soc. Wash. 29: 128. 1916.

**Distribution.** Kerala, Meghalaya, Manipur, Nagaland, Uttar Pradesh [Sri Lanka].

**Habit.** Perennial.

**Remarks.** All records other than that for Kerala fide Naithani (1990).

**611. **Alloteropsis semialata** (R. Br.) Hitchc. var. **viatica** (Griff.) Ellis & Karthik., Bull. Bot. Surv. India 13(3–4): 175. 1974 (“1971”) **

*Aira viatica* Griff., Not. Pl. Asiat. 3: 54. 1851. Type: India: Churra Punjee; October 18, 1835; *It. ass. 178*

*Axonopus semialatus* (R. Br.) Hook. f., Fl. Brit. India (J.D. Hooker). 7(22): 64. 1896, p.p., excluding type

**Distribution.** Kerala, Manipur, Meghalaya.

**Habit.** Perennial.

**Remarks.** All records for India fide Naithani (1990).

***Cenchrus* L., Sp. Pl. 2: 1049. 1753**

**Lectotype. ***Cenchrus echinatus* L. LT designated by Green in Prop. Brit. Bot. 193. 1929.

**612. **Cenchrus americanus** (L.) Morrone, Ann. Bot. (Oxford), n.s. 106: 127. 2010 **

*Panicum americanum* L., Sp. Pl. 1: 56. 1753. Type: “Habitat in America.” Lectotype = “Panicum Americanum” in Clusius, Rar. Pl. Hist., 2: 215, 215, 1601. LT designated by Clayton & Renvoize in Polhill (ed.), Fl. Trop. E. Africa, Gramineae 3: 672. 1982.

*Pennisetum americanum* (L.) K. Schum., Pflanzenw. Ost-Afrikas B: 51. t. 4. 1895.

*Alopecurus typhoides* Burm. f., Fl. Ind. (N. L. Burman) 27. 1768. Type: “Gramen alopecuroides, spica maxima Indiae orientalis. Plukn. Almag. 174. t. 32. f. 4”

*Pennisetum typhoides* (Burm.f.) Stapf & C.E. Hubb., Bull. Misc. Inform. Kew 1933: 271. 1933.

*Pennisetum typhoideum* L.C. Rich., Syn. Pl. (Persoon) 1: 72. 1805, nom. superfl. & illeg. for *Holcus spicatus*.

*Panicum glaucum* L., Sp. Pl. 1: 56. 1753. Type: “Habitat in Indiis.” Lectotype: *Herb. Hermann 3: 17, No. 44* (BM-000621854). LT designated by Rauschert in Feddes Repert. 83: 662. 1973.

*Pennisetum glaucum* (L.) R. Br., Prodr. Fl. Nov. Holland. 1: 195. 1810

*Setaria glauca* (L.) P. Beauv., Ess. Agrostogr. 169, 178. 1812.

*Penicillaria cylindrica* Roem. & Schult., Syst. Veg., ed. 15 bis [Roemer & Schultes] 2: 498. 1817, p.p. Type: “In India orient., et promont. b. spei.”

*Pennisetum americanum* sensu (L.) Leeke, Z. Naturwiss. 79: 52. 1907, non (L.) K. Schum., 1895

*Pennisetum spicatum* (L.) Körn., Handb. Getreidebaus 1: 284. 1885, non Roem. & Schult., 1817

**Distribution.** Andhra Pradesh, Bihar, Gujarat, Karnataka, Madhya Pradesh, Maharashtra, Meghalaya, Nagaland, Punjab, Rajasthan, Sikkim, Tamil Nadu, Tripura, Uttar Pradesh, Uttarakhand, West Bengal [China]. Widely cultivated.

**Habit.** Annual.

**613. **Cenchrus biflorus** Roxb., Fl. Ind. (Carey & Wallich ed.) 1: 238–239. 1820 **

*Cenchrus annularis* Andersson, Naturw. Reise Mossambique 553. 1864. Type: “Standort auf sandigen trocknen und feuchten Feldern, auf Tertiarkalkformation, auf den Querimbainseln und dem gegenuberliegenden Festlande, 10–13 S.B. Im Mai und Juni Eingesammelt.”

*Cenchrus barbatus* Schumach., Beskr. Guin. Pl. 43. 1827. Type: Ghana: Guinea; *P. Thonning s.n.*

*Cenchrus catharticus* Delile, Index Seminum Hort. Reg. Bot. Monspel. 2. 1844, nom. nud.

**Type.** India: dry elevated parts of the Coromandel coast; *Roxburgh s.n.* (BM).

**Distribution.** Andhra Pradesh, Bihar, Daman and Diu, Delhi, Goa, Gujarat, Haryana, Karnataka, Kerala, Madhya Pradesh, Maharashtra, Punjab, Rajasthan, Tamil Nadu, Uttar Pradesh [Pakistan].

**Habit.** Annual.

**614. **Cenchrus ciliaris** L., Mant. Pl. Altera 302. 1771 **

*Pennisetum ciliare* (L.) Link, Hort. Berol. [Link] 1: 213. 1827

*Cenchrus longifolius* Hochst. ex Steud., Syn. Pl. Glumac. 1: 109. 1854. Type: Nubia: Ad montem Cordofanum Arasch-Cool inter frutices; October 16, 1839; *Kotschy 190*

*Pennisetum cenchroides* Rich., Syn. Pl. (Persoon) 1: 72. 1805.

*Pennisetum incomptum* Nees, Syn. Pl. Glumac. (Steudel) 1: 105. 1854. Type: Nepal; *Royle herb. 55*

*Pennisetum petraeum* Steud., Syn. Pl. Glumac. 1: 106. 1854. Type: “Hochst. hrbr. un. it. arab. nr. 153.” “P. refescens Hochst. Kotschy hrb. nr. 170, Arb. petr. Persia.”

*Setaria vulpina* P. Beauv., Ess. Agrostogr. 171, 178. 1812

*Panicum vulpinum* Willd., Enum. Pl. (Willdenow) 1: 1031. 1809, non L., 1753

*Pennisetum rufescens* Hochst. ex Steud., Syn. Pl. Glumac. 1: 106. in syn., non (Desf.) Spreng., 1825

*Cenchrus glaucus* Mudaliar & Sundararaj, J. Bombay Nat. Hist. Soc. 54: 926. 1958. Type: India: Coimbatore, Agricultural College; *D.D. Sundararaj 93840a* (MH).

**Type.** “Habitat ad. Cap. b. spei margines agrorum et in dunis. Koenig.” Lectotype: *Koenig s.n., Herb. Linn. No. 1217.9* (LINN). LT designated by Clayton & Renvoize in Polhill (ed.), Fl. Trop. E. Africa, Gramineae 3: 691. 1982.

**Distribution.** Andhra Pradesh, Assam, Bihar, Chhattisgarh, Daman and Diu, Delhi, Gujarat, Jammu and Kashmir, Karnataka, Madhya Pradesh, Maharashtra, Punjab, Rajasthan, Tamil Nadu, Uttar Pradesh [China, Nepal].

**Habit.** Perennial.

**Remarks.** See also DeLisle (1962).

**615. **Cenchrus clandestinus** (Hochst. ex Chiov.) Morrone, Ann. Bot. (Oxford) n.s., 106: 127. 2010 **

*Pennisetum clandestinum* Hochst. ex Chiov., Annuario Reale Ist. Bot. Roma 8(1): 41, t. 5, f. 2. 1903. Type: “In Abyssinia legit W. Schimper no. 2084.” Ethiopia; *Schimper 2084 *(FI).

**Distribution.** Andhra Pradesh, Assam, Sikkim, Tamil Nadu, Uttarakhand [also in Ethiopia].

**Habit.** Perennial.

**616. **Cenchrus compressus** (R. Br.) Morrone, Ann. Bot. (Oxford) n.s., 106: 127. 2010 **

*Pennisetum compressum *R. Br., Prodr. Fl. Nov. Holland.: 195. 1810

*Panicum alopecuroides* L., Sp. Pl. 1: 55. 1753. Type: “Habitat in China.” Lectotype: Herb. Linn. No. 80.1 (LINN). LT designated by Veldkamp in Cafferty et al. (ed.), Taxon 49: 253. 2000

*Pennisetum alopecuroides* (L.) Spreng., Syst. Veg. (ed. 16) [Sprengel] 1: 303. 1824 (“1825”)

*Penicillaria alopecuroides* (L.) Sweet, Hort. Brit. [Sweet] 440. 1826

*Alopecurusindicus* L., Syst. Veg. ed. 13: 92. 1774, nom.superfl. & illeg.

*Pennisetum villosum* R. Br. ex Fresen., Mus. Senckenberg. 2: 134. 1837. Type: Ethiopia; *H. Salt s.n.*

*Cenchrus villosus* (R. Br. ex Fresen.) Kuntze, Revis. Gen. Pl. 3(3): 347. 1898, non Spreng., 1824

*Pennisetum longistylum* sensu E. Vilm., Fl. Pleine Terre ed. 1: 599. 1863, non Hochst., 1851

**Distribution. **Karnataka, Tamil Nadu [China, Myanmar]. Naturalized. Native to East Africa.

**Habit. **Perennial.

**Remarks.** Author Kailash notes that “According to the USDA-GRIN, *Cenchrus purpurascens* Thunb. (in Trans. Linn. Soc. London 2:329. 1794) is the correct name for this species.”

**617. **Cenchrus divisus** (Forssk. ex J.F. Gmel.) Verloove, Govaerts & Buttler, Phytotaxa 181(1): 59. 2014 **

*Panicum divisum* Forssk. ex J.F.Gmel., Syst. Nat., ed. 13[bis]. 2: 156. 1791. Type: Arabia; *Forsskal s.n. *(C).

*Pennisetum divisum* (Forssk. ex J.F. Gmel.) Henrard, Blumea 3(1): 162. 1938, in obs.

*Panicum dichotomum* Forssk., Fl. Aegypt.-Arab. 20. 1775, non L., 1753. Type: “Abrab Tummdm. Ubique in campis Arabiae. Fimul cum Panico Bockar.”

*Pennisetum dichotomum* Delile, Descr. Egypte, Hist. Nat. 2: 15, t. 8, f.1. 1813–1814

*Pennisetum elatum* Hochst. ex Steud., Syn. Pl. Glumac. 1: 106. 1854. Type: Arabia; *Herb. un.it. arab. 308*

*Gymnotrix longiglumis* Munro ex Hook. f., Fl. Brit. India (J.D. Hooker). 7(21): 85. 1896, nom. nud.

*Cenchrus ramosissimus* Poiret in Lam., Encycl. 6: 51. 1804. Type. “Cette plante croit in Egypte”, V.S. in herb. Lam.

**Distribution.** Gujarat [also in Saudi Arabia, Africa].

**Habit.** Perennial.

**Remarks.** See also Verloove (2012).

**618. **Cenchrus echinatus** L., Sp. Pl. 2: 1050. 1753 **

*Cenchrus brevisetus* E. Fourn., Mexic. Pl. 2: 50. 1886. Type: Mexico: Veracruz, Valle de Orizaba; no date; *Schafn no. 198 inBourgeau 3140 *(US-978731 (fragm. ex LE)). Nicaragua; *P. Levy 235*

*Cenchrus pungens *Kunth, Nov. Gen. Sp. [H.B.K.] 1: 115 (ed. qto.). 1816. Type: “Crescit locis calidis, planis, subinundatis regni Peruviani prope Guayaquil.” Ecuador: Guayaquil; *Humboldt & Bonpland s.n.*(P).

*Cenchrus viridis* Spreng., Syst. Veg. (ed. 16) [Sprengel] 1: 301. 1824 (“1825”). Type: Guadeloupe; 1817–1819; *Bertero s.n.* (B).

**Type.** “Habitat in Jamaica, Curassao.” Lectotype: *Herb. A. van Royen, sheet no. 912.356-116* (L). LT designated by Veldkamp in Jarvis et al. (ed.), Regnum Veg. 127: 31. 1993.

**Distribution.** Kerala [China]. Naturalized. Native to North and South America.

**Habit.** Annual.

**619. **Cenchrus flaccidus** (Griseb.) Morrone, Ann. Bot. (Oxford) n.s., 106: 128. 2010 **

*Pennisetum flaccidum* Griseb., Nachr. Königl. Ges. Wiss. Georg-Augusts-Univ. 86. 1868. Type: India: Kashmir, Ladak, Nubra, alt. 9000–13000 ft.; *Thomson s.n.*

**Distribution.** Jammu and Kashmir, Uttar Pradesh, Uttarakhand [Afghanisthan, Bhutan, China, Nepal, Pakistan].

**Habit.** Perennial.

**620. **Cenchrus hohenackeri** (Hochst. ex Steud.) Morrone, Ann. Bot. (Oxford) n.s., 106: 128. 2010 **

*Pennisetum hohenackeri* Steud., Syn. Pl. Glumac. 1: 103. 1854. Type: India: Tamil Nadu, Nilgherry Mountains; 1851; *R.F. Hohenacker 930*

*Pennisetum alopecuros* Nees ex Steud., Syn. Pl. Glumac. 1: 102. 1854, non J. Jacq., 1844. Type: India; *Wight s.n.* as *Gymnotrix cenchroides*. *Rottler s.n.* as *Cenchrus hordeiformis.*

*Pennisetum aureum* sensu Dalzell & A. Gibson., Bombay Fl. 294. 1861, non Link, 1827.

**Distribution.** Andhra Pradesh, Bihar, Chhattisgarh, Gujarat, Karnataka, Kerala, Madhya Pradesh, Maharashtra, Odisha, Rajasthan, Tamil Nadu [Pakistan].

**Habit.** Perennial.

**621. **Cenchrus hordeoides** (Lam.) Morrone, Ann. Bot. (Oxford) n.s., 106: 128. 2010 **

*Panicum hordeoides* Lam., Tabl. Encycl. 1(1): 170. 1791. Type: “E. Siera Leona, Smeathm.”

*Pennisetum hordeoides* (Lam.) Steud., Syn. Pl. Glumac. 1: 103. 1854

*Gymnotrix hordeoides* (Lam.) Kunth, Révis. Gramin. 1: 48. 1829 (as “Gumnothrix”)

*Cenchrus parviflorus* Poir., Encycl. (Lamarck) 6: 52. 1804. Type: “Cette plante croit a Porto-Ricco. Elle a été communiquée a M. Lamarck par M. Ventenat.” Puerto Rico; *M. Ventenat s.n. *(P).

*Pennisetum parviflorum* (Poir.) Trin., Gram. Panic. [Trinius] 65. 1826

*Pennisetum antillarum* (Poir.) Desv., Opusc. Sci. Phys. Nat. 76. 1831

*Saccharum antillarum* (Poir.) Roem. & Schult., Syst. Veg., ed. 15 bis [Roemer & Schultes] 2: 877. 1817.

*Setaria antillarum* (Poir.) Kunth, Révis. Gramin. 1: 46. 1829

*Pennisetum imberbe* Edgew., J. Asiat. Soc. Bengal 21: 181. 1852, isonym. Type: [India]: “Found at Gurhrampur, November.”

**Distribution.** Bihar, Madhya Pradesh, Rajasthan. Naturalized.

**Habit.** Annual.

**622. **Cenchrus lanatus** (Klotzsch) Morrone, Ann. Bot. (Oxford) n.s., 106: 128. 2010 **

*Pennisetum lanatum* Klotzsch, Bot. Ergebn. Reise Waldemar 65. t. 99. 1862. Type: “Beide Formen werden im Suden von Europa haufig angebaut.” “Prinzen Waldemar auf Ceylon, dem Himalaya und an den Grenzen von Tibet gesammelte Pflanzen.”

*Pennisetum nepalense* sensu Griseb., Nachr. Königl. Ges. Wiss. Georg-Augusts-Univ. 86. 1868, non Spreng., 1825

**Distribution.** Jammu and Kashmir [Afghanistan, China, Nepal, Pakistan].

**Habit. **Perennial.

**623. **Cenchrus mezianus** (Leeke) Morrone, Ann. Bot. (Oxford) n.s., 106: 128. 2010 **

*Pennisetum mezianum* Leeke, Z. Naturwiss. 79: 39. 1907. Type: “Ostafrika, Somaliland, Brit-Ostafrika, Deutsch-Ostafrika.”

*Pennisetum brachystachyum* Hack., Vierteljahrsschr. Naturf. Ges. Zürich 55: 233. 1910. Type: [Africa] “Britisch-Ostafricka”: Makindu River; April 14, 1902; *Kassner 584* (US).

*Pennisetum massaicum* Stapf, Bull. Misc. Inform. Kew 1906: 82. 1906, p.p. Type: British East Africa: Kikumbuliu; *Scott Elliot 6292*. Makindu; *Kassner 584, Linton 72*. Baringo; 3400 ft.; *Johnston s.n.*

**Distribution.** India [also in Africa].

**Habit.** Perennial.

**Remarks.** Distribution in India not recorded.

**624. **Cenchrus orientalis** (Rich.) Morrone, Ann. Bot. (Oxford) n.s., 106: 128. 2010 **

*Pennisetum orientale* Rich., Syn. Pl. (Persoon) 1: 72. 1805. Type: “Hab. in Oriente.”

*Panicum orientale* (Rich.) Willd., Enum. Pl. (Willdenow) 2: 1031. 1809. Type: “Habitat in Galatia.”

*Pennisetum fasciculatum* Trin., Mém. Acad. Imp. Sci. Saint-Pétersbourg, Sér. 6, Sci. Math., Seconde Pt. Sci. Nat. 3, 1(1–2): 181. 1834. Type: “V. ssp. Pers. et e monte Sinai.”

*Pennisetum orientale* Rich. var. *triflorum* (Nees ex Steud.) Stapf ex Hook. f., Fl. Brit. India (J.D. Hooker). 7(21): 86. 1896

*Pennisetum triflorum* Nees ex Steud., Syn. Pl. Glumac. 1: 107. 1854. Type: Nepal; *Royle 59*

**Distribution.** Bihar, Chhattisgarh, Himachal Pradesh, Jammu and Kashmir, Karnataka, Kerala, Madhya Pradesh, Maharashtra, Punjab, Rajasthan, Sikkim, Tamil Nadu, Uttar Pradesh, West Bengal [Afghanistan, Nepal, Pakistan].

**Habit.** Not recorded.

**625. **Cenchrus pedicellatus** (Trin.) Morrone, Ann. Bot. (Oxford) n.s., 106: 128. 2010 **

*Pennisetum pedicellatum* Trin., Mém. Acad. Imp. Sci. Saint-Pétersbourg, Sér. 6, Sci. Math., Seconde Pt. Sci. Nat. 3, 1(2–3): 184. 1834. Type: Cape Verde Islands, St. Iago; *D. Peters s.n.* (LE-TRIN-1101.01).

*Eriochaeta densiflora* Fig. & de Not., Mem. Reale Accad. Sci. Torino ser. 2, 14: 376. t. 31. 1854. Type: Africa: “In Nubia superiore iisdem locis ac praecedens.”

*Pennisetum densiflorum* (Fig. & De Not.) T. Durand & Schinz, Consp. Fl. Afr. [T.A. Durand & H. Schinz] 5: 778. 1894.

*Eriochaeta reversa* Fig. & De Not., Mem. Reale Accad. Sci. Torino ser. 2, 14: 378. t. 32. 1854. Type: Africa: “In nubia superiore cum praecedentibus.”

*Eriochaeta secundiflora* Fig. & De Not., Mem. Reale Accad. Sci. Torino ser. 2, 14: 375. t. 30. 1854. Type: Africa: “In Nubiae superiois regione Kordofan.”

*Pennisetum secundiflorum* (Fig. & De Not.) T. Durand & Schinz, Consp. Fl. Afr. [T.A. Durand & H. Schinz] 5: 784. 1894

*Pennisetum amoenum* Hochst. ex A. Rich., Tent. Fl. Abyss. 2: 386. 1850–1851. Type: “Crescti circa Aderbati, mense Septembre (Quartin Dillon, Schimper).” Ethiopia: crescit circa Aderbati; *Schimper 641 *(P). Ethiopia: crescit circa Aderbati; *Quartin Dillon s.n. *(P).

*Pennisetum araneosum* Edgew., J. Asiat. Soc. Bengal 21: 180. 1852.

*Pennisetum implicatum* Steud., Syn. Pl. Glumac. 1: 107. 1854. Type: “Leprieur legit in Senegambria”; *Leprieur s.n.*

*Pennisetum intertextum* Schlecht., Bot. Zeitung (Berlin) 9: 878. 1851. Type: [Africa: Cape Verde Is.]: “An den Seiten der Thaler zwischen Steinen auf der Insel Mayo”; *C. Pabst s.n.*

*Pennisetum notarisii* T. Durand & Schinz, Consp. Fl. Afr. [T.A. Durand & H. Schinz] 5: 781. 1894

*Pennisetum dillonii* Steud., Syn. Pl. Glumac. 1: 107. 1854, in obs., nom. nud. (as “dilloni”). Type: “Prov. Chire. Abyssin.”

**Distribution.** Andhra Pradesh, Assam, Bihar, Chhattisgarh, Gujarat, Jharkhand, Karnataka, Kerala, Madhya Pradesh, Maharashtra, Odisha, Punjab, Rajasthan, Tamil Nadu, Uttar Pradesh, West Bengal [China].

**Habit.** Annual.

**626. **Cenchrus pennisetiformis** Hochst. & Steud., Syn. Pl. Glumac. 1: 109. 1854 **

*Pennisetum pennisetiforme* (Hochst. & Steud.) Wipff, Sida 19(3): 527. 2001.

*Cenchrus aequiglumis* Chiov., Agron. Colon. 20: 108. 1926. Type: Africa “Flora Somala Transjuliense. racoolte dal Dr. Pompeo Gorini nel 1925.”

*Pennisetum cenchroides* Rich. var. *echinoides* Hook. f., Fl. Brit. India (J.D. Hooker). 7(22): 88–89. 1896

*Cenchrus echinoides* Wight ex Steud., Nomencl. Bot., ed. 2 (Steudel) 1: 317. 1840, nom. nud.

*Cenchrus lappaceus* Tausch, Flora 20: 97. 1837, nom. Later hom., non L., 1763

**Type.** Saudi Arabia: Jedda, in deserto prope oppid. Deschedda; January 28, 1836; *G.H.W. Schimper 973* (P). LT designated by Wipff in Sida 19: 527. 2001.

**Distribution.** Bihar, Daman and Diu, Goa, Gujarat, Jammu and Kashmir, Maharashtra, Rajasthan, Tamil Nadu, Uttar Pradesh [Myanmar, Sri Lanka].

**Habit.** Annual or perennial.

**627. **Cenchrus polystachios** (L.) Morrone, Ann. Bot. (Oxford) n.s., 106: 129. 2010 **

*Panicum polystachion* L., Syst. Nat., ed. 10. 2: 870. 1759. Type: “Habitat [in India.] Sp. Pl., ed. 2. 1: 82. 1762.” Lectotype: Herb. Linn. No. 80.4 (LINN). LT designated by van der Zon in Wageningen Agric. Univ. Pap. 92-1: 335. 1992.

*Pennisetum polystachion* (L.) Schult., Mant. 2 (Schultes) 146. 1824 (as “polystachyum”).

*Panicum barbatum* Roxb., Fl. Ind. (Carey & Wallich ed.) 1: 285. 1820, non Lam. 1791. Type: India: Collected from Sumatra and seed grown in Royal Botanical Garden, Calcutta.

*Panicum triticoides* Poir., Encycl. (Lamarck) Suppl. 4: 274. 1816. Type: “J’ignore le lieu natal de cette plante. (Herb. Desfontaine).”

*Pennisetum triticoides* (Poir.) Roem. & Schult., Syst. Veg., ed. 15 bis [Roemer & Schultes] 2: 877. 1817. Type: Herb. Desfontaine.

*Panicum densispica* Poir., Encycl. (Lamarck) Suppl. 4: 273. 1816, nom. superfl & illeg. for *P. cenchroides* A. Rich.

*Pennisetum pedicellatum* sensu Senaratna, Grasses of Ceylon (Perad. Man. No. 8) 155. 1956, non Trin., 1834

*Panicum cauda-ratti* Schumach., Kongel. Danske Vidensk. Selsk. Naturvidensk. Math. Afh. 3: 79. 1827. Type: Ghana; *Thonning s.n.*

*Pennisetum cauda-ratti* (Schumach.) Franch., Bull. Soc. Hist. Nat. Autun 8: 365. 1895

*Pennisetum amethystinum* P. Beauv., Ess. Agrostogr. 172 1812, nom. nud.

*Pennisetum setosum* (Sw.) Rich., Syn. Pl. (Persoon) 1: 72. 1805.

*Cenchrus setosus* Sw., Prodr. (Swartz) 26. 1788. Type: India orientalis; *Swartz s.n.* (S).

*Gymnotrix geniculata* Schult., Mant. 2 (Schultes) 284. 1824 (as “Gymnothrix”). Type: “In martinica, Hortul”; *D. Sieber s.n.*

*Panicum cenchroides* Rich., Actes Soc. Hist. Nat. Paris 1: 106. 1792. Type: French Guiana: “E Cayenna missarum a Domino Le Blond”; *LeBlond s.n.* (P).

*Setaria cenchroides* (Rich.) Roem. & Schult., Syst. Veg., ed. 15 bis [Roemer & Schultes] 2: 495. 1817.

*Panicum erubescens* Willd., Enum. Pl. (Willdenow) 2: 1031. 1809. Type: “Habitat in insula St. Thomae Americes.”

*Setaria erubescens* (Willd.) P. Beauv., Ess. Agrostogr. 169, 178. 1812

*Pennisetum flavescens* J. Presl in C. Presl, Reliq. Haenk. 1(4–5): 316. 1830. Type: “Habitat in Mexico.” Mexico; *Haenke s.n.* (PR)

*Pennisetum hamiltonii* Steud., Nomencl. Bot., ed. 2 (Steudel) 2: 297. 1841

*Pennisetum hirsutum* Nees, Fl. Bras. Enum. Pl. 2(1): 284. 1829

*Pennisetum nicaraguense* E. Fourn., Bull. Soc. Bot. France 27: 293. 1880. Type: Nicaragua: circa Granada; *P. Levy 1304* (P).

*Pennisetum pallidum* Nees, Fl. Bras. Enum. Pl. 2(1): 285. 1829. Type: Brasilia: “Habitat in apricis praeruptis graminibus parcis obsitis ad latera montium de Mentanha et Itambé, districtus adamantini, provincae Minarum generalium”; *Martius s.n.* (M).

*Pennisetum purpurascens *Kunth, Nov. Gen. Sp. [H.B.K.] 1: 113 (ed. qto.). 1816. Type: “Crescit in aridis, devexis montis ignivomi Mexicani, Volcan de Jorullo, alt 420 hexapodarum.” Mexico: Volcán de Jorullo, alt. 420 ft.;* Humboldt & Bonpland s.n.* (P).

*Pennisetum richardii* Kunth, Révis. Gramin. 1: 49. 1829 (as “richardi”)

*Pennisetum uniflorum *Kunth, Nov. Gen. Sp. [H.B.K.] 1: 114 (ed. qto.). t. 34. 1816. Type: “Crescit locis subtemperatis, planis Prov. Novae Andalusiae, juxta Cumanacoa”; *Humboldt & Humboldt s.n.*

*Pennisetum alopecuroides* Desv. ex Ham., Prodr. Pl. Ind. Occid. (Hamilton) 11. 1825, nom. later hom. & illeg., non (L.) Spreng., 1824.

*Pennisetum erubescens* (Willd.) Link, Hort. Berol. [Link] 1: 215. 1827, non Desv. ex Ham., 1825

**Distribution.** Andhra Pradesh, Assam, Bihar, Chhattisgarh, Karnataka, Kerala, Madhya Pradesh, Maharashtra, Odisha, Tamil Nadu, Uttar Pradesh, West Bengal [China].

**Habit.** Annual.

**628. **Cenchrus prieurii** (Kunth) Maire, Bull. Mus. Natl. Hist. Nat, ser. 2, 3: 523. 1931 var. **prieurii** (as “prieuri”) **

*Cenchrus macrostachyus* Hochst. ex Steud., Syn. Pl. Glumac. 1: 109. 1854. Type: Sudan: Kurdofan, Ad Pagum Cordofanum Abu-Gerad in Collobus Arenosis; September 18, 1839; *K.G.T. Kotschy 4*

*Pennisetum breviflorum* Steud., Syn. Pl. Glumac. 1: 107. 1854. Type: “Leprieur legit in Senegambia”; *Leprieur s.n.*

*Pennisetum prieurii* Kunth, Révis. Gramin. 2: 411. t. 119. 1831. Type: “Crescit in senegambia, collibus arenosis”; *Leprieur s.n.*

**Distribution.** Punjab, Rajasthan, Uttar Pradesh [Pakistan].

**Habit.** Annual.

**629. **Cenchrus prieurii** (Kunth) Maire var. **scabra** Bhandari, Fl. Ind. Desert 395. 1978 **

**Type.** India: Rajasthan, Jodhpur, Massuria; December 6, 1960; *M.M. Bhandari 793* (JAC).

**Distribution.** Rajasthan, Uttar Pradesh.

**Habit.** Annual.

**Remarks.** Record for Rajasthan fide Naithani (1990).

**630. **Cenchrus purpureus** (Schumach.) Morrone, Ann. Bot. (Oxford) n.s., 196: 129. 2010 **

*Pennisetum purpureum* Schumach., Beskr. Guin. Pl. 44. 1827. Type: Ghana: Guinea; *Thonning 355* (C).

*Gymnotrix nitens* Andersson, Naturw. Reise Mossambique 552. 1864 (as “Gymnothrix”). Type: “Standort, Auf trocknen sandigen oder in der Nahe sumpfiger Wiesen der Halbinsel Cabaceira 15 S.B. Eingesammelt in Mai, auch an anderen orten ahnlichen Bodens vokommend.”

*Pennisetum nitens* (Andersson) Hack., Bol. Soc. Brot. 6: 142. 1888

*Pennisetum benthamii* Steud., Syn. Pl. Glumac. 1: 105. 1854 (as “benthami”). Type: “P. Macrostachyum Benth. in Hook. Nig. Fl. 563. (non Trin. 1834) Fernando Po, Guinea.”

*Pennisetum flavicomum* Leeke, Z. Naturwiss. 79: 45. 1907. Type: “Agyprischer Sudan: Kordofan.”

*Pennisetum flexispica* K. Schum., Pflanzenw. Ost-Afrikas C: 105. 1895. Type: “Si-Stuhlm. Coll. I, n. 1099”.

*Pennisetum pallescens* Leeke, Z. Naturwiss. 79: 47. 1907. Type: “Westafrika: Togo”.

*Pennisetum pruinosum* Leeke, Z. Naturwiss. 79: 46. 1907. Type: Ostafrika, Einheimischer Name, “Magugu”

*Pennisetum macrostachyum* Benth., Niger Fl. 563. 1849, nom. Later hom. & illeg., non *Pennisetum macrostachys* (Brongn.) Trin., 1834

**Distribution.** Andhra Pradesh, Assam, Bihar, Delhi, Gujarat, Himachal Pradesh, Karnataka, Kerala, Madhya Pradesh, Maharashtra, Odisha, Rajasthan, Tamil Nadu, West Bengal [China].

**Habit.** Perennial.

**631. **Cenchrus rajasthanensis** Kanodia & P.C. Nanda, Geobios (Jodhpur) 5(4): 157. f. 1. 1978 **

**Distribution.** Rajasthan.

**Habit.** Not recorded.

**Remarks.** Record for India fide Naithani (1990).

**632. **Cenchrus ramosus** (Hochst.) Morrone, Ann. Bot. (Oxford) n.s., 106: 129. 2010 **

*Gymnotrix ramosa* Hochst., Flora 27: 252. 1844 (as “Gymnothrix”). Type: Aethiopica: Sennar Province; *Kotschyi 199*.

*Pennisetum ramosum* (Hochst.) Schweinf., Beitr. Fl. Aethiop. 301. 1867.

*Pennisetum arvense* Pilg., Bot. Jahrb. Syst. 30: 119. 1901. Type: Ethiopia: “auf trockenen Bergrucken in der sonst sumpfigen Ebene, Dembea, 5800 ft.”; October 1863; *Schimper 1399. *Ethiopia: “in Sumpfen bei Hamedo” 4500 ft.; September 1862; *Schimper 1058*.

*Pennisetum ovale* Rupr. ex Steud., Syn. Pl. Glumac. 1: 104. 1854. Type: Ethiopia: Nubia; *Ruprecht. (In Kotschy fl. ath. 199)*

**Distribution.** India [also in Africa].

**Habit.** Annual.

**Remarks.** Distribution in India not recorded.

**633. **Cenchrus setigerus** Vahl, Enum. Pl. (Vahl) 2: 395. 1805 **

*Cenchrus biflorus* sensu Hook. f., Fl. Brit. India (J.D. Hooker). 7(21): 89. 1896, non Roxb., 1820

*Cenchrus bulbifer* Hochst. ex Boiss., Fl. Orient. (Boissier) 5: 448. 1884, nom. nud. pro. syn.

*Cenchrus uniflorus* Ehrenb. ex Boiss., Fl. Orient. (Boissier) 5: 448. 1884, nom. nud. pro. syn.

*Pennisetum vahlii* Kunth, Révis. Gramin. 1: 49. 1829, nom. superfl. & illeg. for Cenchrus setigerus Vahl

**Type.** “Habitat in Arabia. Forskal.” Saudi Arabia; *Forsskal s.n.* (C).

**Distribution.** Andhra Pradesh, Bihar, Chhattisgarh, Daman and Diu, Goa, Gujarat, Haryana, Jammu and Kashmir, Karnataka, Kerala, Madhya Pradesh, Maharashtra, Punjab, Rajasthan, Tamil Nadu, Uttar Pradesh [China, Pakistan].

**Habit.** Perennial.

***Cyrtococcum* Stapf, Fl. Trop. Afr. [Oliver et al.] 9: 15. 1917, 9: 745. 1920**

**Lectotype. ***Cyrtococcum setigerum* (P. Beauv.) Stapf (*Panicum setigerum* P. Beauv.) LT designated by F. W. Gould in R. A. Howard, Fl. Lesser Antilles 3: 121. 1979.

**634. **Cyrtococcum accrescens** (Trin.) Stapf, Hooker’s Icon. Pl. 31: sub t. 3096. 1922 **

*Panicum accrescens* Trin., Sp. Gram. [Trinius] 1(8): t. 88. 1827. Type: “Figura ad specimen nepalense.”

*Cyrtococcum patens* (L.) A. Camus var. *latifolium* (Honda) Ohwi, Acta Phytotax. Geobot. 11(1): 47–48. 1942.

*Panicum patens* L. f. *latifolium* Honda, Bot. Mag. (Tokyo) 37: 25. 1923. Type: China: Taiwan, Liukiu, Onnah; 1899; *K. Miyake s.n.*

**Distribution.** Andaman and Nicobar Islands, Assam, Karnataka, Maharashtra, Nagaland, Odish, Uttar Pradesh, Uttarakhand [China, Nepal].

**Habit. **Not recorded.

**Remarks.** Distribution in Karnataka, Nagaland, and Uttar Pradesh fide Naithani (1990).

**635. **Cyrtococcum deccanense** Bor, Kew Bull. 1956: 255–256. 1956 **

*Panicum patens* sensu Hook. f., Fl. Brit. India (J.D. Hooker). 7(21): 57. 1896, non L., 1753

**Type. **Sri Lanka: Hakgala, gully near Botanical Garden; 6000 ft.; December 30, 1950; *F. Ballard 1341* (K).

**Distribution.** Karnataka, Kerala, Maharashtra, Tamil Nadu [Sri Lanka].

**Habit.** Annual.

**Remarks.** Records from Karnataka and Tamil Nadu fide Naithani (1990).

**636. **Cyrtococcum longipes** (Wight & Arn. ex Hook. f.) A. Camus, Bull. Mus. Natl. Hist. Nat. 27: 118. 1921. **

*Panicum longipes* Wight & Arn. ex Hook. f., Fl. Brit. India (J.D. Hooker). 7(21): 58. 1896. Syntypes: India: Nilghiri and Pulney Hills; *Heyne* (Numer. List [Wallich] no. 8741). *Wight & Arn* (*Wight Cat. no. 1638*). Karkun Ghar, alt. 2000 ft.; *Lawson s.n.*

*Cyrtococcum patens* sensu Senaratna, Grasses of Ceylon (Perad. Man. No. 8) 120. 1956, non (L.) A. Camus, 1921

**Distribution.** Andhra Pradesh, Assam, Karnataka, Kerala, Sikkim, Tamil Nadu [Myanmar].

**Habit.** Perennial.

**637. **Cyrtococcum oxyphyllum** (Hochst. ex Steud.) Stapf, Hooker’s Icon. Pl. 31: sub t. 3096. 1922. **

*Panicum oxyphyllum* Hochst. ex Steud., Syn. Pl. Glumac. 1: 65. 1853. Type: India: Canara; *R.F. Hohenacker 627*

*Panicum hermaphroditum* Steud., Syn. Pl. Glumac. 1: 67. 1853. Type: Philippines; *Cuming no. 554*

*Panicum pilipes* Nees & Arn. ex Buse, Pl. Jungh. 376. 1854. Type: “Habitat insulae Javae sylvas in sinu Wijnkoopsbaai, Junghuhn.” Indonesia: Java; *Cuming 554*

**Distribution.** Andhra Pradesh, Arunachal Pradesh, Assam, Bihar, Chhattisgarh, Goa, Karnataka, Kerala, Madhya Pradesh, Maharashtra, Meghalaya, Mizoram, Nagaland, Odisha, Sikkim, Tamil Nadu, Tripura, West Bengal [Bhutan, China, Sri Lanka].

**Habit.** Annual.

**638. **Cyrtococcum patens** (L.) A. Camus, Bull. Mus. Natl. Hist. Nat. 27: 118. 1921 **

*Panicum patens* L., Sp. Pl. 1: 58. 1753. Type: “Habitat in India; similis e Lusitania.” Lectotype: *Herb. Linn. No. 80.63 *(LINN). LT designated by Mitra & Jain in Manilal (ed.), Bot. Hist. Hort. Malabaricus 151. 1980.

*Panicum muricatum* Retz., Observ. Bot. (Retzius) 4: 18. 1786–1787. Type: “Misit Cel. Koenig.”

*Cyrtococcum muricatum* (Retz.) Bor, Grasses Burma, Ceylon, India and Pakistan. 291. 1960.

*Panicum radicans* Retz., Observ. Bot. (Retzius) 4: 18. 1786–1787. Type: “Ad Canton Lectum dadit Honor. Wennerberg.” Neotype: Philippines: Luzon, Rizal, Manila; December, 1914; *F. Blanco 711*(L).

*Cyrtococcum radicans* (Retz.) Stapf, Hooker’s Icon. Pl. 31: sub t. 3096. 1922

*Panicum schmidtii* Hack., Bot. Tidsskr. 24: 99. 1901. Type: Thailand: Tratpron, Kohchang Island, plains near Klong Munsé; 1899; *c.i. s.n.*

*Cyrtococcum schmidtii* (Hack.) Henrard, Blumea 3(3): 436. 1940.

*Panicum carinatum* J. Presl in C. Presl, Reliq. Haenk. 1(4–5): 309. 1830. Type: “Habitat in insula Luzonia ad Sorzogon.” Philippine Islands: Luzon, Sorzogon; *Haenke s.n.* (PR)

*Panicum obliquum* Roth ex Roem. & Schult. Syst. Veg., ed. 15 bis [Roemer & Schultes] 2: 433. 1817

**Distribution.** Andaman and Nicobar Islands, Assam, Bihar, Karnataka, Kerala, Maharashtra, Manipur, Meghalaya, Odisha, Sikkim, Tamil Nadu, Tripura, Uttar Pradesh, Uttarakhand, West Bengal [Bangladesh, Bhutan, Myanmar, Nepal, Sri Lanka].

**Habit. **Perennial.

**639. **Cyrtococcum trigonum** (Retz.) A. Camus, Bull. Mus. Natl. Hist. Nat. 27: 118. 1921 **

*Panicum trigonum* Retz., Observ. Bot. (Retzius) 3: 9. 1783. Type: India; *Koenig s.n.* (LD). “Specimina javanica melius conceniunt cum Rumph. quam cum Rheed. figura cujus folia longiora.”

*Panicum difforme* Roth, Syst. Veg., ed. 15 bis [Roemer & Schultes] 2: 433. 1817. Type: India orientalis; “Roth, nov. plant. spec. mss” cited

*Panicum patens* sensu Burm. f., Fl. Ind. (N. L. Burman) 26. t. 10. f. 3. 1768, non L., 1753

**Distribution.** Andhra Pradesh, Bihar, Goa, Karnataka, Kerala, Maharashtra, Odisha, Tamil Nadu, West Bengal [Sri Lanka].

**Habit.** Perennial.

***Digitaria* Haller f., Hist. Stirp. Helv. 2: 244. 1768, nom. cons**.

**Type. ***Digitaria sanguinalis* (L.) Scopoli (*Panicum sanguinale* L.) (type cons.).

**640. **Digitaria abludens** (Roem. & Schult.) Veldkamp, Blumea 21(1): 53–55 f. 11d. 12. 1973 **

*Panicum abludens* Roem. & Schult., Syst. Veg., ed. 15 bis [Roemer & Schultes] 2: 457. 1817. Type: India; *Heyne s.n.* (B). “Pancium paradoxum Roth, nov. plant. Spec. Ms. ..”

*Digitaria granularis* (Trin.) Henrard, Monogr. Digitaria 302. 1950

*Paspalum granulare* Trin. in Spreng., Neue Entdeck. Pflanzenk. 2: 47. 1821. Type: “Hab. In India Orientali.” Koenig sub Milium setaceum in Herb. Trin ex Herb. Banks ex Herb. Jacquin (LE).

*Digitaria pedicellaris* Prain, Bengal Pl. 2: 1182. 1903, nom. superfl.

*Paspalum pedicellare* Trin. ex Hook. f., Fl. Brit. India (J.D. Hooker). 7(21): 19. 1896, nom. superfl. for *Paspalum granulare* Trin.

**Distribution.** Andhra Pradesh, Bihar, Chhattisgarh, Gujarat, Karnataka, Kerala, Madhya Pradesh, Maharashtra, Odisha, Rajasthan, Tamil Nadu, Uttar Pradesh, Uttarakhand [Bhutan, China, Myanmar, Nepal, Pakistan].

**Habit.** Annual.

**641. **Digitaria abyssinica** (Hochst. ex A. Rich.) Stapf, Bull. Misc. Inform. Kew 1907: 213. 1907 **

*Panicum abyssinicum* Hochst. ex A. Rich., Tent. Fl. Abyss. 2: 360. 1850–1851. Type: Ethiopia: “in fruticetis opacis ad radices septrionales montis scholoda”; September 22, 1837; *W. Schimper U. I. 1840 Sect. primo 82* (P).

**Distribution.** Tamil Nadu [Sri Lanka].

**Habit.** Perennial.

**642. **Digitaria bicornis** (Lam.) Roem. & Schult., Syst. Veg., ed. 15 bis [Roemer & Schultes] 2: 470. 1817 **

*Paspalum bicorne* Lam., Tabl. Encycl. 1: 176. 1791. Type: “Lieu nat. I’Inde.” India; *Sonnerat in Herb. Lamarck* (P-LAM).

*Panicum bicorne* (Lam.) Kunth, Enum. Pl. (Kunth) 1: 83. 1833.

*Digitaria barbata* Willd., Enum. Pl. (Willdenow) 1: 91. 1809. Type: “Habitat in India Orientali”; *Herb. Desvaux s.n.* (P).

*Digitaria rottleri* Roem. & Schult., Syst. Veg., ed. 15 bis [Roemer & Schultes] 2: 471. 1817. Type: India; *Rottler s.n.*

*Panicum heteranthum* Hook. f., Fl. Brit. India (J.D. Hooker). 7(21): 16. 1896, non Link, 1827, nom illeg., later hom.

*Panicum barbatum* (Willd.) Kunth, Révis. Gramin. 1: 33. 1829, non Lam., 1791

**Distribution.** Andhra Pradesh, Bihar, Chhattisgarh, Himachal Pradesh, Karnataka, Kerala, Maharashtra, Manipur, Rajasthan, Tamil Nadu, Uttar Pradesh, Uttarakhand, West Bengal [China, Myanmar, Nepal, Pakistan, Sri Lanka].

**Habit.** Annual.

**643. **Digitaria biformis** Willd., Enum. Pl. (Willdenow) 1: 92. 1809 **

*Panicum biforme* (Willd.) Kunth, Révis. Gramin. 1: 33. 1829.

*Panicum sanguinale* L. var. *biforme* (Willd.) Hack. ex T. Durand & Schinz, Consp. Fl. Afr. [T.A. Durand & H. Schinz] 5: 762. 1894

**Type.** “Habitat in insula Mauritii et Borboniae”; *Bory de St. Vincent 47* (B-W-1652).

**Distribution.** Karnataka, Tamil Nadu, Uttar Pradesh, Uttarakhand [Pakistan].

**Habit.** Not recorded.

**Remarks.** The taxonomic editors note that this species could be synonymous with *D. bicornis* or *D. ciliaris*.

**644. **Digitaria brownii** (Roem. & Schult.) Hughes, Bull. Misc. Inform. Kew 1923: 313. 1923 **

*Panicum brownii* Roem. & Schult., Syst. Veg., ed. 15 bis [Roemer & Schultes]. 2: 462. 1817, nom. nov. for *Panicum villosum* R. Br.

*Panicum villosum* R. Br., Prodr. Fl. Nov. Holland. 1: 192. 1810, nom. illeg., non Lam., 1791. Type: Australia: Keppel Bay; *R. Brown 6119*, p.p. (BM).

**Distribution.** Tamil Nadu [also in Australia, New Zealand].

**Habit.** Perennial.

**Remarks. **It is unclear if this species is naturalized in Tamil Nadu. Its native range is not recorded.

**645. **Digitaria ciliaris** (Retz.) Koeler, Descr. Gram. (Koeler) 27. 1802 **

*Panicum ciliare* Retz., Observ. Bot. (Retzius) 4: 16. 1786–1787. Type: “E Java et China adtulit honor. Wennerberg.” China: Guangdong, Guangzhou; *Wennerberg s.n.* (LD).

*Paspalum sanguinale* (L.) Lam. var. *ciliare* (Retz.) Hook. f., Fl. Brit. India (J.D. Hooker). 7(21): 15. 1896

*Paspalum sanguinale* (L.) Lam. var. *rottleri* Hook. f., Fl. Brit. India (J.D. Hooker). 7(21): 16. 1896. Type: India; *Wight Herbarium no. 3032 and 3033*

*Panicum adscendens* Kunth, Nov. Gen. Sp. [H.B.K] 1: 97 (ed. qto.). 1816. Type: “Crescit in Nova Andalusia prope Cumanacoa (100 hexap.), in Peruvia ad maris littora prope Guayaquil et Santa; in Nova Hispania juxta Zelaya et Queretaro, alt. 950 hexap.” Lectotype: Peru [Ecuador]: Guyaquil; *Humboldt & Bonpland s.n.* (P). LT designated by Veldkamp in Blumea 21(1): 32. 1973.

*Digitaria adscendens* (Kunth) Henrard, Blumea 1(1): 92. 1934.

*Digitaria adscendens* (Kunth) Henrard var. *criniformis* Henrard, Monogr. Digitaria 999. 1950

*Digitaria adscendens* (Kunth) Henrard ssp. *chrysoblephara* (Fig. & De Not.) Henrard, Monogr. Digitaria 998. 1950.

*Digitaria chrysoblephara* Fig. & De Not., Mem. Reale Accad. Sci. Torino ser. 2, 14: 364. t. 27. 1854. Type: Africa: “In Nubia superiore ad Fazoge”; *Figari s.n.*(FI).

*Digitaria marginata* Link, Enum. Hort. Berol. Alt. 1: 102. 1821. Type: “Hab. in Brasilia.” Brazil; *Herb. Link 67* (B (left side of sheet)).

*Digitaria fimbriata* Link, Hort. Berol. [Link] 1: 226. 1827. Type: “Hab. in Brasilia.” Brazil; *Herb. Link 97* (B [right side of sheet]).

*Digitaria marginata* Link var. *fimbriata* (Link) Stapf, Fl. Trop. Afr. [Oliver et al.] 9: 440. 1919.

**Distribution.** Andhra Pradesh, Arunachal Pradesh, Bihar, Chhattisgarh, Gujarat, Jammu and Kashmir, Karnataka, Kerala, Madhya Pradesh, Maharashtra, Manipur, Meghalaya, Nagaland, Odisha, Rajasthan, Tamil Nadu, Uttar Pradesh, Uttarakhand, West Bengal [China, Myanmar, Nepal, Pakistan].

**Habit.** Not recorded.

**646. **Digitaria compacta** (Roth) Veldkamp, Blumea 21(1): 71. 1973 **

*Paspalum compactum* Roth, Syst. Veg., ed. 15 bis [Roemer & Schultes] 2: 316. 1817. Type: India orientalis; *Heyne s.n.* (B).

*Digitaria cruciata* (Nees) A. Camus var. *esculenta* Bor, Webbia 11: 353. 1955. Type: India: Khasia, Pomrang; September 15, 1850; *J.D. Hooker & T. Thomson s.n.* (K).

**Distribution.** Meghalaya, Uttar Pradesh.

**Habit.** Annual.

**Remarks.** Distribution in India fide Naithani (1990).

**647. **Digitaria cruciata** (Nees) E.G. Camus & A. Camus, Fl. Indo-Chine [P.H. Lecomte et al.] 7: 399. 1922 var. **cruciata

*Panicum cruciatum* Nees, Syn. Pl. Glumac. (Steudel) 1: 39. 1854. Type: Nepal; *Royle Herb. no. 28, 29*.

*Paspalum sanguinale* (L.) Lam. var. *cruciatum* (Nees) Hook. f., Fl. Brit. India (J.D. Hooker). 7(21): 14. 1896

**Distribution.** Meghalaya, Rajasthan, Uttar Pradesh, Uttarakhand [Bhutan, China, Mynamar, Nepal, Pakistan].

**Habit.** Annual.

**648. **Digitaria cruciata** (Nees) A. Camus var. **pectinata** Veldkamp, Blumea 21(1): 72. 1973 **

**Type. **India; *Griffith KD 6574* (W).

**Distribution.** Meghalaya, Rajasthan, Uttar Pradesh [Nepal].

**Habit.** Not recorded.

**Remarks.** Record for Meghalaya fide Naithani (1990).

**649. **Digitaria duthieana** Henrard ex Bor, Kew Bull. 1953: 273. 1953 **

**Type.** India: Madhya Pradesh, Bandelkhand, Barwar Sagor, near Jhansi; December 9, 1886; *J.F. Duthie s.n.* (K).

**Distribution.** Madhya Pradesh, Uttar Pradesh.

**Habit.** Perennial.

**Remarks.** Record for Madhya Pradesh fide Naithani (1990).

**650. **Digitaria eriantha** Steud., Flora 12(2): 468–469. 1829. **

*Digitaria pentzii* Stent, Bothalia 3(1): 147. 1930. Type: Cape Province, Vrijburg District, Vrijburg; *Pentz N.H.P. 8510* (PRE).

*Digitaria eriantha* Steud. var. *stolonifera* Stapf, Fl. Cap. (Harvey) 7: 375. 1898. Type: South Africa: Transvaal, Houtbosch; *A. Rehmann 5712* (K, L). LT designated (as type) by Henrard in Monogr. Digitaria 232. 1950.

**Type.** South Africa: Cape: Cape of Good Hope, *von Ludwig s.n*.

**Distribution.** Maharashtra [also in Africa].

**Habit.** Perennial.

**Remarks. **Possibly only in cultivation.

**651. **Digitaria fuscescens** (J. Presl) Henrard, Meded. Rijks-Herb. 61: 8. 1930 **

*Paspalum fuscescens* J. Presl in C. Presl, Reliq. Haenk. 1(4–5): 213. 1830. Type: “Hab. in regione montana Peruviae” (changed to Hab. ad Monte-Rey Californiae, in the Addendum on p. 351); *Haenke s.n.* (PR).

*Syntherisma fuscescens* (J. Presl) Scribn., Rep. (Annual) Missouri Bot. Gard. 10: 49, t. 10. 1899

*Digitaria pseudoischaemum* Buse, Pl. Jungh. 382. 1854 (as “pseudo-ischaemum”). Type: “Habitat in sumatrae littoralibus arenosis. Inter stirpes Ischaemi multici, cui Sterilis quodammodo similes est, offendi unicum specimen.” *Junghuhn s.n.*

**Distribution.** Manipur, Nagaland, Tamil Nadu [Bhutan, Myanmar, Sri Lanka].

**Habit.** Annual.

**652. **Digitaria griffithii** (Hook. f.) Henrard, Blumea 1(1): 100. 1934 **

*Paspalum sanguinale* (L.) Lam. var. *griffithii* Hook. f., Fl. Brit. India (J.D. Hooker). 7(21): 15. 1896. Type: Cited many syntypes.

*Panicum corymbosum* sensu Thwaites, Enum. Pl. Zeyl. (Thwaites). 436. 1864, non Roxb., 1820

**Distribution.** Karnataka, Kerala, Tamil Nadu, Uttarakhand [Sri Lanka].

**Habit. **Perennial.

**653. **Digitaria ischaemum** (Schreb.) Muhl., Cat. Pl. Amer. Sept. 9. 1813 **

*Panicum ischaemum* Schreb. in Schweigg., Spec. Fl. Erlang. 16. 1804. Type: Germany, Bavaria

*Syntherisma ischaemum* (Schreb.) Nash, N. Amer. Fl. 17(2): 151. 1912

*Digitaria humifusa* Pers., Syn. Pl. (Persoon) 1: 85. 1805. Type: “Hab. In arenosis circa Parisios.”

*Panicum humifusum* (Pers.) Kunth, Révis. Gramin. 1: 33. 1829

*Syntherisma humifusum* (Pers.) Rydb., Mem. New York Bot. Gard. 1: 469. 1900

*Paspalum ambiguum* Lam. & DC., Fl. Franc. (DC. & Lamarck), ed. 3. 3: 16. 1805. Type: cited Paspalum dactylon Lam.

*Digitaria ischaemum* (Schreb.) Muhl., Descr. Gram. (Muhlenberg) 131. 1817, isonym

**Distribution.** India (Northwest Himalaya) [China, Pakistan].

**Habit.** Annual.

**Remarks.** Distribution in India not recorded.

**654. **Digitaria jubata** (Griseb.) Henrard, Blumea 1(1): 100. 1934 **

*Paspalum jubatum* Griseb., Nachr. Königl. Ges. Wiss. Georg-Augusts-Univ. 84. 1868. Type: India: Khasya Hills, 7000 ft.; *J.D. Hooker Paspalum no. 9* (GOET).

**Distribution.** Assam, Meghalaya [China].

**Habit.** Annual.

**655. **Digitaria longiflora** (Retz.) Pers., Syn. Pl. (Persoon) 1: 85. 1805 **

*Paspalum longiflorum* Retz., Observ. Bot. (Retzius) 4: 15. 1786–1787. Type: “E Malabaria, ubi ad margines agrorum crescit, misit hon. Koenig.”

*Panicum longiflorum* (Retz.) J.F. Gmel., Syst. Nat., ed 13 [bis] 2: 158. 1791

*Syntherisma longiflora* (Retz.) Skeels, U.S.D.A. Bur. Pl. Industr. Bull. 261: 30. 1912

*Digitaria caespitosa* Ridl., Fl. Malay. Penin. 5: 215. 1925. Lectotype: Malay Peninsula: Singapore, Tanglin, grass plots in the gardens; *Ridley s.n. *(K). LT designated by Veldkamp, Blumea 21: 64. 1973.

*Panicum propinquum* R. Br., Prodr. Fl. Nov. Holland. 1: 193. 1810. Type: Australia: “Littora Novae Hollandiae intra tropicum”; *R. Brown 6122* (BM).

*Digitaria propinqua* (R. Br.) P. Beauv., Ess. Agrostogr. 160, 170. 1812

*Digitaria roxburghii* Spreng., Syst. Veg. (ed. 16) [Sprengel] 1: 270. 1824 (“1825”). Type: India orientalis

*Paspalum brevifolium* Fluegge, Gram. Monogr., Paspalum 150. 1810 (as “*Paspalus brevifolius*”), nom. superfl. & illeg. Syntypes: “In malabaria ad agrorum margines; *Koenig s.n.* Insula Mauritii; *Dupetit Thouars s.n.* Tranqubaria; *Klein s.n.*

*Paspalum filiculme* Nees in Herb. Wight ex Miq., Prolus. Fl. Jap. 162. 1867

*Digitaria filiculmis* (Nees) Ohwi, Acta Phytotax. Geobot. 11(1): 32. 1942, in obs.

*Paspalum preslii* Kunth, Enum. Pl. (Kunth) 1: 47. 1833. Type: Peruvia

*Digitaria preslii* (Kunth) Henrard, Monogr. Digitaria 589. 1950

*Panicum pseudodurva* Nees, Fl. Afr. Austral. Ill. 1: 21. 1841 (as “pseudo-durva”)

*Panicum parvulum* Trin., Mém. Acad. Imp. Sci. Saint-Pétersbourg, Sér. 6, Sci. Math., Seconde Pt. Sci. Nat. 3, 1(2–3): 205. 1834, nom. superfl. & illeg. for *Paspalum longiflorum* Retz.

**Distribution.** Andhra Pradesh, Bihar, Chhattisgarh, Gujarat, Jammu and Kashmir, Jharkhand, Karnataka, Kerala, Madhya Pradesh, Maharashtra, Meghalaya, Odisha, Rajasthan, Tamil Nadu, Uttarakhand, West Bengal [Bhutan, China, Myanmar, Nepal, Pakistan, Sri Lanka].

**Habit. **Annual.

**656. **Digitaria nodosa** Parl., Pl. Nov. 39. 1842 **

*Syntherisma nodosa* (Parl.) Newbold, Torreya 24: 9. 1924

*Panicum commutatum* Nees var. *nodosum* (Parl.) Hack. ex T. Durand & Schinz, Consp. Fl. Afr. [T.A. Durand & H. Schinz] 5: 744. 1894

*Panicum pabulare* Aitch. & Hemsl., J. Linn. Soc., Bot. 19: 190. 1882. Type: Afghanistan: Kuram District, between Thal and Chapri, also on Tor-ghar Hill

*Paspalum sanguinale* (L.) Lam. var. *pabulare* (Aitch. & Hemsl.) Hook. f., Fl. Brit. India (J.D. Hooker). 7(21): 15. 1896

*Panicum parlatorei* Steud., Syn. Pl. Glumac. 1: 40. 1854. Type: “Ins. Canar., *Parlatore Pl. nov. 39*”

**Type.** “Habitat in arvis insulae Canariae.”

**Distribution.** Punjab [Afghanistan, Pakistan].

**Habit. **Not recorded.

**657. **Digitaria pennata** (Hochst.) T. Cooke, Fl. Bombay 2(5): 941. 1908 var. **pennata

*Panicum pennatum* Hochst., Flora 38: 197. 1855. Type: Abyssinia[Ethiopia]: Gurrsarfa; 1853; *Schimper [Hb. abyss. Buch.] 1497 *(STR).

*Paspalum pennatum* (Hochst.) Hook. f., Fl. Brit. India (J.D. Hooker). 7(21): 16. 1896

**Distribution.** Gujarat, Maharashtra, Rajasthan [Pakistan].

**Habit. **Perennial.

**658. **Digitaria pennata** (Hochst.) T. Cooke var. **shettyana** R.P. Pandey, Parmar & B.L. Vyas, J. Econ. Taxon. Bot. 5(2): 475. 1984 **

**Type.** India: Rajasthan, Jalore, rocky habitat near Sareh Mandir; September 21, 1978; *B.V. Shetty 6659A* (CAL).

**Distribution.** Rajasthan.

**Habit. **Perennial.

**Remarks.** Record for India fide Naithani (1990).

**659. **Digitaria radicosa** (J. Presl) Miq., Fl. Ind. Bat. 3: 437. 1857 **

*Panicum radicosum* J. Presl in C. Presl, Reliq. Haenk. 1(4–5): 297. 1830. Type: “Habitat ad Sorzogon Luzoniae.” Philippine Islands: Luzon, Sorsogon Province; *Haenke s.n.* (PR?).

*Panicum timorense* Kunth, Enum. Pl. (Kunth) 1: 83. 1833

*Digitaria timorensis* (Kunth) Balansa, J. Bot. (Morot) 4: 138. 1890. (See Remarks below.)

*Panicum debile* Desf., Fl. Atlant. 1: 59–60. 1798. Type: Algeria: in pascuis prope La Calle; *Desfontaines 127* (P).

*Paspalum sanguinale* (L.) Lam. var. *debile* (Desf.) Hook. f., Fl. Brit. India (J.D. Hooker). 7(21): 16. 1896

**Distribution.** Andhra Pradesh, Bihar, Chhattisgarh, Gujarat, Jammu and Kashmir, Jharkhand, Karnataka, Kerala, Madhya Pradesh, Maharashtra, Meghalaya, Odisha, Rajasthan, Tamil Nadu, Uttarakhand, West Bengal [China, Myanmar, Nepal, Pakistan].

**Habit.** Annual.

**Remarks.***Digitaria timorensis* (Kunth) Balansa placed in synonymy here, but it is unclear where this name falls within *Digitaria*.

**660. **Digitaria sanguinalis** (L.) Scop., Fl. Carniol., ed. 2. 1: 52. 1771 **

*Panicum sanguinale* L., Sp. Pl. 1: 57. 1753. Type: “Habitat in America, Europa australi.” Lectotype: *Herb. Linn. No. 80.31*(LINN). LT designated by Henrard in Monogr. Digitaria 649. 1950.

*Paspalum sanguinale* (L.) Lam., Tabl. Encycl. 1: 176. 1791

*Digitaria sanguinalis* (L.) Scop. var. *frumentacea* Henrard, Monogr. Digitaria 985. 1950. Type: Cultivated.

*Digitaria sanguinalis* (L.) Scop. var. *rottleriana* Henrard, Monogr. Digitaria 986. 1950. Type: South Africa: Cape Province, Cape town, in gardens Johannesburg and India.

**Distribution.** Andhra Pradesh, Bihar, Gujarat, Jharkhand, Jammu and Kashmir, Karnataka, Kerala, Punjab, Rajasthan, Uttar Pradesh [China, Pakistan]. Naturalized. Native range not recorded.

**Habit. **Annual.

**661. **Digitaria setigera** Roth, Syst. Veg., ed. 15 bis [Roemer & Schultes] 2: 474. 1817 **

*Digitaria setigera* Roth var. *calliblepharata* (Henrard) Veldkamp, Blumea 21(1): 40. 1973

*Panicum corymbosum* Roxb., Fl. Ind. (Carey & Wallich ed.) 1: 295. 1820. Type: India: in the vallies amongst the Circar Mountains; *Roxburgh s.n.*

*Axonopus corymbosus* (Roxb.) Schult., Mant. 2 (Schultes) 177. 1824

*Digitaria corymbosa* (Roxb.) Merr., Enum. Philipp. Fl. Pl. 1: 53. 1923

*Syntherisma corymbosa* (Roxb.) Hosok., Trans. Nat. Hist. Soc. Formosa 24(130): 199. 1934

*Panicum microbachne* J. Presl in C. Presl, Reliq. Haenk. 1: 298. 1830. Lectotype: Philippine Islands: Luzon; *Haenke s.n.* (right- and left-hand specimens) (PR) (vide Henrard, Monogr. Digitaria 450. 1950).

*Digitaria microbachne* (J. Presl) Henrard, Meded. Rijks-Herb. 61: 13. 1930

*Digitaria microbachne* (J. Presl) Henrard ssp. *calliblepharata* Henrard, Monogr. Digitaria 452. 1950. Syntypes: Indonesia: Kangean, Kajoe Waroe; 1920; *Backer 27997* (L). Java: Soerabaja; September 30, 1928; *Van Slooten 2047*

*Panicum dilatatum* Steud., Syn. Pl. Glumac. 1: 39. 1854. Type: India Courtallum; *Wight 1045, 1047*

*Panicum pruriens* Trin., Gram. Panic. [Trinius] 77. 1826. Lectoype: Hawaii: Nukahiwa Island; *G.H. von Langsdorff s.n.* LT designated by Veldkamp in Blumea 21(1): 38. 1973.

*Paspalum sanguinale* (L.) Lam. var. *pruriens* (Trin.) Hook. f., Fl. Brit. India (J.D. Hooker). 7(21): 15. 1896

*Paspalum sanguinale* (L.) Lam. var. *extensum* Hook. f., Fl. Brit. India (J.D. Hooker). 7(21): 15. 1896. Type: *Wight Cat. no. 2340*

*Digitaria extensa* (Hook. f.) Henrard, Blumea 1(1): 100. 1934

**Type.** India; *Heyne s.n.*

**Distribution. **Andhra Pradesh, Arunachal Pradesh, Assam, Bihar, Chhattisgarh, Delhi, Gujarat, Karnataka, Kerala, Madhya Pradesh, Maharashtra, Nagaland, Odisha, Punjab, Rajasthan, Sikkim, Tamil Nadu, Uttar Pradesh, Uttarakhand, West Bengal [Bangladesh, Bhutan, China, Myanmar, Nepal, Pakistan].

**Habit.** Annual.

**662. **Digitaria stewartiana** Bor, Kew Bull. 1951: 166–167. 1951 **

**Type.** India: Kashmir, Chunagund, near Kargil, Ladak, 10000 ft.; August 25, 1940; *R.R. Stewart 21047* (K).

**Distribution.** Jammu and Kashmir, Ladak [China, Pakistan].

**Habit.** Annual.

**Remarks. **Report from Ladak fide Naithani (1990).

**663. **Digitaria stricta** Roth, Syst. Veg., ed. 15 bis [Roemer & Schultes] 2: 474. 1817 var. **stricta

*Setaria stricta* (Roth) Kunth, Révis. Gramin. 1: 47. 1829

*Digitaria stricta* Roth var. *denudata* (Link) Henrard, Monogr. Digitaria 175. 1950

*Digitaria stricta* Roth var. *glabrescens* Bor, Webbia 11: 336. 1955. Syntypes: India: Khasia; *J.D. Hooker & T. Thomson s.n.* Tehri Garhwal, Jumma Valley, alt. 5800 ft.; September 3, 1883; *J.F. Duthie 325*. Dehra Dun, Ramgarh; September 1892; *J.S. Gamble 23932*.

*Agrostis pilosa* Retz., Observ. Bot. (Retzius) 6: 22. 1791. Type: “Ex India orientali mista.” India; *Koenig s.n. *(LD).

*Digitaria denudata* Link, Hort. Berol. [Link] 1: 222. 1827

*Panicum denudatum* (Link) Kunth, Révis. Gramin. 1: 32. 1829

*Digitaria puberula* Link, Hort. Berol. [Link] 1: 223. 1827

*Panicum pseudosetaria* Steud., Syn. Pl. Glumac. 1: 39. 1854 for *Digitaria stricta* Roth. Type: India

*Syntherisma royleana* Newbold, Torreya 24: 9. 1924, nom. superfl.

*Digitaria royleana* Prain, Bengal Pl. 2: 1182. 1903, nom. superfl. & illeg. for *Digitaria puberula*

*Paspalum royleanum* Nees ex Hook. f., Fl. Brit. India (J.D. Hooker). 7(21): 18. 1896, nom. superfl. & illeg. for *Digitaria puberula*

**Type.** India; *B. Heyne s.n.*

**Distribution.** Andhra Pradesh, Bihar, Chhattisgarh, Goa, Karnataka, Madhya Pradesh, Maharashtra, Odisha, Rajasthan, Uttar Pradesh, Uttarakhand [Bhutan, China, Myanmar, Nepal, Pakistan, Sri Lanka].

**Habit.** Annual.

**664. **Digitaria ternata** (Hochst. ex A. Rich.) Stapf, Fl. Cap. (Harvey) 7: 376–377. 1898 **

*Cynodon ternatus* Hochst. ex A. Rich., Tent. Fl. Abyss. 2: 405. 1850–1851. Type: “Crescit in arvis Poae Abyssinicae cultis, prope Adoua (Quartin Dillon, Schimper)”; Ethiopia: Tigray; September 23, 1837; *Schimper 76* (P). LT designated (as “type”) by Henrard, Monogr. Digitaria 738. 1950.

*Panicum ternatum* (Hochst. ex A. Rich.) Hochst. ex Steud., Syn. Pl. Glumac. 1: 40. 1853

*Paspalum ternatum* (Hochst. ex A. Rich.) Hook. f., Fl. Brit. India (J.D. Hooker). 7(21): 17. 1896

*Syntherisma ternata* (Hochst. ex A. Rich.) Newbold, Torreya 24: 9. 1924

*Panicum phaeocarpum* Nees var. *gracile* Nees, Fl. Afr. Austral. Ill. 23. 1841. Type: “In graminosis vallis ad Gekau alt. 800–1000 ft.”

**Distribution.** Andhra Pradesh, Karnataka, Kerala, Madhya Pradesh, Maharashtra, Meghalaya, Nagaland, Sikkim, Tamil Nadu, Uttar Pradesh, West Bengal [Bhutan, China, Myanmar, Nepal].

**Habit.** Annual.

**665. **Digitaria tomentosa** (J. Koenig ex Rottler) Henrard, Blumea 1(1): 100. 1934 **

*Milium tomentosum* J. Koenig ex Rottler, Ges. Naturf. Freunde Berlin Neue Schriften 4: 220. 1803. Type: “Intra fissures rupium in monte Wandiwash.”

*Panicum subeglume* Trin., Mém. Acad. Imp. Sci. Saint-Pétersbourg, Sér. 6, Sci. Math., Seconde Pt. Sci. Nat. 3, 1(2–3): 292. 1834

*Milium capillare* Roth ex Roem. & Schult., Syst. Veg., ed. 15 bis [Roemer & Schultes] 2: 320. 1817, non Rottb., 1778. Type: India orientali; *B. Heyne s.n.*

**Distribution.** Andhra Pradesh, Karnataka, Tamil Nadu [China, Sri Lanka].

**Habit.** Perennial.

**666. **Digitaria violascens** Link, Hort. Berol. [Link] 1: 229. 1827 **

*Paspalum fuscum* J. Presl in C. Presl, Reliq. Haenk. 1(4–5): 214. 1830. Type: Hab. in Luzonia? in Peruviae montanis huanoccensibus? Mexico? Mexico; *Haenke s.n. *(PR).

*Syntherisma fusca* (J. Presl) Scribn., Rep. (Annual) Missouri Bot. Gard. 10: 49, t. 11. 1899

*Digitaria fusca* (J. Presl) Merr., Philipp. J. Sci. 35: 4. 1928, non Chiov., 1919

*Digitaria chinensis* (Nees) A. Camus, Notul. Syst. (Paris) 4: 48. 1923, non Horneman, 1819

**Type.** “Hab. in Brasilia.” Brazil; *Herb. Link 93* (B).

**Distribution.** Assam, Karnataka, Kerala, Maharashtra, Meghalaya, Odisha, Tamil Nadu, Uttar Pradesh, West Bengal [Bhutan, China, Myanmar, Nepal, Pakistan, Sri Lanka].

**Habit.** Annual.

**667. **Digitaria wallichiana** (Wight & Arn. ex Nees) Stapf, Fl. Trop. Afr. [Oliver et al.] 9: 436. 1919 **

*Panicum wallichianum* Wight & Arn. ex Nees, Syn. Pl. Glumac. (Steudel) 1: 41. 1853. Type: India; *Wight Cat. 1607* (KD 3089) (P).

*Panicum multibrachiatum* Hochst. ex Steud., Syn. Pl. Glumac. 1: 74. 1853. Type: India; Nilgiri Mountains; *R.F. Hohenacker 916*

*Paspalum perrottetii* Hook. f., Fl. Brit. India (J.D. Hooker). 7(21): 20. 1896, non *Panicumperrottetii* Kunth, 1833 (vide Bor 1960).

**Distribution.** Karnataka, Kerala, Tamil Nadu [Sri Lanka].

**Habit.** Perennial.

***Echinochloa* P. Beauv., Essai Agrostogr. 53. 1812, nom. cons.**

**Type. ***Echinochloa crus-galli* (L.) P. Beauv. (*Panicum crus-galli* L.)

**668. **Echinochloa colona** (L.) Link, Hort. Berol. [Link] 2: 209. 1833 **

*Panicum colonum* L., Syst. Nat., ed. 10. 2: 870. 1759. Type: “Habitat [in Indiae cultis.] Sp. Pl., ed. 2. 1: 84. 1753.” Lectotype: Browne, Herb. Linn. No. 80.23 (LINN). LT designated by Hitchcock in Contr. U. S. Natl. Herb. 12: 119. 1908.

*Milium colonum* (L.) Moench, Methodus (Moench) 202. 1794

*Oplismenus colonus* (L.) Kunth, Nov. Gen. Sp. [H.B.K.] 1: 108–109 (ed. qto.). 1816

*Echinochloa crus-galli* (L.) P. Beauv. ssp. *colona* (L.) Honda, Bot. Mag. (Tokyo) 37: 122. 1923

*Panicum crus-galli* L. ssp. *colonum* (L.) Makino & Nemoto, Fl. Japan (Makino & Nemoto) 1470. 1925

*Panicum crus-galli* L. var. *minor* Thwaites, Enum. Pl. Zeyl. (Thwaites). 359. 1864. Type: Ceylon Plant 901

*Panicum zonale* Guss., Fl. Sic. Prodr. 1: 82. 1827. Type: “Habitus praecedentis sed gracilior, spiculae minus hispidae, antherae sordide purpureae, stigmata nigricantia.”

*Echinochloa zonalis* (Guss.) Parl., Fl. Panorm. 1: 119. 1839

*Oplismenus repens* J. Presl in C. Presl, Reliq. Haenk. 1(4–5): 321. 1830. Type: “Habitat in Mexico.” Mexico; *Haenke s.n.* (PR).

*Panicum prorepens* Steud., Syn. Pl. Glumac. 1: 46. 1853

**Distribution.** Andaman and Nicobar Islands, Andhra Pradesh, Arunachal Pradesh, Bihar, Chhattisgarh, Goa, Jharkhand, Karnataka, Kerala, Madhya Pradesh, Maharashtra, Manipur, Meghalaya, Odisha, Rajasthan, Tamil Nadu,Tripura, Uttar Pradesh [China, Pakistan].

**Habit.** Annual.

**669. **Echinochloa crus-galli** (L.) P. Beauv., Ess. Agrostogr. 161, 169. 1812 var. **crus-galli

*Panicum crus-galli* L., Sp. Pl. 1: 56. 1753 (as “crus galli”). Type: “Habitat in Europae, Virginiae cultis.” Lectotype: *Kalm, Herb. Linn. No. 80.18 *(LINN). LT designated by Hitchcock in Contr. U. S. Natl. Herb. 12: 117. 1908.

*Milium crus-galli* (L.) Moench, Methodus (Moench) 202. 1794

*Pennisetum crus-galli* (L.) Baumg., Enum. Stirp. Transsilv. 3: 277. 1817

*Echinochloa crus-galli* (L.) P. Beauv. var. *submutica* Neilr., Fl. Nied.-Oesterr 2: 31. 1859 (as “submuticum”). Type: *Echinochloa crus-galli Rchb. Icon. 9. f. 1411*

*Echinochloa glabrescens* Munro ex Eggel., Ann. List. Gram. Uganda 16. 1947, non Murno ex Hook. f, 1896

*Panicum hispidulum* Retz., Observ. Bot. (Retzius) 5: 18. 1788 (“1789”). Type: “Ex India Orientali misit honor. Koenig.”

*Echinochloa hispidula* (Retz.) Nees, Ill. Bot. Himal. Mts. 1: 416, 420. 1840

*Panicum crus-galli* L. var. *stagninum* Trimen ex Hook. f. in Trimen, Handb. Fl. Ceylon 5: 136. 1900, non *Panicum stagninum* Retz., 1788 [Trimen in Handb. Fl. Ceylon published it as com. nov.]

*Panicum grossum* Salisb., Prodr. Stirp. Chap. Allerton 18. 1796, nom. superfl. & illeg.

**Distribution.** Andaman and Nicobar Islands, Andhra Pradesh, Arunachal Pradesh, Assam, Bihar, Delhi, Goa, Jharkhand, Karnataka, Kerala, Madhya Pradesh, Maharashtra, Manipur, Odisha, Rajasthan, Tamil Nadu, Tripura, Uttar Pradesh, Uttarakhand [China, Nepal, Sri Lanka].

**Habit.** Annual.

**670. **Echinochloa crus-pavonis** (Kunth) Schult., Mant. 2 (Schultes) 269. 1824 **

*Oplismenus crus-pavonis* Kunth, Nov. Gen. Sp. [H.B.K.] 1: 108 (ed. qto.). 1816. Type: “Crescit in apricis calidissimis Procinciae Cumanensis prope Bordones.”

*Echinochloa composita* J. Presl ex Nees, Fl. Bras. Enum. Pl. 2(1): 259. 1829 [cited as synonym of *E*. *crus-pavonis*]. Type: “Habitat in sylvis ad Almada et Ferradas provinciae Bahiensis, aliisque in locis Brasiliae”; *Haencker s.n.* (B).

*Oplismenus angustifolius* E. Fourn., Mexic. Pl. 2: 40. 1886. Type: Mexico: Veracruz; August; *Gouin 54* (P).

*Oplismenus jamaicensis* Kunth, Enum. Pl. (Kunth) 1: 147. 1833. Type: Jamaica

*Panicum jamaicense* (Kunth) Steud., Nomencl. Bot., ed. 2 (Steudel) 2: 257. 1841

*Panicum sabulicolum* Nees, Fl. Bras. Enum. Pl. 2(1): 258. 1829. Type: “Habitat in arenosis Parae; *Sieber s.n.*” Uruguay: Montevideo; *Sellow s.n.*

*Oplismenus sabulicolus* (Nees) Kunth, Enum. Pl. (Kunth) 1: 145. 1833 (as “sabulicola”)

*Panicum aristatum* MacFad., Bot. Misc. 2: 115. 1831, non Retz., 1786–1787

**Distribution.** Andaman and Nicobar Islands, Uttar Pradesh, Uttarakhand [China].

**Habit.** Perennial.

**671. **Echinochloa frumentacea** Link, Hort. Berol. [Link] 1: 204. 1827 **

*Echinochloa colona* (L.) Link var. *frumentacea* (Link) Ridl., Fl. Malay. Penin. 5: 223. 1925

*Oplismenus frumentaceus* (Link) Kunth, Révis. Gramin. 1: 45. 1829

*Panicum crusgalli* L. var. *frumentacea* (Link) Trimen, Syst. Cat. Fl. Pl. Ceylon 104. 1885 (as “frumentaceum”)

*Panicum frumentaceum* Roxb., Fl. Ind. (Carey and Wallich ed.) 1: 307. 1820, non Salisb., 1796. Type: “This I have only found in a state of cultivation, ..”

**Type.** India; Cultivated.

**Distribution.** Andhra Pradesh, Arunachal Pradesh, Assam, Bihar, Daman and Diu, Delhi, Goa, Jammu and Kashmir, Karnataka, Kerala, Madhya Pradesh, Maharashtra, Meghalaya, Odisha, Rajasthan, Sikkim, Tamil Nadu, Uttar Pradesh, Uttarakhand, West Bengal [also in Asia, Africa, and introduced to America].

**Habit.** Annual.

**672. **Echinochloa glabrescens** Munro ex Hook. f., Fl. Brit. India (J.D. Hooker). 7(21): 31. 1896 **

**Type. **Western Himalaya, Khasia Hills; Numer. List [Wallich] no. 8687.

**Distribution.** Karnataka, Meghalaya, Rajasthan, Tamil Nadu [Afghanistan, Bhutan, China, Nepal, Pakistan; Africa].

**Habit. **Not recorded.

**673. **Echinochloa oryzoides** (Ard.) Fritsch, Verh. K. K. Zool.-Bot. Ges. Wien 41: 742. 1891 **

*Panicum oryzoides* Ard., Animadv. Bot. Spec. Alt. 16. t. 5. 1764. Type: “Semina hujus Panici inventa a me fuere inter Oryzam.”

**Distribution. **Jammu and Kashmir, Tamil Nadu [China, Pakistan].

**Habit.** Annual.

**Remarks.** Record for Jammu and Kashmir fide Naithani (1990).

**674. **Echinochloa picta** (J. Koenig) P.W. Michael, Philipp. Weed J. Sci. 5: 18. 1978 **

*Panicum pictum* J. Koenig, Naturforscher (Halle) 23: 204. 1788. Type: India: Calcutta; *J. Koenig s.n. *

**Distribution.** Karnataka, Tamil Nadu [China].

**Habit.** Perennial.

**675. **Echinochloa pyramidalis** (Lam.) Hitchc. & Chase, Contr. U. S. Natl. Herb. 18: 345. 1917 **

*Panicum pyramidale* Lam., Tabl. Encycl. 1: 171. 1791. Type: “Lieu nat. le Senegal. Bdles courtes, Blanchatres.” Senegal: E. Senegal; 1789; *D. Roussillon s.n. *(P-LAM).

*Panicum atroviolaceum* A. Rich., Tent. Fl. Abyss. 2: 368. 1850–1851. Type: “Crescit in Abyssinia sine locali indicatione; *Antoine Petit s.n.*” (P).

*Panicum quadrifarium* Hochst. ex A. Rich., Tent. Fl. Abyss. 2: 367. 1850–1851. Type: “Crescit ad marginem stagnorum et in locis paludosis prope Adoua, mensibus Novembre et Decembre.” Syntypes: Ethiopia: Tigre, Adoua; December 1, 1837; *Schimper 206* (P). Ethiopia: Adoua, *Quartin Dillon s.n.* (P).

**Distribution.** Karnataka [also in Saudi Arabia, Africa, Australasia, North America, South America].

**Habit.** Perennial.

**676. **Echinochloa stagnina** (Retz.) P. Beauv., Ess. Agrostogr. 161, 171. 1812 **

*Panicum stagninum* Retz., Observ. Bot. (Retzius) 5: 17. 1788 (“1789”). Type: ‘Habitat in stagnis Indiae Orientalis, D. Koenig.” India; *Koenig s.n. *(LD).

*Panicum crus-galli* sensu Hook. f., Fl. Brit. India (J.D. Hooker). 7(22): 30. 1896, non L. 1753

**Distribution.** Andaman and Nicobar Islands, Andhra Pradesh, Assam, Bihar, Chhattisgarh, Karnataka, Kerala, Gujarat, Madhya Pradesh, Maharashtra, Manipur, Meghalaya, Rajasthan, Tamil Nadu, Tripura [Pakistan, Sri Lanka].

**Habit.** Perennial.

***Eriochloa* Kunth, Nov. Gen. Sp. [H.B.K.] 1: 94 (ed. qto.). t. 30. 31. 1816**

**Lectotype. ***Eriochloa distachya* Kunth. LT designated by G.V. Nash in N. Amer. Fl. 17: 157. 1912; affirmed by Hitchcock, Bull. U.S.D.A. 772: 220. 1920.

**677. **Eriochloa fatmensis** (Hochst. & Steud.) Clayton, Kew Bull. 30(1): 108. 1975 **

*Panicum fatmense* Hochst. & Steud., Unio Itin. Schimper 806. 1837. Type: Arabia: Mecca; *Schimper 806* (K).

*Eriochloa decumbens* F.M. Bailey, Queensland Agric. J. 1: 234. 1897. Type: “Habitat on rocks, Hammond Island, Torres Strait. This grass differs from the other Australian species of the genus, principally in habit.”

*Helopus nubicus* Steud., Syn. Pl. Glumac. 1: 100. 1854. Type: Sudan: Arashcool; *Kotschy 382* (K).

*Eriochloa nubica* (Steud.) Hack. & Stapf ex Thell., Vierteljahrsschr. Naturf. Ges. Zürich 64: 697. 1919, in obs.

*Helopus acrotrichus* Steud., Syn. Pl. Glumac. 1: 100. 1854, nom. superfl. & illeg. for P. fatamense

*Eriochloa acrotricha* Hack., Vierteljahrsschr. Naturf. Ges. Zürich 52: 435. 1907, non (Hook. f.) Schinz, 1906

**Distribution.** Maharashtra, Rajasthan [China, Myanmar, Sri Lanka].

**Habit.** Annual.

**Remarks. **Records for India fide Naithani (1990).

**678. **Eriochloa procera** (Retz.) C.E. Hubb., Bull. Misc. Inform. Kew 1930: 256. 1930 **

*Agrostis procera* Retz., Observ. Bot. (Retzius) 4: 19. 1786–1787. Type: “In graminosis subhumidis Malabariae frequens; Koenig.”

*Milium ramosum* Retz., Observ. Bot. (Retzius) 6: 22. 1791. Type: “Ex India Orientali Habui.”

*Agrostis ramosa* (Retz.) Poir., Encycl. (Lamarck) Suppl. 1: 257. 1810

*Eriochloa ramosa* (Retz.) Kuntze, Revis. Gen. Pl. 2: 775. 1891

*Eriochloa polystachya* sensu Hook. f., Fl. Brit. India (J.D. Hooker). 7(21): 20. 1896, non H.B.K., 1816

*Eriochloa annulata* Kunth, Révis. Gramin. 1: 30. 1829, nom. superfl. & illeg.

*Paspalum annulatum* Fluegge, Gram. Monogr., Paspalum 133. 1810 (as “Paspalus annulatus”), nom. superfl. & illeg. for *Milium ramosum* Retz.

**Distribution.** Andhra Pradesh, Arunachal Pradesh, Assam, Bihar, Chhattisgarh, Dadra and Nagar Haveli, Delhi, Goa, Gujarat, Karnataka, Kerala, Madhya Pradesh, Maharashtra, Nagaland, Odisha, Punjab, Rajasthan, Tamil Nadu, Uttar Pradesh, West Bengal [China, Myanmar, Nepal, Sri Lanka].

**Habit. **Annual.

***Holcolemma* Stapf & C.E. Hubb., Bull. Misc. Inform. Kew 1929: 244. 1929**

**Type. ***Holcolemma canaliculatum* (Nees) Stapf & C.E. Hubb. (*Panicum canaliculatum* Nees ex Steud.).

**679. **Holcolemma canaliculatum** (Nees) Stapf & C.E. Hubb., Bull. Misc. Inform. Kew 1929: 246. 1929 **

*Panicum canaliculatum* Nees, Syn. Pl. Glumac. (Steudel) 1: 55. 1853. Type: India; *Wright 1624* (P).

*Hemigymnia canaliculata* (Nees) Alston, Handb. Fl. Ceylon 6: 324. 1931

*Panicum stenostachyum* Thwaites, Enum. Pl. Zeyl. (Thwaites). 436. 1864. Type: Sri Lanka: Trincomalie; *Rev S.O. Glemie Ceylon Plant 3845*

**Distribution.** Tamil Nadu [Sri Lanka].

**Habit.** Perennial.

***Megathyrsus* (Pilg.) B.K. Simon & S.W.L. Jacobs, Austrobaileya 6(3):572. 2003**

*Panicum* subgen. *Megathyrsus* Pilg., Notizbl. Bot. Gart. Berlin-Dahlem 11: 242. 1931

**Lectotype. ***Megathyrsus maximus *(Jacq.) B.K. Simon & S.W.L. Jacobs (*Panicum maximum *Jacq.) Designated by Zuloaga, Grass Syst. Evol. 296. 1987.

**680. **Megathyrsus maximus** (Jacq.) B.K. Simon & S.W.L. Jacobs, Austrobaileya 6(3): 572. 2003 **

*Panicum maximum* Jacq., Ic. Pl. Rar. 1: 2. t. 13. 1781. Type: West Indies: Lesser Antilles: Leeward Islands, Guadeloupe; *Herb. Jacquin s.n.* (W).

*Urochloa maxima* (Jacq.) R.D. Webster, Austral. Paniceae (Poaceae) 241. 1987

*Panicum giganteum* Mez, Bot. Jahrb. Syst. 34: 143. 1904, non Scheele, 1849

*Panicum heynii* Roth, Syst. Veg., ed. 15 bis [Roemer & Schultes]. 2: 458. 1817. Type: India; *Heyne s.n.*

*Panicum hirsutissimum* Steud., Syn. Pl. Glumac. 1: 72. 1853. Type: “In sylvis Krakakammae et ad flumen Zwartkopsrivier, alt. I (Uitenhage), (Ecklon), inter frutices ad flumen Zontagsrivier et Zoutpansnek alt. 1500–2000 et ad rivum ad Enon alt. 400, (Drege).”

*Panicum maximum* Jacq. var. *hirsutissimum* (Steud.) Oliv., Trans. Linn. Soc. London 29(3): 171. 1875

*Panicum jumentorum* Pers., Syn. Pl. (Persoon) 1: 83. 1805

*Panicum laeve* Lam., Tabl. Encycl. 1: 172. 1791. Type: Dominican Republic: Hispaniola Island; 1780; *E Domingo, ins. Franc.* (P-LAM).

*Panicum maximum* Jacq. ssp. *pubescens* M. Sharma, J. Econ. Taxon. Bot. 7(1): 106. f. 1. 1985. Type: India: Punjab, Patiala District, Rakhra; alt. 700 ft.; September 1, 1971; *M. Sharma 2288* (PUN).

*Panicum pamplemoussense* Steud., Syn. Pl. Glumac. 1: 71. 1853. Type: “Urville legit ad Pamplemousse Ins. Maurit.”

*Panicum polygamum* Sw., Prodr. (Swartz) 24. 1788, non Forssk., 1775. Type: India Orientali; *Brown 366*

*Panicum sparsum* Schumach., Beskr. Guin. Pl. 64. 1827

**Distribution.** Andaman and Nicobar Islands, Andhra Pradesh, Bihar, Chhattisgarh, Delhi, Goa, Gujarat, Jharkhand, Karnataka, Kerala, Madhya Pradesh, Maharashtra, Odisha, Punjab, Rajasthan, Tamil Nadu, Uttar Pradesh, West Bengal [China].

**Habit.** Perennial herb.

**Remarks. **Record for Punjab fide Naithani (1990).

***Melinis* P. Beauv., Ess. Agrostogr. 54. 1812**

**Type. ***Melinis minutiflora* P. Beauv.

**681. **Melinis minutiflora** P. Beauv., Ess. Agrostogr. 54. t. 11. f. 4. 1812 **

*Panicum minutiflorum* (P. Beauv.) Raspail, Ann. Sci. Nat. (Paris) 5: 299. 1825

*Muhlenbergia brasiliensis* Steud., Syn. Pl. Glumac. 1: 177. 1854. Type: Brazil: Bahia; 1830; *M. Salzmann 652* (US-87244).

*Panicum melinis* Trin., Mém. Acad. Imp. Sci. Saint-Pétersbourg, Sér. 6, Sci. Math. Seconde Pt. Sci. Nat. 3, 1(2–3): 291. 1834

*Suardia picta* Schrank, Pl. Rar. Hort. Monac. t. 58. 1820. Type: “Colitur in Tepidario.”

*Tristegis glutinosa* Nees, Horae Phys. Berol. 47: 54. 1820. Type: Brasilia?

**Type.** Brazil: “Cette plante croit a Rio-Janerio. Elle m’a été communiquée par M. De Jussieu.”(G).

**Distribution.** Kerala, Tamil Nadu [China, Pakistan]. Naturalized.

**Habit.** Perennial.

**682. **Melinis repens** (Willd.) Zizka, Biblioth. Bot. 138: 55. 1988 **

*Saccharum repens* Willd., Sp. Pl., ed. 4 [Willdenow] 1(1): 322. 1797. Type: “Habitat in Guinea.” Africa: Ghana; *Isert s.n.* [Thonning and other Danish Botanists] (B-W-1499).

*Rhynchelytrum repens* (Willd.) C.E. Hubb., Bull. Misc. Inform. Kew 1934: 110. 1934

*Tricholaena repens* (Willd.) Hitchc., Man. Grasses W. Ind. 331. 1936

*Monachyron villosum* Parl., Niger Fl. 191. 1849. Type: Cape Verde Islands: S. Jacobi; *J.D. Hooker s.n.* (K). “Hab. in insula S. Jacobi (J.D. Hooker).

*Rhynchelytrum villosum* (Parl.) Chiov., Ann. Bot. (Rome) 8(3): 310. 1908 (as “Rhynchelitrum”)

*Melinis villosa* (Parl.) Hack., Oesterr. Bot. Z. 51: 464. 1901

*Rhynchelytrum roseum* (Nees) Stapf & C.E. Hubb. ex Bews, World’s Grass. 223. 1929 in obs. [reference to basionym is not given]

*Tricholaena rosea* Nees, Fl. Afr. Austral. Ill. 17. 1841. Type: Cited many synonyms.

*Tricholaena wightii* Nees & Arn. ex Steud., Syn. Pl. Glumac. 1: 93. 1854, nom. nud., pro. syn.

**Distribution.** Andhra Pradesh, Assam, Karnataka, Kerala, Madhya Pradesh, Maharashtra, Odisha, Rajasthan, Tamil Nadu, Uttar Pradesh [China, Pakistan].

**Habit.** Annual or perennial.

***Moorochloa* Veldkamp (2004), Reinwardtia 12(2): 138–139**

**Type. ***Moorochloa eruciformis* (Sm.) Veldkamp (*Panicum eruciforme* Sm.).

**683. **Moorochloa eruciformis** (Sm.) Veldkamp, Reinwardtia 12(2): 139. 2004 var. **eruciformis

*Panicum eruciforme* Sm., Fl. Graec. (Sibthorp) 1: 44, t. 59. 1806. Type: Greece: Samos “in arvis circa Junonis templum”; *Sibthorp s.n. *(OXF).

*Urochloa eruciformis* (Sm.) C. Nelson & Fern. Casas, Fontqueria 51: 4. 1998

*Brachiaria eruciformis* (Sm.) Griseb., Fl. Ross. (Ledeb.) 4: 469. 1853

*Echinochloa eruciformis* (Sm.) C. Koch, Linnaea 21: 437. 1848

*Panicum caucasicum* Trin., Sp. Gram. [Trinius] 3(22): t. 262. 1830. Type: “Figura ad specimen a Caucaso orientali.”

*Panicum isachne* Roth, Syst. Veg., ed. 15 bis [Roemer & Schultes] 2: 458. 1817. Type: India; *Heyne in Herb. Roth s.n. *= Numer. List [Wallich] no. 8693 (K).

*Panicum isachne* Roth var. *mexicanum* Beal, Grasses N. Amer. 2: 114. 1896 (as “mexicana”). Type: “Specimen seen was cultivated from seed obtained in Mexico, by U.S. Dept. Agricul 1887.”

*Brachiaria isachne* (Roth) Stapf, Fl. Trop. Afr. [Oliver et al.] 9: 552. 1919

*Panicum pubinode* Hochst. ex A. Rich., Tent. Fl. Abyss. 2: 363. 1850–1851. Type: “Crescit in convalle fluvii Tacazze, Schimper, pl. Schimp. Abyss., sect. III, 1855.” Abyissinia: Tacazze River; December, 1841; *Schimper 1855 *

*Panicum wightii* Nees, Fl. Afr. Austral. Ill. 29. 1841. Type: “In graminosis vallis ad Gekau alt. 500–1000.” South Africa; Drége s.n. LT designated by Chase in Contr. U.S. Natl. Herb. 22: 36. 1920.

**Distribution.** Andhra Pradesh, Assam, Bihar, Chhattisgarh, Goa, Gujarat, Jammu and Kashmir, Karnataka, Kerala, Madhya Pradesh, Maharashtra, Odisha, Rajasthan, Tamil Nadu, West Bengal [also in Malaysia, Papua New Guinea, Africa, Australia, Europe, South America].

**Habit.** Annual.

**684. **Moorochloa eruciformis** (Sm.) Veldkamp var. **divaricata** (Basappa & Muniy.) E. A. Kellogg, comb. nov. **

urn:lsid:ipni.org:names:77212058-1

*Brachiariaeruciformis* (Sm.) Veldkamp var. *divaricata* Basappa & Muniy., Proc. Indian Natl. Sci. Acad., B, 49(4): 379. 1983. Type: India: Karnataka, Mysore, 3 km west of Mysore, along the edge of tank-bund; 12 Aug 1979

**Distribution.** Andhra Pradesh, Goa, Gujarat, Jammu and Kashmir, Karnataka (Naithani), Madhya Pradesh, Maharashtra, Rajasthan, Tamil Nadu [also in Africa, Europe].

**Habit. **Annual.

**Remarks.** The above combination is made here to transfer *Brachiaria eruciformis* (Sm.) Veldkamp var. *divaricata* Basappa & Muniy. to *Moorochloa*.

***Oplismenus* P. Beauv., Fl. Oware 2: 14. 1810 (“1807”), nom. cons.**

**Type. ***Oplismenus africanus* P. Beauv.

**685. **Oplismenus burmanni** (Retz.) P. Beauv., Ess. Agrostogr. 168, 169. 1812 **

*Panicum burmanni* Retz., Observ. Bot. (Retzius) 3: 10. 1783 (as “Burmanni”). Type: India: Madras; *Koenig s.n.* (LD).

*Oplismenus affinis* J. Presl in C. Presl, Reliq. Haenk. 1(4–5): 323. 1830, non Schult.,1824. Type: “Habitat in Panama.”

*Panicum bromoides* Lam., Tabl. Encycl. 1: 170. 1791 (“bromoide”). Type: Lieu nat. les I’lle de France.” *Commerson s.n.* (P).

*Oplismenus bromoides* (Lam.) P. Beauv., Ess. Agrostogr. 168, 169. 1812

*Oplismenus cristatus* J. Presl in C. Presl, Reliq. Haenk. 1(4–5): 323. 1830. Type: “Habitat in Mexico.” Mexico; *Haenke s.n.* (PR)

*Oplismenus multisetus* Hochst. ex A. Rich., Tent. Fl. Abyss. 2: 377. 1850–1851. Type: Crescit in convalle fluvii Tacazzé prope Tchélatchéranne, sub arborum umbra, mense Septembre, Schimper, Pl. Schimp.Abyss., sect. III, 1469.” Ethiopia; *Schimper 1469*

*Oplismenus preslei* Kunth, Enum. Pl. (Kunth) 1: 141. 1833. Type: Panama

*Panicum album* Poir., Encycl. (Lamarck) Suppl. 4: 274. 1816. Type: “Cette plante croit a l’ile de Java (Herb. Desfontaine).”

*Orthopogon albus* Nees ex Steud., Syn. Pl. Glumac. 1: 44. 1853, pro syn. of *Panicum album*.

*Panicum japonicum* Steud., Flora 29: 18. 1846. Type: “Die japanischen Graser der Goringschen Sammlung”

*Panicum multisetum* Hochst. ex A. Rich., Tent. Fl. Abyss. 2: 377. 1850–1851 [cited as synonym of *O*. *multisetus*]

*Panicum hirtellum* sensu Burm. f., Fl. Ind. (N. L. Burman) 24. t. 12. f. 1. 1768, non L., 1763

*Oplismenus humboldtianus* Nees, Fl. Bras. Enum. Pl. 2(1): 264. 1829, nom. superfl. & illeg. for *O*. *burmanni*

*Oplismenus indicus* Duthie, Grass.n. W. India 8. 1883, non Roem. & Schult., 1817

**Distribution.** Andhra Pradesh, Arunachal Pradesh, Assam, Bihar, Chhattisgarh, Goa, Gujarat, Karnataka, Kerala, Rajasthan, Maharashtra, Meghalaya, Odisha, Tamil Nadu, Tripura, Uttar Pradesh, Uttarakhand, West Bengal [China, Sri Lanka].

**Habit.** Not recorded.

**686. **Oplismenus compositus** (L.) P. Beauv., Ess. Agrostogr. 168,169. 1812 **

*Panicum compositum* L., Sp. Pl. 1: 57. 1753. Type: “Habitat in Zeylona.” Lectotype: *Herb. Hermann 3: 45, No. 42 *(BM-000621970). LT designated by Davey & Clayton in Kew Bull. 33: 156. 1978.

*Orthopogon compositus* (L.) R. Br., Prodr. Fl. Nov. Holland. 1: 194. 1810

*Andropogon undatus* Jacq., Collectanea [Jacquin] 3: 237. 1789. Type: “Crescit in insula Mauritii.”

*Panicum undatum* (Jacq.) Steud., Nomencl. Bot., ed. 2 (Steudel) 2: 264. 1841

*Panicum lanceolatum* Retz., Observ. Bot. (Retzius) 5: 17. 1788 (“1789”). Type: “Misit opt. Koenig.”

*Echinochloa lanceolata* (Retz.) Roem. & Schult., Syst. Veg., ed. 15 bis [Roemer & Schultes] 2: 476. 1817

*Oplismenus decompositus* Nees, Prodr. Fl. Norfolk. 19. 1833. Type: India; *Wight 62* (NY).

*Oplismenus elatior* (L. f.) P. Beauv., Ess. Agrostogr. 168, 169. 1812

*Oplismenus jacquinii* Kunth, Révis. Gramin. 1: 44. 1829

*Orthopogon pratensis* Spreng., Syst. Veg. (ed. 16) [Sprengel] 1: 306. 1824 (“1825”). Lectotype: Mauritius; *St. Vincent 43*(B-W). LT designated by H. Scholz in Phan. Monogr. 13: 100. 1981.

*Oplismenus pratensis* (Spreng.) Schult. & Schult. f., Mant. 3 (Schultes & Schultes f.) Add. I 597. 1827

*Orthopogon junghuhnii* Nees in Miq., Fl. Ned. Ind. 3: 444. 1857 (“1855”). Type: Java; *Junghuhn s.n.*

*Orthopogon longeracemosus* (Steud.) Miq., Fl. Ned. Ind. 3: 443. 1857(“1855”)

*Orthopogon remotus* Trin., Fund. Agrost. (Trinius) 181. 1820

*Orthopogon sylvaticus* (Lam.) Miq., Fl. Ned. Ind. 3: 443. 1857(“1857”)

*Panicum sylvaticum* Lam., Encycl. (Lamarck) 4(2): 733. 1798. Type: “Cette plante croit naturellement a I’llsle-de-france dans les bois, ou elle a été recueillie par Commerson.” Mauritius; *P. Commerson s.n.* (L).

*Oplismenus sylvaticus* (Lam.) Roem. & Schult., Syst. Veg., ed. 15 bis [Roemer & Schultes] 2: 481. 1817

*Panicum aristatum* Retz., Observ. Bot. (Retzius) 4: 17. 1786–1787. Type: “E China adduxit plur. Rever. D. Wennerberg.” China; *Wennerberg s.n. *(LD).

*Panicum bidentulum* Steud., Syn. Pl. Glumac. 1: 45. 1853. Lectotype: Rebo Running; *Nees s.n.* (B). LT designated by Scholz in Phan. Monogr. 13: 86. 1981. (vide TROPICOS)

*Panicum certificandum* Steud., Syn. Pl. Glumac. 1: 44. 1853. Type: India: Bengal; *Rottler 869* (M).

*Panicum longeracemosum* Steud., Syn. Pl. Glumac. 1: 45. 1853. Type: Indonesia: Java; *Zollinger 915 & 919*

*Panicum peninsulanum* Steud., Syn. Pl. Glumac. 1: 44. 1853. Type: India; *Rottler s.n.*

*Oplismenus lanceolatus* (Retz.) Kunth, Révis. Gramin. 1: 45. 1829, nom. superfl. & illeg for *Andropogon undatus*

**Distribution.** Andaman and Nicobar Islands, Andhra Pradesh, Arunachal Pradesh, Assam, Bihar, Chhattisgarh, Delhi, Goa, Himachal Pradesh, Jharkhand, Jammu and Kashmir, Karnataka, Kerala, Madhya Pradesh, Maharashtra, Manipur, Meghalaya, Nagaland, Odisha, Rajasthan, Sikkim, Tamil Nadu, Tripura, Uttar Pradesh, Uttarakhand [China, Sri Lanka]. Pantropical.

**Habit.** Not recorded.

**687. **Oplismenus undulatifolius** (Ard.) P. Beauv., Ess. Agrostogr. 171 1812 **

*Panicum undulatifolium* Ard., Animadv. Bot. Spec. Alt. 14, t. 4. 1764. Type: “invento in Jamaica a Clariss. Brown”; *Arduino s.n.* (M).

*Orthopogon undulatifolius* (Ard.) Spreng., Syst. Veg. (ed. 16) [Sprengel] 1: 306. 1824 (“1825”)

*Panicum barbifultum *Hochst. ex Schltdl., Linnaea 31(3): 307. 1861. Type: India: Orissa, “in silvis pr. Kaity mont. Nilagir”, *E. Hohenacker 1279 *(US-1445121).

**Distribution.** Jammu and Kashmir, Himachal Pradesh, Kerala, Meghalaya, Punjab, Sikkim, Tamil Nadu, Uttar Pradesh [China, Sri Lanka].

**Habit.** Not recorded.

***Ottochloa* Dandy, J. Bot. 69: 54. 1931**

*Hemigymnia* Stapf in D. Prain, Fl. Trop. Africa 9: 741. 1920, non Griff. (1842)

**Type. ***Ottochloa nodosa* (Kunth) Dandy (*Panicum nodosum* Kunth).

**688. **Ottochloa nodosa** (Kunth) Dandy, J. Bot. 69: 55. 1931 **

*Panicum nodosum* Kunth, Enum. Pl. (Kunth) 1: 97. 1833

*Panicum arnottianum* Nees, Syn. Pl. Glumac. (Steudel) 1: 59. 1853. Type: India orientalis; *Wight s.n.* Java, Sri Lanka

*Hemigymnia arnottiana* (Nees) Stapf, Fl. Trop. Afr. [Oliver et al.] 9: 742. 1920

*Ottochloa arnottiana* (Nees Dandy, J. Bot. 69: 55. 1931

*Panicum multinode* J. Presl in C. Presl, Reliq. Haenk. 1(4–5): 303. 1830, non Lam., 1798. Type: “Habitat ad Sorzogon Luzoniae.” Philippine Islands: Luzon, Sorsogon Province; *Haenke s.n.* (US-80779)

*Hemigymnia multinodis* Stapf, Fl. Trop. Afr. [Oliver et al.] 9: 742. 1920, in obs., nom. superfl. & illeg.

**Distribution.** Assam, Karnataka, Kerala, Manipur, Meghalaya, Nagaland, Sikkim, Tamil Nadu [China, Myanmar, Sri Lanka].

**Habit.** Perennial.

***Panicum* L., Sp. Pl. 1: 55. 1753**

**Lectotype. ***Panicum miliaceum* L. LT designated by Nash in N. L. Britton & A. Brown, Ill. Fl. N.U.S. ed. 2. 1: 134. 1913; affirmed by Hitchcock, Bull. U.S.D.A. 772: 229. 1920.

**689. **Panicum antidotale** Retz., Observ. Bot. (Retzius) 4: 17. 1786–1787 **

**Type.** “Colitur in hortis Malabarorum. Honor. Koening.” India; *Koenig s.n.* (LD)

**Distribution. **Bihar, Delhi, Gujarat, Himachal Pradesh, Jammu and Kashmir, Karnataka, Kerala, Maharashtra, Odisha, Punjab, Rajasthan, Tamil Nadu, Uttar Pradesh [Afghanistan, China, Myanmar, Pakistan].

**Habit.** Perennial.

**690. **Panicum atrosanguineum** Hochst. ex A. Rich., Tent. Fl. Abyss. 2: 375. 1850–1851 **

*Panicum hydaspicum* Edgew., J. Proc. Linn. Soc., Bot. 6: 207. 1862. Type: “Rechnab Bar.” [Flor. Mallica]

**Type. **“Crescit in locis humidis convallis fluvii Tacazze, prope Tchelatcheranne, mense Augusto, Schimper, in pl. Schimp. Abyss., sect. III 1709.” Ethiopia: Djeladjeranne; August 13, 1840; *Schimper 1709* (P).

**Distribution.** Maharashtra, Odisha, Rajasthan [Pakistan].

**Habit.** Annual.

**691. **Panicum bisulcatum** Thunb., Nova Acta Regiae Soc. Sci. Upsal. 7: 141. 1815 **

*Panicum acroanthum* Steud., Syn. Pl. Glumac. 1: 87. 1854. Lectotype: *Anonymous 2* (L). LT designated by Veldkamp in Blumea 41(1): 188. 1996. “Hrbr. Mus. Lugd. Batav. (sub Milium globosum Thunb. quod non est.). Japonia.”

*Panicum melananthum* F. Muell., Trans. & Proc. Victorian Inst. Advancem. Sci. 1: 47. 1855. Type: Australia: Victoria, Ovens River, “On wet, sandy, or gravelly banks of the Ovens and King River”; February, 1853; *F. von Mueller s.n.*(L).

*Panicum coloratum* sensu F. Muell., Fragm. (Mueller) 8: 192. 1872–1874, non L., 1767

**Syntypes.** Japan; 1808; *Thunberg s.n., 1817, 1818 *(UPS).

**Distribution.** Northeast India [China].

**Habit.** Annual.

**Remarks. **Distribution in India not recorded.

**692. **Panicum brevifolium** L., Sp. Pl. 1: 59. 1753 **

*Isachne tricarinata* Roth, Syst. Veg., ed. 15 bis [Roemer & Schultes] 2: 476. 1817. Type: India; *Heyne s.n.*

*Panicum tricarinatum* (Roth) Steud., Syn. Pl. Glumac. 1: 94. 1854

*Panicum arborescens* L., Sp. Pl. 1: 59. 1753, p.p. Type: “Habitat in India.” Lectotype: *Herb. Hermann 1: 30, No. 43* (BM-000621336). LT designated by Renvoize in Cafferty et al. (ed.), Taxon 49: 253. 2000.

*Panicum biflorum* Lam., Tabl. Encycl. 1: 174. 1791 (as “biflore”). Type: “Lieu nat. l’lsle de France. Tige ram. Panicule mediocre. Le cal. m’a paru univalve.”

*Panicum dubium* Lam., Encycl. (Lamarck) 4: 743. 1798, nom. superfl & illeg. for P. bilforum

*Panicum gladiatum* Wawra, Oesterr. Bot. Z. 12: 170. 1862. Type: “Brasilia; culta in c.r. hort. Schonbr.”

*Panicum ovalifolium* Poir., Encycl. (Lamarck) Suppl. 4: 279. 1816. Type: “Cette plante croit en Guinee.” *Hb. Desvaux in Hb. Lamarck* (P).

*Panicum trichopiptum* Steud., Syn. Pl. Glumac. 1: 85. 1854. Type: Bahia, Senegamb. Guadaloupe.

*Panicum guineense* Desv. ex Poir., Encycl. (Lamarck) Suppl. 4: 279. 1816, nom. nud., pro. syn.

**Type.** “Habitat in India.” Lectotype: *Herb. Linn. No. 80.64* (LINN). LT designated by Clayton & Renvoize in Polhill (ed.), Fl. Trop. E. Africa, Gramineae 3: 496. 1982.

**Distribution.** Andhra Pradesh, Arunachal Pradesh, Assam, Bihar, Chhattisgarh, Karnataka, Kerala, Madhya Pradesh, Meghalaya, Nagaland, Odisha, Sikkim, Tamil Nadu, Tripura [China].

**Habit.** Annual.

**693. **Panicum coloratum** L., Mant. Pl. 30. 1767 **

*Panicum coloratum* L. var. *glaucum* Jauhar, J. Bombay Nat. Hist. Soc. 65:520. 1968

**Type.** “Habitat in Cairi. Forskohl.” Lectotype: Herb. Linn. No. 80.46 (LINN). LT designated by Zuloaga in Görts-van Rijn (ed.), Fl. Guianas, ser. A, 8: 384. 1990.

**Distribution.** Delhi, Rajasthan [China].

**Habit.** Perennial.

**Remarks.** Records for India fide Naithani (1990). Native range not recorded.

**694. **Panicum curviflorum** Hornem., Hort. Bot. Hafn. 116. 1819 var. **curviflorum

*Panicum trypheron* Schult., Mant. 2 (Schultes) 244. 1824. Type: India; *Roxburgh s.n.*

*Panicum roxburghii* Spreng., Syst. Veg. (ed. 16) [Sprengel] 1: 320. 1824 (“1825”). Type: India orientalis

*Panicum tenellum* Roxb., Fl. Ind. (Carey & Wallich ed.) 1: 309. 1820, non Lam., 1791

*Panicum miliaceum* sensu Thwaites, Enum. Pl. Zeyl. (Thwaites). 360. 1864, non L., 1753

**Neotype.** India: Herb. Roxburgh, cultivated in Copenhagen in 1818, from seed obtained from Wallich as Panicum tenellum Roxb. which in turn were collected from plants cultivated in the Calcutta Bot. Gardens (BM). NT designated by Veldkamp in Blumea 34(1): 79. 1989.

**Distribution.** Andhra Pradesh, Assam, Bihar, Chhattisgarh, Delhi, Gujarat, Himachal Pradesh, Jammu and Kashmir, Karnataka, Kerala, Madhya Pradesh, Maharashtra, Meghalaya, Odisha, Punjab, Rajasthan, Tamil Nadu, Tripura, Uttar Pradesh, West Bengal [China, Pakistan, Sri Lanka].

**Habit.** Annual.

**695. **Panicum curviflorum** Horenem var. **suishaense** (Hayata) Veldkamp, Blumea 34(1): 81–82. 1989 **

*Panicum suishaense* Hayata, Icon. Pl. Formosan. 6, Suppl. 98. 1917. Type: Japan: Suisha; August, 1912; *Hayata s.n.* (TI).

*Panicum elegantissimum* Hook. f., Fl. Brit. India (J.D. Hooker). 7(21): 52. 1896. Type: Malay Peninsula: Perak; *Ridley 3116* (K).

*Panicum papuanum* Mez, Bot. Jahrb. Syst. 56 (Beibl. 125): 6. 1921. Type: New Guinea: Waigiou; *Lesson, A° 1825 in Hb. Kunth* (B).

**Distribution.** Bihar [China, Myanmar, Pakistan, Sri Lanka].

**Habit.** Annual.

**696. **Panicum dichotomiflorum** Michx., Fl. Bor.-Amer. 1: 48. 1803 **

**Type.** USA. “Hab. in occidentalibus montium Alleghanis”, Michaux s.n.

**Distribution.** India [China]. Naturalized; possibly native to North America.

**Habit.** Annual.

**Remarks. **Distribution in India not recorded.

**697. **Panicum deccanense** Naik & Patunkar, Reinwardtia 9(4): 405, f. 2. 1980 **

**Type.** India: Nanded, Degloor; September 24, 1974; *Patunkar 2450a* (Marathwada University Herbarium).

**Distribution.** Maharashtra.

**Habit.** Not recorded.

**698. **Panicum fischeri** Bor, Kew Bull. 1956: 257–258. 1956  **

**Type.** India: Madras State, Nilgiris, Kullar, alt. 2500 ft.; October, 1889; *J.S. Gamble 21338* (K).

**Distribution.** Andhra Pradesh, Kerala, Tamil Nadu.

**Habit.** Perennial.

**Remarks. **Records for Kerala and Tamil Nadu fide Naithani (1990).

**699. **Panicum flexuosum** Retz., Observ. Bot. (Retzius) 3: 9. 1783 **

*Panicum psilopodium* Trin. var. *coloratum* Hook. f., Fl. Brit. India (J.D. Hooker). 7(21): 46. 1896. Type: India: Simla and Garhwal, up to 6000 ft., Mt. Abu; *Duthie s.n.*

**Type.** “In Agris Oryzaceis passim obvenientem habui ab amiosiss”; *Koenig s.n.*

**Distribution.** Andhra Pradesh, Himachal Pradesh, Karnataka, Maharashtra, Tamil Nadu, Uttar Pradesh, Uttarakhand [China, Myanmar, Nepal, Pakistan].

**Habit.** Not recorded.

**700. **Panicum garadei** Sundararagh. & Karthik., Bull. Bot. Surv. India 24(1–4): 146. 1983(“1982”) **

**Type.** India: Karnataka, Dharwar; September 17, 1907; *L.D. Garade s.n.* (CAL).

**Distribution.** Karnataka.

**Habit. **Not recorded.

**701. **Panicum gardneri** Thwaites, Enum. Pl. Zeyl. (Thwaites). 359. 1864 **

*Isachne gardneri* (Thwaites) Hook. f., Fl. Brit. India (J.D. Hooker). 7(21): 26. 1896

**Type.** Sri Lanka: Central Province, alt. upto 4000 ft.; *Thwaites Ceylon Plant 894* (K).

**Distribution.** Karnataka, Kerala, Maharashtra, Tamil Nadu [Sri Lanka].

**Habit.** Perennial.

**702. **Panicum hippothrix** K. Schum., Pflanzenw. Ost-Afrikas C: 103. 1895 **

*Isachne obscurans* Woodrow, Gard. Chron. ser. 3, 23: 161. 1898

*Panicum obscurans* (Woodrow) Stapf ex Woodrow, J. Bombay Nat. Hist. Soc. 13: 434. 1901

**Type.** Ghasal, Quellengebiet, Muva; *Holst no. 3177?? (K).*

**Distribution. **Goa, Dadra and Nagar Haveli, Daman and Diu, Maharashtra.

**Habit.** Annual.

**Remarks.** Record for Maharashtra fide Naithani (1990).

**703. **Panicum humidorum** Buch.-Ham. ex Hook. f., Fl. Brit. India (J.D. Hooker). 7(21): 53–54. 1896 **

*Panicum perakense* (Hook. f.) Merr., Philipp. J. Sci., C.11: 52. 1916.

*Panicum humidorum* Buch.-Ham. ex Hook. f. var. *perakense* Hook. f., Fl. Brit. India (J.D. Hooker). 7(21): 54. 1896. Type: Malaysia: Perak; *King’s Collector 2546* (K).

**Lectotype.** India: Assam, Goalpara; *Buchanan-Hamilton in *Numer. List [Wallich] no. 8721 (K). LT designated by Veldkamp in Blumea 41(1): 193. 1996.

**Distribution.** Assam, Kerala, Meghalaya, Odisha. [China].

**Habit.** Perennial.

**704. **Panicum humile** Nees, Syn. Pl. Glumac. (Steudel) 1: 84. 1854 **

*Panicum watense* Mez, Bot. Jahrb. Syst. 34(1): 146–147. 1904. Type: “Senegambien, auf uberschwemmten Flachen bei Wato”; *Leprieur s.n.* (B).

*Panicum austroasiaticum* Ohwi, Acta Phytotax. Geobot. 11(1): 45. 1942 (as “austro-asiaticum”), nom. illeg. superfl.

*Panicum vescum* R.R. Stewart, Brittonia 5(4): 452. 1945, nom. illeg. & superfl.

**Type.** Sri Lanka; *Thwaites Ceylon Plant 3243* (P). B, K, US.

**Distribution.** Andaman and Nicobar Islands, Andhra Pradesh, Assam, Bihar, Goa, Karnataka, Kerala, Maharashtra, Orisssa, Punjab, Sikkim, Uttar Pradesh, West Bengal [China, Myanmar, Pakistan, Sri Lanka].

**Habit.** Not recorded.

**705. **Panicum incisum** Munro ex C.B. Clarke, J. Linn. Soc., Bot. 25: 84. t. 33. 1889 **

**Type. **“Nambre Forest, ..”[India: Assam], “*in Griffith Kew, n. 6505.*” Naga Hills; *C.B. Clarke no. 40799* (K).

**Distribution.** Assam, Nagaland.

**Habit.** Perennial.

**706. **Panicum johnii** S.M. Almeida, J. Bombay Nat. Hist. Soc. 83: 184. f. 3. 1986 **

**Type.** India: Maharashtra, Savantwadi, Sateli; September 5, 1980; *S.M. Almeida 2597* (BLAT).

**Distribution.** Maharashtra.

**Habit.** Not recorded.

**707. **Panicum khasianum** Munro ex Hook. f., Fl. Brit. India (J.D. Hooker). 7(21): 54. 1896 **

**Lectotype.** India: Khasia Hills, in marshes, 4000–6000 ft.; *Griffith 6498* (K). LT designated by Veldkamp in Blumea 41(1): 194. 1996.

**Distribution.** Assam, Meghalaya, Sikkim [Bhutan, China].

**Habit.** Perennial.

**708. **Panicum laevifolium** Hack., Bull. Herb. Boissier 3(8): 378. 1895 **

**Syntypes.** South Africa: Transvaal, Pretoria, Kuduspoort; *Rehmann 4697 *(W). South Africa: Transvaal, Boshveld inter Elandsriver et Klippan; *Rehmann 5123* (W). South Africa: Transvaal, Hogge Veld inter Porter et Trigardsfont; *Rehmann 6614 *(W). South Africa: Transvaal, Donkershoek; *Rehmann 6552* (W).

**Distribution.** India [also in Africa]. Native to South Africa.

**Habit.** Not recorded.

**Remarks.** Distribution in India not recorded.

**709. **Panicum luzonense** J. Presl in C. Presl, Reliq. Haenk. 1(4–5): 308. 1830 **

*Panicum cambogiense* Balansa, J. Bot. (Morot) 4: 142. 1890. Lectotype: Cambodia; *Godefroy 62*. LT designated by Veldkamp in Blumea 41(1): 195. 1996.

*Panicum cruciabile* Chase, J. Arnold Arbor. 20(3): 309. 1939

*Panicum reticulatum* Thwaites ex Trimen, J. Bot. 23: 271. 1885, non Torr., 1852. Type: “Habitat Broders of paddy-fields, Hewessee, Pasdun Korle; August, 1865; *Thwaites s.n.* (Thwaites Ceylon Plant 3890 in Herb. Perad.)

*Panicum caesium* sensu Hook. f., Hooker’s J. Bot. Kew Gard. Misc. 2: 97. 1850, non Nees, 1836

**Type.** Philippine Islands: Luzon; *Haenke s.n.* (PR). ISOTYPE, US.

**Distribution. **Andaman and Nicobar Islands, Assam [Myanmar].

**Habit.** Annual.

**710. **Panicum miliaceum** L., Sp. Pl. 1: 58. 1753 **

*Milium panicum* Mill., Gard. Dict., ed. 8, Milium no. 1. 1768.

*Panicum asperrimum* Fisch., Cat. Jard. Pl. Gorenki 3. 1812. Type: “Cultivated in the garden of the University at Vienna from seed received from Count Razoumovsky of Gorenki [near Moscow]”

*Panicum densepilosum* Steud., Syn. Pl. Glumac. 1: 72. 1853. Type: Japan

*Milium esculentum* Moench, Methodus (Moench) 203. 1794, nom. superfl.

*Panicum milium* Pers., Syn. Pl. (Persoon) 1: 83. 1805, nom. illeg. & superf. for *Panicum miliaceum*

**Type.** “Habitat in India.” Lectotype: *Herb. Linn. No. 80.49* (LINN). LT designated by Sherif & Siddiqi in El-Gadi (ed.), Fl. Libya 145: 282. 1988.

**Distribution.** Andhra Pradesh, Assam, Bihar, Goa, Gujarat, Himachal Pradesh, Jammu and Kashmir, Karnataka, Madhya Pradesh, Maharashtra, Odisha, Rajasthan, Sikkim, Tamil Nadu, Uttar Pradesh, West Bengal [Bhutan, China, Myanmar, Pakistan, Sri Lanka].

**Habit.** Annual.

**Remarks.** Widely cultivated in many parts of the world.

**711. **Panicum nehruense** Jauhar & Joshi, Bull. Bot. Surv. India 8(1): 97, f. A. 1966 **

**Type. **Plant obtained originally from Australia and grown in Jodhpur; *P.P. Jauhar 9* (HCIO).

**Distribution.** Gujarat, Rajasthan [Australia].

**Habit.** Annual.

**Remarks. **Record for Rajasthan fide Naithani (1990).

**712. **Panicum notatum** Retz., Observ. Bot. (Retzius) 4: 18. 1786–1787 **

*Panicum montanum* Roxb., Fl. Ind. (Carey and Wallich ed.) 1: 315. 1820. Type: India: Circar Mountains; *Roxburgh 813* (BM).

**Type.** “In Sumatra lectum dedit nuper nominatus D. Wennerberg.” Indonesia: Sumatra; *Wennerberg *(LD).

**Distribution.** Andhra Pradesh, Bihar, Chhattisgarh, Goa, Karnataka, Kerala, MadhyaPradesh, Maharashtra, Odisha, Rajasthan, Tamil Nadu [China, Myanmar, Nepal].

**Habit. **Perennial.

**713. **Panicum paianum** Naik & Patunkar, Reinwardtia 9(4): 407. 1980 var. **paianum

**Type.** India: Madhya Pradesh, Nanded, Rajgarh; October 26, 1974; *Patunkar 2430a* (Marathwada University Herbarium).

**Distribution.** Maharashtra, Madhya Pradesh.

**Habit.** Annual.

**Remarks.** Record for Maharashtra fide Naithani (1990).

**714. **Panicum paianum** Naik & Patunkar var. **minor** Naik & Patunkar, Reinwardtia 9(4): 409. 1980 **

**Type.** India; *Patunkar 2439a* (Marathwada University Herbarium).

**Distribution.** Maharashtra.

**Habit.** Annual.

**Remarks.** Record for Maharashtra fide Naithani (1990).

**715. **Panicum paludosum** Roxb., Fl. Ind. (Carey & Wallich ed.) 1: 310. 1820 **

*Panicum decompositum* var. *paludosum* (Roxb.) Trimen, Syst. Cat. Fl. Pl. Ceylon 105. 1885

*Panicum proliferum* var. *paludosum* (Roxb.) Stapf, Fl. Cap. 7: 407. 1899

*Panicum repens* var. *paludosum* (Roxb.) Kuntze, Revis. Gen. Pl. 3(3): 363. 1898

*Panicum proliferum* sensu Hook. f., Fl. Brit. India (J.D. Hooker). 7(21): 50. 1896, non Lam., 1797

**Type.** India: Circar Mountains; *Roxburgh 806* (BM). CAL, K.

**Distribution.** Andhra Pradesh, Assam, Bihar, Delhi, Goa, Gujarat, Haryana, Himachal Pradesh, Jammu and Kashmir, Karnataka, Kerala, Madhya Pradesh, Maharashtra, Manipur, Meghalaya, Odisha, Punjab, Rajasthan, Tamil Nadu, Tripura, Uttar Pradesh [China, Nepal, Pakistan, Sri Lanka].

**Habit.** Perennial.

**716. **Panicum phoiniclados** Naik & Patunkar, Reinwardtia 9(4): 403, f. 1. 1980 **

**Type.** Nanded. Gamkgaon; November 17, 1974; *Patunkar 2468a* (Marathwada University Herbarium).

**Distribution.** Madhya Pradesh, Maharashtra.

**Habit.** Perennial.

**Remarks.** Record for Maharashtra fide Naithani (1990).

**717. **Panicum repens** L., Sp. Pl., ed. 2. 1: 87. 1762 **

*Panicum arenarium* Brot., Fl. Lusit. 1: 82. 1804. Type: “Habitat in arenosis subhumidis, occurrit in Algarbiis, ultra Tagum, et Passim ad Mundam in agris Conimbricensibs.”

*Panicum convolutum* P. Beauv. ex Spreng., Syst. Veg. (ed. 16) [Sprengel] 1: 319. 1824 (“1825”). Type: Guinea; *A.M. Palisot de Beauvois Hb. Willdenow 18855 *(B).

*Panicum ischaemoides* Retz., Observ. Bot. (Retzius) 4: 17. 1786–1787. Type: “Vulgatissimum Malabariae in udis ad margines ftagnorum gramen, proceritate reliqua Panica excedens. Cel. Koenig.” India: Malabraiae; *Koenig s.n.* (LD).

*Panicum leiogonum* Delile, Descr. Egypte, Hist. Nat. 3: 51. 1812. Type: Africa: Damietta

**Type.** “Habitat in Hispania? inde missum a Claud. Alstroemer.” Lectotype: *Alstroemer 2a, Herb. Linn. No. 80.47* (LINN). LT designated by Hitchcock & Chase in Contr. U. S. Natl. Herb. 15: 85. 1910.

**Distribution.** Andhra Pradesh, Assam, Bihar, Chhattisgarh, Jharkhand, Karnataka, Kerala, Madhya Pradesh, Maharashtra, Nagaland, Odisha, Punjab, Rajasthan, Sikkim, Tamil Nadu, Tripura, West Bengal [China].

**Habit.** Perennial.

**718. **Panicum sarmentosum** Roxb., Fl. Ind. (Carey & Wallich ed.) 1: 311. 1820 **

*Panicum incomtum* Trin., Gram. Panic. [Trinius] 200. 1826. Type: Philippine Islands: Manila; *Chamisso in Hb. Trinius 0760.02* (LE).

**Type.** Indonesia: Sumatra, brought into Botanic Garden in 1804 by *Dr. Charles Campbell*; *Roxburgh 1778* (HOLOTYPE, BM). ISOTYPES CAL, K.

**Distribution.** Assam, Meghalaya, West Bengal [China, Myanmar].

**Habit.** Perennial.

**719. **Panicum sparsicomum** Nees, Syn. Pl. Glumac. (Steudel) 1: 83. 1854 **

*Agrostis zeylanica* Klein ex Steud., Syn. Pl. Glumac. 1: 83. 1854, nom. nud., pro. syn.

*Cyrtococcum sparsicomum* (Nees) A. Camus, Bull. Mus. Natl. Hist. Nat. 27: 118. 1921

**Type.** Sri Lanka; *Klein s.n., Wight s.n.*

**Distribution.** Tamil Nadu [Sri Lanka].

**Habit.** Annual or perennial.

**720. **Panicum sumatrense** Roth, Syst. Veg., ed. 15 bis [Roemer & Schultes] 2: 434. 1817 **

*Panicum psilopodium* Trin., Gram. Panic. [Trinius] 217. 1826. Lectotype: *Lindley *sub* P. ramosum *Koem (LE). LT designated by Veldkamp in Blumea 41(1): 206. 1996.

*Panicum sumatrense* Roth ssp. *psilopodium* (Trin.) de Wet., J. Agric. Trop. Bot. Appl. 30(2): 162. 1983

*Panicum miliare* Lam., Tabl. Encycl. 1: 173. 1791 (as “miliaire”). Type: “Lieu nat. Inde” India; *Sonnerat s.n.* (P-LAM).

**Type.** Indonesia: Sumatra; *Heyne in Hb. Roth s.n. *(B). LT designated by Veldkamp in Blumea 41(1): 205. 1996.

**Distribution.** Andhra Pradesh, Bihar, Chhattisgarh, Gujarat, Jammu and Kashmir, Karnataka, Kerala, Madhya Pradesh, Maharashtra, Odisha, Rajasthan, Tamil Nadu, Uttar Pradesh, West Bengal [Pakistan, Sri Lanka].

**Habit.** Annual.

**721. **Panicum turgidum** Forssk., Fl. Aegypt.-Arab. 18–19. 1775 **

**Type.** “In desertis Kahirinis Arab. Bockar.” Egypt: Cairo, Kahirini desert; 1761–1762; *Forsskal s.n.* (C).

**Distribution.** Gujarat, Rajasthan [Pakistan].

**Habit.** Perennial.

**722. **Panicum virgatum** L., Sp. Pl. 1: 59. 1753 **

*Milium virgatum* (L.) Lunell, Amer. Midl. Naturalist 4: 212. 1915

*Eatonia purpurascens* Rafin., J. Phys. Chim. Hist. Nat. Arts 89: 104. 1819. Type: USA: New York, Long Island; *Coll. Ukn. s.n.*

*Panicum coloratum* Walter, Fl. Carol. 73. 1788, non L., 1767

*Panicum giganteum* Scheele, Linnaea 22: 340–341. 1849. Type: “In trockenen felsigen Flusebett des Cibolo zwischen San Antonio und Neubraunfels”; *Lindheimer s.n.*

*Panicum glaberrimum* Steud., Syn. Pl. Glumac. 1: 94. 1854. Type: “Cultum ex H. Berol. sem. 1840. sub. Ichnantus glaber. Link. Am. sptr.”

*Panicum kunthii* E. Fourn. ex Hemsl., Biol. Centr.- Amer., Bot. 3: 490. 1885, non Steud., 1841

*Chasea virgata* (L.) Nieuwl., Amer. Midl. Naturalist 2: 64. 1911 [Generic name invalidly published.]

*Panicum pruinosum* Bernh. ex Trin., Gram. Panic. [Trinius] 191. 1826 (as “pruinosi”), nom. nud.

**Type.** “Habitat in Virginia.” Lectotype: *Clayton 578, Herb. Linn. No. 80.61* (LINN). LT designated by Hitchcock in Contr. U. S. Natl. Herb. 12: 118. 1908.

**Distribution.** India [North America, South America]. Native to North America.

**Habit.** Perennial.

**Remarks.** It is unclear if this species is naturalized in India or only cultivated.

***Pseudechinolaena* Stapf, Fl. Trop. Afr. [Oliver et al.] 9: 494. 1919**

**Type. ***Pseudechinolaena polystachya* (Kunth) Stapf (*Echinolaena polystachya* Kunth).

**723. **Pseudechinolaena polystachya** (Kunth) Stapf, Fl. Trop. Afr. [Oliver et al.] 9: 495. 1919 **

*Echinolaena polystachya *Kunth, Nov. Gen. Sp. [H.B.K.] 1: 119 (ed. qto.). 1816. Type: Colombia; *Humboldt & Bonpland s.n. *(P). “Crescit in ripa fluminis Magdalenae inter Tenerife et Zambrano.”

*Echinolaena trinii* Zoll. & Moritz, Syst. Verz. (Zollinger) 102. 1846. Type: “Panicum uncinatum Trin, V.3. non Brown”

*Lappago aliena* Spreng., Neue Entdeck. Pflanzenk. 3: 15. 1822. Type: “Hab. in Brasilia”; *Zeyher s.n.*

*Panicum uncinatum* Raddi, Agrostogr. Bras. [Raddi] 41–42. 1823. Lectoype: Brazil: Rio de Janeiro “Invenitur cum precedenti, in sylvaticis prope Catumby, non procul ab Urbe Rio de Janeiro”; *Raddi s.n.* (PI). LT designated by Baldini & Longhi-Wagner Taxon 55(2): 477. 2006

*Panicum heteranthum* Link, Hort. Berol. [Link] 1: 212. 1827. Type: “Habitat in Brasilia.”

*Panicum nemorosum* sensu Trin., Gram. Panic. [Trinius] 173. 1826, non Sw., 1788

*Panicum glandulosum* Nees ex Trin., Gram. Panic. [Trinius] 174. 1826, nom. nud.

*Panicum polystachyum* (Kunth) K. Schum., Pflanzenw. Ost-Afrikas C: 103. 1895, non A. Rich ex Kunth, 1833, nec J. Presl & C. Presl, 1828.

**Distribution.** Assam, Andhra Pradesh, Arunachal Pradesh, Bihar, Karnataka, Kerala, Manipur, Meghalaya, Mizoram, Nagaland, Sikkim, Tamil Nadu, West Bengal [China, Pakistan].

**Habit.** Annual.

***Pseudoraphis* Griff. ex Pilg., Notizbl. Bot. Gart. Berlin-Dahlem 10(93): 210. 1928**

**Lectotype. ***Pseudoraphis brunoniana* (Wall. & Griff.) Griff. ex Pilg. (*Panicum brunonianum* Wall. & Griff.) LT Designated by Ohwi, Acta Phytotax. Geobot. 10: 272. 1941.

**724. **Pseudoraphis brunoniana** (Wall. & Griff.) Griff. ex Pilg., Notizbl. Bot. Gart. Berlin-Dahlem 10(93): 210. 1928 **

*Panicum brunonianum* Wall. & Griff., J. Asiat. Soc. Bengal 5: 574. 1836. Type: Bangladesh (“Bengal”): Sylhet District, near Goalnuyar; September 28, 1835 (locality uncertain); *W. Griffith 6559* (L). “Hab. In aquis leniter currentibus profundis plagarum Bheels dictarum prope Goalna.” (vide Tropicos).

*Chamaeraphis spinescens* (R. Br.) Poir. var. *brunoniana* (Wall. & Griff.) Hook. f., Fl. Brit. India (J.D. Hooker). 7(21): 62. 1896

*Panicum intermedium* Griff., Not. Pl. Asiat. 3: 29. 1851, nom. invalid.

*Holcus natans* Roxb. ex Hook. f., Fl. Brit. India (J.D. Hooker). 7(21): 62. 1896, nom. nud.

**Distribution.** Bihar, Madhya Pradesh, Manipur, Odisha, Uttarakhand, West Bengal [Bangladesh, China, Myanmar, Sri Lanka].

**Habit.** Perennial.

**725. **Pseudoraphis minuta** (Mez) Pilg., Notizbl. Bot. Gart. Berlin-Dahlem 10(93): 210. 1928 **

*Chamaeraphis minuta* Mez, Notizbl. Bot. Gart. Berlin-Dahlem 7(63): 48. 1917. Syntypes: Vietnam: Bengalia inferior prope Daccar; *C.B. Clarke 17040 *(B). Vietnam: Tonkin, prope Hanoi ad paludum margines; *B. Balansa 1593, 1592, 4779 *(P). Burma; *Kurz s.n.* Cachar; *Keenan s.n.*

*Chamaeraphis gracilis* sensu Hook. f., Fl. Brit. India (J.D. Hooker). 7(21): 62. 1896, non Hack., 1885

**Distribution.** Assam, Bihar, Odisha, West Bengal [Myanmar].

**Habit.** Perennial.

**726. **Pseudoraphis sordida** (Thwaites) S.M. Phillips & S.L. Chen. Novon 13: 469. 2003 **

*Panicum sordidum* Thwaites, Enum. Pl. Zeyl.: 443. 1864. Type: Sri Lanka: Columbo, G. Thwaites C. P. 3857 (K)

*Pseudoraphis spinescens *(R. Br.) Vickery var. *depauperata* (Nees ex Hook.f.) Bor, Grasses Burma, Ceylon, India and Pakistan 354. 1960.

**Distribution.** West Bengal.

**Habit.** Perennial.

**727. **Pseudoraphis squarrosa** (L.f.) Chase, J. Arnold Arbor. 20(3): 313. 1939 **

*Andropogon squarrosus* L. f., Suppl. Pl. 433. 1782 (“1781”) (as “squarrosum”). Type: “Habitat circa Zeylonam natans supra stagna profundiora rarior.” *Koenig s.n.*

*Anatherum squarrosum* (L.f.) P. Beauv., Ess. Agrostogr. 151. 1812

*Echinochloa squarrosa* (L.f.) Roem. & Schult., Syst. Veg., ed. 15 bis [Roemer & Schultes] 2: 479. 1817.

*Orthopogon squarrosus* (L.f.) Spreng., Syst. Veg. (ed. 16) [Sprengel] 1: 307. 1824 (“1825”)

*Chamaeraphis squarrosa* (L.f.) Chase, Contr. U.S. Natl. Herb. 24: 203. 1925

*Panicum spinescens* R. Br., Prodr. Fl. Nov. Holland. 1: 193. 1810. Type: Australia: New South Wales, near Port Jackson; *R. Brown s.n.* (BM).

*Chamaeraphis spinescens* (R. Br.) Poir., Encycl. (Lamarck) Suppl. 2: 189. 1811

*Pseudoraphis spinescens* (R. Br.) Vickery, Proc. Roy. Soc. Queensland 62: 69. 1952 (“1950”)

*Panicum abortivum* R. Br., Prodr. Fl. Nov. Holland. 193. 1810. Type: “Littora Novae Hollandiae intra tropicum.”

*Chamaeraphis abortiva* (R. Br.) Poir., Encycl. (Lamarck) Suppl. 2: 189. 1811

*Orthopogon abortivus* (R. Br.) Spreng., Syst. Veg. (ed. 16) [Sprengel]1: 306. 1824 (“1825”)

*Pseudoraphis abortiva* (R. Br.) Pilg., Notizbl. Bot. Gart. Berlin-Dahlem 10(93): 210. 1928

*Pseudoraphis aspera* Pilg., Notizbl. Bot. Gart. Berlin-Dahlem 10(93): 210. 1928

*Chamaeraphis aspera* Nees, Numer. List. (Wallich). no. 8679. 1849

*Panicum squarrosum* (L.f.) Lam., Encycl. (Lamarck) 4: 733. 1798, non Retz., 1786

*Panicum asperum* J. Koenig, Naturforscher (Halle) 23: 209. 1788, non Lam., 1778. Type: India: Calcutta; January, 1785; *J. Koenig s.n.*

*Chamaeraphis spinosa* P. Beauv. ex Schult., Mant. 2 (Schultes) 253. 1824, nom. nud. pro. syn.

**Distribution.** Andhra Pradesh, Bihar, Chhattisgarh, Goa, Gujarat, Karnataka, Kerala, Madhya Pradesh, Maharashtra, Nagaland, Odisha, Rajasthan, Tamil Nadu, Uttar Pradesh, West Bengal [Sri Lanka].

**Habit.** Perennial.

**Remarks.** It is unclear whether the name for this species should be *Pseudoraphis squarrosa*, based on *Andropogon squarrosus* as noted here, or *P. spinescens*. Bor (1960) suggests that *A. squarrosus* may be a nom. confusum. If so, then the basionym *Panicumspinescens* R. Br. would take priority.

***Sacciolepis* Nash, Man. Fl. N. States (Britton) 89. 1901**

**Type. ***Sacciolepis gibba* (Elliott) Nash (*Panicum gibbum* Elliott).

**728. **Sacciolepis curvata** (L.) Chase, Proc. Biol. Soc. Wash. 21: 8. 1908 **

*Panicum curvatum* L., Syst. Nat., ed. 12. 2: 732. 1767. Type: “Habitat in Suratte.” Lectotype: Herb. Linn. No. 80.60 (LINN). LT designated by Simon in Kew Bull. 27: 390. 1972.

*Panicum coryophorum* Kunth, Révis. Gramin. 1: 387. t. 107. 1831. Type: “Crescit in Madagascaria”; *A. Petit-Thours s.n. *(P).

**Distribution.** Andhra Pradesh, Kerala, Tamil Nadu, West Bengal [Sri Lanka].

**Habit.** Perennial.

**729. **Sacciolepis indica** (L.) Chase, Proc. Biol. Soc. Wash. 21: 8. 1908 var. **indica

*Aira indica* L., Sp. Pl. 1: 63 (“spicata”), Errata (“indicum”). 1753. Type: “Habitat ad villas et plateas Indiae orientalis. Koenig.” Neotype: Sri Lanka. Sabaragamuwa Province, Ratnagsura District; October 22, 1974; *Davidse 7871* (K). NT designated by Renvoize in Cafferty et al. (ed.), Taxon 49: 244. 2000.

*Hymenachne indica* (L.) Buese, Pl. Jungh. 377. 1854

*Panicum angustum* Trin., Sp. Gram. [Trinius] 3: t. 334. 1835. Type: “Figura ad specimen Nepalense”

*Panicum indicum* Mill. var. *angustum* (Trin.) Hook. f., Fl. Brit. India (J.D. Hooker). 7(21): 42. 1896

*Panicum arcuatum* R. Br., Prodr. Fl. Nov. Holland. 189. 1810. Type: “littoral tropical New Holland”; *Banks s.n.*

*Panicum conglomeratum* L., Mant. Pl. Altera 324. 1771. Type: “Habitat [in India.] Sp. Pl. 1: 63 (1753)” Neotype: Sri Lanka: Sabaragamuwa Province, Ratnagsura District; October 22, 1974; *Davidse 7871* (K). Neotype designated by Renvoize in Cafferty et al. (ed.), Taxon 49: 244. 2000.

*Panicum contractum* Wight. & Arn. ex Nees, Linnaea 10 (Litt.-Ber.): 117. 1836. Type: “Hab. in Penins. Ind. ord. Seminiferum exemplum e China retulit co. Meyen.”

*Panicum indicum* Mill. var. *brachiatum* Hook. f. in Trimen, Handb. Fl. Ceylon 5: 148. 1900. Type: Abundant throughout the hotter parts of the Island; Peradeniya; *Thwaites s.n.*

*Panicum microstachyum* Lam., Tabl. Encycl. 1: 170. 1791, nom superfl. & illeg.? Type: “Ex India. Sonner An P. curvatum L.”

*Panicum phalarioides* Roem. & Schult., Syst. Veg., ed. 15 bis [Roemer & Schultes]. 2: 452. 1817 [as “phalaroides” in IPNI and Tropicos]. Type: “Specimen nostrum nominee Phalarides insignitum Lahaye attulit e Java.”

*Sacciolepis spicata* Honda, J. Fac. Sci. Univ. Tokyo, Sect. 3, Bot. 3(1): 261. 1930, invalid

*Panicum myuros* Lam., Tabl. Encycl. 1: 172. 1791 (as “myruos”). Type: “Ex America merid. Comm. A D. Richard.” French Guiana; *D. LeBlond s.n. *(P-LA).

*Panicum indicum* (L.) L., Mant. Pl. Altera 184. 1771, nom. later hom. & illeg., non Mill., 1768

**Distribution.** Andhra Pradesh, Assam, Chhattisgarh, Goa, Jharkhand, Karnataka, Kerala, Madhya Pradesh, Manipur, Maharashtra, Meghalaya, Nagaland, Odisha, Tamil Nadu, Uttar Pradesh, Uttarakhand [Bhutan, China, Myanmar, Nepal].

**Habit.** Annual.

**730. **Sacciolepis indica** (L.) Chase var. **intermedia** Almeida, J. Bombay Nat. Hist. Soc. 83(1): 184–185. 1986 **

**Type.** India: Maharashtra, Savantwadi, Charatha; December 31, 1977; *S.M. Almeida 1393. *

**Distribution.** Maharashtra.

**Habit.** Annual.

**731. **Sacciolepis interrupta** (Willd.) Stapf, Fl. Trop. Afr. [Oliver et al.] 9(4): 757. 1920 **

*Hymenachne interrupta* (Willd.) Buese, Pl. Jungh. 377. 1854

*Panicum interruptum* Willd., Sp. Pl., ed. 4 [Willdenow] 1(1): 341. 1797. Type: “Habitat in Indiae stagnis.”

*Panicum inundatum* Kunth, Révis. Gramin. 1: 34. 1829, nom. illeg. superfl.

*Panicum uliginosum* Roth, Nov. Pl. Sp. 50. 1821. Type: “Heyne ex India Orientali.”

*Panicum indicum* sensu Hack. in Bol. Soc. Brot. 5: 210. 1887, non L., 1771

**Distribution.** Andhra Pradesh, Arunachal Pradesh, Assam, Bihar, Chhattisgarh, Goa, Gujarat, Jharkhand, Karnataka, Kerala, Madhya Pradesh, Maharashtra, Manipur, Meghalaya, Nagaland, Odisha, Rajasthan, Sikkim, Tamil Nadu, Tripura, Uttar Pradesh, West Bengal [Myanmar].

**Habit.** Perennial.

**732. **Sacciolepis myosuroides** (R. Br.) Chase ex E.G. Camus, Fl. Indo-Chine [P.H. Lecomte et al.] 7: 460. 1922 **

*Hymenachne myosuroides* (R. Br.) Balansa, J. Bot. (Morot) 4: 143. 1890

*Panicum myosuroides* R. Br., Prodr. Fl. Nov. Holland. 189. 1810. Type: “Littora novae Hollandiae intra tropicum.”

**Distribution.** Andaman and Nicobar Islands, Andhra Pradesh, Arunachal Pradesh, Assam, Bihar, Chhattisgarh, Himachal Pradesh, Karnataka, Kerala, Madhya Pradesh, Maharashtra, Manipur, Meghalaya, Odisha, Rajasthan, Sikkim, Tamil Nadu, Tripura, Uttar Pradesh, West Bengal [Myanmar].

**Habit. **Annual.

***Setaria* P. Beauv., Ess. Agrostogr. 51, 178. 1812, nom. cons.**

**Type. ***Setaria viridis* (L.) P. Beauv. (*Panicum viride* L.), type cons.

**733. **Setaria barbata** (Lam.) Kunth, Révis. Gramin. 1: 47. 1829 **

*Panicum barbatum* Lam., Tabl. Encycl. 1: 171. 1791. Type: “Ex Insula Franciae.” Mauritius; *P. Commerson s.n.* (P).

*Chaetochloa barbata* (Lam.) Hitchc. & Chase, Contr. U.S. Natl. Herb. 18: 348. 1917

*Panicum costatum* Roxb., Fl. Ind. (Carey & Wallich ed.) 1: 314. 1820. Type: “Introduced into the Botanical Garden from the Mauritius, by Captain Tennant, in 1802.”

*Chamaeraphis costata* (Roxb.) Kuntze, Revis. Gen. Pl. 2: 771. 1891

*Chamaeraphis viatica* Kuntze, Revis. Gen. Pl. 2: 770. 1891

*Panicum basisetum* Steud., Syn. Pl. Glumac. 1: 52. 1853. Type: “Jardin legit in Guinea.”

*Setaria basiseta* (Steud.) T. Durand & Schinz, Consp. Fl. Afric. [T.A. Durand & H. Schinz]. 5: 772. 1894

*Panicum rarisetum* Steud., Syn. Pl. Glumac. 1: 52. 1853. Type: “Ins. Borbonia et var. minor Ins. Maurit. Siber hrbr. Mixt. Nr. 144.”

*Panicum rhachitrichum* Hochst., Flora 27: 254. 1844. Type: “Habitat agros in Nubiae provincia Cordofan.” Sudan; Kotschyi, in pll. exsicc. Un. it. ex Kotschyi itin. *Nubico s.n.*

*Setaria rachitricha* (Hochst.) Rendle, Cat. Afr. Pl. 2(1): 188. 1899. Type: Sudan. Nubia, Prov. Cordofan, C.G.T. Kotschy s.n. (isotypes, BR!, M!, P!, TUB)

*Setaria costata* Stapf, Bull. Agric. Congo Belge 10: 250. 1919, nom. nud.

*Panicum flavescens* sensu Hook. f., Fl. Brit. India (J.D. Hooker). 7(21): 56. 1896, non Sw., 1788

*Panicum viaticum* Salzm. ex Doell, Fl. Bras. (Martius) 2(2): 155. 1877, non Griff. (1851), nom. superfl. & illeg.

**Distribution.** Andaman and Nicobar Islands, Assam, Bihar, Jammu and Kashmir, Karnataka, Kerala, Madhya Pradesh, Meghalaya, Rajasthan, Tamil Nadu, West Bengal [Myanmar, Sri Lanka].

**Habit.** Perennial.

**734. **Setaria flavida** (Retz.) Veldkamp, Blumea 39(1–2): 376. 1994 **

*Panicum flavidum* Retz., Observ. Bot. (Retzius) 4: 15. 1786–1787. Type: “E Zeylona misit honor Koenig.” Sri Lanka; *Koenig s.n.* (LD).

*Paspalidium flavidum* (Retz.) A. Camus, Fl. Indo-Chine [P.H. Lecomte et al.] 7: 419. 1922

*Panicum granulare* Lam., Tabl. Encycl. 1: 170. 1791. Type: “Ex ins. Francis”; *Commerson s.n.*

*Panicum distans* Trinius, Sp. Gram. 2(15): tab. 172. 1829

*Paspalidium flavidum* (Retz.) A. Camus var. *distans *(Trin.) Hook. f., Fl. Brit. India 7: 29. 1896

*Paspalidium distans *(Trin.) Hughes, Bull. Misc. Inform. Kew 1923: 317. 1923

*Setaria distans *(Trin.) Veldkamp, Blumea 39: 376. 1994

*Panicum floridum* Royle, Ill. Bot. Himal. Mts. 1: 420. 1840, nom. nud,

**Distribution.** Andaman and Nicobar Islands, Andhra Pradesh, Assam, Bihar, Dadra and Nagar Haveli, Delhi, Goa, Gujarat, Jammu and Kashmir, Jharkhand, Karnataka, Kerala, Madhya Pradesh, Maharashtra, Odisha, Rajasthan, Tamil Nadu, Tripura, Uttar Pradesh, Uttarakhand, West Bengal [Bhutan, China, Sri Lanka].

**Habit. **Perennial.

**735. **Setaria forbesiana** (Nees) Hook. f., Fl. Brit. India (J.D. Hooker). 7(21): 81. 1896 **

*Panicum forbesianum* Nees, Syn. Pl. Glumac. (Steudel) 1: 98. 1854. Type: Nepal: up to 5000 ft.; *Royle 31*

*Setaria dubia* B.J. Keng & Y.K. Ma, Acta Bot. Yunnan. 2 (4): 421. 1980

**Distribution.** Bihar, Maharashtra, Meghalaya, Nagaland, Odisha. [Bhutan, China, Myanmar, Nepal].

**Habit.** Perennial.

**736. **Setaria geminata** (Forssk.) Veldkamp, Blumea 39(1–2): 377. 1994 **

*Panicum geminatum* Forssk., Fl. Aegypt.-Arab. 18. 1775. Type: “Rofettae in pratis ad littoral Nili.” Egypt: Rashid; November 2–6, 1761; *Forrskal s.n.* (C).

*Paspalidium geminatum* (Forssk.) Stapf, Fl. Trop. Afr. [Oliver et al.] 9: 583. 1920

*Digitaria affinis* Roem. & Schult., Syst. Veg., ed. 15 bis [Roemer & Schultes] 2: 470. 1817. Type: “In Santa Fe de Bogota Ab amiciss. Zea nobiscum communicate.”

*Panicum affine* (Roem. & Schult.) Nees, Fl. Bras. Enum. Pl. 2(1): 113. 1829

*Paspalum appressum* Lam., Tabl. Encycl. 1: 176. 1791. Syntypes: Isle de France (Mauritius), *Commerson s.n. *(syntype, P-LA, isosyntype, US-2941981, fragment ex P-LA).

*Digitaria appressa* (Lam.) Pers., Syn. Pl. (Persoon) 1: 85. 1805

*Panicum appressum* (Lam.) Döll, Fl. Bras. (Martius) 2(2): 184. 1877, non Forssk., 1775

*Panicum beckmanniiforme* J. C. Mikan ex Trin., Neue Entdeck. Pflanzenk. 2: 83. 1821 (as “beckmanniaeforme”). Type: “Hab. in Brasilia.” (LE).

*Panicum briziforme* J. Presl in C.B. Presl, Reliq. Haenk. 1(4–5): 302. 1830 (as “brizaeforme”). Type: “Habitat in Luzonia.” Lectotype: Philippines: Luzon; *Haenke s.n.* (MO, US-80492 (fragm.)). LT designated by Veldkamp in Blumea 39: 377. 1994.

*Panicum fluitans* Retz., Observ. Bot. (Retzius) 3: 8. 1783. Type: “Misit Claris Koenig.” India; *Koenig s.n. in Herb. Retzius* (LD). holotype, LD neg. 7008 K; isotypes, BM, K, fragment).

*Panicum paspaloides* Pers., Syn. Pl. (Persoon) 1: 81. 1805. Type: “Hab. in India.”

*Panicum truncatum* Trin., Gram. Panic. [Trinius] 130. 1826. Syntypes: India orientalis; *Lindley s.n.* Egypt; *Sieber 28*. Brazil; *Nees ab Esenbeck s.n.*

**Distribution. **Andhra Pradesh, Bihar, Delhi, Goa, Gujarat, Karnataka, Kerala, Madhya Pradesh, Maharashtra, Odisha, Rajasthan, Tamil Nadu, Uttar Pradesh [Pakistan].

**Habit.** Perennial.

**737. **Setaria homonyma** (Steud.) Chiov., Nuovo Giorn. Bot. Ital. n. s. 26: 78. 1919 var. **homonyma

*Panicum homonymum* Steud., Syn. Pl. Glumac. 1: 48. 1853. Type: “herb. Royle nr. 47” Nepal; *Royle 47*(P). holotype, P; isotype, K!, photo SI!)

*Panicum chamaeraphis* Nees ex A. Braun., Index Sem. (Berlin) 1855 App. p. 20, non Trin., 1835

*Panicum thollonii* Franchet, Contr. Fl. Congo Franç.: 43. 1895

*Setaria thollonii* (Franchet) Stapf, Bull. Misc. Inform. Kew 1927: 267. 1927. Lectotype: Democratic Republic of the Congo. “Congo Français: dans les sables de Brazzaville”, 1887, *M. Thollon 826* (P!, photo SI!; duplicates, K!, photo SI!)

*Panicum bongaense *Pilger, Bot. Jahrb. Syst. 33 : 44. 1902

*Setaria bongaensis *(Pilger) Mez, Wiss. Erg. Zweit. Deut. Zentr.-Afr. Exped., Bot. 2: 20. 1922. Lectotype: Democratic Republic of the Congo. Bonga, Jul 1899, *A. Schlechter 12647 *(BR!; isolectotypes, G!, L!, US-1445616).

*Setaria aequalis *Stapf, Bull. Misc. Inform. Kew 1927: 267. 1927. Type: Tanzania. Kilimanjaro: Marangu, 1500 m, Sep 1893, *G. Volkens 914* (holotype, K!, photo SI!; isotype, E)

*Setaria kialaensis *Vanderyst, Bull. Agric. Congo Belge 16: 682. 1925. Lectotype (designated here): Democratic Republic of the Congo. Boko-Nzadi, 1916, *H. Vanderyst 6105* (BR!, photo SI!; duplicate BR, photo SI!)

*Setaria lancea *Stapf ex Massey, Sudan Grasses: 34. 1926. Type: Sudan. Mongalla, 7 Apr 1907, *Brown 1114* (K!).

*Setaria microprolepis *Stapf, Fl. Trop. Afr. 9: 849. 1930. Type: Angola. Cungulungulo, *F. Welwitsch 7176* (BM, K!, fragment).

**Distribution.** Bihar, Chhattisgarh, Himachal Pradesh, Karnataka, Kerala, Madhya Pradesh, Maharashtra, Rajasthan, Tamil Nadu, Uttar Pradesh, Uttarakhand, West Bengal [Nepal].

**Habit.** Annual.

**738. **Setaria homonyma** (Steud.) Chiov. var. **depauperata** Babu, Herb. Fl. Dehra Dun 642. 1977 **

**Type.** India: Uttarakhand, Dehra Dun, Robber’s cave; *C.R. Babu 33485* (BSD).

**Distribution.** Uttar Pradesh, Uttarakhand.

**Habit.** Annual.

**Remarks. **Record for Uttar Pradesh fide Naithani (1990).

**739. **Setaria intermedia** Roem. & Schult., Syst. Veg., ed. 15 bis [Roemer & Schultes] 2: 489. 1817 **

*Panicum intermedium* (Roem. & Schult.) Roth, Nov. Pl. Sp. 47–48. 1821, non Vahl, 1813

*Panicum tomentosum* Roxb., Fl. Ind. (Carey and Wallich ed.) 1: 303. 1820. Type: “This is a delicate, rare species, found growing in tufts, or dry pasture ground over various parts of India.” Type: India. *Herb. Roxburgh s.n.* (holotype, BM!, isotypes, CAL!, K, Icon. Ined. 789!; K, Icon. Ined. 787!).

*Setaria tomentosa* (Roxb.) Kunth, Révis. Gramin. 1: 47. 1829

**Type.** “In India orientali; *B. Heyne s.n.*”

**Distribution.** Andhra Pradesh, Bihar, Chhattisgarh, Himachal Pradesh, Jharkhand, Karnataka, Kerala, Madhya Pradesh, Maharashtra, Odisha, Punjab, Rajasthan, Tamil Nadu, Uttar Pradesh, Uttarakhand, West Bengal [Bhutan, Myanmar, Pakistan, Sri Lanka].

**Habit.** Annual.

**740. **Setaria italica** (L.) P. Beauv., Ess. Agrostogr. 51, 170, 178. 1812 **

*Panicum italicum* L., Sp. Pl. 1: 56. 1753. Type: “Habitat in Indiis.” Lectotype: Herb. A. van Royen No. 912.356-242 (L). LT designated by Veldkamp in Cafferty et al. (ed.), Taxon 49: 253. 2000.

*Chaetochloa italica* (L.) Scribn., Bull. Div. Agrostol. U.S.D.A. 4: 39. 1897

*Chamaeraphis italica* (L.) Kuntze, Revis. Gen. Pl. 2: 767. 1891

*Ixophorus italicus* (L.) Nash, Bull. Torrey Bot. Club 22: 423. 1895

*Pennisetum italicum* (L.) R. Br., Prodr. Fl. Nov. Holland. 195. 1810

*Panicum germanicum* Mill., Gard. Dict., ed. 8. Panicum no. 1. 1768. Type: German Panic C.B. P. 27

*Chaetochloa germanica* (Mill.) Smyth, Trans. Kansas Acad. Sci. 25: 89. 1913

*Pennisetum germanicum* (Mill.) Baumg., Enum. Stirp. Transsilv. 3: 277. 1816

*Setaria germanica* (Mill.) P. Beauv., Ess. Agrostogr. 51. t. 13. 169, 178. 1812

*Panicum italicum* L. var. *californicum* Körn. & H. Werner, Handb. Getreidebaus 1: 273. 1885. Type: “Als Kalifornische Kolbenhirse.”

*Setaria californica* Kellogg, Proc. Calif. Acad. Sci. ser. 2, 1: 26. 1873. Type: USA: from the head Valley of the Sacramento River; *Dosh s.n.*

*Setaria italica* (L.) P. Beauv. var. *germanica* (Mill.) Schrad., Linnaea 12: 430. 1838

*Setaria italica* (L.) P. Beauv. subvar. *condensa* F.T. Hubb., Amer. J. Bot. 2: 195. 1915. Type: South Asia: Assam, Hathegaon, May 1902, *Chatterjee s.n.* (holotype, GH!).

*Setaria italica* (L.) P. Beauv. var. *purpureoseta* F.T. Hubb., Amer. J. Bot. 2: 194. 1915. Type: India. Almona, Kumaon, elevation above the sea 5500 feet, *Strachey & Winterbottom 3* (holotype, GH!).

*Setaria italica *(L.) P. Beauv. fo. *brachychaeta* F.T. Hubb., Amer. J. Bot. 2: 192. 1915. Type: India. Mont. Khasia,. *Hooker & Thompson s.n.* (holotype, GH!).

*Setaria italica* (L.) P. Beauv. var. *bruneoseta* F.T. Hubb., Amer. J. Bot. 2 192. 1915. Type: Philippines, Mendez Nuñez, Prov. Of Cavite, Luzon, Aug. 1906, *Bureau of Science 1344(L. Mangubat)* (holotype, GH!).

*Panicum comosum* Steud., Syn. Pl. Glumac. 1: 53. 1853

*Setaria comosa* (Steud.) Miq., Fl. Ned. Ind. 3: 468. 1857. Type: *Cuming 489* (holotype, P!; isotype, K!).

*Setaria itieri* Delile, Ind. Sem. Hort. Monap. Sci. Nat., ser. 3,12: 366. 1849. Type: Egypt. “Setaria italica major. Ex aegypto semina secum attulit et communicavit clarissimus Itier, qui uborrimum opus scripsit “Voyage in la Chine,” 2 vol. in -8 Paris, 1848.”

**Distribution.** Andhra Pradesh, Bihar, Goa, Karnataka, Kerala, Himachal Pradesh, Jharkhand, Karnataka, Madhya Pradesh, Maharashtra, Manipur, Odisha, Rajasthan, Tamil Nadu, Tripura, Uttar Pradesh, Uttarakhand [China].

**Habit.** Annual.

**741. **Setaria palmifolia** (J. Koenig) Stapf, J. Linn. Soc., Bot. 42: 186. 1914 **

*Panicum palmifolium* J. Koenig, Naturforscher (Halle) 23: 208. 1788. Type: Thailand; *J. Koenig s.n.* (US-978043 (fragm. ex FI)). (as” palmaefolium”) holotype, K!; isotypes, C?, FI, US-978043! fragment ex FI).

*Chamaeraphis palmifolia* (J. Koenig) Kuntze, Revis. Gen. Pl. 2: 771. 1891*.*

*Panicum palmifolium *Willd. ex Poir., Encycl. Suppl. 4: 282. 1816, non J. Koenig (1788). Type: “Le lieu natal de cette plante n’est pas connu. (V.S. in herb. Desfont.)”.

*Chaetochloa palmifolia* (Willd. ex Poir.) Hitchc. & Chase, Contr. U. S. Natl. Herb. 18: 348. 1917

*Panicum plicatum *Willd., Enum. Pl.: 1033. 1809, non Lam. (1791).

*Panicum neurodes* Schult., Mant. 2 (Schultes) 228. 1824. Type: Nepal: *Buchanan s.n*.

*Panicum neurodes* Schult. var. *roxburghianum* A. Braun, Index Sem. (Berol.), App.: 20. 1855. Type: ‘In Nepalia (Wallich No. 8702 in herb. Nees ab E.), prope Courtallum (No. 1014) et in Bengalia prope Sillet (No. 8702 in herb. Kunth).’

*Panicum sulcatum *Aubl. var. *roxburghianum* (A. Braun) A. Braun ex K. Schuman & Lauterb., Fl. Schutzgeb. Südsee: 178. 1900

*Chamaeraphis neurodes* (Schult.) Kuntze, Rev. Gen. Pl. 2: 771. 1891.

*Panicum plicatum* Willd., Enum. Pl. (Willdenow) 1033. 1809, non Lam., 1791. Type: “Habitat in India orientali.”

*Panicum nervosum* Roxb., Fl. Ind. (Carey and Wallich ed.) 1: 314. 1820, non Lam., 1791. Type: “From Nepal Dr. Buchanan sent the seed to the Botanical Garden, where the plants blossomed in October, just one year from the time the seed was sown.”

*Panicum lene* Steud., Syn. Pl. Glumac. 1: 54. 1853. Type: Java: *Zollinger 815*.

*Setaria lenis* (Steud.) Miq., Fl. Ned. Ind. 3: 468. 1857

*Panicum neurodes* Schult. var. *lene* (Steud.) A. Braun, Index Sem. (Berol.), App.: 20. 1855

*Chamaeraphissetosa* (Sw.) Kuntze var. *lenis* (Steud.) Kuntze, Rev. Gen. Pl. 2: 769. 1891. Type: Java. Tjikoja, *Zollinger 815 *(holotype, P; isotype, G).

*Panicum sulcatum* Aubl., Hist. Pl. Guiane 1: 50. 1775. Type: “Croît su bord des rivieres.” *Drawing by Plumier* (P).

*Chaetochloa sulcata* (Aubl.) Hitchc., Contr. U. S. Natl. Herb. 17: 260. 1913

*Chamaeraphis sulcata* (Aubl.) Kuntze, Revis. Gen. Pl. 2: 770. 1891

*Setaria sulcata* (Aubl.) A. Camus, Bull. Mus. Hist. Nat. (Paris) 30: 108. 1924, non Raddi, 1823

*Chaetochloa palmifolia *(Kuntze) Hitchcock & Chase, Contr. U.S. Natl. Herb. 18: 348, nom. superfl. 1917.

*Panicum nepalense* Spreng., Syst. Veg., ed. 16 [Sprengel] 1: 321. 1824 (“1825”), nom. superfl.

*Chamaeraphis nepalensis* Kuntze, Rev. Gen. Pl. 2: 771. 1891, nom. superfl.

**Distribution. **Andhra Pradesh, Arunachal Pradesh, Assam, Bihar, Himachal Pradesh, Karnataka, Kerala, Maharashtra, Manipur, Meghalaya, Mizoram, Nagaland, Odisha, Punjab, Sikkim, Tamil Nadu, Tripura, Uttar Pradesh, West Bengal [Bhutan, China, Pakistan, Sri Lanka].

**Habit.** Perennial.

**Remarks. **Taxonomic editor Zuloaga notes that citations of *Setaria paniculifera* (Steud.) E. Fourn. ex Hemsley from India are erroneous, because that species is not present in the Old World. Material listed under this name should correspond to *S. palmifolia* (J. Koenig) Stapf. See also Morrone et al. (2014). Record for Nagaland fide Naithani (1990).

**742. **Setaria parviflora** (Poir.) Kerguélen, Lejeunia, n.s. 120: 161. 1987 **

*Cenchrus parviflorus* Poir., Encycl. 6: 52. 1804

*Pennisetum parviflorum* (Poir.) Trin., Gram. Panic.: 65. 1826

*Chaetochloa corrugata *var. *parviflora* (Poir.) Scribn. & Merr., U.S.D.A. Div. Agrostol. Rep. Agrostol. 21: 24, t. 12. 1900

*Chaetochloa parviflora* (Poir.) Scribn., Field Columbian Mus. Publ. 2: 26. 1900

*Pennisetum indicum *subvar. *parviflora *(Poir.) Leeke, Z. Naturwiss. 79: 19. 1907. Type: Puerto Rico. Without locality, *without collector* (holotype, P-LA, photo US-3049717!).

*Setaria geniculata* P. Beauv., Ess. Agrostogr. 51, 169, 178. 1812

*Panicum geniculatum *Lam., Encycl. 4: 727 [737]. 1798, non *Setaria *P. Beauv. 1812. Type: Leeward Islands: Guadeloupe (type not located).

*Pennisetum geniculatum* (Lam.) Jacq., Ecl. Gram. Rar. 2: 37. t. 26. 1820

*Setaria glauca* (L.) P. Beauv. fo. *ictura *Buse in Miq., Pl. Jungh. 3: 29 (preprint: Feb 1854); 369 (Aug 1854)

*Setaria pallide-fusca* (Schumach.) Stapf & C.E. Hubb. var. *ictura* Jansen, Reinwardtia 2: 340. 1953. Lectotype: *Junghuhn s.n.* (designated by Veldkamp in sched., L-908.94-1139!).

*Setaria glauca* (L.) P. Beauv. ssp. *subtessellata* Buse in Miq., Pl. Jungh. 3: 29 (preprint: Feb 1854); 369 (Aug 1854). Lectotype: Indonesia. Without locality, *Junghuhn s.n.* (designated by Veldkamp, Blumea 39: 380.1994, L-903.342-138!).

*Setaria fieldingii *Müll. Hal, Bot. Zeitung (Berlin) 19: 323. 1861. Type: *J.F. Fielding s.n.* (isotype, SING!).

*Setaria laeta* de Wit, Bull. Jard. Bot. Buitenzorg, ser. 3, 17: 64. 1941. Type: South Africa. Cape, Amanzi, Jan 1939, *J.M. Hector-de Wit s.n. in Herb H.C.D. de Wit 601 *(PRE, photo SI, L, photo SI).

*Setaria roemeri *Jansen, Reinwardtia 2: 340, f. 18. 1953. Type: Indonesia. New Guinea, without locality, *L.S.A.M. von Römer 611 *(L).

*Setaria montana *Reeder, J. Arn. Arbor. 29: 304, t. 3. 1948. Type: Indonesia. New Guinea, 18 km NE of Lake Habbema, Bele River, 2000 m, very abundant on old garden lands, habit ascending, Nov 1938, *L.J. Brass 11488* (holotype, A; isotype, US-1761769!).

*Setaria pallide-fusca* (Schumach.) Stapf & C.E. Hubb. var. *hirtipes* Ohwi, Acta Phytotax. Geobot. 23: 109. 1968. Type: Thailand. Lamphun: en route from Ban Khun Tan to Doi Khun Tan, ca. 700 m alt., 4 Sept. 1967, *Tagawa, Iwatsuki, Koyama, Fukuoka, Nalampoon & Chintayungkun 9188* (holotype, KYO; isotype, A).

*Panicum berteronianum* Steud., Syn. Pl. Glumac. 1: 50. 1854

*Panicum flavum* Nees, Fl. Bras. Enum. Pl. 2(1): 238. 1829. Type: “Campis graminosis provinciae Piauhiensis, tum in campis ad Joazeiro provinciae Pernambucensis et Bahiensis.” Lectotype: Brazil: Bahia; *K.F.P. von Martius s.n. *(M). LT designated by Pensiero in Darwiniana 37: 89. 1999.

*Panicum imberbe* Poir., Encycl. (Lamarck) Suppl. 4: 272. 1816. Type: “In America septentrionali & Brasilia.”

*Setaria imberbis* (Poir.) Roem. & Schult., Syst. Veg., ed. 15 bis [Roemer & Schultes]. 2: 891. 1817

**Distribution.** Rajasthan, Uttar Pradesh, Uttarakhand [also in North America, South America]. Naturalized.

**Habit.** Perennial.

**Remarks.** Probably native to the New World, but possibly Africa; see Kellogg et al. (2009). Widespread and weedy.

**743. **Setaria plicata** (Lam.) T. Cooke, Fl. Bombay 2: 919. 1908 **

*Panicum plicatum* Lam., Tabl. Encycl. 1: 171. no. 892. 1791. Type: “Cette espece est cultivee au Jardin du Museum on la dit originaire de I’lsle-de-france; d’autres pensent qu’elle a été envoyée de saint Domingue.” Cultivated in Paris, origin uncertain; *Anonymous s.n.* (P). Type: *Anon. *in Herb. Lamarck (‘cult. in horto Reg. parisiensis’) (holotype, P, IDC microfiche 6207, fiche 689/19; isotype, B, *Herb. Willdenow 18734/3*, IDC microfiche 7440; US, fragm.).

*Panicum excurrens* Trin., Sp. Gram. [Trinius] 1: t. 89. 1827. Type: Nepal. Without locality, *Wallich s.n. *(holotype, LE; isotype, G, W).

*Setaria excurrens *(Trin.) Miq., Ann. Mus. Bot. Lugduno-Batavum 2: 275. 1867.

*Setaria mauritiana* Spreng., Syst. Veg., ed. 16 [Sprengel] 1: 305. 1824 (“1825”). Type: Mauritius. Without locality, *Bory de Saint-Vincent s.n.* (holotype, B-W-18814!).

*Panicum neurodes* Schult. var. *blepharoneuron* A. Braun, App. Gen. & Sp. Nov. Hort. Reg. Berol. 20. 1856 (“1855”). Type: India; Numer. List [Wallich] no. 8703(B). holotype, B; isotypes, K, L!).

*Setaria palmifolia* Stapf var. *blepharoneuron* (A. Braun) Veldkamp, Floribunda 1(6): 22. 1988

**Distribution.** Andhra Pradesh, Arunachal Pradesh, Assam, Bihar, Chhattisgarh, Jharkhand, Karnataka, Kerala, Madhya Pradesh, Maharashtra, Meghalaya, Odisha, Tamil Nadu, Tripura, Uttar Pradesh, Uttarakhand, West Bengal [China, Nepal, Sri Lanka].

**Habit. **Perennial.

**744. **Setaria pumila** (Poir.) Roem. & Schult., Syst. Veg., ed. 15 bis [Roemer & Schultes]. 2: 891. 1817 **

*Panicum pumilum* Poir., Encycl. (Lamarck) Suppl. 4: 273. 1816. Type: “J’ignore le lieu natal de cette plante. (V.s. in herb. Desfont).” locality unknown but probably France or North Africa; *Desfontaines s.n.* (FI, P).

*Panicum pallide-fuscum* Schumach., Beskr. Guin. Pl. 78. 1827. Type: Ghana; *Thonning s.n.*

*Setaria glauca* var. *pallide-fusca* (Schumacher) T. Koyama, J. Jap. Bot. 37: 237. 1962

*Setaria pumila *var. *pallide-fusca *(Schumacher) B.K. Simon, Austrobaileya 2: 22. 1984. Type: Ghana. Withouth locality, *P. Thonning s.n.* (C, photo BM; isotype and drawing, K!).

*Setaria pallide-fusca* (Schumach.) Stapf & C.E. Hubb., Bull. Misc. Inform. Kew 1930: 259. 1930

*Setaria glauca* sensu Alston in Trimen, Handb. Fl. Ceylon 6(Suppl.): 326. 1931, non (L.) P. Beauv., 1812

*Panicumrubiginosum* Steud., Syn. Pl. Glumac. 1: 50. 1853

*Setaria rubiginosa* (Steud.) Miq., Fl. Ned. Ind. 3: 467. 1857

*Setaria aurea* var. *rubiginosa* (Steud.) Peter, Repert. Spec. Nov. Regni Veg. Beih. 40: 233. 1931. Type: Philippines. Without locality, *H. Cuming 551* (holotype, P; isotypes, G, K, L, MO).

**Distribution.** Andhra Pradesh, Arunachal Pradesh, Assam, Bihar, Gujarat, Madhya Pradesh, Maharashtra, Manipur, Meghalaya, Mizoram, Nagaland, Odisha, Rajasthan, Tamil Nadu, Tripura, Uttar Pradesh, West Bengal [Burma, China, Sri Lanka].

**Habit.** Annual.

**745. **Setaria punctata** (Burm. f.) Veldkamp, Blumea 39(1–2): 381. 1994 **

*Panicum punctatum* Burm. f., Fl. Ind. (N. L. Burman) 26. 1768. Lectotype: India; *Plukenet s.n.* (BM). LT designated by Clayton & Renvoize in Polhill (ed.), Fl. Trop. E. Africa, Gramineae 3: 672. 1982.

*Paspalidium punctatum* (Burm. f.) E.G. Camus & A. Camus, Fl. Indo-Chine [P.H. Lecomte et al.] 7: 419. 1922

*Panicum mucronatum* Roth, Syst. Veg., ed. 15 bis [Roemer & Schultes] 2: 425. 1817. Type: India; *Benj. Heyne s.n.* (B). “Roth Nov. Plant Spec. Mss” cited.

*Paspalum aquaticum* Masamune & Syozi, Trans. Nat. Hist. Soc. Taiwan 34: 303. 1944. Type: Taiwan, *without collector s.n.* (holotype, TAI).

**Distribution.** Andhra Pradesh, Assam, Bihar, Chhattisgarh, Gujarat, Jharkhand, Jammu and Kashmir, Karnataka, Kerala, Madhya Pradesh, Maharashtra, Meghalaya, Odisha, Punjab, Rajasthan, Tamil Nadu, Tripura, Uttar Pradesh, West Bengal [Bangladesh, China, Myanmar].

**Habit.** Perennial.

**746. **Setaria sphacelata** (Schumach.) Stapf & C.E. Hubb in D. Prain, Fl. Trop. Afr. 9: 795. 1930 **

*Panicum sphacelatum* Schumach., Beskr. Guin. Pl. 78–79. 1827. Type: Ghana; *Thonning s.n. *(C, K, photo SI).

*Chaetochloa aurea* (Schumach.) Hitchc., Proc. Biol. Soc. Wash. 29: 128. 1916

*Panicum chrysanthum* Steud., Syn. Pl. Glumac. 1: 50. 1853. Type: “Hochst hrbr. Un. It. 409.”

*Panicum rudimentosum* Steud. Syn. Pl. Glumac. 1: 51. 1853. Type: “Leprieur legit in Senegalia.”

*Setaria aurea* Hochst. ex A.Braun, Flora 24: 276. 1841. Type: Cultivated at Karlruhe from seed collected by Schimper in Ethiopia; *Schimper 409* (MO-IT).

**Distribution.** Tamil Nadu [Sri Lanka].

**Habit.** Perennial.

**747. **Setaria sulcata** Raddi, Agrostogr. Bras. [Raddi] 50. 1823 **

*Panicum poiretianum* Schult., Mant. 2 (Schultes) 229. 1824

*Setaria poiretiana* (Schult.) Kunth, Révis. Gramin. 1: 47. 1829

*Chaetochloa poiretiana* (Schult.) Hitchc., Contr. U. S. Natl. Herb. 22: 159. 1920

*Panicum sulcatum* Bertol., Opusc. Sci. 14: 230. 1820, non Aubl., 1775. Type: “Habitat in Brasilia.” Brazil: Rio de Janeiro; Raddi 17 (FI) (FI, K, US fragment & photo ex FI).

*Panicum elongatum* Poir., Encycl. (Lamarck) Suppl. 4: 278. 1816, non Salisb., 1796, nec Pursh 1813. Type: “Cette plante croit au Bresil”; *Desfontaines s.n.* (FI, K, P, fragment, US-80632).

*Panicum phyllomacrum *Steud., Syn. Pl. Glumac. 1: 53. 1853. Type: Gabon. Without locality, *E. Jardin s.n.* (holotype, P, photo SI).

*Setaria phyllomacra* (Steud.) T. Durand & Schinz, Consp. Fl. Afr. 5: 773. 1895.

*Panicum prolisetum *Steud., Syn. Pl. Glumac. 1: 52. 1853. Type: São Tomé and Príncipe. Without locality and collector (type, P, fragment and photo SI).

*Setaria proliseta* (Steud.) T. Durand & Schinz, Consp. Fl. Afr. 5: 774. 1894.

*Panicum megaphyllum* Steud., Syn. Pl. Glumac. 1: 53. 1853. Type: Gabon. Without locality, 1846,* E. Jardin 26* (holotype, P, photo SI; isotype, G, photo SI, US-80795, fragment ex P).

*Setaria megaphylla* (Steud.) T. Durand & Schinz, Consp. Fl. Afr. 5: 773. 1894.

*Setaria macrophylla *Andersson, Naturw. Reise Mossambique 2: 550. 1854. Type: Mozambique. “An den lichten Randern hoher Walder, in Marschboden... in der Nahe von Boror, 18’ S.B.”, *A. Peter 1842-48 *(holotype, B, destroyed).

*Panicum plicatile *Hochst., Flora 38: 198. 1855. Type: Ethiopia. Without locality, 1853, *G.W. Schimper in Herb. Buchinger 1456* (BR, photo SI, G, photo SI, K, photo SI, P, photo SI, STR).

*Setaria plicatilis* (Hochst.) Hack. ex Engler, Abh. Konigl. Akad. Wiss. Berlin 2: 121. 1891.

*Setaria oligochaete* Schum. in Engler, Pflanzenw. Ost-Afrikas C: 105. 1895*. *Type: Tanzania. Kilimandscharo, 1000 m, 26 Dec 1893, *G. Volkens 1605* (B, photo SI, E, photo SI, G, photo SI, K).

*Panicum oligochaete* (Schum.) Kneucker, Allg. Bot. Z. Syst 21: 28. 1915.

*Panicum sulcatum *var. *stenophyllum *Pilg., Bot. Jahrb. Syst. 33: 46. 1902. Syntypes: Togo. Bismarckburg, 1889, *Kling 225 *(B); Angola. Huilla, May 1895, *Antunes 31* (B).

*Panicum plicatile* var. *glabrescens *Chiov., Ann. Bot. (Rome) 8: 31. 1903. Lectotype (designated here): Eritrea. Amasen, Asmara, 2400 m, Aug- Sep 1892, *V. Ragazzi 43* (FT, photo SI).

*Panicum plicatile *var. *pilosum *Chiov., Annuario Reale Ist. Bot. Roma 8: 31. 1903. Type: Eritrea. Mensa, Rora Ualicaué (Ovest), 1900 m, 8 Jan 1893, *A. Terracciano & A. Pappi 670 *(FI, photo SI).

*Setaria caudula* Stapf, Fl. Trop. Afr. 9: 845. 1930. Type: Uganda. Kipayo, 4000 ft., Mar 1915, *R.A. Dümmer2416* (BR, fragment, photo SI, K, photo SI, WAG).

*Setaria chevalieri* Stapf & C.E. Hubb., Fl. Trop. Afr. 9: 842. 1930. Lectotype (designated here): Gabon. Mayumba, Jan 1904, *A.J.B. Chevalier 11282* (P, photo SI).

*Setaria megaphylla* var. *chevalieri* (Stapf & C.E. Hubb.) Berhaut, Fl. Senegal 2: 401. 1954.

*Setaria acuta* Stapf & C.E. Hubb., Fl. Trop. Afr. 9: 846. 1930. Type: Ethiopia. Amba Sea, 2300 m, 12 Mar 1863, *Schimper 789* (K, photo SI, PRE, photo SI).

*Setaria insignis* de Wit, Bull. Jard. Bot. Buitenzorg, ser. 3, 17: 13, 15. 1941. Type: South Africa. Cape Province, Port St. Johns, S.E. Cape, Eagle´s Nest, slope of Western Gate, 7 Feb 1924, *C. Howlett 35* (holotype, PRE, photo SI, isotype, L, fragment, photo SI).

*Setaria natalensis* de Wit, Bull. Jard. Bot. Buitenzorg, ser. 3, 17: 19. 1941. Type: South Africa. Natal, Kwazulu-Natal, *J. Buchanan 268* (holotype, NH; isotype, L, fragment, photo SI).

**Type.** “In marginibus fossarum udarum prope Catumby, non procul ad urbe Rio de Janeiro.” Brazil: Rio de Janeiro; *Raddi 17* (FI).

**Distribution. **Kerala, Tamil Nadu, Uttarakhand [also in Africa, North America, South America].

**Habit. **Perennial.

**Remarks.** Probably native to the Americas.

**748. **Setaria verticillata** (L.) P. Beauv., Ess. Agrostogr. 51, 171, 178. 1812 **

*Panicum verticillatum* L., Sp. Pl., ed. 2. 1: 82. 1762. Type: “Habitat in Europa australi & Oriente.” Lectotype: Herb. Linn. No. 80.7 (LINN). LT designated by Sherif & Siddiqi in El-Gadi (ed.), Fl. Libya 145: 296. 1988.

*Chaetochloa verticillata* (L.) Scribn., Bull. Div. Agrostol. U.S.D.A. 4: 39. 1897

*Ixophorus verticillatus* (L.) Nash, Bull. Torrey Bot. Club 22: 422. 1895

*Pennisetum verticillatum* (L.) R. Br. ex Roem. & Schult., Syst. Veg., ed. 15 bis [Roemer & Schultes]. 2: 488. 1817 in syn.

*Panicum adhaerens* Forssk., Fl. Aegypt.-Arab. 20. 1775. Type: “Hadie” Lectotype: Yemen, Al Hadiyah; 1763; *Forsskål s.n. *(LD). LT designated by Veldkamp in Blumea 39: 383. 1994.

*Setaria adhaerens* (Forssk.) Chiov., Nuovo Giorn. Bot. Ital. 26: 77. 1919

*Panicum aparine* Steud., Syn. Pl. Glumac. 1: 52. 1853. Type: “Jardin legit in Senegambia, Despreaux in Ins. Canar.”

*Pennisetum respiciens* A. Rich., Tent. Fl. Abyss. 2: 379. 1850–1851. Type: Ethiopia: “Crescit in convalle fluvii Tacazze prope Tchelatchekanne, mensibus Augusto ad Octobrem (Quartin Dillon et Schimper).”

*Panicum rottleri* (Spreng.) Nees, Fl. Afr. Austral. Ill. 53. 1841, non Kunth 1829

*Panicum asperum* Lam., Fl. Franc. (Lamarck). 3: 577. 1779 (“1778”), nom. superfl. & illeg. for *Panicumverticillatum*

*Setaria teysmannii* Miq., Fl. Ned. Ind. 3: 468. 1857. (‘*teysmanni*’). Type: Java., Besuki, Sumberwaru, *Teysmann s.n.* (holotype, U!).

*Panicum adhaerens* Forssk. var. *miquelii *A. Braun, Append. Gen. Sp. Hort. Berol. 1871: 7. 1872

*Setaria verticillata* P. Beauv. fo. *miquelii* (A. Braun) A. Braun ex Backer in Heyne, Nutt. Pl. Ned.-Ind. 1: 205. 1922

*Panicum verticillatum* L. fo. *miquelii* (A. Braun) A. Braun ex Backer in Heyne, Nutt. Pl. Ned.-Ind., ed. 2, 1: 204. 1927. Type: Java, 25 Nov 1843, *Zollinger 2729* (isotypes, G, P).

**Distribution.** Andhra Pradesh, Assam, Bihar, Chhattisgarh, Delhi, Goa, Gujarat, Himachal Pradesh, Jammu and Kashmir, Karnataka, Kerala, Madhya Pradesh, Maharashtra, Odisha, Rajasthan, Tamil Nadu, Uttar Pradesh [China, Pakistan].

**Habit.** Annual.

**749. **Setaria viridis** (L.) P. Beauv., Ess. Agrostogr. 51, 171, 178. 1812 **

*Panicum viride* L., Syst. Nat., ed. 10. 2: 870. 1759. Type: “Habitat [in Europa australi.] Sp. Pl., ed. 2, 1: 83. 1762” Lectotype: Browne, Herb. Linn. No. 80.12 (LINN). LT designated by Meikle in Fl. Cyprus 2: 1861. 1985.

**Distribution.** Delhi, Jammu and Kashmir, Meghalaya, Maharashtra, Tamil Nadu, Uttar Pradesh, Uttarakhand, West Bengal [China, Pakistan].

**Habit.** Annual.

***Spinifex* L., Mant. Pl. Altera 163, 300. 1771**

**Type. ***Spinifex squarrosus* L.

**750. **Spinifex littoreus** (Burm. f.) Merr., Philipp. J. Sci., C 7: 229. 1912 **

*Stipa littorea* Burm. f., Fl. Ind. (N. L. Burman) 29. 1768. Type: “Rompot laut Javanis” Rheed. Hort. Malab. 12. P. 143. t. 75.

*Spinifex squarrosus* L., Mant. Pl. Altera 300. 1771. Type: “Habitat in Indiae orientalis arenosis maritimis.” Lectotype: Herb. Linn. No. 1216.2 (LINN). LT designated by Heyligers in Cafferty et al. (ed.), Taxon 49: 257. 2000.

*Stipa spinifex* L., Mant. Pl. 34. 1767. Type: “Habitat in Indiae arenosis maritimis.” Lectotype = “Cyperus littoreus echinato capite” in Rumphius, Herb. Amboin., 6: 6, t. 2, f. 2, 1750. LT designated by Heyligers in Cafferty et al. (ed.), Taxon 49: 257. 2000.

**Distribution.** Andaman and Nicobar Islands, Andhra Pradesh, Goa, Gujarat, Karnataka, Kerala, Lakshadweep, Maharashtra, Odisha, Tamil Nadu, West Bengal [China, Myanmar, Sri Lanka].

**Habit.** Not recorded.

***Stenotaphrum* Trin., Fund. Agrost. (Trinius) 175. 1820**

**Type. ***Stenotaphrum glabrum* Trin., nom. superfl. (*Panicum dimidiatum* L., *Stenotaphrum dimidiatum* (L.) Brongn.).

**751. **Stenotaphrum dimidiatum** (L.) Brongn., Voy. Monde, Phan. 2(2): 127. 1831 **

*Panicum dimidiatum* L., Sp. Pl. 1: 57. 1753. Type: “Habitat in India.” Lectotype: Herb. Linn. No. 80.25 (LINN). LT designated by Renvoize in Cafferty et al. (ed.), Taxon 49: 253. 2000.

*Stenotaphrum glabrum* Trin., Fund. Agrost. (Trinius) 176. 1820

*Stenotaphrum koenigii* Schrank, Flora 7(2), Beil. 1: 28. 1824. Type: India; 1784; *Koenig s.n.*

*Stenotaphrum secundatum* sensu Alston, Handb. Fl. Ceylon 6(Suppl.): 327. 1931, non *S*. *secundatum* (Walter) Kuntze, 1891

**Distribution.** Bihar, Karnataka, Kerala, Maharashtra, Meghalaya, Tamil Nadu, West Bengal [Sri Lanka].

**Habit.** Perennial.

***Trachys* Pers., Syn. Pl. (Persoon) 1: 85. 1805**

**Type. ***Trachys mucronata* Pers., nom. illeg*.* (*Cenchrus muricatus* L., *Trachys muricata* (L.) Trin.

**752. **Trachys copeana** Kabeer & V.J. Nair, Kew Bull. 62(3): 503–505. f. 1. 2007 **

**Type.** India: Tamil Nadu, Kanyakumari District, between Kollengode and Chinnathurai, near Marthandam; September 18, 2003; *K.A.A. Kabeer 117134* (CAL).

**Distribution.** Tamil Nadu.

**Habit.** Annual or perennial.

**753. **Trachys deccanensis** M. Anil Kumar & B.R.P. Rao. Rheedea 27(2): 79. 2017 **

**Type.** India, Andhra Pradesh, Kurnool district, Kadivella near Yemmiganur, 16 November 2016, B. Ravi Prasad Rao, K. V. Subbaiah & M. Anil Kumar 52099 (holotype: SKU; isotype: CAL)

**Distribution.** Andhra Pradesh.

**Habit.** Annual.

**754. **Trachys muricata** (L.) Pers., Syn. Pl. (Persoon) 1: 85. 1805 (as “mucronata”) **

*Cenchrus muricatus* L., Mant. Pl. Altera 302. 1771. Type: “Habitat in India orientali. Koenig.” Neotype: India: Tamil Nadu, Madras, South Arcot, Chidambarum, Kille, Anuvampattu; *Venugopal in RHT No. 21360* (K). Neotype designated by Renvoize in Cafferty et al. (ed.), Taxon 49: 249. 2000.

*Trachyozus muricata* (L.) Steud., Syn. Pl. Glumac. 1: 112. 1854

*Panicum squarrosum* Retz., Observ. Bot. (Retzius) 4: 15. 1786–1787. Type: “In arenosis Malabariae tempore pluvioso frequens”;* Koenig s.n.*

**Distribution.** Andhra Pradesh, Gujarat, Karnataka, Kerala, Odisha, Tamil Nadu [Myanmar, Sri Lanka].

**Habit.** Annual.

**Remarks.** Persoon misspelled the Linnaean epithet “muricata” as “mucronata” and used the latter in his comb. nov. (a correctable error).

**755. **Trachys narasimhanii** Ravich., Rheedea 23(1): 22. 2013. **

**Type.** India: Tamil Nadu, Ennore, VGP Wonderland, 15 – 22 m, 28 November 1992, P. Ravichandran & D. Narasimhan 408 (holotype: MH; isotype: K, MCCH, SPKCESH).

**Distribution. **Tamil Nadu.

**Habit.** Annual.

***Tricholaena* Schrad., Mant. 2 (Schultes) 8, 163. 1824**

**Lectotype. ***Tricholaena micrantha* Schrad. (*Saccharum teneriffae* L. f., *Tricholaena teneriffae* (L. f.) Link.) LT designated by Hitchcock in Bull. U.S.D.A. 772: 241. 1920.

**756. **Tricholaena teneriffae** (L.f.) Link, Handbuch [Link] 1: 91. 1829 **

*Saccharum teneriffae* L.f., Suppl. Pl. 106. 1782 (“1781”). Type: “Habitat in Teneriffa”; *Fr. Masson s.n.* (LINN).

*Melinis teneriffae* (L.f.) Hack., Oesterr. Bot. Z. 51: 464. 1901

*Panicum plumosum* Presl, Fl. Sicul. (Presl) 1: 43(XLIII). 1826

*Panicum saccharoides* Trin., Gram. Panic. [Trinius] 245. 1826

*Agrostis plumosa* Ten., Fl. Napol. Suppl. 1: 49. 1811. Type: Italy: Naples, presumably

*Tricholaena micrantha* Schrad., Mant. 2 (Schultes) 163. 1824, nom. superfl. & illeg. for *Saccharum teneriffae*

**Distribution.** Karnataka, Punjab [Pakistan].

**Habit.** Perennial.

***Urochloa* P. Beauv., Ess. Agrostogr. 52. 1812**

**Type. ***Urochloa panicoides* P. Beauv.

**757. **Urochloa brizantha** (Hochst. ex A. Rich.) R.D. Webster, The Australian Paniceae (Poaceae) 223. 1987 var. **brizantha

*Panicum brizanthum* Hochst. ex A. Rich., Tent. Fl. Abyss. 2: 363. 1850–1851. Type: Ethiopia: Tigre: “In declivibus meridionalis partis mediae et superioris monte Sellenda prope Adouba.” October 3, 1837; *Schimper 89 *(P). LT designated by Veldkamp in Blumea 41(2): 417. 1996.

*Brachiaria brizantha* (Hochst. ex A. Rich.) Stapf, Fl. Trop. Afr. [Oliver et al.] 9: 531. 1919

**Distribution.** Kerala [Papua New Guinea, Africa, North America, South America].

**Habit.** Perennial.

**Remarks.** Record for India fide Naithani (1990). It is unclear if this taxon actually occurs in India or if this is a misapplication based on *Brachiaria decumben*s sensu Senaratna.

**758. **Urochloa brizantha** (Hochst. ex A. Rich.) R.D. Webster var. **ciliata** (Basappa & Muniy.) Ashalatha & V.J. Nair, Bulletin of the Botanical Survey of India 35(1–4): 29. 1993 **

*Brachiaria brizantha* (Hochst. ex A. Rich.) Stapf var. *ciliata* Basappa & Muniy., Proc. Indian Natl. Sci. Acad., B 49(4): 379. 1983

**Distribution.** Karnataka [Sri Lanka]. Native to Africa.

**Habit.** Perennial.

**Remarks.** Record for India fide Naithani (1990).

**759. **Urochloa chenaveeraiana** (Basappa & Muniy.) Ashalatha & V.J. Nair, Bulletin of the Botanical Survey of India 35(1–4): 28. 1993 **

*Brachiaria chennaveeraiana* Basappa & Muniy., Proc. Indian Natl. Sci. Acad., B 49(4): 378. 1983

**Distribution.** Rajasthan.

**Habit.** Not recorded.

**760. **Urochloa decumbens** (Stapf) R.D. Webster, Austral. Paniceae (Poaceae) 234. 1987 **

*Brachiaria decumbens* Stapf, Fl. Trop. Afr. [Oliver et al.] 9: 528. 1919. Syntypes: Uganda: Mengo District; *Dummer 1070* (K). Tanzania, Bukoba District; *Grant 488* (K).

*Brachiaria brizantha* sensu Senaratna, Grasses of Ceylon (Perad. Man. No. 8) 145. 1956, non Hochst. ex A. Rich., 1851

**Distribution.** Kerala, Rajasthan. Native to Africa.

**Habit.** Perennial.

**Remarks.** Record for Rajasthan fide Naithani (1990).

**761. **Urochloa deflexa** (Schumach.) H. Scholz, Bull. Mus. Natl. Hist. Nat., B, Adansonia Sér. 4, 11(4): 443. 1990 **

*Panicum deflexum* Schumach., Kongel. Danske Vidensk. Selsk. Naturvidensk. Math. Afh. 3: 83–84. 1827

*Brachiaria deflexa* (Schumach.) C.E. Hubb. ex Robyns Bull. Jard. Bot. Etat 9: 181. 1932 (“1931”)

*Pseudobrachiaria deflexa* (Schumach.) Launert, Mitt. Bot. Staatssamml. München 8: 158. 1970

*Brachiaria regularis* (Nees) Stapf, Fl. Trop. Afr. [Oliver et al.] 9: 544. 1919

*Panicum regulare* Nees, Fl. Afr. Austral. Ill. 41. 1841, in obs. Type: “Patria Guinea. Vidi in Herbario Lehmanniano.”

**Distribution.** Andhra Pradesh, Bihar, Madhya Pradesh, Maharashtra, Punjab, Rajasthan, Tamil Nadu, Uttar Pradesh [also in Saudi Arabia, Africa].

**Habit.** Annual.

**Remarks. **Records for Andhra Pradesh, Madhya Pradesh, Maharashtra, Punjab, Tamil Nadu, and Uttar Pradesh fide Naithani (1990).

**762. **Urochloa distachyos** (L.) T.Q. Nguyen, Novosti Sist. Vyssh. Rast. 1966: 13. 1966 (as “distachya”) **

*Panicum distachyon* L., Mant. Pl. Altera 183–184. 1771. Type: “Habitat in India orientali. Koenig.” Lectotype: Herb. Linn. No. 80.41 (LINN). LT designated by Henrard in Monogr. Digitaria 191. 1950

*Brachiaria distachyos* (L.) Stapf, Fl. Trop. Afr. [Oliver et al.] 9: 565. 1919 in obs. (as “distachya”)

*Digitaria distachyos* (L.) Pers., Syn. Pl. Glumac. 1: 41. 1853 (as “distachya”), cited as synonym of *Panicum bicorne* Sieber ex Steud.

*Panicum miliiforme* J. Presl in C. Presl, Reliq. Haenk. 1(4–5): 300. 1830. Type: “Habitat in Luzonia.” Philippine Islands: Luzon; *Haenke s.n.* (PR).

*Brachiaria miliiformis* (J. Presl) A. Chase, Contr. U.S. Natl. Herb. 22(1): 35. 1920

*Brachiaria subquadripara* (Trin.) Hitchc. var. *miliiformis* (J. Presl) S.L. Chen & Y.X. Jin, Acta Phytotax. Sin. 22(6): 472. 1984

*Brachiaria miliiformis* (J. Presl) Chase, Contr. U.S. Natl. Herb. 22(1): 35. 1920

**Distribution.** Andaman and Nicobar Islands, Andhra Pradesh, Assam, Arunachal Pradesh, Bihar, Chhattisgarh, Delhi, Gujarat, Jharkhand, Karnataka, Kerala, Madhya Pradesh, Maharashtra, Manipur, Meghalaya, Odisha, Punjab, Rajasthan, Tamil Nadu, Tripura, Uttar Pradesh, West Bengal [China, Myanmar, Nepal, Pakistan, Sri Lanka].

**Habit.** Annual.

**763. **Urochloa hybrida** (Basappa & Muniy.) Ashalatha & V.J. Nair, Bull. Bot. Surv. India 35(1–4): 28. 1997 (“1993”) **

*Brachiaria hybrida* Basappa & Muniy., Proc. Indian Natl. Sci. Acad., B, 49(4): 379. 1983

**Distribution.** Karnataka.

**Habit. **Not recorded.

**764. **Urochloa kurzii** (Hook. f.) T.Q. Nguyen, Novosti Sist. Vyssh. Rast. 1966: 13. 1966 **

*Panicum kurzii* Hook. f., Fl. Brit. India (J.D. Hooker). 7(21): 38. 1896. Type: Seebpore; *Kurz s.n.*(K)*.* LT designated by Veldkamp in Blumea 41(2): 422. 1996.

*Brachiaria kurzii* (Hook. f.) A. Camus, Fl. Indo-Chine [P.H. Lecomte et al.] 7: 438. 1922

*Brachiaria lanceata* Ohwi, Bull. Tokyo Sci. Mus. 18: 4. 1947. Type: Indonesia: Java, Jawa Timur, Kangean; 1920; *Backer 27829* (BO).

**Distribution. **Andhra Pradesh, Bihar, Karnataka, Madhya Pradesh, Odisha, Rajasthan, Tamil Nadu, Uttar Pradesh, West Bengal [also in Indonesia, Australia].

**Habit.** Annual.

**765. **Urochloa lata** (Schumach.) C.E. Hubb., Bull. Misc. Inform. Kew 1934: 112. 1934 var. **lata

*Panicum latum* Schumach., Beskr. Guin. pl. 61. 1827. Type: Ghana; *Thonning 353*

*Brachiaria lata* (Schumach.) C.E. Hubb., Hooker’s Icon. Pl. 34: sub. t. 3363. 1938

*Panicum insculptum* Steud., Syn. Pl. Glumac. 1: 49. 1853. Type: Guinea; *Jardin s.n.*

*Urochloa insculpta* (Steud.) Stapf, Fl. Trop. Afr. [Oliver et al.] 9: 599. 1920

**Distribution.** Rajasthan, Uttar Pradesh. Native to Africa.

**Habit.** Annual.

**766. **Urochloa lata** (Schumach.) C.E. Hubb. var. **pubescens** (C.E. Hubb.) E.A. Kellogg, comb. nov. **

urn:lsid:ipni.org:names:77212129-1

*Brachiaria lata* (Schumach.) C.E. Hubb. var. *pubescens* C.E. Hubb., Kew Bull. 4: 125. 1949. Type. Nigeria: Sokoto Province, Sokoto; July 30, 1910; *Dalziel 477*

**Distribution.** Rajasthan [also in Africa].

**Habit.** Annual.

**Remarks. **Record for India fide Naithani (1990). The above combination is made here to transfer *Brachiaria lata* (Schumach.) C.E. Hubb. var. *pubescens* C.E. Hubb. to *Urochloa *as a variety of *U*. *lata* (Schumach.) C. E. Hubb.

**767. **Urochloa mosambicensis** (Hack.) Dandy, J. Bot. 69(2): 54. 1931 **

*Panicum mosambicense* Hack., Bol. Soc. Brot. 6: 140. 1888. Type: “Prope Moçambique.” Mozambique; 1886; *M.R. de Carvalho 19* (W).

*Echinochloa notabilis* (Hook. f.) Rhind, Grasses of Burma 50. 1945 (as “notabile”)

*Panicum notabile* Hook. f., Fl. Brit. India (J.D. Hooker). 7(21): 32. 1896. Type: Burma, Petroleum wells of the Irawaddi; *Wallich s.n.*

**Distribution.** Rajasthan, Tamil Nadu [Myanmar]. Native to Africa.

**Habit.** Perennial.

**Remarks.** Record for Rajasthan fide Naithani (1990).

**768. **Urochloa munae** (Basappa) Ashalatha & V.J. Nair, Bull. Bot. Surv. India 35(1–4): 29. 1997 (“1993”) **

*Brachiaria munae* Basappa, Proc. Indian Acad. Sci. Pl. Sci. 93(1): 53. f. 1. 1984. Type: India: Tamil Nadu, Madurai Kamaraj University Campus; December 20, 1979; *Basappa 3001* (CAL).

**Distribution.** Andhra Pradesh, Tamil Nadu.

**Habit.** Annual.

**Remarks.** Record for Tamil Nadu fide Naithani (1990).

**769. **Urochloa mutica** (Forssk.) T.Q. Nguyen, Novosti Sist. Vyssh. Rast. 3: 13. 1966 **

*Panicum muticum* Forssk., Fl. Aegypt.-Arab. 20. 1775. Type: Egypt: “Rosettae” (Rashid); November 2–6, 1761; *Forsskal 86* (C).

*Brachiaria mutica* (Forssk.) Stapf, Prain, Fl. Trop. Afri. 9: 526. 1919

*Brachiaria purpurascens* (Raddi) Henrard, Blumea 3(3): 434. 1940

*Panicum amphibium* Steud., Syn. Pl. Glumac. 1: 61. 1853. Type: Java; *Zollinger no. 416* (P).

*Panicum barbinode* Trin., Sp. Gram. [Trinius] 3(27): t. 318. 1831. Type: “Figura ad specimen Bahiense.” Brazil: Bahia; 1831; *Riedel in Herb. Trinus 0601.01 *(LE).

*Panicum punctulatum* Arn. ex Steud., Syn. Pl. Glumac. 1: 62. 1853. Type: Sri Lanka; *Wight Herb. no. 2039*

*Panicum purpurascens* Raddi, Agrostogr. Bras. [Raddi] 47–48. 1823. Lectotype: Brazil: Rio de Janeiro “ Multum habet affinitatem cum precedenti cum quo mixtum invenitur, licet etiam sponte oriatur”; *G. Raddi s.n. *(PI). LT designated by Baldini & Longhi-Wagner Taxon 55(2): 479. 2006

*Panicum pictigluma* Steud., Syn. Pl. Glumac. 1: 73. 1853, nom superfl. & illeg. for *P. purpurascens* Raddi

*Panicum molle* sensu Griseb., Fl. Brit. W. I. (Grisebach) 547. 1864, non Sw., 17

**Distribution.** Andhra Pradesh, Assam, Bihar, Delhi, Karnataka, Kerala, Madhya Pradesh, Maharashtra, Odisha, Tamil Nadu, Uttar Pradesh, West Bengal [also in Indonesia, Africa, Australia, North America, South America].

**Habit.** Annual.

**770. **Urochloa nilagirica** (Bor) Ashalatha & V.J. Nair, Bull. Bot. Surv. India 35(1–4): 29. 1997 (“1993”) **

*Brachiaria nilagirica* Bor, Bot. Tidsskr. 67(4): 325. 1973. Type: India: Tamil Nadu, Nilgiri Hills; *Perrottet 1357* (K).

**Distribution.** Tamil Nadu.

**Habit.** Not recorded.

**771. **Urochloa oligotricha** (Fig. & De Not.) Henrard, Blumea 4(3): 502. 1941 **

*Panicum oligotrichum* Fig. & de Not., Agrost. Aegypt. 17, t. 10. 1853. Type: “ad amnem coeruleum in Nubia superiore.” Sudan: Nubia; *Figari s.n.*

*Helopus bolbodes* Hochst. ex Steud., Syn. Pl. Glumac. 1: 100. 1854. Type: Ethiopia; *Schimper 2021* (P) “Abyssin; Hochst Hrbr. Abyss. nr. 2021”

*Urochloa bolbodes* (Hochst. ex Steud.) Stapf, Fl. Trop. Afr. [Oliver et al.] 9(4): 593. 1920

*Eriochloa bolbodes* (Hochst. ex Steud.) Schweinf., Bull. Herb. Boissier 2 (App. 2): 17. 1894

*Panicum bolbodes* (Hochst. ex Steud.) Aschers., Beitr. Fl. Aethiop. 300. 1867

*Panicum numidianum* sensu Hack., Bull. Herb. Boissier 4(App. 3): 14. 1896, non Lam., 1791

**Distribution. **India [Africa].

**Habit.** Perennial.

**Remarks.** Distribution in India not recorded.

**772. **Urochloa ovalis** (Stapf) Zon, Blumea 64(3): 215. 2019 **

*Brachiaria ovalis* Stapf, Fl. Trop. Afr. [Oliver et al.] 9: 546. 1919. Syntypes: Somalia: Bohotle; *Appleton s.n.* (K). Somalia: Haud; *Thompson s.n.* (K). Ethiopia: Habab; *Hildebrandt 337* (K). Ethiopia; *Salt s.n.* (BM).

**Distribution. **Punjab [Pakistan].

**Habit.** Annual.

**Remarks. **The above combination is made here to transfer *Brachiaria ovalis* Stapf to *Urochloa*.

**773. **Urochloa panicoides** P. Beauv., Ess. Agrostogr. 53. t. 11. f. 1. 1812 **

*Panicum javanicum* Poir., Encycl. (Lamarck) Suppl. 4: 274. 1816. Type: “Cette plante croit a l’ile de Java (V.s. in herb. Desfont.)”; *Desfontaines s.n.*

*Urochloa marathensis* Henrard, Meded. Rijks-Herb. 43: 2. 1922. Type: “Hab. India orient: Southern Maratha Country and North Canara, Bombay Presidency, collected and presented by A.P. Young, Esq. I.N. Anno 1884 ex Herbario Musie Britannici in Herb. Lugd. Batav. Sub no. 90.92-2300.”

*Urochloa panicoides* P. Beauv. var. *marathensis* (Henrard) Bor, Grasses Burma, Ceylon, India and Pakistan 373. 1960

*Urochloa panicoides* P. Beauv. var. *velutina* (Henrard) Bor, Grasses Burma, Ceylon, India and Pakistan 373. 1960

*Urochloa marathensis* Henrard var. *velutina* Henrard, Meded. Rijks-Herb. 43: 2. 1922. Type: “Hab. cum praecedente Young legit in Herb. Lugd. Bat. Sub no. 908.83-143.”

*Urochloa panicoides* P. Beauv. var. *pubescens* (Kunth) Bor, Grasses Burma, Ceylon, India and Pakistan 372. 1960

*Urochloa pubescens* Kunth, Révis. Gramin. 1: 31. 1829. Type: “Insulae Mascarenae, Nova Hollandia.”

**Type.** Mauritius: Isle de France, to about 4800 ft., mainly in cultivated land; *Jussieu s.n*. (P-type is lost). Neotype: P. Beauv., Ess. Agrost. pl. 11. f. 1. 1812. NT designated by Veldkamp in Blumea 41: 433. 1996.

**Distribution.** Andhra Pradesh, Assam, Bihar, Chhattisgarh, Gujarat, Himachal Pradesh, Karnataka, Kerala, Madhya Pradesh, Maharashtra, Odisha, Rajasthan, Tamil Nadu, Uttar Pradesh, Uttarakhand, West Bengal [Bhutan, China, Myanmar, Nepal, Pakistan, Sri Lanka].

**Habit.** Annual.

**774. **Urochloa paspaloides** J. Presl in C. Presl, Reliq. Haenk. 1(4–5): 318. 1830 **

*Brachiaria paspaloides* (J. Presl) C.E. Hubb., Hooker’s Icon. Pl. 34: sub. t. 3363. 1938

*Panicum ambiguum* Trin., Mém. Acad. Imp. Sci. Saint-Pétersbourg, Sér. 6, Sci. Math., Seconde Pt. Sci. Nat. 3, 1(2–3): 243. 1834, non (DC.) Lapeyr. ex Le Turq., 1816

**Type.** “Habitat in Luzonia ad Sorzogon.” Philippine: Luzon Islands, Sorzogon; *Haenke s.n.* (PR).

**Distribution.** Assam, Uttar Pradesh [Myanmar, Sri Lanka].

**Habit.** Annual.

**775. **Urochloa pullulans** Stapf, Fl. Trop. Afr. [Oliver et al.] 9(4): 590. 1920 **

*Panicum geminatum* sensu Schweinf., Bull. Herb. Boissier 2App. 2): 19. 1894, non Forssk., 1775

**Distribution.** India [also in Africa].

**Habit.** Not recorded.

**Remarks.** Distribution in India not recorded.

**776. **Urochloa ramosa** (L.) T.Q. Nguyen, Novosti Sist. Vyssh. Rast. 1966: 13. 1966 **

*Panicum ramosum* L., Mant. Pl. 29. 1767. Type: “Habitat in Indiis.” Lectotype: *Herb. Linn. No. 80.44* (LINN). LT designated by Cope in Nasir and Ali (ed.), Fl. Pakistan 143: 207. 1982.

*Brachiaria ramosa* (L.) Stapf., Fl. Trop. Afr. [Oliver et al.] 9: 542. 1919

*Urochloa ramosa* (L.) R.D. Webster, Austral. Paniceae (Poaceae) 251. 1987, isonym.

*Panicum arvense* Kunth, Révis. Gramin. 2: 391. t. 109. 1831. Type: “Crescit ubique in arvis Senegaliae.” *Leprieur s.n.*

*Panicum brachylachnum* Steud., Syn. Pl. Glumac. 1: 62. 1853. Type: Senegalia; *Leprieur s.n.*

*Panicum canescens* Roth, Syst. Veg., ed. 15 bis [Roemer & Schultes] 2: 457. 1817. Type: India orientalis

*Panicum cognatissimum* Steud., Syn. Pl. Glumac. 1: 69. 1853. Type: Segegambia; *Leprieur s.n.*

*Urochloa supervacua* (C.B. Clarke) Noltie, Edinburgh J. Bot. 56(3): 394. 1999

*Panicum supervacuum* C.B. Clarke, J. Linn. Soc., Bot. 24: 407–408. f. A-E. 1888. Lectotype: India: West Bengal, Baladum, alt. 400 ft.; May 28, 1884; *C.B. Clarke 35103* (K). LT designated by Noltie in Edinburgh J. Bot. 56(3): 394. 1999.

*Brachiaria marselinii* Gawade & Gavade, J. Bombay Nat. Hist. Soc. 101(2): 291 -293. f. 1. 2004 (as “marselini”). Type: India: Maharashtra, Masure, Malvan, Sindhudurg; September 27, 2000; *N.D. Gawade 1442* (BLAT).

*Panicum patens* sensu Bojer, Hortus Maurit. 365. 1837, non L., 1753

**Distribution.** Andhra Pradesh, Bihar, Chhattisgarh, Daman and Diu, Goa, Gujarat, Karnataka, Kerala, Madhya Pradesh, Maharashtra, Rajasthan, Tamil Nadu, Uttar Pradesh, West Bengal [Bhutan, China, Nepal, Pakistan, Sri Lanka].

**Habit.** Annual.

**777. **Urochloa ramosa** (L.) T.Q. Nguyen var. **pubescens** (Basappa & Muniy.) E.A. Kellogg, comb. nov. **

urn:lsid:ipni.org:names:77212130-1

*Brachiaria ramosa* (L.) Stapf. var. *pubescens* Basappa & Muniy., Proc. Indian Natl. Sci. Acad., B, 49(4): 380. 1983

**Distribution.** Karnataka, Rajasthan.

**Habit.** Annual.

**Remarks.** Distribution in India fide Naithani (1990). The above combination is made here to transfer *Brachiaria ramosa* (L.) Stapf var. *pubescens* to *Urochloa*, as a variety of *U. ramosa*.

**778. **Urochloa remota** (Retz.) Ashalatha & V.J. Nair, Bull. Bot. Surv. India 35(1–4): 29. 1997 (“1993”) **

*Panicum remotum* Retz., Observ. Bot. (Retzius) 4: 17. 1786–1787. Type: “E Tranquebaria misit Opt. Koenig.”

*Brachiaria remota* (Retz.) Haines, Bot. Bihar Orissa 5: 1005. 1924

*Panicum petiveri* Trin., Gram. Panic. [Trinius] 144. 1826 (as “petiverii”). Type: India orientalis; *Lindley s.n.*

**Distribution.** Andhra Pradesh, Bihar, Gujarat, Karnataka, Kerala, Maharashtra, Odisha, Tamil Nadu, Uttar Pradesh [Sri Lanka].

**Habit.** Perennial.

**779. **Urochloa reptans** (L.) Stapf, Fl. Trop. Afr. [Oliver et al.] 9: 601. 1920 var. **reptans

*Panicum reptans* L., Syst. Nat., ed 10. 2: 870. 1759. Lectotype: Browne, Herb. Linn. No. 80.52, upper specimen (LINN). LT designated by Veldkamp. Blumea 41(2): 427. 1996.

*Brachiaria reptans* (L.) C.A. Gardner. & C.E. Hubb., Hooker’s Icon. Pl. 34: sub t. 3363. 1938

*Brachiaria reptans* (L.) Gard. var. *hispida* Basappa & Muniy., Proc. Indian Natl. Sci. Acad., B 49(4): 380. 1983

*Panicum prostratum* Lam., Tabl. Encycl. 1: 171. 1791. Type: Hispaniola: Dominican Republic, St. Dominique; *Anonymous in Herb. Lamarck* (P-LAM).

*Brachiaria prostrata* (Lam.) Griseb., Abh. Königl. Ges. Wiss. Göttingen 7: 263. 1857

*Panicum procumbens* Nees, Fl. Bras. Enum. Pl. 2: 109. 1829. Type: “*Panicum prostratum β *Lam., Encycl. (Lamarck). 4:740.”

*Panicum umbrosum* Retz., Observ. Bot. (Retzius) 4: 16. 1786–1787. Type: “Habitat in graminosis umbrosis Indiae Orientalis. Misit Cel. Koenig.” India; *Koenig s.n. in herb. Retz* (LD).

**Distribution.** Andaman and Nicobar Islands, Andhra Pradesh, Assam, Bihar, Chhattisgarh, Daman and Diu, Delhi, Goa, Gujarat, Haryana, Jammu and Kashmir, Karnataka, Kerala, Madhya Pradesh, Maharashtra, Odisha, Punjab, Rajasthan, Tamil Nadu, Uttar Pradesh, West Bengal [Malaysia, Oceanic Islands, Africa, Australia, China].

**Habit.** Annual.

**Remarks.** The taxonomic editors question whether *Brachiaria reptans* (L.) Gard. var. *hispida* should be recognized as distinct; it is placed in synonymy here.

**780. **Urochloa semiundulata** (Hochst. ex A. Rich.) Ashalatha & V.J. Nair, Bull. Bot. Surv. India 35(1–4): 30. 1997 (“1993”) var. **semiundulata

*Panicum semiundulatum* Hochst. ex A. Rich., Tent. Fl. Abyss. 2: 364. 1850–1851. Type: “Crescit in pratis et locis graminosis prope Adoua, nec non in planitie montana provinciae Chiré, mensibus Septembre et Octobre (Schimper).” Syntypes: Ethiopia: Schire; October 10, 1840; *Schimper 1833*. Ethiopia: Adoam; September 25, 1837; *Schimper 289*

*Brachiaria semiundulata* (Hochst. ex A. Rich.) Stapf, Fl. Trop. Afr. [Oliver et al.] 9: 556. 1919

*Panicum nilagiricum* Steud., Syn. Pl. Glumac. 1: 62. 1853. Type: India: Tamil Nadu, Nilgiri Mountains; *R.F. Hohenacker 919*

*Panicum villosum* sensu Hook. f., Fl. Brit. India (J.D. Hooker). 7(21): 34. 1896, non Lam., 1791

**Distribution.** Andaman and Nicobar Islands, Andhra Pradesh, Assam, Bihar, Chhattisgarh, Daman and Diu, Delhi, Goa, Gujarat, Haryana, Jammu and Kashmir, Karnataka, Kerala, Madhya Pradesh, Maharashtra, Odisha, Punjab, Rajasthan, Tamil Nadu, Uttar Pradesh, West Bengal [China, Sri Lanka].

**Habit.** Annual.

**Remarks.** Record for Tamil Nadu fide Naithani (1990).

**781. **Urochloa semiundulata** (Hochst. ex A. Rich.) Ashalatha & V.J. Nair var. **intermedia** (Basappa & Muniy.) E. A. Kellogg, comb. nov. **

urn:lsid:ipni.org:names:77212131-1

*Brachiaria semiundulata* (Hochst. ex A. Rich.) Stapf var. *intermedia* Basappa & Muniy., Proc. Indian Natl. Sci. Acad., B 49(4): 380. 1983

**Distribution.** Karnataka (Naithani), Kerala, Maharashtra, Tamil Nadu.

**Habit. **Annual.

**Remarks.** The above combination is made here to transfer *Brachiaria semiundulata* (Hochst. ex A. Rich.) Stapf var. *intermedia* Basappa & Muniy. to *Urochloa* as a variety of *U. semiundulata* (Hochst. ex A. Rich.) Ashalatha & V.J.Nair.

**782. **Urochloa semiundulata** (Hochst. ex A. Rich.) Ashalatha & V.J. Nair var. **lanata** (Basappa & Muniy.) Ashalatha & V.J. Nair, Bull. Bot. Surv. India 35(1–4): 30. 1997 (“1993”) **

*Brachiaria semiundulata* (Hochst. ex A. Rich.) Stapf var. *lanata* Basappa & Muniy., Proc. Indian Natl. Sci. Acad., B 49(4): 380. 1983

**Distribution.** Karnataka, Kerala, Maharashtra, Tamil Nadu.

**Habit.** Annual.

**Remarks.** Record for Karnataka fide Naithani (1990).

**783. **Urochloa semiverticillata** (Rottler ex Steud.) Ashalatha & V.J. Nair, Bull. Bot. Surv. India 35(1–4): 30. 1997 (“1993”) **

*Brachiaria semiverticillata* (Rottler ex Steud.) Alston in Trimen, Handb. Fl. Ceylon 6(suppl.): 318. 1931

*Panicum semiverticillatum *Rottler ex Steud., Syn. Pl. Glumac. 1: 62, 1853. Type: India; *Nees s.n.*

*Panicum semiverticillatum* Rottler in Ainslie, Mat. Med. Hindoostan ed. 1, 219. 1813, nom. nud.?

*Panicum firmiculme* Mez, Notizbl. Bot. Gart. Berlin-Dahlem 7(63): 65. 1917. Type: Sri Lanka; *Thwaites 595* (as “895”)

**Distribution.** Kerala, Tamil Nadu [Sri Lanka].

**Habit.** Perennial.

**784. **Urochloa setigera** (Retz.) Stapf, Fl. Trop. Afr. [Oliver et al.] 9: 598. 1920 var. **setigera

*Panicum setigerum* Retz., Observ. Bot. (Retzius) 4: 15. 1786–1787. Type: “E China misit Cel. Bladh.” China: Guangdong, Guangzhou; *Bladh s.n.* (LD).

*Brachiaria setigera* (Retz.) C.E. Hubb., Hooker’s Icon. Pl. 34: sub. t. 3363. f. 2. 1938

*Panicum affine* Poir., Encycl. (Lamarck) Suppl. 4: 273. 1816. Type: “Cette espece croit dans les Indes orientales.”

**Distribution.** Andhra Pradesh, Bihar, Gujarat, Kerala, Maharashtra, Rajasthan, Tamil Nadu, West Bengal [Myanmar, Sri Lanka].

**Habit.** Perennial.

**785. **Urochloa setigera** (Retz.) Stapf var. **albistyla** (Basappa & Muniy.) S. Karthikeyan, Fl. Ind. ser. 4, 1 (Monocotyledon): 273. 1989 **

*Brachiaria setigera* (Retz.) C.E. Hubb. var. *albistyla* Basappa & Muniy., Proc. Indian Acad. Sci., B. 49: 380. 1983. Type: India

**Distribution.** Andhra Pradesh, Bihar, Maharashtra, Tamil Nadu, West Bengal [China, Myanmar, Nepal, Sri Lanka].

**Habit.** Perennial.

**Remarks.** Record for Tamil Nadu fide Naithani (1990).

**786. **Urochloa stapfiana** (Basappa & Muniy.) Ashalatha & V.J. Nair, Bull. Bot. Surv. India 35(1–4): 30. 1997 (“1993”) **

*Brachiaria stapfiana* Basappa & Muniy., Proc. Indian Natl. Sci. Acad., B 49(4): 379. 1983

**Distribution.** Karnataka.

**Habit. **Perennial.

**787. **Urochloa subquadripara** (Trin.) R.D. Webster, Austral. Paniceae (Poaceae) 252. 1987 **

*Panicum subquadriparum* Trin., Gram. Panic. [Trinius] 145. 1826. Lectotype: Mariana Islands; *Chamisso s.n. in Herb. Trinius 0947.01* (LE). LT designated by Veldkamp in Blumea 41(2): 429. 1996.

*Brachiaria subquadripara* (Trin.) Hitchc., Lingnan Sci. J. 7: 214. 1931

*Panicum distachyum* sensu Thwaites, Enum. Pl. Zeyl. (Thwaites). 359. 1864, non L., 1767

**Distribution. **Andaman and Nicobar Islands, Assam, Bihar, Delhi, Karnataka, Kerala, Odisha, Tamil Nadu, West Bengal [China, Sri Lanka].

**Habit.** Annual.

**788. **Urochloa trichopus** (Hochst.) Stapf, Fl. Trop. Afr. [Oliver et al.] 9(4): 589. 1920 **

*Panicum trichopus* Hochst., Flora 27: 254. 1844. Type: “Habitat in cultis provinciae Cordofan”; *Kotschy 74*

**Distribution.** Uttar Pradesh [also in Africa].

**Habit.** Annual.

**789. **Urochloa villosa** (Lam.) T.Q. Nguyen, Novosti Sist. Vyssh. Rast. 1966: 14. 1966 var. **villosa

*Panicum villosum* Lam., Tabl. Encycl. 1(1): 173. 1791. Type: India; *Sonnerat s.n. *(P-LAM).

*Brachiaria villosa* (Lam.) A. Camus, Fl. Indo-Chine [P.H. Lecomte et al.] 7: 433. 1922

*Panicum coccospermum* Steud., Syn. Pl. Glumac. 1: 62. 1853. Type: India orientalis; *Royle Herb. no. 43. 44*

*Panicum nanum* Nees, Syn. Pl. Glumac. (Steudel) 1: 62. 1853. Type: Nepal; *Royle Herb. no. 45*

*Panicum grossarium* sensu Roxb., Fl. Ind. (Carey and Wallich ed.) 1: 300. 1820, non L., 1759

**Distribution.** Assam, Manipur, Meghalaya, Nagaland, Rajasthan, Sikkim, Tamil Nadu, Uttar Pradesh, Uttarakhand [Bhutan, China, Myanmar, Nepal].

**Habit.** Annual.

**790. **Urochloa villosa** (Lam.) T.Q. Nguyen var. **barbata** (Bor) Noltie, Edinburgh J. Bot. 56(3): 395–396. 1999 **

*Brachiaria villosa* (Lam.) A. Camus var. *barbata* Bor, Grass. Bur. Ceyl. Ind. Pak. 386. 1960.

**Distribution.** Uttar Pradesh [Nepal].

**Habit.** Annual.

**791. **Urochloa villosa** (Lam.) T.Q. Nguyen var. **glaberrima** (Basappa & Muniy.) Ashalatha & V.J. Nair, Bull. Bot. Surv. India 35(1–4): 30. 1997 (“1993”) **

*Brachiaria villosa* (Lam.) A. Camus var. *glaberrima* Basappa & Muniy., Proc. Indian Natl. Sci. Acad., B 49(4): 381. 1983

**Distribution.** Meghalaya, Gujarat, Punjab, Rajasthan, Sikkim, Tamil Nadu, Uttar Pradesh [Nepal].

**Habit.** Annual.

**Remarks.** Record for Punjab fide Naithani (1990).

### Tribe Jansenelleae Voronts., 2020

Plant annual. Ligue membranous. Inflorescence dense, capitate, with short branches. Spikelets laterally compressed. Glumes acuminate or awned, lemmas awned. Hilum punctate. Leaf anatomy C3.

The two genera of Jansenelleae were provisionally placed in Tristachyideae by Kellogg (2015b), pending further data. However, recent molecular phylogenetic work (Bianconi et al. 2020) has shown that they are sisters, and form a clade that itself is sister to Andropogoneae s.l. Species of Jansenelleae appear to be C3 and thus help illuminate the origin and development of the C4 pathway in their sister tribe Andropogoneae (Bianconi et al. 2020).

***Chandrasekharania* V.J. Nair, V.S. Ramach. & Sreek., Proc. Indian Acad. Sci. Pl. Sci. 91(2): 79. 1982**

**Type. ***Chandrasekharania keralensis* V.J. Nair, V.S. Ramach. & Sreek.

**792. **Chandrasekharania keralensis** V.J. Nair, V.S. Ramach. & Sreek., Proc. Indian Acad. Sci. Pl. Sci. 91(2): 80. 1982 **

**Type.** India: Kerala, Cannanore District, Kannoth, alt. 500 ft.; February 18, 1978; *V.S. Ramachandran 54064* (CAL).

**Distribution.** Kerala, Karnataka, Maharashtra.

**Habit.** Annual.

**Remarks.** Distribution records fide Naithani (1990).

***Jansenella* Bor, Kew Bull. 1955: 96. 1955**

**Type. ***Jansenella griffithiana* (Müll. Hal.) Bor (*Danthonia griffithiana* Müll. Hal.).

**793. **Jansenella griffithiana** (Müll. Hal.) Bor, Kew Bull. 1955: 98. 1955 **

*Danthonia griffithiana* Müll. Hal., Bot. Zeitung (Berlin) 14(20): 347. 1856. Type: India: Assam, Khasiya; *Griffith 36* (B).

*Arundinella griffithiana* (Müll. Hal.) Bor, Indian Forester Rec., Bot. n.s. 1(3): 73. 1938

*Danthoniopsis griffithiana* (Müll. Hal.) Bor, Fl. Assam 5: 187. 1940

*Arundinella avenacea* Munro ex Thwaites, Enum. Pl. Zeyl. (Thwaites). 362. 1864. Type: Sri Lanka: Saffragam District, at no great elevation; *Munro Ceylon Plant 3471*

*Arundinella campbelliana* Lisboa, J. Bombay Nat. Hist. Soc. 5: 346. 1890. Type: India: Mahableshwar; 1889; *Lisboa s.n.*

**Distribution.** Arunachal Pradesh, Assam, Chhattisgarh, Goa, Karnataka, Kerala, Madhya Pradesh, Maharashtra, Odisha, Tamil Nadu [Myanmar, Sri Lanka].

**Habit.** Annual.

### Tribe Andropogoneae Dumort., 1824

Plants with complex inflorescences, often highly branched and/or bracteate. Spikelets borne in pairs, one sessile and the other pedicellate, the two often differentiated in structure and function. Branches sometimes ending a spikelet morphologically identical to the pedicellate one so that the spikelets appear to be in triplets. Glumes firm to indurate, the lemma and palea generally hyaline. In most species other than those in *Arundinella* and *Garnotia* the rachis disarticulates between the spikelet pairs. *Photosynthetic pathway C4, NADP-ME subtype*.

Taxonomic editor: Elizabeth A. Kellogg

**Andropogon L., Sp. Pl. 2: 1045. 1753, nom. cons.**

**Type.***Andropogon distachyos *L. (as “distachyon”) (*typ. cons.*)

**794. **Andropogon chinensis** (Nees) Merr., Philipp. J. Sci., C. 12(2): 101. 1917 **

*Homoeatherum chinense* Nees, Nat. Syst. Bot. 448. 1836

*Andropogon apricus* var. *chinensis* (Nees) Hack., Monogr. Phan. [A. DC. & C. DC.] 6: 457. 1889

*Andropogon ascinodis* C.B. Clarke, J. Linn. Soc., Bot. 25: 87. t. 36. 1889. Type: India: Assam, Jakpho, alt. 7500 ft.; *C.B. Clarke no. 41890*

*Andropogon apricus* sensu Hook. f., Fl. Brit. India (J.D. Hooker). 7(21): 169. 1896, non Trin., 1836

**Distribution.** Andhra Pradesh, Assam, Bihar, Karnataka, Manipur, Meghalaya, Nagaland, Odisha, Tamil Nadu [China, Myanmar].

**Habit.** Perennial.

**795.** *Andropogon gayanus* **Kunth, Enum. Pl. (Kunth) 1: 491. 1833**

*Andropogon reconditus* Steud., Syn. Pl. Glumac. 1: 386. 1854. Type: Africa: “Leprieur legit in Senegalia”; *Leprieur s.n.*

*Andropogon tomentellus* Steud., Syn. Pl. Glumac. 1: 371. 1854. Type: Africa: “Leprieur legit in Senegalia”; *Leprieur s.n.*

*Andropogon guineensis* Steud., Syn. Pl. Glumac. 1: 371. 1854, nom. Later hom., non Schumach., 1828

**Type.** Senegalia.

**Distribution.** India [also in Africa]. Naturalized; native to Africa.

**Habit. **Perennial.

**Remarks.** Distribution in India not recorded.

**796. **Andropogon hallii** Hack., Sitzungsber. Kaiserl. Akad. Wiss., Math.-Naturwiss. Cl. 89: 127. 1884 **

*Sorghum hallii* (Hack.) Kuntze, Revis. Gen. Pl. 2: 791. 1891

*Andropogon chrysocomus* Nash, Man. Fl. N. States (Britton) 70. 1901. Lectotype: USA: Kansas, Stevens County; July 24, 1891; *M.A. Carleton 343 *(NY-69769). LT designated by Nash in N. Amer. Fl. 17(2): 120. 1912.

*Andropogon geminatus* Hack. ex Beal, Grasses N. Amer. 2: 55. 1896. Type: USA: Texas; 1889; *Nealley s.n.* (US-728855).

*Andropogon paucipilus* Nash, Man. Fl. N. States (Britton) 70. 1901. Lectotype: USA: Nebraska, Grant County, 3 miles NE of Whitman; 31 Jul 1893; *P.A. Rydberg 1607* (NY-69766). LT designated by Nash in North Amer. Fl. 17(2): 121. 1912.

**Type.** USA: Nebraska; 1862; *E. A. Hall & J.P. Harbour 651 *(W).

**Distribution.** Tamil Nadu. Naturalized; native to North America.

**Habit.** Perennial.

**Remarks. **This species may not be distinct from *A. gerardi* (Estep et al. 2014).

**797. **Andropogon lividus** Thwaites, Enum. Pl. Zeyl. (Thwaites). 367. 1864 **

*Cymbopogon lividus* (Thwaites) Willis, Revis. Cat. Fl. Pl. Ceylon 110. 1911

**Type.** Sri Lanka: Central Province, Newera Ellia and other of more elevated parts; *Thwaites Ceylon Plant 953.*

**Distribution.** Kerala, Tamil Nadu [Sri Lanka].

**Habit.** Perennial.

**798. **Andropogon longipes** Hack., Flora 68: 138. 1885 **

**Type.** India: Tamil Nadu, Nilgerries; *Perrotet 1315.*

**Distribution.** Tamil Nadu.

**Habit.** Perennial.

**799. **Andropogon munroi** C.B. Clarke, J. Linn. Soc., Bot. 25: 87–88. t. 37. 1889 **

*Andropogon hookeri* Munro ex Hack., Monogr. Phan. [A. DC. & C. DC.] 6: 614. 1889. Type: Bhutan; *W. Griffith 6767* (W).

*Cymbopogon hookeri* (Munro ex Hack.) Stapf ex Bor, Indian Forest Rec., Bot. n.s. 1(3): 92. 1938

*Andropogon tristis* Nees ex Hack., Monogr. Phan. [A. DC. & C. DC.] 6: 439. 1889. Syntypes: Nepal; Royle 227. India: Kamaon alt. 10000 ft.; *Duthie s.n.* Himalaya; *J.D. Hooker & Thomson s.n.* Massuri; *Hugel s.n.* (C). Chini; *Stoliczka s.n.* (C).

**Type.** India: North Muneypore, alt. 3500 ft.; *C.B. Clarke 41961.*

**Distribution.** Assam, Manipur, Meghalaya, Uttar Pradesh, Uttarakhand [Bhutan, China, Nepal, Pakistan].

**Habit.** Perennial.

**800. **Andropogon polyptychos** Steud., Syn. Pl. Glumac. 1: 380. 1854 **

*Dichanthium polyptychon* (Steud.) A. Camus, Bull. Mus. Natl. Hist. Nat. 27: 549. 1921

*Andropogon polyptychos* Steud. var. *deccanensis* Bor, Grasses Burma, Ceylon, India and Pakistan 91. 1960, nom. inval.? Type: India: Madras; *Bourne 1622, 1337*

*Dichanthium polyptychon* (Steud.) A. Camus var. *deccanensis* Bor, Grasses Burma, Ceylon, India and Pakistan 91. 1960

**Type.** Sri Lanka.

**Distribution.** Himachal Pradesh, Jammu and Kashmir, Kerala, Madhya Pradesh, Tamil Nadu [Myanmar, Sri Lanka].

**Habit.** Perennial.

**Remarks.** Records for Kerala and Tamil Nadu fide Naithani (1990).

**801. **Andropogon pumilus** Roxb., Fl. Ind. (Carey & Wallich ed.) 1: 277. 1820 **

*Andropogon demissus* Steud., Syn. Pl. Glumac. 1: 388. 1854. Syntypes: India; Numer. List [Wallich] no. 8799. India; Numer. List [Wallich] no 8798 A.B.

*Andropogon pachyarthrus* Hack., Monogr. Phan. [A. DC. & C. DC.] 6: 449. 1889, nom. superfl. & illeg.

**Type.** India: “A native of Coromandel.”

**Distribution.** Andhra Pradesh, Bihar, Daman and Diu, Goa, Gujarat, Karnataka, Kerala, Himachal Pradesh, Karnataka, Kerala, Madhya Pradesh, Maharashtra, Odisha, Rajasthan, Tamil Nadu, Uttar Pradesh [Myanmar, Nepal].

**Habit.** Annual.

***Apluda* L., Sp. Pl. 1: 82. 1753**

**Type. ***Apluda mutica* L., Sp. Pl. 1: 82. 1753.

**802. **Apluda mutica** L., Sp. Pl. 1: 82. 1753 **

*Calamina mutica* (L.) P. Beauv., Ess. Agrostogr. 151 1812

*Andropogon glaucus* Retz., Observ. Bot. (Retzius) 5: 20. 1788 (“1789”) (as “glaucum”). Type: “Misit opt. Koenig, qui Anthistiriae speciem credidit.”

*Apluda aristata* L., Cent. Pl. II 7. 1756. Type: “Habitat in India.” Lectotype: Herb. Linn. No. 1213.4 (LINN). LT designated by Renvoize in Cafferty et al. (ed.), Taxon 49: 246. 2000.

*Apluda aristata* L. var. *jainii* R.K. Jain, Ind J. For. 9:345. 1986.

*Apluda geniculata* Roxb., Fl. Ind. (Carey and Wallich ed.) 1: 327. 1820. Type: India: “found in the banks of the Gangas.”

*Apluda varia* Hack., Monogr. Phan. [A. DC. & C. DC.] 6: 196. 1889. Type: Cited many syntypes

*Apluda varia* Hack. ssp. *aristata* Hack., Monogr. Phan. [A. DC. & C. DC.] 6: 196. 1889. Type: Cited many syntypes

*Apluda varia* Hack. var. *ciliata* (Andersson) Hook. f., Fl. Brit. India (J.D. Hooker). 7(21): 151. 1896

*Apluda varia* Hack. var. *rostrata* (Nees ex Arn.) Hook. f., Fl. Brit. India (J.D. Hooker). 7(21): 151. 1896

*Apluda varia* Hack. var. *villosula* Hook. f., Fl. Brit. India (J.D. Hooker). 7(21): 151. 1896. Type: India: *Wight Cat. no. 1713*

*Apluda varia* Hack. var. *humilis* Hook. f., Fl. Brit. India (J.D. Hooker). 7(21): 151. 1896

*Apluda ciliata* Andersson, Ofvers. Forh. Kongl. Svenska Vetensk.-Akad. 12: 177. 1855. Type: India Orientali, Carnar; *Hugel 1639*

*Apluda rostrata* Nees ex Arn., Nov. Actorum Acad. Caes. Leop.-Carol. Nat. Cur., Suppl. 19 (1): 194–195. 1843. Type: Sri Lanka; *Walker s.n.*

*Anthistiria gigantea* Cav., Icon. (Cavanilles) 5(1): 36. t. 458. 1799. Type: “Habitat in Luzon philippica insula tribus leucis ab oppido Guaz. Floret Martio et Aprili. Vidi siccam in eodem herbario.”

*Apluda gigantea* (Cav.) Spreng., Syst. Veg. (ed. 16) [Sprengel] 1: 290. 1824 (“1825”)

*Calamina gigantea* (Cav.) P. Beauv., Ess. Agrostogr. 157 1812

*Apluda mutica *L. var. *major* (Hack.) R.K. Jain, Ind. J. For. 9:347. 1986

*Apluda blatteri* Sur, Bull. Bot. Surv. Ind. 26: 193. 1986

*Calamina humilis* J. Presl in C. Presl, Reliq. Haenk. 1(4–5): 344. 1830, non Roem. & Schult., 1817

*Apluda mutica* L. var. *aristata* (L.) Pilg., Nat. Pflanzenfam., ed. 2 [Engler & Prantl] 14e: 130. 1940, nom. illeg. & hom., non *Apluda mutica* var. *aristata* (L.) Hack. ex Backer, 1928

**Type.** “Habitat in India.” Lectotype: Herb. Linn. No. 1213.1 (LINN). LT designated by Cope in Jarvis et al. (ed.), Regnum Veg. 127: 20. 1993.

**Distribution.** Andhra Pradesh, Arunachal Pradesh, Assam, Bihar, Chhattisgarh, Goa, Madhya Pradesh, Maharashtra, Manipur, Meghalaya, Karnataka, Kerala, Odisha, Rajasthan, Tamil Nadu, Uttar Pradesh, Uttarakhand, West Bengal [Afghanistan, Bhutan, China, Myanmar, Nepal, Pakistan, Sri Lanka].

**Habit.** Perennial.

**Remarks.** Record for Uttar Pradesh fide Naithani (1990).

***Apocopis* Nees, Proc. Linn. Soc. London 1: 93. 1841**

**Type. ***Apocopis royleanus* Nees.

**803. **Apocopis cochinchinensis** A. Camus, Bull. Mus. Hist. Nat. (Paris) 25: 286. 1919 **

**Type.** “Cochinchine (Pierre).” Vietnam; *J.B.L. Pierre s.n.* (US-1062404).

**Distribution.** Karnataka, Kerala [China].

**Habit.** Annual.

**804. **Apocopis courtallumensis** (Steud.) Henrard, Blumea 4(3): 524. 1941 **

*Andropogon courtallumensis* Steud., Syn. Pl. Glumac. 1: 377. 1854. Type: India: Courtallum; *Wight s.n.*

*Apocopis wightii* Nees ex Thwaites, Enum. Pl. Zeyl. (Thwaites). 365. 1864, nom. superfl. & illeg. for *Andropogon courtallumensis*

**Distribution.** Andhra Pradesh, Karnataka, Kerala, Tamil Nadu [Myanmar, Sri Lanka].

**Habit.** Annual.

**805. **Apocopis mangalorensis** (Hochst. ex. Steud.) Henrard, Blumea 4(3): 523. 1941 **

*Amblyachyrum mangalorense* Hochst. ex Steud., Syn. Pl. Glumac. 1: 413. 1854. Type: India; 1847; *R.F. Hohenacker 231* (P).

*Apocopis wightii* Nees ex Thwaites. ssp. *mangalurensis* (Hochst. ex Steud.) Hack., Monogr. Phan. [A. DC. & C. DC.] 6: 259. 1889

*Apocopis wightii* Nees ex Thwaites. var. *zeylanica* Hack., Monogr. Phan. [A. DC. & C. DC.] 6: 259. 1889. Type: Sri Lanka; *Thwaites 401*

*Apocopis courtallumensis* sensu Senaratna, Grasses of Ceylon (Perad. Man. No. 8) 171. 1956, non (Steud.) Henrard., 1941

**Distribution.** Karnataka, Kerala, Maharashtra, Tamil Nadu [Myanmar, Sri Lanka].

**Habit.** Annual.

**806. **Apocopis paleaceus** (Trin.) Hochr., Bull. New York Bot. Gard 6: 262. 1910 (as “paleacea”) **

*Ischaemum paleaceum* Trin. Mém. Acad. Imp. Sci. St.-Pétersbourg, Sér. 6, Sci. Math. 2: 293. 1833. Type: Nepal

*Andropogon paleaceus* (Trin.) Steud., Syn. Pl. Glumac. 1: 376. 1854

*Apocopis royleanus* Nees, Proc. Linn. Soc. London 1: 94. 1841. Type: Not given

*Apocopis himalayensis* Will.Watson in Atkins., Bot. Himalayan Distr. N.W. Prov. 392. 1882, nom. superfl. & illeg.

*Andropogon himalayensis* Steud., Syn. Pl. Glumac. 1: 377. 1854, nom. superfl. & illeg. for *Apocopisroyleanus* Nees.

**Distribution.** Assam, Manipur, Meghalaya, West Bengal [Bhutan, China, Myanmar, Nepal].

**Habit.** Perennial.

**807. **Apocopis vaginatus** Hack., Oesterr. Bot. Z. 41: 8. 1891 **

*Apocopis wightii* Nees ex Thwaites var. *vaginata* (Hack.) Hook. f., Fl. Brit. India (J.D. Hooker). 7(21): 143. 1896

**Type.** India: Hazaribagh; *C.B. Clarke 33849* (W).

**Distribution.** Andhra Pradesh, Bihar, Chhattisgarh, Daman and Diu, Goa, Gujarat, Karnataka, Kerala, Madhya Pradesh, Maharashtra, Odisha, Tamil Nadu, West Bengal, Uttar Pradesh, Uttarakhand [Myanmar].

**Habit.** Annual.

***Arthraxon* P. Beauv., Ess. Agrostogr. 111. 1812**

**Type. ***Arthraxon ciliare* P. Beauv.

**808. **Arthraxon castratus** (Griff.) V. Naray. ex Bor, Fl. Assam 5: 376. 1940 **

*Andropogon castratus* Griff., Not. Pl. Asiat. 3: 89. 1851. Type: “Suddah in Campis graminosis; January 2, 1836; *It. Ass. 292*.”(K).

*Andropogon rudis* Steud., Syn. Pl. Glumac. 1: 383. 1854. Type: [Bangladesh] India: Silhet; Numer. List [Wallich] no. 8837

*Arthraxon rudis* (Steud.) Hochst., Flora 39: 188. 1856 [basionym reference not given]

**Distribution.** Assam, Bihar, Karnataka, Kerala, Manipur, Meghalaya, Odisha, Tamil Nadu, West Bengal [Bangladesh, China, Myanmar, Sri Lanka].

**Habit.** Perennial.

**809. **Arthraxon depressus** Stapf ex C.E.C. Fisch., Bull. Misc. Inform. Kew 1933: 349–350. 1933 **

**Type.** India: “Peninsula, precise locality not indicated”; *Wight 3372* (K).

**Distribution.** Karnataka, Maharashtra, Tamil Nadu.

**Habit.** Annual.

**Remarks.** Distribution in India fide Naithani (1990).

**810. **Arthraxon hispidus** (Thunb.) Makino, Bot. Mag. (Tokyo) 26: 214. 1912 var. **hispidus

*Phalaris hispida* Thunb. in J.A. Murray, Syst. Veg. ed. 14: 104. 1784. Type: Japan; *C.P. Thunberg 1776 *(UPS).

*Alectoridia quartiniana* A. Rich., Tent. Fl. Abyss. 2: 448. t. 102. 1850–1851. Type: “Crescit ad marginem stagnorum juxta Adoua, mense Novembre (Quartin Dillon).” Ethiopia: Adoua; *Quartin Dillon s.n.* (P).

*Arthraxon quartinianus* (A. Rich.) Nash, N. Amer. Fl. 17(2): 99. 1912

*Arthraxon breviaristatus* Hack., Monogr. Phan. [A. DC. & C. DC.] 6: 350. 1889. Syntypes: China: Yunnan, prope Ki-mi-se, ad rivulos montium; *Delavay 1811*(P). India: Khasia, alt. 4000–6000 ft.; *J.D. Hooker & T. Thomson s.n.* (K, L, W).

*Arthraxon ciliaris* P. Beauv., Ess. Agrostogr. 111. t. 11. f. 6. 1812 (as “ciliare”). Type: “Cultivé par son oncle a Trianon”; *Richard s.n.* (P).

*Arthraxon ciliaris* P. Beauv. var. *hookeri* Hack., Monogr. Phan. [A. DC. & C. DC.] 6: 357. 1889. Type: India: Sikkim; *J.D. Hooker & Thomson s.n.*

*Arthraxon inermis* Hook. f. var. *tzvelevii* Jain, Sci. & Cult. 37(1): 55. 1971. Type: India: Purandhar, vazirghad Fort; *Santapau 11361* (CAL)

*Batratherum micans* Nees, Edinburgh New Philos. J. 18: 182–183. 1835. Type: “In Benghala boreli; Royle.”

*Arthraxon micans* (Nees) Hochst., Flora 39: 188. 1856

*Arthraxon nitidulus* Stapf ex Bor, Grasses Burma, Ceylon, India and Pakistan 101, 688. 1960. Type: India: Bombay, Belgaum; *Ritchie 796a* (K).

*Arthraxon satarensis* M. R. Almeida, J. Bombay Nat. Hist. Soc. 66: 515. 1970, emend. Deshp. & Hemadri, Bull. Bot. Surv. India 12: 274. 1970. Type: India: Maharashtra, 8-Satara-Keshyaturda; *McCann 8* (BLAT).

**Distribution.** Andhra Pradesh, Bihar, Karnatata, Madhya Pradesh, Maharashtra, Tamil Nadu, Sikkim, Uttar Pradesh, West Bengal [Bhutan, China, Nepal, Pakistan, Sri Lanka].

**Habit.** Annual.

**811. **Arthraxon hispidus** (Thunb.) Makino var. **junnarensis** (S.K. Jain & Hemadri) Welzen, Blumea 27(1): 277. 1981 **

*Arthraxon junnarensis* S.K. Jain & Hemadri, J. Bombay Nat. Hist. Soc. 68: 300–301. f. a–f. 1971. Type: India: Maharashtra, Poona District, Warsubai plateau, 16km west of Junnar; *Hemadri 106849* (CAL).

**Distribution.** Maharashtra [China].

**Habit.** Annual.

**Remarks.** Record for India fide Naithani (1990).

**812. **Arthraxon inermis** Hook. f., Fl. Brit. India (J.D. Hooker). 7(21): 145. 1896 **

**Type.** India: Concan; *Woodrow 189* (K).

**Distribution.** Goa, Maharashtra.

**Habit.** Annual.

**Remarks.** Record for Maharashtra fide Naithani (1990).

**813. **Arthraxon jubatus** Hack., Monogr. Phan. [A. DC. & C. DC.] 6: 358. 1889 **

**Type.** India: Kerala, Concan, Malabar; *J.E. Stocks s.n.* (W).

**Distribution. **Kerala, Karnataka, Maharashtra.

**Habit.** Annual.

**814. **Arthraxon lanceolatus** (Roxb.) Hochst., Flora 39: 188. 1856 var. **lanceolatus

*Andropogon lanceolatus* Roxb., Fl. Ind. (Carey and Wallich ed.) 1: 262. 1820. Type: India: “A native of Coromendel.” Lectotype: India; *Roxburgh Icon. Ined. 2019* (K, copy in L). LT designated by van P.C. van Welzen in Blumea 27(1): 283. 1981.

*Arthraxon deccanensis* S.K. Jain, J. Bombay Nat. Hist. Soc. 68: 298. 1971. Type: India: Maharashtra, Poona District, Sinhagad; *Patil 7825* (CAL).

**Distribution.** Andhra Pradesh, Assam, Bihar, Chhattisgarh, Daman and Diu, Gujarat, Jharkhad, Karnataka, Kerala, Maharashtra, Odisha, Punjab, Tamil Nadu [China, Pakistan].

**Habit.** Annual.

**Remarks.** Record for Maharashtra fide Naithani (1990).

**815. **Arthraxon lanceolatus** (Roxb.) Hochst. var. **echinatus** (Nees) Hack., Monogr. Phan. [A. DC. & C. DC.] 6: 348. 1889 **

*Batratherum echinatum* Nees, Edinburgh New Philos. J. 18: 181. 1835. Lectotype: India; *Wight KD 1684* (K). LT designated by P.C. van Welzen in Blumea 27(1): 285. 1981.

*Arthraxon echinatus* (Nees) Hochst., Flora 39: 188. 1856.

*Arthraxon spathaceus* Hook. f., Fl. Brit. India (J.D. Hooker). 7(21): 145. 1896. Type: India: Deccan Peninsula, Gunda Cottah Hill fort, Cuddapah; *Wight Herb. 337* (K).

**Distribution.** Andhra Pradesh, Assam, Karnataka, Maharashtra, Odisha, Tamil Nadu, Uttar Pradesh [Nepal].

**Habit.** Annual.

**816. **Arthraxon lancifolius** (Trin.) Hochst., Flora 39: 188. 1856 **

*Andropogon lancifolius* Trin., Mém. Acad. Imp. Sci. St.-Pétersbourg, Sér. 6, Sci. Math. 2(4): 271. 1832. Type: Nepal; Numer. List [Wallich] no. 8828B (LE).

*Pleuroplitis lancifolia* (Trin.) Regel, Bull. Acad. Imp. Sci. Saint-Petersbourg. 10(3): 375. 1866

*Arthraxon lancifolius* (Trin.) Hochst. var. *hindustanicus* S.K. Jain & Deshp., J. Indian Bot. Soc. 51: 176. 1972. Type: India: Goa, near Chopora, Kaisuva Fort; September 5, 1965; *Cherian 88557A* (CAL).

*Andropogon multicaulis* Steud., Syn. Pl. Glumac. 1: 383. 1854. Type: Abyssin; *Hochstetter s.n.*

*Arthraxon microphyllus* (Trin.) Hochst. var. *hindustanicus* (S.K. Jain & Deshp.) S.M. Almeida & M. R. Almeida, J. Bombay Nat. Hist. Soc. 82: 445. 1985

*Batratherum molle* Nees, Edinburgh New Philos. J. 18: 181–182. 1835. Type: Type information given for varieties only

*Arthraxon molle* (Nees) Balf. f., Trans. Roy. Soc. Edinburgh 31: 315. 1888

*Psilopogon schimperi* Hochst. ex A. Rich., Tent. Fl. Abyss. 2: 447. 1850–1851. Type: “Crescit in Planitie montosa Beless, provinciae Chire, mense Octobre (Quartin Dillon) et in locis umbrosis ad declivia rivulorum juxta Adoua, mense Septembre (Schimper).” Syntypes: Ethiopia: Adoua; September 18, 1837; *Schimper 96 *(TUB?). ST: Ethiopia: Schire; *Quartin Dillon s.n.* (P). Ethiopia: Tigray, Adua; September 18, 1837; *Schimper 46 *(WAG).

*Batratherum schimperi* (Hochst. ex A. Rich.) Nees ex Hochst., Flora 39: 179. 1856 (as “Bathratherum”)

*Arthraxon schimperi* (Hochst. ex A. Rich.) Hochst., Flora 39: 189. 1856, p.p.

*Lucaea schimperi* (Hochst. ex A. Rich.) Steud., Syn. Pl. Glumac. 1: 414. 1855.

*Pleuroplitis schimperi* (Hochst. ex A. Rich.) Regel, Bull. Acad. Imp. Sci. Saint-Petersbourg. 10(3): 369. 1866

*Arthraxon microphyllus* sensu Hook. f., Fl. Brit. India (J.D. Hooker). 7(21): 147. 1896, p.p., non (Trin.) Hochst. 1856

*Pleuroplitis microphylla* (Trin.) Regel, Bull. Acad. Imp. Sci. Saint-Petersbourg. 10(3): 370. 1866

*Arthraxon minor* Hochst., Flora 39: 188. 1856, nom. illeg. & superfl.

**Distribution.** Andhra Pradesh, Bihar, Chhattisgarh, Dadra and Nagar Haveli, Daman and Diu, Goa, Gujarat, Himachal Pradesh, Jammu and Kashmir, Karnataka, Kerala, Maharashtra, Madhya Pradesh, Odisha, Rajasthan, Sikkim, Tamil Nadu, Uttar Pradesh, Uttarakhand [China, Myanmar, Nepal, Pakistan, Sri Lanka].

**Habit.** Annual.

**817. **Arthraxon meeboldii** Stapf, Bull. Misc. Inform. Kew 1908: 449. 1908 **

*Arthraxon lanceolatus* (Roxb.) Hochst. var. *meeboldii* (Stapf) Welzen, Blumea 27(1): 285, f. 10. 1981

*Arthraxon purandharensis* Bharucha & Y. Satyan., J. Bombay Nat. Hist. Soc. 52: 481. 1954. Type: India: Bombay, Purandhar, 4000 ft.; *Bharucha 501* (Herb. Inst. Sc. Bombay).

**Type.** India: Concan, in open grassland on a hillside near Khandale, alt. 2000 ft.; *Meebold 9132.*

**Distribution.** Gujarat, Jammu and Kashmir, Karnataka, Madhya Pradesh, Maharashtra.

**Habit.** Annual.

**Remarks.** All records except for Madhya Pradesh fide Naithani (1990).

**818. **Arthraxon microphyllus** (Trin.) Hochst., Flora 39: 188. 1856 **

*Andropogon microphyllus* Trin. Mém. Acad. Imp. Sci. St.-Pétersbourg, Sér. 6, Sci. Math. 2: 275. 1832. Type: Nepal; *Wallich s.n.* (LE).

*Arthraxon sikkimensis* Bor, Kew Bull. 1951: 447. 1952. Type: India: Sikkim, Lachung, alt. 9000 ft.; August 31, 1892; *Gammie 1079* (K).

**Distribution.** Gujarat, Jammu and Kashmir, Maharashtra, Meghalaya, Sikkim, Tamil Nadu [Bhutan, China, Myanmar, Nepal, Sri Lanka].

**Habit.** Annual.

**819. **Arthraxon nudus** (Steud.) Hochst., Flora 39: 188. 1856 **

*Andropogon nudus* Steud., Syn. Pl. Glumac. 1: 383. 1854. Type: India: Tavoy; Numer. List [Wallich] no. 8834A

*Arthraxon ciliaris* P. Beauv. ssp. *nudus* (Steud.) Hack., Monogr. Phan. 6: 356. 1889.

**Distribution.** Assam, Kerala, Uttar Pradesh, Uttarakhand [China, Myanmar].

**Habit.** Annual.

**820. **Arthraxon prionodes** (Steud.) Dandy in F. W. Andrews, Fl. Pl. Sudan 3: 399. 1956 **

*Andropogon prionodes* Steud., Syn. Pl. Glumac. 1: 383. 1854. Type: Ethiopia; *Schimper 1117*(P). LT designated by P.C. van Welzen in Blumea 27: 283. 1981.

**Distribution.** Andhra Pradesh, Chhattisgarh, Himachal Pradesh, Gujarat, Karnataka, Madhya Pradesh, Uttarakhand [China, Myanmar, Nepal, Pakistan].

**Habit.** Perennial.

**821. **Arthraxon raizadae** S.K. Jain, Hemadri & Deshp., J. Indian Bot. Soc. 51: 103. 1972 **

*Arthraxon lanceolatus* (Roxb.) Hochst. var. *raizadae* (S.K. Jain, Hemadri & Deshp.) Welzen, Blumea 27: 287. 1981

**Type.** India: Maharashtra State, Satara District, Mahabaleshwar; December 25, 1969; *Hemadri 98585A* (CAL).

**Distribution.** Karnataka, Maharashtra.

**Habit.** Annual.

**Remarks.** Record for Karnataka fide Naithani (1990).

**822. **Arthraxon santapaui** Bor, Kew Bull. 1951: 446. 1952 **

*Arthraxon hispidus* (Thunb.) Makino var. *santapaui* (Bor) Welzen, Blumea 27(1): 280. f. 6. 1981

**Type.** India: Bombay, Purandhar Fort; October 10, 1950; *H. Santapau 11450a* (K).

**Distribution.** Himachal Pradesh, Maharashtra, Uttarakhand [also in Oman].

**Habit.** Annual.

**Remarks.** Record for Maharashtra fide Naithani (1990).

**823. **Arthraxon submuticus** (Nees ex Steud.) Hochst., Flora 39: 188. 1856 **

*Andropogon submuticus* Nees ex Steud., Syn. Pl. Glumac. 1: 382. 1854. Type: Nepal; Numer. List [Wallich] no. 8836

**Distribution.** Uttar Pradesh, Uttarakhand [China, Nepal].

**Habit.** Annual.

**824. **Arthraxon villosus** C.E.C. Fisch., Bull. Misc. Inform. Kew 1933: 350. 1933 **

*Arthraxon lanceolatus* (Roxb.) Hochst. var. *villosus *(C.E.C. Fisch.) Welzen, Blumea 27(1): 288. 1981

**Type.** India: Western Ghats, Bababudans; April; *Bourne & Bourne s.n.* (K).

**Distribution.** Karnataka, Kerala, Maharashtra, Tamil Nadu.

**Habit.** Annual.

**Remarks.** Records for Maharashtra and Tamil Nadu fide Naithani (1990).

***Arundinella* Raddi, Agrostogr. Bras. [Raddi] 36. t. 1. f. 3. 1823**

**Type. ***Arundinella brasiliensis* Raddi.

**825. **Arundinella bengalensis** (Spreng.) Druce, Rep. Bot. Exch. Club Soc. Brit. Isles 4: 605. 1917 **

*Panicum bengalense* Spreng., Syst. Veg. (ed. 16) [Sprengel] 1: 311. 1824 (“1825”). Type: A native of Bengal, where it is found, though rarely, on dry barren spots.

*Arundinella wallichii* Nees, Syn. Pl. Glumac. (Steudel) 1: 114. 1854. Type: Nepal; Numer. List [Wallich] no. 8669

*Oplismenus strictus* Schult., Mant. 2: 272. 1824.

*Arundinella stricta* (Schult.) Janowski, Repert. Spec. Nov. Regni Veg. 17: 84. 1921, non Nees, 1850

*Panicum strictum* Roxb., Fl. Ind. (Carey and Wallich ed.) 1: 306. 1820, non R. Br., 1810

**Distribution.** Assam, Bihar, Chhattisgarh, Madhya Pradesh, Meghalaya, Tripura, Uttar Pradesh, Uttarakhand, West Bengal [Bangladesh, Bhutan, China, Myanmar, Nepal].

**Habit.** Perennial.

**826. **Arundinella cannanorica** V.J. Nair, Sreek. & N.C. Nair, J. Bombay Nat. Hist. Soc. 80(2): 396–398. f. 11. 1984 **

**Type.** India: Kerala, Cannanore District, Bela, alt. 320 ft.; November 23, 1981; *P.V. Shreekumar 71822* (CAL).

**Distribution.** Kerala.

**Habit.** Annual.

**827. **Arundinella ciliata** (Roxb.) Nees ex Miq., Nieuwe Verh. Eerste Kl. Kon. Ned. Inst. Wetensch. Amsterdam 4: 30. 1851 **

*Holcus ciliatus* Roxb., Fl. Ind. (Carey and Wallich ed.) 1: 321. 1820. Type: India: “A native of Coromendel.”

*Arundinella agrostoides* Hook. f. var. *ciliata* (Roxb.) Hook. f., Fl. Brit. India (J.D. Hooker). 7(21): 71. 1896

*Arundinella pilosa* Hochst. ex Miq., Nieuwe Verh. Eerste Kl. Kon. Ned. Inst. Wetensch. Amsterdam. 3, Pt. 4: 30. 1851, nom. invalid as syn of Arundinella ciliata. Type: India: “Mercara Ind. Or. Hochst. Hbrb. Hohenack. 647 in Sched.”

*Arundinella agrostoides* sensu Hook. f., Fl. Brit. India (J.D. Hooker). 7(21): 71. 1896, non Trin., 1836

**Syntypes.** India; *Wight s.n.* Prope Mercara; *Metz s.n.*

**Distribution.** Andhra Pradesh, Chhattisgarh, Dadra and Nagar Haveli, Karnataka, Kerala, Madhya Pradesh, Maharashtra, Tamil Nadu.

**Habit.** Annual.

**828. **Arundinella decempedalis** (Kuntze) Janowski, Repert. Spec. Nov. Regni Veg. 17: 84. 1921 **

*Panicum decempedale* Kuntze, Revis. Gen. Pl. 2: 783. 1891. Type: Sikkim Himalaya, Terai; January 15, 1875; *Kuntze 6581*

*Arundinella clarkei* Hook. f., Fl. Brit. India (J.D. Hooker). 7(21): 75–76. 1896. Type: India: Sikkim, Terai; *C.B. Clarke s.n.*

**Distribution.** Assam, Sikkim, West Bengal [China, Myanmar].

**Habit.** Annual.

**829. **Arundinella hirta** (Thunb.) Tanaka, Bult. Sci. Fak. Terk. Kjusu Imp. Univ. 1: 196, 208. 1925 **

*Poa hirta* Thunb., in J.A. Murray, Syst. Veg. ed. 14: 104. 1784. Type: Japonia.

*Arundinella anomala* Steud., Syn. Pl. Gl. 1:116. 1854. Type: Japan; *Anonymous s.n.* [Siebold].

**Distribution.** Assam.

**Habit. **Perennial.

**Remarks.** Record for Assam fide Naithani (1990).

**830. **Arundinella holcoides** (Kunth) Trin., Bull. Sci. Acad. Imp. Sci. Saint-Pétersbourg 1: 71. 1836. **

*Brandtia holcoides* Kunth, Révis. Gramin. 2: 511. 1831. Type: Burma. Crescit in Pegu, *M. Reynaud s.n*.

*Arundinella pubescens* Merr. & Hack., Philipp. J. Sci., C 2: 419. 1907. Philippine Islands: Palawan: May 1906, F.W. Foxworthy BS 856

*Arundinella birmanica* Hook. f., Fl. Brit. India 7: 73. 1896.

*Arundinella hirsuta* Nees, Syn. Pl. Glumac. (Steudel) 1: 115. 1854 Type: Himalaya: Coutrallum; Numer. List [Wallich] no. 8671

**Distribution. **Karnataka, Maharashtra, Himalaya.

**Habit.** Annual.

**Remarks.** The circumscription and synonymy of this species are unclear and need to be carefully re-evaluated.

**831. **Arundinella hookeri** Munro ex Keng, Sci. Rep. Natl. Centr. Univ., Ser. B 2(3): 50. 1936 **

*Arundinella villosa* Arn. ex Steud. var. *himalaica* Hook. f., Fl. Brit. India (J.D. Hooker). 7(21): 73. 1896. Syntypes: Bhutan, East Nepal and Sikkim Himalaya, alt. 6000–11000 ft.; *Griffith s.n., J.D. Hooker & C.B. Clarke s.n.*

**Type.** India: South of Yonsum, Sikkim Himalaya; 1888; *King’s collector s.n.* Pulney Hills; *Madras Herb 16526.*

**Distribution.** Sikkim, Tamil Nadu, West Bengal [Bhutan, China, Myanmar, Nepal].

**Habit.** Perennial.

**832. **Arundinella intricata** Hughes, Bull. Misc. Inform. Kew 1920: 112. 1920 **

**Syntypes.** India: Khasia Hills, Maosmai, alt. 4000 ft.; *C.B. Clarke 16588* (K). Mahadeo, alt. 3000 ft.; *Clarke 155622* (K). India: Khasia Hills, Amwé, 4000–4700 ft.; *J.D. Hooker s.n.* (K). Boga-Panee, 4000–6000 ft.; *J.D. Hooker s.n. *(K). India: Arbor Country, Dihang River, bank near Ritung; *I. J. Burkill 373* (K).

**Distribution.** Arunachal Pradesh, Assam, Karnataka, Meghalaya [Bhutan, China]

**Habit.** Perennial.

**Remarks.** Record for Arunachal Pradesh, Assam, and Meghalaya fide Naithani (1990).

**833. **Arundinella khaseana** Nees, Syn. Pl. Glumac. (Steudel) 1: 115. 1854 **

**Type.** India: Jugum Khasea; Numer. List [Wallich] no. 8672.

**Distribution.** Assam, Meghalaya [China, Myanmar].

**Habit.** Perennial.

**834. **Arundinella leptochloa** (Nees) Hook. f., Fl. Brit. India (J.D. Hooker). 7(21): 76. 1896 **

*Panicum leptochloa* Nees, Syn. Pl. Glumac. (Steudel) 1: 62. 1853. Type: India orientalis; *Wight Herb. no. 125*

*Arundinella gigantea* Dalzell in Dalzell & A. Gibson, Bombay Fl. 293. 1861. Type: India: Ghauts, Kineshwur; *Dalzell s.n.*

*Arundinella lawsonii* Hook. f., Fl. Brit. India (J.D. Hooker). 7(21): 76. 1896 (as “lawsoni”). Type: India: Tamil Nadu, Nilghiri Hills, Goodaloor, alt. 3000 ft.; *Lawson s.n.*

**Distribution.** Dadra and Nagar Haveli, Goa, Gujarat, Karnataka, Kerala, Maharashtra, Rajasthan, Tamil Nadu [Sri Lanka].

**Habit.** Perennial.

**835. **Arundinella mesophylla** Nees, Syn. Pl. Glumac. (Steudel) 1: 115. 1854 **

**Type.** India: Courtallum; Numer. List [Wallich] no. 8663.

**Distribution.** Karnataka, Kerala, Tamil Nadu.

**Habit.** Annual.

**836. **Arundinella metzii** Hochst. ex Miq., Nieuwe Verh. Eerste Kl. Kon. Ned. Inst. Wetensch. Amsterdam ser. 3, 4: 31. 1851 **

*Arundinella decomposita* Janowski, Repert. Spec. Nov. Regni Veg. 17: 84. 1921. Type: India; *Meebold 10760*

*Arundinella lawii* Hook. f. in Trimen, Handb. Fl. Ceylon 5: 180. 1900. Syntypes: Sri Lanka: without precise locality; Ferguson in Herb. Peraden. India: Concan, Malabar; *J.E. Stocks s.n.*

*Arundinella pygmaea* Hook. f., Fl. Brit. India (J.D. Hooker). 7(21): 72. 1896. Type: India: North Canara; *Lisboa s.n.*

**Type.** India: Karnataka, Mangalore; *Metz Herb. no. 297.*

**Distribution.** Goa, Karnataka, Kerala, Tamil Nadu [Sri Lanka].

**Habit.** Annual.

**837. **Arundinella muthikulamensis** Sunil, K.M.P. Kumar, & V.P. Thomas, Webbia 72(1): 101. 2017. **

**Type.** India: Kerala, Palghat District, Muthikulam, Elivalmala, 2100 m, 11 November 2016, *Sunil& K. M. Prabhukumar 4565* (holotype: CMPR; isotypes: MH, SNMH, CATH).

**Distribution.** Kerala.

**Habit.** Perennial.

**838. **Arundinella nepalensis** Trin., Gram. Panic. [Trinius] 62. 1826 **

*Acratherum miliaceum* Link, Hort. Berol. [Link] 1: 230. 1827. Type: “Habitat in Nepalia.”

*Arundinella miliacea* (Link) Nees, Hooker’s J. Bot. Kew Gard. Misc. 2: 102. 1890

*Arundinella glabra* Hook. & Arn., Bot. Beechey Voy. 5: 237. 1837. Type: “Hab. Prope Macao”;* G.H. Vachell s.n. *(E?).

*Arundinella ritchei* Munro ex Lisboa, J. Bombay Nat. Hist. Soc. 5: 343. 1890. Type: India: Maharashtra, Common in Thana, Salsette and Lanowli; *Munro s.n.* (Herbarium of Poona College of Science).

*Arundinella virgata* Janowski, Repert. Spec. Nov. Regni Veg. 17: 84–85. 1921. Type: China: South China; 1865; *W. Hillebrand s.n. *(B).

**Type.** Nepal; *Wallich s.n. in Herb. Lindley* (LE).

**Distribution.** Andhra Pradesh, Arunachal Pradesh, Assam, Bihar, Himachal Pradesh, Karnataka, Kerala, Maharashtra, Meghalaya, Nagaland, Sikkim, Tamil Nadu, Uttar Pradesh, Uttarakhand, West Bengal [Bhutan, China, Nepal, Pakistan].

**Habit. **Perennial.

**839. **Arundinella nervosa** (Roxb.) Nees ex Hook. & Arn., Bot. Beechey Voy. 5: 237. 1837 **

*Holcus nervosus* Roxb., Fl. Ind. (Carey and Wallich ed.) 1: 320. 1820. Type: India: A native of Coromandel.

**Distribution.** Goa, Himachal Pradesh, Karnataka, Kerala, Maharashtra, Tamil Nadu.

**Habit.** Annual.

**840. **Arundinella pradeepiana** Sunil & Naveen Kum., Webbia 69(2): 249. 2014. **

**Type.** India: Kerala, Eranakulam district, Edamalayar forest range, Variyam, 800 m, 16 November 2013, *Sunil & Naveen Kumar 6228* (holotype: CAL; isotype: MH, CALI).

**Distribution.** Kerala.

**Habit. **Annual.

**841. **Arundinella pumila** (Hochst. ex A. Rich.) Steud., Syn. Pl. Glumac. 1: 114. 1854 **

*Acratherum pumilum* Hochst. ex A. Rich., Tent. Fl. Abyss. 2: 414. t. 100. 1850–1851. Type: “Crescit in pratis circa Aderbati in regno Tigre, mense Septembre (Quartin Dillon). Lectotype: Ethiopia: “in vallibus prope Adde Arbati”; October, 1839; *Schimper 642* (B). LT cited by van der Zon, Gram. Cameroun 2: 361. 1992.

*Arundinella tenella* Nees, Syn. Pl. Glumac. (Steudel) 1: 115. 1854. Type: India; *Wight herb. no.76, Royle herb. no. 268*

**Distribution.** Andhra Pradesh, Assam, Bihar, Chhattisgarh, Dadra and Nagar Haveli, Goa, Gujarat, Karnataka, Kerala, Madhya Pradesh, Maharashtra, Meghalaya, Odisha, Rajasthan, Tamil Nadu, Uttar Pradesh, Uttarakhand, West Bengal [Myanmar, Nepal, Sri Lanka].

**Habit.** Annual.

**842. **Arundinella purpurea** Hochst. ex Steud., Syn. Pl. Glumac. 1: 115. 1854 var. **purpurea

*Arundinella fuscata* sensu Hook. f., Fl. Brit. India (J.D. Hooker). 7(21): 74. 1896, non Nees ex Buese, 1854

**Type.** India: Tamil Nadu, Nilagiri Mountain; *Hohenacker 928* (P).

**Distribution.** Andhra Pradesh, Karnataka, Kerala, Maharashtra, Tamil Nadu.

**Habit.** Perennial.

**843. **Arundinella purpurea** Hochst. ex Steud. var. **laxa** Bor, Kew Bull. 1955: 407. 1955 **

**Type.** India: Madras, Sispara, alt. 6700 ft.; *J.S. Gamble 13365.*

**Distribution.** Karnataka, Kerala, Tamil Nadu.

**Habit.** Perennial.

**Remarks.** Record for Kerala fide Naithani (1990).

**844. **Arundinella ravii** Shaju & N. Mohanan, Rheedea 14(1–2): 47–50. f. 1. 2004 **

**Type.** India: Kerala, Idukki District, Eravikulam National Park, 6000 ft.; November 8, 2000; *Shaju 43700* (TBGT).

**Distribution.** Kerala.

**Habit.** Annual.

**845. **Arundinella setosa** Trin., Gram. Panic. [Trinius] 63. 1826 var. **setosa

*Arundinella bidentata* Keng, J. Wash. Acad. Sci. 21(8): 159. 1931. Type: China: Fujian, Kuliang, moist hillsides and open lands, alt. 2500 ft.; July 30, 1919; *J.B. Norton 1154* (US-1270758).

*Arundinella capillaris* Hook. f., Fl. Brit. India (J.D. Hooker). 7(21): 74. 1896. Type: India: Deccan; *Heyne s.n. *Pulney Hills;* Herb. Wight no. 3338* Amsterdam. 3, Pt. 4: 30. 1851]. Type: Himalaya: Coutrallum; Numer. List [Wallich] no. 8671

*Arundinella mutica* Nees, Syn. Pl. Glumac. (Steudel) 1: 116. 1854. Type: India; *Heyne s.n.* (Numer. List [Wallich] no. 8665)

*Arundinella setifera* Steud., Syn. Pl. Glumac. 1: 115. 1854. Type: India: Nilagherry Mountains; *Hohenacker 920*

*Arundinella sinensis* Rendle, J. Linn. Soc., Bot. 36: 342. 1904. Type: China: Kwangtung; *C. Ford 212, C. Ford 201* (BM). China: Guangdong, Lofaushan; *Ford 125*

*Arundinella stricta* Nees, Hooker’s J. Bot. Kew Gard. Misc. 2: 102. 1850

*Arundinella zollingeri* Steud., Syn. Pl. Glumac. 1: 115. 1854. Type: Java;* Zollinger 2034*

*Miquelia barbulata* Nees, Nov. Actorum Acad. Caes. Leop.-Carol. Nat. Cur., Suppl. 19(1): 178. 1843. Type: “In Promontorio Syng-moon.”Hong Kong; Julio; *F.J.F. Meyen s.n.*

*Berghausia barbulata* (Nees) Endl. ex Miq., Nieuwe Verh. Eerste Kl. Kon. Ned. Inst. Wetensch. Amsterdam ser. 3, 4: 32. 1851

*Danthonia luzoniensis* Steud., Syn. Pl. Glumac. 1: 245. 1854. Type: Philippines: Luzon; *Cuming herb. no. 1415*

*Danthonia neuroelytrum* Steud., Syn. Pl. Glumac. 1: 245. 1854. Type: China; *Fortune Herb. No. 5* (P).

*Milium cimicinoides* Roxb. ex Hook. f., Fl. Brit. India (J.D. Hooker). 7(21): 70. 1896, nom. nud.

**Type.** Nepal; *Lindley s.n. *(LE).

**Distribution.** Andhra Pradesh, Assam, Arunachal Pradesh, Bihar, Dadra and Nagar Haveli, Gujarat, Himachal Pradesh, Karnataka, Kerala, Maharashtra, Meghalaya, Nagaland, Odisha, Tamil Nadu, Uttar Pradesh, West Bengal [Bhutan, China, Myanmar, Nepal, Sri Lanka].

**Habit.** Perennial.

**846. **Arundinella setosa** Trin. var. **esetosa** Bor, Grasses Burma, Ceylon, India & Pakistan 425. 1960 **

**Syntypes.** India: Bihar; *T. Thomson s.n.* Nepal: Western part; *Stainton, Sykes & Williams 4255, 4461.*

**Distribution.** Andhra Pradesh, Bihar, Tamil Nadu, Uttar Pradesh [China, Myanmar, Nepal].

**Habit.** Perennial.

**Remarks.** Records for Bihar and Tamil Nadu fide Naithani (1990).

**847. **Arundinella setosa** Trin. var. **lanifera** C.E.C. Fisch., Fl. Madras 3(10): 1801. 1934 **

**Type.** India: Cuddapa District, Mogili Kuppa, alt. 6000 ft.; *Meebold s.n.*

**Distribution.** Andhra Pradesh, Arunachal Pradesh, Karnataka, Madhya Pradesh, Tamil Nadu, Uttar Pradesh.

**Habit.** Perennial.

**Remarks.** Records for Andhra Pradesh and Madhya Pradesh fide Naithani (1990).

**848. **Arundinella setosa** Trin. var. **nilagiriana** Subba Rao & Kumari, J. Bombay Nat. Hist. Soc. 72: 827. 1976 **

**Type.** India: Tamil Nadu, Nilgiri District, Koilbetta; September 11, 1970; *G.V. Subba Rao & G.R. Kumari 19795A *(CAL).

**Distribution.** Andhra Pradesh, Arunachal Pradesh, Karnataka, Madhya Pradesh, Tamil Nadu, Uttar Pradesh.

**Habit.** Perennial.

**Remarks.** Record for Tamil Nadu fide Naithani (1990).

**849. **Arundinella spicata** Dalzell in Dalzell & A. Gibson, Bombay Fl. 293. 1861 **

**Type.** India: Maharashtra, Mahableshwur Hills; *Dalzell s.n.*

**Distribution.** Karnataka, Maharashtra, Rajasthan.

**Habit.** Annual.

**850. **Arundinella thirunelliensis** Sunil, Ratheesh, & Sivad., Bangladesh J. Pl. Taxon. 21(2): 153. 2014. **

**Type.** India: Kerala, Wayanad District, Thirunelli, Kalindi riverbed, 900 m, 8 December 2012, *Sunil & Ratheesh Narayanan 4854* (Holotype: CAL; Isotypes: MH).

**Distribution.** Kerala.

**Habit.** Perennial.

**851. **Arundinella tuberculata** Munro ex Lisboa, J. Bombay Nat. Hist. Soc. 5: 344. 1890 **

**Type.** India: Poona and Konkan; *Munro s.n.* (Herbarium of Poona College of Science).

**Distribution.** Bihar, Goa, Gujarat, Karnataka, Kerala, Maharashtra, Tamil Nadu.

**Habit.** Annual.

**852. **Arundinella vaginata** Bor, J. Indian Bot. Soc. 27: 66. 1948 **

*Arundinella villosa* Arn. ex Steud. var. *heynei* Hook. f., Fl. Brit. India (J.D. Hooker). 7(21): 73. 1896 (as “heyne”). Type: India; *Heyne s.n.* (Numer. List [Wallich] no. 8663A). Courtallam; *Wight s.n.*

**Type.** India: Madras, Palni Hills; *Rev. A. Sauliere 817* (K).

**Distribution.** Kerala, Tamil Nadu.

**Habit.** Perennial.

**853. **Arundinella villosa** Arn. ex Steud., Syn. Pl. Glumac. 1: 115. 1854 **

**Type.** Sri Lanka; Wight 2037.

**Distribution.** Karnataka, Meghalaya, Tamil Nadu [Sri Lanka].

**Habit.** Perennial.

***Bhidea* Stapf ex Bor, Kew Bull. 1948: 445. 1949**

**Type. ***Bhidea burnsiana* Bor.

**854. **Bhidea borii** Deshp., V. Prakash & N.P. Singh, Curr. Sci. 58(19): 1094. 1989 **

**Distribution.** India.

**Habit.** Annual.

**Remarks.** Distribution within India not recorded.

**855. **Bhidea burnsiana** Bor, Kew Bull. 1948: 445. 1949 **

**Type.** India: Bombay Lonavla; August, 1918; *R.K. Bhide s.n. *(K).

**Distribution.** Karnataka, Kerala, Maharashtra.

**Habit.** Annual.

**Remarks.** Records for Karnataka and Maharashtra fide Naithani (1990).

**856. **Bhidea fischeri** Sreek. & B. V. Shetty, Kew Bull. 42(3): 683. 1987 **

**Type.** India: Kerala, Cannanore District, Mukkarikandam, alt. 160 ft.; October 18, 1981; *P.V. Sreekumar 71754* (CAL).

**Distribution.** Kerala, Maharashtra.

**Habit.** Annual.

***Bothriochloa* Kuntze, Revis. Gen. Pl. 2: 762. 1891**

**Type. ***Bothriochloa anamitica* Kuntze.

**857. **Bothriochloa bladhii** (Retz.) S.T. Blake, Proc. Roy. Soc. Queensland 80: 62. 1969 **

*Andropogon bladhii* Retz., Observ. Bot. (Retzius) 2: 27. 1781. Type: “In China lectum cum aliis mislit bonorat, D. Bladh.” China; *Bladh s.n. *(LD).

*Dichanthium bladhii* (Retz.) Clayton, Kew Bull. 32(1): 3. 1977

*Andropogon intermedius* R. Br., Prodr. Fl. Nov. Holland. 1: 202. 1810. Type: Australia: “Keppel Bay” (North West end of Curtis Island); *Brown 6184* (BM).

*Amphilophis intermedia* (R. Br.) Stapf, Agric. News (Barbados) 15(368): 179. 1916

*Bothriochloa intermedia* (R. Br.) A. Camus, Ann. Soc. Linn. Lyon ser. 2, 76: 164. var. *Intermedia*

*Andropogon caucasicus* Trin., Mém. Acad. Imp. Sci. St.-Pétersbourg, Sér. 6, Sci. Math. 2(3): 286. 1832. Type: “V. spp. e Cauc. orient.”

*Dichanthium caucasicum* (Trin.) S.K. Jain & Deshp., Bull. Bot. Surv. India 20(1–4): 133. 1979 (“1978”)

*Sorghum caucasicum* (Trin.) Griseb., Fl. Ross. [Ledebour] 4: 476. 1853

*Bothriochloa caucasica* (Trin.) C.E. Hubb. Bull. Misc. Inform. Kew 1939: 101. 1939

*Andropogon intermedius* R. Br. var. *caucasicus* (Trin.) Hack., Monogr. Phan. [A. DC. & C. DC.] 6: 486. 1889

*Andropogon glaber* Roxb., Fl. Ind. (Carey and Wallich ed.) 1: 271. 1820. Type: “A native of Mountainous countries.” India: Bengal; *Roxburgh s.n. *(BM).

*Bothriochloa glabra* (Roxb.) A. Camus, Ann. Soc. Linn. Lyon ser. 2, 76: 164. 1931

*Dichanthium glabrum *(Roxb.) S.K. Jain & Deshp., Bull. Bot. Surv. India 20(1–4): 134. 1979 (“1978”)

*Amphilophis glabra* (Roxb.) Stapf, Fl. Trop. Afr. [Oliver et al.] 9: 172. 1917

*Andropogon haenkei* J. Presl in C. Presl, Reliq. Haenk. 1(4–5): 340. 1830. Type: “Habitat in Marianis, in Luzonia.” Philippines: Luzone; *Haenke s.n.* (PR).

*Andropogon leptanthus* Steud., Syn. Pl. Glumac. 1: 391. 1854. Type: India; *Cuming 1400*

*Andropogon punctatus* Roxb., Fl. Ind. (Carey and Wallich ed.) 1: 268. 1820. Type: India; “a Mountain grass.”

*Bothriochloa intermedia* (R. Br.) A. Camus var. *punctata* (Roxb.) Keng, Clav. Gen. Sp. Gram. Prim. Sin. 244. 1957

*Andropogon vachellii* Nees ex Hook. & Arn., Bot. Beechey Voy. 6: 243. 1838. Type: “Hab. in vicinia urbis macao Imperii Chinensis et in insulis adjacentibus. Millet, G.H. Vachell, n. 50” China: Macao and adjacent islands; *Millett; G.H. Vachell 50* (E).

*Andropogon odoratus* Lisboa, J. Bombay Nat. Hist. Soc. 4: 118, 123. 1889. Syntypes: India: Bombay Presidency, Thana District; *Dymock s.n.* Lanowli; October, 1890; *Lisboa s.n.* (K).

*Bothriochloa odorata* (Lisboa) A. Camus, Ann. Soc. Linn. Lyon ser. 2, 76: 165. 1931

*Amphilophis odorata* (Lisboa) A. Camus, Rev. Bot. Appl. Agric. Colon. 1: 305. 1921

*Dichanthium odoratum* (Lisboa) S.K. Jain & Deshp., Bull. Bot. Surv. India 20(1–4): 134. 1979 (“1978”)

*Andropogon perfossus* Nees & Meyen, ex Steud., Nomencl. Bot. [Steudel], ed. 2. 1: 92. 1840, nom. inval., cited as synonym of *A. punctatus* Roxb.

**Distribution.** Bihar, Chhattisgarh, Karnataka, Kerala, Madhya Pradesh, Maharashtra, Manipur, Rajasthan, Tamil Nadu, Uttar Pradesh, Uttarakhand, West Bengal [Bhutan, China, Myanmar, Nepal, Pakistan, Sri Lanka.

**Habit.** Perennial.

**858. **Bothriochloa compressa** (Hook. f.) Henrard, Blumea 3(3): 456. 1940 **

*Andropogon compressus* Hook. f., Fl. Brit. India (J.D. Hooker). 7(21): 172. 1896. Syntypes: India: Deccan; *Lisboa* (no 6. *A. odoratus*) (K). Deccan; *Woodrow s.n. *(K).

*Dichanthium compressum* (Hook. f.) S.K. Jain & Deshp., Bull. Bot. Surv. India 20(1–4): 133. 1979 (“1978”)

**Distribution.** Maharashtra, Tamil Nadu.

**Habit.** Perennial.

**859. **Bothriochloa ensiformis** (Hook. f.) Henrard, Blumea 3(3): 457. 1940 **

*Andropogon ensiformis* Hook. f., Fl. Brit. India (J.D. Hooker). 7(21): 175. 1896. Type: India: the Concan; *Dalzell s.n.*

**Distribution.** Karnataka.

**Habit.** Perennial.

**860. **Bothriochloa grahamii** (Haines) Bor, Grasses Burma, Ceylon, India & Pakistan 107. 1960 **

*Andropogon grahamii* Haines, Bull. Misc. Inform. Kew 1914: 189. 1914. Type: India: Central Provinces, Amakantak Hills, often gregarious in old jhumed lands (bewars); *Haines 3646* (K).

*Dichanthium grahamii *(Haines) Deshp., Fasc. Fl. India 15: 14. 1984

**Type.** India: Madhya Pradesh; *H.H. Haines 3646* (K).

**Distribution.** Madhya Pradesh.

**Habit.** Perennial.

**861. **Bothriochloa insculpta** (Hochst. ex A. Rich.) A. Camus, Ann. Soc. Linn. Lyon ser. 2, 76: 165. 1931 **

*Andropogon insculptus* Hochst. ex A. Rich., Tent. Fl. Abyss. 2: 458. 1850–1851. Type: “Crescit in montosis provincial Chire (Quartin Dillon), et in terra sicca ad radices montis Selleuda, prope Adoua, mense Septembre (Schimper).” Lectotype: Ethiopia: Tigray, Scholoda; September 22, 1837; *Schimper 80* (US-76201 (fragm)). LT designated (as “holotype”) by Marchi & Longhi-Wagner in Bol. Inst. Bioci. Univ. Fed. Rio Grande do Sul 57: 38. 1998.

*Amphilophis insculpta* (Hochst. ex A. Rich.) Stapf, Fl. Trop. Afr. [Oliver et al.] 9: 176. 1917

*Andropogon pertusus* (L.) Willd. var. *insculptus* (Hochst. ex A. Rich.) Hook. f., Fl. Brit. India (J.D. Hooker). 7(21): 174. 1896

*Dichanthium insculptum* (Hochst. ex A. Rich.) Clayton, Kew Bull. 32(1): 3. 1977

*Andropogon subunifoveolatus* Steud., Syn. Pl. Glumac. 1: 380. 1854, in nota. Type: India: Tamil Nadu, Nilagiri Mountains; *R.F. Honenacker no. 918*

**Distribution.** Bihar, Karnataka, Kerala, Maharashtra, Tamil Nadu [also in Africa, South America].

**Habit.** Perennial.

**862. **Bothriochloa ischaemum** (L.) Keng, Contr. Biol. Lab. Sci. Soc. China, Bot. Ser. 10: 201. 1936 **

*Andropogon ischaemum* L., Sp. Pl. 2: 1047. 1753. Type: “Habitat in Europae australioris aridis.” Lectotype: *Herb. Linn. No. 1211.26* (LINN). LT designated by Marchi & Longhi-Wagner in Bol. Inst. Bioci. Univ. Fed. Rio Grande do Sul 57: 41. 1998.

*Dichanthium ischaemum* (L.) Roberty, Boissiera 9: 160. 1960

**Distribution.** Gujarat, Jammu and Kashmir, Manipur, Punjab, Rajasthan, Madhya Pradesh, Tamil Nadu, Uttar Pradesh [Afghanistan, China, Pakistan].

**Habit.** Perennial.

**863. **Bothriochloa kuntzeana** (Hack.) Henrard, Blumea 3(3): 456. 1940 **

*Andropogon kuntzeanus* Hack., Monogr. Phan. [A. DC. & C. DC.] 6: 478. 1889. Type: Peninsular Indiae orientalis: Assirgar; *Kuntze s.n.* (NY).

*Amphilophis kuntzeana* (Hack.) Haines, Bot. Bihar Orissa 5: 1031. 1924

*Dichanthium kuntzeana* (Hack.) S.K. Jain & Deshp., Bull. Bot. Surv. India 20(1–4): 134. 1979 (“1978”)

*Eulalia clarkei* Haines, Bot. Bihar Orissa 5: 1017. 1924. Type: India: Singbhum, alt. 2500 ft.; *C.B. Clarke 20551*

**Distribution.** Andhra Pradesh, Bihar, Kerala, Madhya Pradesh, Maharashtra, Tamil Nadu, Uttar Pradesh, Uttarakhand [Pakistan].

**Habit.** Perennial.

**864. **Bothriochloa longifolia** (Hack.) Bor, Grasses Burma, Ceylon, India & Pakistan 108. 1960 **

*Andropogon pertusus* (L.) Willd. var. *longifolius* Hack., Monogr. Phan. [A. DC. & C. DC.] 6: 482. 1889. Type: India: Madras; Numer. List [Wallich] no. 8803

**Distribution.** Tamil Nadu.

**Habit.** Perennial.

**865. **Bothriochloa parameswaranii** Sreek., Malathi, & V.J. Nair, J. Bombay Nat. Hist. Soc. 85(1): 163–165. 1988 **

**Type.** India: Kerala, Idukki District, Eravikulam National Park, alt. 6800 ft.; February 14, 1981; *P.V. Sreekumar 71858 *(CAL).

**Distribution.** Kerala.

**Habit.** Perennial.

**Remarks.** Not recognized by Clayton et al. (2006 onwards).

**866. **Bothriochloa pertusa** (L.) A. Camus, Ann. Soc. Linn. Lyon ser. 2, 76: 164. 1931 **

*Holcus pertusus* L., Mant. Pl. Altera 301–302. 1771. Type: “Habitat in India orientali.” Lectotype: *Herb. Linn. No. 1212.16* (LINN). LT designated by Clayton in Kew Bull. 32: 4. 1977.

*Amphilophis pertusa* (L.) Nash ex Stapf, Agric. News (Barbados) 15(368): 179. 1916

*Andropogon pertusus* (L.) Willd., Sp. Pl., ed. 4 [Willdenow] 4: 922. 1806

*Dichanthium pertusum* (L.) Clayton, Kew Bull. 32(1): 4. 1977

*Lepeocercis pertusa* (L.) Hassk., Pl. Jav. Rar. (Hasskarl) 52. 1848

*Andropogon angustifolius* sensu Parl., Fl. Palerm. 1: 269. 1845, non Sibth. & Sm., 1806

*Elionurus pertusus* Nees ex Steud., Syn. Pl. Glumac. 1: 364. 1854, nom. inval., pro. syn.

**Distribution.** Andhra Pradesh, Assam, Bihar, Chhattisgarh, Daman and Diu, Delhi, Goa, Gujarat, Jammu and Kashmir, Karnataka, Kerala, Madhya Pradesh, Maharashtra, Odisha, Punjab, Rajasthan, Tamil Nadu, Uttar Pradesh, West Bengal [Afghanistan, China, Myanmar, Nepal, Pakistan, Sri Lanka].

**Habit.** Perennial.

**867. **Bothriochloa pseudoischaemum** (Nees) Henrard, Blumea 3(3): 457. 1940 **

*Andropogon pseudoischaemum* Nees, Syn. Pl. Glumac. (Steudel) 1: 380. 1854. Type: India orientalis; *Heyne s.n.* (Numer. List [Wallich] no. 8815)

*Dichanthium pseudoischaemum* (Nees) S.K. Jain & Deshp., Bull. Bot. Surv. India 20(1–4): 134. 1979 (“1978”)

*Amphilophis pseudoischaemum* (Nees) Alston in H. Trimen, Handb. Fl. Ceylon 6: 331. 1931 (as “*pseudischaemum*”)

**Distribution.** Andhra Pradesh, Karnataka, Kerala, Tamil Nadu [Pakistan, Sri Lanka].

**Habit.** Perennial.

**868. **Bothriochloa woodrovii** (Hook. f.) A. Camus, Ann. Soc. Linn. Lyon ser. 2, 76: 165. 1931 **

*Amphilophis woodrovii* (Hook. f.) A. Camus, Rev. Bot. Appl. Agric. Colon. 1: 305. 1921

*Andropogon woodrovii* Hook. f., Fl. Brit. India (J.D. Hooker). 7(21): 173. 1896. Type: India: Bombay, Mawal; December, 1894; *Woodrow 27* (K).

*Dichanthium woodrovii* (Hook. f.) S.K. Jain & Deshp., Bull. Bot. Surv. India 20(1–4): 134. 1979 (“1978”) (as “woodrowii”)

**Distribution.** Maharashtra.

**Habit.** Perennial.

***Capillipedium* Stapf, Fl. Trop. Afr. [Oliver et al.] 9: 11, 169. 1917**

**Lectotype. ***Capillipedium parviflorum* (R. Br.) Stapf (*Holcus parviflorus* R. Br.). LT probably designated by G. Roberty, Boissiera 9: 1–455. 1960).

**869. **Capillipedium assimile** (Steud.) A. Camus, Fl. Indo-Chine [P.H. Lecomte et al.] 7: 314. 1922 **

*Andropogon assimilis* Steud., Syn. Pl. Glumac. 1: 397. 1854. Type: Java; *Zollinger Herb. 859* (P).

*Dichanthium assimile* (Steud.) Deshp., Fasc. Fl. India 15: 6. 1984

*Andropogon glaucopsis* Steud., Syn. Pl. Glumac. 1: 397. 1854. Type: Nepal, China; Numer. List [Wallich] no. 8786

*Capillipedium glaucopsis* (Steud.) Stapf, Hooker’s Icon. Pl. 31: t. 3085. 1922

*Andropogon subrepens* Steud., Syn. Pl. Glumac. 1: 397. 1854. Type: Nepal, China; Numer. List [Wallich] no. 8790, 8789

*Capillipedium subrepens* (Steud.) Henrard, Blumea 3(3): 463. 1940

*Andropogon montanus* sensu Hack., Monogr. Phan. [A. DC. & C. DC.] 6: 490. 1889, non Roxb., 1820

**Distribution.** Andhra Pradesh, Arunachal Pradesh, Assam, Bihar, Chhattisgarh, Himachal Pradesh, Jharkhand, Karnataka, Kerala, Madhya Pradesh, Maharashtra, Manipur, Meghalaya, Nagaland, Odisha, Rajasthan, Sikkim, Tamil Nadu, Uttar Pradesh, Uttarakhand, West Bengal [Bangladesh, Bhutan, China, Myanmar, Nepal, Pakistan].

**Habit.** Perennial.

**870. **Capillipedium filiculme** (Hook. f.) Stapf, Hooker’s Icon. Pl. 31: sub t. 3085. 1922 **

*Andropogon filiculmis* Hook. f., Fl. Brit. India (J.D. Hooker). 7(21): 181. 1896. Syntypes: India: the Deccan, Poonah, in rocky places; *Jacquemont 310*. Mawar, Dhomsha; *Woodrow 26* (K).

*Dichanthium filiculme* (Hook. f.) S.K. Jain & Deshp., Bull. Bot. Surv. India 20(1–4): 134. 1979 (“1978”)

**Distribution.** Gujarat, Karnataka, Kerala, Maharashtra, Tamil Nadu.

**Habit.** Annual.

**871. **Capillipedium huegelii** (Hack.) A. Camus, Rev. Bot. Appl. Agric. Colon. 1(4): 306. 1921 **

*Andropogon huegelii* Hack., Monogr. Phan. [A. DC. & C. DC.] 6: 492. 1889 (as “hügelii). Type: Asia; *Hügel 2243* “in h. Vind. verisimiliter, ut ex num. cit. patet in India ad pedes Himalayae lectus.”

*Dichanthium huegelii* (Hack.) S.K.Jain & Deshp., Bull. Bot. Surv. India 20(1–4): 135 1979 (“1978”)

**Distribution.** Andhra Pradesh, Gujarat, Karnataka, Madhya Pradesh, Maharashtra, Rajasthan, Sikkim, Tamil Nadu, Uttar Pradesh.

**Habit.** Annual.

**872. **Capillipedium magdalenae** M.R. Almeida, J. Bombay Nat. Hist. Soc. 72: 813. 1976 (as “magdaleni”) **

*Dichanthium magdalenae* (M.R. Almeida) S.K. Jain & Deshp., Bull. Bot. Surv. India 20(1–4): 135. 1979 (“1978”) (as “magdaleni”)

**Type.** India: Karnataka, Agumbe; November, 1972; *M.R. Almeida 2566* (BLAT).

**Distribution.** Karnataka.

**Habit.** Annual.

**873. **Capillipedium nagense** Bor, Brittonia 16: 228. 1964 **

*Dichanthium nagense* (Bor) Deshp., Bull. Bot. Surv. India 21(1–4): 198. 1981 (“1979”)

**Type.** India: Assam, Naga Hills, Dzulake Valley alt. 8500 ft.; September, 1993; *N.L. Bor’s collector 353* (K).

**Distribution.** Assam, Nagaland.

**Habit.** Perennial.

**874. **Capillipedium parviflorum** (R. Br.) Stapf, Fl. Trop. Afr. [Oliver et al.] 9: 169. 1917 **

*Holcus parviflorus* R. Br., Prodr. Fl. Nov. Holland. 1: 199. 1810. Type: Australia; *R. Brown s.n.* (BM).

*Anatherum parviflorum* (R. Br.) Spreng., Syst. Veg. (ed. 16) [Sprengel] 1: 290. 1824 (“1825”)

*Dichanthium parviflorum* (R. Br.) De Wet & J. R. Harlan, Amer. J. Bot. 54(3): 384. 1967

*Sorghum parviflorum* (R. Br.) P. Beauv., Ess. Agrostogr. 165. 1812

*Andropogon alternans* J. Presl in C. Presl, Reliq. Haenk. 1(4–5): 342. 1830. Type: “Habitat in Peruvia.” Philippines: Luzon island; *Haenke s.n. *(US-76233 (fragm.)).

*Andropogon micranthus* Kunth, Révis. Gramin. 1: 165. 1829

*Andropogon quartinianus* A. Rich., Tent. Fl. Abyss. 2: 469. 1850–1851. Type: “Crescit in planitie montosa Beless, provinciae Chire, mense Octobre (Quartin Dillon).”

*Sorghum quartinianum* (A. Rich.) Schweinf., Beitr. Fl. Aethiop. 306. 1867

*Andropogon parvispica* Steud., Syn. Pl. Glumac. 1: 397. 1854. Type: Nepal; *Royle 283*

*Chrysopogon parvispicus* (Steud.) Will. Watson in Atkins., Bot. Himalayan Distr. N. W. Prov. 392. 1882 (as “parvispica”)

*Andropogon capilliflorus* Steud., Syn. Pl. Glumac. 1: 397. 1854. Type: Java, Japonica

*Chrysopogon violascens* Trin., Mém. Acad. Imp. Sci. St.-Pétersbourg, Sér. 6, Sci. Math. 2: 319. 1832. Type: “Andropogon violascens N. Ab Es. In Sieb. Agrostoth. no. 65”. “V. ssp. nov. Holl.”

*Andropogon violascens* (Trin.) Nees, Syn. Pl. Glumac. (Steudel) 1: 396. 1854

*Holcus caerulescens* Gaudich., Voy. Monde, Phan. 10: 411, t. 27. 1829. Type: “Habitat in Novae-Hollandiae ora orientali; Port-Jackson.”

*Rhaphis caerulescens* (Gaudich.) Desv., Opusc. Sci. Phys. Nat. 69. 1831

*Andropogon serratus* sensu Miq., Ann. Mus. Bot. Lugduno-Batavi 2: 290. 1866, non Thunb., 1784

*Rhaphis microstachya* Nees ex Steud., Syn. Pl. Glumac. 1: 397. 1854, nom. inval., cited as synonym of *A. parvispica*

*Rhaphis villosula* Nees ex Steud., Syn. Pl. Glumac. 1: 397. 1854, nom. inval., cited as synonym of *A. villosul*us

*Chrysopogon parviflorus* (R. Br.) Benth., Fl. Austral. 7: 537. 1878, isonym, non (R. Br.) Nees, 1843

**Distribution.** Andhra Pradesh, Assam, Bihar, Himachal Pradesh, Jammu and Kashmir, Jharkhand, Sikkim, Madhya Pradesh, Manipur, Meghalaya, Nagaland, Odisha, Rajasthan, Uttar Pradesh, Uttarakhand, West Bengal [Bhutan, China, Myanmar, Nepal, Pakistan].

**Habit.** Perennial.

**875. **Capillipedium planipedicellatum** Bor, Kew Bull. 1949: 222. 1949 **

*Dichanthium planipedicellatum* (Bor) S.K. Jain & Deshp., Bull. Bot. Surv. India 20(1–4): 135. 1979 (“1978”)

*Filipedium planipedicellatum* (Bor) Raizada & S.K. Jain, J. Bombay Nat. Hist. Soc. 49: 683. 1951

**Type.** India: Manipure State, Palel, alt. 2500 ft.; October 10, 1942; *N.L. Bor 17059* (K).

**Distribution.** Manipur.

**Habit.** Perennial.

**876. **Capillipedium pteropechys** (C.B. Clarke) Stapf, Hooker’s Icon. Pl. 31: sub t. 3085. 1922 **

*Andropogon pteropechys* C.B. Clarke, J. Linn. Soc., Bot. 25: 88. t. 38. 1899. Syntypes: India: Kohima, alt. 5500 ft.; *C.B. Clarke 41187* (K). Jakpho, alt. 7500 ft.; *C.B. Clarke 41896* (K).

*Dichanthium pteropechys* (C.B. Clarke) S.K. Jain & Deshp., Bull. Bot. Surv. India 20(1–4): 135. 1979 (“1978”). Type: India: Naga Hills, Kohima edge; *C.B. Clarke 61896A *(CAL).

**Distribution.** Manipur, Nagaland.

**Habit. **Perennial.

***Chionachne* R. Br., Pl. Jav. Rar. (Bennett) 15, 18. 1838**

**Type. ***Chionachne barbata* (Roxb.) R. Br. ex Aitch (*Coix barbata* Roxb.).

**877. **Chionachne digitata **(L.f.) E.A. Kellogg, comb. nov. **

urn:lsid:ipni.org:names:77212132-1

*Apluda digitata* L.f., Suppl. Pl. 434. 1782 (“1781”). Type: “Habitat in India”; *Thunberg s.n.*

*Polytoca digitata* (L.f.) Druce, Rep. Bot. Exch. Club Soc. Brit. Isles 1916, Suppl. 2: 641. 1917

*Coix heteroclita* Roxb., Fl. Ind. (Roxburgh) 3: 572. 1832. Type: India: Bengal; *Kyd s.n. Å1810 in herb. Roxburgh* (BM, *Icon. Ined. 2550=* Numer. List [Wallich] no. 8627A, IDC microfiche 7394).

*Polytoca heteroclita* (Roxb.) Koord., Exkurs.-Fl. Java 1: 99. 1911

*Polytoca bracteata* R. Br., Pl. Rar. Jav. 20. t. 5. 1838. Type: Indonesia: Java, Prowoto Hills, 20 miles south of Semarang; 1809; *Horsfield 113* (BM).

**Distribution.** Assam, Bihar, Manipur, Meghalaya, Tripura, Sikkim, West Bengal [Myanmar].

**Habit.** Perennial.

**Remarks.** The above combination is made here to transfer *Polytoca digitata* (L.f.) Druce to *Chionachne*.

**878. **Chionachne gigantea** (J. Koenig) Veldkamp, Blumea 47(3): 559. 2002 **

*Coix gigantea* J. Koenig, Naturforscher (Halle) 23: 211. 1788. Type: India: Andhra Pradesh, Circars; *Koenig s.n. *(BM, K, UPS, Herb. Thunberg 21727, IDC microfiche 1036).

*Coix koenigii* Spreng., Syst. Veg. (ed. 16) [Sprengel] 1: 239. 1824 (“1825”), nom. nov. for *Coix arundinacea *J.Konig ex Willd.

*Chionachne koenigii* (Spreng.) Thwaites, Enum. Pl. Zeyl. (Thwaites). 357. 1864

*Polytoca barbata* (Roxb.) Stapf, Fl. Brit. India (J.D. Hooker). 7(21): 102. 1896

*Coix lacryma-jobi* L. var. *gigantea* (J. Koenig) Stapf, Fl. Brit. India (J.D. Hooker). 7(21): 100. 1896

*Coix lingulata* Hack., Oesterr. Bot. Z. 41: 5. 1891. Type: Burma: Shan Hills, Fort Stedmens, alt. 3000 ft.; *H. Collett. s.n.*

*Coix barbata* Roxb., Fl. Ind. (Roxburgh) 3: 569. 1832. Type: India: Andhra Pradesh, Northern Circars, Moist rice field; *Roxburgh s.n.* (BM, sh. 47254, Icon. Ined. 871=Numer. List [Wallich] no. 8626-A:K, IDC microfiche 7394).

*Chionachne barbata* (Roxb.) R. Br. ex Aitch., Cat. Pl. Punjab Sindh 157. 1869

*Coix crypsoides* C. Mull., Bot. Zeitung (Berlin) 19(45): 334. 1861. Type: Bengalia Indiae orientalis; *Griffith s.n.* (B (destroyed); IT: K).

*Coix arundinacea* J. Koenig ex Willd., Sp. Pl., ed. 4 [Willdenow] 4: 203. 1805, non Lam., 1791. Type: India: Transchaur, Rarissime; *De Friedland s.n.* (B-W-17908-b (IDC microfiche 7440)).

**Distribution.** Andhra Pradesh, Arunachal Pradesh, Assam, Bihar, Chhattisgarh, Gujarat, Karnataka, Kerala, Madhya Pradesh, Maharashtra, Manipur, Meghalaya, Odisha, Rajasthan, Tamil Nadu, Tripura, West Bengal [Myanmar, Nepal, Pakistan, Sri Lanka].

**Habit.** Perennial.

**Remarks.** Some authors maintain *Chionachne* as distinct from *Polytoca* (Jannink and Veldkamp 2002), but Kellogg (2015b) combines them, pointing out that they are sister genera in molecular phylogenies and are morphologically similar.

**879. **Chionachne punctata** (R. Br.) Jannink, Blumea 47(3): 566–567, f. 8. 2002 **

*Sclerachne punctata* R. Br., Pl. Jav. Rar. (Bennett) 15. t. 4. 1838. Type: Indonesia: Java, near Surakarta, Jebus village; *Horsfield s.n.* (BM).

*Polytoca punctata* (R. Br.) Stapf ex Hook. f., Fl. Brit. India (J.D. Hooker). 7(21): 102. 1896

*Chionachne massii* Balansa, J. Bot. (Morot) 4: 78. 1890. Type: “Cette espece a été trouvée par M. Massie, pharmacien de la marine, dans les environs de Sontay. Elle a été recueillie aussi a Bac-ninh par M. Brousemiche.”

**Distribution.** Andhra Pradesh, Karnataka, Maharashtra [Myanmar].

**Habit. **Annual.

**880. **Chionachne semiteres** (Benth. ex Hook. f.) Henrard, Meded. Rijks-Herb. 67: 16. 1931 **

*Polytoca semiteres* Benth. ex Hook. f., Fl. Brit. India (J.D. Hooker). 7(21): 101. 1896. Type: Deccan Peninsula, Palamcotta; *Wight s.n.* Burma: Taong-dong Hills; *Wallich s.n.*

**Distribution.** Andhra Pradesh, Bihar, Maharashtra, Tamil Nadu [Myanmar].

**Habit.** Annual.

**881. **Chionachne wallichiana** (Nees) E.A. Kellogg, comb. nov. **

urn:lsid:ipni.org:names:77212133-1

*Cyathorhachis wallichiana* Nees, Syn. Pl. Glumac. (Steudel) 1: 403. 1854. Type: India; Numer. List [Wallich] no. 8629

*Polytoca wallichiana* (Nees) Benth., J. Linn. Soc., Bot. 19: 52. 1881

**Distribution.** Assam, Mizoram, Sikkim [Myanmar].

**Habit. **Annual.

**Remarks.** Records for Mizoram and Sikkim fide Naithani (1990). The above combination is made here to transfer *Polytoca wallichiana *(Nees) Benth. to *Chionachne*.

***Chrysopogon* Trin., Fund. Agrost. (Trinius) 187. 1820, nom. cons.**

**Type. ***Chrysopogon gryllus* (L.) Trin. (*Andropogon gryllus* L.) (typ. cons.).

**882. **Chrysopogon aciculatus** (Retz.) Trin., Fund. Agrost. (Trinius) 188. 1820 **

*Andropogon aciculatus* Retz., Observ. Bot. (Retzius) 5: 22. 1788 (“1789”) (as “aciculatum”). Type: “Habitat in Indiae Orientalis sterilibus et ruderatis. Gramen aciculatum Rumph. Amb. VI. p. 14. pl. 5. f.1.” Lectotype: India; *Koenig s.n. in herb. Retzius *(LD). LT designated by Veldkamp in Austrobaileya 5(3): 509. 1999.

*Rhaphis aciculatus* (Retz.) Desv., Opusc. Sci. Phys. Nat. 69. 1831 (as “aciculare”)

*Andropogon javanicus* Steud., Syn. Pl. Glumac. 1: 396. 1854. Lectotype: Indonesia: Java; *Junghuhn s.n.* (P). LT designated by Veldkamp in Austrobaileya 5(3): 510. 1999.

*Centrophorum chinense* Trin., Fund. Agrost. (Trinius) 106. t. 5. 1820. Type: “Vidi ramum hujus Graminis singularis in Herbario ditissimo Gorenkensi.”

*Rhaphis trivialis* Lour., Fl. Cochinch. 2: 553. 1790. Type: “Habitat ubique prope vias, hominibus valde incommode in Cochinchina & China, quia vestibus adhaerens taediose avellitur, cum ex cuti nequeat.”

*Chrysopogon trivialis* Arn. & Nees, Nov. Actorum Acad. Caes. Leop.-Carol. Nat. Cur., Suppl. 19(1): 171. 1843, nom. superfl. & illeg. for Chrysopogon aciculatus (Retz.) Trin.

*Rhaphis javanica* Nees ex Steud., Syn. Pl. Glumac. 1: 396. 1854, nom. invalid. cited as synonym of *Andropogon javanicus.*

*Andropogon acicularis* Roem. & Schult., Syst. Veg., ed. 15 bis [Roemer & Schultes] 2: 812. 1817, orth. var.

**Distribution.** Andaman and Nicobar Islands, Andhra Pradesh, Arunachal Pradesh, Assam, Bihar, Chhattisgarh, Delhi, Goa, Gujarat, Jharkhand, Karnataka, Kerala, Madhya Pradesh, Maharashtra, Manipur, Meghalaya, Odisha, Sikkim, Tamil Nadu, Tripura, Uttar Pradesh, Uttarakhand, West Bengal [Afghanistan, Bangladesh, Bhutan, China, Myanmar, Nepal, Pakistan, Sri Lanka].

**Habit.** Perennial.

**883. **Chrysopogon asper** Heyne ex Blatter & McCann, Sci. Monogr. Imp. Council Agric. Res. India (Bombay Grasses) 5: 68. 1935 **

*Andropogon asper* Heyne ex Hook. f., Fl. Brit. India (J.D. Hooker). 7(21): 189. 1896, non (Thunb.) Kunth, 1829, nom. later hom. illeg. Type: India: Madras, Pulicat Hills; *Heyne s.n.*

**Distribution.** Andhra Pradesh, Karnataka, Kerala, Maharashtra, Tamil Nadu.

**Habit.** Perennial.

**884. **Chrysopogon aucheri** (Boiss.) Stapf, Bull. Misc. Inform. Kew 1907: 211. 1907 **

*Andropogon aucheri* Boiss., Diagn. Pl. Orient. ser. 1, 1(5): 77. 1844. Type: “Hab. in desertis Persiae australis; Aucher no. 5465.”

*Chrysopogon ciliolatus* (Nees ex Steud.) Boiss. var. *aucheri* Boiss. (Boiss), Fl. Orient. (Boissier) 5: 458. 1884

**Distribution.** Gujarat, Rajasthan [Afghanistan, Pakistan].

**Habit.** Perennial.

**885. **Chrysopogon castaneus** Veldkamp & Salunkhe, Rheedea 10(1): 59. 2000 **

**Type.** India: Maharashtra, Sindhudurg District, Amboli-Choukul, alt. 2320 ft.; September 18, 1993; *S.R. Yadav 8678* (L).

**Distribution.** Maharashtra.

**Habit. **Perennial.

**886. **Chrysopogon copei** N. Mohanan & Ravi, Rheedea 11(2): 87. 2001. **

**Type.** India: Tamil Nadu, Tirunelveli district, Shengelteri, 700 m, 22 December 2000, *Mohanan45125* (hototype: TBGT; isotype: CAL, CALI, K, MH).

**Distribution.** Tamil Nadu.

**Habit.** Perennial.

**887. **Chrysopogon fulvus** (Spreng.) Chiov., Fl. Somala 1: 327. 1929 **

*Pollinia fulva* Spreng., Pl. Min. Cogn. Pug. 2: 10. 1815. Type: “Habitat in Bengalia”; *Roxburgh s.n.*

*Andropogon sprengelii* Kunth, Révis. Gramin. 1: 166. 1829

*Chrysopogon montanus* Trin. in Spreng., Neue Entdeck. Pflanzenk. 2: 93. 1821. Type: “Hab. in India Orientali”; *Koenig s.n. ex herb. Banks in herb. Jacquin *(W).

*Chrysopogon monticola* Haines, Indian Forester 40(10): 495. 1914, nom. superfl. & illegit.

*Andropogon montanus* J. Koenig ex Trin. in Spreng., Neue Entdeck. Pflanzenk. 2: 93. 1821, pro. syn., nom. nud.

*Andropogon monticola* Schult. & Schult. f., Mant. 3 (Schultes & Schultes f.) Add. I665. 1827, nom. superfl. for *Chrysopogon montanus*

**Distribution.** Andhra Pradesh, Bihar, Chhattisgarh, Daman and Diu, Delhi, Goa, Gujarat, Jammu and Kashmir, Himachal Pradesh, Karnataka, Kerala, Madhya Pradesh, Maharashtra, Odisha, Rajasthan, Tamil Nadu, Uttar Pradesh, Uttarakhand [China, Myanmar, Sri Lanka].

**Habit.** Perennial.

**888. **Chrysopogon gryllus** (L.) Trin., Fund. Agrost. (Trinius) 188. 1820 ssp. **gryllus

*Andropogon gryllus* L., Cent. Pl. II 33. 1756. Type: “Habitat in Rhaetia, Helvetia, Veronae, Sauvages, Seguier.” Lectotype: Seguier, Herb. Linn. No. 1211.2 (LINN). LT designated by Meikle in Fl. Cyprus 2: 1863. 1985.

*Holcus gryllus* (L.) R. Br., Prodr. Fl. Nov. Holland. 1: 199. 1810

*Pollinia gryllus* (L.) Spreng., Pl. Min. Cogn. Pug. 2: 10. 1815

*Andropogon royleanus* Steud., Syn. Pl. Glumac. 1: 397. 1854. Type: Nepal.

*Chrysopogon glabratus* Trin., Mém. Acad. Imp. Sci. St.-Pétersbourg, Sér. 6, Sci. Math. 2(4): 318. 1832. Type: “V. spp. Nepal.” Nepal; *Wallich s.n.*

*Andropogon glabratus* (Trin.) Steud., Nomencl. Bot., ed. 2 (Steudel) 1: 91. 1840

*Apluda gryllus* sensu (L.) C. Presl, Cyper. Gramin. Sicul. 55. 1820, non P. Beauv., 1812

**Distribution.** Assam, Himachal Pradesh, Karnataka, Meghalaya, Nagaland, Jammu and Kashmir, Rajasthan, Sikkim, Uttar Pradesh [Nepal].

**Habit.** Perennial.

**889. **Chrysopogon gryllus** L. ssp. **echinulatus** (Steud.) Cope, Kew Bull. 35(3): 701. 1980 **

*Andropogon echinulatus* Steud., Syn. Pl. Glumac. 1: 397. 1854. Type: Nepal; *Royle 226.*

*Chrysopogon echinulata* (Steud.) Will. Watson in Atkins., Bot. Himalayan Distr. N. W. Prov. 10: 392. 1882

*Andropogon gryllus* L. ssp. *echinulatus* (Steud.) Hack., Monogr. Phan. [A. DC. & C. DC.] 6: 552. 1889

*Chrysopogon aciculatus* Trin. var. *echinulatus* (Steud.) S.K. Jain, Phytotaxonomy 3: 136. 2004

*Rhaphis echinulata* Nees, Ill. Bot. Himal. Mts. 1: 417. 1840, nom invalid., nom. nud. Type: Northwest India; *Royle 226* (LIV).

**Distribution.** Assam, Sikkim, Tamil Nadu, Uttar Pradesh [Nepal].

**Habit.** Perennial.

**890. **Chrysopogon hackelii** (Hook. f.) C.E.C. Fisch., Fl. Madras 3(10): 1739. 1934 **

*Andropogon hackelii* Hook. f., Fl. Brit. India (J.D. Hooker). 7(21): 194. 1896. Type: India: Travancore, Nilghiri Hills; *Wight 1030* (K). Gondaloo Ghat, alt. 4500 ft.; *Lawson s.n.* Numer. List [Wallich] no. 8783

**Distribution.** Andhra Pradesh, Karnataka, Kerala, Maharashtra, Rajasthan, Tamil Nadu, Uttar Pradesh.

**Habit.** Perennial.

**891. **Chrysopogon hamiltonii** (Hook. f.) Haines, Bot. Bihar Orissa 5: 1036. 1924 **

*Andropogon hamiltonii* Hook. f., Fl. Brit. India (J.D. Hooker). 7(21): 190. 1896 (as “hamiltoni”). Type: India: Behar, Monghir; *Buchanan-Hamilton s.n.*

**Distribution.** Andhra Pradesh, Bihar, Gujarat, Karnataka, Maharashtra, Odisha, Tamil Nadu, West Bengal.

**Habit.** Perennial.

**892. **Chrysopogon lancearius** (Hook. f.) Haines, Bot. Bihar Orissa 5: 1036. 1924 **

*Andropogon lancearius* Hook. f., Fl. Brit. India (J.D. Hooker). 7(21): 190. 1896. Syntypes: India: Sikkim Himalaya, Punkabaree; *Kurz s.n.* Chota Nagpore; Ramghur, Ghat, 1750 ft.; *C.B. Clarke s.n.*

**Distribution.** Andhra Pradesh, Bihar, Jharkhand, Maharashtra, Odisha, Sikkim, West Bengal.

**Habit.** Perennial.

**893. **Chrysopogon lawsonii** (Hook. f.) Veldkamp, Austrobaileya 5(3): 515. 1999 **

*Vetiveria lawsonii* (Hook. f.) Blatt. & McCann, J. Bombay Nat. Hist. Soc. 32: 409. 1928 (as “lawsoni”)

*Andropogon lawsonii* Hook. f., Fl. Brit. India (J.D. Hooker). 7(21): 187. 1896. Type: India: Mysore, Bandypore, in moist ground, alt. 3000 ft.; *Lawson s.n.*

**Distribution.** Andhra Pradesh, Karnataka, Madhya Pradesh, Maharashtra, Rajasthan, Tamil Nadu.

**Habit.** Not recorded.

**894. **Chrysopogon narayanii** Sunil, Ratheesh, & Sivadasan, Phytotaxa 307 (4): 249. 2017. **

**Type. **India: Kerala, Kannur district, Madayippara, 40 m, 5 December 2014, *Sunil, R. Narayanam7187* (holotype: CAL; isotype: MH).

**Distribution.** Kerala.

**Habit.** Perennial.

**895. **Chrysopogon nodulibarbis** (Hochst ex Steud.) Henrard, Blumea 4(3): 534. 1941 **

*Andropogon nodulibarbis* Hochst ex Steud., Syn. Pl. Glumac. 1: 396. 1854. Type: India: Tamil Nadu, Nilgiri Mountains;* R.F. Hohencaker 934*

*Andropogon zeylanicus* Steud., Syn. Pl. Glumac. 1: 397. 1854. Type: Sri Lanka.

*Chrysopogon zeylanicus* (Steud.) Thwaites, Enum. Pl. Zeyl. (Thwaites) 366. 1864

*Andropogon peninsulae* Steud., Syn. Pl. Glumac. 1: 396. 1854. Type: India; Numer. List [Wallich] no. 8785 as “Chrysopogon”

**Distribution.** Karnataka, Kerala, Maharashtra, Tamil Nadu [Sri Lanka].

**Habit.** Perennial.

**896. **Chrysopogon orientalis** (Desv.) A. Camus, Fl. Indo-Chine [P.H. Lecomte et al.] 7: 332. 1922 **

*Rhaphis orientalis* Desv., Opusc. Sci. Phys. Nat. 69. 1831. Type: “India orientalis.” India; *Klein 392* (B-W-18636 (sheet 4)).

*Andropogon aristulatus* Steud., Syn. Pl. Glumac. 1: 372. 1854. Type: “Ins. Borbon”

*Andropogon breviaristatus* Steud., Syn. Pl. Glumac. 1: 396. 1854. Type: India: Tamil Nadu, Coimbatore, Nilgiri Hills, prope Kaity; October 18; *R. F. Hohenacker 1285* (P).

*Andropogon wightianus* Steud., Syn. Pl. Glumac. 1: 395. 1854. Lectotype: India; *Wight 1676* (P). LT designated by Veldkamp, Austrobaileya 5(3): 518. 1999.

*Chrysopogon wightianus* (Steud.) Thwaites, Enum. Pl. Zeyl. (Thwaites). 366. 1864

**Distribution.** Andhra Pradesh, Assam, Bihar, Karnataka, Kerala, Madhya Pradesh, Maharashtra, Odisha, Tamil Nadu [China, Myanmar, Sri Lanka].

**Habit.** Perennial.

**897. **Chrysopogon polyphyllus** (Hack. ex Hook. f.) Blatt. & McCann, J. Bombay Nat. Hist. Soc. 32: 416. 1928 **

*Andropogon polyphyllus* Hack. ex Hook. f., Fl. Brit. India (J.D. Hooker). 7(21): 194–195. 1896. Type: India: Madhya Pradesh, Khandua, Chanda and Jubbulpore Districts, by the Nerbudda and Indravati rivers; *J.C. Duthie 8491* (K).

**Distribution.** Andhra Pradesh, Bihar, Dadra and Nagar Haveli, Goa, Gujarat, Karnataka, Madhya Pradesh, Maharashtra, Tamil Nadu, Uttar Pradesh [also in Malaysia, North America].

**Habit.** Perennial.

**898. **Chrysopogon pseudozeylanicus** K.G. Bhat & Nagendran, Reinwardtia 10(2): 128, f. 2. 1985 **

**Type.** India: Karnataka State, South Kanara District, Charmadi ghat, about 15 km from Kotigehar on the way to Dharmasthala; November, 1977; *K.G. Bhat 550A* (CAL).

**Distribution.** Karnataka.

**Habit.** Perennial.

**899. **Chrysopogon purushothamanii** Ravi, N. Mohanan & Kiran Raj, Rheedea 10(2): 94. f. 2. 2000 **

**Type.** India: Kerala State, Pathanamthitta District. Kozhanchery Taluk, Kokkathode, Kattathipara, alt. 500 ft.; November 25, 2000; *Ravi 44811* (TBGT).

**Distribution.** Kerala.

**Habit.** Perennial.

**900. **Chrysopogon serrulatus** Trin., Mém. Acad. Imp. Sci. St.-Pétersbourg, Sér. 6, Sci. Math. 2(3): 318. 1832 **

*Chrysopogon fulvus* (Spreng.) Chiov. var. *serrulatus* (Trin.) R.R. Stewart, Brittonia 5(4): 466. 1945

*Chrysopogon montanus* Trin. var. *serrulatus* (Trin.) Stapf, Fl. Trop. Afr. [Oliver et al.] 9: 160. 1917, in obs.

*Andropogon coeruleus* Steud., Syn. Pl. Glumac. 1: 395. 1854. Type: India; *Royle Herb. no. 254, 255*

*Andropogon ciliolatus* Steud., Syn. Pl. Glumac. 1: 396. 1854. Type: India; *Royle Herb. no. 318*. India; Numer. List [Wallich] no. 8788

*Chrysopogon ciliolatus* (Steud.) Boiss., Fl. Orient. (Boissier) 5: 458. 1884

*Andropogon monticola* Schultes var. *trinii* (Steud.) Hook. f., Fl. Brit. India (J.D. Hooker). 7(21): 193. 1896

*Andropogon trinii* Steud., Syn. Pl. Glumac. 1: 395. 1854. Type: Nepal

*Andropogon trinii* Steud. var. *increscens* Hack., Monogr. Phan. [A. DC. & C. DC.] 6: 558–559. 1889. Syntypes: India: Coutrallum; *Wight 1030*. Sri Lanka; *Thwaites 2954*

*Chrysopogon wightianus* (Steud.) Thwaites var. *leucanthus* Thwaites, Enum. Pl. Zeyl. (Thwaites). 366. 1864. Type: Sri Lanka: Doombera District; *Thwaites Ceylon Plant 3248*

**Type.** Nepal; 1821; *Wallich *(Numer. List [Wallich] no. 8791).

**Distribution.** Andhra Pradesh, Jammu and Kashmir, Karnataka, Madhya Pradesh, Maharashtra, Punjab, Uttar Pradesh, Uttarakhand [Afghanistan, Myanmar, Nepal, Pakistan, Sri Lanka].

**Habit.** Perennial.

**901. **Chrysopogon tadulingamii** Sreek., V.J. Nair & N.C. Nair, J. Bombay Nat. Hist. Soc. 80: 198. f. 15. 1983 **

**Type.** India: Kerala, Cannanore District, Periye, alt. 200 ft.; October 18, 1981; *P.V. Sreekumar 71758* (CAL).

**Distribution.** Andhra Pradesh, Kerala.

**Habit.** Perennial.

**902. **Chrysopogon velutinus** (Hook. f.) Bor, Grasses Burma, Ceylon, India & Pakistan 119. 1960 **

*Andropogon velutinus* Hook. f., Fl. Brit. India (J.D. Hooker). 7(21): 194. 1896. Type: India: Mysore, Chuddapah; *Wight 2314*

*Chrysopogon velutinus* Arn. ex Hook. f., Fl. Brit. India (J.D. Hooker). 7(21): 194. 1896, nom. nud. pro. syn.

**Distribution.** Andhra Pradesh, Karnataka, Kerala, Tamil Nadu.

**Habit.** Annual.

**903. **Chrysopogon verticillatus** (Roxb.) Trin. ex Steud., Nomencl. Bot., ed. 2 (Steudel) 1: 360. 1840 **

*Andropogon verticillatus* Roxb., Fl. Ind. (Carey and Wallich ed.) 1: 267. 1820. Type: India: “A native of Mountains, flowering about the end of the wet season.”

**Distribution.** Andhra Pradesh, Chhattisgarh, Karnataka, Kerala, Madhya Pradesh, Odisha, Tamil Nadu.

**Habit.** Perennial.

**904. **Chrysopogon zizanioides** (L.) Roberty, Bull. Inst. Franç. Afrique Noire 22: 106. 1960 **

*Phalaris zizanioides* L., Mant. Pl. Altera 183. 1771. Type: “Habitat in India orientali. Koenig.” Lectotype: Herb. Linn. No. 78.12 (LINN). LT designated by van der Zon in Wageningen Agric. Univ. Pap. 92-1: 409. 1992.

*Vetiveria zizanioides* (L.) Nash, Fl. S.E. U.S. [Small]. 67, 1326. 1903

*Anatherum zizanioides* (L.) Hitchc. & Chase, Contr. U.S. Natl. Herb. 18: 285. 1917

*Andropogon zizanioides* (L.) Urban, Symb. Antill. (Urban). 4: 79. 1903

*Holcus zizanioides* (L.) Kuntze ex Stuck., Anales Mus. Nac. Buenos Aires 11: 48. 1904

*Sorghum zizanioides* (L.) Kuntze, Revis. Gen. Pl. 2: 791. 1891

*Andropogon festucoides* J. Presl in C. Presl, Reliq. Haenk. 1: 340. 1830. Type: “Hab. in Luzonia.” Philippines: Luzon Island; *T. Haenke s.n. *(PR).

*Andropogon muricatus* Retz., Observ. Bot. (Retzius) 3: 43. 1783 (as “muricatum”). Type: “Dedit Honoratiss Koenig.” India; *Koenig s.n.*

*Anatherum muricatum* (Retz.) P. Beauv., Ess. Agrostogr. 150. t. 22. f. 10. 1812

*Vetiveria muricata* (Retz.) Griseb., Fl. Brit. W.I. [Grisebach] 560. 1864

*Vetiveria arundinacea* Griseb., Fl. Brit. W.I. [Grisebach] 559. 1864. Type: “P.B. Agrost. t. 22. f. 10 anal.”

*Vetiveria odorata* Virey, J. Pharm. 13: 501. 1827. Type: “East Indies. Ref. is made to Bull. Soc. Philom. 1822: 44.”

*Vetiveria odoratissima* Lem.-Lisanc., Bull. Soc. Philom. Paris 43. 1822. Type: Sri Lanka: “à Bourbon à l’lile de France oú elle a été apportée, sous l’ intendance de M. Poivre’, M. Poivre”( P?). (vide Tropicos)

*Andropogon squarrosus* sensu Hook. f. in Trimen, Handb. Fl. Ceylon 5: 233. 1900, non L. f., 1781

*Agrostis verticillata* Lam., Encycl. (Lamarck) 1: 59. 1783, non Vill., 1779. Type: “Cette plante croit dans I’Inde, & m’a été communiqué par M. Sonnerat.”

**Distribution.** Andhra Pradesh, Assam, Bihar, Chhattisgarh, Delhi, Goa, Gujarat, Jharkhand, Jammu and Kashmir, Karnataka, Kerala, Madhya Pradesh, Maharashtra, Odisha, Punjab, Rajasthan, Uttar Pradesh, Uttarakhand, Tamil Nadu, West Bengal [China, Nepal, Pakistan, Sri Lanka].

**Habit.** Perennial.

**Remarks.** Widely cultivated.

***Cleistachne* Benth., Hooker’s Icon. Pl. 14: 60, t. 1379. 1882**

**Type. ***Cleistachne sorghoides* Benth.

**905. **Cleistachne sorghoides** Benth., Hooker’s Icon. Pl. 14: 60, t. 1379. 1882 **

*Cleistachne stocksii* Hook. f., Fl. Brit. India (J.D. Hooker). 7(21): 162–163. 1896. Type: India: Malabar, Bababoodan Hills; *J.E. Stocks s.n., Ritchie s.n.*

**Type. **Mozambique: Shubanga, on the Zambesi; *J. Kirk s.n.* (K).

**Distribution.** Bihar, Gujarat, Karnataka, Kerala, Madhya Pradesh, Maharashtra, Tamil Nadu [also in Saudi Arabia, Africa].

**Habit.** Annual.

**Remarks.** Records for Bihar and Gujarat fide Naithani (1990).

***Coix* L., Sp. Pl. 2: 972. 1753**

**Lectotype. ***Coix lacryma-jobi* L. LT designated by G. V. Nash, N. Amer. Fl. 17: 82. 1909; affirmed by Hitchcock, Amer. J. Bot. 5: 250. 1918.

**906. **Coix aquatica** Roxb., Fl. Ind. (Roxburgh) 3: 571–572. 1832 **

**Type.** India: Lower parts of Bengal, generally found floating on lakes; *Roxburgh s.n.*

**Distribution.** Andhra Pradesh, Bihar, Chhattisgarh, Karnataka, Kerala, Madhya Pradesh, Maharashtra, Odisha, Rajasthan, Tamil Nadu, Uttar Pradesh, West Bengal [Bangladesh, Bhutan, China, Myanmar, Sri Lanka].

**Habit.** Annual.

**907. **Coix lacryma-jobi** L., Sp. Pl. 2: 972. 1753 **

*Lithagrostis lacryma-jobi* (L.) Gaertn., Fruct. Sem. Pl. 1: 7. t. 1. f. 10. 1788

*Coix agrestis* Lour., Fl. Cochinch. 2: 551. 1790. Type: “Habitat agrestis in locis humidis.”

*Coix arundinacea* Lam., Encycl. (Lamarck) 3(2): 422. 1792. Type: “Cette plante croit dans les pays chauds de l’Amerique.”

*Coix exaltata* J. Jacq., Ecl. Gram. Rar. 60. t. 40. 1820. Type: China

*Coix lacryma* L., Syst. Nat., ed 10. 2: 1261. 1759, nom. superfl. & illegit. for *C. lacryma-jobi*.

*Coix ovata* Stokes, Bot. Mat. Med. 4: 343. 1812, nom. superfl. & illeg.

*Coix pendula* Salisb., Prodr. Stirp. Chap. Allerton 28. 1796, nom. superfl. & illeg.

**Type.** “Habitat in Indiis.” Lectotype: *Herb. Linn. No. 1098.1* (LINN). LT designated by Clayton & Renvoize in Polhill (ed.), Fl. Trop. E. Africa, Gramineae 3: 857. 1982.

**Distribution.** Andaman and Nicobar Islands, Andhra Pradesh, Arunachal Pradesh, Assam, Bihar, Chhattisgarh, Daman and Diu, Dadra and Nagar Haveli, Goa, Gujarat, Himachal Pradesh, Karnataka, Kerala, Madhya Pradesh, Maharashtra, Manipur, Meghalaya, Nagaland, Odisha, Rajasthan, Sikkim, Tamil Nadu, Tripura, Uttar Pradesh, Uttarakhand, West Bengal [Bhutan, China, Myanmar, Nepal, Sri Lanka].

**Habit.** Annual.

**908. **Coix lacryma-jobi** L. var. **stenocarpa** Oliv., Hooker’s Icon. Pl. 18: t. 1764. 1888 **

*Coix stenocarpa* (Oliv.) Balansa, J. Bot. (Morot) 4(4): 77–78. 1890

*Coix tubulosa* Hack. ex Warbg. in Engl., Bot. Jahrb. Syst. 13(2): 260. 1890. Type: “Sattelberg bei Finschhafen.” Papua New Guinea; *E.F. Warburg 20946* (B).

**Syntypes.** Burma; *G. Watt s.n.* India: Mergui; *Griffith s.n.*

**Distribution.** Andhra Pradesh, Arunachal Pradesh, Assam, Karnataka, Manipur, Meghalaya, Mizoran, Nagaland, Rajasthan, Tripura, Uttarakhand [China, Myanmar].

**Habit.** Annual.

**909. **Coix lacryma-jobi** L. var. **ma-yuen** (Rom. Caill.) Stapf ex Hook. f., Fl. Brit. India (J.D. Hooker). 7(21): 100. 1896 **

*Coix ma-yuen* Rom. Caill., Bull. Soc. Natl. Acclim. France ser. 2, 8: 442. 1881. Type: India: Sikkim, up to 5000 ft.; *Watt?*

**Distribution.** Assam, Sikkim [Bhutan, China, Myanmar].

**Habit.** Annual.

**910. **Coix puellarum** Balansa, J**. **Bot. (Morot) 4(4): 77. 1890 **

*Coix lacryma-jobi* L. var. *puellarum* (Balansa) A. Camus, Fl. Indo-Chine [P.H. Lecomte et al.] 7: 220. 1922

**Type.** Vietnam: Tonkin; *Balansa s.n.*

**Distribution.** Arunachal Pradesh [China, Myanmar].

**Habit. **Annual.

***Cymbopogon* Spreng., Pl. Min. Cogn. Pug. 2: 14. 1815**

**Lectotype. ***Cymbopogon schoenanthus* (L.) Spreng. (*Andropogon schoenanthus* L.). LT designated by N.L. Britton & P. Wilson in Bot. Porto Rico 1: 27. 1923.

**911. **Cymbopogon caesius** (Nees ex Hook. & Arn.) Stapf, Bull. Misc. Inform. Kew 1906: 360–361. 1906 **

*Andropogon caesius* Nees ex Hook. & Arn., Bot. Beechey Voy. 6: 244. 1838. Syntypes: India; *Wight 1700b *(K). “N. ab E. in Wight Cat. n. 1700”. [China]: “Hab. a et b, in vicinia urbis Macao, et in insulis adjectis; *Millett; G.H. Vachell, n. 41. a*.”

*Andropogon schoenanthus* L. var. *caesius* (Nees ex Hook. & Arn.) Hack., Monogr. Phan. [A. DC. & C. DC.] 6: 610. 1889

*Andropogon schoenanthus* L. var. *gracillimus* Hook. f., Fl. Brit. India (J.D. Hooker). 7(21): 205. 1896. Type: Numer. List [Wallich] no. 8796

**Distribution.** Andhra Pradesh, Bihar, Gujarat, Karnataka, Kerala, Odisha, Tamil Nadu, Uttar Pradesh, Uttarakhand [China, Pakistan, Sri Lanka].

**Habit.** Perennial.

**912. **Cymbopogon citratus** (DC.) Stapf, Bull. Misc. Inform. Kew 1906: 322, 357. 1906 **

*Andropogon citratus* DC., Cat. Pl. Horti Monsp. 78. 1813 (as “citratum”). Type: “Cult. at Montpellier, France, from seed collected in Asia” Rumphius, Herb. Amboin. 5. p. 181. under *Schoenanthum amboinicum*, Specimen from Lambert’s Garden (BM).

**Distribution.** Andhra Pradesh, Goa, Gujarat, Karnataka, Kerala, Maharashtra,Rajasthan, Tamil Nadu, Uttar Pradesh [China].

**Habit.** Perennial.

**Remarks.** Widely cultivated.

**913. **Cymbopogon coloratus** (Hook. f.) Stapf, Bull. Misc. Inform. Kew 1906: 321, 356. 1906 **

*Andropogon nardus* L. var. *coloratus* Hook. f., Fl. Brit. India (J.D. Hooker). 7(21): 206–207. 1896. Syntypes: India; *Heyne s.n., Wight s.n.*

*Andropogon nardus* L. ssp. *glomeratus* Hack., Monogr. Phan. [A. DC. & C. DC.] 6: 604. 1889. Type: Peninsular India Orientalis; *Wight 3087*

**Distribution.** Andhra Pradesh, Karnataka, Tamil Nadu [China, Myanmar].

**Habit.** Perennial.

**914. **Cymbopogon commutatus** (Steud.) Stapf, Bull. Misc. Inform. Kew 1907: 211. 1907 var. **commutatus

*Andropogon commutatus* Steud., Syn. Pl. Glumac. 1: 387. 1854. Type: Ethiopia; *Schimper 685* (P).

*Cymbopogon parkeri* Stapf, Bull. Misc. Inform. Kew 1929(1): 10–11. 1929. Type: India: United Provinces, very common in the Ettawah afforestation area; August, 1923; *R.S. Pearson s.n.*

*Andropogon laniger* sensu Duthie, Fodder Grasses North. India 35. t. 23. 1888, non Desf., 1799

**Distribution.** Goa, Gujarat, Haryana, Jammu and Kashmir, Rajasthan, Uttar Pradesh [Pakistan].

**Habit.** Perennial.

**Remarks.** Records for India, except for Goa and Jammu and Kashmir, all fide Naithani (1990).

**915. **Cymbopogon commutatus** (Steud.) Stapf var. **jammuensis** (Gupta) Naithani, Fl. Pl. India, Nepal & Bhutan 483. 1990 **

*Cymbopogon parkeri* Stapf var. *jammuensis* Gupta, Proc. Ind. Acad. Sci. Sec. B, 71:96. 1970

**Distribution.** Jammu and Kashmir.

**Habit.** Perennial.

**916. **Cymbopogon distans** (Nees) Wats., Bot. Himalayan Distr. N. W. Prov. 392. 1882 **

*Andropogon distans* Nees, Syn. Pl. Glumac. (Steudel) 1: 387. 1854. Lectotype: Nepal; *Royle 236* (LIV). LT designated by Soenarko, Reiwardtia 9: 356. 1977.

*Cymbopogon distans* (Nees) Wats. var. *mundensis* Gupta, Proc. Ind. Acad. Sci. Sec. B, 71:97. 1970

**Distribution.** Jammu and Kashmir, Uttarakhand [China, Nepal, Pakistan].

**Habit.** Perennial.

**Remarks.** Record for Jammu and Kashmir fide Naithani (1990).

**917. **Cymbopogon flexuosus** (Nees) Will. Watson in Atkins., Bot. Himalayan Distr. N.W. Prov. 392. 1982 var. **flexuosus

*Andropogon flexuosus* Nees, Syn. Pl. Glumac.(Steudel) 1: 388. 1854. Type: India; *Wight 1704* (P). “A. nardus Klein herb. Ind. or.”

*Andropogon nardus* L. ssp. *flexuosus* (Nees) Hack., Monogr. Phan. [A. DC. & C. DC.] 6: 603. 1889. Type: Peninsular India; *Wight 171*

*Cymbopogon travancorensis* Bor, J. Bombay Nat. Hist. Soc. 52: 174–176. f. 24. 1954. Type: India: Courtallum, Tinnevelly; November 11, 1908; *Bourne 5309* (K).

**Distribution.** Andhra Pradesh, Bihar, Karnataka, Kerala, Maharashtra, Odisha, Sikkim, Tamil Nadu, West Bengal [China, Myanmar, Nepal].

**Habit.** Perennial.

**918. **Cymbopogon flexuosus** (Nees) Will. Watson var. **coimbatore** B.K. Gupta, Proc. Indian Acad. Sci., B. 71: 94. 1970 **

**Type.** India; *B.K. Gupta 32* (DD).

**Distribution.** Jammu and Kashmir, Sikkim, Tamil Nadu, West Bengal.

**Habit.** Perennial.

**Remarks.** Record for Tamil Nadu fide Naithani (1990).

**919. **Cymbopogon flexuosus** (Nees) Will. Watson var. **sikkimensis** Bor, J. Bombay Nat. Hist. Soc. 52: 162. 1954 **

**Syntypes.** India: Sikkim, Kynanooka, Terai; November, 1878; *J.S. Gamble 7363*. Tuckvar; September 19, 1874; *Treutler 792*. Rungpo, alt.; November 3, 1909; *I.H. Burkill 34063*. West Bengal, Darjeeling District, Badamtam, Ranjit; November 1, 1909; *I.H. Burkill 34012.*

**Distribution.** Arunachal Pradesh, Sikkim, Uttarakhand, West Bengal.

**Habit.** Perennial.

**Remarks.** Record for Sikkim fide Naithani (1990).

**920. **Cymbopogon gidarba** (Buch.- Ham. ex Steud.) Haines, Bot. Bihar Orissa 5: 1048. 1924 var. **gidarba

*Andropogon gidarba* Buch.- Ham. ex Steud., Syn. Pl. Glumac. 1: 387. 1854. Type: India; Numer. List [Wallich] no. 8797

**Distribution.** Andhra Pradesh, Bihar, Gujarat, Jharkhand, Karnataka, Maharashtra, Odisha, Tamil Nadu, West Bengal [China].

**Habit.** Perennial.

**921. **Cymbopogon jwarancusa** (Jones) Schult., Mant. 2 (Schultes) 458. 1824 ssp. **jwarancusa** (“iwarancusa”) **

*Andropogon jwarancusa* Jones, Asiat. Res. 4: 109. 1795. Type: India: Lucnow; *Blane s.n.*

*Andropogon himalayensis* Gand., Bull. Soc. Bot. France 46: 421. 1899, non Steud., 1854. Type: India: Jihri Garhwal, Tous Valley, alt. 3000–4000 ft.

**Distribution.** Andhra Pradesh, Bihar, Jammu and Kashmir, Rajasthan, Tamil Nadu, Uttar Pradesh, Uttarakhand [Afghanistan, Bhutan, China, Nepal, Pakistan, Sri Lanka].

**Habit.** Perennial.

**922. **Cymbopogon jwarancusa** (Jones) Schult. var. **assamensis** Gupta, Proc. Ind. Acad. Sci. Sec. B, 71:95. 1970. **

**Distribution.** Assam.

**Habit.** Perennial.

**923.** Cymbopogon jwarancusa** (Jones) Schult. ssp. **olivieri** (Boiss.) Soenarko, Reinwardtia 9(3): 307. 1977 **

*Andropogon olivieri* Boiss., Diagn. Pl. Orient. ser. 1, 1(5): 76. 1844. Type: Iraq: Baghdad (“Mesopotamia”); 1834; *Archer Eloy 2955* (G).

*Cymbopogon olivieri* (Boiss.) Bor, Notes Roy. Bot. Gard. Edinburgh 25(1): 62. 1963

*Andropogon iwarancusa* Jones ssp. *laniger* (Desf.) Hook. f., Fl. Brit. India (J.D. Hooker). 7(21): 203. 1896

*Andropogon laniger* Desf., Fl. Atlant. 2: 379. 1800. Type: “Habitat in montibus prope Cafsam” (as “lanigerum”)

*Cymbopogon ladakhensis* B.K. Gupta, Proc. Indian Acad. Sci., B. 71: 10. 1970. Type: India: Kashmir; *B.K. Gupta 37* (DD).

*Cymbopogon schoenanthus* sensu Bor, Grasses Burma, Ceylon, India and Pakistan 131. 1960, non (L.) Spreng., 1815

*Andropogon schoenanthus* sensu Hook. f., Fl. Brit. India (J.D. Hooker). 7(21): 204. 1896, non L., 1753

**Distribution.** Arunachal Pradesh, Himachal Pradesh, Jammu and Kashmir, Rajasthan, Tamil Nadu, Uttar Pradesh [Afghanistan, China, Nepal, Pakistan].

**Habit.** Perennial.

**Remarks.** Records for Arunachal Pradesh, Himachal Pradesh, Jammu and Kashmir, Uttar Pradesh and Nepal fide Naithani (1990).

**924. **Cymbopogon khasianus** (Hack.) Stapf ex Bor, Indian Forest Rec., Bot. n.s. 1(3): 92. 1938 **

*Andropogon nardus* L. ssp. *khasianus* Hack., Monogr. Phan. [A. DC. & C. DC.] 6: 603. 1889. Type: [Bangladesh] India: Silhet; Numer. List [Wallich] no. 8794-H; *Griffith 6764, 6765* (K).

*Andropogon khasianus* Munro ex Duthie, Fodder Grasses North. India 88. 1888, nom. nud.

**Distribution.** Assam, Manipur, Meghalaya, Nagaland [Bhutan, China, Myanmar].

**Habit.** Perennial.

**925. **Cymbopogon martini** (Roxb.) Will. Watson in Atkins., Bot. Himalayan Distr. N. W. Prov. 392. 1882 **

*Andropogon martini* Roxb., Fl. Ind. (Carey and Wallich ed.) 1: 280–281. 1820. Type: “A native of the high lands of Balla-ghat, General Martin collected the seeds while there with the army, during the last war with Tippoo Sultan, and has reared abundance of it at Lucknow.”

*Andropogon schoenanthus* L. var. *martini* (Roxb.) Hook. f., Fl. Brit. India (J.D. Hooker). 7(21): 204. 1896

*Cymbopogon martini* (Roxb.) Will. Watson var. *sofia* B.K. Gupta, Proc. Indian Acad. Sci., B 71: 97. 1970

*Andropogon pachnodes* Trin., Mém. Acad. Imp. Sci. St.-Pétersbourg, Sér. 6, Sci. Math. 2(3): 284. 1832. Type: Nepal; *Wallich s.n.*

*Cymbopogon pachnodes* (Trin.) Will. Watson in Atkins., Bot. Himalayan Distr. N.W. Prov. 392. 1882

*Cymbopogon motia* B.K. Gupta, Proc. Indian Acad. Sci., B 71: 92. 1970. Type: India; *B.K. Gupta 25 *(DD).

*Cymbopogon martinianus* Schult., Mant. 2 (Schultes) 459. 1824, nom. superfl. & illegit. for *Andropogon martini *Roxb.

**Distribution.** Andhra Pradesh, Assam, Bihar, Chhattisgarh, Goa, Gujarat, Himachal Pradesh, Karnataka, Kerala, Maharashtra, Odisha, Rajasthan, Tamil Nadu [China, Myanmar, Nepal, Pakistan]. Native to India; also cultivated.

**Habit.** Perennial.

**926. **Cymbopogon microstachys** (Hook. f.) Soenarko, Reinwardtia 9(3): 364. 1977 **

*Andropogon nardus* L. var. *microstachys* Hook. f., Fl. Brit. India (J.D. Hooker). 7(21): 207. 1896. Lectoype: India: Garo Hills; *Mann s.n.* (vide Soenarko, Reinwardtia 9(3): 364. 1977)

*Cymbopogon flexuosus* (Nees ex Steud.) Will.Watson var. *microstachys* (Hook. f.) Bor, J. Bombay Nat. Hist. Soc. 52: 162. 1954

**Distribution.** Assam, Meghalaya [China, Myanmar].

**Habit. **Perennial.

**927. **Cymbopogon microthecus** (Hook. f.) A. Camus, Rev. Bot. Appl. Agric. Colon. 1: 284. 1921 **

*Andropogon microtheca* Hook. f., Fl. Brit. India (J.D. Hooker). 7(21): 208. 1896. Type: Nepal: East Nepal, hot Valleys, alt. 3000–4000 ft.; *J.D. Hooker s.n.*

**Distribution.** Bihar, Sikkim [Nepal].

**Habit.** Perennial.

**928. **Cymbopogon nardus** (L.) Rendle, Cat. Afr. Pl. 2(1): 155. 1899 var. **nardus

*Andropogon nardus* L., Sp. Pl. 2: 1046. 1753. Type: “Habitat in India.” Lectotype: *Herb. Hermann 2: 66, No. 45* (BM-000594628). LT designated by Clayton & Renvoize in Polhill (ed.), Fl. Trop. E. Africa, Gramineae 3: 764. 1982.

*Andropogon ampliflorus* Steud., Syn. Pl. Glumac. 1: 388. 1854. Type: Indonesia: Java; *Zollinger 849* (P).

*Andropogon thwaitesii* Hook. f., Handb. Fl. Ceylon 5: 243. 1900. Type: Sri Lanka: Nuwara Eliya; August, 1864; *Thwaites Ceylon Plant 3784* (K).

*Cymbopogon thwaitesii* (Hook. f.) Willis, Revis. Cat. Fl. Pl. Ceylon 110. 1911

*Andropogon distans* Thwaites, Enum. Pl. Zeyl. (Thwaites) 367. 1864, non Nees ex Steud., 1854

**Distribution.** Andhra Pradesh, Assam, Bihar, Maharashtra, Karnataka, Kerala, Tamil Nadu, Tripura [Bhutan, Myanmar, Sri Lanka].

**Habit.** Perennial.

**929. **Cymbopogon nardus** (L.) Rendle var. **confertiflorus** (Steud.) Stapf ex Bor, J. Bombay Nat. Hist. Soc. 51: 905. 1953 **

*Andropogon confertiflorus* Steud., Syn. Pl. Glumac. 1: 385. 1854. Type: India: Tamil Nadu, Nilagiri Mountains; 1851; *R.F. Hohenacker 932* (K).

*Cymbopogon confertiflorus* (Steud.) Stapf, Bull. Misc. Inform. Kew 1906: 318, 355. 1906

*Andropogon nardus* L. var. *nilagiricus* Hook. f., Fl. Brit. India (J.D. Hooker). 7(21): 206. 1896. Type: India: Tamil Nadu, Nilagiri Mountains; 1851; *R.F. Hohenacker 932* (S-G402).

*Andropogon nardus* L. var. *luridus* Hook. f., Fl. Brit. India (J.D. Hooker). 7(21): 206. 1896. Type: India: Tamil Nadu, Nilghiri Hills; *Schmid s.n.* Sri Lanka; *Gardner s.n.*, *Maxwell s.n.*

*Andropogon nardus* L. ssp. *nilagiricus* Hack., Monogr. Phan. [A. DC. & C. DC.] 6: 604. 1889. Type: cited many syntypes

**Distribution.** Andhra Pradesh, Karnataka, Tamil Nadu [Sri Lanka].

**Habit.** Perennial.

**930. **Cymbopogon osmastonii** R. Parker, Repert. Spec. Nov. Regni Veg. 31: 126. 1932 **

**Type. **India: Assam, Kheri District; *Osmaston 1430* (K).

**Distribution.** Assam, Uttar Pradesh.

**Habit.** Perennial.

**Remarks. **Record for Uttar Pradesh fide Naithani (1990).

**931. **Cymbopogon pendulus** (Nees) Will.Watson in Atkins., Bot. Himalayan Distr. N. W. Prov. 392. 1882 **

*Andropogon pendulus* Nees, Syn. Pl. Glumac. (Steudel) 1: 388. 1854. Type: Nepal

**Distribution.** Assam [Bhutan, China, Nepal].

**Habit.** Perennial.

**932. **Cymbopogon polyneuros** (Steud.) Stapf, Bull. Misc. Inform. Kew 1906: 345, 361. 1906 **

*Andropogon polyneuros* Steud., Syn. Pl. Glumac. 1: 385. 1854. Type: India: Tamil Nadu, Coimbatore, Nilgiri Hills; *R.F. Hohenacker 933* (L).

*Andropogon schoenanthus* L. var. *versicolor* (Steud.) Hack., Monogr. Phan. [A. DC. & C. DC.] 6: 610. 1889

*Andropogon versicolor* Steud., Syn. Pl. Glumac. 1: 388. 1854. Type: East Indies.

*Cymbopogon versicolor* (Steud.) J.F. Watson in Atkins., Bot. Himalayan Distr. N. W. Prov. 392. 1882

**Distribution.** Karnataka, Tamil Nadu [Myanmar, Sri Lanka].

**Habit.** Perennial.

**933. **Cymbopogon pospischilii** (K. Schum.) C.E. Hubb., Kew Bull. 1949: 175. 1949 **

*Andropogon pospischilii* K. Schum. in Engl., Bot. Jahrb. Syst. 24: 328. 1897. Type: Mozambique: Muani; February; *Pospischil s.n.*(B).

*Andropogon nardus* L. var. *stracheyi* Hook. f., Fl. Brit. India (J.D. Hooker). 7(21): 207. 1896. Syntypes: India: Kumaon; *R. Strechey & J.E. Winterbottom s.n.* as *A. martini*. Kalenath, alt. 6000 ft.; *R. Strechey & J.E. Winterbottom s.n.* Pangi; *Stolizka s.n.*

*Cymbopogon stracheyi* (Hook. f.) Raizada & S.K. Jain, Indian Forester 80(1): 44. 1954

**Distribution.** Himachal Pradesh, Uttarakhand [Nepal, Pakistan].

**Habit.** Perennial.

**934. **Cymbopogon ramnagarensis** Gupta, Proc. Ind. Acad. Sci. Sec. B, 71:86. 1970 **

**Type.** B. K. Gupta 30; India (Dehra Dun).

**Distribution.** Jammu and Kashmir.

**Habit.** Perennial.

**Remarks. **Soenarko (1977) suggests that this species is a hybrid and should be known as *C. x stracheyi* (Hook.f.) Raizada & S.K. Jain.

**935. **Cymbopogon traninhensis** (A. Camus) Soenarko, Reinwardtia 9(3): 347. 1977 **

*Cymbopogon confertiflorus* (Steud.) Stapf var. *traninhensis* A. Camus, Bull. Mus. Natl. Hist. Nat. 26: 565. 1920. Type: Laos: Traninh “Spontane sur les hauts plateaux de Tra ninh”; *Mieville s.n.* (P).

*Cymbopogon khasianus* (Hack.) Stapf ex Bor var. *nagensis* Bor, J. Bombay Nat. Hist. Soc. 52: 169. 1954. Type: India: Assam, Naga Hills, Shiloi Jopi, alt. 8000 ft.; November, 1935; *N.L. Bor 10* (K).

**Distribution.** Assam, Nagaland [China, Myanmar].

**Habit. **Perennial.

**936. **Cymbopogon travancorensis** Bor, J. Bombay Nat. Hist. Soc. 52: 174. 1954 **

**Type.** India: Travancore: Courtallam, Tennevelly District; November 11, 1908; *Bourne 5309* (K).

**Distribution. **Kerala, Tamil Nadu.

**Habit.** Perennial.

**937. **Cymbopogon winterianus** Jowitt, Ann. Roy. Bot. Gard. (Peradeniya) 4: 188. 1908 **

**Type.** Sri Lanka: Pillagoda Valley, Buddegama S. P.; February 11, 1908; *A.W. Winter s.n.* (K).

**Distribution.** Maharashtra [China, Sri Lanka]. Native to India, but also cultivated.

**Habit.** Perennial.

***Dichanthium* Willemet, Ann. Bot. (Usteri) 18: 11. 1796**

**Type. ***Dichanthium nodosum* Willemet, nom. illeg. (*Andropogon annulatus* Forssk. (as “annulatum”), *Dichanthium annulatum* (Forssk.) Stapf.

**938. **Dichanthium annulatum** (Forssk.) Stapf, Fl. Trop. Afr. [Oliver et al.] 9: 178. 1917 **

*Andropogon annulatus* Forssk., Fl. Aegypt.-Arab. 173. 1775 (as “annulatum”). Type: “Passim ad ripas Nili.” Egypt: Rashid, banks of Nile River; June, 1762; *Forsskal 127* (C).

*Lepeocercis annulata* (Forssk.) Nees, Fl. Afr. Austral. Ill. 98. 1841

*Andropogon garipensis* Steud., Syn. Pl. Glumac. 1: 379. 1854. Type: “Ad fluv. Garip. Afr. austr.” India; *Buchanan-Hamilton s.n.* (Numer. List [Wallich] no. 8810C)

*Andropogon obtusus* Nees ex Hook. & Arn., Bot. Beechey Voy. 6: 243. 1838 (as “obtuso”). Type: Not given.

*Andropogon scandens* Roxb., Fl. Ind. (Carey and Wallich ed.) 1: 262. 1820. Type: India: “Grows commonly in hedges.”

*Andropogon comosus* sensu Link, Hort. Berol. [Link] 1: 239. 1827, non Spreng., 1819

**Distribution.** Andhra Pradesh, Assam, Bihar, Chhattisgarh, Dadra and Nagar Haveli, Delhi, Goa, Gujarat, Jammu and Kashmir, Karnataka, Kerala, Madhya Pradesh, Maharashtra, Manipur, Meghalaya, Nagaland, Odisha, Rajasthan, Tamil Nadu, Uttar Pradesh, Uttarakhand, West Bengal [Bhutan, China, Myanmar, Nepal, Pakistan, Sri Lanka].

**Habit.** Perennial.

**939. **Dichanthium aristatum** (Poir.) C.E. Hubb., Bull. Misc. Inform. Kew 1939: 654. 1939 **

*Andropogon aristatus* Poir., Encycl. (Lamarck) Suppl. 1: 585. 1811 (as “aristatum”). Type: “Cette plante a été observée a l’lle de-France par Commerson. (Herb. Desfont.)”

*Andropogon mollicomus* Kunth, Révis. Gramin. 1: 365 t. 96. 1830. Type: “Crescit in insulis Franciae et Timor.”

*Lepeocercis mollicoma* (Kunth) Nees, Edinburgh New Philos. J. 18: 185. 1835

*Andropogon caricosus* L. var. *mollicomus* (Kunth) Hack., Monogr. Phan. [A. DC. & C. DC.] 6: 569. 1889

*Diplasanthum lanosum* Desv., Opusc. Sci. Phys. Nat. 67. t. 5. f. 1. 1831. Type: “Habitat in India orientali.”

*Andropogon nodosus* Nash, N. Amer. Fl*.* 17(2): 122. 1912, nom. superfl. & illeg.?

*Dichanthium nodosum* Willemet, Ann. Bot. (Usteri). 18: 11. 1796, nom. superfl. & illeg. for *Andropogon annulatum* Forssk.

**Distribution.** Andhra Pradesh, Chhattisgarh, Karnataka, Madhya Pradesh, Maharashtra, Tamil Nadu [China].

**Habit.** Perennial.

**940. **Dichanthium armatum** (Hook. f.) Blatt. & McCann, J. Bombay Nat. Hist. Soc. 32(2): 357. 1927 (as “Dicanthio armato”) **

*Andropogon armatus* Hook. f., Fl. Brit. India (J.D. Hooker). 7(21): 197. 1896. Type: India: Concan; *J.E. Stocks s.n.*

**Distribution.** Maharashtra.

**Habit.** Annual.

**941. **Dichanthium caricosum** (L.) A. Camus, Bull. Mus. Natl. Hist. Nat. 27: 549. 1921, nom. cons. **

*Andropogon caricosus* L., Sp. Pl., ed. 2. 2: 1480. 1763 (as “caricosum”), nom. cons. Type: “Habitat in India.”

*Andropogon filiformis* Pers., Syn. Pl. (Persoon) 1: 103. 1805 (as “filiforme”)

*Heteropogon concinnus* Thwaites, Enum. Pl. Zeyl. (Thwaites). 368. 1864. Type: Sri Lanka: Badulla District, Bibili, at no great elevation; *Thwaites Ceylon Plant 3556*

*Andropogon serratus* Retz., Observ. Bot. (Retzius) 5: 21. 1788 (“1789”), (as “serratum”) non Thunb., 1784. Type: “Crescit ad oras fluminis in Bengalia, Cel Koening.”

**Distribution.** Andaman and Nicobar Islands, Andhra Pradesh, Assam, Bihar, Chhattisgarh, Gujarat, Jharkhand, Karnataka, Kerala, Madhya Pradesh, Maharashtra, Manipur, Odisha, Punjab, Rajasthan, Tamil Nadu, Uttar Pradesh, West Bengal [China, Myanmar, Sri Lanka].

**Habit.** Perennial.

**942. **Dichanthium concanense** (Hook. f.) S.K. Jain & Deshp., Bull. Bot. Surv. India 20(1–4): 134. 1979 (“1978”) (as “concanensis”) **

*Andropogon concanensis* Hook. f., Fl. Brit. India (J.D. Hooker). 7(21): 174. 1896. Type: India: Maharashtra, Bombay; *Woodrow 28* (K).

*Bothriochloa concanensis* (Hook. f.) Henrard, Blumea 3(3): 457. 1940

*Amphilophis concanensis* (Hook. f.) Blatt. & McCann, J. Bombay Nat. Hist. Soc. 32: 422. 1928

**Distribution.** Karnataka, Maharashtra.

**Habit.** Perennial.

**943. **Dichanthium foulkesii** (Hook. f.) S.K. Jain & Deshp., Bull. Bot. Surv. India 20(1–4): 134. 1979 (“1978”) **

*Andropogon foulkesii* Hook. f., Fl. Brit. India (J.D. Hooker). 7(21): 174. 1896. Syntypes: India: Nilghiri Hills; *Wight s.n.* Kaity; *Foulkes s.n.*(K).

*Bothriochloa foulkesii* (Hook. f.) Henrard, Blumea 3(3): 457. 1940

*Amphilophis foulkesii* (Hook. f.) C.E.C. Fisch., Fl. Madras 3(10): 1732. 1934

**Distribution.** Karnataka, Kerala, Tamil Nadu.

**Habit.** Perennial.

**944. **Dichanthium jainii** (Deshp. & Hemadri) Deshp., Bull. Bot. Surv. India 21(1–4): 198. 1981 (“1979”) **

*Bothriochloa jainii* Deshp. & Hemadri, Indian Forester 97(10): 593. 1971. Type: India: Maharashtra, Poona District, Junnar, Dhak Khilla; October 29, 1964; *K. Hemadri 104241A* (CAL).

**Distribution.** Maharashtra.

**Habit.** Perennial.

**945. **Dichanthium mccannii** Blatt. & McCann, J. Bombay Nat. Hist. Soc. 32: 357. 1927 **

**Type.** India: Bombay Presidency, Satara District, Panchgani; October, 1925; *C. McCann s.n. *(BLAT).

**Distribution.** Maharashtra.

**Habit.** Annual.

**946. **Dichanthium oliganthum** (Hochst. ex Steud.) Cope, Kew Bull. 35(3): 703. 1980 **

*Andropogon oliganthus* Hochst. ex Steud., Syn. Pl. Glumac. 1: 368. 1854. Type: India: Tamil Nadu, Nilgiri Mountains; *Metz, ed R.F. Hohenacker Herb. Ind. no. 1288* (K).

*Heteropogon oliganthus* (Hochst. ex Steud.) Blatt. & McCann, J. Bombay Nat. Hist. Soc. 32: 623. 1928

*Indochloa oligantha* (Hochst. ex Steud.) Bor, Kew Bull. 1954: 79. 1954

**Distribution.** Karnataka, Kerala, Maharashtra, Tamil Nadu.

**Habit.** Annual.

**947. **Dichanthium pallidum** (Hook. f.) Stapf ex C.E.C. Fisch., Fl. Madras 3(10): 1741. 1934 **

*Apocopis pallida* Hook. f., Fl. Brit. India (J.D. Hooker). 7(21): 143. 1896. Type: India: Tamil Nadu, Nilghiri Hills; *Foulkes s.n.*

**Distribution.** Tamil Nadu.

**Habit.** Perennial.

**948. **Dichanthium panchganiense** Blatt. & McCann, J. Bombay Nat. Hist. Soc. 32: 357. t. 1. 1927 **

**Type.** India: Bombay Presidency, Satara District, Panchgani; November, 1925; *C. McCann s.n. *(BLAT).

**Distribution.** Maharashtra.

**Habit.** Annual.

**949. **Dichanthium paranjpyeanum** (Bhide) Clayton, Kew Bull. 32(3): 579. 1978 **

*Andropogon paranjpyeanus* Bhide, J. Proc. Asiat. Soc. Bengal, n.s. 7: 514. 1911. Type: India: Bombay, Castle Rock; *Bhide s.n.* (K, isotype).

*Eremopogon paranjpyeanus* (Bhide) Blatt. & McCann, J. Bombay Nat. Hist. Soc. 32: 427. 1928 (as “paranjpyeanum”)

*Schizachyrium paranjpyeanum* (Bhide) Raizada & Jain, Proc. Indian Sci. Congr. Assoc. 3: 130. 1953

**Distribution.** Karnataka, Maharashtra.

**Habit.** Annual.

**Remarks.** Record for Karnataka fide Naithani (1990).

**950. **Dichanthium tuberculatum** (Hack.) Cope, Kew Bull. 35(3): 703. 1980 **

*Andropogon tuberculatus* Hack., Monogr. Phan. [A. DC. & C. DC.] 6: 404. 1889. Type: India: Assirgar;* O. Kuntze s.n.* (W).

*Eremopogon tuberculatus* (Hack.) A. Camus, Ann. Soc. Linn. Lyon ser. 2, 68: 208. 1921

**Distribution.** Andhra Pradesh, Madhya Pradesh, Maharashtra.

**Habit.** Perennial.

***Diectomis* Kunth, Mém. Mus. Hist. Nat. 2: 69. 1815, nom. cons.**

**Type. ***Diectomis fastigiata* (Sw.) P. Beauv. (*Andropogon fastigiatus* Sw. (as “fastigiatum”)).

**951. **Diectomis fastigiata** (Sw.) P. Beauv., Ess. Agrostogr. 132, 150, 160. 1812 **

*Andropogon fastigiatus* Sw., Prodr. (Swartz) 26. 1788 (as “fastigiatum”). Type: Jamaica; *Swatrz s.n.*

*Pollinia fastigiata* (Sw.) Spreng., Pl. Min. Cogn. Pug. 2: 13. 1815

*Andropogon diatherus* Steud., Syn. Pl. Glumac. 1: 378–379. 1854. Type: Oaxaca

*Andropogon hochstetteri* Steud., Syn. Pl. Glumac. 1: 384. 1854. Type: Abyssinia; *Schimper 2013* (P).

*Heteropogon hochstetteri* (Steud.) Andersson ex Schweinf., Beitr. Fl. Aethiop. 306. 1867

**Distribution.** Andhra Pradesh, Bihar, Chhattisgarh, Jharkhand, Madhya Pradesh, Rajasthan, West Bengal [Myanmar].

**Habit.** Perennial.

***Dimeria* R. Br., Prodr. Fl. Nov. Holland. 204. 1810**

**Type. ***Dimeria acinaciformis* R. Br.

**952. **Dimeria agasthyamalayana** Kiran Raj & Ravi, Rheedea 11(2): 93. 2001 **

**Type.** India: Kerala, Thiruvananthapuram District, Agasthyamala Hills, Bonaccord, alt. 2500 ft.; February 1, 2000; *Kiran Raj 41939* (TBGT).

**Distribution.** Kerala, Tamil Nadu.

**Habit.** Annual.

**953. **Dimeria andamanica** Gosavi, M.Y. Kamble, Chandore & S.R. Yadav, Phytotaxa 270(4): 296. 2016. **

**Type.** India, Andaman and Nicobar Islands, South Andaman, Chidiya Tapu, 28 m, 12 December 2014, *M. Y. Kamble 32185* (holotype: CAL, isotype: PBL, SUK).

**Distribution.** Andaman Islands.

**Habit.** Annual.

**954. **Dimeria aristata** (Hack.) Senaratna, Grasses Ceylon (Perad. Man., No. 8) 163. 1956 **

*Dimeria lehmannii* Hack. var. *aristata* Hack., Monogr. Phan. [A. DC. & C. DC.] 6: 83. 1889

**Distribution.** Orisssa, Tamil Nadu, West Bengal [Sri Lanka].

**Habit.** Perennial.

**955. **Dimeria avenacea** (Retz.) C.E.C. Fisch., Bull. Misc. Inform. Kew 1932: 72. 1932 **

*Anthoxanthum avenaceum* Retz., Observ. Bot. (Retzius) 3: 8. 1783 (as “auenaceum”). Type: “E. Tranquebaria misit cel. Koenig.”

*Dimeria avenacea* (Retz.) C.E.C. Fisch. var. *elatior* (Hook. f.) Bor, Kew Bull.1952: 562. 1952

*Dimeria elatior* (Hook. f.) Senaratna, Grasses of Ceylon 162. 1956

*Dimeria pusilla* Thwaites, Enum. Pl. Zeyl. (Thwaites). 369. 1864. Type: Sri Lanka: Kokotodua; *Gardner Ceylon Plant 954.*

*Dimeria pusilla* Thwaites var. *elatior* Hook. f., Fl. Brit. India (J.D. Hooker). 7(21): 103. 1896. Type: Sri Lanka: Trincomalee; *Glennie Ceylon Plant 957.*

*Dimeria acutipes* Bor, Kew Bull. 1952: 560. 1952. Type: India: Madras State; September, 1898; *Bourne 35* (K).

**Distribution.** Andhra Pradesh, Kerala, Odisha, Tamil Nadu [Myanmar, Sri Lanka].

**Habit.** Annual.

**956. **Dimeria balakrishnaniana** K. Ravik., Sreek. & Lakshm., Kew Bull. 45(3): 573. 1990 **

**Type.** India: Tamil Nadu, Madurai District, High Wavy’s Mountains, Nursery Valley, 4500 ft.; January 22, 1988; *Lakshmanan 87543* (CAL).

**Distribution.** Tamil Nadu.

**Habit.** Annual.

**957. **Dimeria bialata** C.E.C. Fisch., Bull. Misc. Inform. Kew 1933: 351. 1933 **

**Type.** India: Kanara District, Siradi; November; *A. Meebold 10548.*

**Distribution.** Karnataka, Kerala, Tamil Nadu.

**Habit. **Annual.

**Remarks.** Records for Karnataka and Tamil Nadu fide Naithani (1990).

**958. **Dimeria blatteri** Bor, Kew Bull. 1949: 70. 1949 **

**Type.** India: Bombay, Khandala, Echo Point; October, 1918; *Blatter & McCann s.n. *(BLAT).

**Distribution.** Maharashtra.

**Habit.** Annual.

**959. **Dimeria chelariensis** Ravi, Rheedea 5(1): 37. f. 1. 1995 **

**Type.** India: Kerala, Malappuram District, Chelari; November 28, 1992; *N. Ravi 3606* (TBGT).

**Distribution.** Kerala.

**Habit.** Perennial.

**960. **Dimeria connivens** Hack., Monogr. Phan. [A. DC. & C. DC.] 6: 689. (add.) 1889 **

**Type.** India orientalis, loco speciali collectoreque ignoto; *T. Anderson s.n.*

**Distribution.** Bihar, Chhattisgarh, Jharkhand, Kerala, Madhya Pradesh, Maharashtra, Odisha, Rajasthan.

**Habit.** Annual.

**961. **Dimeria copeana** Sreek., V.J. Nair & N.C. Nair, J. Bombay Nat. Hist. Soc. 78: 577. f. 9. 1981 **

**Type.** India: Kerala, Alleppey District, Thrikkunna Puzha; March 13, 1980; *P.V. Sreekumar* 66736 (CAL).

**Distribution.** Kerala.

**Habit. **Perennial.

**962. **Dimeria copei** Ravi, Blumea 41(1): 251. 1996 **

**Type.** India: Kerala, Alappuzha District, Kalavoor; December 3, 1992; *Ravi 3655* (TGBT).

**Distribution.** Kerala.

**Habit.** Annual.

**963. **Dimeria deccanensis** Bor, Kew Bull. 1952: 578. 1952 **

**Type.** India: Madras State, Pilicode, S. Kanara; November 11, 1917; *15340 ex Madras Herb.* (K).

**Distribution.** Karnataka, Kerala, Maharashtra, Tamil Nadu.

**Habit.** Annual.

**Remarks.** Records for Kerala and Tamil Nadu fide Naithani (1990).

**964. **Dimeria eradii** Ravi, Rheedea 5(1): 39. f. 2. 1995 **

**Type.** India: Kerala, Malppuram Distrrict, Tenhipa’am; November 29, 1992; *N. Ravi 3641* (TBGT).

**Distribution.** Kerala.

**Habit. **Annual.

**965. **Dimeria falcata** Hack., Monogr. Phan. [A. DC. & C. DC.] 6: 85. 1889 **

**Distribution.** North East India [China, Myanmar].

**Habit.** Perennial.

**Remarks.** Distribution in India not reported.

**966. **Dimeria fasciculata** P. Kumari & R. Lakra, Int. J. Adv. Res. 6(7): 484–490. 2018. **

**Type.** India: Andaman Islands, Middle Andaman, Panchvati, 08 February 2017, *Reshma Lakra32835* (holotype: CAL; isotype: PBL).

**Distribution.** Andaman Islands.

**Habit.** Annual.

**967. **Dimeria fischeri** Bor, Kew Bull. 1952: 564. 1952 **

**Type.** India: Madras State, Mahendragiri; *C.E.C. Fischer 133* (K).

**Distribution.** Andhra Pradesh, Kerala, Tamil Nadu.

**Habit.** Annual.

**Remarks.** Record for Tamil Nadu fide Naithani (1990).

**968. **Dimeria fuscescens** Trin., Mém. Acad. Imp. Sci. St.-Pétersbourg, Sér. 6, Sci. Math. 2: 335. 1832 **

**Type. **Nepal; Numer. List [Wallich] no. 8841.

**Distribution.** Assam, Kerala, Meghalaya, Maharashtra, Nagaland, Tamil Nadu [Myanmar, Nepal, Sri Lanka].

**Habit.** Perennial.

**969. **Dimeria gracilis** Nees, Syn. Pl. Glumac. (Steudel) 1: 413. 1855 **

*Dimeria laxiuscula* Thwaites & Trimen, J. Bot. 23: 272. 1885. Type: Sri Lanka: Pasdun Korle; September, 1864; *Thwaites Ceylon Plant 3863 in Herb. Perad.*; Kulutara; December, 1882

*Dimeria leptorhachis* Hack., Monogr. Phan. [A. DC. & C. DC.] 6: 89. 1889. Type: Sri Lanka; *Thwaites 3261*

*Dimeria pilosissima* (J. Presl) Thwaites, Enum. Pl. Zeyl. (Thwaites). 369. 1864, non Trin., 1832, isonym

**Type.** Sri Lanka; *Lindley s.n.*

**Distribution.** Karnataka, Maharashtra, Tamil Nadu [Sri Lanka].

**Habit.** Perennial.

**970. **Dimeria hohenackeri** Hochst. ex Miq., Nieuwe Verh. Eerste Kl. Kon. Ned. Inst. Wetensch. Amsterdam ser. 3, 4: 35. 1851 **

*Psilostachys hohenackeri* Steud., Syn. Pl. Glumac. 1: 413. 1855. Type: India; *R.F. Hohenacker 231*

*Arthraxon hohenackeri* Hochst. ex Steud., Syn. Pl. Glumac. 1: 413. 1855, pro syn., nom. inval.

**Type.** India: Manglore; *Metz 231b.*

**Distribution.** Karnataka, Kerala, Maharashtra.

**Habit.** Annual.

**971. **Dimeria idukkiensis** Ravi & N. Anilkumar, Rheedea 2(2): 104. 1992 **

**Type.** India: Kerala, Idukki District, Peermedu, Kuttikkanam; October 19, 1991; *Anil Kumar 3190* (MH).

**Distribution.** Kerala.

**Habit.** Annual.

**972. **Dimeria jainii** Sreek., V.J. Nair & N.C. Nair, Curr. Sci. 52(6): 259. f. 1. 1983 **

**Type.** India: Kerala, Calicut District, Pokkunnamalai, Nanminda, alt. 2700 ft.; October 29, 1981; *P.V. Sreekumar 71814* (CAL).

**Distribution.** Kerala.

**Habit.** Annual.

**973. **Dimeria jayachandranii** Arisdason & P. Daniel, Kew Bull. 64(2): 345, f. 1. 2009. **

**Type.** India: Tamil Nadu, Coimbatore District, Anamalais hills, Karian Shola, 800–1000 m, 5 December 2002, *Arisdason 115578* (holotype: CAL; isotype: MH).

**Distribution.** Tamil Nadu.

**Habit. **Annual.

**974. **Dimeria josephii** Ravi & N. Mohanan, Rheedea 11(2): 90–92. f. 2. 2001 **

**Type.** India: Kerala, Palakkad District, Nenmara, 250 ft.; November 24, 1998; *Ravi 39542* (TBGT).

**Distribution.** Kerala.

**Habit.** Annual.

**975. **Dimeria kalavoorensis** Ravi, Blumea 41(1): 251. 1996 **

**Type.** India: Kerala, Alappuzha District, Kalavoor; December 3, 1992; *Ravi 3654* (TGBT).

**Distribution.** Kerala.

**Habit.** Annual.

**976. **Dimeria kaleri** P. Biju, Josekutty & Augustine, Bangladesh J. Pl. Taxon. 25(1): 14. 2018. **

**Type.** India, Kerala, Kasaragod district, Karakkode lateritic plateau, 75 m, 12 December, *Biju &Jomy 1166* (holotype: CAL; isotype: MH).

**Distribution.** Kerala.

**Habit.** Not mentioned.

**977. **Dimeria kalliyadense** Biju, Josekutty & Augustine, Int. J. Adv. Res. 5(11): 705–711. 2017. **

**Type.** India: Kerala, Kannur district, Kalliyad lateritic plateau, 190 m, 14 September 2013, *Biju &Jomy 2468* (holotype: CAL, isotype: MH).

**Distribution.** Kerala.

**Habit.** Annual.

**978. **Dimeria kanjirapallilana** Jacob, J. Bombay Nat. Hist. Soc. 47: 48. 1947 (as “kanijirapallilana”) **

**Type.** South India, Travancore, Peermade, alt. 3200 ft.; December, 1941; *K.C. Jacob s.n.* (MH-86320).

**Distribution.** Andhra Pradesh, Kerala, Tamil Nadu.

**Habit.** Annual.

**Remarks.** Record for Kerala fide Naithani (1990).

**979. **Dimeria keralae** N.C. Nair, Sreek. & V.J. Nair, J. Bombay Nat. Hist. Soc. 80: 626. f. 1–16. 1984 **

**Type.** India: Kerala, Cannanore District, Paramba, Bandudka, alt. 500 ft.; October 16, 1981; *P.V. Sreekumar 71717* (CAL).

**Distribution.** Kerala.

**Habit.** Annual.

**980. **Dimeria kollimalayana** M. Mohanan & A.V.N. Rao, J. Bombay Nat. Hist. Soc. 80: 617. f. 1–10. 1984 **

**Type.** India: Tamil Nadu, Salem District, Kollimalai, alt. 3900; February 19, 1982; *M. Mohanan 56802* (CAL).

**Distribution.** Karnataka, Kerala, Odisha, Tamil Nadu.

**Habit.** Annual.

**981. **Dimeria kurzii** Hook. f., Fl. Brit. India 7: 103. 1896 **

**Type.** Myanmar: Pegu, 27 December 1870, *S. Kurz 2741* (K).

**Distribution.** Kerala, Nagaland.

**Habit.** Annual.

**982. **Dimeria lawsonii** (Hook. f.) C.E.C. Fisch., Fl. Madras 3(10): 1713. 1934 **

*Dimeria pusilla* Thwaites var. *lawsonii* Hook. f., Fl. Brit. India (J.D. Hooker). 7(21): 103. 1896. Type: India: Malabar, Wynaad; *Lawson s.n.*

**Distribution.** Karnataka, Kerala, Tamil Nadu.

**Habit.** Annual.

**983. **Dimeria lehmannii** (Nees) Hack., Monogr. Phan. [A. DC. & C. DC.] 6: 82. 1889 (as “lehmanni”) **

*Pterygostachyum lehmannii* Nees, Syn. Pl. Glumac. (Steudel) 1: 413. 1855 (as “lehmanni”). Type: India

*Dimeria lehmanii* (Nees) Hack. var. *mutica* Hack., Monogr. Phan. [A. DC. & C. DC.] 6: 83. 1889. Type: Sri Lanka; *Thwaites Ceylon Plant 955*

*Dimeria alata* Hook. f., Fl. Brit. India (J.D. Hooker). 7(21): 105. 1896. Type: Sri Lanka; 1864; *Thwaites Ceylon Plant 955* (K).

**Distribution.** Andhra Pradesh, Tamil Nadu [Sri Lanka].

**Habit.** Perennial.

**984. **Dimeria mahendragiriensis** Ravi, H.O. Saxena & Brahmam, Rheedea 5(2): 142. 1995 **

**Type.** India: Odisha, Gajapati District, Mahendragiri hill, 1350 m, 22 November 1979, *H. O Saxena & M. Brahmam 3880* (holotype: RRLB).

**Distribution.** Odisha.

**Habit.** Perennial

**985. **Dimeria mooneyi** Raizada ex Mooney, Suppl. Bot. Bihar Orissa 263. 1950 **

*Dimeria borii* Sreek., V.J. Nair & N.C. Nair, J. Econ. Taxon. Bot. 3(2): 657. f. 1. 1982. Type: India: Kerala, Calicut District, Kanjeerakkadavu, near Kakkayam, alt. 3000 ft.; November 26, 1981; *P.V. Sreekumar 71855* (CAL).

**Type.** India: Orissa, Sambalpur District, close to Sonabera Village, alt. 2300 ft.; *Mooney 3652 & 3657* (K, DD).

**Distribution.** Karnataka, Kerala, Odisha, Tamil Nadu.

**Habit.** Annual or perennial.

**Remarks.** Records for Karnataka and Tamil Nadu fide Naithani (1990).

**986. **Dimeria namboodiriana** Ravi & N. Mohanan, Rheedea 7(1): 1. 1997. **

**Type.** India: Kerala, Pathanamthitta District, Ponnambalamedu, 5 January 1996, *Ravi 24050* (holotype: TBGT, iso: K, KFRI, L, MH).

**Distribution.** Kerala.

**Habit.** Perennial.

**987. **Dimeria orissae** Bor, Kew Bull. 1952: 579. 1952 **

**Type.** India: Orissa, Pipokhuri, Keonjhar State, alt. 3200 ft.; October 1, 1946; *H.F. Mooney 2758* (K).

**Distribution.** Odisha, Tamil Nadu.

**Habit.** Annual.

**988. **Dimeria ornithopoda** Trin., Fund. Agrost. (Trinius) 167. t. 14. 1820 var. **ornithopoda

*Andropogon roxburghianus* Schult., Mant. 2 (Schultes) 451. 1824. Type: India: Calcutta; *Roxburgh s.n.*

*Andropogon stipiformis* Steud., Syn. Pl. Glumac. 1(4–5): 377. 1854. Japon. ex Hrbo. Mus. Lug. Batav.

*Dimeria stipiformis* Miq., Prolus. Fl. Japon. 176. 1866 (as “*stipaeformis*”)

*Dimeria filiformis* Hochst. ex Miq., Nieuwe Verh. Eerste Kl. Kon. Ned. Inst. Wetensch. Amsterdam ser. 3, 4: 35. 1851, nom. superfl. for *Andropogon roxburghianus *Schult.

*Psilostachys filiformis* Dalzell in Dalzell & A. Gibson, Bombay Fl. 305. 1861 nom. superfl. for *Andropogon roxburghianus *Schult.

*Andropogon filiformis* Roxb., Fl. Ind. (Carey & Wallich ed.) 1: 260. 1820, non Pers., 1805

**Type.** India orientali.

**Distribution.** Andhra Pradesh, Bihar, Chhattisgarh, Goa, Gujarat, Jharkhand, Karnataka, Kerala, Madhya Pradesh, Maharashtra, Meghalaya, Odisha, Rajasthan, Tamil Nadu, West Bengal [China, Myanmar, Nepal].

**Habit.** Annual.

**989. **Dimeria ornithopoda** Trin. var. **gracillima** Bor, Kew Bull. 1952: 576. 1952 **

**Syntypes.** India: Bihar, Parasnath, alt. 4000 ft.; October 1, 1873; *C.B. Clarke 21084B*. Ibidem, alt. 4500 ft.; October 1, 1833; *C.B. Clarke 33719A &C.*

**Distribution.** Andhra Pradesh, Bihar, Karnataka, Madhya Pradesh, Maharashtra, Tamil Nadu.

**Habit.** Annual.

**990. **Dimeria ornithopoda** Trin. var. **khasiana** Bor, Kew Bull. 1952: 576. 1952 **

**Syntypes.** India: Assam, Khasia Hills, Cherra, alt. 4000 ft.; October 6, 1867; *C.B. Clarke 5625*. Ibidem, Mamloo, 4000 ft.; September 24, 1886; *C.B. Clarke 45095A*. Ibidem; *Griffith s.n.*

**Distribution.** Andhra Pradesh, Assam, Chhattisgarh, Karnataka, Maharashtra, Meghalaya, Tamil Nadu.

**Habit. **Annual.

**Remarks.** Records for Karnataka, Maharashtra, and Meghalaya fide Naithani (1990).

**991. **Dimeria ornithopoda** Trin. var. **megalantha** Bor, Kew Bull. 1952: 576. 1952 **

**Type.** India: Maharashtra, Bombay, Belgaum; May 25, 1914; *Anonymous no. 14.*

**Distribution.** Andhra Pradesh, Karnataka (Naithani), Madhya Pradesh, Maharashtra, Tamil Nadu.

**Habit.** Annual.

**Remarks.** Record for Karnataka fide Naithani (1990).

**992. **Dimeria pubescens** Hack., Monogr Phan. 6: 83. 1889 **

*Dimeria ceylanica* Bor, Kew Bull. 1952: 562. 1952. Type: Sri Lanka; *Gardner 1008, Thwaites 956 p.p.* (K, DD).

*Dimeria kurumthotticalana* Jacob, J. Bombay Nat. Hist. Soc. 47: 49. 1947. Type: India: Travancore, Peermade, alt. 3200; December, 1941; *K.C. Jacob s.n.* (MH- 86320A).

*Dimeria trimenii* Hook. f. in Trimen, Handb. Fl. Ceylon 5: 198. 1900. Syntypes: Sri Lanka: Elpiliya Patana; *Pearson s.n.*, Unknown locality; *Trimen s.n*

**Type.** C. P. Thwaites 956 ex p., “in insula Ceylon”.

**Distribution.** Karnataka, Kerala, Odisha, Tamil Nadu [Sri Lanka].

**Habit.** Perennial.

**Remarks.** Records for Karnataka, Odisha and Tamil Nadu fide Naithani (1990).

**993. **Dimeria raizadae** V.J. Nair, Sreek. & N.C. Nair, Indian J. Forest. 6(2): 163. f. 1–8. 1983 **

**Type.** India: Kerala, Calicut District, Pokkunnamalai, near Nanminda, alt. 2700 ft.; October 29, 1981; *P.V. Sreekumar 71812* (CAL).

**Distribution. **Kerala.

**Habit.** Annual.

**994. **Dimeria raviana** Kiran Raj & Sivad., Phytotaxa 195(2): 193. 2015 **

**Type.** India: Kerala, Idukki district, Periyar Tiger Reserve, 950 m, *Jomy s.n.* (holotype: CAL, isotype: KFRI).

**Distribution.** Kerala.

**Habit.** Perennial.

**995. **Dimeria santapaui** M. R. Almeida, J. Bombay Nat. Hist. Soc. 66: 510. f. A–I. 1970 **

**Type. **India: North Kanara, Mirjan flats; October, 1919; *Sedgwick & Bell 6875* (BLAT).

**Distribution.** Karnataka, Maharashtra.

**Habit.** Annual.

**996. **Dimeria sivarajanii** N. Mohanan & Ravi, Rheedea 6(2): 47. f. 1. 1996 **

**Type.** India: Kerala, Pathanamthitta District, Kochu Pampa Hills; January 4, 1996; *Ravi TBG & RI 24041* (TBGT).

**Distribution.** Kerala.

**Habit.** Annual.

**997. **Dimeria sreenarayanae** Ravi & N. Anilkumar, Rheedea 2: 101. 1992 (as “sreenarayanii”) **

**Type.** India: Kerala, Idukki District, Peermudu, Kuttikkanam; December 30, 1991; *Anil Kumar 3297* (MH).

**Distribution.** Kerala.

**Habit.** Annual.

**998. **Dimeria stapfiana** C.E. Hubb. ex Pilg., Nat. Pflanzenfam., ed. 2 [Engler & Prantl] 14e: 109. 1940 **

*Woodrowia diandra* Stapf, Hooker’s Icon. Pl. 25: t. 2447. 1896. Type: India: Poona District, Mawal; *Woodrow s.n.*

*Dimeria diandra* (Stapf) Stapf ex Bhide, J. Proc. Asiat. Soc. Bengal (n.s.) 7(8): 516. 1912 (“1911”), non Griff., 1851

**Type.** “Zierliche, meist einjahrige Graser, Blatter nach oben zu am Halm an Grosse abnehmend.”

**Distribution.** Goa, Karnataka, Maharashtra.

**Habit. **Annual.

**999. **Dimeria thwaitesii** Hack., Monogr. Phan. [A. DC. & C. DC.] 6: 78. 1889 **

*Dimeria pusilla* Thwaites var. *pallida* Hook. f., Fl. Brit. India (J.D. Hooker). 7(21): 103. 1896. Type: Sri Lanka: Damboul; *Thwaites Ceylon Plant 957 *(p.p.).

**Type.** Sri Lanka: Dambool; *Thwaites Ceylon Plant 3965*.

**Distribution.** Kerala, Tamil Nadu [Sri Lanka].

**Habit. **Annual.

**1000. **Dimeria veldkampii** Kiran Raj & Sivad., Novon 18(2): 183, f. 1. 2008. **

**Type.** India: Goa, North Goa district, Taleigao, 50 m, 27 October 2002, M. S. Kiran Raj 81073 (holotype: CALI, isotype: MH, MO).

**Distribution.** Goa.

**Habit.** Annual.

**1001. **Dimeria woodrowii** Stapf, Hooker’s Icon. Pl. 24: t. 2312. 1894 **

**Type.** India: Rutnagherry District, Bombay, near Goa; *Woodrow s.n.*

**Distribution.** Goa, Karnataka, Maharashtra.

**Habit.** Annual.

Elionurus **Humb. & Bonpl. ex Willd., Sp. Pl., ed. 4 [Willdenow] 4(2): 941. 1806 (as “Elyonurus”), nom. & orth. cons. **

**Type. ***Elionurus tripsacoides* Humb. & Bonpl. ex Willd.

**1002. **Elionurus royleanus** Nees ex A. Rich., Tent. Fl. Abyss. 2: 471. 1850–1851 **

**Type.** “Crescit in declivitatibus ad fluvii Tacazze ripam sinistram (Schimper).” Ethiopia: Tacazze; *Schimper 795* (P?).

**Distribution.** Bihar, Delhi, Gujarat, Maharashtra, Uttar Pradesh [Pakistan].

**Habit.** Annual.

***Eremochloa* Buse, Gram. (Buse) 17. 1854**

**Type. ***Eremochloa horneri* Buse,

**1003. **Eremochloa muricata** (Retz.) Hack., Monogr. Phan. [A. DC. & C. DC.] 6: 262. 1889 **

*Aegilops muricata* Retz., Observ. Bot. (Retzius) 2: 27. 1781. Type: “Ex India Orientali diu habui pro exaltata, donec illam benevoletia Cl. D. Konig accepi.” India; *Koenig s.n.* (LD).

*Rottboellia muricata* (Retz.) Retz., Observ. Bot. (Retzius) 3: 12. 1783

*Ischaemum pectinatum* Trin., Mém. Acad. Imp. Sci. St.-Pétersbourg, Sér. 6, Sci. Math. 2: 296. 1832. Lectotype: Burma; 1826–1827; Numer. List [Wallich] no. 015 in herb. Trinius 95.3(LE). LT designated by Buitenhuis & Veldkamp in Blumea 46(2): 410. 2001.

*Andropogon pectinatus* (Trin.) Steud., Syn. Pl. Glumac. 1: 369. 1854

**Distribution.** Andhra Pradesh, Gujarat, Maharashtra, Meghalaya, Odisha, Tamil Nadu [Myanmar, Sri Lanka].

**Habit.** Perennial.

**1004. **Eremochloa zeylanica** Hack., Monogr. Phan. [A. DC. & C. DC.] 6: 263. 1889 **

*Andropogon falcatus* Steud., Syn. Pl. Glumac. 1: 369. 1854

*Ischaemum falcatum* (Steud.) Nees ex Thwaites, Enum. Pl. Zeyl. (Thwaites). 436. 1864

*Ischaemum zeylanicum* (Hack.) Trimen, Syst. Cat. Fl. Pl. Ceylon 107. 1885. Type: Sri Lanka: Kornegala, Hill slopes, 3000–5000 ft.; September, 1862; *Ceylon Plant Thwaites 3322*

**Type.** Sri Lanka; *Thwaites 3322* (K).

**Distribution.** Andhra Pradesh, Karnataka, Maharashtra [Myanmar, Sri Lanka].

**Habit.** Perennial.

***Eremopogon* Stapf (1917), Fl. Trop. Afr. [Oliver et al.] 9: 182–183. 1917**

**Lectotype. ***Eremopogon foveolatus* (Delile) Stapf, Fl. Trop. Afr. [Oliver et al.] 9: 183. 1917 (*Andropogon foveolatus* Delile). LT designated by Maire, Fl. Afrique Nord 1: 275. 1952).

**1005. **Eremopogon foveolatus** (Delile) Stapf, Fl. Trop. Afr. [Oliver et al.] 9: 183. 1917 **

*Andropogon foveolatus* Delile, Descr. Egypte, Hist. Nat. 2: 16, t. 8, f. 2. 1813, (“1812”) (as “*foveolatum*”). Type: “Fleurit en hiver dans les vallees sablonneuses de l’isthme de Soneys.” Egypt: l’isthe de Soneys; no date;* A.R. Delile s.n.*(P).

*Dichanthium foveolatum* (Delile) Roberty, Boissiera 9: 170. 1960

**Distribution.** Andhra Pradesh, Bihar, Delhi, Gujarat, Haryana, Jammu and Kashmir, Karnataka, Madhya Pradesh, Maharashtra, Odisha, Rajasthan, Tamil Nadu, Uttar Pradesh, West Bengal [Pakistan, Sri Lanka].

**Habit.** Perennial.

***Eriochrysis* P. Beauv., Essai Agrostogr. 8. 1812**

**Type. ***Eriochrysis cayanensis* P. Beauv.

**1006. **Eriochrysis rangacharii** C.E.C. Fisch., Bull. Misc. Inform. Kew 1932: 246. 1932 **

**Type. **India: Nilgiri Hills, Pykara; June, 1900; *Sir A. G. & Lady Bourne s.n*. (K).

**Distribution.** Kerala, Tamil Nadu.

**Habit.** Perennial.

***Euclasta* Franch., Bull. Soc. Hist. Nat. Autun 8: 335. 1895**

**Type. ***Euclasta glumaceus* Franch.

**1007. **Euclasta clarkei** (Hack.) Cope, Kew Bull. 35(3): 704. 1980 **

*Andropogon clarkei* Hack., Oesterr. Bot. Z. 41: 49. 1891. Type: India: Chota Nagpur, Parasnath, alt. 4200 ft., Hazaribagh; October 7, 1883; *C.B.Clarke 33780* (K).

*Dichanthium clarkei* (Hack.) Haines, Bot. Bihar Orissa 5: 1040. 1924

*Indochloa clarkei* (Hack.) Bor, Kew Bull. 1954: 76. 1954

**Distribution.** Bihar, Maharashtra, Rajasthan.

**Habit.** Annual.

**1008. **Euclasta condylotricha** (Hochst. ex Steud.) Stapf, Fl. Trop. Afr. [Oliver et al.] 9: 181. 1917 **

*Andropogon condylotrichus* Hochst. ex Steud., Syn. Pl. Glumac. 1: 377. 1854. Type: Ethiopia: Dscheladscheranne; *Schimper 2011*(P).

*Andropogon piptatherus* Hack., Fl. Bras. (Martius) 2(3): 293. 1883. Type: “Habitat in prov. Goyaz ad Porto Imperial et ad fl. Tocantins inter Porto Imperial et Funil; *Burchell 8780*.” Brazil: Goiás; *Burchell 8761-7* (W).

*Amphilophis piptatherus* (Hack.) Nash, N. Amer. Fl. 17(2): 127. 1912

*Sorghum piptatherum* (Hack.) Kuntze, Revis. Gen. Pl. 2: 792. 1891

*Euclasta glumacea* Franch., Bull. Soc. Hist. Nat. Autun 8: 336. t. 8. 1895. Type: Congo Francais: “Comba, entre Londina et Brazzaville; *Thollon 1040.*” Congo Francais: “Lope, dans l’Ogooue”; *Thollon 787*

*Euclasta graminea* sensu T. Durand & H. Durand, Syll. Fl. Congol. 649. 1909 (as “Euclaste”), non Franch, 1895.

**Distribution.** Madhya Pradesh [also in Africa, North America, South America].

**Habit.** Annual.

**Remarks.** Record for Madhya Pradesh fide Naithani (1990). Possibly introduced.

***Eulalia* Kunth, Rév. Gram. 1: 160. 1829**

**Type. ***Eulalia aurea* (Bory) Kunth (*Andropogon aureus* Bory (“aureum”)).

**1009. **Eulalia contorta** (Brongn.) Kuntze, Revis. Gen. Pl. 2: 775. 1891 **

*Pogonatherum contortum* Brongn., Voy. Monde, Phan. 2(2): 90, t. 17. 1831. Type: “Bourou, dans les iles Moluques.”

*Pseudopogonatherum contortum* (Brongn.) A. Camus, Fl. Indo-Chine [P.H. Lecomte et al.] 7: 255. 1922

*Andropogon asthenostachys* Steud., Syn. Pl. Glumac. 1: 381. 1854. Type: Philippine Islands; *H. Cuming 1101.*

*Andropogon koretrostachys* Trin., Mém. Acad. Imp. Sci. St.-Pétersbourg, Sér. 6, Sci. Math. 2: 273. 1832. Type: Philippine Islands: Manila.

*Pseudopogonatherum koretrostachys* (Trin.) Henrard, Blumea 4(3): 521. 1941

*Eulalia koretrostachys* (Trin.) Henrard, Blumea 4(3): 521. 1941

*Pollinia articulata* Trin., Mém. Acad. Imp. Sci. St.-Pétersbourg, Sér. 6, Sci. Math. 4:90. 1836. Type: “Inss. Molucc.”

*Erianthus articulatus* (Trin.) F. Muell., Fragm. (Mueller) 8: 118. 1872–1874

*Puliculum articulatum* (Trin.) Stapf ex Haines, Bot. Bihar Orissa 5: 1018. 1924 (as “articulata”)

*Pollinia articulata* Trin. var. *pedicellata* Hack. ex Hook. f., Fl. Brit. India (J.D. Hooker). 7(21): 110. 1896. Type: India: Kumaon; *Duthei s.n.*

*Eulalia concinna* Nees, Syn. Pl. Glumac. (Steudel) 1: 412. 1854. Type: India; Numer. List [Wallich] no. 8814

*Pollinia collina* Balansa, J. Bot. (Morot) 4(4): 81. 1890. Type: Vietnam: Valleys of Tu-Phap; *Balansa 1771*

*Pseudopogonatherum collinum* (Balansa) A. Camus, Fl. Indo-Chine [P.H. Lecomte et al.] 7: 256. 1922

*Pollinia setifolia* Nees, Hooker’s J. Bot. Kew Gard. Misc. 2: 101. 1850. Type: Philippines Island; *H. Cuming 1101*

*Pseudopogonatherum setifolium* (Nees) E.G. Camus & A. Camus, Fl. Indo-Chine [P.H. Lecomte et al.] 7: 256. 1922

**Distribution.** Andhra Pradesh, Bihar, Chhattisgarh, Karnataka, Madhya Pradesh, Meghalaya, Nagaland, Sikkim, Tamil Nadu, Uttar Pradesh, Uttarakhand, West Bengal [Bhutan, China, Myanmar, Nepal].

**Habit.** Annual.

**1010. **Eulalia fastigiata** (Nees) Haines, Bot. Bihar Orissa 5: 1014. 1924 **

*Saccharum fastigiatum* Nees, Syn. Pl. Glumac. (Steudel)1: 409. 1855. Type: India; Numer. List [Wallich] no. 8847

*Erianthus fastigiatus* (Nees) Hook. f., Fl. Brit. India (J.D. Hooker). 7(21): 125. 1896

**Distribution.** Assam, Karnataka, Maharashtra, Meghalaya, Sikkim, West Bengal.

**Habit.** Perennial.

**1011. **Eulalia fimbriata** (Hack.) Kuntze, Revis. Gen. Pl. 2: 775. 1891 **

*Pollinia fimbriata* Hack., Monogr. Phan. [A. DC. & C. DC.] 6: 164. 1889. Type: India: Malabar & Concan; *J.E. Stocks s.n.*

**Distribution.** Daman and Diu, Dadra and Nagar Haveli, Goa, Himachal Pradesh, Karnataka, Maharashtra, Rajasthan [China].

**Habit.** Annual.

**1012. **Eulalia hirtifolia** (Hack.) Kuntze, Revis. Gen. Pl. 2: 775. 1891 (as “hirtiflora); A. Camus, Ann. Soc. Linn. Lyon ser. 2, 68: 202. 1921 **

*Pollinia hirtifolia* Hack., Monogr. Phan. [A. DC. & C. DC.] 6: 165. 1889. Type: India: Simla; *Hugel 758* (CAL).

*Eulalia quadrinervis* (Hack.) Kuntze var. *hirtifolia* (Hack.) Sur, J. Econ. Taxon. Bot. 6(1): 185. 1985

**Distribution.** Chhattisgarh, Himachal Pradesh, Madhya Pradesh, Uttarakhand [China].

**Habit.** Perennial.

**Remarks. **Author K. Gandhi notes that “Kuntze cited Hackel as the parenthetical author, but perhaps due to confusion, misspelled Hackel’s epithet *hirtifolia* as “hirtiflora” … The typographical error is correctable (vide Shenzhen Code Arts. 60.1, 61.4).”

**1013. **Eulalia leschenaultiana** (Decne.) Ohwi, Bull. Tokyo Sci. Mus. 18: 2. 1947 **

*Andropogon leschenaultianus* Decne., Herb. Timor. Descr. 29. 1835. Type: Timor

*Pollinia cumingii* Nees, Hooker’s J. Bot. Kew Gard. Misc. 2: 98. 1850. Type: Philippine Islands: Mindoro; *H. Cuming 1538*

*Eulalia cumingii* (Nees) A. Camus, Fl. Indo-Chine [P.H. Lecomte et al.] 7: 250. 1922

*Andropogon aureofulvus* Steud., Syn. Pl. Glumac. 1: 373. 1854 (as “aureo-fulvus”). Type: China:* Fortune Herb. 4*. Philippine Islands; *Cuming Herb. no. 1538*

**Distribution.** Chhattisgarh, Uttar Pradesh, Uttarakhand [China].

**Habit.** Perennial.

**1014. **Eulalia madkotiensis** Kandwal, B.K. Gupta & S.K. Srivast., Kew Bull. 62(3): 519–521. f. 1. 2007 **

**Type.** India: Uttaranchal, Pithoragarh District. Madkot, towards Jauljibi; August 2, 2004; *Manish K. Kandwal 3363* (BSD).

**Distribution.** Uttarakhand.

**Habit.** Perennial.

**1015. **Eulalia manipurensis** Bor, Grasses Burma, Ceylon, India & Pakistan 156. 1960 **

**Type.** Burma: Manipur State, Palel, in wet grassland, alt. 2300 ft.; *N.L. Bor 17736* (K).

**Distribution.** Manipur [Bangladesh, China, Myanmar].

**Habit.** Perennial.

**1016. **Eulalia mollis** (Griseb.) Kuntze, Revis. Gen. Pl. 2: 775. 1891 **

*Erianthus mollis* Griseb., Nachr. Königl. Ges. Wiss. Georg-Augusts-Univ. 92. 1868. Type: Himalaya occidentalis, 5000–6000 ft.; *Thomson Erianthus no. 3*. India: Kumaon, Almora; *R. Strachey s.n.*

*Pollinia mollis* (Griseb.) Hack., Monogr. Phan. [A. DC. & C. DC.] 6: 161. 1889

**Distribution.** Himachal Pradesh, Sikkim, Uttar Pradesh, Uttarakhand [Bhutan, China, Nepal].

**Habit.** Perennial.

**1017. **Eulalia pallens** (Hack.) Kuntze, Revis. Gen. Pl. 2: 775. 1891 **

*Pollinia pallens* Hack., Monogr. Phan. [A. DC. & C. DC.] 6: 156. 1889. Type: India; *J.D. Hooker Erianthus no. 17 *(B).

**Distribution.** Assam, Meghalaya [China].

**Habit.** Perennial.

**1018. **Eulalia phaeothrix** (Hack.) Kuntze, Revis. Gen. Pl. 2: 775. 1891 **

*Pollinia phaeothrix* Hack., Monogr. Phan. [A. DC. & C. DC.] 6: 168. 1889. Type: Sri Lanka: Newara Ellia; *Thwaites 959*. India; *Wight s.n.* Nilgiri Mountains; *Perrotet 1317, 1333*. Kunur; *Gamble s.n.*

**Distribution.** Andhra Pradesh, Karnataka, Kerala, Meghalaya, Tamil Nadu [China, Sri Lanka].

**Habit.** Perennial.

**1019. **Eulalia quadrinervis** (Hack.) Kuntze, Revis. Gen. Pl. 2: 775. 1891 **

*Pollinia quadrinervis* Hack., Monogr. Phan. [A. DC. & C. DC.] 6: 158–159. 1889. Type: China: Canton, *Sampson s.n.* Macao; *Vachell s.n.* Cap Syng Moon; *Meyen s.n.* Nepal; Numer. List [Wallich] no. 8808. India: Sikkim, Rinchingpong; *Anderson 1365*.

*Pseudopogonatherum quadrinerve* (Hack.) Ohwi, Bull. Tokyo Sci. Mus. 18: 3. 1947

*Pollinia villosa* Munro ex Benth., Fl. Hongk. 420. 1861, nom. later hom. & illeg., non Spreng., 1824

**Distribution.** Andhra Pradesh, Assam, Madhya Pradesh, Manipur, Meghalaya, Nagpur, Sikkim, Tamil Nadu, Uttarakhand [China, Myanmar, Nepal].

**Habit.** Perennial.

**1020. **Eulalia shrirangii** Salunkhe & Potdar, Kew Bull. 59(4): 625–627. f. 1. 2005 **

**Type.** India: Maharashtra, Satara District, Kas Plateau, alt. 4200 ft.; *Salunkhe 8170* (CAL).

**Distribution.** Maharashtra.

**Habit.** Annual.

**1021. **Eulalia speciosa** (Debeaux) Kuntze, Revis. Gen. Pl. 2: 775. 1891 var. **speciosa

*Erianthus speciosus* Debeaux, Actes Soc. Linn. Bordeaux 32: 53. 1878. Type: “Les hautes montagnes du Tché-foû, de 1000–1100 metres d’alt. dans les fissures des rochers, 1860”; *Debeaux s.n.*

*Pollinia speciosa* (Debeaux) Hack., Monogr. Phan. [A. DC. & C. DC.] 6: 159. 1889

**Distribution.** Meghalaya [China, Myanmar].

**Habit.** Perennial.

**1022. **Eulalia speciosa** (Debeaux) Kuntze var. **velutina** (Hack.) Bor, Grasses Burma, Ceylon, India & Pakistan 157. 1960 **

*Pollinia velutina* Hack., Monogr. Phan. [A. DC. & C. DC.] 6: 169. 1889. Type: India: Khasia, alt. 5000–6000 ft.; *J.D. Hooker & Thomson s.n.*

*Eulalia velutina* (Hack.) Kuntze, Revis. Gen. Pl. 2: 775. 1891

**Distribution.** Meghalaya.

**Habit.** Perennial.

**1023. **Eulalia staintonii** Bor, Kew Bull. 1957: 411–412. 1958 **

**Type.** Nepal: Kali Gandaki, alt. 9000 ft.; September 18, 1954; *Stainton, Sykes & Williams 7919* (BM).

**Distribution.** Maharashtra, Uttar Pradesh [Nepal].

**Habit.** Perennial.

**Remarks.** Records for India fide Naithani (1990).

**1024. **Eulalia thwaitesii** (Hack.) Kuntze, Revis. Gen. Pl. 2: 775. 1891 **

*Pollinia thwaitesii* Hack., Monogr. Phan. [A. DC. & C. DC.] 6: 163. 1889. Type: Sri Lanka: Newara Ellia; *Thwaites 949*.

**Distribution.** Kerala [Sri Lanka].

**Habit.** Perennial.

**Remarks.** Record for Kerala fide Naithani (1990).

**1025. **Eulalia trispicata** (Schult.) Henrard, Blumea 3(3): 453. 1940 **

*Andropogon trispicatus* Schult., Mant. 2 (Schultes) 452. 1824, replaced synonym *Andropogontristachyos* Roxb., 1820

*Andropogon tristachyos* Roxb., Fl. Ind. (Carey and Wallich ed.) 1: 261. 1820, nom. illeg. hom., non Kunth, 1816. Type: India: “found on newly laid down pasture ground in the vicinity of Calcutta.”

*Erianthus hexastachyus* Hochst., Verh. Nederl. Inst. III 4: 35. 1851. Type: India: “Prope Castellum Mulki in terra Canara detexit Metz.”

*Andropogon hexastachyus* (Hochst.) Steud., Syn. Pl. Glumac. 1: 380. 1854

*Eulalia argentea* Brongn., Voy. Monde, Phan. 2(2): 92. 1827. Type: “Amboine et Bourou, dans les Moluques.”

*Pollinia argentea* (Brongn.) Trin., Mém. Acad. Imp. Sci. Saint-Pétersbourg, Sér. 6, Sci. Math., Seconde Pt. Sci. Nat. 5, 2(1): 90. 1836. Type: “Amboina Molucc.”

*Eulalia tristachya* Kuntze, Revis. Gen. Pl. 2: 775. 1891

**Distribution.** Andhra Pradesh, Assam, Bihar, Chhattisgarh, Goa, Gujarat, Himachal Pradesh, Jammu & Kashmir, Karnataka, Kerala, Maharashtra, Meghalaya, Odisha, Rajasthan, Tamil Nadu, Uttarakhand, West Bengal [Bangladesh, Bhutan, China, Myanmar, Nepal, Sri Lanka].

**Habit.** Perennial.

**1026. **Eulalia villosa** (Spreng.) Nees, Fl. Afr. Austral. III. 91. 1841 **

*Pollinia villosa* Spreng., Syst. Veg. [Sprengel] 1: 288. 1824 (“1825”). Replaced name.

*Andropogon villosus* Thunb., Prodr. Pl. Cap. 20. 1794, nom. illeg. hom., non Lam. (1779). Type: South Africa: Hab. Bonae Spei Africaes, *Thunberg s.n.*

*Eulalia wightii* (Hook. f.) Bor, Grasses Burma, Ceylon, India and Pakistan 158. 1960

*Pollinia quadrinervis* Hack. var. *wightii* Hook. f., Fl. Brit. India (J.D. Hooker). 7(21): 110. 1896. Type: India: Pulney Mountains; *Wight 3390 *(K).

**Distribution.** Andhra Pradesh, Daman and Diu, Gujarat, Karnataka, Kerala, Maharashtra, Odisha, Tamil Nadu, Uttar Pradesh [Sri Lanka].

**Habit.** Perennial.

***Eulaliopsis* Honda, Bot. Mag. (Tokyo) 37: 124. 1923**

**Type. ***Eulaliopsis angustifolia* Honda (*Spodiopogon angustifolius* Trin.).

**1027. **Eulaliopsis binata** (Retz.) C.E. Hubb., Hooker’s Icon. Pl. 33: sub t. 3262. 1935 **

*Andropogon binatus* Retz., Observ. Bot. (Retzius) 5: 21. 1788 (“1789”) (as “binatum”). Type: “Tschandranconae lectum dedit amiciff. Koening.” India: Travancore; *Koenig s.n. *(LD).

*Pollinidium binatum* (Retz.) C.E. Hubb., Bull. Misc. Inform. Kew 1932: 72. 1932

*Andropogon involutus* Steud., Syn. Pl. Glumac. 1: 373. 1854. Type: India; *Wallich* (Numer. List [Wallich] no. 8845)

*Andropogon notopogon* Steud., Syn. Pl. Glumac. 1: 373. 1854. Type: India; *Royle s.n.*

*Andropogon obvallatus* Steud., Syn. Pl. Glumac. 1: 373. 1854. Type: Philipines: Luzon, Pangasinan; *H. Cuming 1002*

*Spodiopogon angustifolius* Trin., Mém. Acad. Imp. Sci. St.-Pétersbourg, Sér. 6, Sci. Math. 2: 300. 1832. Type: Nepal

*Ischaemum angustifolium* (Trin.) Hack., Hooker’s Icon. Pl. 18: t. 1773. 1888

*Pollinidium angustifolium* (Trin.) Haines, Bot. Bihar Orissa 5: 1020. 1924

*Eulaliopsis angustifolia* (Trin.) Honda, Bot. Mag. (Tokyo) 38: 56. 1924

*Eulaliopsis duthiei* Sur, J. Bombay Nat. Hist. Soc. 72: 815. 1976. Type: India: Uttar Pradesh, Ganga Valley, (Tehri-Garhwal); June 11, 1883; *J.F. Duthie 14* (CAL).

**Distribution.** Andhra Pradesh, Bihar, Chhattisgarh, Gujarat, Jammu and Kashmir, Madhya Pradesh, Maharashtra, Odisha, Punjab, Rajasthan, Uttar Pradesh, Uttarakhand [Afghanistan, China, Myanmar, Nepal, Pakistan].

**Habit.** Perennial.

***Garnotia* Brongn., Voy. Monde, Phan. 2 (pt. 10): 133. t. 21. 1832**

**Type. ***Garnotia stricta* Brongn.

**1028. **Garnotia acutigluma** (Steud.) Ohwi, Bot. Mag. (Tokyo) 55: 393. 1941 var. **acutigluma

*Urachne acutigluma* Steud., Syn. Pl. Glumac. 1: 121. 1854. Type: Java; 1851; *Goering sect. II. nr. 141* (P-STEUD). (as “Japan” is given in Steudel)

**Distribution.** Arunachal Pradesh, Assam, Meghalaya, Nagaland, Sikkim, West Bengal [Bangladesh, Bhutan, China, Myanmar].

**Habit.** Perennial.

**Remarks.** All records for India fide Naithani (1990).

**1029. **Garnotia acutigluma** (Steud.) Ohwi var. **longiseta** (Hack.) V. Prakash & S.K. Jain, Fasc. Fl. India 3: 6. 1979 **

*Garnotia stricta* Brongn. var. *longiseta* Hack., Allg. Bot. Z. Syst. 15: 141. 1909. Type: Philippines: Luzon, Bataan, Mariveles, alt. 4000 ft.; December 12, 1908; *E.D. Merrill s.n.* (W).

**Distribution.** Arunachal Pradesh, Meghalaya [also in Indonesia].

**Habit.** Perennial.

**Remarks.** Records for India fide Naithani (1990).

**1030. **Garnotia arborum** Stapf ex T. Cooke, Fl. Bombay 2(5): 1013. 1908 **

**Type. **India: Deccan, Western Ghats, Nandgaon; October 1, 1898; *Woodrow 30 *(K).

**Distribution.** Karnataka, Maharashtra.

**Habit.** Annual.

**1031. **Garnotia arundinacea** Hook. f., Fl. Brit. India (J.D. Hooker). 7(22): 243. 1896 **

**Type. **India: Tamil Nadu, Nilghiri Hills, alt. 2000–6000 ft.; *Heyne s.n.*

**Distribution.** Gujarat, Karnataka, Kerala, Madhya Pradesh, Tamil Nadu.

**Habit.** Annual.

**1032. **Garnotia courtallensis** (Arn. & Nees) Thwaites, Enum. Pl. Zeyl. (Thwaites). 363. 1864 **

*Miquelia courtallensis* Arn. & Nees, Nov. Actorum Acad. Caes. Leop.-Carol. Nat. Cur., Suppl. 19(1): 179. 1843. Type: India: Courtallum; *Wight cat. no. 2346*

*Berghausia courtallensis* (Arn. & Nees) Endl. ex Miq., Nieuwe Verh. Eerste Kl. Kon. Ned. Inst. Wetensch. Amsterdam ser. 3, 4: 32. 1851

*Garnotia longipila* Santos, Nat. Appl. Sci. Bull. Univ. Philipp. 10: 119–120. 1950. Type: India: Madhras, Kodaikanal, Pulney (Palni); December 7, 1898; *A.G. Bourne et uxoris num 1956* (US Nat. Herb.).

*Garnotia puchiparensis* Bor, Indian Forest Rec., Bot. n.s. 2(4): 234–236. 1941. Type: India: Madras, Silent Valley, Puchipara rest house, alt. 3000 ft.; *Bor 8443* (DD).

**Distribution.** Karnataka, Kerala, Tamil Nadu [Sri Lanka].

**Habit.** Annual.

**1033. **Garnotia elata** (Arn. ex Miq.) Janowski, Repert. Spec. Nov. Regni Veg. 17: 86. 1921 **

*Berghausia elata* Arn. ex Miq., Nieuwe Verh. Eerste Kl. Kon. Ned. Inst. Wetensch. Amsterdam ser. 3, 4: 32. 1851. Type: India; *Wight cat. no. 2600*

*Garnotia schmidii* Hook. f., Fl. Brit. India (J.D. Hooker). 7(22): 242. 1896. Type: India: Nilghiri Hills; *Schmid s.n.*(K). Gondaloor Ghat, alt. 4500 ft.; *Lawson s.n.*

*Garnotia tenuiglumis* Stapf ex Hook. f., Fl. Brit. India (J.D. Hooker). 7(22): 242. 1896. Type: Western Peninsula; *Wight Herb. no. 3247*

*Garnotia scoparia* Stapf ex Hook. f., Fl. Brit. India (J.D. Hooker). 7(22): 242. 1896, non Thwaites, 1864

**Distribution.** Andhra Pradesh, Karnataka, Kerala, Tamil Nadu, Uttar Pradesh.

**Habit.** Perennial.

**Remarks.***Garnotia schmidii* is treated as a separate species by T.S. Nayar et al.; but Gould (1972) places it here.

**1034. **Garnotia exaristata** Gould, Kew Bull. 27(3): 558. 1972 **

*Garnotia tectorum* Hook. f., Fl. Brit. India (J.D. Hooker). 7(22): 242. 1896, quoad descr. nom. illegit. Type: Sri Lanka: elevated parts of the Island; *Gardner s.n.*

*Garnotia mutica* sensu Bor, Grasses Burma, Ceylon, India and Pakistan 568. 1960, non (Munro) Druce, 1916

**Type.** Sri Lanka: Horton Plains; May 10, 1970; *Gould & Cooray 13777* (K).

**Distribution.** Kerala, Karnataka, Tamil Nadu [Myanmar, Sri Lanka].

**Habit.** Perennial.

**Remarks.** Records for Kerala and Tamil Nadu fide Naithani (1990).

**1035. **Garnotia fergusonii** Trimen, J. Bot. 27: 170. 1889 **

**Type.** Sri Lanka: Central Province, summit of Knuckles Mountains, alt. 6000 ft.; March, 1887; *Ferguson s.n.* (PDA).

**Distribution.** Karnataka, Tamil Nadu [Sri Lanka].

**Habit.** Perennial.

**Remarks.** Records for Karnataka and Tamil Nadu fide Naithani (1990).

**1036. **Garnotia micrantha** Thwaites, Enum. Pl. Zeyl. (Thwaites) 363. 1864 **

**Type. **“Hab. Central Province, alt. 2000–4000 ft.” Sri Lanka; *Thwaites CP 945* (PDA).

**Distribution. **Kerala [Sri Lanka].

**Habit. **Annual.

**1037. **Garnotia emodi** (Arn. & Nees) Janowski, Repert. Spec. Nov. Regni Veg. 17: 86. 1921 **

*Miquelia emodi* Arn. & Nees, Nov. Actorum Acad. Caes. Leop.-Carol. Nat. Cur. Suppl. 19(1): 178–179. 1843. Type: “In emodi iugo, Royle”; *Royle 33*

*Berghausia emodi* (Arn. & Nees) Endl. ex Miq., Nieuwe Verh. Eerste Kl. Kon. Ned. Inst. Wetensch. Amsterdam ser. 3, 4: 32. 1851. Type: “In Emodi jugo (*Royle Herb. no. 33*).”

*Garnotia polypogonoides* Munro ex Oliver, Hooker’s Icon. Pl. 15: 64. t. 1481. 1885. Type. East India, Nepal; Numer. List [Wallich] no. 8884. “Sikkim and the north west Himalaya, also in Wight’s Herbarium unlocalised.”

**Distribution.** Meghalaya, Sikkim [Nepal].

**Habit.** Perennial.

**1038. **Garnotia scoparia** Thwaites, Enum. Pl. Zeyl. (Thwaites). 363. 1864 **

*Garnotia thwaitesii* Stapf ex Hook. f., Fl. Brit. India (J.D. Hooker). 7(22): 241. 1896, nom. superfl. & illeg.

**Type.** Sri Lanka: Hotter parts of the Island; *Munro Ceylon Plant 943* (PDA).

**Distribution.** Karnataka, Kerala, Tamil Nadu [Sri Lanka].

**Habit. **Perennial.

**Remarks.** Record for Karnataka fide Naithani (1990).

**1039. **Garnotia stricta** Brongn., Voy. Monde, Phan. 2(pt. 10): 133, t. 21. 1832 **

*Berghausia pallens* Arn. ex Miq., Nieuwe Verh. Eerste Kl. Kon. Ned. Inst. Wetensch. Amsterdam ser. 3, 4: 33. 1851. Type: India: Kerala, Quilon; *Wight cat. no. 2598*

**Type.** “Ile de taiti”

**Distribution.** Andhra Pradesh, Assam, Bihar, Karnataka, Maharashtra, Meghalaya, Sikkim, Uttar Pradesh, Uttarakhand [China, Myanmar].

**Habit.** Perennial.

**1040. **Garnotia tenella** (Arn. ex Miq.) Janowski, Repert. Spec. Nov. Regni Veg. 17: 86. 1921 **

*Berghausia tenella* Arn. ex Miq., Nieuwe Verh. Eerste Kl. Kon. Ned. Inst. Wetensch. Amsterdam ser. 3, 4: 34. 1851. Type: India; *Wight cat. no. 2599*

*Garnotia stricta* sensu Hook. f., Fl. Brit. India (J.D. Hooker). 7(22): 243. 1896, non Brongn., 1832

**Distribution.** Andhra Pradesh, Bihar, Goa, Gujarat, Karnataka, Kerala, Maharashtra, Meghalaya, Nagaland, Odisha, Sikkim, Tamil Nadu, Uttar Pradesh, West Bengal [Bhutan, China, Myanmar, Nepal].

**Habit.** Annual.

**Remarks.** Record for Nepal and all records for India except Goa fide Naithani (1990).

**1041. **Garnotia variyamensis** Sunil, Naveen Kum. & Sanilkum. in J. Bot. Res. Inst. Texas 8(2): 517. 2014. **

**Type.** India: Kerala, Ernakulam district, Edamalayar Forest Range, Variyam, 794 m, 16 October 2013, *Sunil & Naveen Kumar 6193* (holotype: CAL; isotype: MH).

**Distribution.** Kerala.

**Habit.** Annual.

***Germainia* Balansa & Poitr, Bull. Soc. Hist. Nat. Toulouse 7: 344. 1873**

**Type. ***Germainia capitata* Balansa & Poitr.

**1042. **Germainia khasyana** Hack., Oesterr. Bot. Z. 41: 50. 1891 **

**Type. **India: Khasya, Nartung, alt. 4000; *C.B. Clarke 44830*. Pooriung; *C.B. Clarke 42558.*

**Distribution.** Assam, Meghalaya.

**Habit.** Perennial.

***Glyphochloa* Clayton, Kew Bull. 35(4): 814. 1981**

**Type. ***Glyphochloa forficulata* (C.E.C. Fisch.) Clayton (*Manisuris forficulata* C.E.C. Fisch.)

**1043. **Glyphochloa acuminata** (Hack.) Clayton, Kew Bull. 35(4): 815. 1981 var. **acuminata

*Rottboellia acuminata* Hack., Monogr. Phan. [A. DC. & C. DC.] 6: 291. 1889. Type: Maisur & Carnatic; *J.D. Hooker & Thomson s.n.*

*Manisuris acuminata* (Hack.) Kuntze, Revis. Gen. Pl. 2: 779. 1891

*Peltophorus acuminatus* (Hack.) A. Camus, Bull. Mus. Natl. Hist. Nat. 27: 371. 1921

**Distribution.** Karnataka, Kerala, Maharashtra.

**Habit.** Annual.

**1044. **Glyphochloa acuminata** (Hack.) Clayton var. **stocksii** (Hook. f.) Clayton, Kew Bull. 35(4): 815. 1981 **

*Rottboellia acuminata* Hack. var. *stocksii *Hook. f., Fl. Brit. India (J.D. Hooker). 7(21): 155. 1896. Type: India: Malwar; *J.E. Stocks s.n.* (K).

*Rottboellia acuminata* Hack. var. *stocksii* (Hook. f.) Clayton, Kew Bull. 35(4): 815. 1981

*Manisuris acuminata* (Hack.) Kuntze var. *stocksii* (Hook. f.) Jain, Bull. Bot. Surv. India 12(1–4): 9. 1972 (“1970”)

**Distribution.** Karnataka, Maharashtra, Tamil Nadu.

**Habit.** Annual.

**1045. **Glyphochloa acuminata** (Hack.) Clayton var. **woodrowii** (Bor) Clayton, Kew Bull. 35(4): 815. 1981 **

*Manisuris acuminata* (Hack.) Kuntze var. *woodrowii* Bor, Grasses Burma, Ceylon, India & Pakistan 191. 1960. Type: India: Bombay; *R.K. Bhide s.n.*(K).

**Distribution.** Karnataka, Maharashtra.

**Habit.** Annual.

**1046. **Glyphochloa divergens** (Hack.) Clayton, Kew Bull. 35(4): 815. 1981 var. **divergens

*Rottboellia divergens* Hack., Monogr. Phan. [A. DC. & C. DC.] 6: 292. 1889. Type: India: *Hügel in h. Vindob. 3147*

*Manisuris divergens* (Hack.) Kuntze, Revis. Gen. Pl. 2: 779. 1891

**Distribution.** Karnataka, Kerala, Maharashtra.

**Habit.** Annual.

**1047. **Glyphochloa divergens** (Hack.) Clayton var. **hirsuta** (C.E.C. Fisch.) Clayton, Kew Bull. 35(4): 815. 1981 **

*Manisuris forficulata* C.E.C. Fisch. var. *hirsuta* C.E.C. Fisch., Bull. Misc. Inform. Kew 1933: 355. 1933. Type: India: Bababudan Hills, Kalhatti, alt. 6000 ft.; November; *A. Meebold 10559* (K-Isotype).

*Manisuris divergens* (Hack.) Kuntze var. *hirsuta* (C.E.C. Fisch.) Jain, Bull. Bot. Surv. India 12(1–4): 12. 1972 (“1970”)

**Distribution.** Karnataka, Maharashtra.

**Habit.** Annual.

**1048. **Glyphochloa forficulata** (C.E.C. Fisch.) Clayton, Kew Bull. 35(4): 815. 1981 **

*Manisuris forficulata* C.E.C. Fisch., Bull. Misc. Inform. Kew 1933: 355. 1933 var. *forficulata*. Type: India: Bombay Presidency, Mahableshwar; *J.C. Lisboa s.n.* (K).

*Rottboellia divergens* sensu Hook. f., Fl. Brit. India (J.D. Hooker). 7(21): 155. 1896, non Hack., 1889

**Distribution.** Karnataka, Kerala, Madhya Pradesh, Maharashtra, Tamil Nadu.

**Habit.** Annual.

**Remarks.** Records for Madhya Pradesh, Maharashtra, and Tamil Nadu fide Naithani (1990).

**1049. **Glyphochloa goaensis** (R. S. Rao & Hemadri) Clayton, Kew Bull. 35(4): 815. 1981 **

*Manisuris goaensis* R. S. Rao & Hemadri, Bull. Bot. Surv. India 10(1): 106. 1968. Type: India: Goa, rocky plateau near Verna village on Cortalim-Madgoa road; November 8, 1962; *R.S. Rao 84474A* (CAL.)

**Distribution.** Goa, Maharashtra.

**Habit.** Annual.

**1050. **Glyphochloa maharashtraensis** Potdar & S.R. Yadav, Kew Bull. 66(4): 625. 2012. var. **maharashtraensis

**Type.** India: Maharashtra, Kolhapur district, Udgiri, 906 m, 10 October 2007, *Potdar 1880* (holotype: CAL; isotypes: BLAT, BSI, K, SUK).

**Distribution.** Maharashtra.

**Habit. **Annual

**1051. **Glyphochloa maharashtraensis** Potdar & S.R. Yadav var. **hirsuta** Potdar & S.R. Yadav, Kew Bull. 66(4): 628. 2012. **

**Type.** India; Maharashtra, Kolhapur district, Udgiri, 906 m, 10 October 2007, *Potdar 1927* (holotype: CAL; isotypes: BLAT, K, SUK).

**Distribution.** Maharashtra.

**Habit.** Annual.

**1052. **Glyphochloa mysorensis **(Jain & Hemadri) Clayton, Kew Bull. 35(4): 815. 1981 **

*Manisuris mysorensis* Jain & Hemadri, Bull. Bot. Surv. Inida 10: 280. 1969. Type: India: Mysore State, Castle Rock; October 25, 1902; *G. A. Gammie 15643* (CAL).

**Distribution.** Karnataka, Maharashtra.

**Habit.** Annual.

**1053. **Glyphochloa ratnagirica** (B. G. Kulk. & Hemadri) Clayton, Kew Bull. 35: 815. 1981 **

*Manisuris ratnagirica* B. G. Kulk. & Hemadri, Indian Forester 100(4): 250. 1974. Type: India: Maharashtra, Ratnagiri District, Ambolighat, alt. 2000 ft.; October 12, 1970; *B.G. Kulkarni 121638A* (K).

**Distribution.** Karnataka, Maharashtra.

**Habit.** Annual.

**1054. **Glyphochloa santapaui** (Jain & Deshp.) Clayton, Kew Bull. 35(4): 815. 1981 **

*Manisuris santapaui* Jain & Deshp., Bull. Bot. Surv. India 10(3–4): 277. 1969 (“1968”). Type: India: Maharashtra State, Ratnagiri; September 15, 1961; *C. Saldanha 7130A *(CAL).

**Distribution.** Maharashtra.

**Habit.** Annual.

**1055. **Glyphochloa talbotii** (Hook. f.) Clayton, Kew Bull. 35(4): 816. 1981 **

*Rottboellia talbotii* Hook. f., Fl. Brit. India (J.D. Hooker). 7(21): 155. 1896 (as “Talboti”). Type: India: Goa District; *Talbot 2572 *(K).

*Manisuris talbotii* (Hook. f.) Bor, Grasses Burma, Ceylon, India and Pakistan 192. 1960

*Peltophorus talbotii* (Hook. f.) A. Camus, Bull. Mus. Natl. Hist. Nat. 27: 371. 1921 (as “talboti”)

**Distribution.** Goa, Maharashtra.

**Habit.** Annual.

**1056. **Glyphochloa veldkampii** M.A.Fonseca & Janarth., Rheedea 13(1–2): 35 -38. f. 1. 2004 **

**Type.** India: Goa, Kasauli, along the Panaji-Belgaum highway in the outskirts of Bhagwan Mahavir Wildlife Sanctuary, alt. 272 ft., 15° 21'N, 74°11'E; October 21, 2001; *Janarthanam & Fonseca 1901* (CAL).

**Distribution.** Goa.

**Habit.** Annual.

***Hemarthria* R. Br., Prodr. Fl. Nov. Holland. 207. 1810**

**Lectotype. ***Hemarthria compressa* (L. f.) R. Br. (*Rottboellia compressa* L. f.). LT designated by Nash, N. Amer. Fl. 17: 87. 30 1909; affirmed by Hitchcock, Bull. U.S.D.A. 772: 278. 1920.

**1057. **Hemarthria altissima** (Poir.) Stapf & C.E. Hubb., Bull. Misc. Inform. Kew 1934: 109. 1934 **

*Rottboellia altissima* Poir., Voy. Barbarie 2: 105. 1789 (as “Rottboella”). Type: Algeria: Bastion; *Poiret s.n. *(P-LA).

*Manisuris altissima* (Poir.) Hitchc., J. Wash. Acad. Sci. 24(7): 292. 1934

*Rottboellia compressa* L.f. var. *fasciculata* Hack., Monogr. Phan. [A. DC. & C. DC.] 6: 286. 1889

*Rottboellia fasciculata* Lam., Tabl. Encycl. 1: 204. 1792, nom. superfl. & illeg. for *R. altissima*

*Hemarthria fasciculata* Kunth, Révis. Gramin. 1: 153. 1829, nom. superfl.

**Distribution.** Tamil Nadu [China, Myanmar].

**Habit.** Perennial.

**1058. **Hemarthria compressa** (L.f.) R. Br., Prodr. Fl. Nov. Holland. 1: 207. 1810 **

*Rottboellia compressa* L.f., Suppl. Pl. 114. 1782 (“1781”). Type: “Habitat in indiis” Neotype: Numer. List [Wallich] no. 8871-E (L). Neotype designated by E. van den Heuvel & J.F. Veldkamp in Blumea 45(2): 455. 2000.

*Hemarthria coromandelina* Steud., Syn. Pl. Glumac. 1: 358. 1854. Type: *Roxburgh., Pl. Coromandel 2: pl. 181 (1805)*. Epitype: *Roxburgh s.n.* (BM). Epitype designated by E. van den Heuvel & J.F. Veldkamp in Blumea 45(2): 469. 2000.

*Hemarthria laxa* Steud., Syn. Pl. Glumac. 1: 358. 1854. Type: India; Numer. List [Wallich] no. 8871 (P).

*Rottboellia glabra* Roxb., Fl. Ind. (Carey and Wallich ed.) 1: 353. 1820. Type: India: Bengal, Pasture land; *Roxburgh s.n.* (BM).

**Distribution.** Andhra Pradesh, Chhattisgarh, Dehli, Gujarat, Jammu and Kashmir, Karnataka, Madhya Pradesh, Maharashtra, Odisha, Rajasthan, Tamil Nadu, Uttar Pradesh, Uttarakhand [Afghanistan, Bangladesh, Bhutan, China, Myanmar, Nepal, Pakistan, Sri Lanka].

**Habit.** Perennial.

**1059. **Hemarthria hamiltoniana** Steud., Syn. Pl. Glumac. 1: 358. 1854 **

*Rottboellia compressa* L.f. var. *hamiltoniana* (Steud.) Hack., Monogr. Phan. [A. DC. & C. DC.] 6: 288. 1889

*Rottboellia protensa* (Steud.) Hack. var. *hamiltoniana* (Steud.) Hook. f., Fl. Brit. India (J.D. Hooker). 7(21): 154. 1896

**Type.** India: upper Gangetic Plains, Kathpur; *Buchanan-Hamilton *(Numer. List [Wallich] no. 8870-C) (K).

**Distribution.** Uttar Pradesh.

**Habit.** Perennial.

**1060. **Hemarthria longiflora** (Hook. f.) A. Camus, Fl. Indo-Chine [P.H. Lecomte et al.] 7: 379–380. 1922 **

*Rottboellia longiflora* Hook. f., Fl. Brit. India (J.D. Hooker). 7(21): 154. 1896. Lectotype: India: Tenasserim, Mergui; *Griffith KD 1009 *(K). LT designated by E. van den Heuvel & J.F. Veldkamp in Blumea 45(2): 459. 2000.

*Rottboellia tonkinensis* A. Camus, Bull. Mus. Natl. Hist. Nat. 25: 369. 1919. Lectotype: Vietnam: Sontay; *Balansa 1783* (P). LT designated by E. van den Heuvel & J.F. Veldkamp in Blumea 45(2): 459. 2000.

**Distribution.** Assam [Bangladesh, China, Myanmar].

**Habit.** Perennial.

**1061. **Hemarthria vaginata** Buse, Pl. Jungh. 354. 1854 **

*Hemarthria protensa* Steud., Syn. Pl. Glumac. 1: 359. 1854. Type: India Orientalis; *Wallich *(Numer. List [Wallich] no. 8872).

*Manisuris protensa* (Steud.) Hitchc., Brittonia 2(2): 127. 1936

*Rottboellia protensa* (Steud.) Hack., Monogr. Phan. [A. DC. & C. DC.] 6: 289. 1889

**Type.** Indonesia: Java, Tijibogo; *F.W. Junghuhn s.n.* (L-903.342-454).

**Distribution.** Arunachal Pradesh, Assam, West Bengal, Bihar, Meghalaya, Nagaland [Bangladesh, Bhutan, China, Myanmar, Nepal].

**Habit.** Perennial.

***Hemisorghum* C.E. Hubb., Grasses Burma, Ceylon, India & Pakistan 686. 1960**

**Type. ***Hemisorghum mekongense* (A. Camus) C.E. Hubb. (*Sorghum mekongense* A. Camus).

**1062. **Hemisorghum venustum** (Thwaites) Clayton, Kew Bull. 27(3): 448. 1972 **

*Andropogon venustus* Thwaites, Enum. Pl. Zeyl. (Thwaites). 367. 1864.Type: Sri Lanka: Central Province, Rambodde, alt. upto 4000 ft.; *Thwaites Ceylon Plant 2875* (K)

*Bothriochloa venusta* (Thwaites) A. Camus, Ann. Soc. Linn. Lyon ser. 2, 76: 165. 1931

*Capillipedium venustum* (Thwaites) Bor, Grasses Burma, Ceylon, India and Pakistan 113. 1960

*Vetiveria venusta* (Thwaites) Willis, Revis. Cat. Fl. Pl. Ceylon 110. 1911

**Distribution.** Tamil Nadu [Sri Lanka].

**Habit.** Perennial.

**Remarks.** Record for Tamil Nadu fide Naithani (1990).

***Heteropogon* Pers., Syn. Pl. (Persoon) 2: 533. 1807**

**Lectotype. ***Heteropogon glaber* Pers., nom. illeg. (*Andropogon allioni* DC., *Heteropogon allionii* (DC.) Roem. & Schult.). LT designated by Nash in N. Amer. Fl. 17: 127. 1912; affirmed by Hitchcock, Bull. U.S.D.A. 772: 273. 1920.

**1063. **Heteropogon contortus** (L.) P. Beauv., Syst. Veg., ed. 15 bis [Roemer & Schultes] 2: 836. 1817 **

*Andropogon contortus* L., Sp. Pl. 2: 1045. 1753 (as “contortum”). Type: “Habitat in India.” Lectotype. “Gram. Secalinum, Indicum, spica gracili, tomentoso, aristis longioribus, ad se invicem intortis” in Plukenet, Phytographia, t. 191. f. 5. 1692. Voucher: *Herb. Sloane 92: 78; 96: 69 *(BM-SL). LT designated by Cope in Nasir & Ali (ed.), Fl. Pakistan 143: 312. 1982.

*Andropogon allionii* Lam. ex DC., Fl. Franc. (DC. & Lamarck), ed. 3. 3: 97. 1805. Type: “*Andropogon contortum* All. Ped. n. 2277. t. 91. f. 4. excl. syn.” “Cette espèce croit sur les collines et les rochers, au-dessus du lac d’Ivrée et de la vallée de Suze (All.).”

*Heteropogon allionii* (Lam. ex DC.) Roem. & Schult., Syst. Veg., ed. 15 bis [Roemer & Schultes] 2: 835. 1817

*Andropogon besukiensis* Steud., Syn. Pl. Glumac. 1: 367. 1854. Type: Indonesia: “Ad rupes montis Arak-Ak Prov. Besukie, Java”;* Zollinger Herb. no. 2770*

*Heteropogon firmus* J. Presl, Reliq. Haenk. 1(4–5): 334. 1830. Type: “Habitat in Mexico.”

*Heteropogon hispidissimus* Hochst. ex Steud., Syn. Pl. Glumac. 1: 367. 1854. Type: Ethiopia: Tigray, Gafta; 18 September, 1838; *G.H.W. Schimper 1219* (K).

*Andropogonpolystachyos *Roxb.**, **Fl. Ind. (Carey and Wallich ed.) 1: 265. 1820

*Heteropogon polystachyos *(Roxb.) Schult., Mant. 2 (Schultes) 460. 1824 (as “polystachyus”)

*Heteropogon roxburghii* Arn. ex Nees, Gramineae: 51. 1841.

*Heteropogon glaber* Pers., Syn. Pl. (Persoon) 2: 533. 1807, nom. superfl. & illeg. for *Andropogonallionii*

*Heteropogon hirsutus* P. Beauv., Ess. Agrostogr. 134. 1812, no reference to basionym and nom. nud.

*Andropogon bellardii* Bubani, Nuovo Giorn. Bot. Ital. 5: 317. 1873 (as “bellardi”), nom. superfl. & illeg.

*Heteropogon hirtus* Pers., Syn. Pl. (Persoon) 2: 533. 1807, nom. superfl. & illeg. for *Andropogoncontortus*

*Heteropogon hohenackeri* Hochst. ex Miq., Nieuwe Verh. Eerste Kl. Kon. Ned. Inst. Wetensch. Amsterdam ser. 3, 4: 36. 1851, nom. nud. Type: India: Manglore; *Metz Herb. no. 301*

**Distribution.** Andhra Pradesh, Bihar, Chhattisgarh, Delhi, Goa, Haryana, Himachal Pradesh, Jharkhand, Karnataka, Kerala, Madhya Pradesh, Maharashtra, Nagaland, Odisha, Rajasthan, Tamil Nadu, Uttar Pradesh, Uttarakhand, West Bengal [China, Myanmar, Pakistan].

**Habit.** Perennial.

**1064. **Heteropogon fischerianus** Bor, Kew Bull. 1951: 170–171. 1951 **

*Heteropogon contortus* (L.) P. Beauv. var. *distichus* C.E.C. Fisch., Fl. Madras 3(10): 1743. 1934. Type: India: Anamallais, alt. 3500 ft.; *Barber s.n.* Kodaikanal, alt. 6000–7000 ft.; *Bourne s.n.*

**Type.** India: Madras, Kodaikanal, Pulneys, Jesmond Hill; July 1, 1901; *Ibid 2025* (K).

**Distribution.** Kerala, Tamil Nadu.

**Habit.** Perennial.

**1065. **Heteropogon melanocarpus** (Elliot) Benth., J. Linn. Soc., Bot. 19: 71. 1881 **

*Andropogon melanocarpus* Elliot, Sketch Bot. S. Carolina 1: 146. 1816. Type: USA: Georgia, in the pine barrens between Fort Barrington on the Altamaha and Jefferson on the Satilla; *R. Habersham s.n.* (CHARL).

*Cymbopogon melanocarpus* (Elliot) Spreng., Syst. Veg. (ed. 16) [Sprengel] 1: 289. 1824 (“1825”)

*Heteropogon acuminatus* Trin., Mém. Acad. Imp. Sci. St.-Pétersbourg, Sér. 6, Sci. Math. 2: 254. 1832. Type: Brazil

*Andropogon polystictus* Hoschst. ex Steud., Syn. Pl. Glumac. 1: 369. 1854. Type: Abyssinia [Ethiopia]; *[Schimper] Hrbr. Abyss. 2012*

*Heteropogon polystictus* (Hochst. ex Steud.) Hochst., Flora 39: 28. 1856

*Heteropogon roylei* Nees ex Steud., Syn. Pl. Glumac. 1: 367. 1854, nom. inval., pro. syn. of Andropogon segaenensis

**Distribution.** Rajasthan, Uttar Pradesh, Uttarakhand [China].

**Habit.** Annual.

**1066. **Heteropogon ritchiei** (Hook. f.) Blatt. & McCann, J. Bombay Nat. Hist. Soc. 32: 623. 1928 **

*Andropogon ritchiei* Hook. f., Fl. Brit. India (J.D. Hooker). 7(21): 201. 1896. Type: India: Deccan, Belaum; *Ritchie s.n.*

**Distribution.** Gujarat, Karnataka, Maharashtra, Rajasthan.

**Habit.** Annual.

**1067. **Heteropogon triticeus** (R. Br.) Stapf ex Craib, Bull. Misc. Inform. Kew 1912: 432. 1912 **

*Andropogon triticeus* R. Br., Prodr. Fl. Nov. Holland. 1: 201. 1810. Type: Australia: “Littora Novae Hollandiae intra tropicum.”

*Andropogon ischyranthus* Steud., Syn. Pl. Glumac. 1: 367. 1854. Type: Philippines; *Cuming Herb. no. 1005*

*Andropogon lianatherus* Steud., Syn. Pl. Glumac. 1: 367. 1854. Type: Indonesia: Java

*Andropogon segaenensis* Steud., Syn. Pl. Glumac. 1: 367. 1854. Type: India: “Heteropogon Roylei Nees mpt*., *Numer. List [Wallich] no. 8801”

*Heteropogon insignis* Thwaites, Enum. Pl. Zeyl. (Thwaites). 437. 1864. Type: Sri Lanka: Mahning-galla, Matelle East; *Thwaites Ceylon Plant 3804*

**Distribution.** Gujarat, Karnataka, Maharashtra, Tamil Nadu [China, Myanmar, Sri Lanka].

**Habit.** Perennial.

***Hyparrhenia* Andersson ex E. Fournier, Mex. Pl. 2: 51, 67. 1886**

**Lectotype. ***Hyparrhenia foliosa* (Kunth) E. Fournier (*Anthistiria foliosa* Kunth). LT designated by Clayton in Kew Bull. Add. Ser. 2: 38. 1969.

**1068. **Hyparrhenia cymbaria** (L.) Stapf, Fl. Trop. Afr. [Oliver et al.] 9: 332. 1919 **

*Andropogon cymbarius* L., Mant. Pl. Altera 303. 1771 (as “cymbarium”). Type: “Habitat in India orientali. Koenig.” Lectotype: *Herb. Linn. No. 1211.6* (LINN). LT designated by Clayton in Kew Bull., Addit. Ser. 2: 110. 1969.

**Distribution.** Tamil Nadu [also in Africa].

**Habit.** Perennial.

**1069. **Hyparrhenia griffithii** Bor, Indian Forest Rec., Bot. n. s. 1(3): 92. 1938 **

*Andropogon filipendulus* sensu Hook. f., Fl. Brit. India (J.D. Hooker). 7(21): 210. 1896, non Hochst. 1846, p.p.

*Andropogon finitimus* sensu Hack., Monogr. Phan. [A. DC. & C. DC.] 6: 637. 1889, non Hochst. ex A. Rich., 1851

**Type.** India: Assam, Khasi and Jaintia Hills; 1837; *Griffith 6766* (K).

**Distribution.** Meghalaya. [China, Myanmar].

**Habit.** Perennial.

**1070. **Hyparrhenia hirta** (L.) Stapf, Fl. Trop. Afr. [Oliver et al.] 9: 315. 1919 **

*Andropogon hirtus* L., Sp. Pl. 2: 1046. 1753 (as “hirtum”). Type: “Habitat in Lusitania, Sicilia, Smyrnae.” Lectotype: *Herb. Burser I: 119* (UPS). LT designated by Clayton in Kew Bull., Addit. Ser. 2: 75. 1969.

*Sorghum hirtum* (L.) Kuntze, Revis. Gen. Pl. 2: 792. 1891

*Trachypogon hirtus* (L.) Nees, Fl. Bras. Enum. Pl. 2(1): 346. 1829

*Cymbopogon hirtus* (L.) Nees ex Stapf, Bull. Misc. Inform. Kew 1907: 212. 1907

**Distribution.** Northwest India [China, Pakistan].

**Habit.** Perennial.

**Remarks.** Distribution in India not reported.

**1071. **Hyparrhenia rufa** (Nees) Stapf, Fl. Trop. Afr. [Oliver et al.] 9: 304. 1919 **

*Trachypogon rufus* Nees, Fl. Bras. Enum. Pl. 2(1): 345. 1829. Type: Brasilia: “Habitat in campis agrestibus provinciae Piauhiensis”; *Martius s.n. *(M).

*Andropogon rufus* (Nees) Kunth, Enum. Pl. (Kunth) 1: 492. 1833.

*Cymbopogon rufus* (Nees) Rendle, Cat. Afr. Pl. 2(1): 155. 1899

*Sorghum rufum* (Nees) Kuntze, Revis. Gen. Pl. 2: 792. 1891

**Distribution.** India [China, Myanmar]. Naturalized and weedy; native to Africa.

**Habit.** Perennial.

**Remarks.** Distribution in India not reported.

***Imperata* Cirillo, Pl. Rar. Neapol. 2: 26. 1792**

**Type. ***Imperata arundinacea* Cirillo, nom. illeg. (*Lagurus cylindricus* L., *Imperata cylindrica* (L.) P. Beauv.).

**1072. **Imperata cylindrica** (L.) P. Beauv., Ess. Agrostogr. 165, 166. 1812 var. **cylindrica

*Lagurus cylindricus* L., Syst. Nat., ed. 10. 2: 878. 1759. Type: “Habitat [Monspelii, Creta, Smyrnae. D. Gerardus.] Sp. Pl., ed. 2. 1: 120. 1762.” Lectotype: Herb. Linn. No. 96.2 (LINN). LT designated by Clayton & Renvoize in Polhill (ed.), Fl. Trop. E. Africa, Gramineae 3: 700. 1982.

*Saccharum cylindricum* (L.) Lam., Encycl. (Lamarck) 1(2): 594. 1785

*Saccharum laguroides* Pourr., Mem. Acad. Sci. Toulouse 3: 326. 1788

*Imperata arundinacea* Cirillo, Pl. Rar. Neapol. 2: 26, t. 11. 1792. Type: “Habitat in collibus argillosis, perennis.”

*Imperata arundinacea* Cirillo var. *europaea* sensu Asch. & Graebn., Syn. Mitteleur. Fl. [Ascherson & Graebner]. 2(1): 37. 1898, non Andersson, 1855

*Calamagrostis lagurus* Koeler, Descr. Gram. (Koeler) 112. 1802, nom. superfl. & illeg. for *Laguruscylindricus*

**Distribution.** Andaman and Nicobar Islands, Andhra Pradesh, Assam, Bihar, Gujarat, Himachal Pradesh, Jammu and Kashmir, Karnataka, Kerala, Maharashtra, Madhya Pradesh, Manipur, Meghalaya, Odisha, Punjab, Rajasthan, Sikkim, Tamil Nadu, Tripura, Uttar Pradesh, West Bengal [Afghanistan, Bhutan, China, Myanmar, Nepal, Pakistan, Sri Lanka].

**Habit.** Perennial.

**1073. **Imperata cylindrica** (L.) P. Beauv. var. **latifolia** (Hook. f.) C.E. Hubb., Joint Publ. Imp. Agric. Bur. 7: 12. 1944 **

*Imperata arundinacea* Cirillo var. *latifolia* Hook. f., Fl. Brit. India (J.D. Hooker). 7(21): 106. 1896. Type: Tropical Himalaya from Kuman to Assam.

*Imperata latifolia* (Hook. f.) L. Liou, Vasc. Pl. Hengduan Mountains 2: 2299. 1994

**Distribution.** Andhra Pradesh, Assam, Karnataka, Kerala, Madhya Pradesh, Maharashtra, Tamil Nadu, Tripura, Uttar Pradesh [Nepal].

**Habit.** Perennial.

**1074. **Imperata cylindrica** (L.) P. Beauv. var. **major** (Nees) C.E. Hubb., Grasses Mauritius 96. 1940 **

*Imperata koenigii* (Retz.) P. Beauv. var. *major* Nees, Fl. Afr. Austral. Ill. 90. 1841. Type: “In valle prope Fakoskraal inter Omgaziana et Omsamwubo alt. 1500–2000 ft.; *Drege s.n.*”

*Imperata allang* Jungh., Tijdschr. Natuurl. Gesch. Physiol. 7: 295. 1840. Type: Indonesia: Java, Regio, alt. 3000 ft.

*Imperata cylindrica* (L.) P. Beauv. subvar. *koenigii* (Retz.) Durand & Schinz, Consp. Fl. Afr. [T.A. Durand & H. Schinz] 5: 694. 1894

*Imperata koenigii* (Retz.) P. Beauv., Ess. Agrostogr. 165, 177. 1812

*Saccharum koenigii* Retz., Observ. Bot. (Retzius) 5: 16. 1788 (“1789”). Type: “Misit honor. Konig ab amicissimo Thunberg accepi sub nomine Sacchari spicati ex Japonia, *Konig s.n*.” “Crescit in Insula Nipon ubique vulgate, locis humidis iuxta rivos et alibi.”

*Imperata filifolia* Nees, Syn. Pl. Glumac. (Steudel) 1: 405. 1855. Type: India; Numer. List [Wallich] no. 8850-E as *I. Koeniggi*

*Imperata pedicellata* Steud., Flora 29: 22. 1846. Type: “Ob flosculos omnes pedicellatos forsan novi generic typus, obstat vero habitus cum reliques speciebus omnino conformis.”

*Saccharum spicatum* sensu Thunb., Fl. Jap. (Thunberg) 42. 1784, non L., 1753

**Distribution.** Andhra Pradesh, Karnataka, Kerala, Madhya Pradesh, Maharashtra, Tamil Nadu, Tripura, Uttar Pradesh [Afghanistan, China, Myanmar, Nepal, Pakistan, Sri Lanka].

**Habit.** Perennial.

***Ischaemum* L., Sp. Pl. 2: 1049. 1753**

**Lectotype. ***Ischaemum muticum* L. LT designated by Nash, N. Amer. Fl. 17: 94. 30 Jun 1909; affirmed by M.L. Green, Prop. Brit. Bot. 193. 1929.

**1075. **Ischaemum afrum** (J.F. Gmel.) Dandy, Fl. Pl. Anglo-Egyptian Sudan 3: 476. f. 120. 1956 **

*Andropogon afer *J.F. Gmel., Syst. Nat. 2: 166. 1791. Lectotype: dessins in Bruce, Travels 5: 47. 1790

*Andropogon pilosus* Klein ex Willd., Sp. Pl., ed. 4 [Willdenow] 4: 920. 1806. Type: “Habitat in India orientali Mysore.”

*Ischaemum pilosum* (Klein ex Willd.) Wight, Madras Lit. Sci. J. 138. 1835 (also reprint p. 2, t. 2.)

*Andropogon pilifer* Steud., Syn. Pl. Glumac. 1: 373. 1854. Type: India; *Heyne s.n. as A. pilosus* (Numer. List [Wallich] no. 8817)

*Spodiopogon pilosus* Nees ex Steud., Syn. Pl. Glumac. 1: 373. 1854, nom. inval., pro. syn.

**Distribution.** Andhra Pradesh, Gujarat, Karnataka, Madhya Pradesh, Maharashtra, Rajasthan, Tamil Nadu [China, Myanmar].

**Habit.** Perennial.

**1076. **Ischaemum agastyamalayanum** Sreek., Janarth. & A. N. Henry, J. Bombay Nat. Hist. Soc. 84: 643. 1988 **

**Type.** India: Kerala, Trivandrum District, Western slopes of Agastyamalai, alt. 6000 ft.; October 6, 1973; *J. Joseph 44634* (CAL).

**Distribution.** Kerala.

**Habit.** Perennial.

**1077. **Ischaemum barbatum** Retz., Observ. Bot. (Retzius) 6: 35. 1791 **

*Ischaemum aristatum* L. var. *mangaluricum* Hack., Monogr. Phan. [A. DC. & C. DC.] 6: 204. 1889. Type: Peninsular India: Mangalur; *R.F. Hohenacker 184*

*Ischaemum mangaluricum* (Hack.) Stapf ex C.E.C. Fisch., Fl. Madras 3(10): 1723. 1934

*Ischaemum aristatum* L. var. *imbricatum* Hack., Monogr. Phan. [A. DC. & C. DC.] 6: 203. 1889. Syntypes: India: Khasia Mountains, alt. 4000 ft.; *J.D. Hooker & Thomson s.n.* Sri Lanka; *Thwaites 2625*

*Ischaemum imbricatum* (Hack.) Stapf ex Ridl., Fl. Malay. Penin. 5: 200. 1925

*Ischaemum aristatum* L. var. *fallax* Hack., Monogr. Phan. [A. DC. & C. DC.] 6: 204. 1889. Syntypes: Peninsular India; *Wight s.n.* Jabalpur; *Kuntze s.n.* Sri Lanka; *Wight 2553, Thwaites 700*. Macassar; *Wichura s.n.*

*Ischaemum geniculatum* Roxb., Fl. Ind. (Carey and Wallich ed.) 1: 324. 1820. Type: India: Bengal

*Ischaemum goebelii* Hack., Oesterr. Bot. Z. 51: 149. 1901. Type: Sri Lanka: Colombo; *Goebel s.n.*

*Ischaemum aristatum* sensu Hack., Handb. Fl. Ceylon 5: 211. 1900, non L., 1753

*Ischaemum abrahamii* Ravi, N. Mohanan & R. Rajesh, Bot. Bull. Acad. Sin. (Taipei) 42(3): 225. f. 2. 2001. Type: India, Kerala, Malappuram District, Manathumangalam, alt. 160 ft.; December 12, 1996; *Ravi 33027* (TBGT).

*Ischaemum wayanadense* Ravi, N. Mohanan & Shaju, Bot. Bull. Acad. Sin. (Taipei) 42(3): 223. f. 1. 2001. Type: India: Kerala, Wayand District, Kelloor, Thekkumpara, 2500 ft.; December 14, 1996; *Ravi 33779* (TBGT).

**Type.** “E Jaua accepi a pl. rev. D. Wennerbeg.” Indonesia: Java; *Wennerberg s.n.*

**Distribution.** Goa, Karnataka, Kerala, Maharashtra, Odisha, Sikkim, Tamil Nadu, West Bengal [Bangladesh, China, Myanmar, Sri Lanka].

**Habit.** Perennial.

**1078. **Ischaemum bolei** M.R. Almeida, Indian Forester 98(4): 236. f. 1–11. 1972 **

**Type.** India: Maharashtra, Ratnagiri District, Savantwadi; November 18, 1970; *M.R. Almeida 1535* (BLAT).

**Distribution.** Maharashtra.

**Habit.** Annual.

**1079. **Ischaemum bombaiense** Bor, J. Bombay Nat. Hist. Soc. 49: 165. 1950 **

**Type.** India: Bombay, Khandala, Tata’s Lake; *Blatter 9904* (K).

**Distribution.** Maharashtra, Rajasthan.

**Habit.** Annual.

**Remarks.** Record for Rajasthan fide Naithani (1990).

**1080. **Ischaemum calicutense** Sreek., V.J. Nair, & N.C. Nair, J. Econ. Taxon. Bot. 4(3): 1007–1009. f. 1–16. 1983 (as “calicutensis”) **

**Type.** India: Kerala, Calicut District, Pokkunnamalai, near Nanminda alt. 2800 ft.; October 29, 1981; *P.V. Sreekumar 71803* (CAL).

**Distribution.** Kerala.

**Habit.** Perennial.

**1081. **Ischaemum cannanorense** Sreek., V.J. Nair, & N.C. Nair, J. Econ. Taxon. Bot. 4(3): 1009–1011. f. 1–18. 1983 (as “cannanorensis”) **

**Type.** India: Kerala, Cannanore District, Paramba, on the way to Bendudka, alt. 500 ft.; October 16, 1981; *P.V. Sreekumar 71713* (CAL).

**Distribution.** Kerala.

**Habit.** Perennial.

**1082. **Ischaemum ciliare** Retz., Observ. Bot. (Retzius) 6: 36. 1791 **

*Andropogon asthenos* Steud., Syn. Pl. Glumac. 1: 376. 1854

*Andropogon malacophyllus* Hochst. ex Steud., Syn. Pl. Glumac. 1: 372. 1854. Type: India: Nilgiri Mountains; 1851; *R.F. Hohenacker 917* (S-G400).

*Ischaemum indicum* (Houtt.) Merr. subvar. *malacophyllum* (Hochst. ex Steud.) Bor, Grasses Burma, Ceylon, India and Pakistan 180. 1960

*Ischaemum ciliare* Retz. var. *prorepens* Hack., Monogr. Phan. [A. DC. & C. DC.] 6: 226. 1889. Type: cited many Syntypes

*Ischaemum indicum* (Houtt.) Merr. subvar. *villosum* (Nees) Bor, Grasses Burma, Ceylon, India and Pakistan 180. 1960

*Spodiopogon villosus* Hook & Arn., Bot. Beechey Voy. 6: 242. 1838. Type: “Hab. Prope Macao.”

*Ischaemum indicum* (Houtt.) Merr. subvar. *scorbiculatum* (Hack.) Bor, Grasses Burma, Ceylon, India and Pakistan 180. 1960

*Ischaemum ciliare* Retz. var. *scrobiculatum* Hack., Monogr. Phan. [A. DC. & C. DC.] 6: 226. 1889 (as “subvar”)

*Ischaemum tenellum* Roxb., Fl. Ind. (Carey and Wallich ed.) 1: 324–325. 1820. Type: Not given

*Spodiopogon obliquivalvis* Nees, Nov. Actorum Acad. Caes. Leop.-Carol. Nat. Cur. 19, Suppl. 1: 185. 1843, nom. superfl. & illeg. for Ischaemum ciliare Retz.

*Spodiopogon scrobiculatus* Nees ex Steud., Syn. Pl. Glumac. 1: 373. 1854, nom. invalid., pro. syn.

*Ischaemum indicum* (Houtt.) Merr. var. *wallichi* (Hack.) Bor, Grasses Burma, Ceylon, India and Pakistan 180. 1860

*Ischaemum ciliare* Retz. var. *wallichii* Hack., Monogr. Phan. [A. DC. & C. DC.] 6: 227. 1889. Type: [Bangladesh]: Silhet; Numer. List [Wallich] no. 8860B

**Type.** “E. China accepi.”

**Distribution. **Andhra Pradesh, Assam, Bihar, Dadra and Nagar Haveli, Goa, Gujarat, Karnataka, Kerala, Madhya Pradesh, Maharashtra, Odisha, Tamil Nadu, Tripura, Uttar Pradesh, West Bengal [China, Sri Lanka].

**Habit.** Perennial.

**Remarks.***Ischaemum ciliare* Retz. is sometimes treated as a synonym of *I. indicum* (Houtt.) Merr. However, the type of *I. indicum* (Houtt.) Merr. is *Polytrias indica*, and thus the epithet *indicum* is misapplied to *I. ciliare*.

**1083. **Ischaemum commutatum** Hack. ex Trimen, Syst. Cat. Fl. Pl. Ceylon 107. 1885. **

*Ischaemum semisagittatum* sensu Thwaites, Enum. Pl. Zeyl. (Thwaites). 365. 1864, non Roxb., 1820

**Type.** Sri Lanka; *Thwaites 2625.*

**Distribution.** Karnataka, Kerala, Maharashtra, Tamil Nadu [Sri Lanka].

**Habit.** Perennial.

**1084. **Ischaemum copeanum** Sreek., V.J. Nair & N.C. Nair, J. Bombay Nat. Hist. Soc. 82: 390. f. 1. 1985 **

*Ischaemum keralense* Sreek., V.J. Nair & N.C. Nair, Bull. Mus. Natl. Hist. Nat., B, Adansonia 4e Ser. 7, 2: 135. t. 1. 1985 (as “keralensis”). Type: India: Kerala, Cannanore District, Cherkala, alt. 800 ft.; November 24, 1981; *P.V. Sreekumar 71839 *(CAL).

**Type.** India: Kerala, Cannanore District, Cherkala, alt. 800 ft.; November 24, 1981; *V. Sreekumar 71838* (CAL).

**Distribution.** Kerala.

**Habit.** Annual.

**1085. **Ischaemum dalzellii** Stapf ex Bor, Kew Bull. 1951: 448. 1952 **

*Ischaemum conjugatum* sensu Thwaites, Enum. Pl. Zeyl. (Thwaites). 365. 1864, non Roxb, 1820

*Ischaemum semisagittatum* sensu Hook. f., Handb. Fl. Ceylon 5: 213. 1900, non Roxb., 1832

**Type.** India: Bombay, Yellapur, “roadsides in forest, common”; October 25, 1884; *W.A. Talbot 738* (K).

**Distribution.** Karnataka, Kerala, Maharashtra.

**Habit.** Annual.

**1086. **Ischaemum diplopogon** Hook. f., Fl. Brit. India (J.D. Hooker). 7(21): 129. 1896 **

**Type.** India: Bombay, near Bhorkus; *Woodrow s.n.* (K).

**Distribution.** Gujarat, Maharashtra, Rajasthan.

**Habit.** Annual.

**1087. **Ischaemum elimalayanum** Sreek., V.J. Nair & N.C. Nair, Fl. Kerala - Grasses 459, 134. t. 26. 1991 **

**Type.** India: Kerala, Cannanore District, Elimala, 960 ft.; October 20, 1981; *P.V. Sreekumar 71773* (CAL).

**Distribution.** Kerala.

**Habit.** Perennial.

**1088. **Ischaemum flumineum** Bor, Kew Bull. 1949: 572. 1950 **

**Type.** India: Bombay, Jog; April 28, 1939; *N.L. Bor 11390* (K, DD).

**Distribution.** Karnataka, Maharashtra, Tamil Nadu.

**Habit.** Perennial.

**Remarks.** Records for Karnataka and Tamil Nadu fide Naithani (1990).

**1089. **Ischaemum heterotrichum** Hack., Monogr. Phan. [A. DC. & C. DC.] 6: 220. 1889 **

**Syntypes.** Madagascar: Mayotte, Nosibe; *Boivin s.n.* Nicobares; *Jelinek s.n.*

**Distribution.** Andaman and Nicobar Islands [China].

**Habit.** Perennial.

**1090. **Ischaemum hubbardii** Bor, Indian Forest Rec., Bot. n.s. 1(3): 98. 1938 **

**Type.** India: Assam, Khasi and Jaintia Hills, Cherrapungi Plateu, alt. 4000–5000 ft.; *N.L. Bor s.n. *(K?).

**Distribution.** Assam, Meghalaya.

**Habit.** Perennial.

**Remarks. **Record for Meghalaya fide Naithani (1990).

**1091. **Ischaemum huegelii** Hack., Monogr. Phan. [A. DC. & C. DC.] 6: 252. 1889 **

*Ischaemum inerme* Stapf ex Bor, Kew Bull. 1951: 448. 1952. Type: India: Bombay, Bandra; October 25, 1896; *R.C. Wroughton s.n.* (K).

**Type.** India orientalis; *Hügel 1658* (W).

**Distribution.** Maharashtra.

**Habit. **Annual.

**1092. **Ischaemum impressum** Hack., Monogr. Phan. [A. DC. & C. DC.] 6: 210. 1889 **

**Type.** Asia; *Hügel 4018.*

**Distribution.** Delhi, Karnataka, Maharashtra, Rajasthan.

**Habit.** Annual.

**1093. **Ischaemum jayachandranii** R. Ansari, V.S. Ramach. & Sreek., Curr. Sci. 53(3): 151. 1984 **

**Type.** India: Kerala, Cannanore District, Nileswar, alt. 500 ft.; January 29, 1979; *V.J. Nair 59981* (CAL).

**Distribution. **Kerala.

**Habit.** Perennial.

**1094. **Ischaemum kingii** Hook. f., Fl. Brit. India (J.D. Hooker). 7(21): 129. 1896 **

**Type.** India: Bombay, Rajpootana, Mt. Aboo; *King s.n.* (K).

**Distribution.** Maharashtra, Rajasthan.

**Habit.** Annual.

**1095. **Ischaemum koenigii** (Hook. f.) Stapf ex C.E.C. Fisch., Fl. Madras 3(10): 1719, 1722. 1934 **

*Ischaemum aristatum* L. ssp. *koenigii* Hook. f., Fl. Brit. India (J.D. Hooker). 7(21): 127. 1896. Type: India: Precise locality unknown; *J. Koenig s.n.*

*Ischaemum fasciculatum* Rottl. ex Hook. f., Fl. Brit. India (J.D. Hooker). 7(21): 127. 1896, nom. nud., used as synonym

**Distribution.** Tamil Nadu.

**Habit. **Perennial.

**1096. **Ischaemum kumarakodiense** Ravi, N. Mohanan & Kiran Raj, Rheedea 8(2): 150. 1998 (as “kumarakodiensis”)  **

**Type.** India: Kerala, Alappuzha District, Pallana; December 29, 1996; *TBG &RI 33084* (TBGT).

**Distribution.** Kerala.

**Habit.** Perennial.

**1097. **Ischaemum lanatum** Ravi, N. Mohanan & Shaju, Rheedea 10(2): 91. 2000 **

**Type. **India: Kerala, Kasaragode District, Hosdurg Taluk, Periya, alt. 160 ft.; November 29, 1999; *Ravi 41535* (TBGT).

**Distribution.** Andhra Pradesh, Kerala, Tamil Nadu.

**Habit.** Annual.

**1098. **Ischaemum lisboae** Hook. f., Fl. Brit. India (J.D. Hooker). 7(21): 133. 1896 **

*Ischaemum aristatum* L. var. *barberi* C.E.C. Fisch., Fl. Madras 10(3): 1722. 1934. Type: India: Mangalore; *Barber no. 4803*

**Type.** India: North Canara; *Lisboa s.n.*

**Distribution.** Karnataka, Kerala.

**Habit.** Perennial.

**1099. **Ischaemum malabaricum** Sreek., V.J. Nair & N.C. Nair, Kew Bull. 39(4): 743. f. 1. 1984 **

**Type.** India: Kerala, Cannanore District, Paramba, alt. 500 ft.; October 16, 1981; *Sreekumar 71720* (CAL).

**Distribution.** Kerala.

**Habit. **Annual.

**1100**. Ischaemum molle** Hook. f., Fl. Brit. India (J.D. Hooker). 7(21): 128. 1896 **

**Syntypes.** India: Concan; *Dalzell s.n.* Central Provinces, Chanda District; *Duthie s.n.*

**Distribution.** Gujarat, Karnataka, Kerala, Maharashtra, Rajasthan, Tamil Nadu [Pakistan].

**Habit.** Perennial.

**1101. **Ischaemum muticum** L., Sp. Pl. 2: 1049. 1753 var. **muticum

*Andropogon polymorphus* Steud., Syn. Pl. Glumac. 1: 375. 1854. Type: Java: “Ischaemum muticum Zolling Morit Verz. P. 99 no. 1216.”

*Andropogon relictus* Steud., Syn. Pl. Glumac. 1: 377. 1854. Type: Philippines

*Ischaemum repens* Roxb., Fl. Ind. (Carey and Wallich ed.) 1: 325. 1820. Type: “A native of Pulopenang.”

*Andropogon repens* (Roxb.) Steud., Syn. Pl. Glumac. 1: 374. 1854

*Ischaemum glabratum* J. Presl in C. Presl, Reliq. Haenk. 1(4–5): 328. 1830. Type: “Habitat in insulis Philippines.” Philippines; *Haenke s.n.*

*Andropogon muticus* (L.) Steud., Syn. Pl. Glumac. 1: 374. 1854, non. L., 1763

**Type.** “Habitat in India.” Lectotype: *Herb. Linn. No. 1214.1*(LINN). LT designated by Renvoize in Jarvis et al. (ed.), Regnum Veg. 127: 57. 1993.

**Distribution.** Andaman and Nicobar, Karnataka, Kerala, Lakshadweep, Maharashtra, Tamil Nadu [China, Myanmar, Sri Lanka].

**Habit.** Perennial.

**1102. **Ischaemum nairii** V.J. Nair & Sreek., J. Econ. Taxon. Bot. 5(5): 1205. f. 1. 1984 **

**Type.** India: Kerala, Calicut District, Kanjeerakadavu, near Kakkayam, alt. 3000; November 26, 1981; *P.V. Sreekumar 71843* (CAL).

**Distribution.** Kerala.

**Habit.** Perennial.

**1103. **Ischaemum pappinisseriense** Ravi, N. Mohanan & Kiran Raj, Rheedea 8(2): 155. 1998 (as “pappinisseriensis”) **

**Type.** India: Kerala, Kannuar District, Pappinisseri; December 12, 1996; *Ravi TBG & RI 33707* (TBGT).

**Distribution.** Kerala.

**Habit.** Annual.

**1104. **Ischaemum polystachyum** J. Presl, Reliq. Haenk. 1(4–5): 328. 1830 **

*Ischaemum digitatum *Brongn*.* var. *polystachyum* (J. Presl) Hack., Monogr. Phan. [A. DC. & C. DC.] 6: 233. 1889

*Ischaemum digitatum* Brongn., Voy. Monde 2(2): 70, pl. 13. 1831. Type: Moluccas, Buru.

*Ischaemum duthiei* Stapf ex Bor, Kew Bull. 1950: 188. 1950. Type: India: Bihar, Neterhat, 3200 ft., along streams; October 19, 1918; *H.H. Haines 5856*

*Ischaemum fasciculatum* Brongn., Voy. Monde 2(2): 73. 1831

*Ischaemum hirtum* Hack., Monogr. Phan. [A. DC. & C. DC.] 6: 228. 1889. Type: India: Khasia Mountain, alt. 6000 ft.; *J.D. Hooker & Thomson Spodiopogon no. 13*

*Ischaemum lacei *Stapf ex Bor, Kew Bull. 1950: 187. 1950. Type: Burma: Amherst, Daiona Range, Muleyit Peak, alt. 6400 ft.; January 27, 1912; *J.A. Lace 5627* (K).

*Ischaemum nilagiricum* Hack., Oesterr. Bot. Z. 51: 150. 1901. Type: India: Nilgherries Mountains ad Canoor, 5500 ft.; March 13, 1870; *Clarke 10791*

*Ischaemum hirtum* sensu Hook. f., Fl. Brit. India (J.D. Hooker). 7(21): 135. 1896, non Hack., 1889

**Type. **Mariana Islands, *Haenke s.n*.

**Distribution.** Assam, Andhra Pradesh, Bihar, Himachal Pradesh, Karnataka, Kerala, Madhya Pradesh, Meghalaya, Tamil Nadu, West Bengal [Sri Lanka].

**Habit.** Perennial.

**Remarks.** Records for Himachal Pradesh, Madhya Pradesh, and West Bengal fide Naithani (1990).

**1105. **Ischaemum pushpangadanii** Ravi, N. Mohanan & Kiran Raj, Rheedea 10(1): 49. f. 1. 2000 **

*Ischaemum glabriglaucum* Sunil, Candollea 60(2): 387–391. f. 1–2. 2005. Type: India: Kerala, Trichur District, Vazhachal, 1000 ft.; October 4, 1999; *Sunil 2235* (K).

**Type.** India: Kerala, Kozhikode District, Koilandy Taluk, Kakkayam Hills, 1400 ft.; October 26, 1999; *Ravi 41460* (TBGT).

**Distribution.** Kerala.

**Habit. **Annual.

**1106. **Ischaemum quilonense** Ravi & Shaju, Rheedea 8(2): 152. 1998 (as “quilonensis”) **

**Type.** India: Kerala, Kollam District, Perumkulam Ela near Kollam Town; October 27, 1996; *Ravi TBG& RI 24098* (TBGT).

**Distribution.** Kerala.

**Habit.** Perennial.

**1107. **Ischaemum raizadae** Hemadri & Billore, Indian Forester 96(4): 318. 1970 **

*Ischaemum borii* M.R. Almeida, J. Bombay Nat. Hist. Soc. 66: 513. f. 1. 1970. Type: India: Maharashtra, Ratnagiri, Savantwadi, Amboli; December 24, 1968; *M. R. Almeida 895* (BLAT).

**Type.** India: Maharashtra, Thana District, Harishchandragarh, Sadrya ghat; November 16, 1968; *K.V. Billore 115450A* (CAL).

**Distribution.** Karnataka, Maharashtra.

**Habit.** Annual.

**Remarks.** Record for Karnataka fide Naithani (1990). It is unclear whether these two species are distinct or not. Clayton et al. (2006 onwards) recognize *I. borii* but not *I raizadae*.

**1108. **Ischaemum rangacharianum** C.E.C. Fisch., Bull. Misc. Inform. Kew 1933: 352. 1933 **

*Ischaemum fischeri* Ravi & Kiran Raj, Bot. Bull. Acad. Sin. (Taipei) 42(3): 227. 2001. Type: India: Kerala, Thiruvananthapuram District, Palode-TBGRI campus, 500 ft.; February 12, 1998; *Ravi 36706* (TBGT).

*Ischaemum aristatum* sensu Ranga, Handb. S. Ind. Grasses 151. 1921, non L., 1753

**Type.** India: Malabar District, Shoranur; November; *K. Ranga Achariyar s.n.* (K).

**Distribution.** Andhra Pradesh, Kerala, Maharashtra, Tamil Nadu.

**Habit.** Annual.

**Remarks.** Record for Kerala and Tamil Nadu fide Naithani (1990).

**1109. **Ischaemum raui** Sreek., V.J. Nair & N.C. Nair, Fl. Ind. ser. 2., Grasses: 459, 157. 1991 **

**Type.** India: Kerala, Cannanore District, Muguroad, alt. 500 ft.; October 17, 1981; *P.V. Sreekumar 71747* (CAL).

**Distribution.** Kerala.

**Habit.** Annual.

**1110. **Ischaemum ritchiei** Stapf ex Bor, Kew Bull. 1951: 449. 1952 **

**Type.** India: Bombay, Kanara; *Ritchie s.n. *(fide Munro) (K).

**Distribution.** Karnataka, Maharashtra.

**Habit.** Annual.

**Remarks.** Record for Karnataka fide Naithani (1990).

**1111. **Ischaemum rugosum** Salisb., Icon. Stirp. Rar. 1, t. 1. 1791 var. **rugosum

*Meoschium rugosum* (Salisb.) Arn. & Nees, Nov. Actorum Acad. Caes. Leop.-Carol. Nat. Cur., Suppl. 19(1): 200. 1843

*Meoschium griffithii* Nees & Arn., Ann. Mag. Nat. Hist. 1: 284. 1838. Type: India: Madras; *Griffith s.n.*

*Andropogon griffithsiae* (Nees ex Arn.) Steud., Syn. Pl. Glumac. 1: 375. 1854

*Andropogon tong-dong* Steud., Syn. Pl. Glumac. 1: 375. 1854. Syntypes: Nepal, India: Silhet; Numer. List [Wallich] no. 8864, F.H.J. Tong-Dong. *Wall. Ind. Burm. no. 1013*

*Colladoa distachia* Cav., Icon. (Cavanilles) 5(1): 37, t. 460. 1799. Type: “Habitat in humidis memoratae insulae Mindanao prope Samboangan. Vidi siccam in dicto herbario.”

*Meoschium arnottianum* Nees, Nov. Actorum Acad. Caes. Leop.-Carol. Nat. Cur., Suppl. 19(1): 198. 1843. Type: “Peninsula Indiae orientalis, ad Madras, *Mrs Griffiths in Herb. Lindl*.” Wight Cat. no. 1720

*Andropogon arnottianus* (Nees) Steud., Syn. Pl. Glumac. 1: 375. 1854

*Meoschium wightianum* Nees, Nov. Actorum Acad. Caes. Leop.-Carol. Nat. Cur., Suppl. 19(1): 199. 1843, nom. invalid, cited as synonym of *Meoschium elegans*

*Ischaemum colladoa* Spreng., Syst. Veg. (ed. 16) [Sprengel] 1: 298. 1824 (“1825”), nom. superfl. & illeg. for *Colladoa distachya* Cav.

*Andropogon segetum* Steud., Syn. Pl. Glumac. 1: 376. 1854

*Ischaemum segetum* Trin., Mém. Acad. Imp. Sci. St.-Pétersbourg, Sér. 6, Sci. Math. 2: 294. 1832, nom. superfl. and illeg.? Type: Philippines: Manila

*Meoschium royleanum* Nees ex Steud., Syn. Pl. Glumac. 1: 375. 1854, nom. inval., pro. syn.

**Type.** “Sponte nascentem in Orissa ad margines agrorum oryzaceorum”, India: Orissa, *J. Koenig s.n. *(BM).

**Distribution.** Andaman and Nicobar Islands, Andhra Pradesh, Assam, Bihar, Delhi, Dadra and Nagar Haveli, Gujarat, Karnataka, Kerala, Madhya Pradesh, Maharashtra, Odisha, Rajasthan, Sikkim, Tamil Nadu, Tripura, Uttar Pradesh, West Bengal [China, Myanmar, Pakistan, Sri Lanka].

**Habit. **Annual.

**1112. **Ischaemum santapaui** Bor, J. Bombay Nat. Hist. Soc. 49: 167. 1950 **

**Type.** India: Bombay Presidency, Karjat; December 11, 1949; *H. Santapau 9665* (K).

**Distribution.** Maharashtra.

**Habit.** Annual.

**1113. **Ischaemum sayajiraoi** Raole & R.J. Desai, Kew Bull. 66(2): 303–306, f. 1. 2011 **

**Type. **India: Gujarat, Vadodara district, Bacrol, 3 October 2008*, R. J. Desai 32* (holotype BARO; isotype: K).

**Distribution.** Gujarat.

**Habit.** Annual.

**1114. **Ischaemum semisagittatum** Roxb., Fl. Ind. (Carey & Wallich ed.) 1: 322–323. 1820 **

*Andropogon semisagittatus* (Roxb.) Steud., Syn. Pl. Glumac. 1: 376. 1854

*Meoschium semisagittatum* (Roxb.) Schult., Mant. 2 (Schultes) 435. 1824

*Spodiopogon semisagittatus* (Roxb.) Voigt, Hort. Suburb. Calcutta 706. 1845

*Andropogon cordatifolius* Steud., Syn. Pl. Glumac. 1: 376. 1854

*Ischaemum conjugatum* Roxb., Fl. Ind. (Carey and Wallich ed.) 1: 323. 1820 (as “conjugatam”). Type: India: “A native of pasture land in the vicinity of Calcutta.”

*Spodiopogon conjugatus* (Roxb.) Voigt, Hort. Suburb. Calcutta 706. 1845

**Type.** India: “A native of newly formed pasture land in Bengal.”

**Distribution.** Andhra Pradesh, Dadra and Nagar Haveli, Daman and Diu, Goa, Karnataka, Kerala, Madhya Pradesh, Maharashtra, West Bengal [Sri Lanka].

**Habit.** Annual.

**1115. **Ischaemum sreenarayanii** Sunil, Nithya & Remya Kr., Bangladesh J. Pl. Taxon. 24(1): 33. 2017 **

**Type.** India: Kerala, Thrissur district, Kombazha, 11 m, 8 December 2015, *Sunil & Nithya 9007* (holotype: MH; isotype: CALI, SNMH).

**Distribution.** Kerala.

**Habit. **Perennial.

**1116. **Ischaemum tadulingamii** N.C. Nair & Sreek., Blumea 30(2): 385. f. 1. 1985 **

**Type.** India: Kerala State, Idukki District, Eravikulam National Park, alt. 7000 ft.; April 7, 1980; *Sreekumar 71863* (CAL).

**Distribution.** Kerala, Tamil Nadu.

**Habit. **Annual or Perennial.

**1117. **Ischaemum thomsonianum** Stapf ex C.E.C. Fisch., Fl. Madras 3(10): 1722. 1934 **

*Ischaemum murinum* sensu Hook. f., Fl. Brit. India (J.D. Hooker). 7(21): 135. 1896, non G. Forst., 1786

**Type.** India: Mysoore or the Carnatic; *Thomson s.n. *

**Distribution.** Karnataka, Kerala, Maharashtra, Tamil Nadu [China, Myanmar].

**Habit.** Annual.

**1118. **Ischaemum timorense** Kunth, Révis. Gramin. 1: 369. t. 98. 1830 var. **timorense

*Andropogon timorensis* (Kunth) Steud., Syn. Pl. Glumac. 1: 376. 1854

*Andropogon blumii* Steud., Syn. Pl. Glumac. 1: 373. 1854. Type: Java, Ceylon, Burma: Martaban; Numer. List [Wallich] no. 8863

*Spodiopogon blumii* Nees ex Steud., Syn. Pl. Glumac. 1: 373. 1854, nom. invalid, as syn. of *Andropogon blumii* Nees; mpt. sub: Spodiopogon.

**Type.** “Crescit in insula Timor.”

**Distribution.** Assam, Bihar, Gujarat, Karnataka, Kerala, Maharashtra, Meghalaya, Tamil Nadu, Tripura, West Bengal [Bangladesh, China, Myanmar, Pakistan, Sri Lanka].

**Habit.** Annual.

**1119. **Ischaemum timorense** Kunth var. **zeylanicum** Hack., Monogr. Phan. [A. DC. & C. DC.] 6: 230. 1889 **

*Ischaemum zeylanicola* Bor, Grasses Burma, Ceylon, India and Pakistan 186. 1960

**Type.** Sri Lanka; *Thwaites 3168.*

**Distribution. **Andaman and Nicobar Islands, Karnataka, Kerala, Maharashtra, Tamil Nadu [Sri Lanka].

**Habit.** Annual.

**Remarks.** Records for Andaman and Nicobar Islands, Kerala, Maharashtra, and Tamil Nadu fide Naithani (1990).

**1120. **Ischaemum travancorense** Stapf ex C.E.C. Fisch., Bull. Misc. Inform. Kew 1933: 353. 1933 **

*Ischaemum aristatum* L. ssp. *rottleri* Hook. f., Fl. Brit. India (J.D. Hooker). 7(21): 127. 1896. Type: India: Deccan; *Rottler s.n.*

**Type.** India: Bababudan Hills; April; *Sir A. G. and Lady Bourne s.n.* (K).

**Distribution.** Kerala, Tamil Nadu, Maharashtra.

**Habit.** Perennial.

**1121. **Ischaemum tumidum** Stapf ex Bor, Kew Bull. 1951: 450. 1952 **

**Type.** India: Madras, Concan; *Stocks s.n.* (K).

**Distribution.** Karnataka, Kerala, Madhya Pradesh, Maharashtra, Tamil Nadu.

**Habit.** Annual.

**Remarks.** All records for India except for Karnataka fide Naithani (1990).

**1122. **Ischaemum vembanadense** Patil & D’Cruz, J. Bombay Nat. Hist. Soc. 70: 324. f. 1–8. 1974 **

**Type.** India: Kerala, Allepy backwaters; January 10, 1970; *Patil s.n.* (BSI).

**Distribution. **Kerala.

**Habit.** Perennial.

**1123. **Ischaemum yadavii** Gad & Janarth., Kew Bull. 62(3): 499–501. f. 1. 2007 **

**Type.** India: Goa, Surla, Lateritic rocky plateau, 15°40'03"N, 74°10'28"E, alt. 2600 ft.; October 2, 2005; *Harshala Gad & M.K. Janarthanam 240* (CAL).

**Distribution.** Goa.

**Habit.** Annual.

***Iseilema* Andersson, Nova Acta Regiae Soc. Sci. Upsal. ser. 3. 2: 250. 1856**

**Type.***Iseilema prostratum* (L.) Andersson (*Andropogon prostratus *L. (as “*prostratum*”)).

**1124. **Iseilema anthephoroides** Hack., Monogr. Phan. [A. DC. & C. DC.] 6: 683. 1889 **

**Type.** India orientalis. Numer. List [Wallich] no. 8767 A. Peninsula; *Wight. 2335 *“leg. ?: via in h. Nees, ad Appiahpilly in ditione Cuddapah”. “d na Stead in h. m., pr. Scholapur.”

**Distribution.** Andhra Pradesh, Bihar, Daman and Diu, Dadra and Nagar Haveli, Gujarat, Karnataka, Kerala, Madhya Pradesh, Maharashtra, Odisha, Rajasthan, Tamil Nadu, Uttar Pradesh, Uttarakhand.

**Habit.** Annual.

**1125. **Iseilema hackelii** U. B. Shrestha & Gandhi, Harvard Pap. Bot. 13(2): 296. 2008 **

*Iseilema laxum* Hack., Monogr. Phan. [A. DC. & C. DC.] 6: 682. 1889, nom. illeg. later homonym non *I. laxum* Hack. ex Duthie, 1888

*Iseilema laxum* Hack. ex Duthie, Fodder Grasses North. India 43. 1888, p.p. Type: Protologue.

*Iseilema jainiana* P. Umam. & P. Daniel, J. Bombay Nat. Hist. Soc. 98(3): 425. 2001. Type: India: Tamil Nadu, Tuticorin District, Gulf of Mannar coast, Kanyakumari-Thiruchendur highway, between Karaichuthu and Padakkapathu diversion, c. 130 ft.; January 26, 1996; *P. Daniel; P. Umamaheswari 107240* (CAL).

**Distribution.** Andhra Pradesh, Bihar, Delhi, Chhattisgarh, Goa, Gujarat, Karnataka, Kerala, Madhya Pradesh, Maharashtra, Odisha, Rajasthan, Tamil Nadu, Uttar Pradesh, West Bengal [Sri Lanka].

**Habit.** Perennial.

**1126. **Iseilema holei** Haines, Bot. Bihar Orissa 5: 1055. 1924 **

**Type.** India: Palaman, in moist ground forest; *Haines s.n.*

**Distribution. **Bihar, Madhya Pradesh, Maharashtra.

**Habit. **Annual.

**Remarks.** All records for India fide Naithani (1990).

**1127. **Iseilema hubbardii** Uppuluri, J. Bombay Nat. Hist. Soc. 65: 665. 1969 **

**Type.** India. University campus of Ujjain, Madhya Pradesh. 22 November, 1966, *Murty field number IAU 3*. LT *U. Satyavathi – IAU 3*, designated by Drisya, V. & A. K. Pradeep. Phytotaxa 424(1): 56–60. 2019.

**Distribution.** Madhya Pradesh.

**Habit.** Annual.

**1128. **Iseilema prostratum** (L.) Andersson, Nova Acta Regiae Soc. Sci. Upsal. ser. 3. 2: 251. 1856 (as “prostrata”) **

*Andropogon prostratus* L., Mant. Pl. Altera 304. 1771 (as “prostratum”). Type: “Habitat in India orientali. Koenig.” Lectotype: *Herb. Linn. No. 1211.8* (LINN). LT designated by Renvoize in Cafferty et al. (ed.), Taxon 49: 246. 2000.

*Anthistiria prostrata* (L.) Willd., Sp. Pl., ed. 4 [Willdenow] 4: 901. 1806

*Anthistiria wightii* Nees, Syn. Pl. Glumac. (Steudel) 1: 400. 1855. Type: India Orientalis; Wight in Numer. List [Wallich] no. 8870

*Iseilema wightii* (Nees) Andersson, Nova Acta Regiae Soc. Sci. Upsal. ser. 3. 2: 251. 1856

*Cymbopogon glandulosus* Spreng., Pl. Min. Cogn. Pug. 2: 14. 1815, nom superfl. & illeg. for *Andropogon prostratus* L.

**Distribution.** Andhra Pradesh, Bihar, Chhattisgarh, Goa, Gujarat, Karnataka, Madhya Pradesh, Maharashtra, Rajasthan, Tamil Nadu, Uttar Pradesh [Myanmar, Pakistan, Sri Lanka].

**Habit.** Perennial.

**1129. **Iseilema venkateswarlui** Satyavathi, J. Bombay Nat. Hist. Soc. 65: 666–667. 1969 **

**Type.** India: Andhra Pradesh, Guntur, Lam Farm; September 26, 1966; *Satyavathi s.n.* (K).

**Distribution.** Andhra Pradesh.

**Habit.** Annual.

***Lasiurus* Boiss., Diagn. Pl. Orient. ser. 2. 4(4): 145. 1859**

**Type. ***Lasiurus hirsutus* Boiss., nom. illeg. (*Rottboellia hirsuta* Vahl, nom. illeg.; *Triticum aegilopoides* Forssk.).

**1130. **Lasiurus scindicus** Henrard, Blumea 4(3): 514. 1941 **

*Lasiurus ecaudatus* Satyanar. & Shank., J. Bombay Nat. Hist. Soc. 60: 763–764. 1964. Type: India: Rajastan, Jodhpur, C.R. Farm, Central Arid Zone Research Institute, alt. 800 ft.; January 7, 1962; *Y. Satyanarayan & K.A. Shankarnarayan 719* (BSJO).

*Elionurus hirsutus* Munro, J. Linn. Soc., Bot. 19: 68. 1881, p.p.

*Lasiurus hirsutus* Boiss., Diagn. Pl. Orient. ser. 2, 3(4): 146. 1859, p.p.

*Rottboellia hirsuta* Vahl, Symb. Bot. (Vahl) 1: 11. 1790, p.p.

**Type. **“India: Scind, leg Stocks. Herb. Ind. Or. Hook. fil. et Thomson. Typus in H. L. B. sub no. 908, 87–853.”

**Distribution.** Gujarat, Rajasthan [Afghanistan, Pakistan].

**Habit.** Perennial.

***Leptatherum* Nees, Proc. Linn. Soc. Lond. 1: 92. 1841**

**Type.***Leptatherum royleanum *Nees.

**1131. **Leptatherum nudum **(Trin.) C.Hui Chen, Kuoh & Veldkamp, Blumea 54(1–3): 179. 2009 **

*Pollinia nuda* Trin., Mém. Acad. Imp. Sci. St.-Pétersbourg, Sér. 6, Sci. Math. 2: 307. 1832. Type: Nepal; *Wallich s.n. inHerb. Hornem. *(LE).

*Microstegium nudum* (Trin.) A. Camus, Ann. Soc. Linn. Lyon ser. 2, 68: 201. 1921

*Eulalia nuda* (Trin.) Kuntze, Revis. Gen. Pl. 2: 775. 1891

*Leptatherum japonicum* Franch. & Sav., Enum. Pl. Jap. 2: 190. 1877. Type: Japan: circa Yokoska, in locis humidis, in orizariis; *Savatier 1507, 2557*

*Pollinia japonica* Miq., Ann. Mus. Bot. Lugduno-Batavi 2: 290. 1866. Type: Japan (“Nippon?”); *Keiske s.n.* (L).

*Leptatherum royleanum* Nees, Proc. Linn. Soc. London 1: 92–93. 1841

**Distribution.** Assam, Himachal Pradesh, Jammu and Kashmir, Kerala, Meghalaya, Nagaland, Sikkim, Tamil Nadu, Uttar Pradesh, Uttarakhand, West Bengal [Bhutan, China, Nepal, Pakistan].

**Habit.** Annual.

***Lophopogon* Hack., Nat. Pflanzenfam., ed. 2 [Engler & Prantl] 2(2): 26. 1887**

**Type. ***Lophopogon tridentatus* (Roxb.) Hack. (*Andropogon tridentatus* Roxb.).

**1132. **Lophopogon kingii** Hook. f., Fl. Brit. India (J.D. Hooker). 7(21): 149. 1896 **

**Type.** India: Bihar, Monghir; *Mokim 1408.*

**Distribution.** Bihar.

**Habit.** Perennial.

**1133. **Lophopogon tridentatus** (Roxb.) Hack., Monogr. Phan. [A. DC. & C. DC.] 6: 254. 1889 **

*Andropogon tridentatus* Roxb., Fl. Ind. (Carey and Wallich ed.) 1: 261. 1820. Type: India: Native of Coromandel; *Roxburgh s.n.*

*Apocopis tridentata* (Roxb.) Benth., J. Linn. Soc., Bot. 19: 67. 1881

*Saccharum tridentatum* (Roxb.) Spreng., Syst. Veg. (ed. 16) [Sprengel] 1: 283. 1824 (“1825”)

*Lophopogon duthiei* Stapf ex Bor, Grasses Burma, Ceylon, India and Pakistan 689. 1960. Type: India orientalis: Madhya Pradesh;* J.F. Duthie 8490* (K).

**Distribution.** Andhra Pradesh, Assam, Bihar, Gujarat, Karnataka, Madhya Pradesh, Maharashtra, Tamil Nadu.

**Habit.** Annual or perennial.

***Manisuris* L., Mant. Pl. Altera 164, 300. 1771**

**Type. ***Manisuris myurus* L.

**1134. **Manisuris myurus** L., Mant. Pl. Altera 300. 1771 **

*Rottboellia myurus* (L.) Benth., J. Linn. Soc., Bot. 19: 68. [indirect reference to Linnaean name]

*Peltophorus myurus* (L.) Desv., Nouv. Bull. Sci. Soc. Philom. Paris 2: 188. 1810

**Type. **“Habitat in India.” Lectotype: *Herb. Linn. No. 1215.2* (LINN). LT designated by Clayton in Cafferty et al. (ed.), Taxon 49: 252. 2000.

**Distribution.** Andhra Pradesh, Karnataka, Manipur, Tamil Nadu.

**Habit.** Perennial.

**Remarks.** Although *Manisuris* L. is rejected against *Rottboellia* L.f. (1782), recent studies recognize them as distinct genera.

***Microstegium* Nees, Nat. Syst. Bot., ed. 2 447. 1836**

**Type. ***Microstegium willdenovianum* Nees.

**1135. **Microstegium borianum** Sur, J. Bombay Nat. Hist. Soc. 79: 652. f. 1. 1983 (“1982”) **

**Type.** India.

**Distribution.** Meghalaya.

**Habit.** Perennial.

**1136. **Microstegium ciliatum** (Trin.) A. Camus, Ann. Soc. Linn. Lyon ser. 2, 68: 201. 1921. **

*Pollinia ciliata* Trin., Mém. Acad. Imp. Sci. St.-Pétersbourg, Sér. 6, Sci. Math. 2: 306. 1832. Type: Nepal; *Wallich ex hb. Hornem.* (LE).

*Eulalia ciliata* (Trin.) Kuntze, Revis. Gen. Pl. 2: 775. 1891

*Andropogon biaristatus* Steud., Syn. Pl. Glumac. 1: 379. 1854. Type: Nepal; Numer. List [Wallich] no. 8823

*Microstegium biaristatum* (Steud.) Keng, Sinensia 3(3): 92. 1932

*Pollinia monantha* Nees, Syn. Pl. Glumac. (Steudel) 1: 410. 1854. Type: India; Numer. List [Wallich] no. 8819 as “Andropogon”

*Eulalia monantha* (Nees) Kuntze, Revis. Gen. Pl. 2: 775. 1891

*Microstegium monanthum* (Nees) A. Camus, Ann. Soc. Linn. Lyon ser. 2, 68: 200. 1921

*Pollinia ciliata* Trin. var. *seminuda* Hack., Monogr. Phan. [A. DC. & C. DC.] 6: 177. 1889. Type: Sri Lanka; *Thwaites 411*

*Pollinia lancea* Nees, Syn. Pl. Glumac. (Steudel) 1: 410. 1854. Type: India; Numer. List [Wallich] no. 8815-B, Numer. List [Wallich] no. 8831

*Pollinia laxa* Nees, Syn. Pl. Glumac. (Steudel) 1: 410. 1854. Type: India; Numer. List [Wallich] no. 8823 as “Andropogon”

*Pollinia wallichiana* Nees, Syn. Pl. Glumac. (Steudel) 1: 410. 1854. Type: India: Sylhet; Numer. List [Wallich] no. 8822 as “Andropogon”

**Distribution.** Andhra Pradesh, Arunachal Pradesh, Assam, Bihar, Himachal Pradesh, Karnataka, Kerala, Madhya Pradesh, Maharashtra, Meghalaya, Nagaland, Odisha, Sikkim, Tamil Nadu, Uttar Pradesh, West Bengal [Bangladesh, Bhutan, China, Myanmar, Nepal, Sri Lanka].

**Habit.** Perennial.

**1137. **Microstegium eucnemis** (Nees) A. Camus, Ann. Soc. Linn. Lyon ser. 2, 68: 200. 1921. **

*Pollinia eucnemis* Nees, Syn. Pl. Glumac. (Steudel) 1: 409. 1854. Type: India; Numer. List [Wallich] no. 8812 as “Andropogon”

*Eulalia eucnemis* (Nees) Kuntze, Revis. Gen. Pl. 2: 775. 1891

*Coelarthron brandisii* Hook. f., Fl. Brit. India (J.D. Hooker). 7(21): 164. 1896. Type: Burma: high ground, generally with teak; 1883; *Brandis s.n. *(K).

*Microstegium brandisii* (Hook. f.) D. Rhind, Grass. Burma 62. 1945

**Distribution. **Tamil Nadu, Uttar Pradesh [China, Myanmar].

**Habit.** Perennial.

**1138. **Microstegium falconeri** (Hook. f.) Clayton, Kew Bull. 35(4): 816. 1981 **

*Ischnochloa falconeri* Hook. f., Hooker’s Icon. Pl. 25: t. 2466. 1896. Type: India: North-Western Himalaya, growing amongst moss; *Falconer s.n.* (K).

**Distribution.** India (North western Himalaya).

**Habit.** Annual.

**Remarks.** Distribution in India not reported.

**1139. **Microstegium fasciculatum** (L.) Henrard, Blumea 3(3): 453. 1940 **

*Andropogon fasciculatus* L., Sp. Pl. 2: 1047. 1753. Type: “Habitat in Indiis.”, Lectotype: Herb. Linn. No. 1211.27, lower central specimen (LINN). LT designated by Cope in Cafferty et al. (ed.), Taxon 49: 245. 2000. Original designation by Henrard in Blumea 3: 454. 1940

*Tripsacum fasciculatum* (L.) Raspail, Ann. Sci. Nat. (Paris) 5: 306. 1825

*Pollinia vagans* Nees, Syn. Pl. Glumac. (Steudel) 1: 410. 1854. Type: Nepal; Numer. List [Wallich] no. 8807-B

*Microstegium vagans* (Nees) A.Camus, Ann. Soc. Linn. Lyon ser. 2, 68: 200. 1921

*Eulalia vagans* (Nees) Kuntze, Revis. Gen. Pl. 2: 775. 1891

*Pollinia grata* Hack., Monogr. Phan. [A. DC. & C. DC.] 6: 175. 1889. Syntypes: India: Bengal, Silligori; *O. Kuntze s.n.* Sikkim, alt. 4500–6000 ft.; *J.D. Hooker & T. Thomson s.n.* Java; *Hoffmannsegg s.n.* (B) and many syntypes.

*Microstegium gratum* (Hack.) A. Camus, Ann. Soc. Linn. Lyon ser. 2, 68: 201. 1921

*Pollinia montana* Nees, Syn. Pl. Glumac. (Steudel) 1: 409. 1854. Type: Indonesia: Java, Mountains; *Junghuhn s.n.*

*Ephebopogon gratus* Nees & Mey. ex Steud., Nomencl. Bot., ed. 2 (Steudel) 1: 556. 1840, nom. nud.

**Distribution.** Arunachal Pradesh, Assam Meghalaya, Uttar Pradesh, Uttarakhand [Bhutan, China, Myanmar, Nepal].

**Habit.** Perennial.

**1140. **Microstegium petiolare** (Trin.) Bor, Indian Forest Rec., Bot. n.s. 1(3): 87. 1938 **

*Spodiopogon petiolaris* Trin., Mém. Acad. Imp. Sci. St.-Pétersbourg, Sér. 6, Sci. Math. 2: 301–302. 1832. Type: Nepal; *Wallich* (Numer. List [Wallich] no. 8807)

*Andropogon petiolaris* (Trin.) Steud., Syn. Pl. Glumac. 1: 398. 1854

*Ischaemum petiolare* (Trin.) Hack., Monogr. Phan. [A. DC. & C. DC.] 6: 238. 1889

*Pollinia lehmannii* Arn. & Nees, Nov. Actorum Acad. Caes. Leop.-Carol. Nat. Cur., Suppl. 19 (1): 186. 1843 (as “lehmanni”). Type: Not given.

*Spodiopogon lehmannii* (Arn. & Nees) Griseb., Nachr. Königl. Ges. Wiss. Georg-Augusts-Univ. 91. 1868 (as “lehmanni”)

**Distribution.** Assam, Chhattisgarh, Madhya Pradesh, Meghalaya [China, Myanmar, Nepal].

**Habit.** Perennial.

**1141. **Microstegium reticulatum** B. S. Sun ex H. Peng & X. Yang, Acta Phytotax. Sin. 34(2): 213–215, pl. 1. 1996 **

**Distribution.** North East India [China].

**Habit.** Annual.

**Remarks.** Distribution in India not reported.

**1142. **Microstegium vimineum** (Trin.) A. Camus, Ann. Soc. Linn. Lyon, ser. 2. 68: 201. 1921. **

*Andropogon vimineus* Trin., Mém. Acad. Imp. Sci. St.-Pétersbourg, Sér. 6, Sci. Math. 2: 268. 1832. Type: Nepal; *Wallich* (Numer. List [Wallich] no. 8838 (LE).

*Eulalia viminea* (Trin.) Kuntze, Revis. Gen. Pl. 2: 775. 1891

*Pollinia viminea* (Trin.) Merr., Enum. Philipp. Fl. Pl. 1: 35. 1923 (“1922”)

*Pollinia imberbis* Nees, Syn. Pl. Glumac. (Steudel) 1: 410. 1854. Type: Nepal; Numer. List [Wallich] no. 8832 as “Bathratherum”

*Microstegium willdenovianum* Nees in Lindley, Nat. Syst. Bot., ed. 2 447. 1836. Type: Nepal; Herb. Willd.

*Pollinia willdenoviana* (Nees) Benth., J. Linn. Soc., Bot. 19: 67. 1881 (as “willdenowianum”)

**Distribution.** Assam, Himachal Pradesh, Meghalaya, Sikkim [Bhutan, China, Myanmar, Nepal].

**Habit.** Annual.

***Miscanthus* Andersson, Öfvers. Förh. Kongl. Svenska Vetensk.-Akad. 12: 165. 1855**

**Lectotype. ***Miscanthus japonicus *(Trin.) Andersson (*Eulalia japonica *Trin.). Designated by Hitchcock, Bull. U.S.D.A. 772: 254. 1920.

**1143. **Miscanthus fuscus** (Roxb.) Benth., J. Linn. Soc., Bot. 19: 65. 1881 **

*Sclerostachya fusca* (Roxb.) A. Camus, Fl. Indo-Chine [P.H. Lecomte et al.] 7: 243. 1922

*Saccharum fuscum* Roxb., Fl. Ind. (Carey and Wallich ed.) 1: 241. 1920. Type: “A native of damp places over Bengal.”

*Eriochrysis fusca* (Roxb.) Trin., Mém. Acad. Imp. Sci. Saint-Pétersbourg, Sér. 6, Sci. Math., Seconde Pt. Sci. Nat. 2: 315. 1832

*Eriochrysis attenuata* Nees, Syn. Pl. Glumac. (Steudel) 1: 411. 1854

*Sclerostachya milroyi* Bor, Indian Forest Rec., Bot. n. s. 1(3): 85. 1938. Type: India: Assam, Sibsagar; *N.L. Bor s.n.*

**Distribution.** Assam, Arunachal Pradesh, Bihar, Manipur, Meghalaya, Uttar Pradesh, West Bengal [Bangladesh, China, Myanmar].

**Habit.** Perennial.

**1144. **Miscanthus nepalensis** (Trin.) Hack., Monogr. Phan. [A. DC. & C. DC.] 6: 104. 1889 **

*Eulalia nepalensis* Trin., Mém. Acad. Imp. Sci. St.-Pétersbourg, Sér. 6, Sci. Math. 2: 333. 1832. Type: Nepal.

**Distribution.** Arunachal Pradesh, Assam, Delhi, Himachal Pradesh, Meghalaya, Nagaland, Rajasthan, Sikkim, Tamil Nadu, Uttar Pradesh, Uttarakhand, West Bengal [Bhutan, China, Myanmar, Nepal].

**Habit.** Perennial.

**1145. **Miscanthus nudipes** (Griseb.) Hack., Monogr. Phan. [A. DC. & C. DC.] 6: 109. 1889 **

*Erianthus nudipes* Griseb., Nachr. Königl. Ges. Wiss. Georg-Augusts-Univ. 92. 1868. Type: Himalaya occidentalis: Sikkim, alt. 9000–12000 ft.; *J.D. Hooker & Thomson Erianthus no. 10*.

*Miscanthus taylorii* Bor, Kew Bull. 1953: 273–274. 1953. Type: China: Xizang, Kongbo Province, Trim La, Mayër, 12200 ft.; July 9, 1938; *Ludlow, Sherriff & Taylor 5799* (BM).

*Diandranthus taylorii* (Bor) L. Liu, Fl. Reipubl. Popularis Sin. 10(2): 14. 1997

**Distribution.** Assam, Sikkim [Bhutan, China, Nepal].

**Habit.** Perennial.

**1146. **Miscanthus wardii** Bor, Kew Bull. 1953: 273. 1953 **

**Type.** India: Assam, Di Chu Gorge, Lohit Valley; April 12, 1950; *F. Kingdon-Ward 19328* (K).

**Distribution. **Arunachal Pradesh (Naithani), Assam.

**Habit.** Perennial.

**Remarks.** Record for Arunachal Pradesh fide Naithani (1990).

***Mnesithea* Kunth, Rév. Gram. 1: 153. 1829**

**Type. ***Mnesithea laevis* (Retz.) Kunth (*Rottboellia laevis* Retz.).

**1147. **Mnesithea clarkei** (Hack.) de Koning & Sosef, Blumea 31(2): 290. 1986 **

*Rottboellia clarkei* Hack., Oesterr. Bot. Z. 41: 8. 1891. Type: India: Chota Nagpur, Parasnath, alt. 2000 ft.;* C.B. Clarke 21075* (W).

*Coelorachis clarkei* (Hack.) Blatt. & McCann, J. Bombay Nat. Hist. Soc. 32: 33. 1927 (as “Coelorrhachis”)

*Rottboellia gibbosa* Hack. ex Lisboa, J. Bombay Nat. Hist. Soc. 6: 194. 1891. Type: India: Kanara; 1890; *Talbot s.n.*

*Manisuris clarkei* (Hack.) Bor, Rec. Bot. Surv. India 16: 357. 1953

**Distribution.** Bihar, Gujarat, Karnataka, Madhya Pradesh, Maharashtra, Odisha,Tamil Nadu, West Bengal.

**Habit. **Annual.

**1148. **Mnesithea glandulosa** (Trin.) de Koning & Sosef, Blumea 31(2): 290. 1986 **

*Rottboellia glandulosa* Trin., Mém. Acad. Imp. Sci. St.-Pétersbourg, Sér. 6, Sci. Math. 2(4): 250. 1832. Type: Java; *Trinius s.n. *(LE-TRIN-0113.01).

*Manisuris glandulosa* (Trin.) Kuntze, Revis. Gen. Pl. 2: 780. 1891

*Coelorachis glandulosa* (Trin.) Stapf ex Ridl., Fl. Malay. Penin. 5: 204. 1925

*Ophiuros muricatulus* Steud., Syn. Pl. Glumac. 1: 360. 1855. Type: Java: “In humidis Tjikoya”; *Zollinger s.n.*

**Distribution.** Andaman and Nicobar Islands [Myanmar].

**Habit.** Perennial.

**Remarks.** Record for Andaman and Nicobar Islands fide Naithani (1990).

**1149. **Mnesithea granularis** (L.) de Koning & Sosef, Blumea 31(2): 295. 1986 **

*Cenchrus granularis* L., Mant. Pl. Altera 575. 1771. Type: “Habitat in India orientali.” Lectotype: *Herb. Linn. No. 1217.12* (LINN). LT designated by Clayton & Renvoize in Polhill (ed.), Fl. Trop. E. Africa, Gramineae 3: 849. 1982.

*Hackelochloa granularis* (L.) Kuntze, Revis. Gen. Pl. 2: 776. 1891

*Manisuris granularis* (L.) Naezén, Nov. Gram. Gen. 37. 1779

*Rytilix granularis* (L.) Skeels, U.S.D.A. Bur. Pl. Industr. Bull. 282: 20. 1913

*Manisuris porifera* Hack., Oesterr. Bot. Z. 41: 48. 1891. Type: India: Sikkim, Darjeeling, alt. 3000 ft.; October 22, 1869; *C.B. Clarke 9752B* (W).

*Hackelochloa porifera* (Hack.) D. Rhind, Grass. Burma 77. 1945

**Distribution.** Andaman and Nicobar Islands, Andhra Pradesh, Bihar, Chhattisgarh, Goa, Gujarat, Jammu and Kashmir, Karnataka, Kerala, Madhya Pradesh, Maharashtra, Rajasthan, Sikkim, Tamil Nadu, Tripura, Uttar Pradesh, West Bengal [China, Myanmar, Sri Lanka].

**Habit.** Annual.

**1150. **Mnesithea khasiana** (Hack.) de Koning & Sosef, Blumea 31: 291. 1986 **

*Rottboellia striata* Nees ssp. *khasiana* Hack., Monogr. Phan. [A. DC. & C. DC.] 6: 302. 1889. Syntypes: India: Assam, Khasia; *J.D. Hooker & Thomson s.n.* Buleschwar; *O. Kuntze s.n.*

*Coelorachis khasiana* (Hack.) Stapf ex Bor, Indian Forest Rec., Bot. n.s. 1(3): 101. 1938

**Distribution.** Assam, Nagaland, Meghalaya, Sikkim [Myanmar].

**Habit.** Perennial.

**1151. **Mnesithea laevis** (Retz.) Kunth, Révis. Gramin. 1: 154. 1829 var. **laevis

*Rottboellia laevis* Retz., Observ. Bot. (Retzius) 3: 11. 1783 (as “laeuis”). Type: “Misit e Tranquebaria Cl. Koening.”

*Ophiuros laevis* (Retz.) Benth., J. Linn. Soc., Bot. 19: 69. 1881 (as “Ophiurus”)

*Thyridostachyum laeve* (Retz.) Nees, Nomencl. Bot., ed. 2 (Steudel) 2: 685. 1841

*Diperium cylindricum* Desv., Opusc. Sci. Phys. Nat. 76. t. 6. f. 3. 1831. Type: “Ex indiae orientali?”

*Rottboellia perforata* Roxb., Pl. Coromandel 2: 43. t. 182. 1798. Type: India: grows on pasture ground; *Roxburgh s.n.* (BM).

*Ophiuros perforatus* (Roxb.) Trin., Mém. Acad. Imp. Sci. St.-Pétersbourg, Sér. 6, Sci. Math. 2: 246. 1832 (as “Ophiurus”)

**Distribution.** Andhra Pradesh, Bihar, Chhattisgarh, Himachal Pradesh, Karnataka, Kerala, Madhya Pradesh, Maharashtra, Meghalaya, Odisha, Rajasthan, Tamil Nadu, Uttar Pradesh, Uttarakhand, West Bengal [Afghanistan, China, Myanmar, Sri Lanka].

**Habit.** Perennial.

**1152. **Mnesithea laevis** (Retz.) Kunth var. **cochinchinensis** (Lour.) de Koning & Sosef, Blumea 31(2): 286. 1986 **

*Phleum cochinchinense *Lour., Fl. Cochinch. 1: 48. 1790. Type: “Habitat spontaneum in Cochinchina.” Vietnam; *Loureiro s.n.* (BM).

*Heteropholis cochinchinensis* (Lour.) Clayton, Kew Bull. 35(4): 816. 1981

*Thaumastochloa cochinchinensis* (Lour.) C.E. Hubb., Hooker’s Icon. Pl. 34: t. 3313- 3314. 1936

*Ophiuros monostachyus* J. Presl in C.B. Presl, Reliq. Haenk. 1(4–5): 330. 1830 (as “Ophiurus”). Type: “Habitat in Luzonia.”

**Distribution.** Andhra Pradesh, Bihar (Naithani), Himachal Pradesh, Karnataka, Kerala, Madhya Pradesh, Maharashtra, Rajasthan, Tamil Nadu, Uttar Pradesh [also in Indonesia].

**Habit. **Perennial.

**1153. **Mnesithea striata** (Nees) de Koning & Sosef, Blumea 31(2): 292. 1986 var. **striata

*Rottboellia striata* Nees, Syn. Pl. Glumac. (Steudel) 1: 361. 1854. Lectotye: Burma: Tavoy; October, 1827; *Gomez* (Numer. List [Wallich] no. 8877-C). LT designated by de Koning & Sosef in Blumea 31(2): 292. 1986.

*Coelorachis striata* (Nees) A. Camus, Ann. Soc. Linn. Lyon ser. 2, 68: 197. 1921

**Distribution.** Meghalaya, Manipur, Sikkim [Bangladesh, China, Myanmar, Nepal].

**Habit.** Perennial.

**1154. **Mnesithea striata** (Nees) de Koning & Sosef var. **pubescens** (Hack.) S.M. Phillips & S.L. Chen, Novon 15 (3): 470. 2005. **

*Rottboellia striata* Nees var. *pubescens* Hack., Monogr. Phan. [A. DC. & C. DC.] 6: 302. 1889. Type: India: Khasia Mountains; *J.D. Hooker & Thomson s.n.*

*Coelorachis striata* (Nees) A. Camus var. *pubescens* (Hack.) Henrard, Blumea 4(3): 519. 1941

**Distribution.** Assam, Meghalaya, Sikkim [Bangladesh].

**Habit.** Perennial.

**1155. **Mnesithea veldkampii** Potdar, S.P. Gaikwad, Salunkhe & S.R. Yadav, Kew Bull. 59(4): 629–631. f. 1. 2005 **

**Type. **India: Maharashtra, Satara District, Mawashi Plateau; November 6, 2002; *Yadav 1466* (CAL).

**Distribution.** Maharashtra.

**Habit.** Perennial.

***Ophiuros* C.F. Gaertn., Suppl. Carp. 3. 1805**

**Lectotype. ***Ophiuros corymbosus *C. F. Gaertn. (“corymbosa”), nom. illeg. (*Aegilops exaltata *L., *Ophiuros exaltatus *(L.) Kuntze) Probably designated by Clayton cf., Revis. Handb. Fl. Ceylon [Dassanayake] 2: 321. 1994.

**1156. **Ophiuros bombaiensis** Bor, Kew Bull. 1951: 167–168. 1951 **

**Type.** India: Madras, Talyuppa Mysore, 2000–3200 ft.; October, 1998; *A. Meebold 10764* (K).

**Distribution.** Karnataka, Maharashtra, Tamil Nadu.

**Habit.** Annual.

**1157. **Ophiuros exaltatus** (L.) Kuntze, Revis. Gen. Pl. 2: 780. 1891 **

*Aegilops exaltata* L., Mant. Pl. Altera 575. 1771. Type: “Habitat in Malabaria ad fossas agrorum.” Neotype: *Herb. Linn. No. 1218.15* (LINN). LT designated by Simon in Taxon 31: 564. 1982.

*Ophiuros corymbosus *C.F. Gaertn., Suppl. Carp. 4. 1805 (“corymbosa”), nom. illeg. *Rottboellia corymbosa *L. f., nom. illeg. superfl. for *Aegilops exaltata *L.

**Distribution.** Andhra Pradesh, Assam, Bihar, Chhattisgarh, Daman and Diu, Goa, Gujarat, Karnataka, Madhya Pradesh, Maharashtra, Rajasthan, Tamil Nadu, Uttar Pradesh, Uttarakhand [China, Myanmar, Sri Lanka].

**Habit.** Perennial.

**1158. **Ophiuros megaphyllus** Stapf ex Haines, Bot. Bihar Orissa 5: 1058. 1924 **

*Ophiuros corymbosus* sensu Hook. f., Fl. Brit. India (J.D. Hooker). 7(21): 160. 1896, p.p., non (L. f.) C. F. Gaertn., 1805

**Type.** India: in marshy places Tarai and Duars, probably in Purneah; *Haines s.n.*

**Distribution.** Assam, Meghalaya, Nagaland, West Bengal [Myanmar].

**Habit.** Perennial.

**Remarks.** Records for Meghalaya and Nagaland fide Naithani.

***Parahyparrhenia* A. Camus, Bull. Mus. Natl. Hist. Nat. Ser. 2, 22: 404. 1950**

**Type. ***Parahyparrhenia jaergeriana* A. Camus.

**1159. **Parahyparrhenia bellariensis** (Hack.) Clayton, Kew Bull. 27(3): 448. 1972 **

*Andropogon bellariensis* Hack., Flora 68: 123. 1885. Type: India: Anantapur District, on Gooty Fort Hill, Bellari; *Wight 2321* (K).

*Heteropogon bellariensis* (Hack.) C.E.C. Fisch., Fl. Madras 3(10): 1744. 1934

**Distribution.** Andhra Pradesh, Karnataka.

**Habit.** Perennial.

***Phacelurus* Griseb. Spic. Fl. Rumel. 2(5/6): 423. 1846**

**Type. ***Phacelurus digitatus* (Sm.) Griseb. (*Rottboellia digitata* Sm.).

**1160. **Phacelurus speciosus** (Steud.) C.E. Hubb., Bull. Misc. Inform. Kew 1928: 35. 1928 **

*Andropogon speciosus* Steud., Syn. Pl. Glumac. 1: 375. 1854. Type: Nepal; *Royle Herb. no. 263* (B).

*Pseudophacelurus speciosus* (Steud.) A. Camus, Bull. Mus. Natl. Hist. Nat. 27: 370. 1921

*Rottboellia speciosa* (Steud.) Hack., Monogr. Phan. [A. DC. & C. DC.] 6: 282. 1889

*Vossia speciosa* (Steud.) Benth., J. Linn. Soc., Bot. 19: 70. 1881 [combination is not made here]

*Andropogon corollatus* Steud., Syn. Pl. Glumac. 1: 369. 1854. Type: Nepal; *Royle Herb. no. 264*

*Ischaemum corollatum* (Steud.) Will. Watson, Bot. Himalayan Distr. N. W. Prov. 392. 1882

*Ischaemum robustum* Hook. f., Fl. Brit. India (J.D. Hooker). 7(21): 139. 1896. Type: Northwest Himalaya: Pauni, Bursahi, alt. 7000 ft.; *Brandeis s.n.*

*Ischaemum speciosum* Nees ex Steud., Syn. Pl. Glumac. 1: 375. 1854, nom. inval., as “A. Speciosus Nees (mpt. sub. Ischaemum).”

**Distribution.** Uttar Pradesh, Uttarakhand [Afghanistan, Nepal, Pakistan].

**Habit.** Perennial.

**1161. **Phacelurus zea** (C.B. Clarke) Clayton, Kew Bull. 33(2): 177. 1978 **

*Rottboellia zea* C.B. Clarke, J. Linn. Soc., Bot. 25: 86–87. t. 35. 1889. Type: India: Muneypore; *C.B. Clarke 41980* (K).

*Thyrsia zea* (C.B. Clarke) Stapf, Hooker’s Icon. Pl. 31: sub t. 3078. 1922

**Distribution.** Manipur, West Bengal [Bhutan, China, Myanmar, Nepal].

**Habit.** Perennial.

***Pogonachne* Bor, Kew Bull. 1949: 176. 1949**

**Type. ***Pogonachne racemosa* Bor.

**1162. **Pogonachne racemosa** Bor, Kew Bull. 1949: 176. 1949 **

**Type.** India: Matheran, Western Ghats; October 25, 1896; *Woodrow s.n.* (K).

**Distribution.** Bihar, Maharashtra.

**Habit.** Annual.

***Pogonatherum* P. Beauv., Ess. Agrostogr. 56, 176. 1812**

**Type. ***Pogonatherum saccharoideum* P. Beauv., nom. illeg. (*Saccharum paniceum* Lam., *Pogonatherum paniceum* (Lam.) Hack.).

**1163. **Pogonatherum crinitum** (Thunb.) Kunth, Enum. Pl. (Kunth) 1: 478. 1833 **

*Andropogon crinitus* Thunb., Fl. Jap. (Thunberg) 40. t. 7. 1784 (as “crinitum”). Type: Japan

*Homoplitis crinita* (Thunb.) Trin., Fund. Agrost. (Trinius) 166. 1820

*Ischaemum crinitum* (Thunb.) Trin., Mém. Acad. Imp. Sci. St.-Pétersbourg, Sér. 6, Sci. Math. 2: 298. 1832

*Andropogon monandrus* Roxb., Fl. Ind. (Carey and Wallich ed.) 1: 264. 1820. Type: India: “A native of Mountainous countries.”

*Pollinia monandra* Spreng., Syst. Veg. (ed. 16) [Sprengel] 1: 288. 1824 (“1825”). Type: India orientalis

**Distribution.** Andhra Pradesh, Arunachal Pradesh, Assam, Bihar, Chhattisgarh, Karnataka, Kerala, Madhya Pradesh, Maharashtra, Meghalaya, Mizoram, Nagaland, Odisha, Sikkim, Tamil Nadu, Tripura, Uttar Pradesh, Uttarakhand, West Bengal [Afghanistan, Bhutan, China, Myanmar, Nepal, Pakistan, Sri Lanka].

**Habit. **Perennial.

**1164. **Pogonatherum paniceum** (Lam.) Hack., Allg. Bot. Z. Syst. 12: 178. 1906 **

*Saccharum paniceum* Lam., Encycl. (Lamarck) 1: 595. 40. f. 1. 1785. Type: “Cette plante croit dans les Indes orientales, & nous a été communiqué par M. Sonnerat.” East Indies; *Sonerat s.n.*(P).

*Pogonatherum santapaui* Sur, J. Bombay Nat. Hist. Soc. 73(1): 190. 1976. Type: India: Uttar Pradesh, Garhwal, alt. 2300 ft.; February 26, 1960; *J.N. Vohra 11248*

*Pogonatherum polystachyum* Roem. & Schult., Syst. Veg., ed. 15 bis [Roemer & Schultes] 2: 497. 1817, nom. illeg. & superfl. for *Saccharum paniceum*

*Pollinia polystachys* Spreng., Syst. Veg. (ed. 16) [Sprengel] 1: 288. 1825 nom. illeg. & superfl. for *Saccharum paniceum*

*Perotis polystachya* Willd., Sp. Pl., ed. 4 [Willdenow] 1: 324. 1797, nom. illeg. & superfl. for *S*. *paniceum*

*Pogonatherum saccharoideum* P. Beauv., Ess. Agrostogr. 176, 177. 1812, nom. illeg. & superfl. for *Saccharum paniceum*

**Distribution. **Andaman and Nicobar Islands, Andhra Pradesh, Arunachal Pradesh, Assam, Bihar, Himachal Pradesh, Jammu and Kashmir, Karnataka, Kerala, Madhya Pradesh, Maharashtra, Manipur, Meghalaya, Nagaland, Odisha, Punjab, Sikkim, Tamil Nadu, Uttar Pradesh, West Bengal [Afghanistan, Bhutan, China, Myanmar, Nepal, Pakistan, Sri Lanka].

**Habit.** Perennial.

**1165. **Pogonatherum rufobarbatum** Griff., Not. Pl. Asiat. 3: 81. 1851 **

**Type.** India: Assam, in aquois, Moosmai; October 18, 1835; *It. Ass. 159.*

**Distribution.** Andhra Pradesh, Assam, Arunachal Pradesh, Bihar, Chhattisgarh, Himachal Pradesh, Karnataka, Kerala, Madhya Pradesh, Meghalaya, Odisha, Tamil Nadu, Uttarakhand, West Bengal.

**Habit.** Perennial.

***Polytrias* Hack. (1887), in Engler & Prantl, Nat. Pflanzenfam. 2(2): 24. 1887**

**Type. ***Polytrias praemorsa* (Nees) Hack. (*Pollinia praemorsa* Nees).

**1166. **Polytrias indica** (Houtt.) Veldkamp, Blumea 36(1): 180–181. 1991. **

*Phleum indicum* Houtt., Nat. Hist. 2(13): Aanwyz. Plaat. [1], 198, pl. 90, f. 2. 1782. (13 May 1782). Type: Koenig s.n., Indonesia, Java

*Ischaemum indicum* (Houtt.) Merr., J. Arnold Arbor. 19(4): 320. 1938 var. *indicum*

*Andropogon diversiflorus* Steud., Syn. Pl. Glumac. 1(4–5): 370. 1854. Type: Indonesia: Java; “Andropogon ? no. *539*, p. 99, Zoll. Moritz.”

*Eulalia amaura* Ohwi, Bull. Tokyo Sci. Mus. 18: 2. 1947, nom. superfl. & illeg. for *Andropogon diversiflorus*

*Polytrias amaura* Kuntze Revis. Gen. Pl. 2: 788. 1891, nom. superfl. & illeg. for *Andropogon diversiflorus*

*Andropogon amaurus* Buse, Pl. Jungh. 360. 1854, nom. superfl. & illeg. for *Andropogondiversiflorus*. Type: Indonesia: Java; *Zollinger 539 Herb. Acad. L. Bata.*

**Distribution.** West Bengal [also in Indonesia].

**Habit.** Annual.

**Remarks.** Record for West Bengal fide Naithani (1990).

***Pseudanthistiria* (Hack.) Hook. f., Fl. Brit. India (J.D. Hooker). 7 (pt. 21): 219. 1896**

*Andropogon* sect. *Pseudanthistiria* Hack., Monogr. Phan. [A. DC. & C. DC.] 6: 400. 1889.

**Lectotype. ***Pseudanthistiria heteroclita* (Roxb.) Hook. f. (*Anthistiria heteroclita* Roxb.). LT designated by B.P. Uniyal in Taxon 33: 501. 1984.

**1167. **Pseudanthistiria burmanica** Hook. f., Fl. Brit. India (J.D. Hooker). 7(21): 220. 1896 **

**Type.** Burma: Pegu; *Kurz s.n.*

**Distribution.** Kerala [Myanmar].

**Habit.** Annual.

**1168. **Pseudanthistiria heteroclita** (Roxb.) Hook. f., Fl. Brit. India (J.D. Hooker). 7(21): 219. 1896 **

*Anthistiria heteroclita* Roxb., Fl. Ind. (Carey and Wallich ed.) 1: 253–254. 1820. Type: India: “A native of newly made pasture land in the vicinity of Calcutta.”

*Andropogon heteroclitus* (Roxb.) Nees, Fl. Afr. Austral. Ill. 115. 1841

*Pseudanthistiria hispida* Hook. f., Fl. Brit. India (J.D. Hooker). 7(21): 219. 1896. Syntypes: India: the Concan; *J.E. Stocks s.n., J.S. Law s.n.*, *Dalzell s.n.* Central province, Khandwa; *Duthie s.n.*

*Pseudanthistiria intermedia* Birari & D’cruz, J. Bombay Nat. Hist. Soc. 73: 192. f. 1–5. 1976. Type: India: Maharashtra, Khandesh, Satpura Range; November 17, 1968; *S.R. Birari I-681BC* (AHMA).

**Distribution.** Andhra Pradesh, Bihar, Daman & Diu, Goa, Gujarat, Karnataka, Kerala, Madhya Pradesh, Maharashtra, Tamil Nadu, West Bengal.

**Habit.** Annual.

**1169. **Pseudanthistiria umbellata** (Hack.) Hook. f., Fl. Brit. India (J.D. Hooker). 7(21): 220. 1896 **

*Andropogon umbellatus* Hack., Monogr. Phan. [A. DC. & C. DC.] 6: 401. 1889. Type: Sri Lanka; *Thwaites 963* (W).

**Distribution.** Andhra Pradesh, Karnataka, Kerala, Maharashtra, Odisha, Tamil Nadu [Sri Lanka].

**Habit.** Annual.

***Pseudodichanthium* Bor, Indian Forester 66: 271. 1940**

**Type. ***Pseudodichanthium serrafalcoides* (Cooke & Stapf) Bor (*Andropogon serrafalcoides* Cooke & Stapf).

**1170. **Pseudodichanthium serrafalcoides** (Cooke & Stapf) Bor, Indian Forester 66: 272. 1940 **

*Andropogon serrafalcoides* Cooke & Stapf, Bull. Misc. Inform. Kew 1908: 450. 1908. Type: India: Western Ghats, Sakarpattar, near Lanauli; *Woodrow s.n.*

*Dichanthium serrafalcoides* (Cooke & Stapf) Blatt. & McCann., J. Bombay Nat. Hist. Soc. 32: 426. 1928

*Andropogon cookei* Stapf ex Woodrow, J. Bombay Nat. Hist. Soc. 13: 438. 1901, nom. nud.

**Distribution.** Maharashtra.

**Habit.** Annual.

Pseudosorghum **A. Camus, Bull. Mus. Natl. Hist. Nat. 26(7): 662. 1920–1921 **

**Lectotype. ***Pseudosorghum fasciculare *(Roxb.) A. Camus (*Andropogon fascicularis *Roxb.).

**1171. **Pseudosorghum fasciculare** (Roxb.) A. Camus, Bull. Mus. Natl. Hist. Nat. 26(7): 662. 1920–1921 **

*Andropogon fascicularis* Roxb., Fl. Ind. (Carey and Wallich ed.) 1: 269. 1820. Type: India: “A native of Mountains.”

*Sorghum fasciculare* (Roxb.) Haines, Bot. Bihar Orissa 5: 1034. 1924

*Andropogon gangeticus* Hack. ex Duthie, Fodder Grasses of North. India 34. 1988

*Sorghum gangeticum* (Hack. ex Duthie) Kunze, Revis. Gen. Pl. 2: 791. 1891

*Andropogon nitidulus* Hook. f., Fl. Brit. India (J.D. Hooker). 7(21): 199. 1896. Type: NorthWest Himalaya: on the Sewalik Hills; and Chanda in the Central Provinces; *Duthie s.n.*

*Andropogon tonkinensis* Balansa, J. Bot. (Morot) 4(6): 112. 1890. Type: Vietnam: Tonkin, Phuong-Lam, Sur les Collines incultes; January 13, 1887; *Balansa 1770* (L).

**Distribution.** Andhra Pradesh, Assam, Bihar, Chhattisgarh, Karnataka, Kerala, Madhya Pradesh, Nagaland, Odisha, Tamil Nadu, Uttar Pradesh, West Bengal [China, Myanmar, Nepal].

**Habit.** Annual.

***Rottboellia* L. f., Suppl. Pl. 13 (Rottböllia), 114 (“Rottbölla”). 1782 (“1781”), nom. cons.**

**Type. ***Rottboellia exaltata* L. f., nom. illeg. (non (L.) Naezen, 1779) (typ. cons*.*).

**1172. **Rottboellia cochinchinensis** (Lour.) Clayton, Kew Bull. 35(4): 817. 1981 **

*Stegosia cochinchinensis* Lour., Fl. Cochinch. 1: 51. 1790. Type: Vietnam: “Nullibi eam vidi praeterquam in Cochinchina.”

*Rottboellia exaltata* L.f., Suppl. Pl. 114. 1782 (“1781”), non (L.) Naezen, 1779. Type: “Habitat in indiis”; *Thunberg s.n.* (LINN).

*Rottboellia exaltata* L.f. var. *robusta* Hook. f., Fl. Brit. India (J.D. Hooker). 7(21): 156. 1896. Syntypes: India: Malabar, Palamocotta; *Wight 3286*, Numer. List [Wallich] no. 8875E. Poone; *Woodrow s.n.*

**Distribution.** Andaman and Nicobar Islands, Andhra Pradesh, Assam, Bihar, Gujarat, Himachal Pradesh, Jammu and Kashmir, Karnataka, Kerala, Madhya Pradesh, Maharashtra, Meghalaya, Odisha, Rajasthan, Sikkim, Tamil Nadu, Tripura, Uttar Pradesh, West Bengal [China, Pakistan].

**Habit.** Annual.

**1173. **Rottboellia goalparensis** Bor, Indian Forest Rec., Bot. n. s. 1(3): 100. 1938 **

*Robynsiochloa goalparensis* (Bor) Clayton, Kew Bull. 35(4): 817. 1981

**Type.** India: Assam, Goalpara District, Kochugaon; *Das 10471* (K).

**Distribution.** Assam.

**Habit.** Annual.

***Saccharum* L., Sp. Pl. 1: 54. 1753**

**Lectotype. ***Saccharum officinarum* L. (“officinaram”) LT designated by Nash, N. Amer. Fl. 17: 89. 30 Jun 1909; affirmed by Hitchcock, Bull. U.S.D.A. 772: 255. 1920.

**1174. **Saccharum fallax** Balansa, J. Bot. (Morot) 4(4): 80. 1890 **

*Narenga fallax* (Balansa) Bor, Kew Bull. 1948: 162. 1948

*Erianthus chrysothrix* Hack., Oesterr. Bot. Z. 41: 6. 1891. Syntypes: India: Naga Hills ad Piffima, alt. 2000 ft.; *C.B. Clarke 40913*. Khasia Mountains. ad Nungklas; *C.B. Clarke 40209*. India: Shillong, alt. 4000 ft.; *C.B. Clarke 44484*. India; *C.B. Clarke 44484*

**Type.** Vietnam: Hills and Valleys of Tu-Phap; *Balansa s.n.*

**Distribution.** Assam, Bihar, Manipur, Meghalaya, Nagaland, Tripura [China, Myanmar].

**Habit.** Perennial.

**1175. **Saccharum filifolium** Steud., Syn. Pl. Glumac. 1: 409. 1854 **

*Erianthus filifolius* (Steud.) Nees ex Hack., Monogr. Phan. [A. DC. & C. DC.] 6: 146. 1889

*Erianthus macratherus* Pilg., Notizbl. Bot. Gart. Berlin-Dahlem 14: 347. 1939. Type: Sikkim, Nanga-Parbat, Gebiet, Indus-Tal, Drang, Jalipur, 4000 ft.; May 22, 1937; *C. Troll 7170*

*Saccharum macratherum* (Pilg.) Ahmad & Stewart, Grasses of West Pakistan 1: 79. 1958 (as “macrantherus”)

**Type.** Nepal.

**Distribution.** Sikkim [Afghanisthan, Nepal, Pakistan].

**Habit.** Perennial.

**1176. **Saccharum griffithii** Munro ex Boiss., Fl. Orient. (Boisser) 5: 453. 1884 **

*Erianthus griffithii* (Munro ex Boiss.) Hook. f., Fl. Brit. India (J.D. Hooker). 7(21): 122. 1896

*Saccharum ciliare* Andersson var. *griffithii* (Munro ex Boiss.) Hack., Monogr. Phan. [A. DC. & C. DC.] 6: 119. 1889

**Type.** Afghanistan: Kuram District, on the arid shingle plains and borders of fields, up to 6000 ft. “Hab. ad Schah Bilawul Afghaniae”; *Griffith exs. 507.*

**Distribution.** Punjab [Afghanistan, Pakistan].

**Habit. **Perennial.

**1177. **Saccharum longisetosum** (T. Anderson) V. Naray., Fl. Assam 5: 461. 1940 var. **longisetosum

*Erianthus longisetosus* T. Anderson, Ofvers. Forh. Kongl. Svenska Vetensk.-Akad. 163. 1855. Syntypes: India: Sillet. India; *F. de Silva s.n.* India; Numer. List [Wallich] no. 8846

**Distribution.** Assam, Meghalaya, West Bengal [Bangladesh, Bhutan, China, Myanmar].

**Habit.** Perennial.

**1178. **Saccharum longisetosum** (T. Anderson) V. Naray. var. **hookeri** (Hack.) Bor, Grasses Burma, Ceylon, India & Pakistan 212. 1960 **

*Erianthus hookeri* Hack., Monogr. Phan. [A. DC. & C. DC.] 6: 142. 1889. Type: India: Sikkim; *J.D. Hooker Erianthus no. 15*

*Saccharum hookeri* (Hack.) V. Naray., Fl. Assam 5: 461. 1940

*Erianthus longisetosus* T. Anderson var. *hookeri *(Hack.) Bor, Grasses Burma, Ceylon, India and Pakistan 151. 1960

**Distribution.** Assam, Manipur, Meghalaya, Nagaland, Sikkim, Uttar Pradesh, West Bengal [Bhutan].

**Habit.** Perennial.

**1179. **Saccharum narenga** (Nees) Wall. ex Hack., Monogr. Phan. [A. DC. & C. DC.] 6: 119. 1889 **

*Eriochrysis narenga* Nees, Syn. Pl. Glumac. (Steudel) 1: 411. 1854. Type: India; Numer. List [Wallich] no. 8856 B (K).

*Eriochrysis porphyrocoma* Hance, J. Bot. 14(166): 294. 1876. Type: China: Guangdong, “secus amnem Lien chau”; October, 1875; *Galbraith 19285*

*Narenga porphyrocoma* (Hance) Bor, Indian Forester 66(5): 268. 1940

**Distribution.** Assam, Bihar, Chhattisgarh, Jharkhand, Madhya Pradesh, Manipur, Meghalaya, Tamil Nadu, Uttar Pradesh, West Bengal [Bangladesh, China, Myanmar, Nepal, Pakistan].

**Habit.** Perennial.

**1180. **Saccharum officinarum** L., Sp. Pl. 1: 54. 1753 **

**Lectotype.** “Arundo saccharifera C.B.” in Sloane, Voy. Jamaica, 1: 108, t. 66, 1707. Typotype: Herb. Sloane 2: 14 (BM-SL). LT designated by Reveal et al. in Taxon 38: 96. 1989.

**Distribution.** Andhra Pradesh, Assam, Bihar, Delhi, Goa, Gujarat, Karnataka, Kerala, Madhya Pradesh, Maharashtra, Odisha, Rajasthan, Tamil Nadu, Tripura, Uttar Pradesh, West Bengal [Bangladesh, China, Pakistan, Sri Lanka]. Cultivated.

**Habit. **Perennial.

**1181. **Saccharum rufipilum** Steud., Syn. Pl. Glumac. 1: 409. 1854 **

*Erianthus rufipilus* (Steud.) Griseb., Nachr. Ges. Wiss. Gottingen, Math.-Phys. Kl. 93. 1868

*Erianthus pallens* Hack., Monogr. Phan. [A. DC. & C. DC.] 6: 145. 1889. Type: India: Himalayas; *J.D. Hooker & Thomson s.n.* (B).

*Saccharum versicolor* Steud., Syn. Pl. Glumac. 1: 409. 1854. Type: India

*Erianthus versicolor* (Steud.) Hack. var. *pallens* (Hack.) Hook. f., Fl. Brit. India (J.D. Hooker). 7(21): 124. 1896

*Erianthus fulvus* Nees ex Hack., Monogr. Phan. [A. DC. & C. DC.] 6: 147. 1889, non (R. Br.) Kunth, 1829

**Type.** Nepal; Numer. List [Wallich] no. 8849.

**Distribution.** Bihar, Jammu and Kashmir, Meghalaya, Sikkim, Uttarakhand [Bhutan, China, Myanmar, Nepal, Pakistan].

**Habit.** Perennial.

**1182. **Saccharum sikkimense** (Hook. f.) V. Naray., Fl. Assam 5: 462. 1940 (as “sikkimensis”) **

*Erianthus sikkimensis* Hook. f., Fl. Brit. India (J.D. Hooker). 7(21): 123. 1896. Type: India: Sikkim Himalaya, Lachen Valley, alt. 600 ft.; *J.D. Hooker s.n.* (K).

**Distribution.** Sikkim [Bhutan].

**Habit.** Perennial.

**1183. **Saccharum sinense** Roxb., Pl. Coromandel 3: 26. t. 232. 1815 **

*Saccharum barberi* Jesweit, Arch. Suikerindustr. Ned. Ned.-Indie 12: 396. 1925. Type: India: Chunnee-riet.

**Type.** Introduced into Botanical Garden of Calcutta from China in 1796.

**Distribution. **West Bengal [Bangladesh, China]. Cultivated.

**Habit.** Perennial.

**1184. **Saccharum spontaneum** L., Mant. Pl. Altera 183. 1771 **

*Imperata spontanea* (L.) P. Beauv., Ess. Agrostogr. 8. 165. 1812

*Saccharum canaliculatum* Roxb., Fl. Ind. (Carey and Wallich ed.) 1: 251. 1820. Type: “A most beautiful stately species, a native of Bengal in moist thickets.”

*Saccharum insulare* Brongn., Voy. Monde, Phan. 2(2): 99. 1831. Type: “Sauvage dans les lieux frais près des ruisseaux de l’ile Oualan, l’une des iles Carolines” Caroline Islands: Kusaie (Kosrae, Oualan); 1825; *Lesson s.n.* (P).

*Saccharum propinquum* Steud., Syn. Pl. Glumac. 1: 406. 1854. Type: “Ex Hrbo. Prisiensi. Coromandel.”

*Saccharum semidecumbens* Roxb., Fl. Ind. (Carey and Wallich ed.) 1: 241. 1820. Type: “A native of Bengal, where it delights in low wet places.”

**Type.** “Habitat in Malabariae aquosis. Koenig.” Lectotype: Herb. Linn. No. 77.1 (LINN). LT designated by Cope in Nasir and Ali (ed.), Fl. Pakistan 143: 263. 1982.

**Distribution.** Andhra Pradesh, Assam, Bihar, Chhattisgarh, Delhi, Goa, Gujarat, Himachal Pradesh, Jharkhand, Jammu and Kashmir, Karnataka, Kerala, Madhya Pradesh, Maharashtra, Meghalaya, Nagaland, Odisha, Punjab, Rajasthan, Tamil Nadu, Tripura, Uttar Pradesh, West Bengal [Afghanistan, Bhutan, China, Myanmar, Pakistan, Sri Lanka].

**Habit.** Perennial.

**1185. **Saccharum wardii** (Bor) Bor ex Cope, Kew Bull. 35:702. 1980 **

*Erianthus wardii* Bor, Kew Bull. 1954: 498. 1954. Type: Burma: North Triangle, Kainjaw, alt. 3200 ft.; December 2, 1953; *F. Kingdon-Ward 21666 *(K).

**Distribution.** Arunachal Pradesh [Myanmar].

**Habit.** Perennial.

**Remarks.** Record for India fide Naithani.

**1186. **Saccharum williamsii** (Bor) Bor ex Cope, Kew Bull. 35:703. 1980 **

*Erianthus williamsii* Bor, Kew Bull. 12(3): 413. 1958. Type Nepal: NW of Gurjakhani, 9000 ft.; July 30, 1954; *Polunin, Sykes & Williams 3670* (BM).

**Distribution.** India [Nepal (Naithani)].

**Habit.** Perennial.

**Remarks.** Distribution in India not reported.

***Sarga* Ewart, Proc. Roy. Soc. Victoria 23(2): 296. 1911**

**Type.***Sarga stipoidea* Ewart & Jean White.

**1187. **Sarga leiocladus** (Hack.) Spangler, Austral. Syst. Bot. 16(3): 290. 2003 (as “leiocladum”) **

*Andropogon australis* Spreng. ssp. *leiocladus* Hack., Monogr. Phan. [A. DC. & C. DC.] 6: 524. 1889. Type: Australia: New South Wales, Central Coast, Port Jackson; February, 1825; *C. Gaudichaud s.n.* (P).

*Sorghum leiocladus* (Hack.) C.E. Hubb., Hooker’s Icon. Pl. 34: t. 3364. 1938 (as “leiocladum”)

**Distribution.** India [also in Australia]. Native to eastern Australia.

**Habit.** Perennial.

**Remarks.** Distribution in India not reported. It is possible that this report is erroneous.

***Schizachyrium* Nees, Fl. Bras. Enum. Pl. 331. 1829**

**Lectotype. ***Schizachyrium condensatum* (Kunth) Nees (*Andropogon condensatus* Kunth). LT designated by Pfeiffer, Nomencl. Bot. 2: 1077. 1874.

**1188. **Schizachyrium brevifolium** (Sw.) Nees ex Buse, Pl. Jungh. 359. 1854 **

*Andropogon brevifolius* Sw., Prodr. (Swartz) 26. 1788 (as “brevifolium”). Type: “Jamaica.” Lectotype: West Indies: Jamaica; *O.P. Swartz s.n.* (M). LT designated by Hitchcock in Contr. U.S. Natl. Herb. 12: 143. 1908.

*Pollinia brevifolia* (Sw.) Spreng., Pl. Min. Cogn. Pug. 2: 13. 1815

*Andropogon floridus* Trin., Mém. Acad. Imp. Sci. Saint-Pétersbourg, Sér. 6, Sci. Math., Seconde Pt. Sci. Nat. 2: 265. 1832

*Andropogon obtusifolius* Poir., Encycl. (Lamarck) Suppl. 1: 583. 1810. Type: “Cette plante a été recueillie par M. Lendru a Porto Ricco.” Puerto Rico; *M. Lendru s.n. *(P).

*Andropogon parviflorus* Roxb., Fl. Ind. (Carey and Wallich ed.) 1: 277. 1820. Type: “A very beautiful most delicate species, a native of pasture land up amongst the Circar Mountains and also of the Mountains themselves.”

*Pollinia vaginata* Spreng., Pl. Min. Cogn. Pug. 2: 11. 1815. Type: “Habitat in Bengalia.”

*Andropogon debilis* Kunth, Enum. Pl. [Kunth] 1: 488. 1833. Type: Mexico.

*Andropogon tenellus* J. Presl in C. Presl., Reliq. Haenk. 1(4–5): 335. 1830, nom. later hom. non Roxb., 1820. Type: “Hab. in Mexico”; *Haenke s.n.* (PR).

**Distribution.** Andhra Pradesh, Assam, Bihar, Chhattisgarh, Jharkhand, Karnataka, Kerala, Madhya Pradesh, Maharashtra, Manipur, Meghalaya, Nagaland, Odisha, Rajasthan, Tamil Nadu, Tripura, Uttar Pradesh, Uttarakhand, West Bengal [Bangladesh, Bhutan, China, Myanmar, Nepal].

**Habit.** Annual.

**1189. **Schizachyrium delavayi** (Hack.) Bor, Indian Forest Rec., Bot. new ser. 1(3): 95. 1938 **

*Andropogon delavayi* Hack., Monogr. Phan. [A. DC. & C. DC.] 6: 404–405. 1889. Syntypes: China: Yunnan, Kimi-se, ad pedes Hee-schan-man prope Koken; *J.M. Delavay 1800*; China: Yunnan, in pratis ad Ki-pin-Kay prope Tali; *J.M. Delavay 2242* (P).

*Eremopogon delavayi* (Hack.) A. Camus, Ann. Soc. Linn. Lyon sér. 2. 68: 208. 1921

*Andropogon bootanensis* Hook. f., Fl. Brit. India (J.D. Hooker). 7(21): 166. 1896. Type: Bhutan Himalaya: Tassangsee, alt., 5000–6000 ft.; *Griffith s.n.*

*Schizachyrium bootanense* (Hook. f.) A. Camus, Ann. Soc. Linn. Lyon sér. 2. 70: 90. 1923

**Distribution.** Assam, Manipur, Nagaland, Sikkim [Bhutan, China, Myanmar, Nepal].

**Habit.** Perennial.

**1190. **Schizachyrium exile** (Hochst.) Pilg., Bot. Jahrb. Syst. 54: 284. 1917 **

*Andropogon exilis* Hochst., Flora 27: 241. 1844. Syntypes: Sudan: “Ad latus, orientale montis Cordofani Arasch-Cool locis humidis habitat”; *Kotschyi 370* (K). Ethiopia: ad latus orientale montis Cordofani Arasch-Cool locis humidis habitat; 1837–1838; *Kotschyi 19* (L).

**Distribution.** Andhra Pradesh, Bihar, Chhattisgarh, Jharkhand, Karnataka, Kerala, Madhya Pradesh, Maharashtra, Meghalaya, Odisha, Rajasthan, Tamil Nadu, Uttar Pradesh, West Bengal [China].

**Habit.** Annual.

**Remarks.** Records for Karnataka, Madhya Pradesh and Tamil Nadu fide Naithani.

**1191. **Schizachyrium impressum** (Hack.) A. Camus, Ann. Soc. Linn. Lyon sér. 2, 70: 91. 1923 **

*Andropogon impressus* Hack., Oesterr. Bot. Z. 41: 49. 1891. Type: India: Kashmir, Kishtwar, 4000 ft.; *C.B. Clarke 31433* (W).

**Distribution.** Jammu and Kashmir [Pakistan].

**Habit.** Perennial.

**1192. **Schizachyrium sanguineum** (Retz.) Alston in Trimen, Handb. Fl. Ceylon 6(Suppl.): 334. 1931 **

*Rottboellia sanguinea* Retz., Observ. Bot. (Retzius) 3: 25. 1783. Type: “Ramulum misit Cl. Bladh e China.” China; *Bladh s.n. *(LD).

*Andropogon sanguineus* (Retz.) Merr., Philipp. J. Sci., C 12: 101. 1917

*Thelepogon sanguineus* (Retz.) Spreng., Syst. Veg. (ed. 16) [Sprengel] 1: 299. 1824 (“1825”)

*Schizachyrium semiberbe* Nees, Fl. Bras. Enum. Pl. 2(1): 336. 1829. Type: “Habitat in Brasilia australiori”; *Sellow s.n.* (B).

*Andropogon semiberbis* (Nees) Kunth, Enum. Pl. [Kunth] 1: 489. 1833

*Andropogon semiberbis* (Nees) Kunth var. *leptostachyus* (Benth.) Hack., Monogr. Phan. [A. DC. & C. DC.] 6: 370. 1889

*Andropogon leptostachyus* Benth., Niger Fl. [W. J. Hooker]. 571. 1849. Type: “On the Quarra; Vogel.” Nigeria: River Niger [Quorra]; *Vogel s.n.* (K)

*Andropogon pseudograya* Steud., Syn. Pl. Glumac. 1: 365. 1854. Type: Sri Lanka; *Arnott s.n.*

*Schizachyrium hirtiflorum* Nees, Fl. Bras. Enum. Pl. 2(1): 334. 1829. Type: “Habitat in Brasilia australiori.” Brazil; *Sellow s.n. *(B).

*Andropogon hirtiflorus* (Nees) Kunth, Révis. Gramin. 2: 571. 1832

**Distribution.** Meghalaya [China, Myanmar, Sri Lanka].

**Habit.** Perennial.

**1193. **Schizachyrium sudhanshui** N.P. Singh, J. Indian Bot. Soc. 60(3–4): 359. 1981 **

**Type.** “lecti ad Raichur-Hyderabad via 19th km ad alt. C. 400 m in dist. Raichur, ditionae Karnataka dei 15th Novembris anni 1975.” Holotype: *Singh 141725A* (CAL); isotypes: *Singh 141725B-D* (BSI).

**Distribution.** Karnataka.

**Habit.** Annual.

**1194. **Schizachyrium villosum** (Poir.) Veldkamp, Blumea 31: 306. 1986 **

*Rottboellia villosa* Poir., Encycl. (Lamarck) 6: 313. 1804 (as “Rottbolla”). Type: “Cette plante a été recueillie dans les Indes par Commerson (V.S. in Herb. Juss.).” *Commerson s.n.* (P.-JU).

**Distribution.** India.

**Habit.** Perennial.

**Remarks.** Distribution in India not reported.

***Sehima* Forssk., Fl. Aegypt.-Arab. 178. 1775**

**Type. ***Sehima ischaemiides* Forssk.

**1195. **Sehima ischaemoides** Forssk., Fl. Aegypt.-Arab. 178. 1775 **

*Andropogon lineatus* Steud., Syn. Pl. Glumac. 1: 369. 1854

*Andropogon rhyncophorus* Stapf, Mém. Soc. Bot. France. 8: 101. 1908. Type: Central African Republic: Dar-el-Hadjer; *Chevalier 9797 *(K).

*Sehima inscalptum* Hochst., Flora 27: 247. 1844. Type: “Ad agrorum margines locis siccioribus prope Gapdiam in Abyssiniae provincia Tigre lectum est. Septembri floriferum.” Ethiopia; September 24, 1839; *Schimper 739* (K, MO).

*Andropogon inscalptus* (Hochst.) Andersson, Beitr. Fl. Aethiop. 310. 1867

*Ischaemum inscalptum* (Hochst.) A. Rich., Tent. Fl. Abyss. 2: 472. 1850–1851.

*Sehima kotschyi* Hochst., Flora 27: 247. 1844. Type: “Ad radices montis Cordofani Arasch-Cool in fissuris rupium graniticarum”; Sudan: Kordofan, Arashkol; *Kotschy 373*

*Andropogon sehima* Steud., Syn. Pl. Glumac. 1: 369. 1854, nom. superfl. & illeg. for *Sehimaischaemoides*

**Type.** “Yeman in montibus ad Hadle.” Yemen: Al Hadiyah, in montibus ad Hadie; 1763; *Forsska s.n.* (C).

**Distribution.** Gujarat, Karnataka, Maharashtra, Tamil Nadu [Pakistan].

**Habit.** Annual.

**1196. **Sehima nervosa** (Rottler) Stapf, Fl. Trop. Afr. [Oliver et al.] 9(1): 36. 1917 (as “nervosum”) **

*Andropogon nervosus* Rottler, Ges. Naturf. Freunde Berlin Neue Schriften 4: 218. 1803. Type: India: Madras;* Rottler s.n. *(K).

*Hologamium nervosum* (Rottler) Nees, Edinburgh New Philos. J. 18: 185. 1835

*Ischaemum nervosum* (Rottler) Thwaites, Enum. Pl. Zeyl. (Thwaites). 365. 1864

*Andropogon striatus* Klein ex Willd., Sp. Pl., ed. 4 [Willdenow] 4(1): 903. 1806. Type: “Habitat in Malabaria.”

*Ischaemum laxum* R. Br., Prodr. Fl. Nov. Holland. 205. 1810. Type: Australia: “littora Novae Hollandiae intra tropicum.”

*Ischaemum macrostachyum* A. Rich., Tent. Fl. Abyss. 2: 472. 1850–1851. Type: “Crescit in montibus versus fluvium Tacazze, mense Augusto (Schimper)”

*Andropogon macrostachyus* (A. Rich.) Andersson, Beitr. Fl. Aethiop. 310. 1867

*Sehima macrostachyum* Hochst. ex Hack., Monogr. Phan. [A. DC. & C. DC.] 6: 245. 1889, invalid, pro syn.

*Andropogon tacazensis* Steud., Syn. Pl. Glumac. 1: 369. 1854, nom. superfl. & illeg.

**Distribution.** Andhra Pradesh, Bihar, Chhattisgarh, Deccan, Gujarat, Jharkhand, Karnataka, Kerala, Madhya Pradesh, Maharashtra, Manipur, Odisha, Rajasthan, Tamil Nadu [China, Myanmar, Pakistan, Sri Lanka].

**Habit.** Perennial.

**1197. **Sehima notata** (Hack.) A. Camus, Bull. Mus. Hist. Nat. (Paris) 27: 373. 1921 (as “notatum”) **

*Ischaemum notatum* Hack., Monogr. Phan. [A. DC. & C. DC.] 6: 246. 1889. Type: India: Kamaon District; 5100–7300 ft.; *J.F. Duthie 5057*. Prope Dunagiri supra Duarahat ad 6600 ft.

**Distribution.** Bihar, Himachal Pradesh, Maharashtra, Uttar Pradesh, Uttarakhand.

**Habit.** Perennial.

**1198. **Sehima sulcata** (Hack.) A. Camus, Bull. Mus. Hist. Nat. (Paris) 27: 373. 1921 (as “sulcatum”) **

*Ischaemum sulcatum* Hack., Monogr. Phan. [A. DC. & C. DC.] 6: 248. 1889. Type: “India orientalis, prope Narsingpur province, Centralis alt. 1000 ft.”; *O. Kuntze in h. prop. s.n. *Sholapur province, Bombay Stead in h. m.”

**Distribution.** Andhra Pradesh, Bihar, Gujarat, Karnataka, Madhya Pradesh, Maharashtra, Rajasthan, Tamil Nadu [Myanmar].

**Habit.** Perennial.

***Sorghum* Moench, Methodus (Moench) 207. 1794, nom. cons.**

**Type. ***Sorghum bicolor* (L.) Moench (*Holcus bicolor* L.).

**1199. **Sorghum arundinaceum** (Desv.) Stapf, Fl. Trop. Afr. [Oliver et al.] 9(1): 114. 1917 **

*Rhaphis arundinacea* Desv., Opusc. Sci. Phys. Nat. 69. 1831 (as “arundinaceus”). Type: ‘Habitat in Guinea.’ (West Tropical Africa).

*Andropogon stapfii* Hook. f., Fl. Brit. India (J.D. Hooker). 7(21): 184. 1896. Type: Tinnevelly, Palamcottah; *Wight s.n.*

*Sorghum stapfii* (Hook. f.) C.E.C. Fisch., Fl. Madras 3(9): 1735. 1931

*Andropogon verticilliflorus* Steud., Syn. Pl. Glumac. 1: 393. 1854. Type: “An praecedentis var. Ins. Borbon.”

*Sorghum verticilliflorum* (Steud.) Stapf, Fl. Trop. Afr. [Oliver et al.] 9(1): 116. 1917

*Sorghum pugionifolium* Snowden, J. Linn. Soc., Bot. 55: 240. 1955. Type: India: Punjab, Gurgaon; October 24, 1886; *J.R. Drummond 21901*

*Andropogon arundinaceus* Willd., Sp. Pl., ed. 4 [Willdenow] 4: 906. 1806, nom. illeg. later hom., non P.J. Bergius, 1767. Type: Guinea (West Tropical Africa); *P.E. Isert s.n.* (B-W).

**Distribution.** Bihar, Maharashtra, Punjab, Tamil Nadu [Pakistan].

**Habit. **Annual or Perennial.

**1200. **Sorghum bicolor** (L.) Moench, Methodus (Moench) 207. 1794 **

*Holcus bicolor* L., Mant. Pl. Altera 301. 1771. Type: “Habitat in Persia. D. Lerche. H. U.” Lectotype: Herb. Clifford: 468, Holcus 1 (BM-000647533). LT designated by Davidse in Cafferty et al. (ed.), Taxon 49: 251. 2000.

*Andropogon bicolor* (L.) Roxb., Fl. Ind. (Carey and Wallich ed.) 1: 272. 1820

*Holcus sorghum* L., Sp. Pl. 2: 1047. 1753. Type: “Habitat in India.” Lectotype: Herb. Clifford: 468,* Holcus* 1 (BM-000647533). LT designated by Davidse in Cafferty et al. (ed.). Taxon 49: 251. 2000.

*Andropogon sorghum* (L.) Brot., Fl. Lusit. 1: 88. 1804

*Sorghum vulgare* Pers., Syn. Pl. (Persoon) 1: 101. 1805

*Sorghum bicolor* (L.) Moench var. *arduini* (Körn.) Snowden, Bull. Misc. Inform. Kew 1935: 236. 1935

*Andropogon sorghum* (L.) Brot. var. *arduini* Körn., Handb. Getreidebaus 1: 312. 1885. Type: “Dies ist vielleicht in Europa die zur samengewinnung und als Grunfutterpflanze am moisten gebaute varietat, und die erste, welche in deutsche Garten gelangte.”

*Sorghum bicolor* (L.) Moench var. *picigutta* Snowden, Bull. Misc. Inform. Kew 1935: 236. 1935. Type: Burma: Rangoon; *McClelland s.n.*

*Sorghum bicolor* (L.) Moench var. *subglobosum* (Hack.) Snowden, Bull. Misc. Inform. Kew 1935: 237. 1935

*Andropogon sorghum* (L.) Brot. var. *subglobosus* Hack., Monogr. Phan. [A. DC. & C. DC.] 6: 515. 1889. Type: “Ins. Hawaii, Honolulu in hortis (Wawra).”

*Andropogon subglabrescens* Steud., Syn. Pl. Glumac. 1: 393. 1854. Type: “Abyssin, An interim, A. Sorghi var.”

*Sorghum subglabrescens* (Steud.) Schweinf. & Asch., Beitr. Fl. Aethiop. 302, 306. 1867

*Andropogon sorghum* (L.) Brot. var. *subglabrescens* (Steud.) Hack., Monogr. Phan. [A. DC. & C. DC.] 6: 519. 1889

*Sorghum subglabrescens* (Steud.) Schweinf. & Asch. var. *latum* Snowden, Bull. Misc. Inform. Kew 1935: 255. 1935. Type: Somaliland: without precise locality; *Farquharson 1*

*Sorghum subglabrescens* (Steud.) Schweinf. & Asch. var. *pabulare* Snowden, Bull. Misc. Inform. Kew 1935: 252. 1935. Type: India: Bombay, Baroda; *Ryan s.n.*

*Sorghum subglabrescens* (Steud.) Schweinf. & Asch. var. *paniculatellum* (Chiov.) Snowden, Bull. Misc. Inform. Kew 1935: 253. 1935 (as “paniculatella”)

*Sorghum subglabrescens* (Steud.) Schweinf. & Asch. var. *rugulosum* (Hack.) Snowden, Bull. Misc. Inform. Kew 1935: 252. 1935

*Andropogon sorghum* (L.) Brot. var. *rugulosus* Hack., Monogr. Phan. [A. DC. & C. DC.] 6: 508. 1889. Type: “In insulis Capitis Viridis praesertim in ins. S. Nicolae, ex Bolle (in sched. h. berol.) spontaneous.” Abyssinia: Cultivated; *Herb. Braun* (B).

*Sorghum subglabrescens* (Steud.) Schweinf. & Asch. var. *oviforme* Snowden, Bull. Misc. Inform. Kew 1935: 254. 1935. Type: India: Bombay, Puna; *Herb. Econ. Bot. Poona Agric. Coll. 72*

*Sorghum subglabrescens* (Steud.) Schweinf. & Asch. var. *rubidum* (Burkill ex Benson & Subbarao) Snowden, Bull. Misc. Inform. Kew 1935: 253. 1935

*Andropogon sorghum* (L.) Brot. subvar. *rubidus* Burkill ex Benson & Subbarao, Madras Dept. Agric. Bull. no. 55: 68, 69. 1906. Type: India: Madras, Coimbatore; *Herb. Rep. Econ. Prod. India; Barber 961*

*Andropogon sorghum* (L.) Brot. var. *mediocris* Burkill ex Benson & Subbarao, Madras Dept. Agric. Bull. no. 55: 68, 69. 1906

*Andropogon sorghum* (L.) Brot. var. *compactus* Burkill ex Benson & Subbarao, Madras Dept. Agric. Bull. no. 55: 67, 69. 1906, p.p. Type: India: Madras, Bellary; *Herb. Rep. Econ. Prod. India; Barber 17840*

*Sorghum subglabrescens* (Steud.) Schweinf. & Asch. var. *compactum* (Burkill ex Benson & Subbarao) Snowden, Bull. Misc. Inform. Kew 1935: 253. 1935

*Andropogon sorghum* (L.) Brot. var. *irungiformis* Burkill ex Benson & Subbarao, Madras Dept. Agric. Bull. no. 55: 67, 69. 1906, p.p.

*Andropogon sorghum* (L.) Brot. var. *mediocris* Burkill ex Benson & Subbarao, Madras Dept. Agric. Bull. no. 55: 67, 69. 1906, p.p.

*Sorghum subglabrescens* (Steud.) Schweinf. & Asch. var. *irungiforme* (Burkill ex Benson & Subbarao) Snowden, Bull. Misc. Inform. Kew 1935: 253. 1935

*Andropogon sorghum* (L.) Brot. var. *irungiformis* Burkill ex Benson & Subbarao, Madras Dept. Agric. Bull. no. 55: 69. 1906, p.p. Type: India: Madras, Trichinopoly; *Herb. Rep. Econ. Prod. India; Barber 17840*

*Andropogon sorghum* (L.) Brot. var. *mediocris* Burkill ex Benson & Subbarao, Madras Dept. Agric. Bull. no. 55: 69. 1906, p.p.

*Sorghum basiplicatum* Chiov var. *paniculatellum* Chiov., Monogr. Rapp. Col. Roma no. 19: 38. 1912. Type: Eritrea: without precise locality; *International Exhibition Turin 1911, 9*

*Andropogon sorghum* (L.) Brot. var. *caudatus* Hack., Monogr. Phan. [A. DC. & C. DC.] 6: 517. 1889. Type: “Colitur in Africa centrali in terra Djur (Schweinf. Ser. III, n. 180 ex p.).”

*Sorghum caudatum* (Hack.) Stapf, Fl. Trop. Afr. [Oliver et al.] 9(1): 131. 1917, p.p.

*Holcus cernuus* Ard., Saggi Sci. Lett. Accad. Padova 1: 128. t. 3. f. 1, 2. 1786

*Sorghum cernuum* (Ard.) Host, Icon. Descr. Gram. Austriac. 4: 2. t. 3. 1809

*Sorghum cernuum* (Ard.) Host var. *orbiculatum* Snowden Bull. Misc. Inform. Kew 1935: 252. 1935. Type: India: Central Procinces, Yeotmal; *Youngman 56*

*Sorghum truchmenorum* C. Koch, Linnaea 21: 442. 1848. Type: Russia, Transcaspian Province; *C. Koch s.n.*

*Sorghum cernuum* (Ard.) Host var. *truchmenorum* (C. Koch) Snowden, Bull. Misc. Inform. Kew 1935: 251. 1935

*Andropogon sorghum* (L.) Brot. var. *truchmenorum* (C. Koch) Körn., Handb. Getreidebaus 1: 315. 1885

*Sorghum cernuum* (Ard.) Host var. *subcylindricum* Snowden, Bull. Misc. Inform. Kew 1935: 252. 1935. Type: India: Bombay, Poona; *Herb Econ. Bot. Poona Agric. Coll. 65*

*Andropogon sorghum* (L.) Brot. var. *globosus* Hack., Monogr. Phan. [A. DC. & C. DC.] 6: 517. 1889. Type: India orient, colitur e. gr. Prope Seramput (Voigt. In H. Havn.) India: Bihar & Orissa, Serampur; *Voigt s.n.* (Mus. Bot. Haun.).

*Sorghum cernuum* (Ard.) Host var. *globosum* (Hack.) Snowden, Bull. Misc. Inform. Kew 1935: 251. 1935

*Andropogon sorghum* (L.) Brot. var. *yemensis* Körn., Bull. Herb. Boissier 2(App. 2): 11. 1894. Type: “Arab Jemen im Tiefland der Tehama ostl von Marraua 162. Ernte Ende Dec 1888. Arab Sudkuste Schugra 60 coll, 81.” Arabia: Yemen, east of Marraua; *Schweinfurth 162* (B).

*Sorghum cernuum* (Ard.) Host var. *yemense* (Körn.) Snowden, Bull. Misc. Inform. Kew 1935: 251. 1935

*Andropogon sorghum* (L.) Brot. var. *agricolarum* Burkill ex Benson & Subbarao, Madras Dept. Agric. Bull. no. 55: 67. 1906, p.p. Type: India: Central Provinces, Ellichpur District; *Herb. Rep. Econ. Prod. India 22941*

*Sorghum cernuum* (Ard.) Host var. *agricolarum* (Burkill ex Benson & Subbarao) Snowden, Bull. Misc. Inform. Kew 1935: 251. 1935

*Sorghum conspicuum* Snowden, Bull. Misc. Inform. Kew 1935: 226. 1935. Type: Tanzania: Tanganyika Territory, Rufiji District; *Wakefield 9* (K).

*Sorghum conspicuum* Snowden var. *orientale* Snowden, Bull. Misc. Inform. Kew 1935: 227. 1935. Type: India: Bihar & Orissa, Bhagalpur; *Alam 6* (K).

*Andropogon sorghum* (L.) Brot. var. *usaramense* Buese & Pilg., Bot. Jahrb. Syst. 32: 184. 1902 (as “usaramensis”). Type: Tanzania: Tanganyika Territory, Usaramo, Kisserawe; *Busse 395* (B).

*Sorghum conspicuum* Snowden var. *usaramense* (Buese & Pilg.) Snowden, Bull. Misc. Inform. Kew 1935: 227. 1935

*Holcus dochna* Forssk., Fl. Aegypt.-Arab. 174. 1775. Type: “Rosetta primum mihi obveniebat in horto, cultus ob pabulum Aviculatum, florens initio Novemb. Potlea, cereale vulgare in Arabia in veniebatur.” Egypt: Rashid (Rosetta); 1761; *Forsskal 108 *(C).

*Sorghum dochna* (Forssk.) Snowden, Bull. Misc. Inform. Kew 1935: 234. 1935

*Andropogon sorghum* (L.) Brot. var. *saccharatus* Hack., Monogr. Phan. [A. DC. & C. DC.] 6: 509. 1889. Type: “Habitat in India.”

*Andropogon sorghum* (L.) Brot. var. *obovatus* Hack., Monogr. Phan. [A. DC. & C. DC.] 6: 514. 1889, p.p. Type: “Vidi ex Italia sup. Pr. Veronam (Reichb. F. germ. exs. No. 2408 sub S. vulgari) Lectum, Hispania (murcia, I. Bourgeau Pl Esp. 1522), Madeira (subv. 4).”

*Sorghum dochna* (Forssk.) Snowden var. *obovatum* (Hack.) Snowden, Bull. Misc. Inform. Kew 1935: 235. 1935

*Sorghum rubens* Willd., Enum. Pl. (Willdenow) 1036. 1809.

*Andropogon rubens* (Willd.) Kunth, Révis. Gramin. 1: 164. 1829

*Andropogon sorghum* (L.) Brot. var. *irungu* Burkill ex Benson & Subbarao, Madras Dept. Agric. Bull. no. 55: 68. 1906. Type: India: Tamil Nadu, Madras, Madura; *Herb. Rep. Econ. Prod. India 15616*

*Sorghum dochna* (Forssk.) Snowden var. *irungu* (Burkill ex Benson & Subbarao) Snowden, Bull. Misc. Inform. Kew 1935: 235. 1935

*Andropogon sorghum* (L.) Brot. var. *technicus* Körn., Handb. Getreidebaus 1: 308. 1885

*Sorghum dochna* (Forssk.) Snowden var. *technicum* (Körn.) Snowden, Bull. Misc. Inform. Kew 1935: 235. 1935

*Sorghum technicum* (Körn) Battand. & Trabut, Fl. Alger (Battandier & Trabut) 128. 1895

*Andropogon sorghum* (L.) Brot. var. *pulcher* Burkill ex Benson & Subbarao, Madras Dept. Agric. Bull. no. 55: 67. 1906, p.p. Type: Burma: Saging; *Herb. Rep. Econ. Prod. India 11793a*

*Sorghum dochna* (Forssk.) Snowden var. *pulchrum* (Burkill ex Benson & Subbarao) Snowden, Bull. Misc. Inform. Kew 1935: 235. 1935

*Sorghum dochna* (Forssk.) Snowden var. *atrum* Snowden, Bull. Misc. Inform. Kew 1935: 235. 1935. Type: Burma: Saging; *Herb. Rep. Econ. Prod. India 11795a*

*Sorghum dochna* (Forssk.) Snowden var. *melliferum* Snowden, Bull. Misc. Inform. Kew 1935: 236. 1935. Type: India: Tamil Nadu, Madras, Tinnevelly; *Anstead 3*

*Andropogon sorghum* (L.) Brot. var. *wightii* Hack., Monogr. Phan. [A. DC. & C. DC.] 6: 511. 1889. Type: “Peninsula Indiae or. (Wight h. prop. N. 185).” India: Without precise Locality; *Herb. Wight propr. 185* (B).

*Sorghum dochna* (Forssk.) Snowden var. *wightii* (Hack.) Snowden, Bull. Misc. Inform. Kew 1935: 236. 1935

*Sorghum durra* (Forssk.) Stapf, Fl. Trop. Afr. [Oliver et al.] 9(1): 129. 1917, non (Forssk.) Batt. & Trab., 1895

*Holcus durra* Forssk., Fl. Aegypt.-Arab. 174. 1775. Type: “Kahirae Durra: in arab. Taam. Colitur fere unicum cereale, finale cum H. Dochn.” Yemen: Wadi Mawr; 1763; *Forsskål 111*(C).

*Andropogon sorghum* (L.) Brot. var. *durra* (Forssk.) Hack., Monogr. Phan. [A. DC. & C. DC.] 6: 516. 1889. Type: “Maxime cultus in Aegypto tota (Schweinf. 1523) sub nominee durra awageh, in Abyssinia (Schimp.) Darfur (Pfund), Senegalia (Lelievre, et Verisimiliter etiam in Africa centrali.”

*Sorghum durra* (Forssk.) Stapf var. *maximum* Snowden, Bull. Misc. Inform. Kew 1935: 251. 1935. Type: Anglo-Egyptian Sudan: Gedarif; *Punter 5*

*Andropogon sorghum* (L.) Brot. var. *aegyptiacus* Körn., Mem. Inst. Egypt. 2: 164. 1887. Type: Egypt: Assouan; *Ehrenberg s.n.* (B).

*Sorghum durra* (Forssk.) Stapf var. *aegyptiacum* (Körn.) Snowden, Bull. Misc. Inform. Kew 1935: 249. 1935

*Andropogon sorghum* (L.) Brot. var. *coimbatoricum* Burkill ex Benson & Subbarao, Madras Dept. Agric. Bull. no. 55: 68. 1906. Type: India: Madras, Coimbatore District; *Herb. Rep. Econ. Prod. India 15593*

*Sorghum durra* (Forssk.) Stapf var. *coimbatoricum* (Burkill ex Benson & Subbarao) Snowden, Bull. Misc. Inform. Kew 1935: 250. 1935

*Sorghum durra* (Forssk.) Stapf var. *fuscum* Snowden, Bull. Misc. Inform. Kew 1935: 250. 1935. Type: India: Bombay, Sind, Hyderbad; *Navani 2*

*Andropogon sorghum* (L.) Brot. var. *mediocris* Burkill ex Benson & Subbarao, Madras Dept. Agric. Bull. no. 55: 68. 1906, p.p. Type: India: Madras, Kristna; *Herb. Rep. Econ. Prod. India 15560*

*Sorghum durra* (Forssk.) Stapf var. *mediocre* (Burkill ex Benson & Subbarao) Snowden, Bull. Misc. Inform. Kew 1935: 249. 1935

*Sorghum durra* (Forssk.) Stapf var. *rivulare* Snowden, Bull. Misc. Inform. Kew 1935: 251. 1935. Type: Anglo-Egyptian Sudan: White Nile; *Punter 56*

*Sorghum durra* (Forssk.) Stapf var. *fecundum* Snowden, Bull. Misc. Inform. Kew 1935: 250. 1935. Type: India: Bombay, Sind; *Herb. Econ. Bot. Poona Agric. Coll. 40*

*Sorghum durra* (Forssk.) Stapf var. *elongatum* Snowden, Bull. Misc. Inform. Kew 1935: 250. 1935. Type: India: Bombay, Sind; *Herb. Econ. Bot. Poona Agric. Coll. 26*

*Andropogon sorghum* (L.) Brot. var. *bicolor* Körn., Bull. Herb. Boissier 2(App. 2): 11–12. 1894, non Hack., 1889

*Andropogon sorghum* (L.) Brot. var. *usorum* Körn., Bull. Herb. Boissier 2(App. 2): 12. 1894, non (Nees) Körn. & Wern., 1885

*Andropogon sorghum* (L.) Brot. var. *eois* Burkill ex Benson & Subbarao, Madras Dept. Agric. Bull. no. 55: 67. 1906. Type: India: Punjab, Dera Ismail Khan; *Herb. Rep. Econ. Prod. India 15924*

*Sorghum durra* (Forssk.) Stapf var. *eois* (Burkill ex Benson & Subbarao) Snowden, Bull. Misc. Inform. Kew 1935: 250. 1935

*Andropogon sorghum* (L.) Brot. var. *javanicus* Hack., Monogr. Phan. [A. DC. & C. DC.] 6: 517. 1889. Type: “E seminibus javanicis in horto bot. Vindobonensi cultum vidi in herb. Vind.” Austria: Vienna, cultivated in the Botanical Gardens, seed from Java (Herb. Vindob.).

*Sorghum durra* (Forssk.) Stapf var. *javanicum* (Hack.) Snowden, Bull. Misc. Inform. Kew 1935: 250. 1835

*Sorghum membranaceum* Chiov., Monogr. Rapp. Col. Roma 19, 23, 24, 27. 1912. Type: “Collezione della Colonia Eritrea Presentata all’ Esposizione Internat, Torino 1911”

*Sorghum papyrascens* Stapf, Fl. Trop. Afr. [Oliver et al.] 9(1): 134. 1917. Type: Sudan: Sennar, without special localization; *Rubber Exhibition London 1914*

*Sorghum membranaceum* Chiov. var. *ehrenbergianum* (Körn.) Snowden, Bull. Misc. Inform. Kew 1935: 231. 1935

*Andropogon sorghum* (L.) Brot. var. *ehrenbergianus* Körn., Mem. Inst. Egypt. 2: 163. 1887. Type: Egypt: near Assouan; *Ehrenberg s.n.* (B).

*Sorghum membranaceum* Chiov. var. *lateritium* (Stapf) Snowden, Bull. Misc. Inform. Kew 1935: 231. 1935

*Sorghum papyrascens* Stapf var. *lateritium* Stapf, Fl. Trop. Afr. [Oliver et al.] 9(1): 134. 1917. Type: Anglo-Egyptian Sudan: Sennar Province; *Rubber Exhibition London 1914*

*Sorghum miliiforme* (Hack.) Snowden, Bull. Misc. Inform. Kew 1935: 237. 1935

*Andropogon sorghum* (L.) Brot. var. *milliformis* Hack., Monogr. Phan. [A. DC. & C. DC.] 6: 518. 1889. Type: “Colitur in Bengaliae regione trop. (Hook. f. et Thoms.) in Ceylon.” India: Sikkim; *J.D. Hooker & T. Thomson s.n.*

*Sorghum milliforme* (Hack.) Snowden var. *rotundulum* Snowden, Bull. Misc. Inform. Kew 1935: 237. 1935. Type: Bihar & Orissa, Bhagalpur; *Alam 9*

*Sorghum milliforme* (Hack.) Snowden var. *sikkimense* Snowden, Bull. Misc. Inform. Kew 1935: 237. 1935. Type: India: Sikkim, Tukvar; *Gamble 3354a*

*Sorghum nervosum* Bess. ex Schultes, Mant. 2 (Schultes) Add. II: 669. 1827

*Andropogon besseri* Kunth, Révis. Gramin. 1: 166. 1829

*Sorghum roxburghii* Stapf, Fl. Trop. Afr. [Oliver et al.] 9(1): 126. 1917

*Andropogon sorghum* (L.) Brot. var. *hians* Stapf, Fl. Brit. India (J.D. Hooker). 7(21): 184. 1896. Type: *Wight Cat. no. 1670*

*Sorghum roxburghii* Stapf var. *hians* (Stapf) Stapf, Fl. Trop. Afr. [Oliver et al.] 9(1): 127. 1917

*Andropogon sorghum* (L.) Brot. var. *fulvus* Hack., Monogr. Phan. [A. DC. & C. DC.] 6: 512. 1889. Type: “Colitur in Madagascar (Hildebrandt 3219 pr. Nosi-be), in ins Angorna (Peters in h. berol.).” Madagascar: Nossi-Be; *Hildebrandt 3219* (K).

*Sorghum roxburghii* Stapf var. *fulvum* (Hack.) Snowden, Bull. Misc. Inform. Kew 1935: 228. 1935

*Sorghum roxburghii* Stapf var. *semiclausum* Stapf, Fl. Trop. Afr. [Oliver et al.] 9(1): 127. 1917, p.p., nom. & stat. nov.

*Sorghum roxburghii* Stapf var. *nanum* Snowden, Bull. Misc. Inform. Kew 1935: 228. 1935. Type: India: Bihar & Orissa, Patna; *Alam 2*

**Distribution.** Andhra Pradesh, Assam, Bihar, Chhattisgarh, Gujarat, Himachal Pradesh, Karnataka, Kerala, Madhya Pradesh, Maharashtra, Meghalaya, Mizoram, Odisha, Punjab, Sikkim, Tamil Nadu, Tripura, Uttar Pradesh, Uttarakhand, West Bengal [Bangladesh, Myanmar, Pakistan, Sri Lanka]. Cultivated; native to Africa.

**Habit.** Annual.

**Remarks. ***Sorghum bicolor* was divided into many infraspecific taxa by Snowden (1935), but Harlan and DeWet (1972) provided a much simpler classification, reducing most of Snowden’s taxa to synonymy.

**1201. **Sorghum controversum** (Steud.) Snowden, J. Linn. Soc., Bot. 55: 210. 1955 **

*Andropogon controversus* Steud., Syn. Pl. Glumac. 1: 391. 1854. Type: “A. laxus Roxb. Fl. Ind. 275 vix Thunberg. Ind. or.”

**Type.** Not designated. Until Roxburgh’s type specimen has been located, a specimen in Kew Herbarium, *Bourne 3270*, from Tapeswarani, Godavery District, Madras province, dated December 1907, has been selected as characteristic of the awned state of this species.

**Distribution.** Andhra Pradesh, Bihar, Madhya Pradesh, Maharashtra, Odisha, Tamil Nadu, West Bengal.

**Habit.** Perennial.

**1202. **Sorghum deccanense** Stapf in Bor, Grasses Burma, Ceylon, India & Pakistan 245. 1960 **

**Type.** India: Madhya Pradesh; *J.F. Duthie 8538, 10025*. Deccan; *Woodrow s.n.*

**Distribution.** Gujarat, Madhya Pradesh, Maharashtra, Rajasthan.

**Habit.** Annual.

**Remarks.** Records from Gujarat and Maharashtra fide Naithani (1990).

**1203. **Sorghum halepense** (L.) Pers., Syn. Pl. (Persoon) 1: 101. 1805 **

*Holcus halepensis* L., Sp. Pl. 2: 1047. 1753. Type: “Habitat in Syria, Mauritania.” Lectotype: Herb. Linn. No. 1212.7 (LINN). LT designated by Meikle in Fl. Cyprus 2: 1869. 1985.

*Milium halepense* (L.) Cav., Descr. Pl. (Cavanilles). 306. 1802

*Andropogon halepensis* (L.) Brot., Fl. Lusit. 1: 89. 1804

*Andropogon sorghum* (L.) Brot. ssp. *halepensis* (L.) Hack., Monogr. Phan. [A. DC. & C. DC.] 6: 501. 1889

*Blumenbachia halepensis* (L.) Koeler, Descr. Gram. (Koeler) 29. 1802 (as “haleppensis”)

*Andropogon miliaceus* Roxb., Fl. Ind. (Carey and Wallich ed.) 1: 276. 1820. Lectotype: *Wallich Numer. List no 8778 B*. LT designated by Snowden in J. Linn. Soc., Bot. 55: 207. 1955.

*Sorghum miliaceum* (Roxb.) Snowden, J. Linn. Soc., Bot. 55: 207. 1955

*Sorghum miliaceum* (Roxb.) Snowden var. *parvispiculum* Snowden, J. Linn. Soc., Bot. 55: 209. 1955. Type: India: Dehra Dun; *Gamble 27139* (K).

*Andropogon arundinaceus* Scop., Fl. Carniol., ed 2. 2: 274. 1772, non P.J.Bergius, 1767. Type: “Habitat in Afro Tergeftino, calamitosum.”

**Distribution.** Andaman and Nicobar Islands, Andhra Pradesh, Bihar, Chhattisgarh, Delhi, Jammu and Kashmir, Himachal Pradesh, Goa, Gujarat, Karnataka, Kerala, Madhya Pradesh, Maharashtra, Nagaland, Odisha, Punjab, Rajasthan, Tamil Nadu, Uttar Pradesh, Uttarakhand, West Bengal [Bangladesh, Sri Lanka].

**Habit.** Perennial.

**1204. **Sorghum nitidum** (Vahl) Pers., Syn. Pl. (Persoon) 1: 101. 1805 **

*Holcus nitidus* Vahl, Symb. Bot. (Vahl) 2: 102. 1791. Type: “Habitat in India Orientali.”

*Holcus fulvus* R. Br., Prodr. Fl. Nov. Holland. 199. 1810. Type: Australia: “littora Novae Hollandiae intra tropicum.”

*Sorghum fulvum* (R. Br.) P. Beauv., Ess. Agrostogr. 131, 164. 1812

*Andropogon fulvus* (R. Br.) Spreng., Pl. Min. Cogn. Pug. 2: 8. 1815

*Sorghum tropicum* Buse, Pl. Jungh. 359. 1854, nom. superfl. & illeg. for *Holcus fulvus*.

*Andropogon serratus* Thunb., Syst. Veg., ed. 14 (J. A. Murray). 903. 1784

*Sorghum serratum* (Thunb.) Kuntze, Revis. Gen. Pl. 2: 792. 1891, non (Thunb.) Roem. & Schult., 1817

*Andropogon tropicus* Spreng., Syst. Veg. (ed. 16) [Sprengel] 1: 287. 1824 (“1825”), nom. superfl. & illeg. for *Holcus fulvus*

**Distribution.** Andhra Pradesh, Assam, Bihar, Chhattisgarh, Himachal Pradesh, Jharkhand, Karnataka, Kerala, Madhya Pradesh, Manipur, Meghalaya, Nagaland, Odisha, Tamil Nadu, Uttar Pradesh, Uttarakhand, West Bengal [Bhutan, China, Myanmar, Sri Lanka].

**Habit.** Perennial.

**1205. **Sorghum propinquum** (Kunth) Hitchc., Lingnan Sci. J. 7: 249. 1931 (“1929”) **

*Andropogon propinquus* Kunth, Enum. Pl. [Kunth] 1: 502. 1833. Type: “Ins. Neu-Hannover, ad ripas.”

*Sorghum propinquum* (Kunth) Hitchc. var. *siamense* (Piper) Snowden, J. Linn. Soc., Bot. 55: 214. 1955

*Sorghum affine* A. Camus, Fl. Indo-Chine [P.H. Lecomte et al.] 7: 321. 1922, non (R. Br.) Kuntze, 1891

*Andropogon affinis* J. Presl, Reliq. Haenk. 1: 343. 1830, non R. Br., 1810. Type: Philippines: Luzon; *T. Haenke s.n.* [according to Piper, 1915] (US-76438).

*Andropogon halepensis* (L.) Brot. ssp. *siamensis* Piper, Proc. Biol. Soc. Wash. 28: 30. 1915. Syntypes: Cambodia: Pnum Penh; October, 1878; *Godefroy-lebeuf 83* (K). Siam: Pak Bawag; September 4, 1911; *Kerr 2006* (K). Siam: Near Kampang, 350 ft., growing in pampas along the banks of Mei Ping river; October 11, 1911; *Kerr 2156* (K).

**Distribution.** Tamil Nadu [China, Sri Lanka].

**Habit.** Perennial.

**Remarks.** Record for Tamil Nadu fide Naithani (1990).

**1206. **Sorghum purpureosericeum** (Hochst. ex A. Rich.) Schweinf. & Asch., Beitr. Fl. Aethiop. 310. 1867 **

*Andropogon purpureosericeus* Hochst. ex A. Rich., Tent. Fl. Abyss. 2: 469. 1850–1851. Type: “Crescit in convalle fluvii Mareb, mense Novembre; Quartin Dillon et Ant. Petit et in locis paludosis in regione Montana Walcha provinciae Sana, mense Augusto; *Schimper s.n*.”

**Distribution.** Gujarat, Karnataka, Maharashtra, Rajasthan [also in Africa].

**Habit.** Annual.

***Spodiopogon* Trin., Fund. Agrost. (Trinius) 192. 1820**

**Type. ***Spodiopogon sibiricus* Trin.

**1207. **Spodiopogon aristatus** R.J. Desai & Raole, Kew Bull. 67(1): 103. 2012 **

**Type.** India, Gujarat, Tapi District, Bunadha, 10 November 2009, *R.J. Desai 241* (holotype: BARO).

**Distribution.** Gujarat.

**Habit.** Perennial.

**1208. **Spodiopogon cotulifer** (Thunb.) Hack., Monogr. Phan. [A. DC. & C. DC.] 6: 187. 1889 **

*Andropogon cotulifer* Thunb., Syst. Veg., ed. 14 (J. A. Murray). 903. 1784 (“cotuliferum”). Type: Japonia: Nagasaki; *Thunburg s.n.*

*Eccoilopus cotulifer* (Thunb.) A. Camus, Ann. Soc. Linn. Lyon ser. 2. 70: 92. 1924

**Distribution.** Jammu and Kashmir, Meghalaya [China].

**Habit.** Perennial.

**1209. **Spodiopogon dubius** Hack., Monogr. Phan. [A. DC. & C. DC.] 6: 186. 1889 **

**Type.** “In Himalayae boreali-occidentalis regione tropica (Hook. f. et Thomos h. Ind. Or. Andropogon 11).” Nepal, alt. 10000 ft.; *J.D. Hooker. & Thomson s.n.*

**Distribution.** Himachal Pradesh, Jammu and Kashmir, Uttarakhand [China, Nepal].

**Habit.** Perennial.

**1210. **Spodiopogon jainii** V.J. Nair, A.N. Singh & N.C. Nair, Curr. Sci. 50: 730. 1981 **

**Distribution.** Madhya Pradesh.

**Habit.** Annual.

**1211. **Spodiopogon lacei** Hole, Indian Forest Rec., Bot. 5(6): 185. 1915 **

**Syntypes.** Burma: Maymyo; *Lace no. 4372*. Burma: Maymyo; *Lace no. M (6)*. Burma: Maymyo; *Lace 6025* (DD).

**Distribution.** Manipur [Myanmar].

**Habit.** Perennial.

**Remarks.** Record for India fide Naithani (1990).

**1212. **Spodiopogon rhizophorus** (Steud.) Pilg., Nat. Pflanzenfam., ed. 2 [Engler & Prantl], 14e: 119. 1940 **

*Andropogon rhizophorus* Steud., Syn. Pl. Glumac. 1: 381. 1854. Type: “Hinc praecedenti, hinc A. biaristato Steud nr. 197 affinis. *Hrbr Wall. Nr 1821*.”

*Spodiopogon albidus* Benth., J. Linn. Soc., Bot. 19: 66. 1881, nom. nud.

**Distribution.** Dadra and Nagar Haveli, Goa, Gujarat, Karnataka, Kerala, Madhya Pradesh, Maharashtra, Rajasthan, Tamil Nadu.

**Habit.** Annual.

***Thelepogon* Roth, Syst. Veg. Syst. Veg., ed. 15 bis [Roemer & Schultes]. 2: 46. 1817**

**Type. ***Thelepogon elegans* Roth.

**1213. **Thelepogon elegans** Roth, Syst. Veg., ed. 15 bis [Roemer & Schultes]. 2: 788. 1817 **

*Andropogon princeps* A. Rich., Tent. Fl. Abyss. 2: 470. 1850–1851. Type: “Crescit in campis humidis convallis fluvii Tacazze, mense Julio; Quartin Dillon.” Type: Ethiopia: Tacazze; *Quartin Dillon & Petit s.n.* (P)

*Jardinea abyssinica* Steud., Syn. Pl. Glumac. 1: 360. 1854, nom. superfl. & illeg. for *Andropogonprinceps*

*Rhiniachne princeps* (A. Rich.) Hochst. ex Steud., Syn. Pl. Glumac. 1: 360. 1854, nom. invalid. Cited as synonym of *Jardinea abyssinica*

**Type.** “In India Orientali; *Roth s.n.*”

**Distribution.** Andhra Pradesh, Bihar, Chhattisgarh, Gujarat, Karnataka, Madhya Pradesh, Maharashtra, Rajasthan, Tamil Nadu [also in Africa].

**Habit.** Annual.

***Themeda* Forssk., Fl. Aegypt.-Arab 178. 1775**

**Type. ***Themeda triandra* Forssk.

1214. Themeda anathera (Nees) Hack., Monogr. Phan. [A. DC. & C. DC.] 6: 669. 1889

*Anthistiria anathera* Nees, Syn. Pl. Glumac. (Steudel) 1: 402. 1854. Type: India Orientali; Numer. List [Wallich] no. 8773 (K).

*Androscepia anathera* (Nees) Andersson, Nov. Act. Nat. Soc. Sc. Upsal. ser. 3. 2: 249. 1856

*Themeda dacurzii *Birari, J. Bombay Nat. Hist. Soc. 70: 346. f. 1–5. 1973. Type: India: Uttar Pradesh, Dehra Dun, Mussoorie Hill; December 17, 1969; *Birari I-774* (College of Agriculture).

**Distribution.** Himachal Pradesh, Jammu and Kashmir, Uttar Pradesh, Uttarakhand [Afghanistan, Nepal, Pakistan].

**Habit.** Perennial.

**1215. **Themeda arguens** (L.) Hack., Monogr. Phan. [A. DC. & C. DC.] 6: 657. 1889 **

*Stipa arguens* L., Sp. Pl., ed. 2. 1: 117. 1762. Type: “Habitat in India.” Lectotype: Herb. Linn. No. 94.10 (LINN). LT designated by Merrill in Interpret. Rumph. Herb. Amb. 89. 1917. Epitype: Thailand. Flora of Siam 8036 (BM). Epitype designated by Cope in (ed.), SC/CEJ.

*Anthistiria arguens* (L.) Willd., Sp. Pl., ed. 4 [Willdenow] 4(1): 901. 1806

*Anthistiria pilifera* Steud., Syn. Pl. Glumac. 1: 400. 1854. Type: “Hrbr. Goering sect II nr. 145. A. arguens Morit et Zolling Verz. p. 99 nr. 373.”

**Distribution.** Andaman and Nicobar [China].

**Habit.** Annual.

**1216. **Themeda arundinacea** (Roxb.) Ridley, Trans. Linn. Soc. London, Bot. Ser. 2. 3: 401. 1893 **

*Anthistiria arundinacea* Roxb., Fl. Ind. (Carey and Wallich ed.) 1: 256. 1820. Type: “A native of Bengal.”

*Anthistiria gigantea* Cav. ssp. *arundinacea* (Roxb.) Hack., Monogr. Phan. [A. DC. & C. DC.] 6: 674. 1889

*Cymbopogon arundinaceus* (Roxb.) Schultes, Mant. 2 (Schultes) 457. 1824

*Anthistiria subsericans* Nees, Syn. Pl. Glumac. (Steudel) 1: 401. 1854. Type: India Orientali; *Numer. List [Wallich] no. 8774B*

*Themeda subsericans* (Nees) Ridl., Fl. Malay. Penin. 5: 212. 1925

*Anthistiria gigantea* Cav. subvar. *subsericans *(Nees) Hack., Monogr. Phan. [A. DC. & C. DC.] 6: 674. 1889

**Distribution.** Andhra Pradesh, Assam, Bihar, Jharkhand, Manipur, Meghalaya, Nagaland, Odisha, Tripura, Uttar Pradesh, Uttarakhand, West Bengal [Bangladesh, Bhutan, China, Myanmar, Nepal].

**Habit.** Perennial.

**1217. **Themeda caudata** (Nees) A. Camus, Fl. Indo-Chine [P.H. Lecomte et al.] 7: 364. 1922 **

*Anthistiria caudata* Nees in Hook. & Arn., Bot. Beechey Voy. 245. 1838. Type: “Hab. Prope Macao et in vicinis insulis; *G.H. Vachell no. 46*”

*Themeda gigantea* (Cav.) Hack. ssp. *caudata* (Nees) Hack., Monogr. Phan. [A. DC. & C. DC.] 6: 676. 1889, p.p.

*Androscepia gigantea* (Cav.) Brongn. var. *armata* Andersson, Nov. Act. Nat. Soc. Sc. Upsal. ser. 3. 2: 248. 1856. Type: “Hab. in insula Java; Zollinger (in sylvis p. *Tjkoya n. 1029*) et in India Orientali (Mirra Poonje: de Silva et W. Gomez: *Numer. List [Wallich] no. 8774*)”

*Themeda longispatha* (Hack.) Raizada & Jain, Indian Forester 80: 45. 1954

*Themeda gigantea* (Cav.) Hack. var. *longispatha* Hack., Monogr. Phan. [A. DC. & C. DC.] 6: 677. 1889. Type: “Terai ad pedes montium Himalaya (O. Kuntze in h. prope.).”

**Distribution.** Assam, Bihar, Jharkhand, Madhya Pradesh, Manipur, Meghalaya, Tripura, Odisha, Uttar Pradesh, Uttarakhand, West Bengal [Bhutan, Myanmar, Nepal, Sri Lanka].

**Habit.** Perennial.

**1218. **Themeda cymbaria** Hack., Monogr. Phan. [A. DC. & C. DC.] 6: 668. 1889 **

*Anthistiria cymbaria* Roxb., Hort. Beng. 6. 1814, p.p. (excl. basionym)

**Syntypes. **“India or. in montosis (Roxb.) Peninsula (*Wight 1707, 175* [h. prop.] Maisur et Carnatic (*Hook. f. Thoms*.); Ceylon (*Thwaites 3257, 38*03).”

**Distribution.** Goa, Gujarat, Karnataka, Kerala, Maharashtra, Tamil Nadu [Sri Lanka].

**Habit.** Perennial.

**1219. **Themeda hookeri** (Griseb.) A. Camus, Bull. Mus. Hist. Nat. (Paris) 26: 425. 1920 **

*Anthistiria hookeri* Griseb., Nachr. Ges. Wiss. Gottingen, Math.-Phys. Kl. 91. 1868. Type: “H. or Sikkim. 6000–9000 ft. (H. Androscep. No. 2), Khasya 5000 (H.)”

**Distribution.** Meghalaya, Sikkim [China, Nepal].

**Habit.** Perennial.

**1220. **Themeda huttonensis** Bor, Indian Forest Rec., Bot. new ser., 1(3): 96. 1938 **

**Type.** India: Naga Hills, near Laruri, on the bank of Nanteilek river; *N.L. Bor s.n.*

**Distribution.** Assam, Nagaland.

**Habit.** Perennial.

**1221. **Themeda intermedia** (Hack.) Bor, Indian Forest Rec., Bot. new ser., 1(3): 96. 1938 **

*Themeda gigantea* Hack. var. *intermedia* Hack., Monogr. Phan. [A. DC. & C. DC.] 6: 675. 1889. Type: “In montium Khasia reg. trop. (Hook. f. et Thoms. sub Androscepia nr. 4).”

**Distribution.** Arunachal Pradesh, Assam, Manipur, Meghalaya, Nagaland [Bhutan, China].

**Habit.** Perennial.

**1222. **Themeda laxa** (Andersson) A. Camus, Bull. Mus. Hist. Nat. (Paris) 26: 423. 1920 **

*Anthistiria laxa* Andersson, Nov. Act. Nat. Soc. Sc. Upsal. ser. 3. 2: 243. 1856. Type: “Hab. in Nepalia.” *Numer. List [Wallich] no. 8775* (S).

**Distribution.** Andhra Pradesh, Assam, Bihar, Chhattisgarh, Jharkhand, Karnataka, Madhya Pradesh, Maharashtra, Manipur, Meghalaya, Odisha, Rajasthan, Tamil Nadu [Bhutan, Nepal].

**Habit.** Perennial.

**1223. **Themeda mooneyi** Bor, Kew Bull. 1951: 451. 1952 **

**Type.** India: Orissa, near Pottangi, Koraput District, alt. 4100 ft.; October 1950; *H.F. Mooney 4064* (K).

**Distribution.** Odisha.

**Habit.** Perennial.

**1224. **Themeda odishae** Chorghe, K. Prasad, Prasanna & Y. V. Rao, Phytotaxa 245 (2): 183. 2016 **

**Type.** India, Orissa, Gajapati District, Mahendragiri Hills, 17th September 2014, *Alok Chorghe & K. Prasad* 4209 (BSID, CAL).

**Distribution.** Odisha.

**Habit.** Perennial.

**1225. **Themeda palakkadensis** Chorghe, K Prasad & Lakshmin., Taiwania 64(3): 231. 2019 **

**Type.** India, Kerala, Palakkad District, Elvai Malai, 12th November, 2016, *K. Prasad 8485 *(CAL, BSI).

**Distribution.** Kerala.

**Habit.** Perennial.

**1226. **Themeda quadrivalvis** (L.) Kuntze, Revis. Gen. Pl. 2: 794. 1891 var. **quadrivalvis

*Andropogon quadrivalvis* L., Syst. Veg., ed. 13. 1: 758. 1774, replaced synonym *Andropogon nutans* L. (1771), nom. illeg*.*, non L. (1753). Lectotype: Herb. Linn. No. 1211.5 (LINN). LT designated by Cope in Cafferty et al. (ed.), Taxon 49: 246. 2000.

*Anthistiria scandens* Roxb., Fl. Ind. (Carey and Wallich ed.) 1: 253. 1820. Type: “A Native of Bengal.”

*Themeda ciliata* Hack., Monogr. Phan. [A. DC. & C. DC.] 6: 664. 1889, nom. superfl. & illeg.

*Anthistiria ciliata* L.f., Suppl. Pl. 113. 1782 (“1781”), nom. superfl. & illeg.

**Distribution.** Andhra Pradesh, Bihar, Goa, Gujarat, Karnataka, Kerala, Maharashtra, Manipur, Nagaland, Odisha, Rajasthan, Sikkim, Tamil Nadu, Uttar Pradesh, Uttarakhand, West Bengal [Africa, China, Myanmar, Nepal].

**Habit.** Annual.

**1227. **Themeda quadrivalvis** (L.) Kuntze var. **helferi** (Hack.) Bor, Grasses Burma, Ceylon, India & Pakistan 252. 1960 **

*Themeda helferi* Hack., Monogr. Phan. [A. DC. & C. DC.] 6: 665. 1889. Type: Burma: Andaman Islands (Bay of Bengal), Tenasserim; March 1862; *J.W. Helfer 6809*

**Distribution.** Andaman and Nicobar Islands, Andhra Pradesh, Madhya Pradesh, Uttar Pradesh [Myanmar].

**Habit. **Annual.

**Remarks.** Records for Andaman and Nicobar Islands and Madhya Pradesh fide Naithani.

**1228. **Themeda sabarimalayana** Sreek. & V.J. Nair, Bull. Bot. Surv. India 29(1–4): 127. 1989 (“1987”) **

**Type.** India: Kerala, Pathanamthitta District, on way to Sabarimala, alt. 1500 ft.; December 23, 1980; *P.V. Sreekumar 69433 *(CAL).

**Distribution.** Kerala, Tamil Nadu.

**Habit.** Perennial.

**1229. **Themeda saxicola** Bor, Kew Bull. 1951: 452. 1952 **

**Type.** India: Orissa, Raisili Village, Koraput District, alt. 3200 ft.; October 25, 1950; *H.F. Mooney 4241* (K).

**Distribution.** Odisha.

**Habit.** Perennial.

**1230. **Themeda strigosa** (Buch.- Ham. ex Hook. f.) A. Camus, Bull. Mus. Hist. Nat. (Paris) 26: 423. 1920 **

*Anthistiria strigosa* Buch.- Ham. ex Hook. f., Fl. Brit. India (J.D. Hooker). 7(21): 214. 1896. Type: India: Assam; *Buchanan-Hamilton s.n.* Bihar, Monghyr; *Herb Calcutta*

**Distribution. **Assam, Bihar, Maharashtra, Uttar Pradesh, West Bengal.

**Habit.** Perennial.

**1231. **Themeda tremula** (Nees) Hack., Monogr. Phan. [A. DC. & C. DC.] 6: 667. 1889 **

*Anthistiria tremula* Nees, Syn. Pl. Glumac. (Steudel) 1: 401. 1855. Type: Sri Lanka; *Heyne s.n.* (Numer. List [Wallich] no. 8765).

*Androscepia tremula* (Nees) Andersson, Nov. Act. Nat. Soc. Sc. Upsal. ser. 3. 2: 247. 1856

*Themeda tremula* (Nees) Hack. var. *thwaitesii* (Hook. f.) Senaratna, Grasses of Ceylon 198. 1956

*Anthistiria thwaitesii* Hook. f., Fl. Brit. India (J.D. Hooker). 7(21): 215. 1896. Type: Sri Lanka; *Thwaites s.n.*

*Anthistiria tremula* Nees var. *thwaitesii* (Hook. f.) Hook. f. in Trimen, Handb. Fl. Ceylon 5: 250. 1900

*Themeda thwaitesii* (Hook. f.) A. Camus, Bull. Mus. Hist. Nat. (Paris) 26: 424. 1920

*Anthistiria tremula* Nees var. *brunnea* Hook. f. in Trimen, Handb. Fl. Ceylon 5: 250. 1900. Syntypes: Abundant throughout the Island. Moragala; *Trimen s.n.* Madulsema; *Pearson s.n.*

*Themeda tremula* (Nees) Hack. var. *brunnea* (Hook. f.) Senaratna, Grasses of Ceylon 198. 1956

**Distribution.** Andhra Pradesh, Bihar, Goa, Gujarat, Karnataka, Kerala, Maharashtra, Meghalaya, Rajasthan, Tamil Nadu, Uttar Pradesh [Sri Lanka].

**Habit.** Perennial.

**1232. **Themeda triandra** Forssk., Fl. Aegypt.-Arab. 178. 1775 **

*Anthistiria australis* R. Br., Prodr. Fl. Nov. Holland. 200. 1810. Type: Australia: “littora Novae Hollandiae intra tropicum.” Australia; *R. Brown 6194* (BM).

*Themeda australis* (R. Br.) Stapf, Fl. Trop. Afr. [Oliver et al.] 9(3): 420. 1919, in obs.

*Anthistiria glauca* Desf., Fl. Atlant. 2: 380. t. 254. 1799. Type: “Habitat in collibus arenosis prope Bone, Constantine et aliis locis.”

*Anthistiria imberbis* Retz., Observ. Bot. (Retzius) 3: 11. 1783. Type: South Africa: Cape, Cape of Good Hope; *Sparrmann s.n.* (LD).

*Themeda imberbis* (Retz.) T. Cooke, Fl. Bombay 2: 993. 1908

*Anthistiria paleacea* Ball, J. Linn. Soc., Bot. 16: 734. 1878. Type: “In collibus et locis aridis raro, loco natali non indicto.”

*Anthistiria punctata* Hochst. ex A. Rich., Tent. Fl. Abyss. 2: 448. 1850–1851. Syntypes: Ethiopia: Scholoda; October 3, 1837; *G.H.W. Schimper Abyss., sect. I, 73* (P). Ethiopia: in monte Selleuda nec non in montibus probe Tecli, in provincia Sana, mensibus Augusto ad Octobrem; Schimper, pl. Schimp. Abyss., sect. III, 1555 (P). Ethiopia: in locis montosis juxta Memsah; *Quartin Dillon s.n. *(P).

*Stipa arguens* Thunb., Prodr. Pl. Cap. 20. 1794, non L., 1762

*Calamina imberbis* sensu (Retz.) Roem. & Schult., Syst. Veg., ed. 15 bis [Roemer & Schultes]. 2: 810. 1817, non P. Beauv., 1812

*Themeda forskalii* Hack., Monogr. Phan. [A. DC. & C. DC.] 6: 659. 1889

**Type.** “Yemen in montibus ad Hadiyah”; 1763; *Forsskål s.n.* (C (destroyed)).

**Distribution. **Andhra Pradesh, Assam, Bihar, Chhattisgarh, Goa, Gujarat, Haryana, Himachal Pradesh, Jammu and Kashmir, Karnataka, Kerala, Madhya Pradesh, Maharashtra, Manipur, Meghalaya, Nagaland, Punjab, Rajasthan, Tamil Nadu, Uttar Pradesh [Bhutan, China, Myanmar, Nepal, Sri Lanka].

**Habit. **Perennial.

**1233. **Themeda villosa** (Poir.) A. Camus, Fl. Indo-Chine [P.H. Lecomte et al.] 7: 364. 1922 **

*Anthistiria villosa* Poir., Encycl. (Lamarck) Suppl. 1: 396. 1810. Type: “Cette plante a été recueillie a Java par Commerson (V.S. in herb Lam. & Desfont.).”

*Themeda gigantea* (Cav.) Hack. ssp. *villosa* (Poir.) Hack., Monogr. Phan. [A. DC. & C. DC.] 6: 675. 1889

*Aristaria mutica* Hassk., Tijdschr. Natuurl. Gesch. Physiol. 10: 117. 1843. Type: Cited Anth. Gigantea Cav. (Wild. Sp. 4: 902, 7. Spreng vol.1.291.15)

*Heterelytron scabrum* Jungh., Tijdschr. Natuurl. Gesch. Physiol. 7: 294. 1840. Type: Indonesia: Java “Per totam insulam locis apricis, siccis usque ad 2000 pedum altitudinem.”

*Themeda gigantea* sensu Alston in Trimen, Handb. Fl. Ceylon 6: 336. 1931, non (Cav.) Hack., 1889

*Anthistiria mutica* sensu Steud., Syn. Pl. Glumac. 1: 401. 1854, non, Hassk., 1843

*Androscepia mutica* Andersson ex Hook. f., Fl. Brit. India (J.D. Hooker). 7(21): 217. 1896, nom. nud., pro. syn.

**Distribution.** Arunachal Pradesh, Assam, Bihar, Manipur, Meghalaya, Nagaland, Tripura, Odisha, Uttar Pradesh, Uttarakhand, West Bengal [Bangladesh, Bhutan, China, Nepal, Sri Lanka].

**Habit. **Perennial.

***Thuarea* Pers., Syn. Pl. (Persoon) 1: 110. 1805**

**Type. ***Thuarea sarmentosa* Pers.

**1234. **Thuarea involuta** (G. Forst.) Sm., Cycl. (Rees) 35(2): **Thuarea** [no. 3] [unpaginated]. 1817 **

*Ischaemum involutum* G. Forst., Fl. Ins. Austr. 73. 1786. Type: “Societatis insulae et passim intra tropicos.”

*Ornithocephalochloa arenicola* Kurz, J. Bot. 13: 332. t. 171. f. 1–18. 1875. Type: “Hab. in arenosis calcareis littorum ins. Katchall, frequens. – Gramen valde peculiare nullo generi inter Phalarideas arcte affine.”

*Thuarea sarmentosa* Pers., Syn. Pl. (Persoon) 1: 110. 1805. Type: “Hab. in Madagascariae arenos.”

**Distribution.** Andaman and Nicobar Islands, Lakshadweep [China, Sri Lanka].

**Habit.** Perennial.

***Trilobachne* Schenck ex Henrard, Meded. Rijks-Herb. 67: 4. 1931**

**Type. ***Trilobachne cookei* (Stapf) Schenck ex Henrard (*Polytoca cookei* Stapf).

**1235. **Trilobachne cookei** (Stapf) Schenck ex Henrard, Meded. Rijks-Herb. 67: 4. 1931 **

*Polytoca cookei* Stapf, Hooker’s Icon. Pl. 24: t. 2333. 1894. Type: “Hab. in sylvis insulae Salsetta Bombay. *Jacquemont 706*. Bombay Herb. Dalzell, ad Mahableshewar, *Woodrow; October 1893*; Concan Herb. Ind. Or. Hook. f. et Th. In concan meridionali in Canara septentrionali, *Dr. Lisboa Comm. 1891*.”

**Distribution.** Goa, Gujarat, Karnataka, Maharashtra, Dadra and Nagar Haveli, Tamil Nadu [Sri Lanka].

**Habit.** Annual.

***Tripidium* H. Scholz in Willdenowia 36(2): 664. 2006**

*Ripidium *Trin., Fund. Agrost. (Trinius) 169. 1820, non Bernh. (1800).

**Type. ***Tripidium ravennae* (L.) H. Scholz. (*Andropogon ravennae* L).

**1236. **Tripidium arundinaceum** (Retz.) Welker, Voronts. & E.A. Kellogg, Taxon 68 (2): 255. 2019 **

*Saccharum arundinaceum* Retz., Observ. Bot. (Retzius) 4: 14. 1786–1787. Type: “Colitur juxta sepes et ad stagnorum margines prope Tranquebar, alibi rarius. Inde misit honor. *Koenig*. “

*Erianthus arundinaceus* (Retz.) Jeswiet, Arch. Suikerindustr. Ned. Ned.-Indie 33: 399. 1925

*Saccharum procerum* sensu Trimen, Syst. Cat. Fl. Pl. Ceylon 106. 1885, non Roxb., 1820

*Saccharum arundinaceum* var. *trichophyllum* (Hand.-Mazz.) S.M. Phillips & S.L. Chen, Novon 15(3): 469–470. 2005. [This combination is not made here.]

*Erianthus griffithii* var. *trichophyllus* Hand.-Mazz., Akad. Wiss. Wien, Math.-Naturwiss. Kl., Anz. 58: 154. 1921

*Erianthus trichophyllus* (Hand.-Mazz.) Hand.-Mazz., Akad. Wiss. Wien, Math.-Naturwiss. Kl., Anz. 62: 254. 1926

**Distribution.** Andaman and Nicobar Islands, Arunachal Pradesh, Assam, Bihar, Chhattisgarh, Himachal Pradesh, Karnataka, Kerala, Madhya Pradesh, Maharashtra, Meghalaya, Odisha, Punjab, Rajasthan, Sikkim, Tamil Nadu, Uttar Pradesh, West Bengal [Bangladesh, Bhutan, China, Myanmar, Sri Lanka].

**Habit.** Perennial.

**Remarks. **Welker et al. (2019) suggest that *Saccharum arundinaceum* var. *trichophyllum* probably belongs in *Tripidium*, but have chosen not to make the combination in *Tripidium* pending additional data. The variety is placed here provisionally.

**1237. **Tripidium bengalense** (Retz.) H. Scholz, Willdenowia 36(2): 664. 2006 **

*Saccharum bengalense* Retz., Observ. Bot. (Retzius) 5: 16. 1788 (“1789”). Type: “Habitat Bengaliae, unde benevole communicavit cel. Koening.”

*Saccharum ciliare* T. Anderson, Ofvers. Forh. Kongl. Svenska Vetensk.-Akad. 155. 1855. Type: India: Madhya Pradesh?, Balapur; *Hugel 877* (W).

*Erianthus ciliaris* (T. Anderson) Jeswiet, Arch. Suikerindustr. Ned. Ned.-Indie 33: 399. 1925

*Saccharum munja* Roxb., Fl. Ind. (Carey and Wallich ed.) 1: 250. 1820. Type: India: “A native of the countries about Benares, Botanical Gardern, Calcutta.”

*Erianthus munja* (Roxb.) Jeswiet, Arch. Suikerindustr. Ned. Ned.-Indie 33: 399. 1925

*Saccharum sara* Roxb., Fl. Ind. (Carey and Wallich ed.) 1: 249. 1820. Type: India: “found in the vicinity of Calcutta but rather rare.”

*Erianthus sara* (Roxb.) Rumke, Arch. Suikerindustr. Ned. Ned.-Indie Meded. 2: 223. t. 2. 1934

*Saccharum arundinaceum* sensu Hook. f., Fl. Brit. India (J.D. Hooker). 7(21): 119–120. 1896, p.p., non Retz., 1786

**Distribution.** Assam, Bihar, Gujarat, Jammu and Kashmir, Himachal Pradesh, Madhya Pradesh, Rajasthan, Uttar Pradesh, Uttarakhand, West Bengal [Pakistan].

**Habit.** Perennial.

**1238. **Tripidium procerum** (Roxb.) Welker, Voronts. & E.A. Kellogg, Taxon 68(2): 2019 **

*Saccharum procerum* Roxb., Fl. Ind. (Carey and Wallich ed.) 1: 248. 1820. Type: “A native of Bengal.”

**Distribution.** Arunachal Pradesh, Assam, Bihar, Meghalaya, Manipur, Tripura, Sikkim, West Bengal [Bangladesh, China, Nepal].

**Habit.** Perennial.

**1239. **Tripidium ravennae** (L.) H. Scholz, Willdenowia 36(2): 664. 2006 **

*Andropogon ravennae* L., Sp. Pl., ed. 2. 2: 1481. 1763. Lectotype: “Habitat in Italia.” Herb. Linn. -77.4. LT designated by S. Cafferty, C.E. Jarvis & N.J. Turland. Taxon 49(2): 246. 2000

*Saccharum ravennae* (L.) L., Syst. Veg., ed. 13. 88. 1774

*Erianthus ravennae* (L.) P. Beauv., Ess. Agrostogr. 14. 162. 1812

*Ripidium ravennae* (L.) Trin., Fund. Agrost. (Trinius) 169. 1820. Type: “Habitat [in Italia.]” Lectotype: Herb. Linn. No. 77.4 (LINN). LT designated by Cope in Cafferty et al. (ed.), Taxon 49: 246. 2000.

*Erianthus elephantinus* Hook. f., Fl. Brit. India (J.D. Hooker). 7(21): 122. 1896. Syntypes: India: Assam; *Griffith s.n.*(K). India: Assam;* Simonds s.n.*(K).

**Distribution.** Arunachal Pradesh, Assam, Bihar, Delhi, Himachal Pradesh, Jammu and Kashmir, Manipur, Maharashtra, Meghalaya, Nagaland, Punjab, Rajasthan, Sikkim, Uttar Pradesh [Afghanistan, China, Myanmar].

**Habit.** Perennial.

***Triplopogon* Bor, Kew Bull. 1954: 52. 1954**

**Type. ***Triplopogon spathiflorus* (Hook. f.) Bor (*Ischaemum spathiflorum* Hook. f.).

**1240. **Triplopogon ramosissimus** (Hack.) Bor, Kew Bull. 1954: 501. 1954 **

*Ischaemum ramosissimum* Hack., Monogr. Phan. [A. DC. & C. DC.] 6: 249. 1889. Type: “Asia (Hugel in h. vind, no 1977, sine dubio ex Ind. or.)” India: Bombay; *Jacquemont 797* (K).

*Ischaemum spathiflorum* Hook. f., Fl. Brit. India (J.D. Hooker). 7(21): 138. 1896. Type: India: Bombay, Hills of Salsette Island; *Jacquemont no. 797*

*Sehima spathiflora* (Hook. f.) Blatt. & McCann, J. Bombay Nat. Hist. Soc. 32: 23. 1927 (as “spathiflorum”)

*Triplopogon spathiflorus* (Hook. f.) Bor, Kew Bull. 1954: 54. 1954

**Distribution.** Maharashtra.

**Habit.** Annual.

***Tripsacum* L., Syst. Nat., ed. 10. 2: 1261. 1759**

**Lectotype. ***Tripsacum dactyloides* (L.) L. (*Coix dactyloides* L.). LT designated by Hitchcock in Contr. U. S. Natl. Herb. 12: 124. 1908.

**1241. **Tripsacum dactyloides** (L.) L., Syst. Nat. ed. 10. 2: 1261. 1759 **

*Coix dactyloides* L., Sp. Pl. 2: 972. 1753. Type: “Habitat in America.” Lectotype: Herb. Linn. No. 1097.1 (LINN). LT designated by Hitchcock in Contr. U. S. Natl. Herb. 12: 124. 1908.

*Coix angulata* Mill., Gard. Dict., ed. 8. 1768. *Coix* no. 2.

*Dactylodes angulatum* (L.) Kuntze, Revis. Gen. Pl. 2: 773. 1891

*Ischaemum glabrum* Walter, Fl. Carol. 249. 1788. Type: USA: South Carolina, No precise locality cited.

*Tripsacum monostachyum* Willd., Sp. Pl., ed. 4 [Willdenow] 4(1): 202. 1805. Type: “Habitat in Carolina meridionali.”

**Distribution.** India [also in Europe, North America]. Native to Central and North America.

**Habit.** Perennial.

**Remarks.** Distribution in India not recorded. Likely known only in cultivation.

**1242. **Tripsacum laxum** Nash, N. Amer. Fl. 17: 81. 1909 **

*Tripsacum fasciculatum* Trin. ex Asch., Verh. Bot. Vereins Prov. Brandenburg 17:79, adnot. 1875, non (L.) Raspail, 1825. Lectotype: Mexico: Veracruz, Hacienda de la Laguna; 1836; *C.J.W. Schiede 947*; ILT: US-727179 (fragm. ex LE-TRIN)). LT designated by de Wet et al. Phytologia 33: 208. 1976.

**Type.** Southern Mexico: ‘Hacienda de la Laguna, Mexico.’

**Distribution.** Andhra Pradesh, Bihar, Kerala, Tamil Nadu [also in North America, South America]. Native to the Americas, introduced into India.

**Habit.** Perennial.

***Vossia* Wall. & Griff., J. Asiat. Soc. Bengal 5: 572. 1836, nom. cons.**

**Type. ***Vossia procera* Wall. & Griff., nom. illeg*.* (*Ischaemum cuspidatum* Roxb., *Vossia cuspidata* (Roxb.) Griff.).

**1243. **Vossia cuspidata** (Roxb.) Griff., Not. Pl. Asiat. 3: 70, Index 12. 1851 (as “cuspidatum”) **

*Ischaemum cuspidatum* Roxb., Fl. Ind. (Carey and Wallich ed.) 1: 325. 1820. Type: “A native of Bengal, where it is found floating on pools of sweet water and blossoming about the close of the rains in October.”

*Vossia procera* Wall. & Griff., J. Asiat. Soc. Bengal 5: 573. 1836, nom. illeg. superfl.

**Distribution.** Assam, West Bengal [Myanmar].

**Habit.** Perennial.

**Zea L., Sp. Pl. 2: 971. 1753**

**Type. ***Zea mays* L.

**1244. **Zea mays** L., Sp. Pl. 2: 971. 1753 ssp. **mays

*Mays zea* Gaertn., Fruct. Sem. Pl. 1: 6, t. 1. 1789

*Zea erythrolepis* Bonaf., Hist. Nat. Mais 30. t. 5. 1836. Type: “Le Mais a farle rouge cultive sur les rives du Missouri.”

*Zea hirta* Bonaf., Ann. Sci. Nat. (Paris) 17: 158. 1829

*Zea saccharata* Sturtev., New York Agric. Exp. Sta. Bull. 156. 1885. Type: Group name for “Sweet corns.”

*Zea segetalis* Salisb., Prodr. Stirp. Chap. Allerton 28. 1796, nom. superf. & illeg.

*Mays americana* Baumg., Enum. Stirp. Transsilv. 3: 281. 1816, nom. superfl. & illeg.

*Mayzea cerealis* Raf., Med. Fl. 2: 241. 1830, nom. superfl. & illeg. for *Zea mays*

**Type.** “Habitat in America.” Lectotype: Herb. Linn. No. 1096.1 (LINN). LT designated by Iltis & Doebley in Amer. J. Bot. 67: 1001. 1980.

**Distribution.** Andhra Pradesh, Assam, Bihar, Delhi, Goa, Gujarat, Himachal Pradesh, Karnataka, Madhya Pradesh, Maharashtra, Rajasthan, Tamil Nadu, Tripura, Uttar Pradesh [China, Pakistan, Sri Lanka]. Cultivated; derived from species native to Mexico.

**Habit.** Annual.

### Subfamily Danthonioideae N.P. Barker & H.P. Linder, 2001

Danthonioideae includes 281 species described in the monograph by Linder et al. (2010). As such it is the only grass subfamily to have a complete species-level revision based on up-to-date phylogenetic data. The subfamily has no obvious morphological synapomorphy, although Verboom et al. (1994) have suggested that haustorial synergids may be universally shared and uniquely derived. However, this character is difficult to observe and has only been recorded for a subset of the genera. None of the species has any particular economic importance.

Taxonomic editor H. Peter Linder, University of Zurich, Switzerland.

***Cortaderia* Stapf, Gard. Chron. ser. 3., 22: 378. 1897, nom. cons.**

**Type. ***Cortaderia selloana* (Schult. & Schult.f.) Asch. & Graebn. (*Arundo selloana* Schult. & Schult. f.).

**1245. **Cortaderia selloana** (Schult. & Schult. f.) Asch. & Graebn., Syn. Mitteleur. Fl. [Ascherson & Graebner]. 2(1): 325. 1900 **

*Arundo selloana* Schult. & Schult. f., Mant. 3 (Schultes & Schultes f.) Add. i Mant. cl. iii: 605. 1827. Type: Brazil: Monte Video; *F. Sellow 316* (B).

*Gynerium argenteum* Nees, Fl. Bras. Enum. Pl. 2(1): 462. 1829. Type: “Habitat in Sebastianopolin et in provincia S. Pauli”; *Martius s.n. *(B). Uruguay: ad Monte-Video; *Sellow 570* (B).

*Cortaderia argentea* (Nees) Stapf, Gard. Chron. ser. 3, 22: 396. 1897

*Cortaderia dioica* Speg., Anales Mus. Nac. Buenos Aires ser. 2, 7: 194. 1902

*Arundo dioeca* Spreng., Syst. Veg. (ed. 16) [Sprengel] 1: 361. 1824 (“1825”), non *A. dioica *Lour., 1790. Type. “Monte Video. *Sello* [*s.n., s.d.*].”

**Distribution.** Tamil Nadu; cultivated [China]. Native to South America.

**Habit.** Perennial.

**Remarks.** It is unclear whether this species is naturalized anywhere in India. Taxonomic editor Linder comments that *Cortaderia dioica* and *Arundo dioeca* are both based on the same type as *Arundo selloana*.

***Schismus* P. Beauv., Ess. Agrostogr. 73. 1812**

**Lectotype. ***Schismus marginatus* P. Beauv., nom. illeg. (*Festuca calycina* Loefl., *Schismus calycinus* (Loefl.) K. Koch). LT designated by Pfeiffer, Nomencl. Bot. 2: 1075. 1874.

**1246. **Schismus arabicus** Nees, Fl. Afr. Austral. Ill. 422. 1841 **

*Schismus marginatus* sensu Hook. f., Fl. Brit. India (J.D. Hooker). 7(21): 336. 1896, non P. Beauv., 1812

**Type.** Egypt: Chabba, “In valle Hamme Arabiae pereaeae; March 21, 1835; *Schimper 391* (W).

**Distribution.** Jammu and Kashmir, Ladak [Afghanistan, China, Pakistan].

**Habit.** Annual.

**Remarks. **Distribution in India fide Naithani (1990).

**1247. **Schismus barbatus** (L.) Thell., Bull. Herb. Boissier Ser. 2. 7: 391. 1907 **

*Festuca barbata* L., Demonstr. Pl. 3. 1753. Type: “Habitat in Hispania.” Lectotype: Herb. Linn. No. 92.26 (LINN). LT designated by Scholz in Cafferty et al. (ed.), Taxon 49: 250. 2000.

*Festuca calycina* Loefl., Iter. Hisp. 116. 1758. Type: Not given.

*Schismus calycinus* (Loefl.) K. Koch, Linnaea 21(3): 397. 1848

*Schismus brevifolius* Nees, Fl. Afr. Austral. Ill. 422. 1841. Type: South Africa: “In monte ad Bokpoort”, alt. 4500 ft.; *Drège s.n.* (BM).

*Schismus fasciculatus* P. Beauv., Ess. Agrostogr. 74. t. 15. f. 4. 177. 1812. Type: “Plante communiqué par M. Persoon et M. Balbis.”

**Distribution.** Northwest India; specific states not recorded [Afghanistan, China, Pakistan].

**Habit.** Annual.

***Tenaxia* N.P. Barker & H.P. Linder, Ann. Missouri Bot. Gard. 97(3): 350. 2010**

**Type. ***Tenaxia stricta* (Schrad.) N.P. Barker & H.P. Linder (*Danthonia stricta* Schrad.).

**1248. **Tenaxia cachemyriana** (Jaub. & Spach) N.P. Barker & H.P. Linder, Ann. Missouri Bot. Gard. 97(3): 352. 2010 **

*Danthonia cachemyriana* Jaub. & Spach, Ill. Pl. Orient. 4: 46. t. 331. 1851. Type: India: Kashmir [“Cachemyriani”]; August, 1831; *Jacquemont s.n. *(P).

*Danthonia exilis* Hook. f., Fl. Brit. India (J.D. Hooker). 7(22): 281. 1896. Type: India: Kashmir, Tilail and Bargil, alt. 9000–11000 ft.; *C.B. Clarke 30684 (K).* Gurail, alt. 8000–9000 ft.; *Duthie 12591 (K).*

*Danthonia schneideri* Pilg. var. *minor* (Hook. f.) Karthik. in S. Karthikeyan et al., Fl. Ind. ser. 4, 1 (Monocotyledon): 204. 1989

*Danthonia cachemyriana* Jaub. & Spach var. *minor *Hook. f., Fl. Brit. India (J.D. Hooker). 7(22): 282. 1896. Type: India: Sikkim, Yeumting, 12000 ft.; September 2, 1849; *Danthonia 2 Herb. R. Strachey & J.E. Winterbottom* (K).

**Distribution.** Jammu and Kashmir, Sikkim, Uttar Pradesh, Uttarakhand [Afghanistan, Bhutan, China, Nepal, Pakistan].

**Habit.** Perennial.

**1249. **Tenaxia cumminsii** (Hook.f.) N.P. Barker & H.P. Linder, Ann. Missouri Bot. Gard. 97(3): 352. 2010 **

*Danthonia cumminsii* Hook. f., Fl. Brit. India (J.D. Hooker). 7(22): 282. 1896. Type: Bhutan: Guatong in the Sikkim frontier, alt. 12000 ft.; *Cummins s.n.* (K).

*Danthonia schneideri* Pilg., Repert. Spec. Nov. Regni Veg. 17: 131. 1921. Type: China: Yunan, Lichiang, alt. 13000 ft.; September, 1914; *C. Schneider 2342* (B).

*Danthonia jacquemontii* Bor, Kew Bull. 1952: 80–81. 1952, nom. invalid

**Distribution.** Himachal Pradesh, Jammu and Kashmir, Sikkim, Uttar Pradesh, Uttarakhand [China, Pakistan].

**Habit.** Perennial.

### Subfamily Chloridoideae Kunth ex Beilschm., 1833

Plants annual or perennial, rhizomatous, stoloniferous, or caespitose. Leaves, inflorescences, and spikelets variable, with no obvious shared morphological character. Glumes 2, disarticulation above the glumes.

Chloridoideae includes over 1700 species, of which all but two (the southern African *Ellisochloa papposa* and *E. rangei*) exhibiting C4 photosynthesis and associated radiate chlorenchyma in the leaves (Peterson et al. 2011). Many chloridoids have characteristic microhairs in which the apical cell is enlarged and bulbous (VandenBorre and Watson 1997). Although the subfamily is definitely monophyletic, it is unclear which morphological characters are synapomorphic (Kellogg 2015b). Members of the subfamily are among the most drought-tolerant of the grasses, and a few (e.g. *Oropetium* spp., *Sporobolus stapfianus*) are so-called resurrection plants, which are able to dry out almost completely and then resume photosynthesis upon rewatering (VanBuren et al. 2015; Yobi et al. 2017). The subfamily includes few crop species, but *Eragrostis tef* (tef) and *Eleusine coracana* (finger millet) are particularly important in parts of Africa where productivity is critical in the face of drought. A set of phylogenetic studies by Peterson and colleagues (Peterson et al. 2007, 2010a, 2010b, 2011, 2012, 2014a; 2014b, 2015; 2016; Snow and Peterson 2012a, 2012b) has provided considerable insight into the phylogeny and generic limits in this subfamily.

Taxonomic editor Paul M. Peterson, Smithsonian Institution, Washington, DC.

### Incertae sedis

***Indopoa* Bor, Kew Bull. 1958: 225. 1958**

**Type. ***Indopoa paupercula* (Stapf) Bor ex Ramamoorthy (*Tripogon pauperculus* Stapf).

**1250. **Indopoa paupercula** (Stapf) Bor ex Ramamoorthy, Fl. Hassan Dist. [Saldanha & Nicolson] 735. 1976 **

*Tripogon pauperculus* Stapf, Hooker’s Icon. Pl. 25: t. 2442. 1896. Type: India: Bombay, Western Ghats; September 20, 1895; *G.M. Woodrow s.n.* (K).

**Distribution.** Karnataka, Kerala, Maharashtra, Tamil Nadu.

**Habit.** Annual.

**Remarks.**Soreng et al. (2017) place this taxon in Chloridoideae, incertae sedis.

***Myriostachya* (Benth.) Hook. f., Fl. Brit. India (J.D. Hooker). 7: 327. 1896**

*Eragrostis* sect. *Myriostachya* Benth., J. Linn. Soc., Bot. 19: 117. 1881

**Type. ***Myriostachya wightiana* (Nees) Hook. f. (*Leptochloa wightiana* Nees ex Steud.).

**1251. **Myriostachya wightiana** (Nees) Hook. f., Fl. Brit. India (J.D. Hooker). 7(22): 327. 1896 **

*Leptochloa wightiana* Nees, Syn. Pl. Glumac. (Steudel) 1: 209. 1854. Type: India; *Wight s.n.* as Dineba verticillata

*Eragrostis wightiana* (Nees) Benth., J. Linn. Soc., Bot. 19: 117. 1881

*Myriostachya wightiana* (Nees) Hook. f. var. *longispicula* Hook. f., Fl. Brit. India (J.D. Hooker). 7(22): 328. 1896. Syntypes: MalayPenang; *Curtis s.n.* Sri Lanka: Kottiyar; *Trimen s.n.*

*Myriostachya longispicula* (Hook. f.) Senaratna, Grasses of Ceylon (Perad. Man. No. 8) 66. 1956

**Distribution.** Andhra Pradesh, Madhya Pradesh, Odisha, Tamil Nadu, West Bengal [Sri Lanka].

**Habit.** Perennial.

**Remarks. **Soreng et al. (2017) place this taxon in Chloridoideae incertae sedis.

***Silentvalleya* V.J. Nair, Sreek., Vajr. & Bhargavan, J. Bombay Nat. Hist. Soc. 79: 654. 1983 (“1982”)**

**Type. ***Silentvalleya nairii* V.J. Nair, Sreek., Vajr. & Bhargavan.

**1252. **Silentvalleya chandwadensis**, Gosavi, B.R. Pawar & S.R. Yadav, Kew Bull. 67(3): 545. 2012 **

**Type.** India, Maharashtra, Nasik, Chandwad Ghat, 20°20'41.08"N, 74°15'01.07"E, 770 m alt., 10 Oct. 2010, *SUK-2997.*

**Distribution.** Maharashtra.

**Habit.** Perennial.

**1253. **Silentvalleya nairii** V. J. Nair, Sreek., Vajr. & Bhargavan, J. Bombay Nat. Hist. Soc. 79: 654–657. f. 1. 1983 (“1982”) **

**Distribution.** Kerala.

**Habit. **Perennial.

**Remarks. **Soreng et al. (2017) place this taxon in Chloridoideae incertae sedis.

### Tribe Centropodieae P.M. Peterson, N.P. Barker & H.P. Linder, 2011

Plants perennial, caespitose or rhizomatous. Lemma with 9 veins, with hairs tufted, in a transverse row, the apex with two deep lobes, awned from the sinus, the awn straight or geniculate.

***Centropodia* (R. Br.) Rchb., Consp. Regn. Veg. 212a. 1828**

**Lectotype. ***Centropodia forskaolii* (Vahl) Cope (“forskalii”) (*Avena forskaolii *Vahl (“forskålei”)).

**1254. **Centropodia forskaolii** (Vahl) Cope, Kew Bull. 37(4): 658. 1983 (“forskalii”) **

*Avena forskaolii* Vahl, Symb. Bot. (Vahl) 2: 25. 1791 (“forskålei”)

*Asthenatherum forskaolii* (Vahl) Nevski, Trudy Sredne-Aziatsk. Gosud. Univ., Ser. 8b, Bot. 17: 8. 1934

*Avena pensylvanica* Forssk., Fl. Aegypt.-Arab. 23. 1775, non L., 1753. Type: “In desertis Kahirinis”; *Forsskal s.n.* (C).

**Distribution.** Jammu and Kashmir [Pakistan].

**Habit.** Perennial.

### Tribe Triraphideae P.M. Peterson, 2010

Plants annual or perennial. Leaves with the ligule a fringe of hairs. Lemmas with pubescence on the lateral veins.

***Neyraudia* Hook. f., Fl. Brit. India (J.D. Hooker). 7: 305. 1896**

**Type. ***Neyraudia madagascariensis* Hook. f., nom. illeg. (*Arundo madagascariensis* Kunth, nom. illeg*.*; *Donax thuarii* P. Beauv.).

**1255. **Neyraudia arundinacea** (L.) Henrard, Meded. Rijks-Herb. 58: 8. 1929, in obs. **

*Aristida arundinacea* L., Mant. Pl. Altera 186. 1771. Type: “Habitat in India orientali. Koenig.” Lectotype: *Herb. Linn. No. 98.8 *(LINN). LT designated by Hubbard in Milne-Redhead and Polhill (ed.), Fl. Trop. E. Africa, Gramineae 1: 133. 1970.

*Arundo madagascariensis* Kunth, Révis. Gramin. 1: 273. t. 48. 1830. Type: “Crescit in Madagascaria.”

*Neyraudia madagascariensis* (Kunth) Hook. f., Fl. Brit. India (J.D. Hooker). 7(22): 305. 1896

**Distribution.** Kerala, Maharashtra, Punjab, Uttar Pradesh [China, Nepal, Pakistan].

**Habit.** Perennial.

**1256. **Neyraudia reynaudiana** (Kunth) Keng ex Hitchc., Amer. J. Bot. 21(3): 131. 1934 **

*Arundo reynaudiana* Kunth, Révis. Gramin. 1: 275. t. 49. 1830. Type: “Crescit in Pegu. Legit cel Reynaud.”

*Arundo zollingeri* Buse, Pl. Jungh. 343. 1854. Type: Indonesia: Java; *Zollinger 337, 380* (P).

*Neyraudia madagascariensis* (Kunth) Hook. f. var. *zollingeri* Hook. f., Fl. Brit. India (J.D. Hooker). 7(22): 305. 1896. Type: Sikkim Himalaya, Assam, Bengal, Khasia Hills, Chittagong, Tenasserim, Penang

*Phragmites zollingeri* Steud., Syn. Pl. Glumac. 1: 196. 1854. Type: Java; *Zollinger 337*

**Distribution.** Arunachal Pradesh, Assam, Meghalaya, Sikkim, West Bengal [Bangladesh, Bhutan, China, Myanmar, Nepal].

**Habit. **Perennial.

### Tribe Eragrostideae Stapf, 1898

Plants annual or perennial, rhizomatous, stoloniferous, or caespitose. Ligule generally a fringe of hairs but sometimes membranous. Spikelets with 1 to many flowers, the callus often pubescent. Lemma generally glabrous, with 3 veins, although up to 13 veins in a few species, the apex generally entire but occasionally mucronate or awned.

***Enneapogon* Desv. ex P. Beauv., Ess. Agrostogr. 81, 161. 1812**

**Lectotype. ***Enneapogon desvauxii* Desv. ex P. Beauv. LT designated by Hitchcock in U.S. Dept. Agric. Bull. 772: 83. 1920.

**1257. **Enneapogon cenchroides** (Licht.) C.E. Hubb., Bull. Misc. Inform. Kew 1934: 119. 1934 **

*Pappophorum cenchroides* Licht., Syst. Veg., ed. 15 bis [Roemer & Schultes] 2: 616. 1817. Type: “Habit in terra coranarum inter Haigarieb et Leeuwenkuil.” South Africa: near Vaal Rivier;* Lichtenstein s.n. *(B).

*Enneapogon mollis* Lehm., Nov. Stirp. Pug. 3: 40. 1831. Type: “Habitat in Promontorio Bonae Spei, in terrae tractu, qui vocatur Beaufort” South Africa; *Ecklon s.n.*

*Pappophorum robustum* Hook. f., Fl. Brit. India (J.D. Hooker). 7(22): 302. 1896. Type: India: Upper Gangetic plain, Hissar; *Drummond 3004, 3007* (K).

**Distribution.** Rajasthan, Punjab, Uttar Pradesh [Pakistan].

**Habit. **Tufted annual, occasionally with a few perennating shoots at the base.

**1258. **Enneapogon desvauxii** P. Beauv., Ess. Agrostogr. 82. t. 16 f. 11. 1812 **

*Pappophorum brachystachyum* Jaub. & Spach, Fl. Ill. pl. orient. 4:34, t. 324. 1851; Ann. Sci. Nat., Bot. Ser. 3. 14: 365. 1851 (“1850”)

*Enneapogon brachystachyus* (Jaub. & Spach) Stapf, Fl. Cap. (Harvey) 7: 654–655. 1900

**Lectotype. **P. Beauv., Ess. Agrost. t. 16. f. 11. 1812. LT designated (as type) by Ali Chaudahry, Grass. Saudi Arabia 221. 1989.

**Distribution.** Rajasthan [China, Pakistan].

**Habit.** Annual or perennial.

**1259. **Enneapogon persicus** Boiss., Diagn. Pl. Orient. ser. 1, 1(5): 71. 1844 **

*Pappophorum persicum* (Boiss.) Steud., Syn. Pl. Glumac. 1: 200. 1854

*Pappophorum aucheri* Jaub. & Spach, Ill. Pl. Orient. 4: 32. t. 323. 1851. Type: Persia; *Aucher-Eloy 5440* (error for 5430? vide Kew Bull. 22: 400. 1968) (P).

*Pappophorum turcomanicum* Trautv., Trudy Imp. S.-Peterburgsk. Bot. Sada 1: 27. 1871. Type: “Ind Turcomania, prope Krasnowodsk (Radde).” Russia: Turkmenistan; *Radde s.n.*

*Pappophorum schimperianum* Hochst. ex A. Rich., Tent. Fl. Abyss. 2: 403. 1850–1851. Type: “Crescit in collibus siccis prope Adoua, mense Septembre (Schmper).” Ehiopia: Adoua; *Schimper 323* (K).

*Enneapogon schimperanus* (Hochst. ex A. Rich.) Renvoize, Kew Bull. 22: 400. 1968

*Pappophorum elegans* Nees, Syn. Pl. Glumac. (Steudel) 1: 199. 1854

*Enneapogon elegans* (Nees) Stapf, Bull. Misc. Inform. Kew 1907: 224. 1907

**Type.** Persia; *Aucher-Eloy 5430* (P).

**Distribution.** Rajasthan [Afghanistan, China, Myanmar, Pakistan].

**Habit.** Perennial.

***Eragrostis* Wolf, Gen. Pl. (Wolf) 23. 1776**

**Lectotype. ***Eragrostis minor* Host (Icon. Descr. Gram. Austriac. 4: 15. 1809) (*Poa eragrostis* L.). LT designated by Pfeiffer, Nomencl. Bot. 1(2): 1226. 1874–1875.

**Notes.** Taxonomic editors for *Eragrostis*: Amanda Ingram, Wabash College; P. (Parigi) Venkateswara Prasanna and Alok R. Chorghe.

**1260. **Eragrostis aspera** (Jacq.) Nees, Fl. Afr. Austral. Ill. 408. 1841 **

*Poa aspera* Jacq., Hort. Bot. Vindob. 3: 32, t. 56. 1777. Type: India: “Cultivated in Europe with seed from India.” (W).

*Poa paniculata* Roxb., Fl. Ind. (Carey and Wallich ed.) 1: 341. 1820. Type: India; *Roxburgh s.n.*

*Eragrostis paniculata* (Roxb.) Steud., Syn. Pl. Glumac. 1: 266. 1854

*Eragrostis laxiflora* Schrad., Linnaea 12: 451. 1838, nom. nud. Type: *Drege 4285 *(LE).

**Distribution.** Andhra Pradesh, Karnataka, Madhya Pradesh, Maharashtra, Odisha, Rajasthan, Tamil Nadu [Malayasia, Sri Lanka, Africa].

**Habit. **Caespitose annual.

**1261. **Eragrostis atrovirens** (Desf.) Trin. ex Steud., Nomencl. Bot., ed. 2 (Steudel) 1: 562. 1840 **

*Poa atrovirens* Desf., Fl. Atlant. 1: 73. t. 14. 1798. Type: Algeria: Barbaria, “in arvis incultis prope La Calle”; *Desfontaines 160 *(FI).

*Poa biformis* Kunth, Révis. Gramin. 2: 471. t. 149. 1831. Type: “Crescit in Senegambia.”

*Eragrostis biformis* (Kunth) Benth., Niger Fl. 568. 1849

*Eragrostis bromoides* Jedwabn., Bot. Arch. 5(3–4): 190. 1924, non (Vahl) Steud., 1854. Type: Africa: Sudan Central, “In paludibus flum. Bailli”; *Chevalier no. 8588*

*Eragrostis atroviridis* Maire, Bull. Soc. Hist. Nat. Afrique N. 28(6): 385. 1937, nom. superfl. & illegit. for *Poa atrovirens*

**Distribution.** Andaman and Nicobar Islands, Andhra Pradesh, Assam, Bihar, Chhattisgarh, Jammu and Kashmir, Himachal Pradesh, Karnataka, Kerala, Madhya Pradesh, Maharashtra, Manipur, Meghalaya, Odisha, Punjab, Rajasthan, Sikkim, Tamil Nadu, Uttar Pradesh, Uttarakhand [China, Myanmar, Nepal, Sri Lanka, Africa].

**Habit.** Perennial.

**1262. **Eragrostis burmanica** Bor, Kew Bull. 6(2): 166. 1951 **

**Type.** Myanmar: Prome District, 20 July 1948, U. Thein Lwin 582 (K)

**Distribution. **Kerala.

**Habit.** Perennial.

**1263. **Eragrostis cilianensis** (All.) Vignolo ex Janch., Mitt. Naturwiss. Vereins Univ. Wien 5(9): 110. 1907 **

*Poa cilianensis* All., Fl. Pedem. 2: 246, t. 91, f. 2. 1785. Type: “In agro patrio Ciliani legit Cl. Bellardi.” Lectotype: Italy: Ciliani; *Bellardi s.n. *(TO-8242 (photo, K)). LT designated by Vignolo, Malpighia 18: 380. 1904.

*Briza eragrostis* L., Sp. Pl. 1: 70. 1753. Type: “Habitat in Europa australi ad agrorum versuras.” Lectotype: *Herb. Burser I: 10* (UPS). LT designated by Clayton in Polhill (ed.), Fl. Trop. E. Africa, Gramineae 2: 232. 1974.

*Eragrostis eragrostis* (L.) MacMill., Metasp. Minnesota Valley 75. 1892, nom. invalid.

*Megastachya eragrostis* (L.) P. Beauv. ex Roem. & Schult., Syst. Veg., ed. 15 bis [Roemer & Schultes] 2: 584. 1817

*Eragrostis costata* F. Turner, Proc. Linn. Soc. New South Wales 30: 91. 1905. Type: Australia: New South Wales, Breeza Plains, Werris Creek, Namoi River and near Tomworth; *Fred Turner s.n.*

*Eragrostis flexuosa* Steud., Syn. Pl. Glumac. 1: 266. 1854. Type: India; *Roxburgh Icon. 341*

*Eragrostis megastachya* (Koeler) Link, Hort. Berol. [Link] 1: 187. 1827

*Eragrostis megastachya* (Koeler) Link var. *cilianensis* (All.) Asch. & Graebn., Syn. Mitteleur. Fl. [Ascherson & Graebner]. 2(1): 371. 1900 [This combination is not made here.]

*Eragrostis minor* Host var. *major* Beck, Fl. Nieder-Osterreich 1: 88. 1890

*Eragrostis poaeoides* P. Beauv. ex Roem. & Schult. var. *megastachya* A. Gray, Manual (Gray), ed. 2. 563. 1856

*Eragrostis minor* Host var. *megastachya* (A. Gray) Burtt Davy, Fl. W. Calif. 60. 1901

*Eragrostis polymorpha* Roem. & Schult., Syst. Veg., ed. 15 bis [Roemer & Schultes] 2: 575. 1817

*Eragrostis schweinfurthiana* Jedwabn., Bot. Arch. 5: 194. 1924. Type: “Orae nubicae, in montibus Ssoturba, Gebel Schettoi; *Schweinfurth 1492*”

*Eragrostis starosselskyi* Grossh., Bot. Mater. Gerb. Glavn. Bot. Sada RSFSR 4: 18. 1923. Syntypes: TranscaucPr. Tiflis; July 20, 1921; *Karajazy s.n.* July 20, 1922; *V. Starosselsky s.n.* Turkestania: Buchara; *N. Troitzky s.n.*

*Eragrostis vulgaris* Coss. & Germ. var. *megastachya* Coss. & Germ., Fl. Env. Paris 2: 641. 1845

*Eragrostis vulgaris* Coss. & Germ. ssp. *major* Rouy, Fl. France (Rouy) 14: 262. 1913

*Eragrostis major* Host, Icon. Descr. Gram. Austriac. 4: 14. t. 24. 1809

*Erosion ciliare* Lunell, Amer. Midl. Naturalist 4: 221. 1915. Type: Based on *Poa cilianensis* All.

*Megastachya oblonga* (Moench) P. Beauv., Ess. Agrostogr. 167, 175. 1812

*Poa cachectica* Schumach., Kongel. Danske Vidensk. Selsk. Naturvidensk. Math. Afh. 3: 86. 1827. Type: Guinea; *Thonning s.n.*

*Poa eragrostis* (L.) Brot., Fl. Lusit. 1: 103. 1804, non L., 1753

*Poa roxburghiana* Schult., Mant. 2 (Schultes) 314. 1824. Type: India; *Roxburgh s.n.*

*Poa tortuosa* Spreng., Syst. Veg. (ed. 16) [Sprengel] 1: 345. 1824 (“1825”). Type: India orientalis

*Poa flexuosa* Roxb., Fl. Ind. (Carey and Wallich ed.) 1: 340. 1820, non Sm., 1800

*Poa megastachya* Koeler, Descr. Gram. (Koeler) 181. 1802, nom. superfl. & illeg. for *Brizaeragrostis*

*Poa oblonga* Baumg., Enum. Stirp. Transsilv. 3: 238. 1817, nom. superfl. & illeg. for *Brizaeragrostis*

*Poa polymorpha* J. Koenig ex Rottler, Ges. Naturf. Ferunde Berlin Neue Schriften 4: 194. 1803, non Wibel, 1799

*Briza oblonga* Moench, Methodus (Moench) 185. 1794, nom. superfl. & illeg. for *Briza eragrostis*

*Eragrostis articulata* De Wild., Bull. Jard. Bot. Etat 6: 58. t. 2. 1919, non (Schrank) Nees, 1829

*Briza megastachya* Hort. ex Roem. & Schult., Syst. Veg., ed. 15 bis [Roemer & Schultes] 2: 585. 1817, in syn.

**Distribution.** Andhra Pradesh, Bihar, Chhattisgarh, Delhi, Gujarat, Himachal Pradesh, Karnataka, Kerala, Madhya Pradesh, Maharashtra, Odisha, Punjab, Rajasthan, Tamil Nadu, Uttar Pradesh [China]. Naturalized; native to Europe.

**Habit.** Annual.

**1264. **Eragrostis ciliaris** (L.) R. Br., Observ. Congo 59. 1818; in Tuckey, Narr. Exped. Zaire 478. 1818 var. **ciliaris

*Poa ciliaris* L., Syst. Nat., ed. 10. 2: 875. 1759. Type: “Habitat [in Jamaica.] Sp. Pl., ed. 2. 1: 102. 1762” Lectotype: Browne, Herb. Linn. No. 87.66 (LINN). LT designated by Hitchcock in Contr. U. S. Natl. Herb. 12: 121. 1908.

*Cynodon ciliaris* (L.) Raspail, Ann. Sci. Nat. (Paris) 5: 302. 1825

*Megastachya ciliaris* (L.) P. Beauv., Ess. Agrostogr. 167, 174. 1812

*Poa boryana* Willd., Enum. Pl. (Willdenow) 1: 109. 1809. Type: “Habitat in insula Borboniae et Mauritii; *Bory do St. Vincent.*”

*Eragrostis boryana* (Willd.) Steud., Nomencl. Bot., ed. 2 (Steudel) 1: 562. 1840

*Megastachya boryana* (Willd.) Roem. & Schult, Syst. Veg., ed. 15 bis [Roemer & Schultes] 2: 592. 1817.

*Poa lepida* A. Rich., Tent. Fl. Abyss. 2: 424. 1850–1851. Type: “Crescit ad Pagum Ailet in Provincia Medat, mense Martio (Schimper).

*Eragrostis lepida* (A. Rich.) Hochst. ex Steud., Syn. Pl. Glumac. 1: 269. 1854.

*Eragrostis pulchella* Parl., Atti Congr. Sci. Ital. 8: 586. 1846. Type: “Hab. in insula S. jacobi, valle S. doninici (*J.D. Hooker no. 81*, November, 1839).”

*Poa amboinica* L., Mant. Pl. 557. 1771. Type: Habitat in India.

*Eragrostis lobata* Trin., Mém. Acad. Imp. Sci. St.-Pétersbourg, Sér. 6, Sci. Math. 1: 396. 1830, nom superfl. & illeg. for Poa cylindrica Roxb. Type: Africa: Mauritius Island, “V spp. Guin, Ins. Franc.”

*Eragrostis villosa* Trin., Fund. Agrost. (Trinius) 137. 1820, nom. superfl. & illeg. for *Poa ciliaris* L.

*Eragrostis ciliaris* sensu Stapf, Fl. Brit. India (J.D. Hooker). 7(22): 314. 1896 var. *ciliaris*, non (L) Link, 1827

**Distribution.** Andhra Pradesh, Bihar, Daman and Diu, Delhi, Goa, Gujarat, Karnataka, Kerala, Madhya Pradesh, Maharashtra, Odisha, Rajasthan, Tamil Nadu, Uttar Pradesh [China].

**Habit.** Annual.

**1265. **Eragrostis ciliaris** (L.) R. Br. var. **brachystachya** Boiss., Fl. Orient. (Boissier) 5: 582. 1884 **

**Type.** “Hab. in arenosis littoris Egyptiaco-Arabici inter Kosseir et Ras Benass; Schv. 1091. Belutschia; *J.E. Stocks s.n.*

**Distribution.** Punjab, Rajasthan [Egypt].

**Habit.** Annual.

**1266. **Eragrostis ciliaris** (L.) R. Br. var. **clarkei** Stapf ex Hook. f., Fl. Brit. India (J.D. Hooker). 7(22): 315. 1896 **

**Distribution.** Delhi, Rajasthan.

**Habit.** Annual.

**Remarks.** Taxonomic editor Peterson questioned which characters are used to recognize this taxon.

**1267. **Eragrostis ciliata** (Roxb.) Nees, Fl. Bras. Enum. Pl. 2(1): 512. 1829, in obs. (as “Eragrostin ciliatam”) [No reference to basionym] **

*Poa ciliata* Roxb., Fl. Ind. (Carey and Wallich ed.) 1: 336. 1820. Type: Generally found in poor dry soil.

*Megastachya rupestris* Roem. & Schult., Syst. Veg., ed. 15 bis [Roemer & Schultes] 2: 592. 1817. Type: India orientalis; *B. Heyne s.n.*

*Eragrostis rupestris* (Roem. & Schult.) Steud., Syn. Pl. Glumac. 1: 265. 1854

**Distribution.** Andhra Pradesh, Bihar, Chhattisgarh, Delhi, Gujarat, Jharkhand, Kerala, Madhya Pradesh, Maharashtra, Odisha, Tamil Nadu [China, Myanmar, Sri Lanka].

**Habit.** Perennial.

**1268. **Eragrostis coarctata** Stapf, Fl. Brit. India (J.D. Hooker). 7(22): 313. 1896 **

**Type.** India: Upper Gangetic Plain, Moradabad; *Thomson s.n.* Sikkim, Behar, Chittagong, Arracan and Myanmar. Central Provinces & Chota Nagpore; *Clarke s.n.*

**Distribution.** Andhra Pradesh, Assam, Bihar, Chhattisgarh, Jharkhand, Karnataka, Madhya Pradesh, Maharashtra, Odisha, Rajasthan, Sikkim, Tamil Nadu, Uttar Pradesh, Uttarakhand, West Bengal [Bangladesh, China, Myanmar, Nepal].

**Habit.** Perennial.

**1269. **Eragrostis collinensis** Vivek, G.V.S. Murthy & V.J. Nair, Indian J. Forest. 36(3): 401. 2013 **

**Type.** India: Tamil Nadu, Nilgiri District, Avalanche, 1925 m 14 October 1972, K. Vivekanandan 42951 (holo: CAL; iso: MH).

**Distribution.** Kerala and Tamil Nadu*.*

**Habit.** Annual.

**1270. **Eragrostis cumingii** Steud., Syn. Pl. Glumac. 1: 266. 1854 **

*Eragrostis distans* Hack., Publ. Bur. Sci. Gov. Lab. 35: 81. 1906 (“1905”). Type: Philippines: Kias, Benguet, Luzon; June, 1904; *Elmer 6608*

**Type.** Philippines; *Cuming Herb. no. 672 & 1104.*

**Distribution.** Kerala [China, Myanmar]. Naturalized; native to Australia.

**Habit.** Perennial.

**Remarks.** Record from Kerala fide Naithani (1990).

**1271. **Eragrostis curvula** (Schrad.) Nees, Fl. Afr. Austral. Ill. 397. 1841 **

*Poa curvula* Schrad., Gött. Gel. Anz. 3: 2073. 1821. Type: South Africa: Cape Province, Cape of Good Hope; *Hesse s.n. in Herb. Trinius 2327.1 (2327.3?)* (holo PH?; LE, iso, microfiche IDC BT-16/1).

**Distribution.** Madhya Pradesh, Rajasthan, Tamil Nadu [China, Pakistan]. Naturalized; native to southern Africa.

**Habit.** Perennial.

**1272. **Eragrostis deccanensis** Bor, Grasses Burma, Ceylon, India & Pakistan 507. 1960 **

*Eragrostis phleoides* Stapf, Fl. Brit. India (J.D. Hooker). 7(22): 313. 1896, non Hillebr., 1888

*Eragrostis spicata* (Heyne ex Stapf) Jedwabn., Bot. Arch. 5: 185. 1924, non Vasey, 1891

**Type.** India: Madras; *Narayanaswami 3027*. Madras; Numer. List [Wallich] no. 5015.

**Distribution.** Andhra Pradesh, Karnataka, Kerala, Odisha, Tamil Nadu.

**Habit.** Perennial.

**1273. **Eragrostis diplachnoides** Steud., Syn. Pl. Glumac. 1: 268. 1854 **

*Eragrostis interrupta* P. Beauv. var. *diplachnoides* (Steud.) Stapf, Fl. Brit. India (J.D. Hooker). 7(22): 316. 1896

**Type. **Egypt: Nubia; *Hochstetter Herb. no. 346.*

**Distribution.** Karnataka, Tamil Nadu [Bangladesh, Sri Lanka].

**Habit.** Annual.

**1274. **Eragrostis ferruginea** (Thunb.) P. Beauv., Ess. Agrostogr. 71, 162, 174. 1812 **

*Poa ferruginea* Thunb., in J. A. Murray, Syst. veg. ed. 14: 114. 1784. Type: Japan; *Thunberg s.n.* (UPS).

**Distribution.** Sikkim [Bhutan, China, Nepal].

**Habit.** Perennial.

**1275. **Eragrostis gangetica** (Roxb.) Steud., Syn. Pl. Glumac. 1: 266. 1854 **

*Poa gangetica* Roxb., Fl. Ind. (Carey and Wallich ed.) 1: 341. 1820. Type: India: on the banks of the Ganges; *Roxburgh s.n.*

*Eragrostis stenophylla* Hochst. ex Miq., Nieuwe Verh. Eerste Kl. Kon. Ned. Inst. Wetensch. Amsterdam ser. 3, 4: 39. 1851, p.p. Type: India: Mangalor; *Metz no. 664*

**Distribution.** Andhra Pradesh, Arunachal Pradesh, Assam, Bihar, Chhattisgarh, Daman and Diu, Goa, Himachal Pradesh, Jharkhand, Karnataka, Kerala, Madhya Pradesh, Maharashtra, Manipur, Meghalaya, Odisha, Rajasthan, Tamil Nadu, Tripura, Uttar Pradesh, Uttarakhand, West Bengal [Myanmar, Sri Lanka].

**Habit.** Annual.

**1276. **Eragrostis henryi** Vivek, G.V.S. Murthy & V.J. Nair, Nelumbo 54: 10. 2012. **

**Type.** India: Tamil Nadu, Kanyakumari district, Near Anjugramam, 25 m a.m.s.l., 27 March 1979, *A. N. Henry 6156* (holotype CAL, isotypes: MH.

**Distribution. **Tamil Nadu (Kanyakumari).

**Habit.** Not reported.

**1277. **Eragrostis jainii** Vivek, G.V.S. Murthy & V. J. Nair in Nelumbo55: 1. 2013. **

**Type.** India: Kerala, Kannur District, Manjeshwar, 100 m, 13 October 1979, *R. Ansari 64888* (holotype: CAL, isotype: MH).

**Distribution.** Kerala and Karnataka.

**Habit.** Annual or short-lived perennial.

**1278. **Eragrostis japonica** (Thunb.) Trin., Mém. Acad. Imp. Sci. St.-Pétersbourg, Sér. 6, Sci. Math. 1: 405. 1830 **

*Poa japonica* Thunb., in J. A. Murray, Syst. veg. ed. 14: 114. 1784. Type: Japan: Kosido, near Nagasaki; *Thunberg s.n. *(UPS).

*Poa diarrhena* Schult. & Schult. f., Mant. 3 (Schultes & Schultes f.) Add. i Mant. cl. iii: 616. 1827. Type: ‘Mant. II. p. 315. n. 100 a.’ ‘In Bengalia’.

*Eragrostis diarrhena* (Schult. & Schult. f) Steud., Syn. Pl. Glumac. 1: 266. 1854. Type: India: Bengal

*Eragrostis interrupta* P. Beauv. var. *tenuissima* Stapf, Fl. Brit. India (J.D. Hooker). 7(22): 316. 1896

*Poa tenellula* Kunth, Révis. Gramin. 1: 113. 1829. Type: India orientalis.

*Eragrostis tenellula* (Kunth) Steud., Syn. Pl. Glumac. 1: 279. 1854

*Poa tenella* sensu R. Br., Prodr. Fl. Nov. Holland. 1: 181. 1810, non L., 1753

*Eragrostis tenuissima* Schrad. ex Nees, Fl. Afr. Austral. Ill. 409. 1841, nom. nud.

**Distribution.** Andhra Pradesh, Assam, Bihar, Chhattisgarh, Gujarat, Himachal Pradesh, Jammu and Kashmir, Karnataka, Kerala, Madhya Pradesh, Maharashtra, Nagaland, Odisha, Punjab, Rajasthan, Sikkim, Tamil Nadu, Uttar Pradesh, Uttarakhand, West Bengal [Bhutan, China, Myanmar, Nepal, Pakistan, Sri Lanka].

**Habit.** Annual or short-lived perennial.

**1279. **Eragrostis lehmanniana** Nees, Fl. Afr. Austral. Ill. 402. 1841 var. **lehmanniana

**Type. **“Ad rupes nudas prope Slengerfontein in collibus ad Nieuwe Hantom, alt. 4000–5000 ft.; Feb; *Drège s.n.*”.

**Distribution.** India; states not recorded [also in Africa, Mexico]. Introduced but not truly naturalized; native to southern Africa.

**Habit.** Perennial.

**1280. **Eragrostis lehmanniana** Nees var. **chaunantha** (Pilg.) de Winter, Grass. Pastures South Africa 147. 1955 **

*Eragrostis chaunantha* Pilg., Bot. Jahrb. Syst. 40: 84. 1907. Type: Africa: Kalahari “Zwischen Kokong und Kang”; December 1904; *Schultze 290*

**Distribution.** India; states not recorded [also in Africa]. Introduced but not truly naturalized; native to southern Africa.

**Habit.** Perennial.

**1281. **Eragrostis macilenta** (A. Rich.) Steud., Syn. Pl. Glumac. 1: 268. 1854 **

*Poa macilenta* A. Rich., Tent. Fl. Abyss. 2: 428. 1850–1851. Type: Abyssinie: Trigre, Adua; *Quartin-Dillon et Petit s.n.* (P).

**Distribution. **Tamil Nadu [also in the Middle East, Africa].

**Habit.** Annual.

**Remarks.** Record from Tamil Nadu fide Naithani (1990).

**1282. **Eragrostis maderaspatana** Bor, Grasses Burma, Ceylon, India & Pakistan 509. 1960 **

*Eragrostis willdenoviana* Nees ex Hook. f., Fl. Brit. India (J.D. Hooker). 7(22): 322. 1896, non Nees ex Hook. & Arn., Bot. Beechey Voy. 252. 1838. Syntypes: India: Mysore; *Rottler s.n., Heyne s.n.* Dindygul; *Wight s.n.* Sri Lanka; Trincomalee; *Glemie Ceylon Plant 3944*

**Distribution.** Andhra Pradesh, Karnataka, Kerala, Odisha, Tamil Nadu [Sri Lanka].

**Habit.** Annual.

**1283. **Eragrostis minor** Host, Icon. Descr. Gram. Austriac. 4: 15. 1809) (as “Eragrostin minorem”) **

*Poa eragrostis* L., Sp. Pl. 1: 68. 1753. Type: “Habitat in Italia supra muros. D. Baeck.” Lectotype: Baeck 7, Herb. Linn. No. 87.23 (LINN). LT designated by Clayton in Polhill (ed.), Fl. Trop. E. Africa, Gramineae 2: 234. 1974.

*Eragrostis poaeoides* P. Beauv., Ess. Agrostogr. 162 1812, nom. nud.

*Eragrostis multiflora* (Roxb.) Trin. var. *pappiana *Chiov., Ann. Bot. (Rome) 8(1): 65. 1903. Syntypes: Africa: Ethiopia, Samhar, Saati; March 8, 1892; *Terracciano & Pappi 2868, 2869* (P). Emberemi; March 28, 1892; *Pappi nn. 1190 & 1566*

*Eragrostis pappiana* (Chiov.) Chiov., Ann. Bot. (Rome) 8(3): 371. 1908

**Distribution.** Andhra Pradesh, Bihar, Chhattisgarh, Gujarat, Himachal Pradesh, Jharkhand, Karnataka, Kerala, Madhya Pradesh, Maharashtra, Odisha, Punjab, Rajasthan, Tamil Nadu, Uttarakhand, West Bengal [China]. Naturalized; native to Europe.

**Habit.** Annual.

**Remarks. **Records from Madhya Pradesh, Maharashtra, and Rajasthan fide Naithani (1990).

**1284. **Eragrostis multicaulis** Steud., Syn. Pl. Glumac. 1: 426. 1854 **

*Eragrostis damiensiana* (Bonnet) Thell., Repert. Spec. Nov. Regni Veg. 24: 323. 1928

*Eragrostis pilosa* (L.) P. Beauv. var. *damiensiana* Bonnet, Naturaliste 3: 412. 1881. Type: “France: Paris: dans la cour du ministere de la Guerre”; *M. Damiens s.n.*

*Eragrostis peregrina* Wiegand, Rhodora 19: 95. 1917, nom. & stat. nov.

*Eragrostis pilosa* (L.) P. Beauv. var. *condensata* Hack., Allg. Bot. Z. Syst. 7: 13. 1902 (“1901”). Type: Germany: Karlsurhe, Baden-Wurttemberg, alt. 400 ft.; July 17, 1900; *A. Kneucker no. 115 *(BAA-1072).

*Glyceria airoides* Steud., Syn. Pl. Glumac. 1: 287, 426. 1854, non (Koeler) Rchb., 1829 [Name emended]

**Lectotype.** Japan; *Burger s.n.* (L-908.97-2116). LT designated by Veldkamp in Blumea 47(1): 181. 2002.

**Distribution.** Karnataka, Maharashtra, Rajasthan, Sikkim, Uttarakhand [China].

**Habit.** Annual.

**1285. **Eragrostis nigra** Nees, Syn. Pl. Glumac. (Steudel) 1: 267. 1854 **

*Eragrostis atropurpurea* Hochst. ex Steud., Syn. Pl. Glumac. 1: 267. 1854. Type: India; *R. F. Hohenacker Herb. no. 938*

*Eragrostis paniculata* sensu Thwaites, Enum. Pl. Zeyl. (Thwaites). 373. 1864, non (Roxb.) Steud., 1854

**Type.** Peninsular India.

**Distribution.** Andhra Pradesh, Assam, Bihar, Himachal Pradesh, Jammu and Kashmir, Karnataka, Kerala, Maharashtra, Manipur, Meghalaya, Nagaland, Odisha, Rajasthan, Sikkim, Tamil Nadu, Uttar Pradesh, Uttarakhand, West Bengal [Bhutan, China, Myanmar, Nepal, Sri Lanka].

**Habit.** Perennial.

**1286. **Eragrostis nilgiriensis** Vivek., Murthy & V.J. Nair, Nordic Journal of Botany 31(6): 700. 2013 **

**Type.** India. Tamil Nadu, Nilgiri district, Coonoor (10o20’37.13” N, 76o47’40.98” E) 1700 m asl., 22 Oct 2011, *C. P Vivek 126153* (CAL). Isotypes: MH.

**Distribution.** Tamil Nadu.

**Habit.** Not recorded.

**1287. **Eragrostis nutans** (Retz.) Nees ex Steud., Nomencl. Bot., ed. 2 (Steudel) 1: 563. 1840 **

*Poa nutans* Retz., Observ. Bot. (Retzius) 4: 19. 1786–1787. Type: “E Tranquebaria misit cel Koenig, ubi ad margines agrorum et in agris oryza ceis minus submerses sat frequens esse scripsit.” India: Tranquebaria; *Koenig s.n. *(BM).

*Poa chariis* Schult., Mant. 2 (Schultes) 314. 1824. Type: India; *Roxburgh s.n.*

*Eragrostis chariis* (Schult.) Hitchc., Lingnan Sci. J. 7: 193. 1931

*Poa elegantula* Kunth, Révis. Gramin. 1: 114. 1829

*Eragrostis elegantula* (Kunth) Nees, Syn. Pl. Glumac. (Steudel) 1: 266. 1854

*Eragrostis stenophylla* sensu Hook. f., Fl. Brit. India (J.D. Hooker). 7(22): 318. 1896, p.p., non Hochst. Ex Miq., 1851

*Poa elegans* Roxb., Fl. Ind. (Carey and Wallich ed.) 1: 339. 1820, non Poir., 1804

**Distribution.** Andhra Pradesh, Bihar, Chhattisgarh, Gujarat, Himachal Pradesh, Karnataka, Kerala, Madhya Pradesh, Maharashtra, Odisha, Rajasthan, Tamil Nadu, Uttar Pradesh, West Bengal [China, Sri Lanka].

**Habit.** Perennial.

**1288. **Eragrostis paniciformis** (A. Braun) Steud., Syn. Pl. Glumac. 1: 268. 1854. **

*Poa paniciformis *A. Braun, Flora 24: 274, 1841. Type: Ethiopia: 1841, *A. Braun s. n*. (lectotype K).

**Distribution.** Kerala.

**Habit.** Perennial.

**1289. **Eragrostis papposa** (Roem. & Schult.) Duf. ex Steud., Nomencl. Bot., ed. 2 (Steudel) 1: 564. 1840 **

*Megastachya papposa* Roem. & Schult., Syst. Veg., ed. 15 bis [Roemer & Schultes] 2: 585. 1817. Type: “In monte Saguntino regni Valentiae Confer Scheuchz. Gram p. 192. Dufour.” Spain: Sagunto; *Dufour s.n. *(NTM).

*Eragrostis speirostachya* Coss. & Dur., Vidensk. Meddel. Dansk Naturhist. Foren. Kjobenhavn 47. 1860. Type: not given.

*Poa papposa* Duf. ex Roem. & Schult., Syst. Veg., ed. 15 bis [Roemer & Schultes] 2: 585. 1817, nom. nud., pro. syn.

*Eragrostis verticillata* sensu Coss. ex Asch. & Graebn., Syn. Mitteleur. Fl. [Ascherson & Graebner]. 2(1): 370. 1900, non (Cav.) P. Beauv., 1812

*Eragrostis rigidifolia* Hochst. ex Engl., Abh. Königl. Akad. Wiss. Berlin 1891: 134. 1892, nom. nud.

**Distribution.** Andhra Pradesh, Punjab, Rajasthan [Pakistan].

**Habit. **Annual to short lived-perennial.

**1290. **Eragrostis pilosa** (L.) P. Beauv., Ess. Agrostogr. 175. 1812 **

*Poa pilosa* L., Sp. Pl. 1: 68. 1753. Type: “Habitat in Italia.” Lectotype = “Gramen paniculis elegantissimis, majus, locustis purpureo-spadiceis, minoribus” in Scheuchzer, Agrostographia, 193. t. 4. f. 3. 1719. LT designated by Du Puy et al. in George et al. (ed.), Fl. Australia 50: 472. 1992. Epitype: Italy. an Wegen zwischen den Reisfeldern von Oldenico unweit Vercelli in Oberitalien; August 1–10, 1902; *A. Kneucker, Gram. Exsicc. XII, No. 344* (B). Epitype designated by Scholz in Cafferty et al. (ed.), Taxon 49: 256. 2000.

*Eragrostis filiformis* Link, Hort. Berol. [Link] 1: 191. 1827. Type: “Habitat in America boreali.”

*Poa linkii* Kunth, Révis. Gramin. 1: 113. 1829

*Eragrostis linkii* (Kunth) Steud., Syn. Pl. Glumac. 1: 273–274. 1854

*Eragrostis tenuiflora* Rupr. ex Steud., Syn. Pl. Glumac. 1: 268. 1854. Type: “Sennaar Nubiae. Hrbr. Fl. aeth. Kotsch nr. 185”

*Poa senegalensis* Desv., Opusc. Sci. Phys. Nat. 100. 1831, Hort.Par. ex Desf., 1829 (vide IPNI). Type: “Habitat in Africa.”

*Eragrostis senegalensis* sensu Chev., Rev. Bot. Appl. Agric. Trop. 14: 120. 1934, non Nees, 1834

**Distribution.** Andhra Pradesh, Arunachal Pradesh, Bihar, Chhattisgarh, Delhi, Goa, Gujarat, Himachal Pradesh, Jammu and Kashmir, Jharkhand, Karnataka, Kerala, Madhya Pradesh, Maharashtra, Odisha, Punjab, Rajasthan, Sikkim, Tamil Nadu, Uttar Pradesh, Uttarakhand [China, Myanmar, Pakistan, Sri Lanka].

**Habit.** Annual.

**1291. **Eragrostis plana** Nees, Fl. Afr. Austral. Ill. 390. 1841 **

**Type.** “Ad flumen prope Kachu et Zandplaat alt. 1500 ft., inter Geckau et Basche alt. 1500–2000.” *Drege s.n.*

**Distribution. **India [China]. Naturalized; native to southern Africa.

**Habit.** Perennial.

**Remarks. **The report of this species for India is dubious; documentation is lacking.

**1292. **Eragrostis riparia** (Willd.) Nees, Fl. Bras. Enum. Pl. 2(1): 512. 1829 **

*Poa riparia* Willd., Ges. Naturf. Ferunde Berlin Neue Schriften 4: 185. 1803. Type: India: Madras

*Megastachya riparia* (Willd.) Roem. & Schult., Syst. Veg., ed. 15 bis [Roemer & Schultes] 2: 593. 1817

*Eragrostis tenella* (L.) P. Beauv. var. *riparia* (Willd.) Stapf, Fl. Brit. India (J.D. Hooker). 7(22): 315. 1896

*Eragrostis plumosa* (Retz.) Link var. *maritima* Thwaites ex Trimen, Syst. Cat. Fl. Pl. Ceylon 109. 1885, nom. nud. (cited C.P. 3944 only)

*Poa amboinica* sensu Retz., Observ. Bot. (Retzius) 4: 20. 1786–1787, non L., 1771

**Distribution.** Andhra Pradesh, Assam, Bihar, Chhattisgarh, Karnataka, Kerala, Maharashtra, Odisha, Rajasthan, Sikkim, Tamil Nadu, Uttar Pradesh, West Bengal [Myanmar, Sri Lanka].

**Habit.** Annual or short-lived perennial.

**1293. **Eragrostis robusta** Stent, Bothalia 2(1b): 288. 1927 **

*Eragrostis valida* Stent, Bothalia 1(3): 172. 1923, non Pilg., 1916. Type: Maritzburg: Natal, Angus 2 (Govt. Herb. 13862)

**Type.** ‘The Maritzburg, Natal Angus 2’ (Govt. Herb. 13862.).

**Distribution.** India [also in Africa]. Naturalized; native to southern Africa.

**Habit.** Perennial.

**Remarks.** The report of this species for India is dubious; documentation is lacking. Taxonomic editor Ingram notes that this taxon is not included by Clayton et al. (2006 onwards), suggesting that it should probably be placed in synonymy.

**1294. **Eragrostis rottleri** Stapf, Fl. Brit. India (J.D. Hooker). 7(22): 321. 1896 **

**Syntypes.** The Carnatio, Tranquebar; *Heyne s.n.*, *Rottler s.n.* as *Poa pauciflora* & *Eragrostisviscosa*; *Wight s.n.*

**Distribution.** Kerala, Tamil Nadu [Pakistan].

**Habit.** Annual.

**1295. **Eragrostis santapaui** K.G. Bhat & Nagendran, Reinwardtia 10(2): 127, f. 1. 1985 **

**Type. **India: Karnataka State, Coorg District, Mercara, growing in moist soil; December 18, 1980; *K.G. Bhat 794 A* (CAL).

**Distribution.** Karnataka.

**Habit. **Annual.

**1296. **Eragrostis schweinfurthii** Chiov., Annuario Reale Ist. Bot. Roma 8(3): 368–369. 1908 var. **schweinfurthii

**Syntypes.** Eritrea: Ocule Cusai, bosco dell’Assare presso Halai, alt. 8000 ft.; September 30, 1902; *Pappi 1961 *(FT). Eritrea: gruppo dei monti Soyrá, mt. Mamahot verso il torrente Arigot, 8500–9200 ft.; August 23,1902; *Pappi 1245.*

**Distribution. **Tamil Nadu [Sri Lanka].

**Habit.** Annual or short-lived perennial.

**1297. **Eragrostis schweinfurthii** Chiov. var. **kiwuensis** (Jedwabn.) S. M. Phillips, Kew Bull. 42(4): 930. 1987 **

*Eragrostis kiwuensis* Jedwabn., Bot. Arch. 5: 206. 1924. Type: “Africa orientalis tropi., Kiwu, ad Karisimbi”; *Mildbraed 1784*

**Distribution.** Tamil Nadu [also in the Middle East, Africa].

**Habit.** Not reported.

**1298. **Eragrostis subsecunda** (Lam.) E.Fourn., Mexic. Pl. 2: 118. 1886 **

*Poa subsecunda* Lam., Tabl. Encycl. 1: 184. 1791. Type: China.

**Distribution.** Assam, Kerala, Tamil Nadu [Bangladesh, China, Myanmar, Sri Lanka].

**Habit.** Perennial.

**1299. **Eragrostis superba** Peyr., Sitzungsber. Kaiserl. Akad. Wiss., Math.-Naturwiss. Cl. 38: 584. 1860 **

**Type.** Africa: Angola, “in locis umbrosis prope Benguela ac Loanda”; *H. Wawra 244* (W).

**Distribution. **Karnataka, Tamil Nadu [Pakistan]. Naturalized; native to tropical Africa.

**Habit.** Perennial.

**1300. **Eragrostis tef** (Zucc.) Trotter, Boll. Soc. Bot. Ital. 1918(1): 62. 1918, in obs. **

*Poa tef* Zucc., Diss. Ditef [unpaged]. 1775. Type: “cultivated at botanical garden in Florence, Italy from seed collected in “Abyssinia”; naturalized in the New World (Brazil), Ethiopia (Northeast Tropical Africa, Africa) “ (FI).

*Poa abyssinica* Jacq., Misc. Austriac. 2: 364. 1781. Type: Abyssinia.

*Eragrostis abyssinica* (Jacq.) Link, Hort. Berol. [Link] 1: 192. 1827 (as “abessinica”)

**Distribution. **Kerala, Madhya Pradesh, Maharashtra, Rajasthan, Tamil Nadu, Uttar Pradesh, Uttarakhand [also in Saudi Arabia, Africa, Europe, North America, South America]. Naturalized; native to tropical East Africa.

**Habit.** Annual.

**Remarks.** Records from Madhya Pradesh, Rajasthan and Tamil Nadu fide Naithani (1990).

**1301. **Eragrostis tenella** (L.) P. Beauv., Syst. Veg., ed. 15 bis [Roemer & Schultes] 2: 576. 1817 var. **tenella

*Poa tenella* L., Sp. Pl. 1: 69. 1753. Type: “Habitat in India.” Lectotype: *Herb. Linn. No. 87.33* (LINN). LT designated by Mitra & Jain in Manilal (ed.), Bot. Hist. Hort. Malabaricus 151. 1980.

*Megastachya tenella* (L.) Bojer, Hortus Maurit. 369. 1837

*Poa amabilis* L., Sp. Pl. 1: 68. 1753. Type: “Habitat in India.” Lectotype: *Herb. Hermann 2: 59, No. 46* (BM-000621703). LT designated by Veldkamp in Cafferty et al. (ed.), Taxon 49: 254. 2000.

*Eragrostis amabilis* (L.) Wight & Arn., Cat. Indian Pl. [pt. 2]: 105, no. 1777. 1834

*Poa plumosa* Retz., Observ. Bot. (Retzius) 4: 20. 1786–1787. Type: “E. Tranquebaria misit amicis Koenig.” India: Tranquebaria; *Koenig in herb. Retzius 378* (LD).

*Eragrostis plumosa* (Retz.) Link, Hort. Berol. [Link] 1: 192. 1827

*Eragrostis tenella* (L.) P. Beauv. var. *plumosa* (Retz.) Stapf, Fl. Brit. India (J.D. Hooker). 7(22): 315. 1896

**Distribution.** Andaman and Nicobar Islands, Andhra Pradesh, Bihar, Chhattisgarh, Daman and Diu, Goa, Gujarat, Himachal Pradesh, Jharkhand, Karnataka, Kerala, Lakshadweep, Madhya Pradesh, Maharashtra, Odisha, Rajasthan, Sikkim, Tamil Nadu, Tripura, Uttar Pradesh, Uttarakhand, West Bengal [Sri Lanka, Myanmar].

**Habit.** Annual.

**Remarks.** Taxonomic editor Ingram notes that the name *Eragrostis amabilis* is used fairly commonly for this taxon, but *E. tenella* is the correct name. See also Peterson et al. (2018), who also suggest using *E. tenella*.

**1302. **Eragrostis tenella** (L.) P. Beauv. var. **breviculmis** Stapf, Fl. Brit. India (J.D. Hooker). 7(22): 316. 1896 **

**Type.** India: Punjab, Behar.

**Distribution. **Bihar, Jharkhand, Odisha, Maharashtra, Punjab [Sri Lanka].

**Habit. **Annual.

**1303. **Eragrostis tenella** (L.) P. Beauv. var. **insularis** C.E. Hubb., Bull. Misc. Inform. Kew 1939: 654. 1939 **

**Type.** Mauritius: Moka; *Vaughan 1937. *

**Distribution.** Kerala, Maharashtra, Rajasthan, Tamil Nadu, West Bengal [Sri Lanka, Mauritius, Bangladesh].

**Habit. **Annual.

**Remarks.** Records for Maharashtra and Rajasthan fide Naithani (1990).

**1304. **Eragrostis tenuifolia** (A. Rich.) Hochst. ex Steud., Syn. Pl. Glumac. 1: 268. 1854 **

*Poa tenuifolia* A. Rich., Tent. Fl. Abyss. 2: 425. 1850–1851. Type: “Crescit in montosis provinciae Chire mense Octobre (Quartin Dillon), et in locis incultis valium prope Adoua mense Septembre (Schimper). Lectotype: Ethiopia: Adoam; January 1837 [September 18, 1837]; *Schimper 92* (P). LT designated by S. Phillips in Fl. Ethiopia 7: 122. 1995.

*Eragrostis parviglumis* Hochst. ex Steud., Syn. Pl. Glumac. 1: 267. 1854. Type: India: Nilagiri; *Hochstetter no. 936*

*Eragrostis collocarpa* K. Schum in Engler, Pflanzenw. Ost-Afrikas C: 114. 1895. Type. “Holst n. 54, 3228, 8923). – Auf trockenen Grasplatzen und im gelichteten Hochwalde.”

**Distribution.** Andhra Pradesh, Bihar, Daman and Diu, Gujarat, Karnataka, Kerala, Madhya Pradesh, Maharashtra, Odisha, Rajasthan, Tamil Nadu, Uttar Pradesh [Myanmar].

**Habit.** Perennial.

**1305. **Eragrostis tremula** Hochst. ex Steud., Syn. Pl. Glumac. 1: 269. 1854 **

*Poa tremula* Lam., Tabl. Encycl. 1: 185. 1791. Type: “E. Senegal, D. Roussillon. “Africa: Senegal; *Roussillon s.n. *(P-LAM).

*Eragrostis rhachitricha* Hochst. ex Miq., Nieuwe Verh. Eerste Kl. Kon. Ned. Inst. Wetensch. Amsterdam ser. 3, 4: 37. 1851. Type: India: Manglore; *Metz no. 280*

*Poa multiflora* Roxb., Fl. Ind. (Carey and Wallich ed.) 1: 340. 1820, non Forssk., 1775. Type: India: “It is found on dry elevated places.”

*Eragrostis multiflora* Trin., Mém. Acad. Imp. Sci. St.-Pétersbourg, Sér. 6, Sci. Math. 1(4): 401. 1830

*Eragrostis lamarckii* Steud., Syn. Pl. Glumac. 1: 269. 1854, nom. nov. for *Poa tremula*, non *E. tremula*.

**Type.** Nubia; *HochstetterHrbr. un. it. no. 6.*

**Distribution.** Andhra Pradesh, Bihar, Chhattisgarh, Delhi, Goa, Gujarat, Himachal Pradesh, Jharkhand, Karnataka, Kerala, Madhya Pradesh, Maharashtra, Manipur, Odisha, Punjab, Rajasthan, Tamil Nadu, Uttar Pradesh, West Bengal [Afghanistan, China, Myanmar, Sri Lanka].

**Habit.** Annual.

**1306. **Eragrostis trichodes** (Nutt.) Alph. Wood, Class-book Bot. (ed. 1861). 796. 1861 **

*Poa trichodes* Nutt., Trans. Amer. Philos. Soc., ser. 2, 5: 146. 1835. Type: USA: Arkansas Territory, in brushy prairies and open alluvial lands; *Nuttall s.n.* (PH).

*Eragrostis capillacea* Jedwabn., Bot. Arch. 5: 196. 1924. Type: “Americae sept. Civit. Unitae, Nebraska centr., ad Plumer Ford, Dismal River, Thomas Co.; *Rydberg 1832*”

*Eragrostis tenuis* A. Gray, Manual (Gray), ed. 6. 661. 1890, non Steud., 1854

**Distribution.** India [also in North America]. Naturalized; native to North America.

**Habit.** Perennial.

**Remarks.** The report of this species for India is dubious; documentation is lacking.

**1307. **Eragrostis unioloides** (Retz.) Nees, Syn. Pl. Glumac. (Steudel) 1: 264. 1854 var. **unioloides

*Poa unioloides* Retz., Observ. Bot. (Retzius) 5: 19. 1788 (“1789”). Type: “Communicavit Honor Koenig.” India; *Koenig s.n.* (LD).

*Poa rubens* Lam., Tabl. Encycl. 1: 184. t. 45. f. 2. 1791. Type: “Ex India. Sonner. An. P. Amabilis.” India: Mangalore; *Sonnerat s.n.* (P-LAM?).

*Eragrostis rubens* (Lam.) Hochst. ex Miq., Nieuwe Verh. Eerste Kl. Kon. Ned. Inst. Wetensch. Amsterdam ser. 3, 4: 38. 1851

*Eragrostis amabilis* sensu Stapf, Fl. Brit. India (J.D. Hooker). 7(22): 317. 1896, non Wight & Arn. ex Nees, 1838 (excluding syn *Poa amabilis* L. Sp. Pl. 1: 68. 1753)

**Distribution.** Andaman and Nicobar Islands, Andhra Pradesh, Arunachal Pradesh, Assam, Bihar, Chhattisgarh, Dadra and Nagar Haveli, Goa, Gujarat, Himachal Pradesh, Karnataka, Kerala, Madhya Pradesh, Maharashtra, Meghalaya, Odisha, Sikkim, Tamil Nadu, Tripura, Uttar Pradesh, Uttarakhand, West Bengal [China, Myanmar, Nepal, Pakistan, Sri Lanka].

**Habit. **Annual or short-lived perennial.

**1308. **Eragrostis unioloides** (Retz.) Nees var. **tremula** Jacob, J. Bombay Nat. Hist. Soc. 47: 51. 1947 **

**Type.** India: Travancore, Tiruvalla, alt. 100 ft.; *K.C. Jacob s.n.* (MH-86288).

**Distribution.** Kerala, Tamil Nadu.

**Habit.** Annual or perennial.

**Remarks.** Record for Kerala fide Naithani (1990).

**1309. **Eragrostis viscosa** (Retz.) Trin., Mém. Acad. Imp. Sci. St.-Pétersbourg, Sér. 6, Sci. Math. 1(4): 397. 1830 **

*Poa viscosa* Retz., Observ. Bot. (Retzius) 4: 20. 1786–1787. Type: “In glareosis aridis Malabariae minus frequens tempore calido viget. Cl. Koening.” India: Malabar; *Koenig s.n.* (LD).

*Eragrostis mangalorica* Hochst. ex Miq., Nieuwe Verh. Eerste Kl. Kon. Ned. Inst. Wetensch. Amsterdam ser. 3, 4: 38. 1851. Type: India, Karnataka, Mangalore; *R.F. Hohenacker Herb. Metz no. 262*

*Eragrostis tenella* (L.) P. Beauv. var. *viscosa* (Retz.) Stapf, Fl. Brit. India (J.D. Hooker). 7(22): 315. 1896

**Distribution.** Andhra Pradesh, Assam, Bihar, Chhattisgarh, Daman and Diu, Dadra and Nagar Haveli, Delhi, Goa, Gujarat, Himachal Pradesh, Karnataka, Kerala, Madhya Pradesh, Maharashtra, Odisha, Rajasthan, Tamil Nadu, Uttar Pradesh, West Bengal [Myanmar, Nepal, Pakistan, Sri Lanka].

**Habit.** Annual.

**1310. **Eragrostis zeylanica** Nees, Nov. Actorum Acad. Caes. Leop.-Carol. Nat. Cur., Suppl. 19(1): 204. 1843 **

*Eragrostis elongata* sensu Stapf, Fl. Brit. India (J.D. Hooker). 7(22): 319. 1896, non Jacq., 1813

**Type. **“In Ceylona, Macrae, Julio 1829 (Herb. Lindl. et Arnott.)” Sri Lanka; July 1829; *Macrae s.n.*

**Distribution.** Andaman and Nicobar Islands, Assam, Chhattisgarh, Kerala, Madhya Pradesh, West Bengal [Myanmar, Sri Lanka].

**Habit.** Perennial.

### Tribe Zoysieae Benth., 1881

Ligule generally a fringe of hairs. Spikelets laterally compressed, with a single flower. Lower glume absent, or with no veins, or with only one vein. Lemma with one vein, awnless. Pericarp free from seed coat.

Note that the genera *Spartina* and *Crypsis* are derived from within *Sporobolus* so the two are combined here, following the work of Peterson et al. (2014b, 2014c).

***Sporobolus* R. Br., Prodr. Fl. Nov. Holland. 169. 1810**

**Lectotype. ***Sporobolus indicus* (L.) R. Br. (*Agrostis indica* L.). LT designated by Pfeiffer, Nomencl. Bot. 2: 1247. 1874.

**1311. **Sporobolus africanus** (Poir.) A. Robyns & Tournay, Bull. Jard. Bot. État Bruxelles 25: 242. 1955 **

*Agrostis africana* Poir., Encycl. (Lamarck) Suppl. 1. 254. 1810. Type: South Africa: Cape Province; *Thunberg s.n.* (UPS).

**Distribution.** Tamil Nadu. Naturalized; native to Africa, Arabia.

**Habit.** Perennial.

**1312. **Sporobolus airoides** (Torr.) Torr., Pacific Railr. Rep. 7, Pt. 3 (Parkes) 7: 21. 1856 **

*Agrostis airoides* Torr., Ann. Lyceum Nat. Hist. New York 1(1): 151–152. 1824. Type: “Hab. on the branches of the Arkansas, near Rocky Mountains.” USA: Colorado, headwaters of the Arkansas River; *E. James s.n.* (NY-327612).

*Vilfa airoides* (Torr.) Trin. ex Steud., Nomencl. Bot., ed. 2 (Steudel) 2: 766. 1841

*Sporobolus diffusissimus* Buckl., Proc. Acad. Nat. Sci. Philadelphia 1862: 90. 1862. Type: USA: Texas, Western Texas; *Wright 726*

**Distribution.** Rajasthan [also in North America]. Naturalized; native to North America.

**Habit.** Perennial.

**Remarks.** Locality in India fide Naithani (1990).

**1313. **Sporobolus alterniflorus** (Loisel.) P. M. Peterson & Saarela, Taxon 63(6): 1236. 2014 **

*Spartina alterniflora* Loisel., Fl. Gall. 719. 1807. Type: “Habitat circa Baionam, in pascuis limosis ad ripas Aturi.”

**Distribution.** Gujarat, Maharashtra [China]. Naturalized; native to North America.

**Habit.** Perennial.

**Remarks. **Record for Maharashtra fide Naithani (1990).

**1314. **Sporobolus capillaris** Miq., Nieuwe Verh. Eerste Kl. Kon. Ned. Inst. Wetensch. Amsterdam ser. 3, 4: 37. 1851 **

*Sporobolus scabrifolius* Bhide, J. Proc. Asiat. Soc. Bengal, n.s., 8: 312. 1912

**Type.** India: “Habitat in Peninsula Indiae orientalis”; *R. Wight s.n. *(Glasgow 3309).

**Distribution.** Bihar, Chhattisgarh, Karnataka, Madhya Pradesh, Maharashtra, Rajasthan, Tamil Nadu [Myanmar].

**Habit.** Annual.

**Remarks.** Records for Karnataka, Madhya Pradesh, Maharashtra and Tamil Nadu fide Naithani (1990).

**1315. **Sporobolus coromandelianus** (Retz.) Kunth, Révis. Gramin. 1: 68. 1829 **

*Agrostis coromandeliana* Retz., Observ. Bot. (Retzius) 4: 19. 1786–1787. Type: “In aridis duris Malabariae legit indefessis Koenig.” India: Malabar, Naguhr, propre Sammelliota; 1782; *C.D.E. Koenig s.n.* (LD).

*Vilfa coromandeliana* (Retz.) P. Beauv., Ess. Agrostogr. 16: 147. 1812

*Vilfa commutata* Trin., Sp. Gram. [Trinius] 1: t. 10. 1823. Type: “V. sp. Ind. Or.” Klein ex Stephan ex Wildenow in Herb Trinius; November 15, 1799; India: Madras: Red Hills (LE).

*Sporobolus commutatus* (Trin.) Kunth, Enum. Pl. [Kunth] 1: 214. 1833

*Vilfa roxburghii* Nees ex Trin., Mém. Acad. Imp. Sci. Saint-Pétersbourg, Sér. 6, Sci. Math., Seconde Pt. Sci. Nat. 5: 59. 1839, nom. invalid, cited as synonym of *Vilfa commutata*

*Vilfa roxburghiana* Nees ex Wight., Cat. no. 1742. 1834, nom. nud.

**Distribution.** Andhra Pradesh, Bihar, Daman and Diu, Goa, Gujarat, Haryana, Karnataka, Kerala, Madhya Pradesh, Maharashtra, Punjab, Odisha, Rajasthan, Tamil Nadu, Uttar Pradesh [Afghanistan, China, Myanmar, Pakistan, Sri Lanka].

**Habit.** Annual.

**1316. **Sporobolus diandrus** (Retz.) P. Beauv., Ess. Agrostogr. 26, 147, 178. 1812 **

*Agrostis diandra* Retz., Observ. Bot. (Retzius) 5: 19. 1788 (“1789”). Type: “Missit cel. Koenig sub nominee Cinnae.” India; *J. Koenig s.n.* (LD).

*Sporobolus indicus* (L.) R. Br. var. *diandrus* (Retz.) Jovet & Guedes, Taxon 22(1): 163. 1973 (as “diander”)

*Vilfa erosa* Trin., Mém. Acad. Imp. Sci. Saint-Pétersbourg, Sér. 6, Sci. Math., Seconde Pt. Sci. Nat. 5(2): 86. 1839. Type: Sri Lanka; *Koenig s.n.* (LE-TRIN-1698.01). LT designated by Baaijens & Veldkamp in Blumea 35: 434. 1991.

*Vilfa retzii* Steud., Nomencl. Bot., ed. 2 (Steudel) 2: 768. 1841

*Sporobolus diandrus* (Retz.) P. Beauv. var. *major* Buse, Pl. Jungh. 3: 343. 1854. Type: Java: Cibogo; June; *Junghuhn s.n.* (L-903.342-96).

*Sporobolus indicus* (L.) R. Br. var. *major* (Buse) Baaijens, Blumea 35(2): 437. 1991

**Distribution.** Assam, Bihar, Maharashtra, Manipur, Meghalaya, Nagaland, Odisha, West Bengal [China, Myanmar, Nepal, Pakistan, Sri Lanka].

**Habit.** Perennial.

**1317. **Sporobolus fertilis** (Steud.) Clayton, Kew Bull. 19: 291. 1965 **

*Agrostis fertilis* Steud., Syn. Pl. Glumac. 1: 170. 1854. Type: “Ex Hrbo. Musei Logd. Bat. Japonia.” Lectotype: Japan; *Burger s.n. *(L-908.97-171). LT designated by Baaijens & Veldkamp in Blumea 35: 437. 1991.

*Sporobolus indicus* (L.) R. Br. var. *fertilis* (Steud.) Jovet & Guedes, Taxon 22: 163. 1973

**Distribution.** Andhra Pradesh, Arunachal Pradesh, Assam, Bihar, Chhattisgarh, Gujarat, Himachal Pradesh, Karnataka, Kerala, Madhya Pradesh, Maharashtra, Manipur, Meghalaya, Nagaland, Odisha, Rajasthan, Tamil Nadu, Uttar Pradesh, Uttarakhand, West Bengal [Bhutan, China, Myanmar, Nepal, Sri Lanka].

**Habit.** Perennial.

**1318. **Sporobolus festivus** Hochst. ex A. Rich., Tent. Fl. Abyss. 2: 398. 1850–1851 **

**Syntypes.** “Crescit in ripis rivuli Mariam-Chawisto, mense November; Quartin Dillon.” Ethiopia: prope Tchelatcheranne July 31, 1840; *G.H.W. Schimper s.n. *(P). Ethiopia: in campis prope Avar Semmaka in provincia Chire; *Quartin Dillon s.n. *(P).

**Distribution.** Bihar, Maharashtra [Africa].

**Habit.** Perennial.

**Remarks.** Record for Maharashtra fide Naithani (1990).

**1319. **Sporobolus fimbriatus** (Nees ex Trin.) Nees, Fl. Afr. Austral. Ill. 156. 1841 **

*Vilfa fimbriata* Nees ex Trin., Mém. Acad. Imp. Sci. Saint-Pétersbourg, Sér. 6, Sci. Math., Seconde Pt. Sci. Nat. 4(1–2): 69. 1840. Type: South Africa: Cape

**Distribution.** India [Africa].

**Habit.** Perennial.

**Remarks.** The report of this species for India is dubious; documentation is lacking.

**1320. **Sporobolus hajrae** P. Umam. & P. Daniel, Nordic J. Bot. 18(5): 577. 1998 **

**Type.** India: Tamil Nadu, Chidambaranar District, Veppalodai seacoast, c. 25km from Tuticorin; July 30, 1995; *P. Daniel; P. Umamaheswari 103594* (CAL).

**Distribution. **Tamil Nadu.

**Habit.** Annual.

**1321. **Sporobolus helvolus** (Trin.) T.Durand & Schinz, Consp. Fl. Afr. [T.A. Durand & H. Schinz] 5: 820. 1894 (“1895”) (as “helvola”) **

*Vilfa helvola* Trin., Mém. Acad. Imp. Sci. Saint-Pétersbourg, Sér. 6, Sci. Math., Seconde Pt. Sci. Nat. 4(2): 52. 1840. Syntypes: Saudia Arabia: Wadi Diara, Habessin, in siccis; *Ehrenberg s.n.* (LE). Senegal; *Leprieur s.n.* (LE). Senegal; *Lelievre s.n.* (LE).

*Vilfa glaucifolia *Hochst. ex Steud, Syn. Pl. Glumac. 1: 154. 1854. Type: Africa: Sudan: Nubia, Kordovan, Arasch-Cool; September 30, 1839; *Kotschy 74* (WAG).

*Sporobolus glaucifolius* (Hochst. ex Steud.) Hochst. ex T. Durand & Schinz, Consp. Fl. Afr. [T.A. Durand & H. Schinz] 5: 820. 1894 (“1895”)

**Distribution.** Gujarat, Maharashtra, Rajasthan [Pakistan].

**Habit.** Perennial.

**1322. **Sporobolus humilis** Presl ssp. **minor** Veldkamp, Blumea 35: 420. 1991 **

*Vilfa geniculata* Nees, Syn. Pl. Glumac. (Steudel) 1: 156. 1854. Type: India: Madras

*Sporobolus geniculatus* (Nees) Aitch., Cat. Punjab Pl. 165. 1869

*Sporobolus tremulus* auct. non Kunth: Kunth, Révis. Gramin. 1: 67. 1829

**Type. **India: Madras, not indicated (P, holo, not found).

**Distribution.** Assam, Bihar, Maharashtra, Punjab, Tamil Nadu [Myanmar, Pakistan].

**Habit.** Perennial.

**1323. **Sporobolus indicus** (L.) R. Br., Prodr. Fl. Nov. Holland. 170. 1810 var. **indicus

*Agrostis indica* L., Sp. Pl. 63. 1753. Type: “Habitat in India.” Lectotype: Clayton 460b, Herb. Linn. No. 84.36 (LINN). LT designated by Baaijens & Veldkamp in Blumea 35: 422. 1991.

**Distribution.** Andhra Pradesh, Arunachal Pradesh, Assam, Bihar, Chhattisgarh, Jharkhand, Madhya Pradesh, Maharashtra, Nagaland, Punjab, Tamil Nadu, Tripura [Myanmar, Pakistan].

**Habit. **Perennial.

**1324. **Sporobolus indicus** (L.) R. Br. var. **flaccidus** (Roem. & Schult.) Veldkamp, Blumea 35(2): 433. 1991 **

*Agrostis elongata* (R. Br.) Roth var. *flaccida* Roem. & Schult., Syst. Veg., ed. 15 bis [Roemer & Schultes] 2: 368. 1817. Type: India; *Roth 80 β*

**Distribution.** Andaman and Nicobar Islands, Andhra Pradesh, Assam, Bihar, Chhattisgarh, Goa, Gujarat, Himachal Pradesh, Karnataka, Kerala, Madhya Pradesh, Maharashtra, Nagaland, Odisha, Sikkim, Tamil Nadu, Tripura, Uttar Pradesh, West Bengal [Pakistan, Sri Lanka].

**Habit.** Perennial.

**1325. **Sporobolus ioclados** (Nees ex Trin.) Nees, Fl. Afr. Austral. Ill. 161. 1841 **

*Vilfa ioclados* Nees ex Trin., Mém. Acad. Imp. Sci. Saint-Pétersbourg, Sér. 6, Sci. Math., Seconde Pt. Sci. Nat. 4(2): 65. 1840. Type: South Africa: Cape; *Drége & Wonderhuivel s.n.* (LE).

*Sporobolus marginatus* Hochst. ex A. Rich., Tent. Fl. Abyss. 2: 397. 1850–1851. Syntypes: Ethiopia: in valle lata Meda; April; *G.H.W. Schimper s.n. *(L). “Crescit in provincia Chiré”; *Quartin Dillon s.n.* et in valle lata Meda mense Aprili*; Schimper s.n. *

*Sporobolus arabicus* Boiss., Diagn. Pl. Orient. ser. 1, 13: 47. 1854. Type: “Hab. in aridis Arabiae prope Mascate Aucher no. 5425.” Saudi Arabia: Muscat, desert; *Aucher-Eloy 5425*

**Distribution. **Gujarat, Haryana, Madhya Pradesh, Maharashtra, Rajasthan, Tamil Nadu, Uttar Pradesh [Pakistan, Africa].

**Habit.** Perennial.

**Remarks. **Distribution in Gujarat, Haryana, Madhya Pradesh and Uttar Pradesh fide Naithani (1990).

**1326. **Sporobolus maderaspatanus** Bor, Kew Bull. 1957: 234. 1957 **

**Type. **India: Madras, Guntur District; October 22, 1902; *C.A. Barber 4720* (K).

**Distribution.** Andhra Pradesh, Goa, Gujarat, Karnataka, Maharashtra, Rajasthan, Tamil Nadu [Sri Lanka].

**Habit.** Perennial.

**1327. **Sporobolus piliferus** (Trin.) Kunth, Enum. Pl. 1: 211. 1833 **

*Vilfa pilifera* Trin., Gram. Unifl. Sesquifl. 157. 1824. Type: “V. spp. Nepal.” Lectotype: Nepal, *Wallich 3774 *(LE). LT designated by Baaijens & Veldkamp in Blumea 35: 441. 1991.

*Sporobolus stachyanthus* A. Rich., Tent. Fl. Abyss. 2: 394. 1850–1851. Type: “Crescit in montosis planitiei provinciae Chire, mense Octobre; *Quartin Dillon s.n.*”

*Triachyrum nilagiricum* Steud., Syn. Pl. Glumac. 1: 176. 1854. Type: India: Tamil Nadu, Nilagiri Mountain; 1851; *Hohenacker 931*

*Sporobolus ciliatus* Munro ex Hook. f., Fl. Brit. India (J.D. Hooker). 7(21): 251. 1896, nom. nud.

**Distribution.** Andhra Pradesh, Arunachal Pradesh, Assam, Bihar, Himachal Pradesh, Jammu and Kashmir, Karnataka, Kerala, Maharashtra, Meghalaya, Odisha, Tamil Nadu, Uttar Pradesh, Uttarakhand, West Bengal [Bhutan, China, Nepal].

**Habit.** Annual.

**1328. **Sporobolus schoenoides** (L.) P. M. Peterson, Taxon 63(6): 1234. 2014 **

*Phleum schoenoides* L., Sp. Pl. 1: 60. 1753. Type: “Habitat in Italia, Smyrna, inque Hispania. Loefling.” Lectotype: *Herb. Linn. No. 81.7* (LINN). LT designated by Clayton in Polhill (ed.), Fl. Trop. E. Africa, Gramineae 2: 353. 1974.

*Crypsis schoenoides* (L.) Lam., Tabl. Encycl. 1: 166. t. 42. f. 1. 1791

*Heleochloa schoenoides* (L.) Host, Icon. Descr. Gram. Austriac. 1: 23. t. 30. 1801

**Distribution. **Gujarat, Madhya Pradesh, Punjab, Rajasthan [Afghanistan, China, Pakistan].

**Habit.** Annual.

**1329. **Sporobolus spicatus** (Vahl) Kunth, Révis. Gramin. 1: 67. 1829 **

*Agrostis spicata* Vahl, Symb. Bot. (Vahl) 1: 9. 1790. Type: Egypt; *Forsskal s.n. *(C).

*Vilfa spicata* P. Beauv., Ess. Agrostogr. 16, 147, 182. 1812, nom. nud.

**Distribution.** Andhra Pradesh, Karnataka, Tamil Nadu [also in Saudi Arabia, Africa].

**Habit.** Perennial.

**1330. **Sporobolus tenuissimus** (Schrank) Kuntze, Revis. Gen. Pl. 3(3): 369. 1898 **

*Panicum tenuissimum* Schrank, Denkschr. Königl.-Baier. Bot. Ges. Regensburg 2: 26. 1822. Type: “Brasilien, Hr. Dr. Martius” “Pflege: in warmen Hause.”

*Sporobolus mangaloricus* Miq., Nieuwe Verh. Eerste Kl. Kon. Ned. Inst. Wetensch. Amsterdam ser. 3, 4: 36. 1851. Type: India: Tamil Nadu, Canara, near Mangalore; 1847; *Hochstetter 137 *(U).

*Vilfa mangalorica* (Miq.) Hochst. ex Steud., Syn. Pl. Glumac. 1: 159. 1854.

*Vilfa minutiflora* Trin., Gram. Unifl. Sesquifl. 158. 1824. Type: cultivated in Leningrad from seeds collected in Brazil; *Martius s.n.* (LE-TRIN-1718.01).

*Sporobolus minutiflorus* (Trin.) Link, Hort. Berol. [Link] 1: 88. 1827

**Distribution.** Andhra Pradesh, Bihar, Goa, Karnataka, Kerala, Madhya Pradesh, Maharashtra, Rajasthan, Tamil Nadu [China]. Naturalized; native to tropical America.

**Habit.** Annual.

**1331. **Sporobolus tetragonus** Bor, Kew Bull. 1949: 251. 1949 **

*Sporobolus pulchellus* sensu Hook. f., Fl. Brit. India (J.D. Hooker). 7(21): 252. 1896, non R. Br., 1810

**Type.** Siam, Kanburi; *A.F. G. Kerr 19762* (K).

**Distribution.** Bihar, Madhya Pradesh, Maharashtra [Myanmar].

**Habit.** Annual.

**1332. **Sporobolus tourneuxii** Coss., Bull. Soc. Bot. France 36: 250. 1889 **

*Sporobolus sindicus* Stapf ex T. Cooke, Fl. Bombay 2: 1018. 1908. Type: Pakistan: Sind, Dadu District, Thano Bula Khan to Kotri; *Woodrow s.n.* (K).

**Type.** Algeria: Tunetia, desert; 1884; *Letourneux s.n.* (K).

**Distribution.** Rajasthan [Pakistan].

**Habit.** Perennial.

**1333. **Sporobolus virginicus** (L.) Kunth, Révis. Gramin. 1: 67. 1829 **

*Agrostis virginica* L., Sp. Pl. 1: 63. 1753. Type: “Habitat in Virginia.” Lectotype: Clayton 507, Herb. Linn. No. 84.30 (LINN). LT designated by Hitchcock in Contr. U.S. Natl. Herb. 12: 119. 1908.

*Crypsis virginica* (L.) Nutt., Gen. N. Amer. Pl. [Nuttall] 1: 49. 1818

*Podosemum virginicum* (L.) Link, Hort. Berol. [Link] 1: 85. 1827

*Vilfa virginica* (L.) P. Beauv., Ess. Agrostogr. 16, 149, 182. 1812

*Agrostis littoralis* Lam., Tabl. Encycl. 1: 161. 1791. Type: “America merid., Communic. a D. Richard.” Lectotype: Antilles: Virgin Islands: bayis Trium insularum Danarum; May 1787; *L.C.M. Richard s.n.*(P). LT designated by Baaijens & Veldkamp in Blumea 35: 446. 1991.

*Sporobolus littoralis* (Lam.) Kunth, Révis. Gramin. 1: 68. 1829

*Vilfa littoralis* (Lam.) P. Beauv., Ess. Agrostogr. 16: 147, 181. 1812

*Vilfa intermedia* Trin., Gram. Unifl. Sesquifl. 156. 1824. Type: “V. sp. Ind. occ.” [error, corrected in 1840 to Mauritan. Sieber]. Mauritius; *Sieber II-38* (LE-TRIN-1710.01).

*Agrostis pungens* Muhl., Descr. Gram. (Muhlenberg) 72. 1817, non Schreb., 1769. Type: “Agrostis dure Willd. persimilis et Agrostis virginicae Swarz.”

*Sporobolus tremulus* (Willd.) Kunth, Révis. Gramin. 1: 67. 1829

*Agrostis juncea* Lam., Tabl. Encycl. 1: 161. 1791. Type: “Ex India, insula Franciae. A matrella. L. fotre varietas praecedentis.” India; *Sonnerat s.n.* (P).

*Vilfa tremula* Trin., Gram. Unifl. Sesquifl. 155. 1824. nom. nov. for *Agrostis juncea *Lam., non *V. juncea* Trin. (1824)

*Agrostis tremula* Willd., Sp. Pl., ed. 4 [Willdenow] 1: 372. 1797, nom. superfl. & illeg. for *A*. *juncea* Lam.

**Distribution.** Andhra Pradesh, Daman and Diu, Goa, Gujarat, Karnataka, Kerala, Maharashtra, Tamil Nadu [China, Sri Lanka].

**Habit.** Perennial.

**Remarks.** Cosmopolitan.

**1334. **Sporobolus wallichii** Munro ex Trimen., J. Bot. 27: 171. 1889 **

**Type.** “Collected by the late W. Ferguson, early in 1886, somewhere between Trincomalie and Kantalai Tank.” Numer. List [Wallich] no. 3769A.

**Distribution.** Andhra Pradesh, Bihar, Jharkhand, Karnataka, Odisha, Maharashtra, Rajasthan, Tamil Nadu [China, Myanmar, Sri Lanka].

**Habit. **Annual or perennial.

***Urochondra* C.E. Hubb., Hooker’s Icon. Pl. 35: sub t. 3457. 1947**

**Type. ***Urochondra setulosa* (Trin.) C.E. Hubb. (*Vilfa setulosa* Trin.).

**1335. **Urochondra setulosa** (Trin.) C.E. Hubb., Hooker’s Icon. Pl. 35: sub t. 3457. 1947 **

*Vilfa setulosa* Trin., Mém. Acad. Imp. Sci. Saint-Pétersbourg, Sér. 6, Sci. Math., Seconde Pt. Sci. Nat. 4(2): 55. 1840. Type: [Saudi] Arabia: Ga-el-Ma, banks of salt-water creeks and saline flats; *Ehrenberg & Hemprich s.n.*

*Crypsis setulosa* (Trin.) Mez, Repert. Spec. Nov. Regni Veg. 17: 292. 1921

*Heleochloa setulosa* (Trin.) Blatt. & McCann, Sci. Monogr. Imp. Council Agric. Res. India 5: 205. t. 134. 1935

*Sporobolus setulosus* (Trin.) A. Terracc., Annuario Reale Ist. Bot. Roma 5: 95. 1893

*Crypsis dura* Boiss., Diagn. Pl. Orient. ser. 2. 4: 125. 1859. Type: “Hab. in ditionibus Scinde et Beloutschistan”; *Griffith 455*

*Heleochloa dura* (Boiss.) Boiss., Fl. Orient. [Boissier] 5: 477. 1884

**Distribution.** Goa, Gujarat, Karnataka, Maharashtra, Rajasthan [Pakistan].

**Habit.** Perennial.

***Zoysia* Willd., Ges. Naturf. Freunde Berlin Neue Schriften 3: 440. 1801, nom. cons.**

**Type.***Zoysia pungens* Willd.

**1336. **Zoysia japonica** Steud., Syn. Pl. Glumac. 1: 414. 1854 **

*Osterdamia japonica* (Steud.) Hitchc., Bull. U.S.D.A. 772: 166. 1920

**Type.** Japonia.

**Distribution.** Maharashtra [also in Japan, Korea, North America]. Naturalized and cultivated; native to eastern Asia, Japan.

**Habit. **Perennial.

**1337. **Zoysia matrella** (L.) Merr., Philipp. J. Sci., C 7: 230. 1912 var. **matrella

*Agrostis matrella* L., Mant. Pl. Altera 185. 1771. Type: “Habitat in Malabariae arenosis. Koenig. 56.” Lectotype: König 56, Herb. Linn. No. 84.11 (LINN). LT designated by Goudswaard in Blumea 26: 171. 1980.

*Osterdamia matrella* (L.) Kuntze, Revis. Gen. Pl. 2: 781. 1891

*Zoysia pungens* Willd., Ges. Naturf. Ferunde Berlin Neue Schriften 3: 441. 1801. Type: India; *Klein s.n.*

**Distribution.** Andhra Pradesh, Daman and Diu, Goa, Gujarat, Karnataka, Kerala, Maharashtra, Odisha, Tamil Nadu, Uttar Pradesh, West Bengal [China, Sri Lanka].

**Habit.** Perennial.

**1338. **Zoysia pacifica** (Goudsw.) M. Hotta & S. Kuroki, Acta Phytotax. Geobot. 45: 71. 1994 **

*Zoysia matrella* (L.) Merr. var. *pacifica* Goudsw., Blumea 26: 172. 1980. Type: Rhukyu I., Yonakuni I.; *Hatusima 24127* (L.)

*Zoysia tenuifolia* sensu Bor, Grasses Burma, Ceylon, India and Pakistan 684. 1960, non Willd. ex Trin. 1836

**Distribution.** Andhra Pradesh, Daman and Diu, Goa, Gujarat, Karnataka, Kerala, Maharashtra, Odisha, Tamil Nadu [China].

**Habit.** Perennial.

### Tribe Cynodonteae Dumort., 1824

Morphology in this tribe is almost as variable as within the subfamily as a whole. There are no obvious morphological synapomorphies (Kellogg 2015b).

***Acrachne* Wight & Arn. ex Chiov., Ann. Bot. (Rome) 8(3): 361. 1908**

**Type. ***Acrachne verticillata* (Roxb.) Chiov. (*Eleusine verticillata* Roxb.).

**1339. **Acrachne henrardiana** (Bor) S.M. Phillips, Kew Bull. 37(1): 158. 1982 **

*Dactyloctenium henrardianum* Bor, Blumea, Suppl. 3: 44. 1946. Type: India: Tamil Nadu, Ramnad District, Pamban; January 19, 1945; *Daniel & S.R. Raju 20089* (K).

*Arthrochloa henrardiana* (Bor) J. W. Lorch, J. Indian Bot. Soc. 39: 490. f. 1–5. 1960

*Normanboria henrardiana* (Bor) Butzin, Taxon 27(2–3): 301. 1978

**Distribution.** Tamil Nadu.

**Habit.** Annual.

**1340. **Acrachne racemosa** (B. Heyne ex Roth) Ohwi, Bull. Tokyo Sci. Mus. 18: 1. 1947 **

*Eleusine racemosa* B. Heyne ex Roth, Syst. Veg., ed. 15 bis [Roemer & Schultes] 2: 583. 1817. Type: India Orientali; *Heyne s.n.*

*Leptochloa racemosa* (B. Heyne ex Roth) Kunth, Révis. Gramin. 1: 91. 1829

*Eleusine verticillata* Roxb., Fl. Ind. (Carey and Wallich ed.). 1: 346. 1820. Type: India: “A native of moist pasture ground.”

*Acrachne verticillata* (Roxb.) Lindl. ex Chiov., Ann. Bot. (Rome) 8(3): 361–362. 1908

*Leptochloa verticillata* (Roxb.) Kunth, Révis. Gramin. 1: 91. 1829

**Distribution.** Andhra Pradesh, Assam, Bihar, Delhi, Gujarat, Himachal Pradesh, Jammu and Kashmir, Karnataka, Madhya Pradesh, Maharashtra, Punjab, Rajasthan, Tamil Nadu, Uttar Pradesh, West Bengal [Afghanistan, China, Myanmar, Pakistan, Sri Lanka].

**Habit.** Annual.

**1341. **Acrachne sundararajii** P. Umam., Muthuk. & P. Daniel, Kew Bull. 52(4): 1007 1997 **

**Type.** India: Tamil Nadu, Kanyakumari District, Vivekanandhapuram; November 21, 1995; *Muthukumar 106403* (CAL).

**Distribution.** Tamil Nadu.

**Habit. **Annual.

***Aeluropus* Trin., Fund. Agrost. (Trinius) 143. 1820**

**Type. ***Aeluropus laevis* Trin., nom. illeg. (*Dactylis brevifolia* J. Koenig ex Willd., *Aeluropus brevifolius* (J. Koenig ex Willd.) Trin. ex Wall.).

**1342. **Aeluropus lagopoides** (L.) Trin. ex Thwaites, Enum. Pl. Zeyl. (Thwaites). 374. 1864 (as “lagopodioides”) **

*Dactylis lagopoides* L., Mant. Pl. 33. 1767. Type: “Habitat in India. Burmannus.” Lectotype: *J. Burman s.n., Herb. Linn. No. 90.5* (LINN). LT designated by Renvoize in Cafferty et al. (ed.), Taxon 49: 250. 2000.

*Koeleria lagopoides* (L.) Panz ex Spreng., Syst. Veg. (ed. 16) [Sprengel] 1: 332. 1824 (“1825”) (as “Kölera”)

*Poa lagopoides* (L.) Kunth, Révis. Gramin. 1: 111. 1829 (as “lagopodioides”)

*Sesleria lagopoides* (L.) Spreng., Pl. Min. Cogn. Pug. 2: 22. 1815 (as “Sessleria lagopodioides”)

*Aeluropus concinnus* Fig. & de Not., Mem. Reale Accad. Sci. Torino ser. 2, 12: 257. 1852. Type: “In arenosis salsugineis ad Wadi-Dahab”; *Figari s.n.*

*Dactylis repens* Desf., Fl. Atlant. 1: 79. t. 15. 1798. Type: “Habitat in arensis ad maris littoral et in deserto.”

*Aeluropus repens* (Desf.) Parl., Fl. Ital. (Parlatore) 1: 462. 1848

*Poa repens* (Desf.) M. Bieb., Fl. Taur.-Caucas. 3: 69. 1819

*Calotheca repens* (Desf.) Spreng., Syst. Veg. (ed. 16) [Sprengel] 1: 347. 1824 (“1825”)

*Calotheca niliaca* Spreng., Syst. Veg. (ed. 16) [Sprengel] 1: 348. 1824 (“1825”)

*Aeluropus lagopoides* (L.) Trin. ex Trimen, Syst. Cat. Fl. Pl. Ceylon 110. 1885, isonym

*Aeluropus brevifolius* Nees ex Steud., Nomencl. Bot., ed. 2 (Steudel) 1: 30. 1840, nom. nud.

*Poa tunetana* Spreng., Pl. Min. Cogn. Pug. 2: 20. 1815, nom. superfl. & illeg. for *Dactylis repens* Desf.,

*Aeluropus laevis* Trin., Fund. Agrost. (Trinius) 143. t. 12. 1820, nom. superfl. & illeg. for *Dactylis brevifolia*

*Dactylis lagopoides* sensu Dalzell & A. Gibson, Bombay Fl. 298. 1861 (as “lagopodioides”), non L., 1767

*Aeluropus villosus* Trin. in C.A. Mey., Verz. Pfl. Casp. Meer. (C.A. von Meyer) 18. 1831, nom. superf. & illeg. for *Poa repens* (Desf.) M. Bieb.

**Distribution.** Andhra Pradesh, Bihar, Daman and Diu, Delhi, Gujarat, Kerala, Maharashtra, Rajasthan, Odisha, Punjab, Tamil Nadu, West Bengal [Afghanistan, Pakistan, Sri Lanka].

**Habit.** Perennial.

**1343. **Aeluropus littoralis** (Gouan) Parl., Fl. Ital. (Parlatore) 1: 461. 1848 **

*Poa littoralis* Gouan, Fl. Monsp. 470. 1765. Type: In maritimis arenosis a Villenueve, Maguelone, France, *Gouan s.n*.

*Calotheca litoralis* (Gouan) Spreng., Syst. Veg. (ed. 16) [Sprengel] 1: 347. 1824 (“1825”)

*Dactylis littoralis* (Gouan) Willd., Sp. Pl., ed. 4 [Willdenow] 1: 408. 1797

*Melica littoralis* (Gouan) Raspail, Ann. Sci. Nat. (Paris) 5: 443. 1825

*Poa pungens* M. Bieb., Fl. Taur.-Caucas. 1: 65. 1808

*Aeluropus pungens* (M. Bieb.) K. Koch, Linnaea 21(4): 408. 1848

*Aeluropus littoralis* ssp. *pungens* (M. Bieb.) Tzvelev, Novosti Sist. Vyss. Rast. 8: 73. 1971

**Distribution.** India; states not recorded. [China].

**Habit.** Perennial.

***Bouteloua* Lag., Varied. Ci. 2(4): 134. 1805 (‘Botelua’), nom. et orth. cons.**

**Type. ***Bouteloua racemosa* Lag.

**1344. **Bouteloua aristidoides** (Kunth) Griseb., Fl. Brit. W. I. (Grisebach) 537. 1864 **

*Dinebra aristidoides *Kunth, Nov. Gen. Sp. 1: 171–172 (ed. qto.). 1816. Type: “Crescit in asperis frigidis convallis Tolucensis, alt. 1320 hexap.” Mexico: near Nevada, Toluca; *Humboldt & Bonpland 67* (P).

*Atheropogon aristidoides* (Kunth) Roem. & Schult., Syst. Veg., ed. 15 bis [Roemer & Schultes] 2: 415. 1817 (as “aristidioides”)

*Eutriana aristidoides* (Kunth) Trin., Gram. Unifl. Sesquifl. 242. 1824

*Triathera aristidoides* (Kunth) Nash in Small, Fl. S.E. U.S. 137. 1903

*Bouteloua ciliata* Griseb., Abh. Königl. Ges. Wiss. Göttingen 24: 302. 1879. Type: Argentina: Salta, cerca del Pasaje del Río Juramento; February 21, 1873; *P.G. Lorentz & G. Hieronymus 352* (GOET).

*Dinebra hirsuta* J. Presl in C. Presl, Reliq. Haenk. 1(4–5): 292. 1830. Type: “Habitat in Peruvia” Peru; *Haenke s.n.* (PR).

*Eutriana hirsuta* (J. Presl) Kunth, Enum. Pl. (Kunth) 1: 280. 1833

*Aristida unilateralis* Willd. ex Steud., Nomencl. Bot., ed. 2 (Steudel) 1: 132. 1840, nom. superfl & illeg. for *Eutriana aristidoides*

**Distribution.** India; states not recorded. Naturalized; native to North and South America.

**Habit.** Annual.

**1345. **Bouteloua curtipendula** (Michx.) Torr. in Marcy, Explor. Red River Louisiana 300. 1853 **

*Chloris curtipendula* Michx., Fl. Bor.-Amer. (Michaux) 1: 59. 1803. Type: USA; 1795; *Michaux s.n. *(P). “Hab. in aridis regionis Illinoensis ad Wabast et in rupibus ad prairie du rocher.”

*Andropogon curtipendulus* (Michx.) Spreng. ex Steud., Nomencl. Bot., ed. 2 (Steudel) 1: 90. 1840

*Atheropogon curtipendulus* (Michx.) E. Fourn., Mexic. Pl. 2: 138. 1886

C*ynodon curtipendula* (Michx.) Raspail, Ann. Sci. Nat. (Paris) 5: 303. 1825

*Dinebra curtipendula *(Michx.) P. Beauv., Ess. Agrostogr. 98. 158 t. 16, f. 1. 1812

*Eutriana curtipendula* (Michx.) Trin., Fund. Agrost. (Trinius) 161. 1820

*Eutriana affinis* Hook. f., Trans. Linn. Soc. London 20(2): 174. 1847. Syntypes: North America; *Schweinnitz s.n.* Missouri, St. Louis & Texas;* Drummond s.n.*

*Atheropogon affinis* (Hook. f.) E. Fourn., Mexic. Pl. 2: 141. 1886

*Atheropogon apludoides* Muhl. ex Willd., Sp. Pl., ed. 4 [Willdenow] 4: 937. 1806. Type: “Habitat in America boreali.” USA; *G.H.E. Muhlenberg 394* (PH).

*Atheropogon medius* E. Fourn., Mexic. Pl. 2: 139. 1886. Lectotype: Mexico: between T. Miguel and Sadani; October; *Liebmann 581* (US-2307995 (fragm.)). LT designated by Swallen, N. Amer. Fl. 17: 633. 1939.

*Bouteloua racemosa* Lag., Varied. Ci. 2(4): 141. 1805. Type: Collected by L. Nee in Mexico, possibly then cultivated in Madrid.

*Atheropogon racemosus* (Lag.) Roem. & Schult., Syst. Veg., ed. 15 bis [Roemer & Schultes] 2: 414. 1817

*Cynosurus secundus* Pursh, Fl. Amer. Sept. [Pursh] 2(Suppl.): 728. 1814. Type: USA: “upper Louisiana” [Territory = northern middle western states]; *J. Bradbury s.n. *(BM).

*Chloris secundus* (Pursh) Eaton, Man. Bot. (A. Eaton), ed. 5. 173. 1829

*Dineba secunda* (Pursh) Roem. & Schult., Syst. Veg., ed. 15 bis [Roemer & Schultes] 2: 711. 1817

*Heterostegon curtipendula* Schweinf. ex Hook. f., Trans. Linn. Soc. London 20(2): 175. 1847 (as “curtipendulus”), nom. nud., pro syn.

*Melica curtipendula* Steud., Nomencl. Bot. (Steudel) 1: 519. 1821, pro syn. of *Atheropogon apludoides*

*Cynodon melicoides* Raspail, Ann. Sci. Nat. (Paris) 5: 303. 1825, nom superfl. & illeg.

*Bouteloua melicoides* P. Beauv., Ess. Agrostogr. 40. 155. t. 9. f. 6. 1812, nom. superf. & illeg.

*Aristida secunda* Rudge ex Roem. & Schult., Syst. Veg., ed. 15 bis [Roemer & Schultes] 2: 711. 1817, nom. nud., pro. syn.

**Distribution.** India [China]. Naturalized; native to the western hemisphere.

**Habit. **Perennial.

**Remarks.** Taxonomic editor Peterson questions whether any verified records exist for India.

**1346. **Bouteloua gracilis** (Kunth) Lag. ex Griffiths, Contr. U.S. Natl. Herb. 14: 375. 1912, nom**. **cons**.

*Chondrosum gracile* Kunth, Nov. Gen. Sp. 1: 176 (ed. qto.). t. 58. 1816. Type: “Crescit in crepidinibus et devexis montis porphyritici La Buffa de Guanazuato Mexicanorum, alt. 1270.” Mexico: Guanajuato, La Bufa; *Humboldt & Bonpland s.n.* (B-W-1628).

*Actinochloa gracilis* (Kunth) Willd. ex Roem. & Schult., Syst. Veg., ed. 15 bis [Roemer & Schultes] 2: 418. 1817

*Atheropogon gracilis* (Kunth) Spreng., Syst. Veg. (ed. 16) [Sprengel] 1: 293. 1824 (“1825”)

*Atheropogon oligostachyus* Nutt., Gen. N. Amer. Pl. [Nuttall] 1: 78. 1818 (as “oligostachyum”). Type: USA: “on the plains of Missouri [River]”; 1811; *T.Nuttall s.n.* (BM?).

*Bouteloua oligostachya* (Nutt.) Torr. ex A. Gray, Manual (Gray), ed. 2. 553. 1856

*Chondrosum oligostachyum* (Nutt.) Torr. in Marcy, Explor. Red River Louisiana 300. 1853 (as “Chondrosium”)

*Bouteloua stricta* Vasey, Bull. Torrey Bot. Club 15(2): 49. 1888. Type: USA: Texas, western Texas; 1887; *G.C. Nealley s.n.* (US-81710).

**Distribution.** India [China]. Naturalized; native to North America.

**Habit.** Perennial.

**Remarks.** Taxonomic editor Peterson questions whether any verified records exist for India.

***Chloris* Sw., Prodr. (Swartz) 25. 1788**

**Lectotype. ***Chloris cruciata* (L.) Sw. (*Agrostis cruciata* L.) LT designated by G.V. Nash in N.L. Britton et A. Brown, Ill. Fl. N. U.S. ed. 2. 1: 225. 1913; affirmed by Hitchcock, Bull. U.S.D.A. 772: 187. 1920.

**1347. **Chloris barbata** Sw., Fl. Ind. Occid. 1: 200. 1797 **

*Chloris rufescens* Steud., Syn. Pl. Glumac. 1: 206. 1854, nom. later. hom., non Lag., 1805. Type: *Urville* legit in Ins. Maurit.

*Chloris inflata* Link, Enum. Hort. Berol. Alt. 1: 105. 1821. Type grown in the Berlin Botanic Garden from seed said to come from ‘California,’ probably from Mexico” (Hitchcock, 1936, p. 134).

*Andropogon barbatus* L., Mant. Pl. Altera 302. 1771, (as “barbatum”), nom. later hom., non L., 1759. Type: Maurit; *Urville s.n.*

**Type.** Burma: Rangoon; July, 1904; *G. Forrest 346 *(E).

**Distribution. **Andaman and Nicobar Islands, Andhra Pradesh, Assam, Bihar, Delhi, Goa, Gujarat, Jharkhand, Karnataka, Kerala, Madhya Pradesh, Maharashtra, Odisha, Rajasthan, Tamil Nadu, Uttar Pradesh [China, Myanmar, Pakistan, Sri Lanka].

**Habit.** Annual or perennial.

**1348. **Chloris bournei** Rang. & Tadul., J. Indian Bot. Soc. 2: 189. 1921 **

**Syntypes.** India: Coimbatore, on black cotton soil; *K. Rangachariar 1317, 1318, 4443, 5348,6166, 6569 *(MH).

**Distribution.** Andhra Pradesh, Karnataka, Maharashtra, Tamil Nadu [Pakistan].

**Habit.** Perennial.

**Remarks. **Records outside Andhra Pradesh fide Naithani (1990).

**1349. **Chloris flagellifera** (Nees) P. M. Peterson, Taxon 64(3): 458. 2015 **

*Eleusine flagellifera* Nees, Linnaea 16: 220. 1842. Type: India and Arabia; *Royle 86 & Schimper 800*

*Panicum compressum* Forssk., Fl. Aegypt.-Arab. 18. 1775. Type: Yemen: Al Hadiyah “In montibus Hadiensibus”; March 1763; *Forsskal 46* (C), non *Chloris compressa* DC, 1813

*Ochthochloa compressa* (Forssk.) Hilu, Kew Bull. 36(3): 559–563. f. 1(A-G). 1981

*Eleusine compressa* (Forssk.) Asch. & Schweinf. ex C. Chr., Dansk Bot. Ark. 4(3): 12. 1922

*Ochthochloa dactyloides* Edgew., J. Asiat. Soc. Bengal 11: 27. 1842. Type: Missing (vide Kew Bull. 36(3): 559–563. 1981)

*Eleusine arabica* Hochst. ex Steud., Syn. Pl. Glumac. 1: 211. 1854, nom. inval., as syn. of *Eleusineflagellifera* Nees

**Distribution.** Rajasthan [Afghanistan, Pakistan, Africa].

**Habit.** Perennial.

**1350. **Chloris gayana** Kunth, Révis. Gramin. 1: 293, t. 58. 1830 **

*Eustachys gayana* (Kunth) Mundy, Rhodesian Agric. J. 19: 142. 1922

*Chloris abyssinica* Hochst. ex A. Rich., Tent. Fl. Abyss. 2: 406. 1850–1851. Syntypes: Ethiopia: Adoam; September 20, 1837; *Schimper 79* (P). Ethiopia: Djeladjeranne; October 23, 1840; *Schimper 1800* (P). Ethiopia: Aduoa; *Quartin Dillon s.n.*(P).

*Chloris glabrata* Andersson, Naturw. Reise Mossambique 557. 1864. Type: “Standort: auf feuchtem sandigen und moorigen Boden; Wiesengrund bei Tette, 17° S.B., 120 Meilen im Innern des Landes, Kohlen-Sandsteinfrmation”; *Peters in 1842–1848*

**Type.** “Senegalia” Senegal; February 4, 1823; *Doellinger 21 *(K).

**Distribution.** Andhra Pradesh, Delhi, Karnataka, Maharashtra, Rajasthan, Tamil Nadu [China]. Naturalized; native in Africa.

**Habit. **Perennial.

**1351. **Chloris montana** Roxb., Fl. Ind. (Carey & Wallich ed.) 1: 331. 1820 **

*Chloris montana* Roxb. var. *glauca* Hook. f. in Trimen, Handb. Fl. Ceylon 5: 276. 1900. Type: Sri Lanka: hot drier parts of the Island; *Trimen s.n.*

*Chloris decora* sensu Thwaites, Enum. Pl. Zeyl. (Thwaites). 371. 1864, non Nees, 1854

**Type.** India: “This is native of Mountainous tracts only.”

**Distribution.** Andhra Pradesh, Bihar, Daman and Diu, Goa, Gujarat, Karnataka, Kerala, Madhya Pradesh, Maharashtra, Rajasthan, Tamil Nadu, Uttar Pradesh, West Bengal [China, Sri Lanka].

**Habit.** Stoloniferous perennial.

**1352. **Chloris prieurii** Kunth, Révis. Gramin. 2: 441. t. 134. 1831 **

*Enteropogon prieurii* (Kunth) Clayton, Kew Bull. 37(3): 419. 1982

*Chloris punctulata* Hochst. ex Steud., Syn. Pl. Glumac. 1: 205–206. 1854. Type: Egypt: Nubia; September 20, 1839; *T. Kotschy 33350* (L).

**Type.** Senegal: St. Louis; *Leprieur s.n.*

**Distribution.** Maharashtra, Rajasthan [also in Saudi Arabia, Africa].

**Habit.** Annual.

**Remarks.** Record for Rajasthan fide Naithani (1990).

**1353. **Chloris pycnothrix** Trin., Gram. Unifl. Sesquifl. 234. 1824 **

**Type. **“V. spp. Ex Ins. St. Catharnae, ubi detexit ill. De Chamisso.” Brazil: Santa Catarina Island; *Chamisso s.n.* (LE).

**Distribution.** Maharashtra, Tamil Nadu [China, Myanmar, Sri Lanka]. Naturalized. Native to Central and South America.

**Habit.** Annual.

**Remarks.** Record for Tamil Nadu fide Naithani (1990).

**1354. **Chloris quinquesetica** Bhide, J. Proc. Asiat. Soc. Bengal (n.s.) 8(7&8): 311. 1912 **

**Type.** India: Papadi Bassein, growing on the bounds of rice fields in semisalt lands, near Railway Station Road; August 30, 1911; *R.K. Bhide s.n.* (K).

**Distribution.** Andhra Pradesh, Gujarat, Karnataka, Kerala, Maharashtra, Rajasthan [Pakistan].

**Habit.** Perennial.

**Remarks.** Records outside Andhra Pradesh fide Naithani (1990).

**1355. **Chloris roxburghiana** Schult., Mant. 2 (Schultes) 339. 1824 **

*Chloris myriostachya* Hochst., Flora 38: 204. 1855. Type: “Eine sehr ausgezeichnete Art.” Abyssinia [Ethiopia]: Gagevos; 1858; *Schimper, Hb. abyss. Buch. 1416* (STR).

*Chloris polystachya* Roxb., Fl. Ind. (Carey and Wallich ed.) 1: 332. 1820, non Lag., 1816

**Type.** Peninsular India; *Roxburgh s.n.*

**Distribution.** Andhra Pradesh, Karnataka, Kerala, Tamil Nadu. Naturalized; native to Africa.

**Habit.** Perennial.

**1356. **Chloris virgata** Sw., Fl. Ind. Occid. 1: 203. 1797 **

*Chloris alba* J. Presl, Reliq. Haenk. 1(4–5): 289. 1830. Type: “Hab. in Mexico” Mexico; *Haenke s.n.* (PR, US-2830891 isotype fragm,).

*Chloris caudata* Trin. ex Bunge, Enum. Pl. Chin. Bor. 70. 1833. Type: China: “Pekinum” Hebei; 1831; *A. Bunge Herb. Ledebour* (LE, Isotype).

*Chloris compressa* DC., Cat. Pl. Horti Monsp. 94–95. 1813. Type: South America; September, 1828 and August 20, 1828; *A.P. de Candolle 4* (US-2830890, US-80851 isotype fragm.). Cultivated at Montpellier southern France.

*Chloris elegans* Kunth, Nov. Gen. Sp. [H.B.K.] 1: 166 (ed. qto.). t. 49. 1816. Type: “Crescit iisdem fere locis inter Mexico et Queretaro.” Mexico: between Mexico City and Queretaro; *Humboldt & Bonpland 4194 *(P).

*Chloris pubescens* Lag., Varied. Ci. 2(4): 143. 1805. Type: Peru

*Rhabdochloa virgata* (Sw.) P. Beauv., Ess. Agrostogr. 158, 176. 1812

*Chloris penicillata* Willd. ex Steud., Nomencl. Bot., ed. 2 (Steudel) 1: 353. 1840, nom. nud., non (Vahl.) Pers., 1805

**Type.** Antigua; *Swartz s.n.* (S).

**Distribution.** Andhra Pradesh, Bihar, Chhattisgarh, Delhi, Goa, Gujarat, Himachal Pradesh, Jammu and Kashmir, Jharkhand, Madhya Pradesh, Maharashtra, Karnataka, Rajasthan, Uttar Pradesh, West Bengal [Afghanistan, Bhutan, China, Myanmar, Nepal, Pakistan]. Cosmopolitan.

**Habit.** Annual.

**1357. **Chloris wightiana** Nees, Syn. Pl. Glumac. (Steudel) 1: 206. 1854 **

*Chloris incompleta* Wight ex Steud., Syn. Pl. Glumac. 1: 206. 1854, pro syn.

**Syntypes.** Peninsula Indiae Orientalis; *Wight 1766 *(K). India; *Wight 1337* (K).

**Distribution.** Tamil Nadu.

**Habit.** Perennial.

***Cleistogenes* Keng, Sinensia 5: 147. 1934**

**Type. ***Kengia serotina* (L.) Packer (*Festuca serotina* L.).

**1358. **Cleistogenes gatacrei** (Stapf) Bor, Grasses Burma, Ceylon, India & Pakistan 487. 1960 **

*Diplachne gatacrei* Stapf, Bull. Misc. Inform. Kew 1898: 229. 1898. Type: Pakistan: Chitral; *W. Gatacre 17626* (K).

*Kengia gatacrei* (Stapf) Cope, Kew Bull. 35(3): 701. 1980

**Distribution.** Northwest India; states not recorded [Pakistan].

**Habit. **Perennial.

**1359. **Cleistogenes songorica** (Roshev.) Ohwi, J. Jap. Bot. 18: 540. 1942 **

*Diplachne songorica* Roshev., Fl. URSS. 2: 752. 1934. Type: *Keng 3378* (NAS).

*Cleistogenes mutica* Keng, J. Wash. Acad. Sci. 28: 299. 1938

*Kengia mutica* (Keng) Packer, Bot. Not. 113(3): 292. 1960

**Distribution.** Ladakh [China].

**Habit.** Perennial.

**Remarks.** Record for Ladakh fide Naithani (1990).

***Coelachyrum* Hochst. & Nees, Linnaea 16: 221. 1842**

**Type. ***Coelachyrum brevifolium* Hochst. & Nees.

**1360. **Coelachyrum lagopoides** (Burm.f.) Senaratna, Grasses of Ceylon (Perad. Man. No. 8) 79. 1956 **

*Cynosurus lagopoides* Burm. f., Fl. Ind. (N. L. Burman) 29. 1768. Lectotype: *J. Burman s.n., Herb. Linn. No. 90.5 *(LINN). LT designated by Renvoize in Cafferty et al. (ed.), Taxon 49: 250. 2000.

*Eleusine lagopoides* (Burm. f.) Merr., Philipp. J. Sci., 19(3): 339. 1921

*Coelachyropsis lagopoides* (Burm.f.) Bor, Ann. Naturhist. Mus. Wien. 75: 25. 1971

*Coelachyrum brevifolium* Hochst. & Nees, Linnaea 16: 221. 1842. Type. “In arenosis prope Dschiddam d. 30 Decembris legit *W. Schimper*.” cited as “Eleusine brevifolia Hochst et Steud. Plant. Aegypt. -Arab. n. 799”

*Coelachyrum indicum* Hack., Nat. Pflanzenfam., ed. 2 [Engler & Prantl] 2(2): 61. 1887, nom. superfl. & illegit. for *Cynosurus lagopoides*

*Koeleria brevifolia* Spreng., Pl. Min. Cogn. Pug. 2: 21. 1815, nom. superfl. & illegit.

*Poa brevifolia* Kunth, Révis. Gramin. 1: 111. 1829, nom. superfl. & illegit.

*Eragrostis brevifolia *Benth., Hooker’s Icon. Pl. 14: 51. 1881, nom. superfl. & illegit.

*Dactylis brevifolia* J. Koenig ex Willd., Sp. Pl., ed. 4 [Willdenow] 1: 410. 1797, nom. superfl. & illeg. for *Cynosurus lagopoides*

*Eleusine brevifolia* R. Br. ex Hook. f., Fl. Brit. India (J.D. Hooker). 7(22): 294. 1896, nom. superfl. & illegit., non *E. brevifolia* (Nees) Steud., 1854.

**Distribution.** Andhra Pradesh, Karnataka, Tamil Nadu [Sri Lanka].

**Habit.** Annual.

**Remarks.** Distribution outside Karnataka fide Naithani (1990).

***Cynodon* Rich., Syn. Pl. (Persoon) 1: 85. 1805, nom. cons.**

**Type. ***Cynodon dactylon* (L.) Pers. (*Panicum dactylon* L.).

**1361. **Cynodon aethiopicus** Clayton & Harlan, Kew Bull. 24:187. 1970 **

**Type.** Ethiopia, *de Wet OKLA9224. *

**Distribution.** Delhi. Naturalized; native to Africa.

**Habit.** Stoloniferous perennial.

**Remarks. **Record from Delhi fide Naithani (1990).

**1362. **Cynodon barberi** Rangachari & Tadul., J. Bombay Nat. Hist. Soc. 24: 846. 1916 **

*Cynodon barberi* Rang. & Tadul. f. *longifolium *S.K. Jain, Indian Forester 92(11): 699. 1966. Type: India: Orissa, Puri; 1889; *J.H. Walsh s.n.* (CAL).

**Type. **India: Tinnevelly and Godavari District; 1901–1902; *C.A. Barber s.n.*

**Distribution.** Andhra Pradesh, Bihar, Chhattisgarh, Gujarat, Karnataka, Kerala, Lakshadweep, Madhya Pradesh, Maharashtra, Odisha, Rajasthan, Tamil Nadu, Uttar Pradesh, West Bengal [Myanmar, Sri Lanka].

**Habit.** Perennial.

**Remarks.** Most records from India fide Naithani (1990).

**1363. **Cynodon dactylon** (L.) Pers., Syn. Pl. (Persoon) 1: 85. 1805 **

*Panicum dactylon* L., Sp. Pl. 1: 58. 1753. Type: “Habitat in Europa australi.” Lectotype: *Herb. Linn. No. 80.35* (LINN). LT designated by Clayton & Harlan in Kew Bull. 24: 186. 1970.

*Capriola dactylon* (L.) Kuntze, Revis. Gen. Pl. 2: 764. 1891

*Digitaria dactylon* (L.) Scop., Fl. Carniol., ed. 2. 1: 53. 1771

*Milium dactylon* (L.) Moench, Suppl. Meth. (Moench) 67. 1802

*Paspalum dactylon* (L.) Lam., Tabl. Encycl. 1: 176. 1791

*Cynodon erectus* J. Presl in C. Presl, Reliq. Haenk. 1(4–5): 290. 1830. Type: “Hab. in Mexico, et in Peruviae montanis huanaccensibus.” Mexico. Peruano montano; *Haenke s.n. *(PR-495767).

*Cynodon maritimus* Kunth, Nov. Gen. Sp. [H.B.K.] 1: 170 (ed. qto.). 1816

*Digitaria maritima* (Kunth) Spreng., Syst. Veg. (ed. 16) [Sprengel] 1: 272. 1824 (“1825”)

*Cynodon tenuis* Trin. in Spreng., Neue Entdeck. Pflanzenk 2: 63. 1821. Type: “Habitat in America boreali.”

*Dactilon officinale* Vill., Hist. Pl. Dauphiné (Villars) 2: 69. 1787, nom. superfl. & illeg. for *Panicum dactylon*

*Digitaria littoralis* Salisb., Prodr. Stirp. Chap. Allerton 19. 1796, nom. superfl. & illeg. for *Cynodondactylon*

*Chloris cynodon* Trin., Gram. Unifl. Sesquifl. 229. 1824, nom. superfl. & illeg. for *Cynodondactylon*

*Cynodon occidentalis* Willd. ex Steud., Nomencl. Bot., ed. 2 (Steudel) 1: 463. 1840, nom. nud.

*Cynodon portoricensis* Willd. ex Steud., Nomencl. Bot., ed. 2 (Steudel) 1: 463. 1840, nom. nud.

*Agrostis bermudiana* Tussac ex Kunth, Enum. Pl. (Kunth) 1: 259. 1833, nom. nud., pro syn.

*Agrostis filiformis* J. Koenig ex Kunth, Enum. Pl. (Kunth) 1: 261. 1833, nom. nud. pro syn.

*Digitaria stolonifera* Schrad., Fl. Germ. (Schrader) 1: 165.t. 3. f. 9. 1806, nom. superfl. & illeg.

*Fibichia umbellata* Koeler, Descr. Gram. (Koeler) 308. 1802, nom. superfl. & illeg. for *Panicumdactylon*

**Distribution.** Andaman and Nicobar Islands, Andhra Pradesh, Bihar, Chhattisgarh, Delhi, Goa, Gujarat, Himachal Pradesh, Jharkhand, Karnataka, Kerala, Lakshadweep, Madhya Pradesh, Maharashtra, Odisha, Rajasthan, Tamil Nadu, Tripura, Uttar Pradesh, West Bengal [Bangladesh, China, Myanmar]. Cosmopolitan; native range unknown.

**Habit. **Perennial.

**1364. **Cynodon plectostachyus** (K. Schum.) Pilg., Bot. Jahrb. Syst. 40: 82. 1907 (as “plectostachyum”) **

*Leptochloa plectostachya* K. Schum., Pflanzenw. Ost-Afrikas C. 112. 1895. Type: Tanzania “Steppen sudlich vom Kilimandscharo, sehr haufiges Gras in den Steppen am Pangani”, alt. 2500 ft.; July 2, 1893; *G. Volkens 477* (B).

**Distribution. **Tamil Nadu. Naturalized; native to Africa.

**Habit. **Perennial.

**1365. **Cynodon radiatus** Roth, Syst. Veg., ed. 15 bis [Roemer & Schultes] 2: 411. 1817 **

*Cynodon arcuatus* J. Presl in C. Presl, Reliq. Haenk. 1(4–5): 290. 1830. Type: “Habitat in Luzonia.” Philippines: Luzon Island; *Haenke s.n.* (PR).

*Cynodon intermedius* Rang. & Tadul., J. Bombay Nat. Hist. Soc. 26: 304. f. 1. 2. 1918. Syntypes: India: Godavari District, Gokavaram; *Anonymous 8262*. Chingleput; *Anonymous* 11488. Tinnevelly District; *Anonymous* 13129, 13259. Kallar on the Nilgiris; *Rangachari & Tadulingham 13988*

*Cynodon dactylon* (L.) Pers. var. *intermedius* (Rang. & Tadul.) C.E.C. Fisch., Fl. Madras 3(10): 1835. 1934

**Type.** India; 1814; *B. Heyne s.n.* (L).

**Distribution. **Andaman and Nicobar Islands, Andhra Pradesh, Assam, Bihar, Karnataka, Kerala, Madhya Pradesh, Maharashtra, Nagaland, Odisha, Tamil Nadu, Uttar Pradesh, West Bengal [Bhutan, China, Myanmar, Nepal (Naithani), Pakistan, Sri Lanka].

**Habit.** Annual.

**Remarks. **Most records from India fide Naithani (1990).

***Dactyloctenium* Willd., Enum. Pl. (Willdenow) 2: 1029. 1809**

**Lectotype. ***Dactyloctenium aegyptium* (L.) Willd. (“aegyptiacum”) (*Cynosurus aegyptius* L.). LT designated by Pfeiffer, Nomencl. Bot. 1: 1000. 1873.

**1366. **Dactyloctenium aegyptium** (L.) Willd., Enum. Pl. (Willdenow) 2: 1029. 1809 (as “aegyptiacum”) **

*Cynosurus aegyptius* L., Sp. Pl. 1: 72. 1753. Type: “Habitat in Africa, Asia, America.” Lectotype: *Herb. Linn. No. 91.11*(LINN). LT designated by Kit Tan in Davis (ed.), Fl. Turkey 9: 578. 1985.

*Eleusine aegyptia* (L.) Forsyth f., Bot. Nomencl. 70. 1794

*Chloris mucronata* Michx., Fl. Bor.-Amer. (Michaux) 1: 59. 1803. Type: USA; *Michaux s.n.* (P). “Hab. in cultis Carolinae.”

*Dactyloctenium mucronatum* (Michx.) Willd., Enum. Pl. (Willdenow) 2: 1029. 1809

*Eleusine pectinata* Moench, Suppl. Meth. (Moench) 68. 1802, nom. superfl. & illegit. for *Cynosurus aegyptius*

**Distribution.** Andhra Pradesh, Assam, Bihar, Daman and Diu, Chhattisgarh, Delhi, Goa, Gujarat, Jammu and Kashmir, Himachal Pradesh, Karnataka, Kerala, Lakshadweep, Madhya Pradesh, Maharashtra, Odisha, Rajasthan, Tamil Nadu, Tripura, Uttar Pradesh, Uttarakhand, West Bengal [Myanmar, Sri Lanka].

**Habit. **Annual.

**1367. **Dactyloctenium aristatum** Link, Hort. Berol. [Link] 1: 59. 1827 **

**Type.** “Hab. in Aegypto; *Eherenb. s.n.*”

**Distribution.** Andhra Pradesh, Bihar, Chhattisgarh, Daman and Diu, Goa, Madhya Pradesh, Maharashtra, Rajasthan, Tamil Nadu, Uttar Pradesh, Uttarakhand [Pakistan].

**Habit.** Annual.

**Remarks.** Record for Tamil Nadu fide Naithani (1990).

**1368. **Dactyloctenium australe** Steud., Syn. Pl. Glumac. 1: 212. 1854 **

**Type.** “Afr. austr.”

**Distribution.** Maharashtra. Naturalized; native to Africa.

**Habit.** Perennial.

**1369. **Dactyloctenium scindicum** Boiss., Diagn. Pl. Orient. ser. 2, 3(4): 131. 1859 **

*Eleusine scindica* (Boiss.) Duthie, Fodder Grasses North. India. 58. 1888

*Dactyloctenium glaucophyllum* Courbai, Ann. Sci. Nat., Bot. Ser. 4, 18: 133. 1862. Type: “In locis siccis, sabulosis.”

*Eleusine glaucophylla* (Courbai) Munro ex Benth., J. Linn. Soc., Bot. 19: 107. 1881

**Type.** “Hab. in ditione Scinde cl. Griffith No. 637.”

**Distribution.** Daman and Diu, Delhi, Gujarat, Haryana, Madhya Pradesh, Maharashtra, Punjab, Rajasthan, Uttar Pradesh [Pakistan].

**Habit.** Perennial.

**Remarks. **Records in India fide Naithani, except for Maharashtra, Punjab.

***Desmostachya* (Stapf) Stapf, Fl. Cap. (Harvey) 7: 316. 1898; 632. 1900**

*Eragrostis* sect. *Desmostachya* Stapf, Fl. Brit. India (J.D. Hooker) 7: 324. 1896.

**Lectotype. ***Desmostachya bipinnata* (L.) Stapf (*Briza bipinnata* L.).

**1370. **Desmostachya bipinnata** (L.) Stapf, Fl. Cap. (Harvey) 7: 632. 1900 **

*Briza bipinnata* L., Fl. Palaest. 12. 1756. Type: “Habitat in Herjil. Aegypt. Aegypt. frequ. Palaest. rar.” Lectotype: Hasselquist, Herb. Linn. No. 89.2 (LINN). LT designated by Danin in Cafferty et al. (ed.), Taxon 49: 248. 2000.

*Leptochloa bipinnata* (L.) Hochst., Flora 38: 422. 1855

*Pogonarthria bipinnata* (L.) Chiov., Ann. Bot. (Rome) 8(3): 362. 1908

*Stapfiola bipinnata* (L.) Kuntze in T. Post & Kuntze, Lex. Gen. Phan. 532. 1903 (“1904”)

*Uniola bipinnata* (L.) L. Sp. Pl., ed. 2. 1: 104. 1762.

*Poa cynosuroides* Retz., Observ. Bot. (Retzius) 4: 20. 1786–1787. Type: “Habitat in aridis sterilioribus I. Or. Cel. Konig.”

*Eragrostis cynosuroides* (Retz.) P. Beauv., Ess. Agrostogr. 174. 1812

*Cynosurus durus* Forssk., Fl. Aegypt.-Arab. 21. 1775, nom. later hom. & illeg., non L., 1753. Type: “In hortis Kahirinis spontaneus. Arab Hhalfe.”

**Distribution.** Andhra Pradesh, Assam, Bihar, Chhattisgarh, Daman and Diu, Goa, Gujarat, Haryana, Himachal Pradesh, Jammu and Kashmir, Karnataka, Maharashtra, Odisha, Punjab, Rajasthan, Tamil Nadu, Uttar Pradesh, West Bengal [China, Myanmar, Nepal, Pakistan].

**Habit.** Rhizomatous perennial.

***Dignathia* Stapf, Hooker’s Icon. Pl. 30: sub t. 2950. 1911**

**Lectotype. ***Dignathia gracilis* Stapf.

**1371. **Dignathia hirtella** Stapf, Hooker’s Icon. Pl. 30: t. 2950. f. 13. 1911 **

**Type.** British East Africa: Without precise locality; *Linton 4* (cum *Latipede senegalensi* Kunth).

**Distribution.** Gujarat, Rajasthan. Native; also in Africa.

**Habit.** Annual.

**Remarks.** Distribution in India fide Naithani (1990).

***Dinebra* Jacq., Fragm. Bot. 77. 1809**

**Type. ***Dinebra arabica* Jacq., nom. illeg. (*Cynosurus retroflexus* Vahl, *Dinebra retroflexa* (Vahl) Panz.).

**1372. **Dinebra chinensis** (L.) P.M. Peterson & N. Snow, Ann. Bot. 109: 1326. 2012 **

*Poa chinensis* L., Sp. Pl. 1: 69. 1753. Type: “Habitat in India. Osbeck.” Lectotype: *Osbeck s.n., Herb. Linn. No. 87.32* (LINN). LT designated by Phillips in Polhill (ed.), Fl. Trop. E. Africa, Gramineae 2: 279. 1974

*Leptochloa chinensis* (L.) Nees, Syll. Pl. Nov. 1: 4. 1824

*Poa malabarica* sensu Retz., Observ. Bot. (Retzius) 5: 19. 1788 (“1789”), non L., 1753

**Distribution.** Andhra Pradesh, Assam, Bihar, Chhattisgarh, Gujarat, Himachal Pradesh, Karnataka, Kerala, Madhya Pradesh, Maharashtra, Odisha, Tamil Nadu, Uttar Pradesh, West Bengal [Bhutan, China, Myanmar, Sri Lanka].

**Habit.** Annual.

**1373. **Dinebra polystachyos** (R. Br.) E.A. Kellogg, comb. nov. **

urn:lsid:ipni.org:names:77212134-1

*Cynodon polystachyos* R. Br., Prodr. Fl. Nov. Holland. 1: 187. 1810 (as “polystachyon”). Type: Australia: Northern Territory: Darwin & Gulf Distr.: Maria Island, Gulf of Carpenteria. *R. Brown 6238*.

*Cynodon neesii* Thwaites, Enum. Pl. Zeyl. (Thwaites). 371. 1864. Type: India; *Rottler s.n., Wight s.n.* Sri Lanka: Trincomalee; *Thwaites Ceylon Plant 3749*

*Leptochloa neesii* (Thwaites) Benth., J. Linn. Soc., Bot. 19: 108. 1881

*Dinebra neesii* (Thwaites) P.M. Peterson & N. Snow, Ann. Bot. 109: 1326. 2012

*Leptochloa polystachyos* (R. Br.) Benth., Fl. Austral. 7: 617–618. 1878, non *L. polystachya* (Michx.) Kunth, 1829

*Cynodon virgatus* Nees ex Steud., Syn. Pl. Glumac. 1: 213. 1854, non (L.) Raspail, 1825

**Distribution.** Andhra Pradesh, Tamil Nadu [Myanmar, Sri Lanka].

**Habit.** Annual or perennial.

**Remarks. **The above combination is made here to transfer *Cynodon polystachyos* R. Br. to *Dinebra*.

**1374. **Dinebra panicea** (Retz.) P.M. Peterson & N. Snow, Ann. Bot. 109: 1326. 2012 **

*Poa panicea* Retz., Observ. Bot. (Retzius) 3: 11. 1783. Type: “E China misit Honoratiss. Bladh.” China: Honoratiss; *Bladh s.n.* (LD).

*Leptochloa panicea* (Retz.) Ohwi, Bot. Mag. (Tokyo) 55: 311. 1941

*Aira filiformis* J. Koenig ex Roxb., Fl. Ind. (Carey and Wallich ed.) 1: 328. 1820. Type: India: “A native of pasture ground.”

*Leptochloa filiformis* sensu Hook. f., Fl. Brit. India (J.D. Hooker). 7(22): 298. 1896, non (Pers.) P. Beauv., 1812

**Distribution.** Andhra Pradesh, Bihar, Chhattisgarh, Delhi, Gujarat, Madhya Pradesh, Maharashtra, Odisha, Tamil Nadu, Uttar Pradesh, Uttarakhand [China, Sri Lanka].

**Habit.** Annual.

**1375. **Dinebra retroflexa** (Vahl) Panz., Ideen Revis. Gräs. 59, 60. 1813 var. **retroflexa

*Cynosurus retroflexus* Vahl, Symb. Bot. (Vahl) 2: 20. 1791. Type: “Habitat in India Orientali. Dn. La Mark.” India: common weed of cultivation; *La Mark s.n. *(C).

*Dinebra arabica* Jacq., Fragm. Bot. 77, 98. t. 121. f. 1. 1807. Type: Cultivated at the Vienna Botanical Garden.

*Leptochloa arabica* (Jacq.) Kunth, Révis. Gramin. 1: 91. 1829

*Eleusine calycina* Roxb., Fl. Ind. (Carey and Wallich ed.) 1: 347. 1820. Type: India: “Grows in small tufts on dry pasture ground, but generally amongst bushes.”

**Distribution.** Andhra Pradesh, Bihar, Chhattisgarh, Dadra and Nagar Haveli, Goa, Gujarat, Karnataka, Madhya Pradesh, Maharashtra, Odisha, Punjab, Rajasthan, Tamil Nadu [China, Pakistan].

**Habit.** Annual.

**1376. **Dinebra retroflexa** (Vahl) Panz. var. **condensata** S.M. Phillips, Kew Bull. 28(3): 413–414. 1973 **

**Type.** Tanzania; *Procter 3209* (K).

**Distribution.** Andhra Pradesh, Bihar, Goa, Gujarat, Jammu and Kashmir, Karnataka, Madhya Pradesh, Maharashtra, Odisha, Punjab, Rajasthan, Tamil Nadu, Uttar Pradesh [Afghanistan, Pakistan, Sri Lanka].

**Habit.** Annual.

***Diplachne* P. Beauv., Ess. Agrostogr. 80, pl. 16, f. 9. 1812**

**Type. ***Diplachne fascicularis* (Lam.) P. Beauv. (*Festuca fascicularis* Lam.; *Diplachne fusca* ssp. *fasciculata* (Lam.) P.M. Peterson & N. Snow).

**1377. **Diplachne fusca** (L.) P. Beauv., Syst. Veg. 2: 615. 1817 **

*Festuca fusca* L., Syst. Nat., ed. 10. 2: 876. 1759. Type: “Habitat [in Palaestina.] Sp. Pl., ed. 2. 1: 109 (1762)” Lectotype: Hasselquist, Herb. Linn. No. 92.21 (LINN). LT designated by Phillips in Polhill (ed.), Fl. Trop. E. Africa, Gramineae 2: 281. 1974.

*Leptochloa fusca* (L.) Kunth, Révis. Gramin. 1: 91. 1829

*Uralepis fusca* (L.) Steud., Syn. Pl. Glumac. 1: 247. 1854

*Leptochloa malabarica* (L.) Veldkamp, Blumea 19(1): 64. 1971

*Poa malabarica* L., Sp. Pl. 1: 69. 1753, nom. rej. Type: “Habitat in India arenosis.” Lectotype = “Tsjama-pullu” in Rheede, Hort. Malab., 12: 83. t. 45. 1693. LT designated by Merrill in Bull. Torrey Bot. Club 60: 635. 1933.

*Diplachne malabarica* (L.) Merr., Bull. Torrey Bot. Club 60: 635. 1933

*Poa procera* Roxb., Fl. Ind. (Carey and Wallich ed.) 1: 334. 1820. Type: India

**Distribution.** Andhra Pradesh, Bihar, Delhi, Gujarat, Karnataka, Kerala, Madhya Pradesh, Maharashtra, Odisha, Tamil Nadu, West Bengal [China, Myanmar, Pakistan, Sri Lanka].

**Habit. **Perennial.

***Disakisperma* Steud., Syn. Pl. Glumac. 1: 287. 1854**

**Type. ***Disakisperma mexicanum* Steud. (“mexicana”) (*Disakisperma dubia* (Kunth) P.M. Peterson & N. Snow).

**1378. **Disakisperma obtusiflorum** (Hochst) P.M. Peterson & N. Snow, Ann. Bot. 109: 1327. 2012 (as “obtusiflora”) **

*Leptochloa obtusiflora* Hochst., Flora 38: 203. 1855. Type: Abyssinia [Ethiopia]; *Schimper Hb. Buch. nr. 1204* (STR)

*Eleusine obtusiflora* (Hochst.) Blatt., Rec. Bot. Surv. India 8: 505. 1936

**Distribution.** Kerala, Tamil Nadu [also in Saudi Arabia, Africa].

**Habit.** Perennial.

***Eleusine* Gaertn., Fruct. Sem. Pl. 1: 7. 1788**

**Lectotype. ***Eleusine coracana* (L.) Gaertn. (*Cynosurus coracanus* L.). LT designated by Nash in Britton & Brown, Ill. Fl. N. U.S., ed. 2, 1: 228. 1913; affirmed by Hitchcock, Bull. U.S.D.A. 772: 175. 1920.

**1379. **Eleusine africana** Kenn.-O’Byrne, Kew Bull. 12:65. 1957 **

**Type.** South Africa: Cape Province, Kimberley Dist.: Warrenton-on-Waal, Mar 1950, *M. Wilman H.K.*1 (K; IT: US-2589173).

**Distribution.** Madhya Pradesh. Naturalized; native to Africa.

**Habit.** Annual.

**Remarks.** Record for Madhya Pradesh fide Naithani (1990).

**1380. **Eleusine coracana** (L.) Gaertn., Fruct. Sem. Pl. 1: 8. t. 1. f. 11. 1788 **

*Cynosurus coracanus* L., Syst. Nat., ed. 10. 2: 875. 1759. Type: “Habitat [in India.] Sp. Pl., ed. 2. 1: 107. 1762.” Lectotype = “Gramen dactylon Orientale, majus, frumentaceum, semine Napi” in Plukenet, Phytographia t. 91. f. 5. 1691. Typotype: Herb. Sloane 96: 74 (BM-SL). Voucher: Herb. Sloane 93: 189 (BM-SL). Typotype designated by Phillips in Kew Bull. 27: 254. 1972.

**Distribution.** Andaman and Nicobar Islands, Andhra Pradesh, Arunachal Pradesh, Assam, Bihar, Chhattisgarh, Delhi, Gujarat, Himachal Pradesh, Jammu and Kashmir, Jharkhand, Karnataka, Kerala, Madhya Pradesh, Maharashtra, Meghalaya, Odisha, Rajasthan, Sikkim, Tamil Nadu, Tripura, Uttar Pradesh, Uttarakhand, West Bengal [China]. Cultivated.

**Habit.** Annual.

**1381. **Eleusine indica** (L.) Gaertn., Fruct. Sem. Pl. 1: 8. 1788 **

*Cynosurus indicus* L., Sp. Pl. 1: 72–73. 1753. Type: “Habitat in Indiis.” Lectotype = “Gramen Dactyloides spicis deorsum aristatis” in Burman, Thes. Zeylan., 106. t. 47. f. 1. 1737. LT designated by Phillips in Cafferty et al. (ed.), Taxon 49: 249. 2000. Epitype: Sri Lanka: Central Province, Matale District, 5 miles South of Matale on Kandy Road; 1970; *Clayton 5330 *(K). Epitype designated by Phillips in Cafferty et al. (ed.), Taxon 49: 249. 2000.

**Distribution.** Andaman and Nicobar Islands, Andhra Pradesh, Arunachal Pradesh, Assam, Bihar, Delhi , Dadra and Nagar Haveli, Goa, Gujarat, Himachal Pradesh, Jammu and Kashmir, Karnataka, Kerala, Lakshadweep, Maharashtra, Madhya Pradesh, Manipur, Meghalaya, Nagaland, Odisha, Rajasthan, Sikkim, Tamil Nadu, Tripura, Uttar Pradesh, West Bengal [China, Myanmar, Nepal, Sri Lanka].

**Habit.** Annual.

**Remarks.** Cosmopolitan; native to Africa and Asia.

***Enteropogon* Nees, Nat. Syst. Bot., ed. 2 448. 1836**

**Type. ***Enteropogon melicoides* (J. Koenig ex Willd.) Nees (*Ischaemum melicoides* J. Koenig ex Willd.).

**1382. **Enteropogon dolichostachyus** (Lag.) Keng ex Lazarides, Austral. J. Bot., Suppl. Ser. 5: 31. 1972 **

*Chloris dolichostachya* Lag., Gen. Sp. Pl. (Lagasca) 5. 1816. Type: Philippines: “in Phillip insulis D. Ludovicus *Nee* legit.” (MA).

*Melica digitata* Roxb., Fl. Ind. (Carey and Wallich ed.) 1: 328. 1820. Type: India: “A native of hedges and thickets.”

*Chloris digitata* (Roxb.) Steud., Syn. Pl. Glumac. 1: 207. 1854

*Chloris incompleta* Roth, Syst. Veg., ed. 15 bis [Roemer & Schultes]. 2: 607. 1817. Type: India; *Heyne s.n.*

*Lophacme incompleta* (Roth) Chiov., Ann. Bot. (Rome) 8(3): 350. 1908

**Distribution.** Andhra Pradesh, Bihar, Chhattisgarh, Delhi, Gujarat, Himachal Pradesh, Karnataka, Kerala, Madhya Pradesh, Maharashtra, Odisha, Rajasthan, Tamil Nadu, Uttarakhand [Afghanistan, Bhutan, China, Myanmar, Nepal, Pakistan, Sri Lanka].

**Habit.** Caespitose perennial.

**1383. **Enteropogon monostachyos** (Vahl) K. Schum., Abh. Konigl. Akad. Wiss. Berlin 17. 1894 **

*Cynosurus monostachyos* Vahl, Symb. Bot. (Vahl) 2: 20. 1791. Type: “Habitat in India Orientali. Dn. Koenig.”

*Leptochloa monostachyos* (Vahl) P. Beauv. Ex Roem. & Schult., Syst. Veg., ed. 15 bis [Roemer & Schultes] 2: 581. 1817 (as “monostachya”)

*Enteropogon badamicus* Bhide, J. Proc. Asiat. Soc. Bengal (n. s.) 7(8): 517. 1912 (“1911”). Type: India: Bombay, Badami; September, 1909; *R.K. Bhide s.n.* (as “badamicum”)

*Ischaemum melicoides* J. Koenig ex Rottler, Ges. Naturf. Freunde Berlin Neue Schriften 4: 211. 1803

*Enteropogon melicoides* (J. Koenig ex Rottler) Nees in Lindley, Nat. Syst. Bot. ed. 2. 449. 1836

*Rottboellia pilosa* Roth, Syst. Veg., ed. 15 bis [Roemer & Schultes] 2: 785. 1817. Type: India; *B. Heyne s.n.*

*Rottboellia tricantha* Roth, Nov. Pl. Sp. 43. 1821

*Enteropogon coimbatorensis* K.K.N. Nair, S.K. Jain, & M.P. Nayar, Proc. Indian Acad. Sci., B 86(2): 84, t. 1a-g. 1977. Type: India: Tamil Nadu, Coimbatore District, Kuridimalai; sd.; *K. Subramanyam 1828* (MH). Type: India: “veneratiss”; *B. Heyne s.n.*

**Distribution.** Andhra Pradesh, Karnataka, Kerala, Maharashtra, Tamil Nadu [Myanmar, Sri Lanka, Africa].

**Habit.** Caespitose perennial.

***Eragrostiella* Bor, Indian Forester 66: 269. 1940**

**Type. ***Eragrostiella leioptera* (Stapf) Bor (*Eragrostis leioptera* Stapf).

**1384. **Eragrostiella bifaria** (Vahl) Bor, Indian Forester 66(5): 270. 1940 var. **bifaria

*Poa bifaria* Vahl, Symb. Bot. (Vahl) 2: 19. 1791. Type: “Habitat in India Orientali.”

*Eragrostis bifaria* (Vahl) Steud., Syn. Pl. Glumac. 1: 264. 1854

*Poa coromandelina* J. Koenig ex Rottler., Ges. Naturf. Freunde Berlin Neue Schriften 4: 191. 1803.

*Eragrostis coromandelina* (J. Koenig ex Rottler) Trin., Mém. Acad. Imp. Sci. St.-Pétersbourg, Sér. 6, Sci. Math. 1: 415. 1830

*Catapodium bifarium* Link, Hort. Berol. [Link] 2: 194. 1833 (as “bifario”), nom. nud., in obs.

*Catapodium coromandelianum* Link, Hort. Berol. [Link] 2: 194. 1833 (as “coromandeliano”), nom. nud., in obs.

**Distribution. **Andhra Pradesh, Bihar, Chhattisgarh, Delhi, Haryana, Gujarat, Karnataka, Kerala, Madhya Pradesh, Maharashtra, Odisha, Rajasthan, Tamil Nadu [Myanmar, Sri Lanka, Africa].

**Habit.** Perennial.

**1385. **Eragrostiella bifaria** (Vahl) Bor var. **walkeri** (Stapf) Lazarides, Contr. Herb. Austral. 22: 7. 1976 **

*Eragrostis walkeri* Stapf in Trimen, Handb. Fl. Ceylon 5: 298. 1900. Syntypes: Sri Lanka; *Walker s.n.* (K). Sri Lanka: Central Province, Galagama, below Horton plains; *Thwaites s.n.* Sri Lanka: Western province, Kurunegala; *Trimen s.n*

*Eragrostiella walkeri* (Stapf) Bor, Indian Forester 66(5): 270. 1940

**Distribution.** Andhra Pradesh, Bihar, Delhi, Haryana, Karnataka, Kerala, Madhya Pradesh, Maharashtra, Rajasthan, Tamil Nadu [Myanmar, Sri Lanka].

**Habit.** Perennial.

**Remarks.** Record for Tamil Nadu fide Naithani (1990).

**1386. **Eragrostiella brachyphylla** (Stapf) Bor, Indian Forester 66(5): 270. 1940 **

*Eragrostis brachyphylla* Stapf, Fl. Brit. India (J.D. Hooker). 7(22): 327. 1896. Type: India: Behar and the Central Provinces, Nilghiris; *Wight, Perrottet*.

**Distribution.** Andhra Pradesh, Bihar, Chhattisgarh, Gujarat, Himachal Pradesh, Karnataka, Madhya Pradesh, Maharashtra, Odisha, Rajasthan, Tamil Nadu, West Bengal [Bangladesh, Sri Lanka].

**Habit.** Perennial.

**1387. **Eragrostiella leioptera** (Stapf) Bor, Indian Forester 66(5): 270. 1940 **

*Eragrostis leioptera* Stapf, Fl. Brit. India (J.D. Hooker). 7(22): 325. 1896. Type: India: Khasia Hills, alt. 4000–5000 ft.; *Griffith s.n.*

**Distribution.** Assam, Madhya Pradesh, Meghalaya.

**Habit.** Perennial.

**1388. **Eragrostiella nardoides** (Trin.) Bor, Indian Forester 66(5): 270. 1940 **

*Eragrostis nardoides* Trin., Mém. Acad. Imp. Sci. St.-Pétersbourg, Sér. 6, Sci. Math. 1(4): 415. 1830. Type: “V. spp. Nepal.” Nepal; *Wallich* (Numer. List [Wallich] no. 3827F) (LE).

*Catapodium nepalense* Link, Hort. Berol. [Link] 2: 194. 1833. Type: “Habiat in Nepalia.”

**Distribution. **Bihar, Chhattisgarh, Madhya Pradesh, Punjab, Rajasthan, Uttar Pradesh [Nepal].

**Habit.** Perennial.

***Gymnopogon* P. Beauv., Ess. Agrostogr. 41, 164. 1812**

**Type. ***Gymnopogon racemosus* P. Beauv., nom. illeg*.* (“racemosa”) (*Andropogon ambiguus* Michx., *Gymnopogon ambiguus* (Michx.) Britton, Sterns & Poggenb.).

**1389. **Gymnopogon delicatulus** (C.B. Clarke ex Hook. f.) Bor, Grasses Burma, Ceylon, India & Pakistan 472. 1960 **

*Chloris delicatula* C.B. Clarke ex Hook. f., Fl. Brit. India (J.D. Hooker). 7(22): 290. 1896. Syntypes: India: Chota Nagpore, Mandu, Hazarabagh; *C.B. Clarke 33855.* Pegu-Yomah; *Kurz s.n.*

**Distribution.** Jharkhand, Odisha [Mynamar].

**Habit.** Annual.

***Halopyrum* Stapf, Hooker’s Icon. Pl. 25: t. 2448. 1896**

**Type. ***Halopyrum mucronatum* (L.) Stapf (*Uniola mucronata* L.).

**1390. **Halopyrum mucronatum** (L.) Stapf, Hooker’s Icon. Pl. 25: t. 2448. 1896 **

*Uniola mucronata* L., Sp. Pl., ed. 2. 1: 104. 1762. Type: “Habitat in India. Burmannus.” Neotype: Sri Lanka: Wilpatti National Park; December 30, 1968; *Fosberg & al. 50918 *(K). LT designated by Renvoize in Cafferty et al. (ed.), Taxon 49: 258. 2000.

*Desmazeria unioloides* Deflers, Voy. Yemen. 220. 1889, nom. superfl. & illeg. for *Uniolamucronata* L.

*Eragrostis mucronata* (L.) Deflers, Bull. Soc. Bot. France 34: 69. 1887, non *Eragrostis mucronata* Roem. & Schult., 1817

**Distribution.** Delhi, Gujarat, Maharashtra, Rajasthan, Tamil Nadu [Pakistan, Sri Lanka].

**Habit.** Perennial.

***Hilaria* Kunth, Nov. Gen. Sp. [H.B.K.] 1(1–2): 116 (ed. qto.). t. 37. 1816 (“1815”)**

**Type. ***Hilaria cenchroides* Kunth.

**1391. **Hilaria jamesii** (Torr.) Benth., J. Linn. Soc., Bot. 19: 62. 1881 **

*Pleuraphis jamesii* Torr., Ann. Lyceum Nat. Hist. New York 1(1): 148–150, t. 10. 1824. Type: “Habitat on the high plains of the trap formation at the sources of the Canadian river.” USA: Texas or New Mexico, sources of the Canadian River; 1852; *James s.n. *(NY).

*Pleuraphis sericea* Nutt. ex Benth., J. Linn. Soc., Bot. 19: 62. 1881, nom. nud. as synonym of *Hilaria sericea* Benth.

**Distribution.** India. Native to North America.

**Habit.** Perennial.

**Remarks.** The report of this species for India is dubious; documentation is lacking.

***Leptochloa* P. Beauv., Ess. Agrostogr. 71. 1812**

**Lectotype.***Leptochloa virgata* (L.) P. Beauv. (*Cynosurus virgatus* L.). LT designated by Nash in N.L. Britton & A. Brown, Ill. Fl. N. U.S. ed. 2. 1: 229. 1913; affirmed by Hitchcock, Bull. U.S.D.A. 772: 172. 1920.

**1392. **Leptochloa crinita** (Lag.) P.M. Peterson & N. Snow, Ann. Bot. 109: 1327. 2012 (as “crinata”) **

*Chloris crinita* Lag., Varied. Ci. 4: 143. 1805. Type: Philippines: “Ex Philippinis Insulis” [from Nee letter] [error for Mexico]; *Nee s.n.* (MA).

*Chloropsis crinita* (Lag.) Kuntze, Revis. Gen. Pl. 2: 771. 1891

*Trichloris crinita* (Lag.) Parodi, Revista Argent. Agron. 14: 63. 1947

*Chloris mendocina* Phil., Anales Univ. Chile 36: 208- 209. 1870. Type: Chile: Mendoza (P).

*Chloropsis mendocina* (Phil.) Kuntze, Revis. Gen. Pl. 3(3): 348. 1898

*Trichloris mendocina* (Phil.) Kurtz, Mem. Fac. Ci. Exact. Fic. Nat. Univ. Nac. Cordoba 1896: 36. 1897

*Trichloris fasciculata* E. Fourn., Mex. Pl. 2: 142. 1886. Type: Mexico: San Luis de Potosi; *Virlett 1440 *(P).

*Chloropsis fasciculata* (E. Fourn.) Kuntze, Revis. Gen. Pl. 2: 771. 1891

*Trichloris blanchardiana* E. Fourn. ex Scribn., Bull. Torrey Bot. Club 9: 146. 1882. Type: USA: Arizona, Santa Cruz Valley, near Tucson; May 19, 1881; *C.G. Pringle 13921* (US).

*Leptochloris greggii* Munro ex Merr., U.S.D.A. Div. Bot. Circ. 32: 7. 1901, nom. nud., pro. syn.

*Leptochloris crinita* Munro ex Kuntze, Revis. Gen. Pl. 2: 771. 1891, nom. nud.

*Chloris trichodes* Lag. ex Parodi, Revista Argent. Agron. 14: 62. 1947, nom. nud., pro. syn

**Distribution.** India; individual states not recorded. Naturalized; native to North and South America.

**Habit.** Perennial.

**1393. **Leptochloa pluriflora** (E. Fourn.) P.M. Peterson & N. Snow, Ann. Bot. 109: 1327. 2012 **

*Trichloris pluriflora* E. Fourn., Mex. Pl. 2: 142. 1886. Lectotype: USA: Texas, inter Laredo et Bejar; *Berlandier 1430* (P). LT designated by Hitchcock in Contr. U.S. Natl. Herb. 17: 335. 1917.

*Chloropsis pluriflora* (E. Fourn.) Kuntze, Revis. Gen. Pl. 2: 771. 1891

**Distribution.** India; individual states not recorded. Naturalized; native to North and South America.

**Habit.** Perennial.

***Lepturus* R. Br., Prodr. Fl. Nov. Holland. 207. 1810**

**Type. ***Lepturus repens* (G. Forst.) R. Br. (*Rottboellia repens* G. Forst.).

**1394. **Lepturus radicans** (Steud.) A. Camus, Ann. Soc. Linn. Lyon ser. 2, 69: 87. 1923 **

*Ophiuros radicans* Steud., Syn. Pl. Glumac. 1: 430. 1855. Type: Nossibe Insula; *Boivin 1979, 1980*

**Distribution.** Karnataka [Sri Lanka, Africa].

**Habit.** Perennial.

**Remarks.** Distribution in India fide Naithani (1990).

**1395. **Lepturus repens** (G. Forst.) R. Br., Prodr. Fl. Nov. Holland. 1: 207. 1810 **

*Rottboellia repens* G. Forst., Fl. Ins. Austr. 9. 1786. Type: “Insula intra tropicos.”

*Monerma repens* (G. Forst.) P. Beauv., Ess. Agrostogr. 168, 177. 1812

**Distribution.** Lakshadweep [Sri Lanka, Africa, Australia].

**Habit.** Perennial.

***Melanocenchris* Nees, Proc. Linn. Soc. London 1: 94. 1841**

**Type.***Melanocenchris rothiana* Nees (*Pommereulla monoica* Roth, non Rottler, 1803).

**1396. **Melanocenchris abyssinica** (R. Br. ex Fresen.) Hochst., Flora 38: 274. 1855 **

*Eutriana abyssinica* R. Br. ex Fresen., Mus. Senckenberg. 2: 142. 1837. Type: Abyssinia; *Rueppell s.n.* (FR).

*Melanocenchris plumosa* Jaub. & Spach, Ill. Pl. Orient. 4: 37. 1851. Type: *Pennisetum plumosum* Hochst. et Steud. in Schimperi plantis Arab no. 794

*Gracilea royleana* (Nees) Hook. f. var. *plumosa* (Jaub. & Spach) Hook. f., Fl. Brit. India (J.D. Hooker). 7(22): 284. 1896

*Ptiloneilema plumosum* (Jaub. & Spach) Steud., Syn. Pl. Glumac. 1: 201. 1854.

*Pennisetum plumosum* Hochst. ex Steud., Syn. Pl. Glumac. 1: 201. 1854, nom. nud. pro. syn.

**Distribution.** Gujarat, Maharashtra, Punjab, Rajasthan [Pakistan].

**Habit.** Annual.

**1397. **Melanocenchris jacquemontii** Jaub. & Spach, Ill. Pl. Orient. 4: 36. t. 325. 1851 **

*Melanocenchris royleana* Nees, Syn. Pl. Glumac. (Steudel) 1: 218. 1854. Type: India Orientali

*Gracilea royleana* (Nees) Hook. f., Fl. Brit. India (J.D. Hooker). 7(22): 284. 1896

**Type.** “India tam cistropica (in provincial Delhi) quam australiori (inter urbes Bombay et Poonah) legit *Jacquemont*.” (P).

**Distribution.** Andhra Pradesh, Bihar, Chhattisgarh, Dadra and Nagar Haveli, Daman and Diu, Delhi, Goa, Gujarat, Karnataka, Madhya Pradesh, Maharashtra, Odisha, Rajasthan, Tamil Nadu, Uttar Pradesh [Pakistan].

**Habit.** Annual.

**1398. **Melanocenchris monoica** (J. Koenig ex Rottler) C.E.C. Fisch., Fl. Madras 3(10): 1831. 1934 **

*Pommereulla monoica* J. Koenig ex Rottler, Ges. Naturf. Ferunde Berlin Neue Schriften 4: 218. 1803 (as “Pommereullia”). Type: India: In dry localities from the Kistna River to S. Arcot, Nilgiri Distict; *Perrotte s.n.*

*Melanocenchris perrottetii* Jaub. & Spach, Ill. Pl. Orient. 4: 38. t. 326. 1851. Type: India: Neligherry Mountains; 1840; *Perrottet s.n.* (P)

*Melanocenchris monoica* Roth, Nov. Pl. Sp. 33 (1821), non Rottler (1803)

*Melanocenchris rothiana* Nees, Proc. Linn. Soc. London 1: 95. 1841, nom. nov. for *Melanocenchrismonoica* Roth

*Gracilea nutans* J. Koenig ex Rottler, Ges. Naturf. Ferunde Berlin Neue Schriften 4: 218. 1803, cited as synonym of *Pommereulla monoica*

**Distribution.** Andhra Pradesh, Tamil Nadu [Sri Lanka].

**Habit.** Perennial.

***Microchloa* R. Br., Prodr. Fl. Nov. Holland. 1: 208. 1810**

**Type. ***Microchloa setacea* R. Br., nom. illeg. (*Nardus indica* L. f., *Microchloa indica* (L. f.) P. Beauv.).

**1399. **Microchloa indica** (L.f.) P. Beauv., Ess. Agrostogr. 168, Atlas 13, t. 20, fig. 8. 1812 **

*Nardus indica* L.f., Suppl. Pl. 105. 1782(“1781”). Type: “Habitat in Tranquebaria.” India; *Koenig s.n.*

*Microchloa setacea* R. Br., Prodr. Fl. Nov. Holland. 208. 1810, nom. superfl. & illegit. for *Nardus indica*

*Rottboellia setacea* Roxb., Pl. Coromandel 2: 17. t. 132. 1800, nom. superfl. & illeg.

**Distribution.** Andhra Pradesh, Bihar, Karnataka, Kerala, Madhya Pradesh, Maharashtra, Odisha, Tamil Nadu, West Bengal [China].

**Habit.** Annual.

**Remarks.** Worldwide in tropical habitats.

**1400. **Microchloa kunthii** Desv., Opusc. Sci. Phys. Nat. 75. 1831 **

*Paspalum tenuissimum* M.E. Jones, Contr. W. Bot. 18: 24. 1935. Type: Mexico: Baja California, On the prairie at the Laguna Mountains, alt. 6500 ft.; September 24, 1930; *M.E. Jones 27584* (POM).

*Microchloa elongata* R. Br., Numer. List [Wallich] no. 3807. 1831, nom. nud.

**Type.** “Habitat in America aequinoxialia.” Mexico: Hidalgo, Omitlan et Llano de las Tinaxas; *Humboldt & Bonpland s.n.* (P).

**Distribution.** Andhra Pradesh, Assam, Bihar, Meghalaya, Nagaland, Odisha, Tamil Nadu [Bhutan, China, Myanmar].

**Habit.** Perennial.

**Remarks.** Worldwide in tropical habitats.

***Muhlenbergia* Schreb., Gen. Pl., ed. 8[a] 44. 1789**

**Type. ***Muhlenbergia schreberi* J.F. Gmel., Syst. Nat., ed. 13[bis] 2: 171. 1791.

**1401. **Muhlenbergia duthieana** Hack., Oesterr. Bot. Z. 52: 11. 1902 **

*Muhlenbergia sylvatica* sensu Hook. f., Fl. Brit. India (J.D. Hooker). 7: 259. 1896, non (Torr.) Torr. ex A. Gray, 1834

**Type.** Pakistan: Hazara District; Panj Gali, alt 6900 ft.; *J.F. Duthie 7611* (K) and many syntypes.

**Distribution.** Jammu and Kashmir, Himachal Pradesh [China, Nepal, Pakistan].

**Habit.** Perennial.

**1402. **Muhlenbergia himalayensis** Hack. ex Hook. f., Fl. Brit. India (J.D. Hooker). 7(22): 259. 1896 **

**Type.** Western Himalaya, alt. 6000–7000 ft., Chamba to Simla; *Thomson s.n., Madden s.n.*

**Distribution.** Jammu and Kashmir, Himachal Pradesh, Maharashtra, Uttar Pradesh, Uttarakhand [Afghanistan, Bhutan, China, Nepal].

**Habit.** Perennial.

**1403. **Muhlenbergia huegelii** Trin., Mém. Acad. Imp. Sci. Saint-Pétersbourg, Sér. 6, Sci. Math. Seconde Pt. Sci. Nat. 6, 4(3–4): 293. 1841 **

*Muhlenbergia geniculata* Nees, Syn. Pl. Glumac. (Steudel) 1: 178. 1854. Type: Nepal.

*Muhlenbergia viridissima* Nees, Syn. Pl. Glumac. (Steudel) 1: 178. 1854. Type: India, *Royle 271*

**Type.** Himalaya: Massuri, *de Hugel s.n.*

**Distribution.** Assam, Jammu and Kashmir, Himachal Pradesh, Sikkim, Uttar Pradesh, Uttarakhand [Afghanistan, Bhutan, China, Nepal, Pakistan].

**Habit.** Perennial.

**1404. **Muhlenbergia mexicana** (L.) Trin., Gram. Unifl. Sesquifl. 189. 1824 **

*Agrostis mexicana* L., Mant. Pl. 31. 1767. Type: “Habitat in America calidiore. D. Jacquin.” Lectotype: *Herb. Linn. No. 84.33* (LINN). LT designated by Fernald in Rhodora 45: 225. t. 751. 1943.

*Podosemum mexicanum* (L.) Link, Hort. Berol. [Link] 1: 84. 1827

*Trichochloa mexicana* (L.) Trin., Fund. Agrost. (Trinius) 117. 1820

*Vilfa mexicana* (L.) P. Beauv., Ess. Agrostogr. 16, 148, 161. 1812

*Agrostis tenacissima* L. f., Suppl. Pl. 107. 1782(“1781”). Type: “Habitat in India Orientali.”

**Distribution.** Uttarakhand. Naturalized; native to North America.

**Habit.** Perennial.

***Orinus* Hitchc., J. Wash. Acad. Sci. 23: 136. 1933**

**Type. ***Orinus arenicola* Hitchc.

**1405. **Orinus thoroldii** (Stapf ex Hemsl.) Bor, Kew Bull. 1951: 454. 1952 (“1951”) **

*Diplachne thoroldii* Stapf ex Hemsl., J. Linn. Soc., Bot. 30: 121. 1894. Type: China: Tibet, alt. 16000 ft.; July-September, 1891; *Thorold 120* (K).

*Orinus arenicola* Hitchc., J. Wash. Acad. Sci. 23(3): 136. 1933. Type: India: Ladak, Kashmir, Tsaka, alt. 15000 ft.; July 18, 1931; *Walter Koelz 2365* (US-1535771)

**Distribution.** Jammu and Kashmir, Ladakh. [China, Nepal].

**Habit.** Perennial.

**Remarks.** Records for Jammu and Kashmir and Nepal fide Naithani (1990).

***Oropetium* Trin., Fund. Agrost. (Trinius) 98. 1820**

**Type. ***Oropetium thomaeum* (L. f.) Trin. (*Nardus thomaea* L. f.).

**1406. **Oropetium roxburghianum** (Steud.) S.M. Phillips, Kew Bull. 30(3): 469. 1975 **

*Lepturus roxburghianus* Steud., Syn. Pl. Glumac. 1: 357. 1854. Type: India Orientalis; *Roxburgh s.n.*

*Tripogon roxburghianus* (Steud.) Bhide, J. Proc. Asiat. Soc. Bengal (n. s.) 7(8): 515. 1912 (“1911”) [Basionym reference not given]

**Distribution.** Andhra Pradesh, Karnataka, Madhya Pradesh, Maharashtra, Rajasthan, Tamil Nadu.

**Habit.** Perennial.

**Remarks.** Tropical Asia.

**1407. **Oropetium thomaeum** (L.f.) Trin., Fund. Agrost. (Trinius) 98. t. 3. 1820 **

*Nardus thomaea* L.f., Suppl. Pl. 105. 1782 (“1781”). Type: “Habitat in Tranquebaria ad Montem Sancti Thomae.” India; *Koenig s.n.* (LINN).

*Rottboellia thomaea* (L.f.) Willd., Sp. Pl., ed. 4 [Willdenow] 1(1): 464. 1797

**Distribution.** Andhra Pradesh, Bihar, Chhattisgarh, Gujarat, Delhi, Haryana, Karnataka, Kerala, Madhya Pradesh, Maharashtra, Odisha, Punjab, Rajasthan, Tamil Nadu, Uttar Pradesh, West Bengal [Myanmar, Pakistan, Sri Lanka].

**Habit.** Caespitose annual.

**1408. **Oropetium villosulum** Stapf ex Bor, Kew Bull. 1949: 571. 1950 (“1949”) **

**Type. **India: Central Provinces, Nimar District, Humra; December 21, 1888; *J.F. Duthie 8523* (K).

**Distribution.** Bihar, Chhattisgarh, Karnataka, Madhya Pradesh, Maharashtra, Odisha, Rajasthan.

**Habit.** Annual.

**Remarks.** Records for Karnataka, Madhya Pradesh, Maharashtra and Odisha fide Naithani (1990).

***Perotis* Aiton, Hortus Kew. (W. Aiton) 1: 85 1789**

**Type. ***Perotis latifolia* Aiton, nom. illeg. (*Saccharum spicatum* L., *Perotis spicata *(L.) T. Durand & H. Durand).

**1409. **Perotis hordeiformis** Nees in Hook. & Arn., Bot. Beechey Voy. 6: 247–248. 1838 **

*Perotis birmanica* Gand., Bull. Soc. Bot. France 66(7): 301. 1920 (“1920”). Type: Birmania: Minbu; *Mokin 560*

*Perotis glabrata* Steud., Syn. Pl. Glumac. 1: 186. 1854. Type: Philippines; *H. Cuming 1399* (L).

**Type.** Himalaya Mountain; *Royle 280.*

**Distribution.** Rajasthan, Uttar Pradesh, West Bengal [China, Myanmar, Nepal, Pakistan, Sri Lanka].

**Habit.** Annual.

**1410. **Perotis indica** (L.) Kuntze, Revis. Gen. Pl. 2: 787. 1891 var. **indica

*Anthoxanthum indicum* L., Sp. Pl. 1: 28. 1753. Type: “Habitat in India.” Lectotype: *Herb. Hermann 1: 29, No. 25* (BM-000621332). LT designated by Clayton in Polhill (ed.), Fl. Trop. E. Africa, Gramineae 2: 395. 1974.

*Perotis patula* Nees in Hook. & Arn., Bot. Beechey Voy. 6: 247–248. 1838

*Perotis perottetii* Gand., Bull. Soc. Bot. France 66: 301. 1920 (“1919”). Type: India: Nilagiri Mountains; *Perrottet 1390*

*Saccharum spicatum* L., Sp. Pl. 1: 54. 1753. Type: “Habitat in Indiae petrosis.” Lectotype: “Tsjeria-kuren-pullu” in Rheede, Hort. Malab. 12: 117. t. 62. 1693. LT designated by Clayton in Polhill (ed.), Fl. Trop. E. Africa, Gramineae 2: 395. 1974.

*Perotis spicata* (L.) T. Durand & H. Durand, Syll. Fl. Congol. 628. 1909

*Perotis latifolia* Aiton, Hortus Kew. (W. Aiton) 1: 85. 1789, nom. superfl. & illeg.

*Perotis hordeiformis* sensu Trin., Mém. Acad. Imp. Sci. Saint-Pétersbourg, Sér. 6, Sci. Math., Seconde Pt. Sci. Nat. 6(2): 267. 1841, non Hook. & Arn. 1838

**Distribution.** Andhra Pradesh, Assam, Bihar, Chhattisgarh, Daman and Diu, Delhi, Goa, Himachal Pradesh, Karnataka, Kerala, Madhya Pradesh, Maharashtra, Odisha, Rajasthan, Tamil Nadu, Uttar Pradesh, West Bengal [Bhutan, China, Myanmar, Nepal, Sri Lanka].

**Habit.** Annual.

**1411. **Perotis indica** (L.) Kuntze var. **keelakaraiensis** P. Umam. & P. Daniel, J. Econ. Taxon. Bot. 23(3): 691. 1999 **

**Type. **India: Tamil Nadu, Ramanathapuram District, Keelakarai; December 15, 1995; *P. Daniel & P. Umamaheswari 106547* (CAL).

**Distribution.** Andhra Pradesh, Karnataka, Kerala, Maharashtra, Tamil Nadu.

**Habit.** Annual.

**1412. **Perotis ornithocephala** (Hook.) P.M. Peterson, Taxon 63(2): 284. 2014 **

*Holboellia ornithocephala* Hook., Bot. Misc. 2: 144. t. 76. 1831. Type: “In India orientali, apud montes Madurae; *Koenig s.n., Wight s.n.*, M.D.”

*Lopholepis ornithocephala* (Hook.) Steud., Syn. Pl. Glumac. 1: 112. 1854

**Distribution.** Kerala, Tamil Nadu [Sri Lanka].

**Habit.** Annual.

**Remarks.** Following Peterson et al. (2014a), *Lopholepis* is subsumed in *Perotis*.

***Pommereulla* Naezén, Nov. Gram. Gen. 31. 1779**

**Type. ***Pommereulla cornucopiae* L. f.

**1413. **Pommereulla cornucopiae** L.f., Suppl. Pl. 105. 1782 (“1781”) **

**Type. **“Habitat in India.”

**Distribution.** Andhra Pradesh, Karnataka, Tamil Nadu [Sri Lanka].

**Habit.** Perennial.

***Schoenefeldia* Kunth, Révis. Gramin. 1: 86. 1829**

**Type. ***Schoenefeldia gracilis* Kunth.

**1414. **Schoenefeldia gracilis** Kunth, Révis. Gramin. 1: 87. 1829; 1: 283. t. 53. 1830 **

*Chloris myosuroides* Hook. f., Fl. Brit. India (J.D. Hooker). 7(22): 290. 1896. Type: India: Gangetic Plain, ravines near Etawah; *Duthie s.n.*

*Schoenefeldia pallida* Edgew., J. Asiat. Soc. Bengal 21: 183. 1852

*Chloris pallida* (Edgew.) Hook. f., Fl. Brit. India (J.D. Hooker). 7(22): 289. 1896, non Willd., 1806. nom. illeg. hom.

**Type. **“Crescit in Senegalia.” Africa: Senegal, dry sandy places; *Kunth s.n.* (B).

**Distribution.** Bihar, Gujarat, Rajasthan [Pakistan].

**Habit.** Annual.

***Tetrapogon* Desf., Fl. Atlant 2: 388. 1799 (“1800”)**

**Type. ***Tetrapogon villosus* Desf. (as “villosum”).

**1415. **Tetrapogon tenellus** (J. Koenig ex Roxb.) Chiov., Annuario Reale Ist. Bot. Roma 8: 352. 1908 (as “tenellum”) **

*Chloris tenella* J. Koenig ex Roxb., Fl. Ind. (Carey and Wallich ed.) 1: 330. 1820. Type: “A native of the Peninsula of India.” Lectotype: India; *Roxburgh painting 2022* (K). LT cited by Ali Chaudahry in Grass. Saudi Arabia 302. 1989

*Chloris triangulata* Hochst. ex A. Rich., Tent. Fl. Abyss. 2: 409. 1850–1851. Type: “Crescit in valle Mai-Ouid (Fons-Calidus) provinciae Medat, Schimper, pl. Schimp. Abyss., sect. II, 1048”

*Tetrapogon triangularis* Hochst. ex Hook. f., Fl. Brit. India (J.D. Hooker). 7(22): 291. 1896, pro syn.

*Ctenium indicum* Spreng., Syst. Veg. (ed. 16) [Sprengel]. 1: 274. 1824 (“1825”), nom. superfl. & illeg. for *Chloristenella*

**Distribution.** Andhra Pradesh, Bihar, Delhi, Gujarat, Karnataka, Kerala, Madhya Pradesh, Maharashtra, Punjab, Rajasthan, Tamil Nadu [Pakistan].

**Habit.** Annual.

**1416. **Tetrapogon villosus** Desf., Fl. Atlant. 2: 389, t. 255. 1799 (as “villosum”) **

*Chloris villosa* (Desf.) Pers., Syn. Pl. (Persoon) 1: 87. 1805

*Chloris tetrapogon* P. Beauv., Ess. Agrostogr. 158. 1812, nom. superfl. for *Tetrapogon villosus*

**Type.** “Habitat in Arenis prope Cafsam.” Africa: Tunisia, Cafsam, desert areas and dry Mountain slopes; *Desfontaines s.n.* (P).

**Distribution.** Gujarat, Himachal Pradesh, Rajasthan [Pakistan].

**Habit.** Perennial.

***Tragus* Haller, Hist. Stirp. Helv. 2: 203. 1768, nom. cons.**

**Type. ***Tragus racemosus* (L.) All. (*Cenchrus racemosus* L.).

**1417. **Tragus mongolorum** Ohwi, Acta Phytotax. Geobot. 10: 268. 1941 **

*Tragus roxburghii* Panigrahi, Kew Bull. 29: 496. 1974. Type: India: Tamil Nadu, Madras, Vela Cherry; July, 1845; *G. Thomson s.n.* (K).

*Tragus biflorus* auct. non (Roxb.) Schultes, 1824: Senaratna, Grasses of Ceylon 102. 1956

*Tragus racemosus* sensu Hook. f., Fl. Brit. India (J.D. Hooker). 7(21): 97. 1896, non Scop., 1777, nec (L.) All., 1785

**Type.** Mongolia.

**Distribution.** Andhra Pradesh, Assam, Bihar, Delhi, Gujarat, Jammu and Kashmir, Himachal Pradesh, Karnataka, Kerala, Madhya Pradesh, Maharashtra, Meghalaya, Odisha, Punjab, Rajasthan, Tamil Nadu, Uttar Pradesh, Uttarakhand, West Bengal [China, Myanmar, Pakistan, Sri Lanka].

**Habit.** Annual.

***Trigonochloa* P.M. Peterson & N. Snow, Ann. Bot. 109: 1327. 2012**

**Type. ***Trigonochloa uniflora *(Hochst. ex A Rich.) P.M. Peterson & N. Snow (*Leptochloa uniflora* Hochst. ex A. Rich.).

**1418. **Trigonochloa uniflora** (Hochst. ex A Rich.) P.M. Peterson & N. Snow, Ann. Bot. 109: 1328. 2012 **

*Leptochloa uniflora* Hochst. ex A. Rich., Tent. Fl. Abyss. 2: 409. 1850–1851. Type: “Crescit in valle fluvii Tacazzé sub arborum umbra, mense Augusto, Schimper, pl. Abyss. Schimp., sect. III, 1707.” Ethiopia: Tacaze; August 15, 1840; *Schimper 1707 *(P).

*Craspedorhachis uniflora* (Hochst. ex A. Rich.) Chippind., Grass. Pastures South Africa 205. f. 182. 1955

*Cynodon gracilis* Nees, Syn. Pl. Glumac. (Steudel) 1: 213. 1854. Type: India; *Wight s.n.* as *Leptochloa gracilis*

*Agrostis montana* Rottl. ex Hook. f., Fl. Brit. India (J.D. Hooker). 7(22): 298. 1896, pro syn.

**Distribution.** Andhra Pradesh, Kerala, Tamil Nadu [Sri Lanka, Asia, Africa, Arabia].

**Habit.** Annual or perennial.

***Tripogon* Roem. & Schult., Syst. Veg., ed. 15 bis [Roemer & Schultes] 2: 34. 1817**

**Type. ***Tripogon bromoides* Roem. & Schult.

**Notes.** Taxonomic editor for *Tripogon*, Alok R. Chorge, National Museum of Natural History (New Delhi).

**1419. **Tripogon anantaswamianus** Sreek., V.J. Nair & N.C. Nair, Bull. Bot. Surv. India 25(1–4): 185. f. 1. 1985 (“1983”) **

**Type.** India: Kerala, Idukki District, alt. 6400 ft.; November 15; 1980; *P.V. Sreekumar 69432* (CAL).

**Distribution.** Kerala, Tamil Nadu.

**Habit.** Perennial.

**1420. **Tripogon ashihoi** M. Murugesan, S. Arumugam & K.A.A. Kabeer, Indian Journal Forestry 42(1): 67. 2019 **

**Type.** India: Tamil Nadu, Coimbatore district, Ayyamalai, 600 – 1200 m, 18 October 2014, *Murugesan 207* A (holotype: MH; isotypes: 2017 B-D MH).

**Distribution.** Tamil Nadu.

**Habit.** Perennial.

**1421. **Tripogon bimucronatus** Thoiba & Sunil, Gard. Bull. Singapore 67(1): 151, f. 1. 2015 **

**Type.** India: Kerala, Palakkad District, Nelliyampathy hilltop, 1200 m, 16 November 2010, *Sunil*, *C. N. 4477* (holotype SING; isotype: BRIT, CALI, K).

**Distribution.** Kerala.

**Habit.** Perennial.

**1422. **Tripogon borii** Kabeer, V.J. Nair & G.V.S. Murthy, Bull. Bot. Surv. India 50: 115, f. 1. 2009 (“2008”) **

**Type.** India: Tamil Nadu, Kanyakumari district, Nagerkoil, Marunthuvamalai, 360 m, 30 November 2005, *K. Aftaf Ahamed Kabeer 118632* (holotype: CAL; isotype: MH).

**Distribution.** Tamil Nadu.

**Habit.** Perennial.

**1423. **Tripogon bromoides** Roem. & Schult., Syst. Veg., ed. 15 bis [Roemer & Schultes]. 2: 600. 1817 **

*Tripogon bromoides* Roem. & Schult. var. *major* Stapf ex Hook. f., Fl. Brit. India (J.D. Hooker). 7(22): 288. 1896. Type: India: Nilghiri Hills, Nadavattam, alt. 6000 ft.; *Lawson s.n.*

*Tripogon bromoides* Roem. & Schult. var. *longifolius* Hook. f., Fl. Brit. India (J.D. Hooker). 7(22): 288. 1896. Type: India: Nilghiri and Pulney Hills

*Tripogon festucoides* Jaub. & Spach, Ill. Pl. Orient. 4: 49. t. 333. 1851. Type: “India montibus Nelligherry dictis legit *Perrottet*; anno 1840 (Herb. Mus. Par.). “

*Tripogon lanatus* Hochst. ex Steud., Syn. Pl. Glumac. 1: 301. 1854. Type: “Montes Nilagiri, Hrbr Ind. or. *A Hohenack. Edit nr. 922*.”

*Tripogon zeylanicus* sensu Thwaites, Enum. Pl. Zeyl. (Thwaites). 374. 1864, non Nees ex Steud., 1855

*Avena mysorensis* Spreng., Syst. Veg. (ed. 16) [Sprengel] 1: 337. 1824 (“1825”), nom. superfl. & illeg. for *Tripogon bromoides*

*Plagiolytrum calycinum* Nees, Proc. Linn. Soc. London 1: 95. 1841, nom. nud.

*Triathera bromoides* Roth ex Roem. & Schult., Syst. Veg., ed. 15 bis [Roemer & Schultes]. 2: 600. 1817, nom. nud., pro syn.

**Type.** “Heyne in Mysore.”

**Distribution.** Andhra Pradesh, Bihar, Karnataka, Kerala, Madhya Pradesh, Maharashtra, Meghalaya, Odisha, Rajasthan, Tamil Nadu [Nepal, Sri Lanka].

**Habit.** Perennial.

**1424. **Tripogon capillatus** Jaub. & Spach, Ill. Pl. Orient. 4: 47, t. 332. 1851 **

**Type.** “Peninsula Indica, circa urben Poonah, inter Muscos supra arbores et saxa, Legit Jacquemont, September anni 1832 (Herb Mus. Par.) India: Poona; September, 1832; *V. Jacquemont 580* (L).

**Distribution.** Bihar, Goa, Gujarat, Karnataka, Kerala, Madhya Pradesh, Maharashtra, Nepal, Rajasthan, Tamil Nadu [Nepal].

**Habit.** Annual.

**1425. **Tripogon copei** Newmaster, V. Balas., Murug. & Ragup., Syst. Bot. 33(4): 698, f. 2. 2008 **

**Type.** India: Tamil Nadu, Velliangiri Hills, 1840 m, 18 December 2006, *Ragu and Newmast 55277* (holotype: OAC; Isotype: KASCH).

**Distribution.** Tamil Nadu.

**Habit.** Annual.

**1426. **Tripogon filiformis** Nees, Syn. Pl. Glumac. (Steudel) 1: 301. 1854 **

*Tripogon filiformis* var. *tenuispica* Hook. f., Fl. Brit. India (J.D. Hooker). 7(22): 288. 1896. Type: N.W. Himal., Sikkim and Khasia Hills; *Numer. List [Wallich] no. 8892*

*Tripogon semitruncatus* Nees, Syn. Pl. Glumac. (Steudel) 1: 301. 1854. Type: “In monte Laeou Javae.”

*Tripogon unidentatus* Nees, Syn. Pl. Glumac. (Steudel) 1: 301. 1854. Type: “India Orientali” NorthWest India: [India herb. of Dr. Royle p. 92] (P).

*Plagiolytrum unidentatum* Nees, Proc. Linn. Soc. London 1: 95. 1841, nom. nud.

*Plagiolytrum filiforme* Nees, Proc. Linn. Soc. London 1: 95. 1841, nom. nud.

**Type.** India: Northwest; *Herb. Royle s.n.* (P).

**Distribution.** Arunachal Pradesh, Assam, Himachal Pradesh, Jammu and Kashmir, Maharashtra, Meghalaya, Nagaland, Sikkim, Uttar Pradesh, Uttarakhand [Bhutan, China, Myanmar, Nepal, Pakistan].

**Habit.** Perennial.

**1427. **Tripogon idukkianus** Sunil, Pradeep and Thoiba, Phytotaxa 202: 295, f. 1. 2015 **

**Type.** India: Kerala, Idukki district, Ramakkalemedu, 1500 m, 10 November 2002, C. N. Sunil 2267 (holotype: K; isotypes: CALI, MH).

**Distribution.** Kerala.

**Habit.** Perennial.

**1428. **Tripogon jacquemontii** Stapf, Bull. Misc. Inform. Kew 1892: 85. 1892 **

**Syntypes.** Habitat in India from western Ghats to Bengal. Bombay Presidency; *Lisboa s.n.;G.M. Woodrow 79*. Poona; *Jacquemont 320 bis*. Sholapur, Herb Munro. Bengal; *Griffith Kew Distribution no. 6636* (K).

**Distribution.** Andhra Pradesh, Assam, Bihar, Gujarat, Karnataka, Kerala, Madhya Pradesh, Maharashtra, Odisha, Rajasthan, Tamil Nadu, West Bengal.

**Habit.** Perennial.

**1429. **Tripogon jayachandranii** Arum. & Murugan, Indian J. Forest. 40: 159. 2017 **

**Type.** India: Tamil Nadu, Theni District, Meghamalai Wildlife Sanctuary, 1450 m, 04 October 2016, *Arumugam, S. & C. Murugan 134462* (holotype: CAL; Isotypes: MH).

**Distribution.** Tamil Nadu.

**Habit.** Perennial.

**1430. **Tripogon karnatakensis** Thoiba & Pradeep, Phytotaxa 272 (2): 126, f. 1, 2. 2016 **

**Type.** India: Karnataka, Chikmagalur district, Kavikal Gandi Hills, 1450 m, 13 September 2014, *T. Kottekkattu & M. Yoonus T 137510* (holotype: CAL; isotype: CALI, MH, K).

**Distribution.** Karnataka.

**Habit.** Perennial.

**1431. **Tripogon lisboae** Stapf, Bull. Misc. Inform. Kew 1892: 84. 1892 **

**Syntypes.** India: Canara and Mysore to Mt. Abu, Canara and Mysore; *J.S. Law s.n.* Bombay Presidency; *Lisboa s.n. *On the rocks near the Carley Coves, near Poonah; *Jacquemont 581*; Rajputana, Mt. Abu, on rocks; *Duthie 6788.*

**Distribution.** Bihar, Gujarat, Karnataka, Madhya Pradesh, Maharashtra, Rajasthan, Tamil Nadu.

**Habit.** Perennial.

**1432. **Tripogon mahendragiriensis** Chorghe, Sangita Dey, K. Prasad, Prasanna & Y. V. Rao, Nordic Journal of Botany 33: 655. 2015 **

**Type.** India, Odisha state, Gajapati District, Mahendragiri Hill, 17th September 2014, *Chorghe & Prasad 4210* (BSID, CAL).

**Distribution.** Odisha.

**Habit.** Perennial.

**1433. **Tripogon malabarica** Thoiba & Pradeep, J. Bot. Res. Inst. Texas 8(2): 523. 2014 **

**Type.** India, Kerala, Kozhikode district, Malabar Wildlife Sanctuary, Kakkayam, 990 m, *ThoibaKottekkattu 134436* (BRIT, CALI).

**Distribution.** Kerala.

**Habit.** Perennial.

**1434. **Tripogon munnarensis** Thoiba, Manudev & Pradeep, Phytotaxa 272: 130, f. 4, 5. 2016 **

**Type.** India: Kerala, Idukki district, Munnar Udumbanchola, near Mundiyaruma, 1430 m, 16 December 2014, *T. Kottekkattu & A. K. Pradeep 144105* (CAL, CALI, MH, K).

**Distribution.** Kerala.

**Habit. **Perennial.

**1435. **Tripogon nallamalayanus** Rasingam & J. Swamy, Phytotaxa 351(4): 296. 2018 **

**Type.** India: Telangana, Mahbubnagar district, Umamaheshwaram, 700 m, 22 October 2016, *L*. *Rasingam & J. Swamy 7396* (holotype: CAL; isotypes: BSID).

**Distribution.** Telangana.

**Habit.** Perennial.

**1436. **Tripogon narayanae** Sreek., V.J. Nair & N.C. Nair, J. Bombay Nat. Hist. Soc. 80: 196. f. 1–9. 1983 (as “narayanii”) **

**Type.** India: Kerala, Idukki District, Eravikulam National Park, alt. 6400 ft.; August 28, 1980; *P.V. Sreekumar 68412* (CAL).

**Distribution.** Kerala, Tamil Nadu.

**Habit.** Perennial.

**1437. **Tripogon paramjitianus** Murug., Arum. & Kabeer, Indian J. Forest. 40 (3): 285. 2017 **

**Type.** India: Tamil Nadu, Velliangiri hills, Western Ghats, 22 November 2014, *Murugesan 302 A* (holotype & isotype: MH).

**Distribution.** Tamil Nadu.

**Habit.** Perennial.

**1438. **Tripogon polyanthus** Naik & Patunkar, Bull. Bot. Surv. India 15: 158. 1976 (“1973”) **

**Type.** India: Deccan Plateau, Marathwada, Daulatabad; October 18, 1973; *Patunkar 1859A *(Marathwada University, Aurangabad).

**Distribution.** Bihar, Maharashtra.

**Habit.** Perennial.

**1439. **Tripogon pungens** C.E.C. Fisch., Bull. Misc. Inform. Kew 1934: 170–172. f. 4. 1934 **

**Type.** India: South Coimbatore District, Anaimalis Hills, Punachi, 3500 ft.; *C.A. Barber 3717* (K).

**Distribution.** Karnataka, Maharashtra, Tamil Nadu.

**Habit.** Perennial.

**Remarks.** Distribution in India fide Naithani (1990).

**1440. **Tripogon purpurascens** Duthie, Ann. Roy. Bot. Gard. (Calcutta) 9: 74. t. 92. 1901 **

*Tripogon jacquemontii* Stapf var. *submuticus* Hook. f., Fl. Brit. India (J.D. Hooker). 7(22): 287. 1896. Syntypes: India: Western Himalaya, below Kotgurh; *Thomson s.n.* Simla, alt. 7000–8000 ft.; *Duthie s.n.*

*Tripogon abyssinicus* sensu Hook. f., Fl. Brit. India (J.D. Hooker). 7(22): 287. 1896, p.p., non Nees ex Steud., 1854

*Tripogon hookerianus* Bor ex Sultan & R.R. Stewart, Grasses of West Pakistan 2: 254. 1959, nom. invalid.

*Tripogon hookerianus *Bor, Grasses Burma, Ceyl., Ind. & Pakist. 522. 1960, nom. invalid.

**Type.** India: Western Himalaya, Tehri-Garhwal, Tons Valley, 3000–7000 ft.; *Duthie 23532, Duthie 22699* (K).

**Distribution.** Himachal Pradesh, Karnataka, Maharashtra, Rajasthan, Tamil Nadu, Uttar Pradesh, Uttarakhand [Afghanistan, Bhutan, China, Nepal, Pakistan].

**Habit.** Perennial.

**1441. **Tripogon ravianus** Sunil & Pradeep, Sida 19(4): 803. 2001 **

**Type.** India: Tamil Nadu, Nilgiri District, Pykara near Udagamandalam, alt. 6200 ft.; December 3, 2000; *Sunil 2176* (BRIT).

**Distribution.** Tamil Nadu.

**Habit.** Perennial.

**1442. **Tripogon rupestris** S.M. Phillips & S.L. Chen, Kew Bull. 57(4): 917. 2002 **

**Type.** China, Xizang, Dudh Kosi Gorge; 30 Aug 1982; *Miehe 808* (K).

**Distribution.** Uttarakhand [China, Nepal].

**Habit.** Perennial.

**1443. **Tripogon sivarajanii** Sunil, Sida 18(3): 809. f. 1. 1999 **

**Type.** India: Kerala, Idukki District, Valakettimala near Moolamattam, 3200 ft.; September 2, 1997; *C.N. Sunil 2117* (MH).

**Distribution.** Kerala.

**Habit.** Perennial.

**1444. **Tripogon tirumalae** Chorghe, Rasingam, Prasanna & M. Sankara Rao, Phytotaxa 131 (1): 17, 2013 **

**Type.** India, Andhra Pradesh, Tirumala Hills, 4th September 2012, *L. Rasingam, M. S. Rao & Alok Chorghe2914 *(Holotype CAL, Isotype BSID).

**Distribution.** Andhra Pradesh.

**Habit.** Perennial.

**1445. **Tripogon trifidus** Munro ex Hook. f., Fl. Brit. India (J.D. Hooker). 7(22): 286. 1896 **

*Tripogon trifidus* Munro ex Stapf, Bull. Misc. Inform. Kew 1892: 85. 1892, nom. invalid. Lectotype: India: Meghalaya, Khasia; *Griffith HEIC (KD) 6634* (K). LT designated by H.J. Noltie in Edinburgh J. Bot. 56(3): 394. 1999.

**Distribution.** Maharashtra, Meghalaya, Sikkim [Bhutan, China, Myanmar, Nepal].

**Habit.** Perennial.

**1446. **Tripogon umaganeshii** B.R.P. Rao & M. Anil Kumar, Indian J. Forest. 41: 97. 2018 (as “**uma-ganeshii**”) **

**Type.** India: Andhra Pradesh, Horsley hills, 1195 m elevation, 19 September 2016, *B.R. Prasad Rao & M.A. Kumar 51980* (holotype: SKU; isotype: BSID).

**Distribution.** Andhra Pradesh.

**Habit.** Perennial.

**1447. **Tripogon vellarianus** Pradeep, Sida 18(3): 811. f. 2. 1999 **

**Type.** India: Kerala, Kozhikode District, Vellarimala, 11°25.877'N, 76°06.765'E, 4200 ft.; October 17, 1997; *A.K. Pradeep 56110 *(MH).

**Distribution.** Kerala.

**Habit.** Perennial.

**1448. **Tripogon velliangiriensis** Murug. & V. Balas., Indian J. Forest. 31(1): 109. 2008 **

**Type.** India: Tamil Nadu, Coimbatore district, Velliangiri hills, 1850 m, 12 September 2003, Murugesan 1181 (holotype: KASCH; isotype: MH).

**Distribution.** Tamil Nadu.

**Habit.** Annual.

**1449. **Tripogon wightii** Hook. f., Fl. Brit. India (J.D. Hooker). 7(22): 286. 1896 var. **wightii

**Type.** India: Mysore, Bellary; 1834; *Wight 1793* (K).

**Distribution.** Andhra Pradesh, Karnataka, Kerala, Tamil Nadu.

**Habit.** Perennial.

**1450. **Tripogon wightii** Hook. f. var. **kanyakumariensis** Kabeer & V.J. Nair, Fl. Tamil Nadu: Grasses 183, 2009 **

**Type.** India: Tamil Nadu, Kanyakumari District, Mothiramalai, Thomarai, near Pechiparai, alt. 2000 ft.; December 16, 2005; *K.A.A. Kabeer 118769* (MH).

**Distribution.** Tamil Nadu.

**Habit.** Perennial.

### Subfamily Micrairoideae Pilg., 1956

Micrairoideae is a subfamily with no obvious synapomorphies, although most species have spikelets with only two flowers (Kellogg 2015b). The ligule is a fringe of hairs. The genera are morphologically heterogeneous, and include both C3 and C4 species, some of which are adapted to forest shade and others to extreme drought (Hardion et al., 2017; Teisher et al. 2019).

Taxonomic editors: Jordan K. Teisher, Drexel University, Philadelphia, P. (Parigi) Venkateswara Prasanna, Botanical Survey of India, and Alok R. Chorghe, Botanical Survey of India.

***Coelachne* R. Br., Prodr. Fl. Nov. Holland. 187. 1810**

**Type. ***Coelachne pulchella* R. Br.

**1451. **Coelachne minuta** Bor, J. Bombay Nat. Hist. Soc. 58: 317. 1961 **

*Coelachne ghatica* Naik, Reinwardtia 9(4): 393, f. 1. 1980. Type: India: Western Ghats, Amboli Hill station; September 13, 1971; *V.N. Naik 1300a* (Herb. MU Aurangabad).

**Type.** India: Bombay, Mahableshwar; September 14, 1958; *H. Santapau 22731* (K).

**Distribution.** Bihar, Maharashtra [China].

**Habit.** Annual.

**1452. **Coelachne perpusilla** (Arn. ex Steud.) Thwaites, Enum. Pl. Zeyl. (Thwaites). 373. 1864 var. **perpusilla

*Panicum perpusillum* Arn. ex Steud., Syn. Pl. Glumac. 1: 96. 1854. Type: Sri Lanka

*Coelachne perpusilla* (Arn. ex Steud.) Thwaites var. *muscosa* Hook. f. in Trimen, Handb. Fl. Ceylon 5: 270. 1900

*Coelachne pulchella* R. Br. var. *perpusilla* (Arn. ex Steud.) Hook. f., Fl. Brit. India (J.D. Hooker). 7(22): 271. 1896.

**Distribution.** Kerala, Manipur, Maharashtra, Tamil Nadu [Myanmar, Sri Lanka].

**Habit.** Perennial.

**1453. **Coelachne perpusilla** (Arn. ex Steud.) Thwaites var. **nilagirica** V. Prakash & S.K. Jain, Bull. Bot. Surv. India 24(1–4): 187. f. 12. 1983 (“1982”) **

**Type.** India: Tamil Nadu, Nilgiri District, Parthimund, alt. 7500 ft.; July 11, 1970; *I.L. Ellis 34627* (CAL).

**Distribution.** Kerala, Tamil Nadu.

**Habit.** Perennial.

**1454. **Coelachne simpliciuscula** (Wight & Arn. ex Steud.) Munro ex Benth., J. Linn. Soc., Bot. 19: 93. 1881 **

*Panicum simpliciusculum* Wight & Arn. ex Steud., Syn. Pl. Glumac. 1: 96. 1854. Type: Sri Lanka; *Nees s.n.*

*Coelachne pulchella* R. Br. var. *simpliciuscula* (Wight & Arn. ex Steud.) Hook. f., Fl. Brit. India (J.D. Hooker). 7(22): 271. 1896

*Isachne simpliciuscula* (Wight & Arn. ex Steud.) Thwaites, Enum. Pl. Zeyl. (Thwaites). 373. 1864

*Isachne hispidula* Nees ex Steud., Syn. Pl. Glumac. 1: 96. 1854, nom. nud., pro. syn.

*Coelachne pulchella* sensu Hook. f., Fl. Brit. India (J.D. Hooker). 7(22): 270. 1896, non R. Br., 1810

**Distribution.** Andhra Pradesh, Arunachal Pradesh, Assam, Bihar, Chhattisgarh, Karnataka, Kerala, Madhya Pradesh, Maharashtra, Meghalaya, Nagaland, Odisha, Sikkim, Tamil Nadu, West Bengal [Bhutan, China, Myanmar, Nepal, Sri Lanka].

**Habit.** Annual.

**Eriachne R. Br., Prodr. Fl. Nov. Holland. 183. 1810**

**Lectotype. ***Eriachne squarrosa* R. Br. LT designated by Eck-Borsboom in Blumea 26: 127, 130. 1980.

**1455. **Eriachne pallescens** R. Br., Prodr. Fl. Nov. Holland. 1: 184. 1810 **

*Aira chinensis* Retz., Observ. Bot. (Retzius) 3: 10. 1783. Type: “E China missit Cl.D. Bladh.” China; *Bladh s.n. *(LD).

*Tricholaena chinensis* (Retz.) Domin, Biblioth. Bot. 20 (Heft. 85): 327. 1915

*Eriachne chinensis* Hance, Ann. Sci. Nat., Bot. Ser. 4, 15: 228. 1861. Type: “In summis collibus ins. Hongkong necnon ad Whampoa. Legi Aug-Oct. 1861.”

**Type.** Australia: Queensland, Cook District, Endeavor River; *Banks &Solander s.n. *(BM).

**Distribution.** Andaman and Nicobar Islands, Assam [Bangladesh, China, Myanmar].

**Habit.** Perennial.

**Remarks.***Aira chinensis* and *Eriachne chinensis *are heterotypic synonyms. Thus *E. chinensis* is not based on *A. chinensis*, and the latter cannot be transferred to *Eriachne*.

**1456. **Eriachne triseta** Nees, Syn. Pl. Glumac. (Steudel) 1: 237. 1854 **

*Massia triseta* (Nees) Balansa, J. Bot. (Morot) 4(9): 165. 1890

*Megalachne zeylanica* Thwaites, Enum. Pl. Zeyl. (Thwaites). 372, 444. 1864, nom. invalid.

**Type.** Sri Lanka; *Walker s.n.* (P).

**Distribution.** North East India [China, Sri Lanka].

**Habit.** Perennial.

***Hubbardia* Bor, Kew Bull. 1950: 385. 1951**

**Type. ***Hubbardia heptaneuron* Bor.

**1457. **Hubbardia diandra** Chandore, Gosavi & S. R. Yadav, Kew Bull. 67 (3): 533–537. 2012 **

**Type.** India, Maharashtra, Kohlapur, Tillari Ghat, 15°47.400'N, 74°10.769'E, 15 Sept. 2010, *Chandore 1301* (Holotype CAL; isotype BSI, K, SUK).

**Distribution.** Karnataka, Maharashtra.

**Habit. **Annual.

**1458. **Hubbardia heptaneuron** Bor, Kew Bull. 1950: 385. 1951 (“1950”) **

**Type.** India: Bombay, N. Kanara, Jog, Gersoppa Falls, 500 ft.; October, 1919; *L.F. Sedgwick 7089 *(K).

**Distribution.** Karnataka, Maharashtra.

**Habit.** Annual.

**Remarks.** Endangered.

***Isachne* R. Br., Prodr. Fl. Nov. Holland. 196. 1810**

**Type. ***Isachne australis* R. Br.

**1459. **Isachne albens** Trin., Sp. Gram. [Trinius] 1(8): t. 85. 1827 var. **albens

**Type.** “Figura ad specimen nepalense.”

**Distribution.** Andra Pradesh, Arunachal Pradesh, Assam, Himachal Pradesh, Maharashtra, Manipur, Meghalaya, Nagaland, Odisha, Sikkim, Uttar Pradesh, Uttarakhand, West Bengal [Bangladesh, Bhutan, China, Myanmar, Nepal].

**Habit.** Perennial.

**1460. **Isachne albens** Trin. var. **hirsuta** Hook. f., Fl. Brit. India (J.D. Hooker). 7(21): 23. 1896 **

*Isachne hirsuta* (Hook. f.) Keng, Acta Phytotax. Sin. 10: 11. 1965.

**Type.** Numer. List [Wallich] no. 8657. Silhet; *De Silva s.n.* Cachar; *Keenan s.n.*

**Distribution.** Arunachal Pradesh, Assam, Himachal Pradesh, Maharashtra, Manipur, Meghalaya, Nagaland, Sikkim, Uttar Pradesh, West Bengal [Bangladesh, Bhutan, China, Nepal].

**Habit.** Perennial.

**1461. **Isachne angladei** C.E.C. Fisch., Bull. Misc. Inform. Kew 1932: 323. 1932 **

**Type.** India: “Pulney Hills”, Shembaganur, alt. 6000 ft.; *L. Anglade 914.*

**Distribution.** Karnataka, Maharashtra, Tamil Nadu.

**Habit.** Perennial.

**Remarks.** Records for Karnataka and Tamil Nadu fide Naithani (1990).

**1462. **Isachne bhatii** P. Biju, Josekutty & Augustine, Int. J. Pl. Anim. Environm. Sci. 6(3): 135. 2016 **

**Type.** India: Kerala, Kannur district, Kalliad lateritic plateau, 82 m, 23 August 2014, Biju & Jonny 2491 (holotype CAL, iso MH).

**Distribution.** Kerala.

**Habit.** Annual.

**1463. **Isachne bicolor** Naik & Patunkar, Bull. Bot. Surv. India 15(1–2): 157. 1976(“1973”) **

**Type. **India: Maharashtra, Aurangabad District, Mhaismal Plateau; October 8, 1973; *B.W. Patunkar 1849A* (Marathwada University Aurangabad).

**Distribution.** Bihar, Maharashtra.

**Habit.** Perennial.

**1464. **Isachne borii** Hemadri, Indian Forester 97(4): 223. 1971 **

**Type.** India: Maharashtra, Puna District, 27 km West of Junnar, Dhak Khilla; September 22, 1968; *Hemadri 117968A* (CAL).

**Distribution.** Maharashtra.

**Habit.** Annual.

**1465. **Isachne bourneorum** C.E.C. Fisch., Bull. Misc. Inform. Kew 1932: 324. 1932 **

**Type. **India: Tamil Nadu, Palni Hills, Kodaikanal, alt. 6500 ft.; June 14, 1898; *Sir A.G. & Lady Bourne 2491.*

**Distribution.** Karnataka, Kerala, Tamil Nadu.

**Habit.** Perennial.

**Remarks.** Record for Karnataka fide Naithani (1990).

**1466. **Isachne clarkei** Hook. f., Fl. Brit. India (J.D. Hooker). 7(21): 24. 1896 **

**Lectotype.** North East India: Kohima, alt. 5000 ft.; October 20, 1885S; *C.B. Clarke s.n.* (K). LT designated by M. Phillips & S.L. Chen, Novon 15(3): 465. 2005.

**Distribution.** Meghalaya, Nagaland, Sikkim [China, Myanmar].

**Habit.** Annual.

**1467. **Isachne confusa** Ohwi, Bull. Tokyo Sci. Mus. 18: 14. 1947 **

*Isachne rigida* Hook. f., Fl. Brit. India (J.D. Hooker). 7(21): 24. 1896, non Nees ex Steud., 1854

**Type.** Indonesia: Sumatra, Bangka Island; *Bunnemeijer 1577 in Herb. Bogor.*

**Distribution.** Andaman and Nicobar Islands [China].

**Habit.** Perennial.

**1468. **Isachne deccanensis** Bor, Kew Bull. 1949: 95–96. 1949 **

**Type.** India: Tamil Nadu, Nilgiris, Ootacamund Downs; August, 1884; *Gamble 15290* (K).

**Distribution.** Tamil Nadu.

**Habit.** Perennial.

**1469. **Isachne dimyloides** Bor, Kew Bull. 1949: 96. 1949 **

**Type.** India: Sikkim Terai, Dulkajhar, alt. 700 ft.; October 16, 1884; *C.B. Clarke 36764* (K).

**Distribution.** Sikkim.

**Habit.** Annual.

**1470. **Isachne edamalayarensis** Sunil, Naveen Kum. & Sivad., Nordic J. Bot. 35(3): 354–358. 2016 **

**Type.** India: Kerala, Ernakulam district, Edamalayar Forest Range, 913 m a.s.l., 26 December 2014, *S. and N. Kumar 6926* (holotype: MH, Isotypes: CAL).

**Distribution.** Kerala.

**Habit.** Perennial.

**1471. **Isachne elegans** Dalzell in Dalzell & A. Gibson, Bombay Fl. 291. 1861 **

**Type.** India: Deccan, on the margins of rivulets; *Dalzell s.n.*

**Distribution.** Karnataka, Maharashtra, Rajasthan, Tamil Nadu.

**Habit.** Annual.

**1472. **Isachne fischeri** Bor, Kew Bull. 1949: 69–70. 1949 **

**Type.** India: Kerala, Madras, Summit of Anaimudi, Travancore High Range, 8840 ft.; September, 1933; *E. Barnes s.n.* (K).

**Distribution.** Kerala.

**Habit.** Annual.

**Remarks. **Taxonomic editor Teisher questions the interpretation of “Madras” in the description of the type. He notes that “the specimen is cited as part of a project on the Flora of Madras, and Madras is another name for Chennai, the capital of Tamil Nadu. However, I think it more likely refers to the historic Madras Presidency, which covered most of Southern India, including Kerala. Anamudi is a mountain in Kerala. Thus, I would recommend emending the type description, which would include Kerala and exclude the Tamil Nadu distribution.”

**1473. **Isachne globosa** (Thunb.) Kuntze, Revis. Gen. Pl. 2: 778. 1891 **

*Milium globosum* Thunb., in J. A. Murray, Syst. veg. ed. 14: 109. 1784. Type: Japan: without specific locality; *Thunberg 2041* (UPS).

*Isachne australis* R. Br., Prodr. Fl. Nov. Holland. 1: 196. 1810. Type: Australia: New South Wales, inclusis ripis aestuarii Hunter’s River vel Coal River (Page VI in Praemonenda); *R. Brown 6129 *(BM).

*Isachne australis* R. Br. var. *effusa* Trimen ex Hook. f., Fl. Brit. India (J.D. Hooker). 7(21): 25. 1896. Type: Sri Lanka: Peradeniya; *Trimen s.n.*

*Isachne miliacea* Roth, Syst. Veg., ed. 15 bis [Roemer & Schultes]. 2: 476. 1817. Type: India: *Heyne 1814 *(BM).

*Isachnedispar* Trin., Sp. Gram. [Trinius] 1(8): t. 86. 1827 var. dispar. Type: “figura ad specimen nepalense” Nepal: Herb. Trin. 678.1 (LE, microfiche IDC BT-1671-B5).

*Panicum dispar* (Trin.) Steud., Syn. Pl. Glumac. 1: 96. 1854

*Isachne dispar* Trin. var. *villosa* C.E.C. Fisch., Fl. Madras 3(10): 1797. 1934. Type: India: High Wavy Mountains; *Blatter and Hallberg s.n.*

*Isachne lutaria* Santos, J. Wash. Acad. Sci. 33(5): 140, f. 3. 1943. Type: India: Travancore, Trivandrum, at the edge of a paddy field; January 8, 1934; *E.W. Erlanson 5190* (MICH).

*Panicum nodibarbatum* Hochst. ex Steud., Syn. Pl. Glumac. 1: 95. 1854. Type: India: Nilgiri Mountains;* R.F. Hohenacker 1275*

*Isachne nodibarbata* (Hochst. ex Steud.) Henrard, Blumea 3(3): 464. 1940

*Panicum violaceum* Klein, Linnaea 9: 307. 1834. Type: “Habitat in agris oryzaceis Indiae orientalis (Klein).”

**Distribution.** Andhra Pradesh, Assam, Bihar, Chhattisgarh, Dadra and Nagar Haveli, Daman and Diu, Goa, Gujarat, Jharkhand, Karnataka, Kerala, Madhya Pradesh, Maharashtra, Manipur, Odisha, Rajasthan, Sikkim, Tamil Nadu, Tripura, Uttar Pradesh, West Bengal [Bangladesh, Bhutan, China, Myanmar, Nepal, Sri Lanka].

**Habit.** Annual or perennial.

**Remarks.** Taxonomic editor Teisher notes that *Isachne dispar* vars. *dispar* and *villosa* are treated as synonymys of *I*. *globosa* by Clayton et al (2006 onwards), whereas Iskandar and Veldkamp (2004) claim *Isachne dispar *is a synonym of *Isachne pulchella *Roth based on the type description. However, Prakash and Jain (1984) note that “*Isachne dispar* Trin.” in Indian Floras was misidentified throughout, and “the specimens which were assigned to *I. dispar* in Indian Herbaria and Floras actually belong either to *I. globosa* or to *I. miliacea*”.

**1474. **Isachne gracilis** C.E. Hubb., Bull. Misc. Inform. Kew 1927: 77. 1927 **

**Type.** India: Mysore, Baba, Budan Hills, Santavera, alt. 4000 ft., in deep shade on rocks; *Meebold 10781.*

**Distribution.** Karnataka, Kerala, Madhya Pradesh, Maharashtra.

**Habit.** Annual.

**Remarks.** All records from India fide Prakash and Jain (1984).

**1475. **Isachne henryi** S. R. Sriniv. & Sreek., J. Bombay Nat. Hist. Soc. 84: 647. f. 1 (A–H). 1988 **

**Type.** India: Kerala, Wynad District, Poothumoola, Hiladale R.F.; 2400 ft.; November 13, 1981; *S.R. Srinivasan 72358* (CAL).

**Distribution.** Kerala.

**Habit.** Annual.

**1476. **Isachne himalaica** Hook. f., Fl. Brit. India (J.D. Hooker). 7(21): 23. 1896 **

**Syntypes.** India: Kumaon;* R. Strachey & J.E. Winterbottom s.n.* Nepal; *Wallich(Numer. List [Wallich] no. 8656 D, E*. Pakistan: Peshawur; *Aitchison s.n.* India: Rajaori; *Jacquemont s.n*.

**Distribution.** Assam, Himachal Pradesh, Jammu and Kashmir, Meghalaya, Nagaland, Punjab, Uttar Pradesh, Uttarakhand [Afghanistan, Nepal, Pakistan].

**Habit.** Perennial.

**1477. **Isachne jayachandranii** Gopalan & V. Chandras., Kew Bull. 55(4): 1005. 2000 **

**Type.** India: Kerala, Thiruvananthapuram District, southern Western Ghats, western slopes of Agasthiyamalai, c. 4500 ft.; December 6, 1973; *J. Joseph 44628* (CAL).

**Distribution.** Kerala.

**Habit.** Annual.

**1478. **Isachne kannurensis** Sunil, Ratheesh, Sujana & Sreek., Int. J. Advanced Res. 2(12): 149. 2014 (as ‘**kannurense**’) **

**Type.** India: Kerala, Kannur District, Kanai Kanam, 200 m, 24 September 2012, *Sunil, RatheeshNarayanan & Nandakumar 4258* (holotype CAL, Isotypes MH, MSSH).

**Distribution.** Kerala.

**Habit.** Annual.

**1479. **Isachne kinabaluensis** Merr., J. Straits Branch Roy. Asiat. Soc. 76: 77. 1917 **

*Isachne javana* sensu Hook. f., Fl. Brit. India (J.D. Hooker). 7(21): 24. 1896, non Nees ex Steud., 1854

**Type.** British North Borneo: Mount Kinabalu, Paka Cave to Lobang, alt. 4600- 9200 ft.; November 15, 1916; *Clemens 10704.*

**Distribution.** Meghalaya [Myanmar].

**Habit.** Perennial.

**1480. **Isachne kunthiana** (Wight & Arn. ex Steud.) Nees ex Miq., Fl. Ned. Ind. 3(3): 460. 1857 var. **kunthiana

*Panicum kunthianum* Wight & Arn. ex Steud., Syn. Pl. Glumac. 1: 96. 1854. Type: Indonesia: Java; *Zollinger Herb. no.271*

*Panicum cuspidigluma* Steud., Syn. Pl. Glumac. 1: 96. 1854. Type: Sri Lanka; Arnott Herb. no. 2045 as *Isachne neesiana*

*Panicum neesianum* Wight & Arn. ex Steud., Syn. Pl. Glumac. 1: 74. 1853. Type: Sri Lanka; *Lindley s.n.*

*Panicum metzii* Hochst. ex Steud., Syn. Pl. Glumac. 1: 95. 1854. Type: India: Nilgiri Mountains;* R.F. Hohenacker 1276*

*Isachne metzii* (Hochst. ex Steud.) Henrard, Blumea 3(3): 467. 1940

*Isachne elatior* Hook. f., Fl. Brit. India (J.D. Hooker). 7(21): 22. 1896. Syntypes: Sri Lanka; *Gardner s.n.* (K, PDA). Sri Lanka: Nuwara Eliya; *Thwaites Ceylon Plant 314* (K).

*Isachne kunthiana* (Wight & Arn. ex Steud.) Miq. var. *elatior* (Hook. f.) Alston in Trimen, Handb. Fl. Ceylon 6: 316. 1931

*Isachne kunthiana* (Wight & Arn. ex Steud.) Miq. var. *latifolia* Hook. f., Fl. Brit. India (J.D. Hooker). 7(21): 22. 1896. Syntypes: India: Tamil Nadu, Nilghiri Hills, alt. 6000 ft.; *Lawson s.n.* India: Anamallay Hills; *Beddome s.n.*

**Distribution.** Kerala, Karnataka, Tamil Nadu [Sri Lanka].

**Habit.** Perennial.

**Remarks.** Taxonomic editor Teisher notes that “Prakash & Jain (1984) lump *I. kunthiana* var. *latifolia* with *I. kunthiana* on the grounds that the species is highly variable in size and shape, and the varieties were based on quantitative characters that blend together in the study of a larger number of specimens. In the original description, Hooker (1896) describes *I. kunthiana* var. *latifolia* as “almost intermediate between *I. kunthiana* and *I. elatior*”. Since the latter is treated as a synonym under *I. kunthiana*, I would be inclined to agree that var. *latifolia* should also be lumped (this is also the position taken by the World Grass Species Synonymy database, Clayton et al. 2002).”

**1481. **Isachne lisboae** Hook. f., Fl. Brit. India (J.D. Hooker). 7(21): 22. 1896 **

**Type.** India: the Concan, Mahableshwar; *Lisboa s.n.*

**Distribution.** Karnataka, Maharashtra.

**Habit.** Annual.

**1482. **Isachne manilaliana** Sunil, K.M.P. Kumar & V.P. Thomas in Webbia 72(2): 161. 2017 **

**Type.** India: Kerala: Palakkad district, Muthikulam, Elivalmala, 2000 m, 11 November 2016, *Sunil & K. M. Prabhukumar 4574* (holotype CMPR, Isotypes MH, SNMH, CATH).

**Distribution.** Kerala.

**Habit.** Annual.

**1483. **Isachne meeboldii** C.E.C. Fisch., Bull. Misc. Inform. Kew 1932: 323. 1932 **

**Type.** India: Mysore, Shimoga, alt. 2000–3000 ft.; October; *A. Meebold 10747.*

**Distribution.** Karnataka.

**Habit.** Perennial.

**1484. **Isachne minutula** (Gaudich.) Kunth, Révis. Gram. 2: t. 117. 1831 **

*Panicum minutulum *Gaudich. in Freyc., Voy. Uranie: 410. 1829. Type: Mariana Islands. Lectotype: *Gaudichaud-Beaupré s.n.* (P [P01934348]). LT designated by Veldkamp in Turner *et al.*, Gard. Bull. Sing. 71(1): 27. 2019.

*Isachne geniculata* Griff., Not. Pl. Asiat. 3: 41. 1851. Type: “Bengal Prope Osunpoor; September 27, 1835”

*Isachne stigmatosa* Griff., Not. Pl. Asiat. 3: 42. t. 147. t. 2. 1851. Type: “In humidis super inundatis, ad ripas flum. Boorea Barak Paulo infra Luckipoor; October 3, 1835.” India; October 3, 1835; *Griffith s.n. *(K-245425).

**Distribution.** Andaman and Nicobar Islands, Andhra Pradesh, Assam, Bihar, Goa, Karnataka, Kerala, Madhya Pradesh, Maharashtra, Meghalaya, Odisha, Rajasthan, Sikkim, Tamil Nadu, West Bengal [China, Nepal, Sri Lanka].

**Habit.** Perennial.

**Remarks.**Iskandar and Veldkamp (2004) mention that this taxon has been erroneously referred to in the literature as *Isachne miliacea *Roth or *Isachne pulchella *Roth, but claim the type specimen of *I. miliacea* belongs in *Isachne globosa*. This unfortunate confusion renders interpretations of historic references in the literature to this taxon difficult.

**1485. **Isachne mysorensis** Sundararagh., Indian Forester 97(6): 304. t. 1. f. 1–9. 1971 **

**Type.** India: Karnataka, Shimoga District, Kundadagudda near Agumbe; August 19, 1963; *Sundararaghavan 90008A* (BSI).

**Distribution.** Karnataka.

**Habit.** Annual.

**1486. **Isachne oreades** (Domin) Bor, Grasses Burma, Ceylon, India & Pakistan 582. 1960 **

*Panicum oreades* Domin, Biblioth. Bot. 20(Heft. 85): 297. 1915, nom. nov. for *P. aequiglume* Hook. f.

*Panicum aequiglume* Hook. f., Fl. Brit. India (J.D. Hooker). 7(21): 44. 1896, non Hack. & Arechav. 1894. Type: India: Tamil Nadu, Nilghiri Hills, Goodadoor Ghats, alt. 5000 ft.; *Lawson s.n.*

**Distribution.** Tamil Nadu.

**Habit.** Perennial.

**1487. **Isachne pulchella** Roth, Syst. Veg., ed. 15 bis [Roemer & Schultes] 2: 476. 1817 **

**Type.** India;* B. Heyne s.n.* (B).

**Distribution.** Andaman and Nicobar Islands, Andhra Pradesh, Assam, Bihar, Kerala, Madhya Pradesh, Maharashtra, Manipur, Nagaland, Odisha, Tamil Nadu, Uttar Pradesh, West Bengal [Bangladesh, China, Myanmar, Nepal].

**Habit.** Annual.

**Remarks.** Taxonomic editor Teisher notes that Iskandar and Veldkamp (2004) include *I. dispar* in the synonymy of *I. pulchella*. See comments under *I. dispar*.

**1488. **Isachne scabrosa** Hook. f., Fl. Brit. India (J.D. Hooker). 7(21): 23. 1896 **

**Type.** India: Khasia Hills, alt. 4500–5000 ft.; *C.B. Clarke s.n.*

**Distribution.** Assam, Meghalaya [China, Nepal].

**Habit.** Perennial.

**1489. **Isachne setosa** C.E.C. Fisch., Bull. Misc. Inform. Kew 1932: 247. 1932 **

**Type.** India: Cochin, Kavalai, alt. 3000–4000 ft.; *A. Meebold 12125* (BRLU) “Breslau Herbarium”).

**Distribution.** Karnataka, Kerala, Tamil Nadu.

**Habit.** Annual.

**Remarks.** Records from India fide Bor (1960), Prakash and Jain (1984), and Iskandar and Veldkamp (2004).

**1490. **Isachne sikkimensis** Bor, Kew Bull. 1949: 115. 1949 **

**Type.** India: Sikkim, Karponang, 9000 ft.; August 5, 1945; *Bor’s collector s.n.* (K).

**Distribution.** Sikkim, West Bengal, Uttar Pradesh [Bhutan, China, Nepal].

**Habit.** Annual.

**Remarks.** Records from Sikkim and West Bengal fide Prakash and Jain (1984).

**1491. **Isachne swaminathanii** Ved Prakash & S.K. Jain, Proc. Indian Acad. Sci. Pl. Sci. 92(1): 19. f. 10. 1983 **

**Type.** India: Maharashtra, Satara District, Mahabaleshwar, 4500 ft.; October 9, 1979; *Ved Prakash 337A* (CAL).

**Distribution.** Maharashtra.

**Habit.** Perennial.

**1492. **Isachne sylvestris** Ridley, J. Straits Branch Roy. Asiat. Soc. 44: 206. 1905 **

**Type.** Indonesia: Malay Peninsula: Telok Sera; March 17, 1896; *Ridley 7265 *(K).

**Distribution.** North East India; information on specific states lacking. [Bangladesh, China].

**Habit.** Perennial.

**1493. **Isachne veldkampii** K. G. Bhat & Nagendran, Curr. Sci. 52(6): 258. f. 1. 1983 **

**Type.** India: Karnataka State, South Kanara District, Manipal, alt. 500 ft.; October 29, 1977; *K.G. Bhat 468A* (CAL).

**Distribution.** Karnataka.

**Habit.** Annual.

**1494. **Isachne walkeri** (Steud.) Wight & Arn., Enum. Pl. Zeyl. (Thwaites). 361. 1864 **

*Panicum walkeri* Steud., Syn. Pl. Glumac. 1: 97. 1854. Type: India Orientalis, Sri Lanka; *R.F. Hohenacker 1280*

**Distribution.** Kerala, Tamil Nadu [Sri Lanka].

**Habit.** Perennial.

***Limnopoa* C.E. Hubb., Hooker’s Icon. Pl. 35: sub t. 3432. 1943**

**Type. ***Limnopoa meeboldii* (C.E.C. Fisch.) C.E. Hubb. (*Coelachne meeboldii* C.E.C. Fisch.).

**1495. **Limnopoa meeboldii** (C.E.C. Fisch.) C.E. Hubb., Hooker’s Icon. Pl. 35: t. 3432. 1943 **

*Coelachne meeboldii* C.E.C. Fisch., Bull. Misc. Inform. Kew 1934: 169. 1934. Type: India: Cochin state, Chalakudi; November; *A. Meebold 12520* (K).

**Distribution.** Karnataka, Kerala.

**Habit.** Annual.

***Sphaerocaryum* Nees ex Hook. f., Fl. Brit. India (J.D. Hooker). 7(22): 246. 1896**

**Type. ***Sphaerocaryum elegans* (Arn. ex Steud.) Nees ex Hook. f. (*Graya elegans* Arn. ex Steud*.*, *Isachne pulchella* Roth; *Sphaerocaryum pulchellum* (Roth) Merr.).

**1496. **Sphaerocaryum malaccense** (Trin.) Pilger, Repert. Spec. Nov. Regni Veg. 45: 2. 1938, in obs. **

*Panicum malaccense* Trin., Gram. Panic. [Trinius] 204. 1826. Type: “V. sp. malacc. (Stephan, qui a beato Willdenovio sub nominee Agrostis malaccensis acceperat.” India; *Willdenow s.n.* as Agrostios malaccensis (LE-TRIN-0806.01 (ex hrbr. Willd. 1737 & fig.)) fragment & drawing.

*Sphaerocaryum elegans* Nees ex Steud., Nomencl. Bot., ed. 2 (Steudel) 2: 620. 1841, nom. nud.

**Distribution.** Assam, Meghalaya, Nagaland [Bangladesh, China, Myanmar, Sri Lanka].

**Habit.** Annual.

***Zenkeria* Trin., Linnaea 11: 150. 1837**

**Type. ***Zenkeria elegans* Trin.

**1497. **Zenkeria elegans** Trin., Linnaea 11: 150. t. 3. 1837 **

*Amphidonax tenella* Wight. & Arn. ex Steud., Syn. Pl. Glumac. 1: 197. 1854. Type: “Peninsula Ind. Or.”

*Amphidonax heynei* Nees in Wight., Cat. 1747. 1834, nom. nud.

**Type.** “Habitat in Indiae orientalis montibus Nilagiri dictis.”

**Distribution.** Kerala, Tamil Nadu [Myanmar, Sri Lanka].

**Habit.** Perennial.

**1498. **Zenkeria jainii** N.C. Nair, Sreek. & V.J. Nair, J. Bombay Nat. Hist. Soc. 78: 354. 1981 **

**Type.** India: Kerala, Idukki District, Eravikulam Sanctuary, alt. 6400 ft.; August 20, 1980; *P.V. Sreekumar 68419* (CAL).

**Distribution.** Kerala, Tamil Nadu.

**Habit.** Perennial.

**1499. **Zenkeria sebastinei** A.N. Henry & Chandrab., Bull. Bot. Surv. India 15: 142. 1976 (“1973”) **

**Type. **Tamil Nadu, Tirunelveli District, Agastyamalai; August 26, 1963; *Henry 17325* (CAL).

**Distribution.** Kerala, Tamil Nadu.

**Habit.** Perennial.

**1500. **Zenkeria stapfii** Henrard, Repert. Spec. Nov. Regni Veg. 17: 396. 1921 **

**Type.** India Orientalis: Madras Province, Nilgiri Mountains; *Perrotet 1336.*

**Distribution.** Karnataka, Tamil Nadu [Sri Lanka].

**Habit.** Perennial.

### Subfamily Arundinoideae Kunth ex Beilschm., 1833

Plants perennial, from several meters tall and reed-like to only a few cm in height. Spikelets laterally compressed.

Arundinoideae has historically been a subfamily to accommodate misfit genera (Clayton and Renvoize 1986; Watson and Dallwitz 1992 onwards), but morphological and molecular phylogenetic analyses showed that the traditionally circumscribed subfamily was polyphyletic (Barker et al. 1995; Clark et al. 1995; Kellogg and Campbell 1987). Over the last several decades the subfamily has been reduced to a monophyletic core of genera. Surprisingly, despite the sharp reduction in genera in the subfamily, it still has no obvious morphological synapomorphy or even any general facies to help with recognition. The current circumscription of the subfamily follows Teisher et al. (2017) and Hardion et al. (2017).

Taxonomic editor: Jordan K. Teisher, Drexel University, Philadelphia

***Arundo* L., Sp. Pl. 1: 81. 1753**

**Lectotype. ***Arundo donax* L. LT designated by Britton, Fl. Bermuda 29. 1918; affirmed by Hitchcock, Bull. U.S.D.A. 772: 60. 1920.

**1501. **Arundo donax** L., Sp. Pl. 1: 81. 1753 **

*Cynodon donax* (L.) Raspail, Ann. Sci. Nat. (Paris) 5: 302. 1825

*Scolochloa donax* (L.) Gaudin, Fl. Helv. 1: 202. 1828

*Arundo bifaria* Retz., Observ. Bot. (Retzius) 4: 21. 1786–1787. Type: “Habitat ad margines stagnorum et fossarum in India Orientali. *Cel. Koenig* dedit.”

*Donax arundinaceus* P. Beauv., Ess. Agrostogr. 152, 161. 1812

*Scolochloa arundinacea* (P. Beauv.) Mert. & Koch ex Roehl., Deutschl. Fl., ed. 3, 1(2) : 530. 1823, nom. superfl. illeg.

*Arundo glauca* sensu Bubani, Fl. Pyren. (Bubani) 4: 303. 1901, non Bieb., 1808

*Arundo latifolia* Salisb., Prodr. Stirp. Chap. Allerton 24. 1796, nom. superfl. & illeg.

*Arundo sativa* Lam., Fl. Franc. (Lamarck) 3: 616. 1779, nom. superfl. & illeg. for *Arundo donax*

*Donax donax* (L.) Asch. & Graebn., Fl. Nordostdeut. Flachl. 101. 1898, nom. invalid, tautonym

**Type.** “Habitat in Hispania, Galloprovincia.” Lectotype: *Herb. A. van Royen, sheet no. 912.356-93 *(L). LT designated by Renvoize in Jarvis et al. (ed.), Regnum Veg. 127: 21. 1993.

**Distribution.** Andhra Pradesh, Arunachal Pradesh, Assam, Bihar, Delhi, Goa, Gujarat, Himachal Pradesh, Jammu and Kashmir, Karnataka, Kerala, Maharashtra, Madhya Pradesh, Manipur, Meghalaya, Nagaland, Odisha, Punjab, Rajasthan, Tamil Nadu, Tripura, Uttar Pradesh, West Bengal [Afghanistan, Bhutan, China, Myanmar, Nepal, Pakistan].

**Habit.** Tall perennial.

***Danthonidium* C.E. Hubb., Hooker’s Icon. Pl. 34: sub t. 3331. 1937**

**Type. ***Danthonidium gammiei* (Bhide) C.E. Hubb. (*Danthonia gammiei* Bhide).

**1502. **Danthonidium gammiei** (Bhide) C.E. Hubb., Hooker’s Icon. Pl. 34: t. 3331. 1937 **

*Danthonia gammiei* Bhide, J. Proc. Asiat. Soc. Bengal (n.s.) 7(8): 513, 51. t. 6. 1912 (“1911”). Type: India: Bombay, Castle rock; October, 1902; *G.A. Gammie s.n.*

**Type.** India: Bombay Presidency, Castle Rock; October, 1902; *Gammie in Herb. Econ. Bot. Bombay 15636 *(K).

**Distribution.** Karnataka, Kerala, Maharashtra.

**Habit.** Annual.

***Elytrophorus* P. Beauv., Essai Agrostor. 67. 1812**

**Type. ***Elytrophorus articulatus* P. Beauv.

**1503. **Elytrophorus spicatus** (Willd.) A. Camus, Fl. Indo-Chine [P.H. Lecomte et al.] 7: 547. 1923 **

*Dactylis spicata* Willd., Ges. Naturf. Ferunde Berlin Neue Schriften 3: 416. 1801. Type: “Wachst an der malabarischen Kuste bei Tangore auf Reifsackern und wird von den Tamulen Wajel tenei genannt.”

*Sesleria spicata* (Willd.) Spreng., Pl. Min. Cogn. Pug. 2: 21. 1815

**Distribution.** Andhra Pradesh, Assam, Bihar, Chhattisgarh, Himachal Pradesh, Gujarat, Jharkhand, Karnataka, Kerala, Madhya Pradesh, Maharashtra, Manipur, Meghalaya, Nagaland, Odisha, Rajasthan, Sikkim, Tamil Nadu, Uttar Pradesh, West Bengal [Bhutan, China, Myanmar, Nepal, Sri Lanka].

**Habit.** Annual.

***Phragmites* Adans., Fam. Pl. (Adanson) 2: 34, 559. 1763**

**Type. ***Phragmites communis* Trin. (*Arundo phragmites* L.).

**1504. **Phragmites australis** (Cav.) Trin. ex Steud., Nomencl. Bot., ed. 2 (Steudel) 1: 143. 1840 **

*Arundo australis* Cav., Anales Hist. Nat. 1: 100. 1799. Type: “Se cria en el agua y a’ la orilla del rio, que esta como media legua antes de llegar a la Bahia Botanica viniendo de Jackson alli la cogio en abril, *Don Luis Née*.”

*Phragmites communis* Trin., Fund. Agrost. (Trinius) 134. 1820

*Arundo phragmites* L., Sp. Pl. 1: 81. 1753. Type: “Habitat in Europae lacubus fluviis.” Lectotype: *Herb. Linn. No. 97.6* (LINN). LT designated by Clayton in Milne-Redhead and Polhill (ed.), Fl. Trop. E. Africa, Gramineae 1: 118. 1970.

*Phragmites australis* (Cav.) Trin. ex Steud. var. *stenophylla* (Boiss.) S.K. Jain & Doli Das, Indian Forester 99(8): 575. 1973

*Phragmites communis* Trin. var. *stenophylla* Boiss., Fl. Orient. (Boissier) 5: 563. 1884. Syntypes: in arenosis saepius maritimis vel salsas; *Heldr. s.n.* Anatolia ad Smyrnam; *Ky s.n. *Syria prope Tripoli; *Bl. s.n.* Egypt: Oasis Magna; *Scheinfurth s.n.* Assyria prope Mossul; *Ky. s.n.*, Persia borealis in monte Elbrus; *Ky s.n.* Persia borealis circa Tehran; *Haussk. s.n.*

**Distribution.** Jammu and Kashmir, Rajasthan [China, Pakistan].

**Habit.** Perennial.

**1505. **Phragmites karka** (Retz.) Trin. ex Steud., Nomencl. Bot., ed. 2 (Steudel) 2: 324. 1841 var. **karka

*Arundo karka* Retz., Observ. Bot. (Retzius) 4: 21. 1786–1787. Type: “Dedit cl. Koenig.” India?; *Koenig s.n. *(LD).

*Trichoon karka* (Retz.) Roth, Arch. Bot. [Leipzig] 1(3): 37. 1798

*Oxyanthe japonica* (Steud.) Steud., Syn. Pl. Glumac. 1: 197. 1854. Type: Japon; *Thunburg s.n.*

*Phragmites nepalensis* Nees, Syn. Pl. Glumac. (Steudel) 1: 196. 1854. Type: Nepal; *Royle 78, 79, 90*

*Arundo roxburghii* Kunth, Révis. Gramin. 1: 79. 1829. Type: India Orientalis

*Phragmites roxburghii* (Kunth) Steud., Nomencl. Bot., ed. 2 (Steudel) 2: 324. 1841

*Sericura japonica* Steud., Flora 29: 20. 1846. Type: Not given

*Phragmites vallatorius* Veldkamp, Blumea 37(1): 233. 1992, nom. superfl. & illegit.

*Arundo vallatoria* Pluk. ex L., Herb. Amb. 15. 1754, nom. invalid.

**Distribution.** Andhra Pradesh, Arunachal Pradesh, Assam, Bihar, Chhattisgarh, Goa, Gujarat, Himachal Pradesh, Jammu and Kashmir, Karnataka, Kerala, Madhya Pradesh, Maharashtra, Manipur, Nagaland, Odisha, Punjab, Rajasthan, Sikkim, Tamil Nadu, Tripura, Uttar Pradesh, West Bengal [China, Myanmar, Nepal, Pakistan, Sri Lanka].

**Habit.** Perennial.

**1506. **Phragmites karka** (Retz.) Trin. ex Steud. var. **cincta** Hook. f., Fl. Brit. India (J.D. Hooker). 7(22): 305. 1896 **

**Syntypes.** India: Near Calcutta Salt Lakes; *Kurz s.n. *Mutlah; *Clarke 24714.*

**Distribution.** Andhra Pradesh, Kerala, West Bengal.

**Habit.** Perennial.
